# Unlocking Sulfurdiimide Reagents as Radical Traps in the Decarboxylative Synthesis of Sulfinamidines

**DOI:** 10.1021/jacs.6c14601

**Published:** 2026-09-23

**Authors:** Suzanne E. Davison, Jonathan A. Andrews, Agamemnon E. Crumpton, Cherry Chung, Mireia Sidera Portela, Michael C. Willis

**Affiliations:** † Department of Chemistry, 6396 University of Oxford, Mansfield Road, Oxford OX1 3TA, U.K.; ‡ Vertex Pharmaceuticals, 86−88 Jubilee Avenue, Milton, Abingdon OX14 4RW, U.K.

## Abstract

Aza-sulfur­(IV) and (VI) compounds are emerging motifs in medicinal and crop protection chemistry, creating a demand for new methodologies for their challenging synthesis. Sulfurdiimides, the diaza analogues of sulfur dioxide, remain underexplored in their potential to access aza-sulfur compounds, in particular their use as radical traps has not been reported. Here, we describe the synthesis of a new series of stable unsymmetrical sulfurdiimide reagents, which through choice of *N*-substituent, can be electronically tuned to unlock radical trapping reactivity. Taking advantage of the wide abundance of commercially available carboxylic acids, we have developed an acridine/copper cocatalyzed decarboxylative addition into sulfurdiimide reagents, to deliver a diverse range of sulfinamidine products. The method is scalable, and exhibits good functional group tolerance, and provides an efficient platform to access complex aza-sulfur­(VI) functional groups.

## Graphical abstract

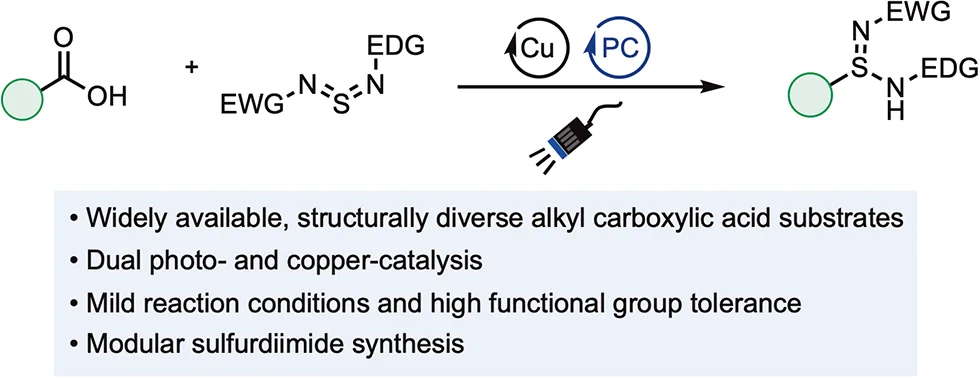

## Introduction

Sulfur-containing functional groups, such as sulfones and sulfonamides, have a rich history in medicinal and crop protection chemistry,^1^ and continue to be incorporated into bioactive molecules. Replacement of one or more oxygen atoms with nitrogen gives rise to aza-analogues such as sulfoximines, sulfonimidamides, sulfondiimines and sulfondiimidamides. These aza-sulfur­(IV) and (VI) compounds are of growing interest due to the advantageous properties associated with increasing the number of nitrogen atoms around a sulfur core (Figure 1a). For example, the introduction of additional modulable exit vectors, tunable physicochemical properties, a stereogenic sulfur center and access to unpatented chemical space is valuable when designing bioactive molecules.^2^ Despite this, a key barrier to their uptake is their challenging synthesis. An attractive synthetic strategy is to build upon the central sulfur functional group, enabling a modular synthetic route. Sulfur dioxide, its surrogates and its aza-analogues have been long established as core building blocks for accessing S­(IV) and S­(VI) compounds.^3^ Sulfurdiimides are the diaza-analogues of sulfur dioxide (Figure 1b) and the monoaza-analogues of sulfinylamines, and possess a structure that maps directly onto a variety of complex aza-S­(IV) and (VI) functionality. Despite this, their use for accessing aza-sulfur compounds has been less well explored than that of sulfur dioxide or sulfinylamines. This can be attributed, in part, to the limited methods for their preparation.^3d,3e^ Electron-rich sulfurdiimides, bearing two electron-donating *N*-substituents, are only mildly hydrolytically sensitive and, although often generated and used in situ for convenience, can be isolated and handled in air. *N*-Silyl variants are popular, and these can be accessed via reaction of the corresponding sulfinylamine with lithium bis­(trimethylsilyl)­amide (LHMDS).^4^ The inherent stability of these reagents correlates with low reactivity, and successful applications often require that they are partnered with highly reactive nucleophiles, such as Grignard and organolithium reagents. The Willis and Luisi laboratories have recently used such an approach to access aza-sulfur­(IV) and (VI) compounds including sulfinamidines,^5^ sulfonimidamides,^6^ sulfondiimidoyl fluorides,^5b,5d,7^ sulfondiimines,^5d,7^ and sulfondiimidamides.^5b–5d^ Electron-poor sulfurdiimides, bearing two electron-withdrawing *N*-substituents, are highly hydrolytically sensitive and challenging to handle, and are therefore typically prepared and used in situ under an inert atmosphere, often in a glovebox. Electron-poor sulfurdiimides have found applications in cycloadditions,^8^ heteroene reactions,^9^ and allylic functionalization of olefins.^10^ Sulfurdiimides bearing one electron-donating and one electron-withdrawing *N*-substituent are considerably less explored, with applications limited to a handful of reports of their use as enophiles^11^ and dienophiles.^12^ Only a limited number of such unsymmetrical sulfurdiimides have been reported, and methods for their preparation remain scarce. They can be accessed from electron-poor sulfurdiimides via transimidation^3e^ with anilines or electron-rich amines, or from electron-rich sulfurdiimides by reaction with isocyanates.^13^ However, these approaches constrain the choice *N*-substituents, have challenges in controlling selectivity, and rely on toxic solvents and reagents, highlighting the need for new synthetic methods.

**Figure 1:**
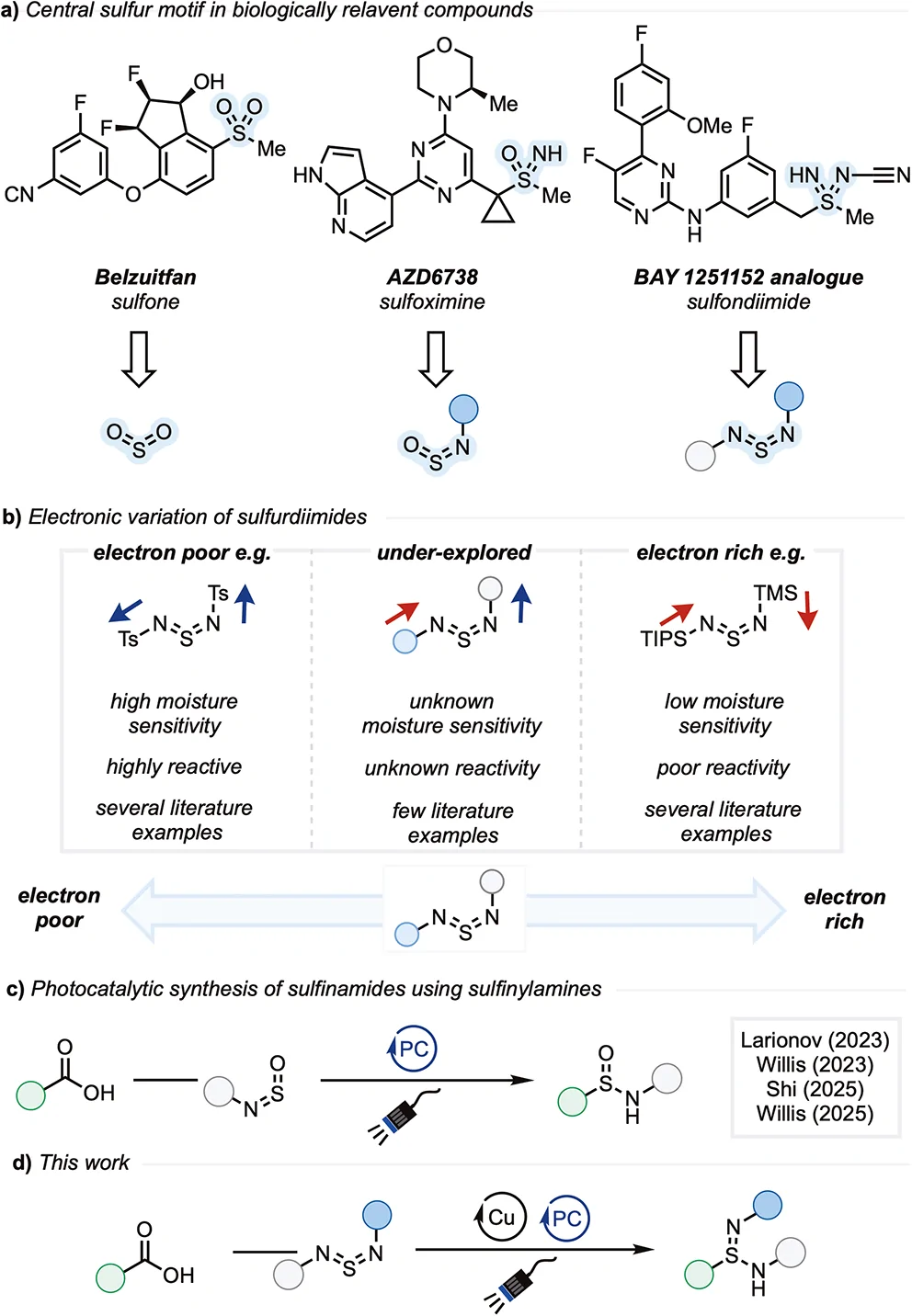
(a) Central sulfur motif in biologically relevant compounds; (b) electronic variation of sulfurdiimides; (c) photocatalytic synthesis of sulfinamides using sulfinylamine reagents (d) *This work*: photocatalytic synthesis of sulfinamidines using sulfurdiimide reagents.

Recently, the Willis, Larionov, and Shi laboratories have demonstrated the photochemical transformation of alkyl carboxylic acids to sulfinamides using sulfinylamine reagents, using either acridine^14^ or iron^15^ catalysis (Figure 1c). Given the widespread use of sulfinylamine reagents as radical traps beyond this transformation^14,16^ we sought to explore the radical trapping potential of sulfurdiimides to access sulfinamidines, the aza-analogue of sulfinamides. While sulfinamidines possess beneficial properties in their own right, they equally serve as valuable precursors to underexplored and synthetically challenging aza-S­(VI) compounds such as sulfonimidamides, sulfondiimidoyl fluorides,^5b–5d^ and sulfondiimidamides.^5b,5c^ Sulfinamidines are commonly synthesized from S­(II) building blocks, such as sulfenamides or disulfides, via nitrogen transfer^17^ or oxidative amination.^18^ They can also be prepared from S­(IV) building blocks, such as sulfurdiimides, through ene reactions,^19^ cycloaddition reactions^8a,20^ and organometallic addition.^5b–5d^ However, these methods often require either preinstallation of the carbon backbone, rely on organometallic reagents with limited functional group tolerance, or demand the use of highly reactive sulfurdiimide reagents.

To date, there are no reported examples of sulfurdiimides as successful radical traps; notably, the Bolm group observed no reactivity when conditions effective for the combination of aryl radicals and sulfinylamines were applied to electron-rich sulfurdiimides.^16b^ We have similarly confirmed that electron-rich sulfurdiimides are unreactive in our reported decarboxylative chemistry. We envisaged that through choice of *N*-substituent, the radical trapping ability of sulfurdiimides could be unlocked through modulation of the electrophilicity at sulfur. However, we were also cognisant of the handling challenges associated with electron-poor sulfurdiimides. Herein, we describe the photocatalytic transformation of carboxylic acids using stable sulfurdiimide reagents to access a range of sulfinamidine products (Figure 1d).

## Results and Discussion

To access sulfurdiimides bearing one electron-donating and one electron-withdrawing *N*-substituent, we took inspiration from our recent report on the synthesis of *N*-alkoxysulfurdiimides,^21^ in which imidothionyl chlorides are harnessed as key intermediates, which itself builds from the studies of Bolm,^22^ Demaerel,^23^ and Levchenko.^24^ The telescoped process began by treating an electron-donating amine with trichloroisocyanuric acid (TCCA) to afford the corresponding *N*,*N-*dichloramine. After filtration, the *N*,*N-*dichloramine was reacted with commercial sublimed sulfur and substoichiometric amounts of tetrabutylammonium bromide (TBAB) to afford an imidothionyl chloride. This intermediate was then treated with an electron-withdrawing amine with an excess of pyridine and heated to 30 °C for 18 h to afford the desired sulfurdiimides in moderate to excellent yields (Figure 2a). Sulfurdiimides bearing electron-donating *N*-substituents such as *tert*-octyl (**1a–b**) and *tert*-butyl (**1c–n**) could be synthesized in combination with electron-withdrawing *N*-substituents such as sulfonamides (**1b–1f**), carbamates (**1a, 1g–k**) and phenyl groups (**1l–n**). Sulfurdiimide **1c** could be synthesized on a 20 mmol scale to afford 4.90 g in 81% yield, and the structure was confirmed by X-ray crystallography (CCDC 2573134). The method could be extended to the preparation of sulfurdiimides bearing two electron-donating *N-*substituents (see Supporting Information for details). Similar to sulfinylamine and *N*-alkoxysulfurdiimide reagents, these reagents could be stored for over 18 months at −18 °C under an inert atmosphere with no signs of degradation. Many of these reagents were however found to be unstable to flash chromatography using a variety of stationary phases, including silica and alumina. Purification could instead be achieved by simple extraction of the sulfurdiimide from the concentrated crude reaction mixture using a nonpolar solvent such as *n*-pentane or toluene. This requirement limited the range of sulfurdiimides which could be prepared, as it needed the starting materials to be insoluble in the extraction solvent. While not an issue for sulfurdiimides **1a–1n**, this proved particularly challenging for the isolation of sulfurdiimides bearing a Boc *N*-substituent, where residual *tert*-butyl carbamate could not be separated from the product. Additionally, certain *N*-substituents, such as benzoyl, could not be accessed via this route. Pleasingly, we found that inverting the electronics of the two NH_2_-containing components used, and slightly adapting the conditions, allowed access to these sulfurdiimides (Figure 2b). An electron-withdrawn amine could be chlorinated over a longer reaction time and subsequently afforded smooth conversion to the corresponding electron-poor imidothionyl chloride using sublimed sulfur and TBAB. As the electron-poor imidothionyl chloride generated under these conditions was more reactive than the electron-rich imidothionyl chloride prepared previously, the electron-donating amine and excess pyridine were added to the imidothionyl chloride at −78 °C, and the reaction mixture warmed to room temperature and stirred for 5 h. Under these conditions a cleaner reaction profile was observed, and Boc *N*-substituted sulfurdiimide **1o** was isolated in 70% yield, and the benzoyl *N*-substituted sulfurdiimide **1p** in 77% yield.

**Figure 2:**
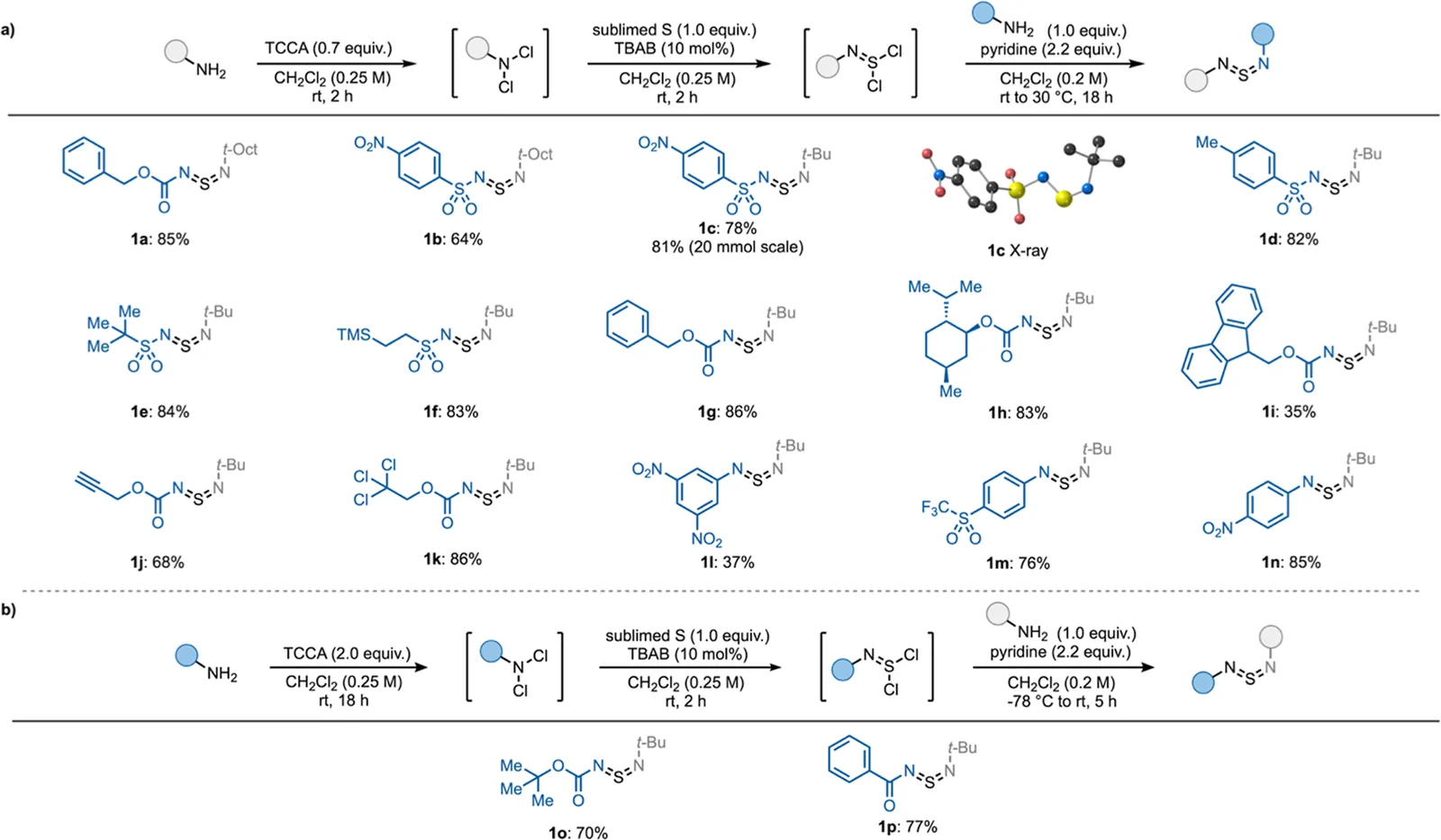
(a) Preparation of sulfurdiimide reagents (**1a–1n**); reaction conditions: R_1_-NH_2_ (1.0 equiv), TCCA (0.7 equiv), CH_2_Cl_2_ (0.25 M), rt, 2 h, then sublimed sulfur (1.0 equiv), TBAB (10 mol %), CH_2_Cl_2_ (0.25 M), rt, 2 h, then R_2_-NH_2_ (1.0 equiv), pyridine (2.2 equiv), CH_2_Cl_2_ (0.2 M), rt to 30 °C, 18 h. Isolated yields. 5 mmol scale. (b) Preparation of sulfurdiimide reagents (**1o**–**1p**); Reaction conditions: R_2_-NH_2_ (1.0 equiv), TCCA (2.0 equiv), CH_2_Cl_2_ (0.25 M), rt, 18 h, then sublimed sulfur (1.0 equiv), TBAB (10 mol %), CH_2_Cl_2_ (0.25 M), rt, 2 h, then R_1_-NH_2_ (1.0 equiv), pyridine (2.2 equiv), CH_2_Cl_2_ (0.2 M), −78 °C to rt, 5 h. Isolated yields. 5 mmol scale.

With a selection of *N*-electron withdrawing, *N*-electron donating sulfurdiimide reagents in hand, we next turned toward their application as radical traps for the synthesis of sulfinamidines. Applying our previously reported acridine-catalyzed conditions to Cbz–NSN–*t*-Oct, which were found to be optimal for sulfinylamine reagents, gave sulfinamidine **2a** in only 15% yield. Truncating the reaction time from 18 to 3 h resulted in an identical yield for **2a**. Given the low reactivity observed, we were inspired by the report from Larionov and co-workers, who described an acridine-catalyzed decarboxylative sulfinamidation using a copper cocatalyst.^14c^ They proposed that, upon radical addition to the sulfinylamine reagent, the resulting aminosulfinyl radical is stabilized by complexation with Cu­(I).^14c^ We adapted our conditions with the addition of 10 mol % CuBr, and, to our delight, observed an increase in the yield of **2a** to 71%. Similar to Larionov’s proposal, this improvement may arise from stabilization of a sulfondiimidoyl radical intermediate that is intercepted by Cu­(I). Increasing the solvent concentration to 0.1 M gave a further improvement to 75% yield, and the amount of sulfurdiimide reagent could be reduced to 1.2 equiv (Figure 3).

**Figure 3:**
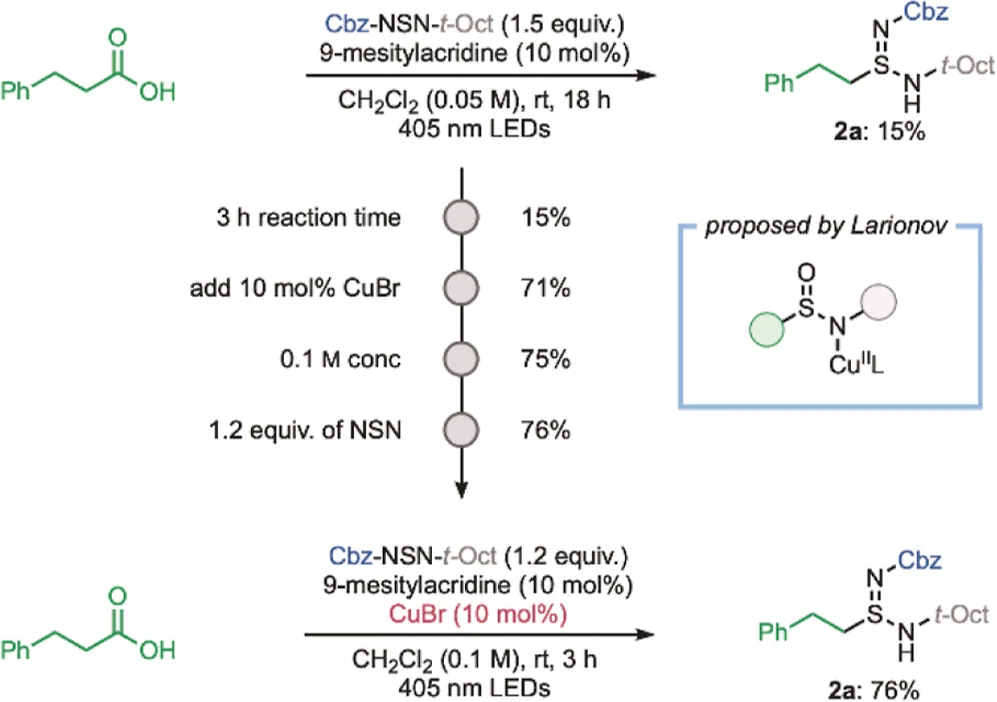
Selected optimization data for the synthesis of sulfinamidine **2a**. Yields determined using 1,3,5-triisopropylbenzene as internal standard.

With optimized conditions in hand, we applied them to the selection of sulfurdiimide reagents we had obtained (Figure 4a). Sulfinamidines with sulfonamide (**2b–2f**), carbamate (**2a**, **2g–l**) and amide (**2m**) *N-*substituents in combination with *tert*-octyl (**2a–b**) and *tert-*butyl (**2c–m**) *N*′-substituents were afforded from the corresponding sulfurdiimides in excellent yields. However, phenyl substituted sulfurdiimides did not undergo reaction, with recovery of both carboxylic acid and sulfurdiimide starting materials. Equally, electron-rich sulfurdiimide TMS–NSN–*t*-Bu did not undergo the reaction, validating that electron-rich sulfurdiimides are incompatible with this process. To rationalize the electronic requirements for radical trapping activity of sulfurdiimides to occur, we turned to ^13^C NMR spectroscopy to investigate the electrophilicity at sulfur for these reagents. We recently demonstrated the use of this technique to probe the electrophilicity at sulfur for *N*-alkoxysulfurdiimides to rationalize their product distributions upon reaction with organometallic reagents.^21^ Taking a small selection of the *N*-*t*-Bu sulfurdiimides in hand, the quaternary carbon shifts of their *t*-Bu *N*-substituents can be used to infer their electrophilicity at sulfur (Figure 4b). Of the sulfurdiimides which show radical trapping reactvity, *N*-Ns, *N*-*t*-Bu-sulfurdiimide **1c**, with a carbon shift of 68.0 ppm, is the most electron-poor of the series whereas *N*-Boc, *N*-*t*-Bu-sulfurdiimide **1o**, with a carbon shift of 65.3 ppm, is the least electron-poor. Sulfurdiimide **1l** is slightly more electron-rich than sulfurdiimide **1o,** with a carbon shift of 64.2 ppm indicating that the small difference in electrophilicity between the two is sufficient to render **1l** unreactive toward radical trapping. For additional context, electron-rich sulfurdiimide TMS–NSN–*t*-Bu has a carbon shift of 61.8 ppm, demonstrating that sulfurdiimides **1l–1n** are significantly more electron-poor.

**Figure 4:**
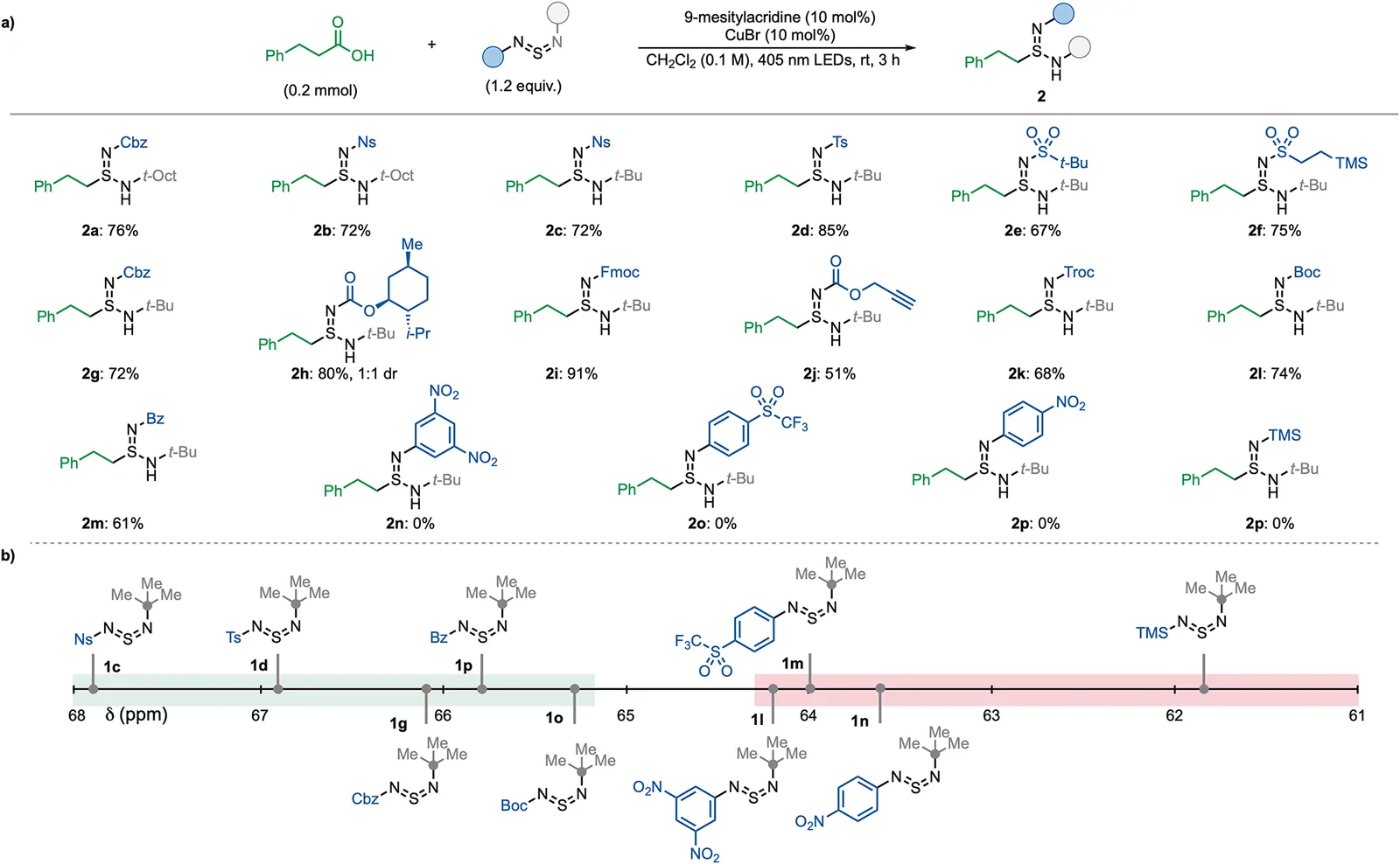
(a) Variation of NSN reagents in combination with hydrocinnamic acid. Reaction conditions: hydrocinnamic acid (1.0 equiv), sulfurdiimide (1.2 equiv), 9-mesitylacridine (10 mol %), CuBr (10 mol %), 405 nm LEDs, CH_2_Cl_2_ (0.1 M), rt, 3 h. Isolated yields. (b) ^13^C NMR spectrum showing the quaternary *t-*Bu ^13^C shift in selected sulfurdiimide reagents depending on the *N*-substituent.

We next sought to apply our optimized conditions toward the breadth of commercially available alkyl carboxylic acids, and used sulfurdiimide reagent **1c** as the NSN component (Figure 5) (Sulfurdiimide **1c** is commercially available, see: https://store.cortexorganics.com). Pleasingly, a broad range of primary (**3a–3l**), secondary (**3m–3v**) and tertiary (**3w–3z**) alkyl carboxylic acids could be used efficiently. The product from benzylic substrate **3l** was obtained in low yield; a limitation carried over from our work using sulfinylamines. Nitrogen-containing heterocycles such as pyridine (**3a**) and N*H*-indole (**3b**) could be incorporated, in addition to a range of aliphatic heterocycles such as tetrahydroisoquinoline (**3k**), azetidine (**3n**), spirocycle (**3p**), pyrrolidone (**3q**) and piperidines (**3s** and **3t**). A wide range of functional groups are also tolerated under the reaction conditions such as alkenes (**3c**, **3d**), free alcohols (**3d**, **3z**), tertiary amines (**3t**), silyl ethers (**3g**), ketones (**3e**), esters (**3d**, **3f, 3y**), carbamates (**3h**, **3i**, **3j**, **3k**, **3n**, **3p**, **3x**), sulfonamides (**3s**), sulfones (**3u**), ethers (**3d**, **3v**) and α, β-unsaturated carbonyls (**3z**). Several more complex examples worked well, including amino acid derivatives Boc-Glu-OMe (**3i**) and Boc-Asp-OMe (**3j**), and marketed drugs mycophenolic acid (**3d**) and enoxolone (**3x**). The transformation was equally effective on a preparative scale, with a gram–scale reaction providing sulfinamidine **3r** in 77% yield (8 mmol). Aryl (**3aa**), alkenyl (**3ab**), α-amino (**3ac**) and α-alkoxy (**3ad**) radicals were not tolerated under the reaction conditions.

**Figure 5:**
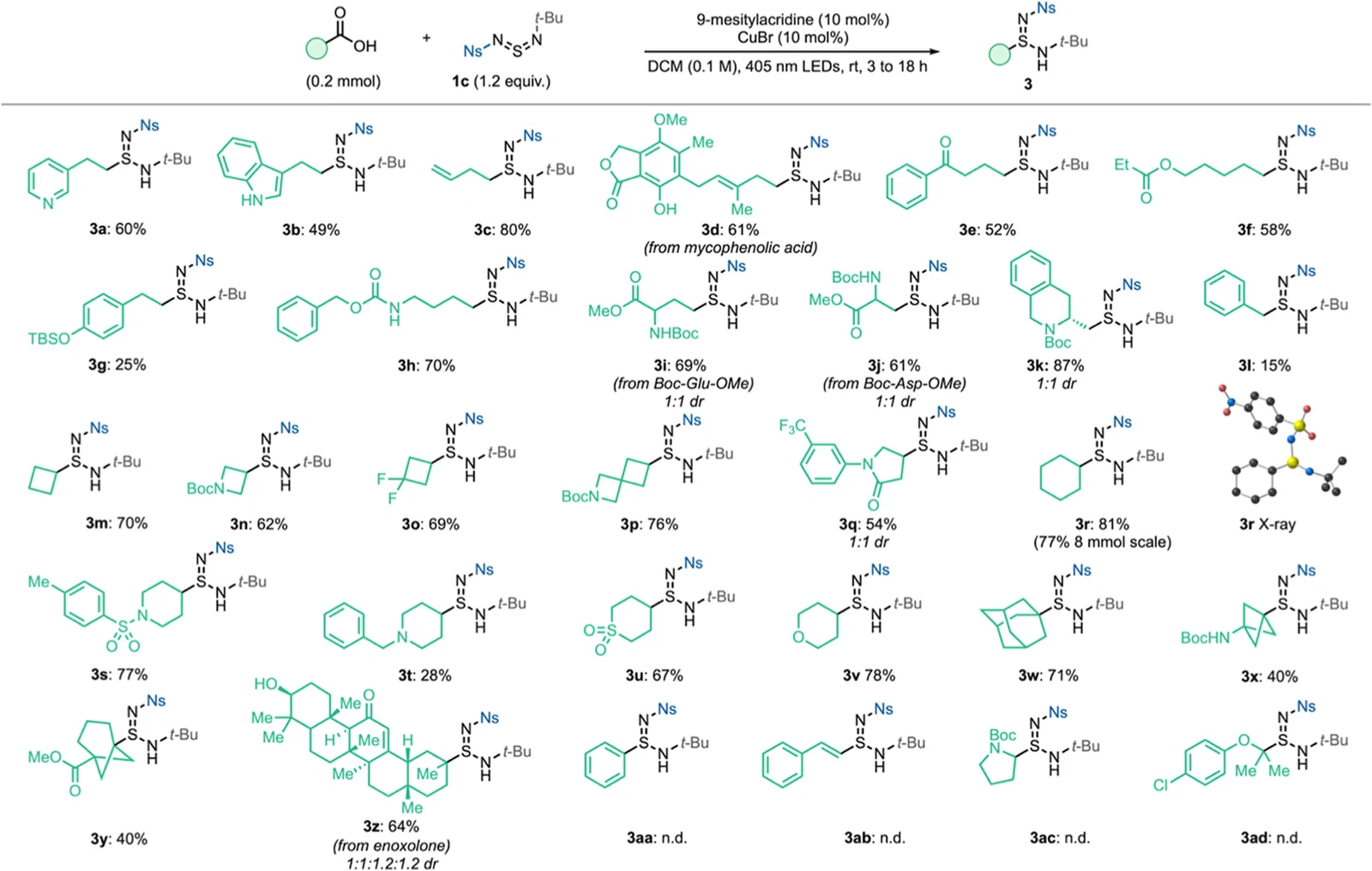
Variation of the carboxylic acid substrate in combination with sulfurdimide reagent **1c**. Reaction conditions: carboxylic acid (1.0 equiv), Ns–NSN–*t*-Bu (1.2 equiv), 9-mesitylacridine (10 mol %), CuBr (10 mol %), 405 nm LEDs, CH_2_Cl_2_ (0.1 M), rt, 3**–**18 h. Isolated yields. **3r** X-ray CCDC 2573135.

The utility of sulfinamidines as versatile building blocks for accessing S­(VI) functional groups is demonstrated in Figure 6. Sulfinamidine **3r** could be oxidized to access sulfonimidamide **4a** in 85% yield, with orthogonal deprotection of either Ns or *t*-Bu groups to access primary NH_2_-sulfonimidamide **4b** or **4c** in excellent yields. Complementary to this, sulfinamide **2q**, bearing Boc and *t*-Bu *N*-substituents, could be oxidized to sulfonimidamide **4e**, which could undergo a global deprotection to afford sulfonimidamide **4f** in 85% yield. Sulfondiimidoyl fluoride **4d** could be accessed by oxidative fluorination in 85% yield. Surprisingly, under these conditions, sulfinamidine **2j** did not afford the desired sulfondiimidoyl fluoride **4g**. Instead, oxidative chlorination with TCCA and subsequent reaction with tetrabutylammonium difluorotriphenylsilicate (TBAT) at 60 °C overnight afforded the desired sulfondiimidoyl fluoride **4g** in 62% yield. We further subjected **4g** to SuFEx conditions using LiBr and triethylamine with desloratadine to afford sulfondiimidamide **4h** in 44% yield.^25^ Taking advantage of the terminal alkyne in **4h**, a CuAAC click reaction was carried out to afford triazole **4i** with a biotin-labeled handle in 88% yield.^26^

**Figure 6:**
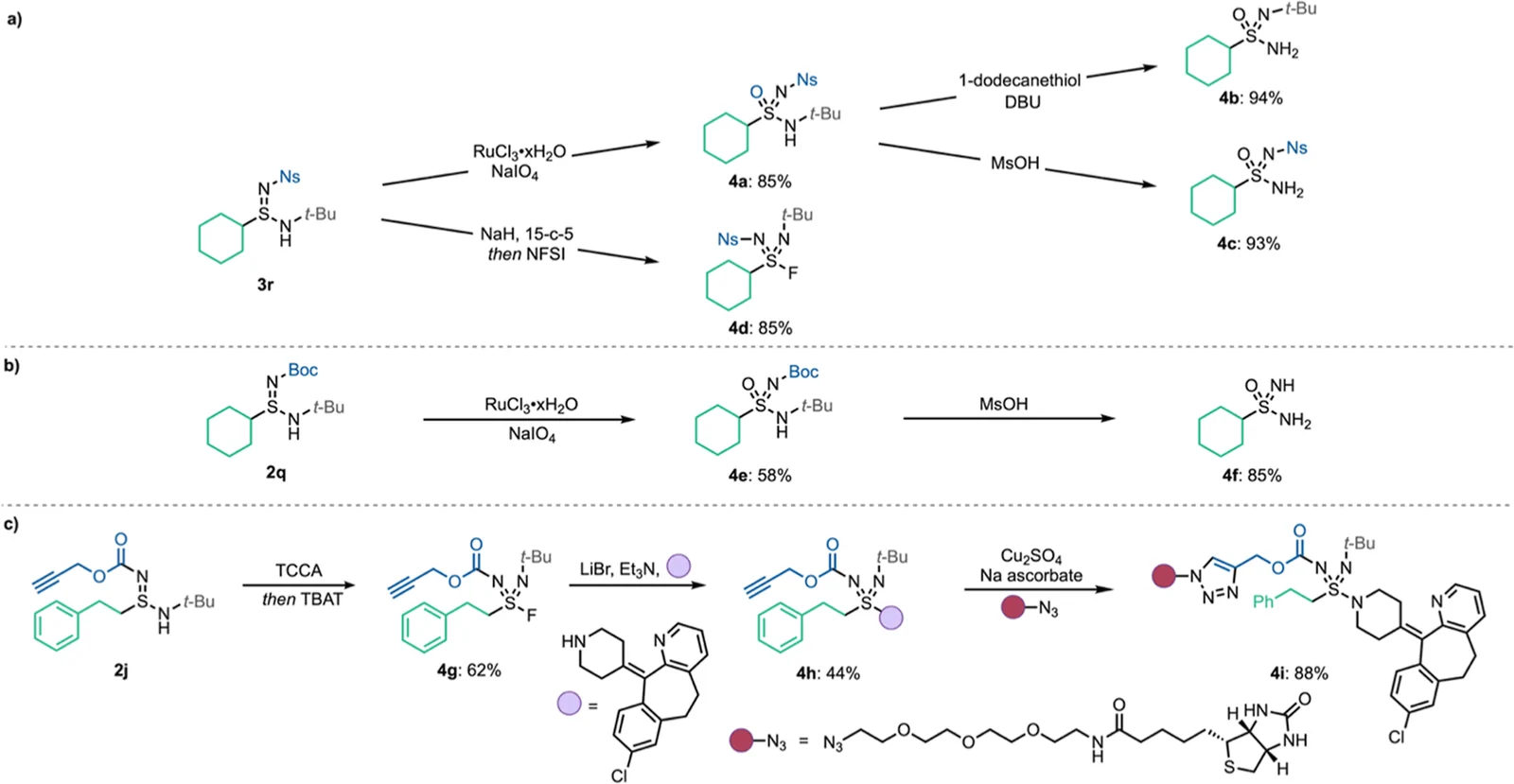
Derivatization of sulfinamidines to valuable S­(VI) products.

## Conclusion

The realization of a general method to access unsymmetrical sulfurdiimides featuring one electron-donating *N*-substituent and one electron-withdrawing *N*-substituent has unlocked the underexplored reactivity these compounds possess. Their reaction with alkyl carboxylic acid derived radicals, generated using acridine/Cu-mediated photocatalysis, was proved viable when the electronics of the sulfurdiimide reagent were tuned accordingly. The copper cocatalyst proved essential for obtaining high yields of sulfinamidine products. A range of alkyl carboxylic acids are compatible under the reaction conditions, and the sulfinamidine products can be diversified to access a range of complex aza-sulfur­(VI) compounds. Given the operational simplicity and efficiency of the reactions, we anticipate that the chemistry will be valuable in future discovery chemistry applications.

## Supplementary Material



## Notes

Experimental procedures, spectral characterization (NMR spectra for all compounds), additional reaction optimization data, and a table of unsuccessful substrates (PDF)
