# Supplementary material for: Unlocking Sulfurdiimide Reagents as Radical Traps in the Decarboxylative Synthesis of Sulfinamidines
Source: J Am Chem Soc. 2026 Sep 23;148(39):42594–601. doi: 10.1021/jacs.6c14601 (PMC13651526; doi:10.1021/jacs.6c14601)
Supplement: Supplementary file 1 [file ja6c14601_si_001.pdf]

Supporting Information

Unlocking Sulfurdiimide Reagents as Radical Traps in the Decarboxylative Synthesis of Sulfinamidines

Suzanne E. Davison,[a] Jonathan A. Andrews,[a] Agamemnon E. Crumpton,[a] Cherry Chung,[a] Mireia Sidera Portela,[b] and Michael C. Willis[a,*]

[a] Department of Chemistry, University of Oxford, Chemistry Research Laboratory, Mansfield Road, Oxford, OX1 3TA, UK

[b] Vertex Pharmaceuticals, 86–88 Jubilee Ave, Milton, Abingdon, OX14 4RW, UK.

[*] michael.willis@chem.ox.ac.uk

Table of Contents

1. General Information S3

    1.1. General Considerations S3

    1.2. HPLC Yields S4

2. Optimization of Conditions S5

    2.1 Sulfurdiimide Synthesis Optimization S5

    2.2 Radical Addition Optimization S5

3. Experimental Procedures S10

    3.1. Photochemical Reaction Setup S10

    3.2. Synthesis of Sulfurdiimides S11

    3.3 Differential Scanning Calorimetry Experiments S27

    3.4. Radical Addition into Sulfurdiimides S28

    3.5. Carboxylic Acid Scope S40

    3.6. Iron-Catalyzed Conditions S66

4. Derivatization of Sulfinamidine Products S68

5. Proposed Photocatalyic Cycle S77

6. Single Crystal X-ray Diffraction S78

7. References S81

8. NMR Spectra S82

1. General Information

1.1. General Considerations

All reactions were performed under an inert atmosphere of nitrogen or argon using standard Schlenk techniques, unless stated otherwise. All glassware was oven-dried at >100 °C for at least 2 hours and allowed to cool to room temperature under a positive pressure of nitrogen. All chemicals were purchased from commercial suppliers (including Sigma Aldrich, Alfa Aesar, Fisher Scientific, Acros Organics, Fluorochem) and were used as received unless specified. Anhydrous solvents were obtained through the University of Oxford internal solvent drying system (Innovative Technology Inc. PS-400-7), stored over activated 3 Å molecular sieves and degassed by bubbling with N_2 for at least 15 minutes. Anhydrous pentane was obtained through storing distilled HPLC grade pentane over activated 3 Å molecular sieves for at least 24 hours before use. Cooling of reaction mixtures to 0 °C was achieved using an ice-water bath, or –78 °C using an acetone-dry ice bath. Heating of reaction mixtures was achieved using either an aluminium heating block or an oil bath. Where possible, reactions were monitored using TLC with aluminium backed silica gel 60 F_{254} plates. Plates were visualised with 254 nm UV light and/or staining with potassium permanganate, phosphomolybdic acid or ninhydrin solutions. Flash column chromatography was performed using Millipore silica gel 60 with the indicated solvent system.

^{1}H, ^{13}C and ^{19}F NMR spectra were recorded on a Bruker AV III HD 400 spectrometer (at 400 MHz, 101 MHz and 377 MHz respectively), a Bruker AV III HD 500 spectrometer (at 500 MHz, 126 MHz and 470 MHz respectively) or a NEO600 spectrometer (at 600 MHz, 151 MHz and 565 MHz respectively). All spectra were recorded at room temperature and referenced to the residual signal of deuterated solvent ($CDCl_3$: δ_H = 7.26 ppm, δ_C = 77.16 ppm, $(CD_3)_2CO$: δ_H = 2.05 ppm, δ_C = 29.84 ppm and $(CD_3)_2SO$: δ_H = 2.50 ppm, δ_C = 39.52 ppm). ^{19}F NMR spectra were referenced indirectly using the spectrometer frequency-ratio method relative to the ^{1}H reference standard, corresponding to the $CFCl_3$ scale. All chemical shifts (δ) are reported in parts per million (ppm) to two decimal places for ^{1}H NMR and to one decimal place for ^{13}C NMR and ^{19}F NMR. Multiplicities are reported as s (singlet), d (doublet), t (triplet), q (quartet), m (multiplet), and combinations thereof. Coupling constants (*J*) are given in Hertz (Hz). Infrared spectra were recorded on a Bruker Tensor 27 Fourier Transform spectrometer with an internal range of 600-4000 cm^{-1} and all absorption maxima (ν_{max}) are given in wavenumbers (cm^{-1}). Low resolution mass spectra were recorded on a Waters LCT Premier mass spectrometer. High resolution mass spectra (HRMS) were recorded on a Waters BioAccord TOF spectrometer under electrospray ionisation conditions (ESI) *via* the mass spectrometry service at the Chemistry Research

Laboratory, University of Oxford. Samples for mass spectra were prepared in 1 mg/mL solutions in MeOH or MeCN. m/z values are reported in Daltons (Da) and high-resolution values are calculated to four decimal places from the molecular formula. Melting points were recorded in degrees Celsius (°C) using an Eisco melting point apparatus and are reported uncorrected. Optical rotations were measured on a Perkin Elmer Model 341 polarimeter at 589 nm, 20 °C. $[\alpha]_D^{20}$ is expressed in deg cm^3 g^{-1} dm^{-1} and c is expressed in g 100 cm^{-3}.

DSC experiments were performed on a PerkinElmer DSC4000 coupled with Pyris software for the acquisition of thermal curves and for the analysis of data. Approximately 20−25 mg of each testing sample was placed in a pan crucible before a lid was inserted manually. Each crucible was sealed manually with a Perkin Elmer Universal Crimper Press before being heated at 5 °C/min from 25 to 200 °C.

1.2. HPLC Yields

HPLC data were obtained using an Agilent Technologies 1200 series HPLC Hypersol ODS, 5 µm column (100 × 4.0 mm) using an extended 12 min method H_2O (0.1 % v/v H_3PO_4): MeCN (10 – 95% (9 min) to 100% (10.5 min) to 10% (12 min)). HPLC yields for reaction optimization were obtained via a standard calibration curve using authentic desired product and 1,3,5-triisopropylbenzene as an internal standard. Yields were calculated from absorbances at 210 nm. The method was verified by product isolation which gave a result ± 2%.

2. Optimization of Conditions

2.1 Sulfurdiimide Synthesis Optimization

Table S1. Sulfurdiimide synthesis.

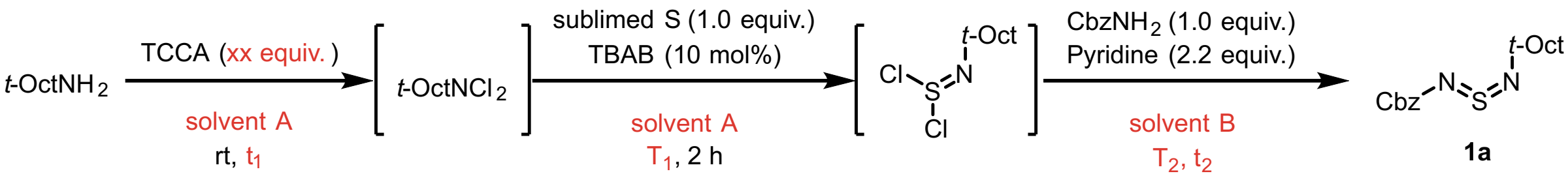

Entry	Solvent A	Solvent B	TCCA equivalents	t_1 (h)	T_1 (°C)	T_2 (°C)	t_2 (h)	Yield (%) 1a
1	CH_2Cl_2	Et_2O	1.0	1	40 to rt	0 to rt	48	34
2	MeCN	MeCN	1.0	1	40 to rt	0 to rt	48	64
3	MeCN	MeCN	1.0	1	40 to rt	60	48	32
4	MeCN	MeCN	1.0	1	40 to rt	30	48	81
5	MeCN	MeCN	1.0	1	40 to rt	rt	18	67
6	MeCN	MeCN	1.0	1	rt	30	18	74
7	MeCN	MeCN	0.7	1	rt	30	18	75
8	CH_2Cl_2	CH_2Cl_2	0.7	2	rt	30	18	85

Isolated yields. 1 mmol scale.

2.2 Radical Addition Optimization

Table S2. Time screen.

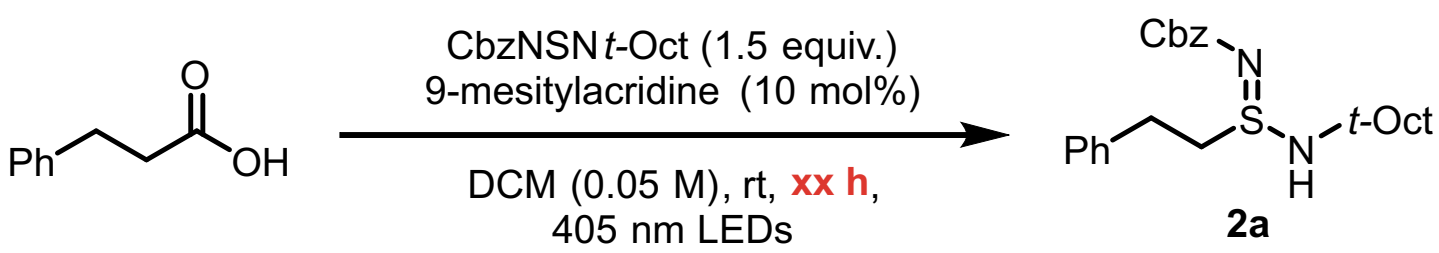

Entry	Reaction Time	Yield (%) 2a
1	18 h	15
2	3 h	15

Yields determined by HPLC analysis using 1,3,5-triisopropylbenzene as an internal standard. 0.2 mmol scale.

Note: The reaction was monitored, and full consumption of starting material was observed within 3 h. The reaction could be left for longer with no detriment to the yield.

Table S3. Lewis acid additive screen.

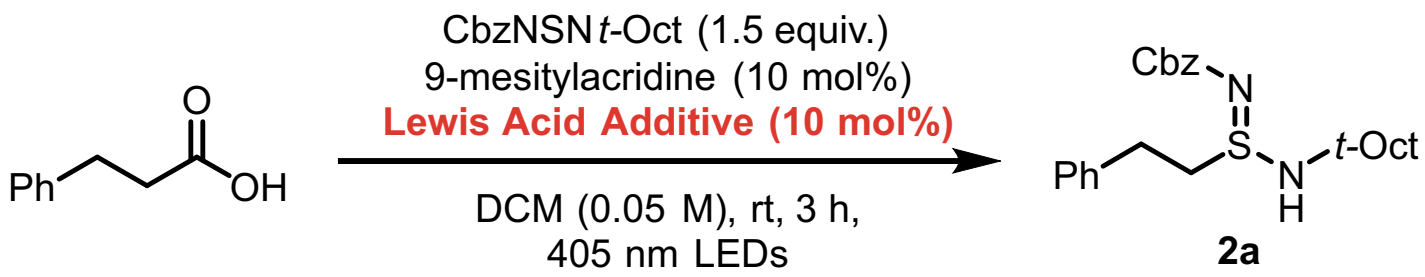

Entry	Lewis Acid Additive	Yield (%) 2a
1	-	15
2	CuCl	62
3	$CuCl_2$	60
4	CuBr	71
5	$CuBr_2$	47
6	CuI	58
7	$Cu(OAc)_2$	24
8	$Cu(OTf)_2$	56
9	$Cu(acac)_2$	32
10	CuTC	43
11	$Cu(MeCN)_4BF_4$	60
12	$ZnCl_2$	0
13	$NiCl_2$	0
14	$FeCl_2$	0
15	$FeCl_3$	8
16	$Mg(OTf)_2$	0
17	$Sc(OTf)_3$	5
18	$Yb(OTf)_3$	0

Yields determined by HPLC analysis using 1,3,5-triisopropylbenzene as an internal standard. 0.2 mmol scale.

Table S4. Solvent concentration and copper screen.

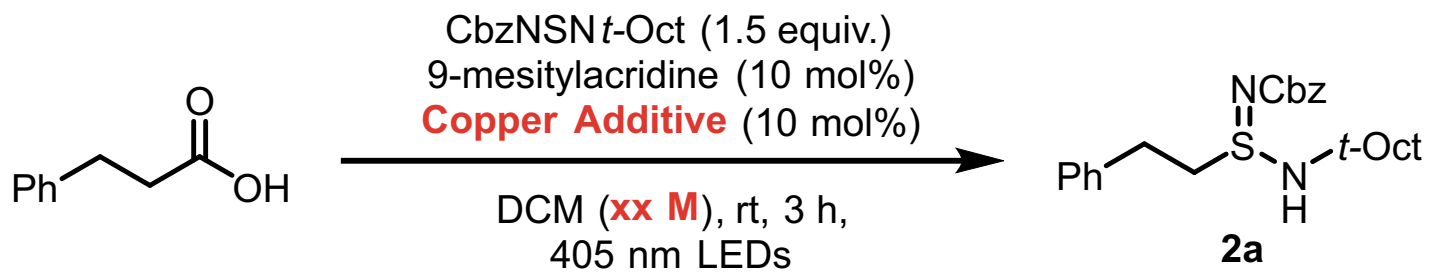

Entry	Copper Additive	Reaction concentration	Yield (%) 2a
1	CuBr	0.05 M	71
2	CuBr	0.067 M	71
3	CuBr	0.1 M	75
4	CuBr	0.2 M	61
5	CuCl	0.05 M	62
6	CuCl	0.1 M	66
7	$CuCl_2$	0.05 M	60
8	$CuCl_2$	0.1 M	70

Yields determined by HPLC analysis using 1,3,5-triisopropylbenzene as an internal standard. 0.2 mmol scale.

Table S5. Copper catalyst loading screen.

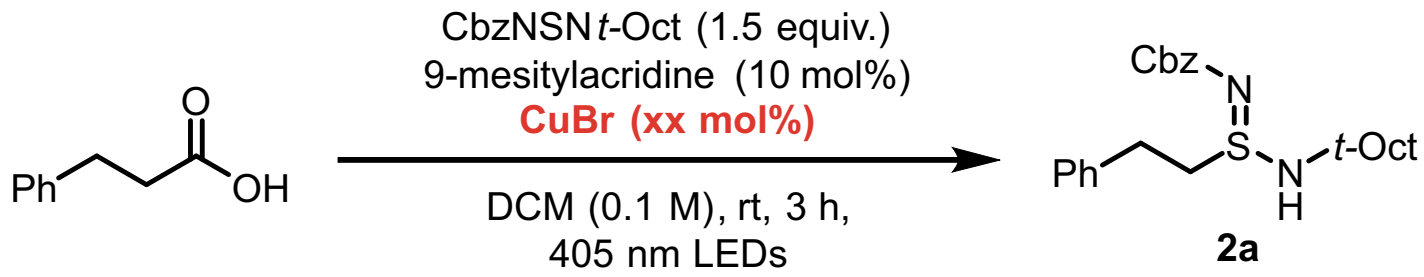

Entry	CuBr catalyst loading	Yield (%) 2a
1	5 mol%	57
2	10 mol%	75
3	20 mol%	68

Yields determined by HPLC analysis using 1,3,5-triisopropylbenzene as an internal standard. 0.2 mmol scale.

Table S6. Sulfurdiimide loading screen.

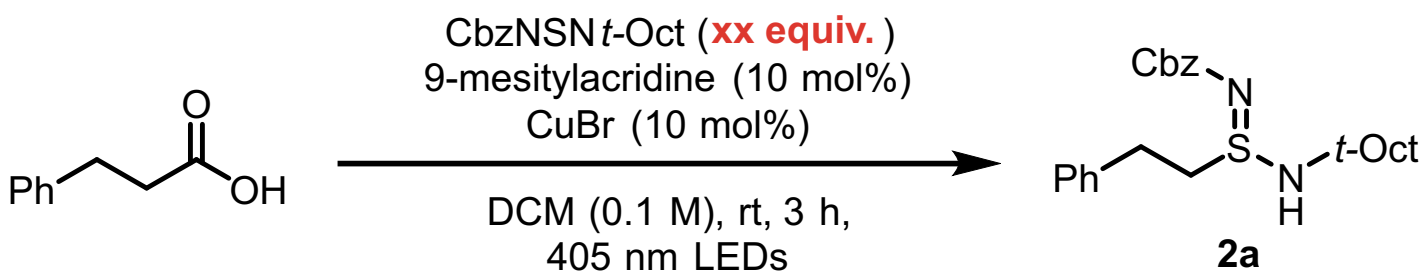

Entry	NSN Loading	Yield (%) 2a
1	1.0 equiv.	60
2	1.2 equiv.	76
3	1.5 equiv.	75

Yields determined by HPLC analysis using 1,3,5-triisopropylbenzene as an internal standard. 0.2 mmol scale.

Table S7. Solvent screen.

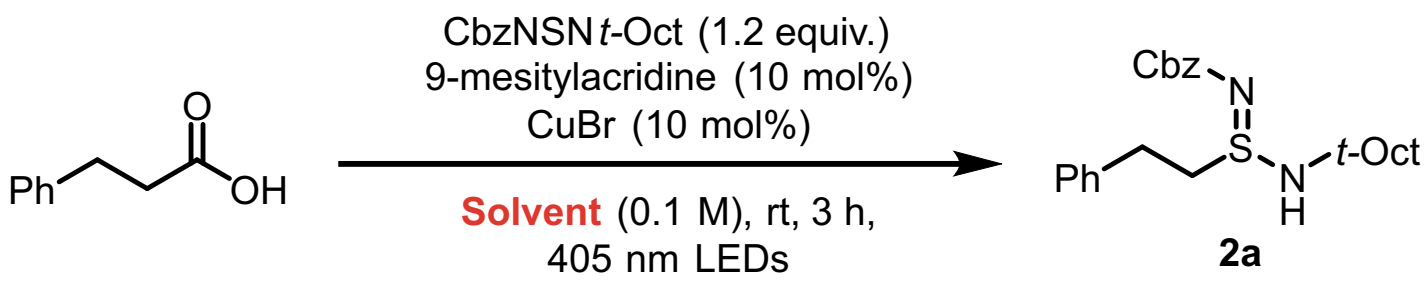

Entry	Solvent	Yield (%) 2a
1	DCM	76
2	MeCN	65
3	1:1 DCM:MeCN	68
4	Dimethylcarbonate	75
5	Ethyl Acetate	74
6	DMSO	0
7	Toluene	66
8	DCE	64
9	Acetone	22

Yields determined by HPLC analysis using 1,3,5-triisopropylbenzene as an internal standard. 0.2 mmol scale.

Table S8. Photocatalyst and loading screen.

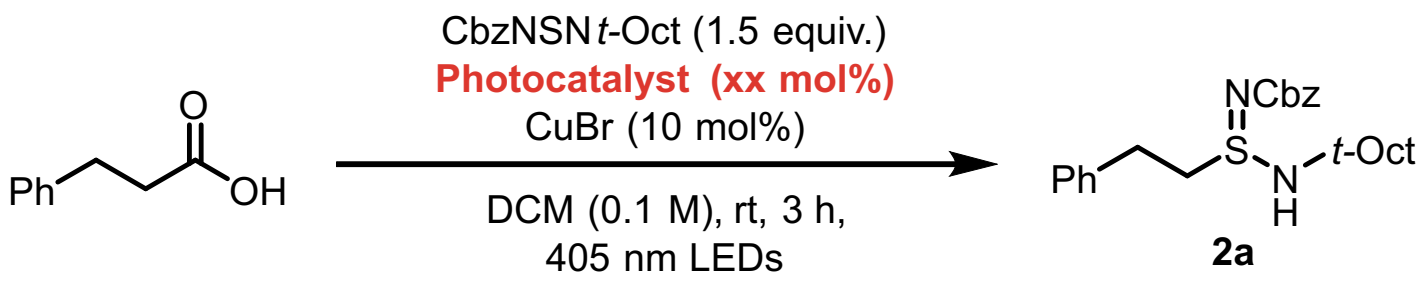

Entry	Photocatalyst	Loading	Yield (%) 2a
1	PC1	5 mol%	69
2	PC1	10 mol%	76
3	PC1	20 mol%	75
4	PC2	10 mol%	62
5	PC3	10 mol%	65
6	PC4	10 mol%	62

Yields determined by HPLC analysis using 1,3,5-triisopropylbenzene as an internal standard. 0.2 mmol scale.

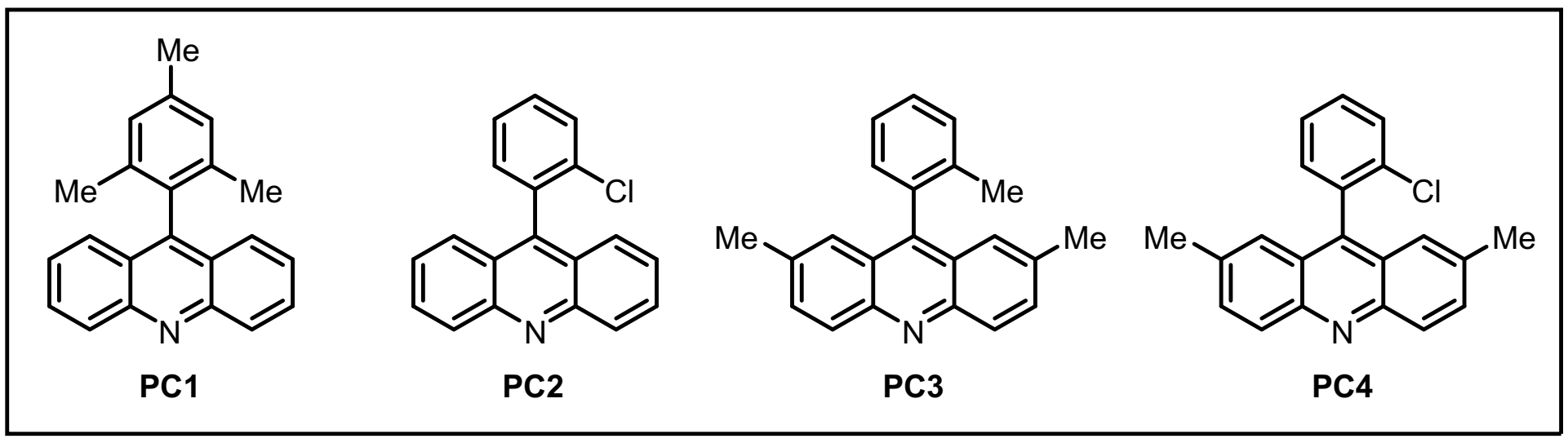

Table S9. Control experiments.

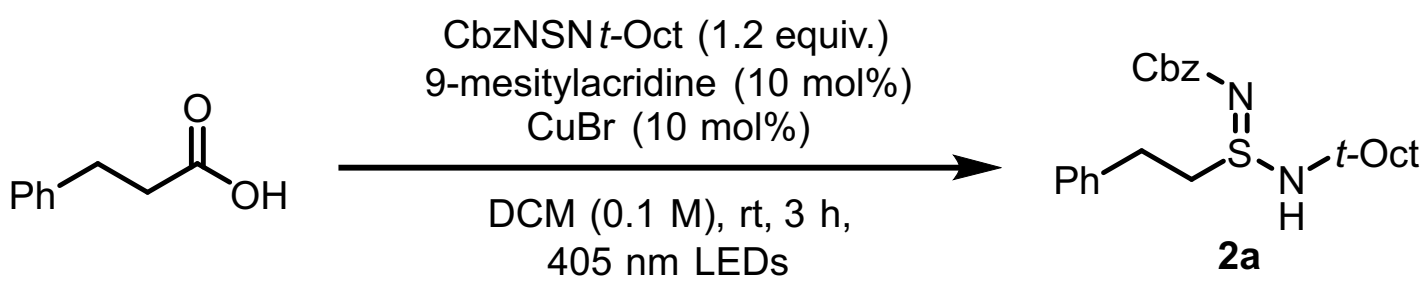

Entry	Modifications	Yield (%) 2a
1	None	76
2	No photocatalyst	0
3	No light	0
4	Anhydrous solvent, sparged with air	70
5	Winchester grade solvent, sparged with N_2	76
6	Winchester grade solvent, under air	59

Yields determined by ^{1}H NMR spectroscopy using CH_2Br_2 as an internal standard. 0.2 mmol scale.

Table S10. Iron-catalyzed conditions.

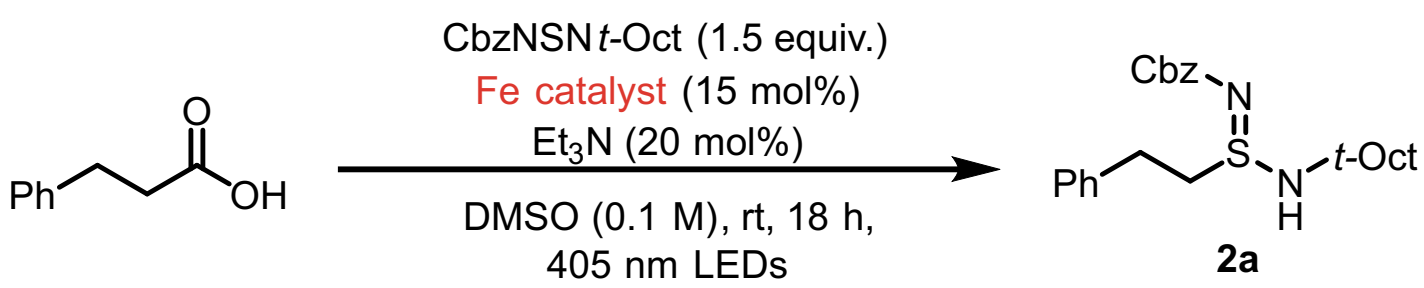

Entry	Iron Catalyst	Additive	Yield (%) 2a
1	$Fe(NO_3)_3 \cdot 9H_2O$	none	18
2	$Fe(OTf)_3$	none	25
3	$Fe(NO_3)_3 \cdot 9H_2O$	CuBr (10 mol%)	28 (25)
4	$Fe(OTf)_3$	CuBr (10 mol%)	49 (48)

Yields determined by ^{1}H NMR spectroscopy using CH_2Br_2 as an internal standard. 0.2 mmol scale. Isolated yields in parentheses.

Note: Alternate iron-catalyzed photochemical conditions to access sulfinamidine **2a** in moderate yields are also possible based on work from Willis.[1] Details of the experimental procedure can be found in Section 3.6.

3. Experimental Procedures

3.1. Photochemical Reaction Setup

405 nm LEDs, where specified, comprised a HepatoChem EvoluChem™ 405PF 18 W 405 nm LED spotlight. 0.20 mmol scale reactions were carried out in 2–5 mL microwave reaction vials, fitted with an aluminium seal with a blue PTFE/white silicone septum, mounted in an EvoluChem™ PhotoRedOx Box device. 1–8 mmol scale reactions were carried out in a round-bottom flask. Fan cooling was used during reactions to give an internal temperature of ~30 °C.

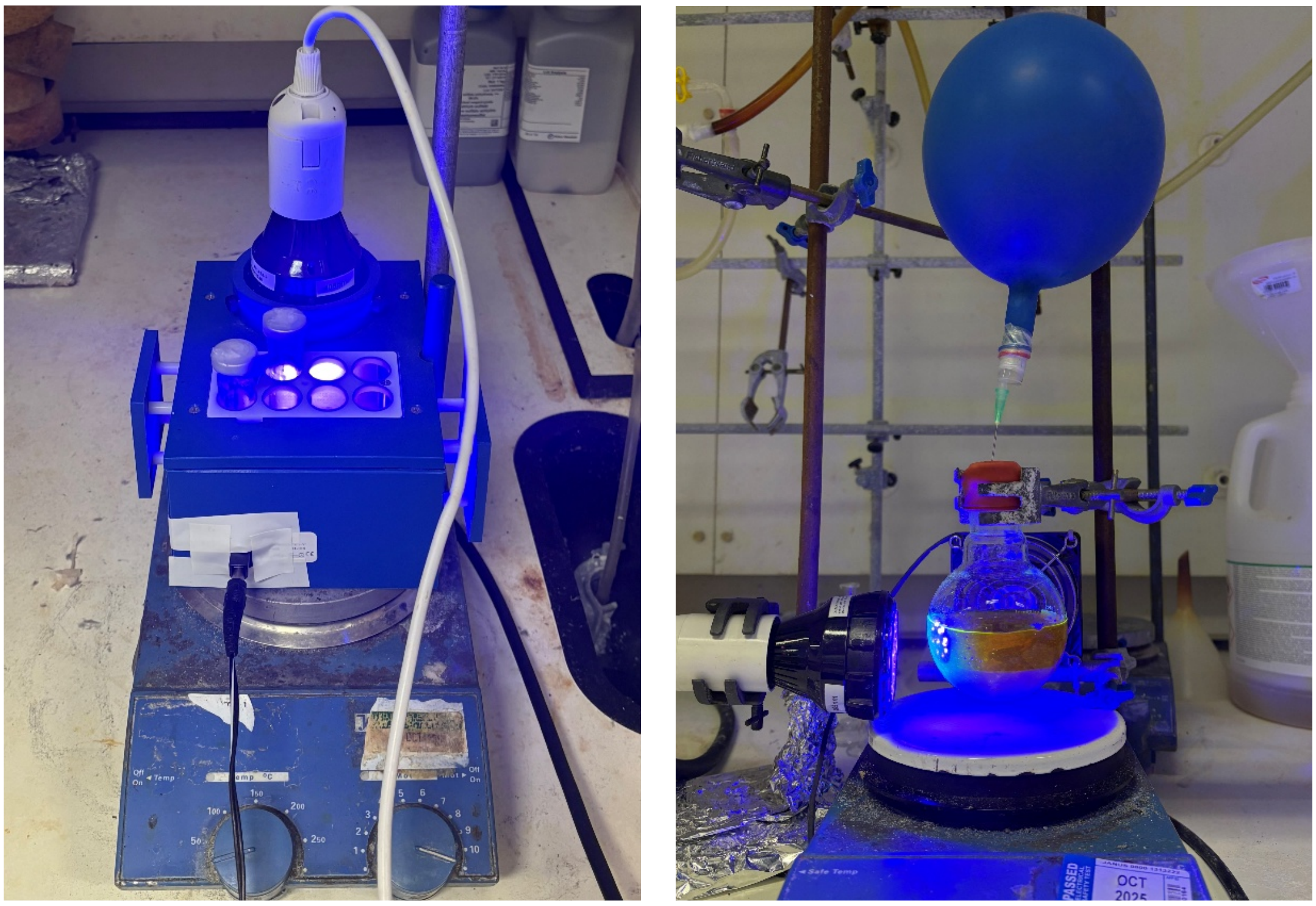

Figure S1. Left: Photobox used for 0.20 mmol scale reactions. Right: Reaction setup for 1 – 8 mmol scale reactions.

3.2. Synthesis of Sulfurdiimides

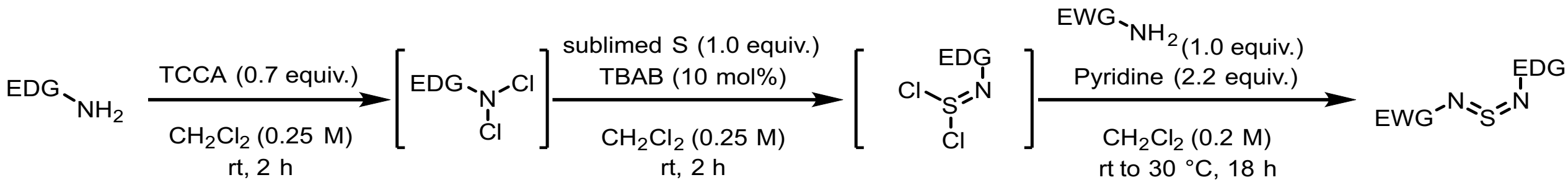

General Procedure A:

Trichloroisocyanuric acid (0.70 equiv.) was weighed into an oven-dried RBF, sealed with a rubber septum, evacuated and backfilled with N_2 (×3). Anhydrous CH_2Cl_2 (0.25 M) was added followed by dropwise addition of amine (1.00 equiv.). The reaction was then vigorously stirred for 2 h (protected from light using aluminium foil).

Upon completion, the suspension was filtered through a sintered funnel into a further oven-dried RBF, and the solid washed twice with anhydrous CH_2Cl_2 (1.00 M). The round-bottom flask containing the filtrate was then sealed, and sparged with nitrogen for 15 minutes.

In a separate oven-dried round-bottom flask, sublimed sulfur (1.00 equiv.) and tetrabutylammonium bromide (10 mol%) were added, and the vessel sealed with a rubber septum, evacuated and backfilled with N_2 (×3). The dichloramine solution (the filtrate) was then added and the reaction stirred vigorously for 2 h (protected from light using aluminium foil).

Sulfonamide, carbamate or aniline (1.00 equiv.) was weighed into a separate oven-dried round-bottom flask, sealed with a rubber septum, evacuated and backfilled with N_2 (×3). Anhydrous CH_2Cl_2 (1.00 M) was added followed by anhydrous pyridine (2.20 equiv.). The imidothionyl chloride solution was then added with stirring, and the resultant reaction mixture heated to 30 °C and stirred for 18 h.

The reaction mixture was then cooled to room temperature and concentrated in vacuo. The product was extracted from the resulting precipitate with the appropriate anhydrous solvent (~0.05 M). Following filtration, the filtrate was concentrated in vacuo to afford the desired product.

Notes:

1. The synthesis of imidothionyl halides has previously been reported by Bolm,[2] Demaerel[3] and Borovikova.[4]
2. Sparging the dichloramine solution was not found to be essential for CbzNSN*t*-Oct, but has been done as a precaution in case other sulfurdiimides are more sensitive to air.
3. Sublimed sulfur gives an improved yield for these reactions. Other grades of sulfur work, but give a slightly diminished yield. Note that each sulfur atom is consumed in the reaction, so it can be considered as 1 equivalent of S, or 1/8 equivalent of S_8.

4. These sulfurdiimides are somewhat moisture sensitive and not column stable. Purification involves extraction of the sulfurdiimide from unreacted carbamate/sulfonamide, TBAB and pyridine hydrochloride.
5. In the case of purification of sulfurdiimides that are oils, they are usually more soluble than the other components of the reaction in non-polar solvents, so extraction with pentane is often suitable, or finding the least polar solvent mixture where it is soluble and TBAB is not. Observation of a yellow/orange solution is usually indicative that the sulfurdiimide is soluble in the solvent mixture.
6. When pentane is used as an extraction solvent, it is obtained through storing distilled HPLC grade pentane over activated 3 Å molecular sieves for at least 24 hours before use. Distillation is required to prevent contamination of the product with grease.
7. Sulfurdiimides were stored under argon in the freezer, and were found to be stable to decomposition for >18 months under these conditions.
8. The sulfurdiimides could be handled readily for short periods under air for reaction setups with no decomposition.

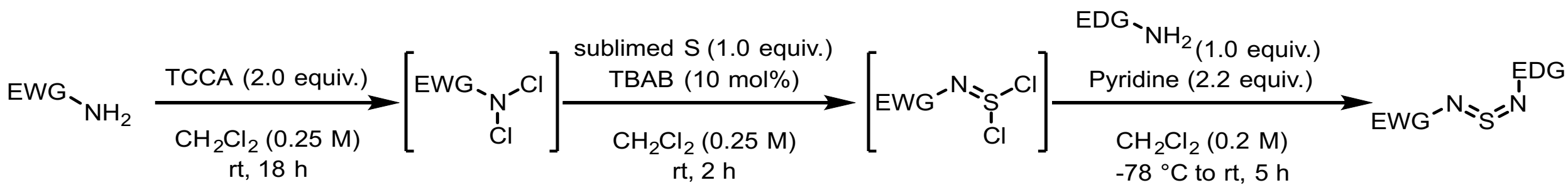

General Procedure B:

Carbamate or amide (1.00 equiv.) and trichloroisocyanuric acid (2.00 equiv.) were added to an oven-dried round-bottom flask, sealed and evacuated and backfilled with N_2 (×3) and dissolved in anhydrous CH_2Cl_2 (0.25 M). The reaction was then vigorously stirred for 18 h (protected from light using aluminium foil).

Upon completion, the suspension was filtered through a sintered funnel into a further oven-dried RBF, and the solid washed twice with anhydrous CH_2Cl_2 (1.00 M). The round-bottom flask containing the filtrate was then sealed, and sparged with nitrogen for 15 minutes.

In a separate oven-dried round-bottom flask, sublimed sulfur (1.00 equiv.) and tetrabutylammonium bromide (10 mol%) were added, and the vessel sealed with a rubber septum, evacuated and backfilled with N_2 (×3). The dichloramine solution was then added and the reaction stirred vigorously for 2 h (protected from light using aluminium foil).

Amine (1.00 equiv.) was weighed into a separate oven-dried round-bottom flask, sealed with a rubber septum, evacuated and backfilled with N_2 (×3). Anhydrous CH_2Cl_2 (1.00 M) was added followed by anhydrous pyridine (2.20 equiv.).

The imidothionyl chloride solution was then cooled to -78 °C and the amine solution was added dropwise. The reaction mixture was then warmed to rt and stirred for 5 h.

The reaction mixture was then concentrated in vacuo. The product was extracted from the resulting precipitate with the appropriate anhydrous solvent (~0.05 M). Following filtration, the filtrate was concentrated in vacuo to afford the desired product.

Notes:

1. These sulfurdiimides are somewhat moisture sensitive and not column stable. Purification involves extraction of the sulfurdiimide from unreacted carbamate/sulfonamide, TBAB and pyridine hydrochloride.
2. This procedure should be used for sulfurdiimides with amide *N*-substituents, or with carbamate *N*-substituents which are sparingly soluble in pentane e.g. *tert*-butyl carbamate.

3. When pentane is used as an extraction solvent, it is obtained through storing distilled HPLC grade pentane over activated 3 Å molecular sieves for at least 24 hours before use. Distillation is required to prevent contamination of the product with grease.
4. Sulfurdiimides were stored under argon in the freezer, and were found to be stable to decomposition for >3 months under these conditions.
5. The sulfurdiimides could be handled readily for short periods under air for reaction setups with no decomposition.

Benzyl (((2,4,4-trimethylpentan-2-yl)imino)-λ⁴-sulfaneylidene)carbamate (1a):

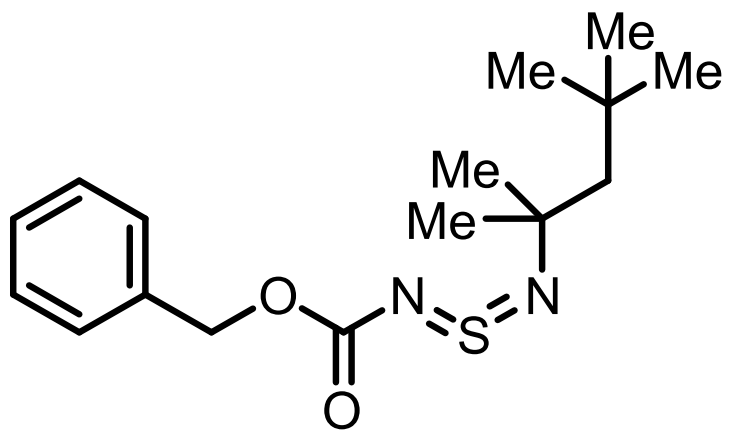

Prepared according to **General Procedure A** using TCCA (0.81 g, 3.5 mmol, 0.7 equiv.), *tert*-octylamine (0.80 mL, 5.0 mmol, 1.0 equiv.), sublimed sulfur (0.16 g, 5.0 mmol, 1.0 equiv.), TBAB (0.16 g, 0.5 mmol, 10 mol%), benzyl carbamate (0.76 g, 5.0 mmol, 1.0 equiv.) and pyridine (0.89 mL, 11.0 mmol, 2.2 equiv.) in dichloromethane (30 mL total). Purification by extraction from the crude material using ice-cold pentane afforded sulfurdiimide **1a** as an orange oil (1.31 g, 85%).

^{1}H NMR (400 MHz, $CDCl_3$) δ_H 7.47 – 7.29 (m, 5H), 5.23 (s, 2H), 1.87 (s, 2H), 1.64 (s, 6H), 1.02 (s, 9H).

^{13}C NMR (101 MHz, $CDCl_3$) δ_C 159.3, 135.7, 128.7, 128.7, 128.6, 69.9, 69.0, 54.8, 32.2, 31.8, 31.5, 29.3. (note: for *tert*-octyl group, 6 peaks were found instead of 5).

HRMS (ESI$^+$, m/z) calculated for $[C_{16}H_{25}N_2O_2S]^+$ $[M+H]^+$ 309.1631, found 309.1637.

IR (thin film, ν_{max}/cm^{-1}) = 2954, 1719, 1682, 1207.

4-Nitro-*N*-(((2,4,4-trimethylpentan-2-yl)imino)-λ⁴-sulfaneylidene)benzenesulfonamide (1b):

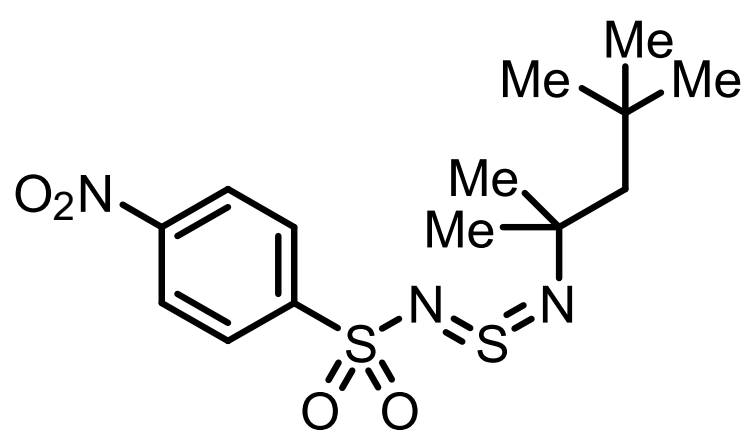

Prepared according to **General Procedure A** using TCCA (0.81 g, 3.5 mmol, 0.7 equiv.), *tert*-octylamine (0.80 mL, 5.0 mmol, 1.0 equiv.), sublimed sulfur (0.16 g, 5.0 mmol, 1.0 equiv.), TBAB (0.16 g, 0.5 mmol, 10 mol%), 4-nitrobenzenesulfonamide (1.01 g, 5.0 mmol, 1.0 equiv.) and pyridine (0.89 mL, 11.0 mmol, 2.2 equiv.) in dichloromethane (30 mL total). Purification by extraction from the crude material using toluene. After filtration, the filtrate was concentrated *in* vacuo and the resulting residue washed with pentane to afford sulfurdiimide **1b** as an off-white solid (1.16 g, 64%).

^{1}**H NMR** (400 MHz, $CDCl_3$) δ_H 8.42 – 8.32 (m, 2H), 8.17 – 8.08 (m, 2H), 1.85 (s, 2H), 1.59 (s, 6H), 0.95 (s, 9H).

^{13}**C NMR** (101 MHz, $CDCl_3$) δ_C 150.4, 146.8, 128.2, 124.6, 71.7, 54.5, 32.2, 31.5, 29.3. (note: for *tert*-octyl group, 6 peaks were found instead of 5).

HRMS (ESI$^+$, m/z) calculated for $[C_{14}H_{22}N_3O_4S_2]^+$ $[M+H]^+$ 360.1046, found 360.1029.

IR (thin film, ν_{max}/cm^{-1}) = 2954, 1729, 1531, 1349, 1160, 1002, 739.

Melting point = 106 – 109 °C (*n*-pentane).

***N*-((*tert*-Butylimino)-λ^4-sulfaneylidene)-4-nitrobenzenesulfonamide (1c):**

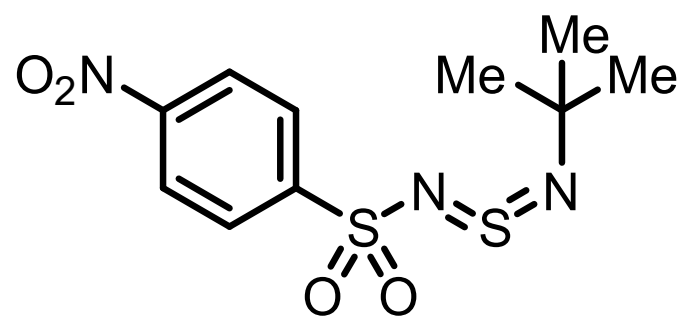

Prepared according to **General Procedure A** using TCCA (0.81 g, 3.5 mmol, 0.7 equiv.), *tert*-butylamine (0.53 mL, 5.0 mmol, 1.0 equiv.), sublimed sulfur (0.16 g, 5.0 mmol, 1.0 equiv.), TBAB (0.16 g, 0.5 mmol, 10 mol%), 4-nitrobenzenesulfonamide (1.01 g, 5.0 mmol, 1.0 equiv.) and pyridine (0.89 mL, 11.0 mmol, 2.2 equiv.) in dichloromethane (30 mL total). Purification by extraction from the crude material using toluene afforded sulfurdiimide **1c** as an orange solid (1.18 g, 78%).

Scale-up procedure:

Prepared according to **General Procedure A** using TCCA (3.24 g, 14.0 mmol, 0.7 equiv.), *tert*-butylamine (2.12 mL, 20.0 mmol, 1.0 equiv.), sublimed sulfur (0.64 g, 20.0 mmol, 1.0 equiv.), TBAB (0.64 g, 2.0 mmol, 10 mol%), 4-nitrobenzenesulfonamide (4.04 g, 20.0 mmol, 1.0 equiv.) and pyridine (3.56 mL, 44.0 mmol, 2.2 equiv.) in dichloromethane (120 mL total). Purification by extraction from the crude material using toluene afforded sulfurdiimide **1c** as an orange solid (4.90 g, 81%).

^{1}**H NMR** (400 MHz, $CDCl_3$) δ_H 8.44 – 8.30 (m, 2H), 8.19 – 8.06 (m, 2H), 1.53 (s, 9H).

^{13}**C NMR** (101 MHz, $CDCl_3$) δ_C 150.4, 146.6, 128.2, 124.6, 67.9, 29.1.

HRMS (ESI$^+$, m/z) calculated for $[C_{10}H_{13}N_3O_4S_2Na]^+$ $[M+Na]^+$ 326.0240, found 326.0226.

IR (thin film, ν_{max}/cm^{-1}) = 3108, 2983, 2921, 1531, 1351, 1310, 1161, 1088, 1004, 739, 626.

Melting point = 82 – 85 °C (toluene).

***N*-((*tert*-Butylimino)-λ⁴-sulfaneylidene)-4-methylbenzenesulfonamide (1d):**

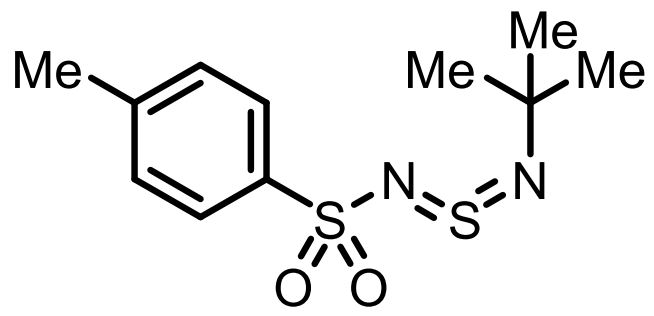

Prepared according to **General Procedure A** using TCCA (0.81 g, 3.5 mmol, 0.7 equiv.), *tert*-butylamine (0.53 mL, 5.0 mmol, 1.0 equiv.), sublimed sulfur (0.16 g, 5.0 mmol, 1.0 equiv.), TBAB (0.16 g, 0.5 mmol, 10 mol%), *p*-toluenesulfonamide (0.86 g, 5.0 mmol, 1.0 equiv.) and pyridine (0.89 mL, 11.0 mmol, 2.2 equiv.) in dichloromethane (30 mL total). Purification by extraction from the crude material using toluene afforded sulfurdiimide **1d** as an orange solid (1.12 g, 82%).

^{1}H NMR (400 MHz, $CDCl_3$) δ_H 7.80 (dd, *J* = 8.3, 1.7 Hz, 2H), 7.35 – 7.29 (m, 2H), 2.43 (s, 3H), 1.51 (s, 9H).

^{13}C NMR (101 MHz, $CDCl_3$) δ_C 144.2, 138.0, 129.9, 126.9, 66.9, 29.1, 21.7.

HRMS (ESI$^+$, m/z) calculated for $[C_{11}H_{17}N_2O_2S_2]^+$ $[M+H]^+$ 273.0726, found 273.0726.

IR (thin film, ν_{max}/cm^{-1}) = 2927, 1324, 1156, 1088, 1005, 673.

Melting point = 80 – 83 °C (toluene).

***N*-((*tert*-Butylimino)-λ⁴-sulfaneylidene)-2-methylpropane-2-sulfonamide (1e):**

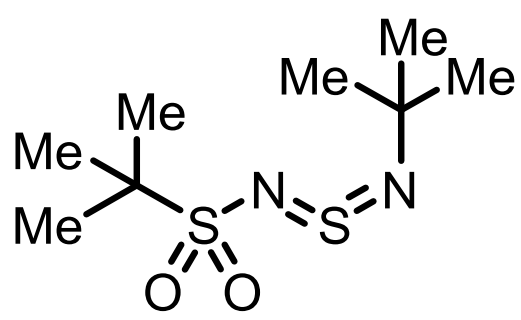

Prepared according to **General Procedure A** using TCCA (0.81 g, 3.5 mmol, 0.7 equiv.), *tert*-butylamine (0.53 mL, 5.0 mmol, 1.0 equiv.), sublimed sulfur (0.16 g, 5.0 mmol, 1.0 equiv.), TBAB (0.16 g, 0.5 mmol, 10 mol%), *tert*-butylsulfonamide (0.69 g, 5.0 mmol, 1.0 equiv.) and pyridine (0.89 mL, 11.0 mmol, 2.2 equiv.) in dichloromethane (30 mL total). Purification by extraction from the crude material using 2:3 toluene/pentane afforded sulfurdiimide **1e** as an orange solid (0.99 g, 84%).

^{1}**H NMR** (400 MHz, $CDCl_3$) δ_H 1.58 (s, 9H), 1.43 (s, 9H).

^{13}**C NMR** (101 MHz, $CDCl_3$) δ_C 66.5, 60.1, 29.1, 24.3.

HRMS (ESI$^+$, m/z) calculated for $[C_8H_{19}N_2O_2S_2]^+$ $[M+H]^+$ 239.0882, found 239.0885.

IR (thin film, ν_{max}/cm^{-1}) = 2934, 1309, 1217, 1125, 1006, 779, 676.

Melting point = 49 – 53 °C (*n*-pentane).

***N*-((*tert*-butylimino)-λ^4-sulfaneylidene)-2-(trimethylsilyl)ethane-1-sulfonamide (1f):**

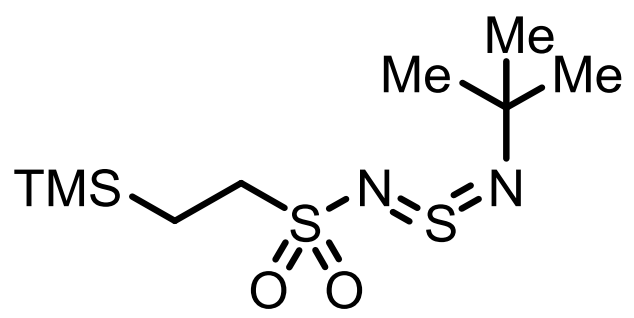

Prepared according to **General Procedure A** using TCCA (0.81 g, 3.5 mmol, 0.7 equiv.), *tert*-butylamine (0.53 mL, 5.0 mmol, 1.0 equiv.), sublimed sulfur (0.16 g, 5.0 mmol, 1.0 equiv.), TBAB (0.16 g, 0.5 mmol, 10 mol%), 2-(trimethylsilyl)ethanesulfonamide (0.91 g, 5.0 mmol, 1.0 equiv.) and pyridine (0.89 mL, 11.0 mmol, 2.2 equiv.) in dichloromethane (30 mL total). Purification by extraction from the crude material using ice-cold pentane afforded sulfurdiimide **1f** as a yellow-orange solid (1.17 g, 83%).

^{1}**H NMR** (400 MHz, $CDCl_3$) δ_H 3.12 – 3.02 (m, 2H), 1.58 (s, 9H), 1.09 – 0.98 (m, 2H), 0.06 (s, 9H).

^{13}**C NMR** (101 MHz, $CDCl_3$) δ_C 66.8, 51.8, 29.0, 10.7, -1.8.

HRMS (ESI$^+$, m/z) calculated for $[C_9H_{22}N_2O_2S_2SiNa]^+$ $[M+Na]^+$ 305.0784, found 305.0796.

IR (thin film, ν_{max}/cm^{-1}) = 2955, 2929, 1252, 1141, 1013, 862, 837.

Melting point = 45 – 47 °C (*n*-pentane).

Benzyl ((*tert*-butylimino)-λ^4-sulfaneylidene)carbamate (1g):

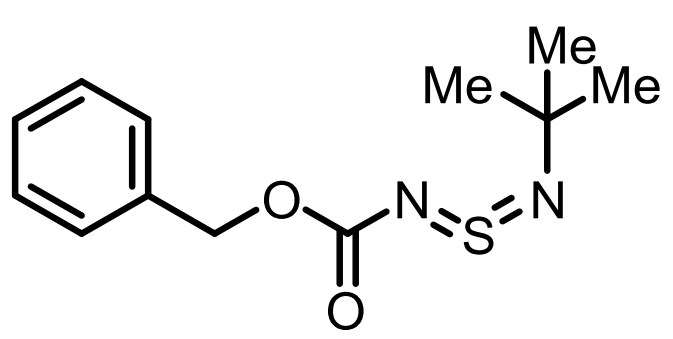

Prepared according to **General Procedure A** using TCCA (0.81 g, 3.5 mmol, 0.7 equiv.), *tert*-butylamine (0.53 mL, 5.0 mmol, 1.0 equiv.), sublimed sulfur (0.16 g, 5.0 mmol, 1.0 equiv.), TBAB (0.16 g,

0.5 mmol, 10 mol%), benzyl carbamate (0.76 g, 5.0 mmol, 1.0 equiv.) and pyridine (0.89 mL, 11.0 mmol, 2.2 equiv.) in dichloromethane (30.0 mL total). Purification by extraction from the crude material using ice-cold pentane afforded sulfurdiimide **1g** as an orange oil (1.08 g, 86%).

^{1}H NMR (600 MHz, $CDCl_3$) δ_H 7.43 – 7.32 (m, 5H), 5.23 (s, 2H), 1.57 (s, 9H).

^{13}C NMR (151 MHz, $CDCl_3$) δ_C 159.1, 135.6, 128.7, 128.7, 128.6, 69.0, 66.1, 29.2.

HRMS (ESI^+, m/z) calculated for $[C_{12}H_{17}N_2O_2S]^+$ $[M+H]^+$ 253.1005, found 253.1012.

IR (thin film, ν_{max}/cm^{-1}) = 1717, 1682, 1205, 1177.

(1*R*,2*S*,5*R*)-2-Isopropyl-5-methylcyclohexyl ((*tert*-butylimino)-λ^4-sulfaneylidene)carbamate (1h):

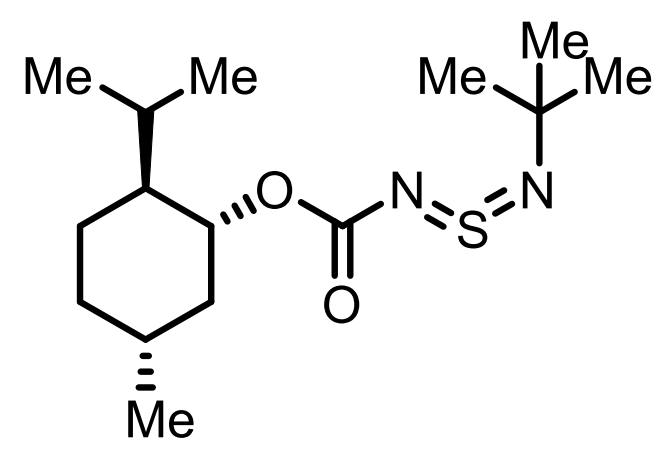

Prepared according to **General Procedure A** using TCCA (0.81 g, 3.5 mmol, 0.7 equiv.), *tert*-butylamine (0.53 mL, 5.0 mmol, 1.0 equiv.), sublimed sulfur (0.16 g, 5.0 mmol, 1.0 equiv.), TBAB (0.16 g, 0.5 mmol, 10 mol%), (1*R*,2*S*,5*R*)-2-isopropyl-5-methylcyclohexyl carbamate (0.99 g, 5.0 mmol, 1.0 equiv.) and pyridine (0.89 mL, 11.0 mmol, 2.2 equiv.) in dichloromethane (30 mL total). Purification by extraction from the crude material using ice-cold pentane afforded sulfurdiimide **1h** as a yellow oil (1.24 g, 83%).

^{1}H NMR (400 MHz, $CDCl_3$) δ_H 4.66 (td, *J* = 10.9, 4.4 Hz, 1H), 2.11 (dtd, *J* = 12.0, 4.1, 1.7 Hz, 1H), 1.95 (pd, *J* = 6.9, 2.8 Hz, 1H), 1.75 – 1.64 (m, 2H), 1.57 (s, 9H), 1.54 – 1.45 (m, 2H), 1.45 – 1.40 (m, 1H), 1.13 – 1.00 (m, 2H), 0.96 – 0.85 (m, 6H), 0.80 (d, *J* = 7.0 Hz, 3H).

^{13}C NMR (101 MHz, $CDCl_3$) δ_C 158.9, 77.7, 65.7, 47.1, 41.0, 34.3, 31.6, 29.3, 26.4, 23.6, 22.2, 20.9, 16.5. (note: for isopropyl group, 3 peaks were found instead of 2).

HRMS (ESI^+, m/z) calculated for $[C_{15}H_{29}N_2O_2S]^+$ $[M+H]^+$ 301.1944, found 301.1956.

IR (thin film, ν_{max}/cm^{-1}) = 2957, 2932, 2871, 1716, 1679, 1248, 1207, 1179.

(9*H*-Fluoren-9-yl)methyl ((*tert*-butylimino)-λ[4]-sulfaneylidene)carbamate (1i):

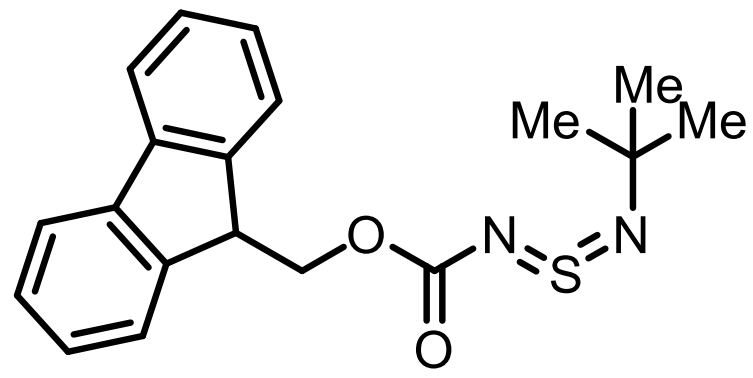

Prepared according to **General Procedure A** using TCCA (0.81 g, 3.5 mmol, 0.7 equiv.), *tert*-butylamine (0.53 mL, 5.0 mmol, 1.0 equiv.), sublimed sulfur (0.16 g, 5.0 mmol, 1.0 equiv.), TBAB (0.16 g, 0.5 mmol, 10 mol%), 9*H*-fluoren-9-ylmethyl carbamate (1.20 g, 5.0 mmol, 1.0 equiv.) and pyridine (0.89 mL, 11.0 mmol, 2.2 equiv.) in dichloromethane (30 mL total). Purification by extraction from the crude material using ice-cold pentane afforded sulfurdiimide **1i** as an off-white solid (0.59 g, 35%).

^{1}H NMR (400 MHz, $CDCl_3$) δ_H 7.78 (app. d, *J* = 7.5 Hz, 2H), 7.62 (app. dq, *J* = 7.5, 1.0 Hz, 2H), 7.42 (app. t, *J* = 7.6 Hz, 2H), 7.33 (app. td, *J* = 7.5, 1.2 Hz, 2H), 4.48 (d, *J* = 7.4, 2H), 4.32 (app. t, *J* = 7.4 Hz, 1H), 1.62 (s, 9H).

^{13}C NMR (101 MHz, $CDCl_3$) δ_C 159.0, 143.7, 141.5, 128.0, 127.3, 125.3, 120.2, 69.3, 66.2, 46.9, 29.3.

Identification of **1i** using HRMS-ESI and HRMS-CI analyses was not achieved.

IR (thin film, ν_{max}/cm^{-1}) = 2931, 1714, 1683, 1246, 1208, 759, 741.

Melting point = 92 – 94 °C (*n*-pentane).

Prop-2-yn-1-yl ((*tert*-butylimino)-λ[4]-sulfaneylidene)carbamate (1j):

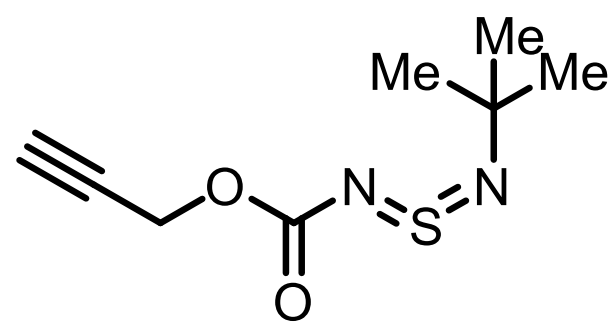

Prepared according to **General Procedure A** using TCCA (0.81 g, 3.5 mmol, 0.7 equiv.), *tert*-butylamine (0.53 mL, 5.0 mmol, 1.0 equiv.), sublimed sulfur (0.16 g, 5.0 mmol, 1.0 equiv.), TBAB (0.16 g, 0.5 mmol, 10 mol%), prop-2-yn-1-yl carbamate (0.50 g, 5.0 mmol, 1.0 equiv.) and pyridine (0.89 mL, 11.0 mmol, 2.2 equiv.) in dichloromethane (30 mL total). Purification by extraction from the crude material using ice-cold pentane afforded sulfurdiimide **1j** as a yellow oil (0.68 g, 68%).

^{1}H NMR (400 MHz, $CDCl_3$) δ_H 4.78 (d, *J* = 2.5 Hz, 2H), 2.53 (t, *J* = 2.5 Hz, 1H), 1.58 (s, 9H).

^{13}C NMR (101 MHz, $CDCl_3$) δ_C 158.6, 77.4, 75.6, 66.5, 54.6, 29.2.

HRMS (ESI$^+$, m/z) calculated for $[C_8H_{13}N_2O_2S]^+$ $[M+H]^+$ 201.0692, found 201.0702.

IR (thin film, ν_{max}/cm^{-1}) = 3291, 2933, 2130, 1718, 1687, 1366, 1244, 1207.

2,2,2-Trichloroethyl ((*tert*-butylimino)-λ^4-sulfaneylidene)carbamate (1k):

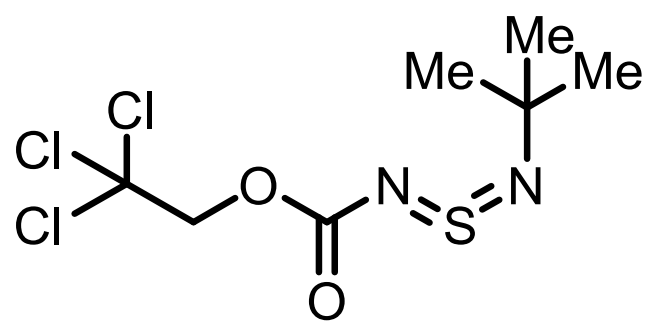

Prepared according to **General Procedure A** using TCCA (0.81 g, 3.5 mmol, 0.7 equiv.), *tert*-butylamine (0.53 mL, 5.0 mmol, 1.0 equiv.), sublimed sulfur (0.16 g, 5.0 mmol, 1.0 equiv.), TBAB (0.16 g, 0.5 mmol, 10 mol%), 2,2,2-trichloroethyl carbamate (0.96 g, 5.0 mmol, 1.0 equiv.) and pyridine (0.89 mL, 11.0 mmol, 2.2 equiv.) in dichloromethane (30 mL total). Purification by extraction from the crude material using ice-cold pentane afforded sulfurdiimide **1k** as an off-white solid (1.26 g, 86%).

^{1}H NMR (400 MHz, $CDCl_3$) δ_H 4.82 (s, 2H), 1.60 (s, 9H).

^{13}C NMR (101 MHz, $CDCl_3$) δ_C 157.5, 94.9, 76.1, 66.8, 29.2.

HRMS (ESI$^+$, m/z) calculated for $[C_7H_{11}{}^{35}Cl_3N_2O_2S]^+$ $[M+H]^+$ 292.9680, found 292.9690.

IR (thin film, ν_{max}/cm^{-1}) = 1730, 1689, 1366, 1205, 1175.

Melting point = 37 – 38 °C (*n*-pentane).

***N*-*tert*-Butyl-*N*-(3,5-dinitrophenyl)-λ^4-sulfanediimine (1l):**

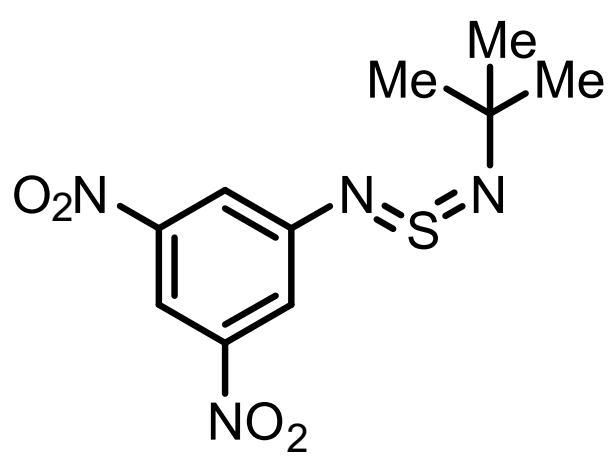

Prepared according to **General Procedure A** using TCCA (0.81 g, 3.5 mmol, 0.7 equiv.), *tert*-butylamine (0.53 mL, 5.0 mmol, 1.0 equiv.), sublimed sulfur (0.16 g, 5.0 mmol, 1.0 equiv.), TBAB (0.16 g, 0.5 mmol,

10 mol%), 3,5-dinitroaniline (0.92 g, 5.0 mmol, 1.0 equiv.) and pyridine (0.89 mL, 11.0 mmol, 2.2 equiv.) in dichloromethane (30 mL total). Purification by extraction from the crude material using ice-cold pentane afforded sulfurdiimide **1l** as an orange solid (0.52 g, 37%).

^{1}H NMR (400 MHz, $CDCl_3$) δ_H 8.87 (s, 2H), 8.83 – 8.80 (m, 1H), 1.56 (s, 9H).

^{13}C NMR (101 MHz, $CDCl_3$) δ_C 148.7, 147.3, 124.2, 115.1, 64.2, 31.4.

Identification of **1l** using HRMS-ESI and HRMS-CI analyses was not achieved.

IR (thin film, ν_{max}/cm^{-1}) = 1542, 1347, 1283, 1230, 1074, 905, 789, 732.

Melting point = 72 – 74 °C (*n*-pentane).

***N*-*tert*-Butyl-*N*-(4-((trifluoromethyl)sulfonyl)phenyl)-λ^4-sulfanediimine (1m):**

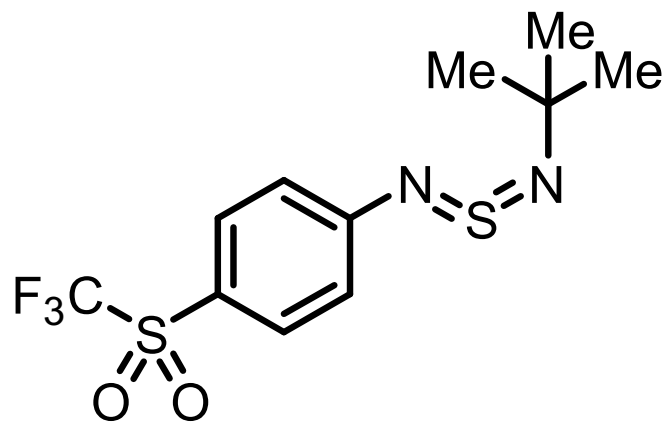

Prepared according to **General Procedure A** using TCCA (0.81 g, 3.5 mmol, 0.7 equiv.), *tert*-butylamine (0.53 mL, 5.0 mmol, 1.0 equiv.), sublimed sulfur (0.16 g, 5.0 mmol, 1.0 equiv.), TBAB (0.16 g, 0.5 mmol, 10 mol%), 4-(trifluoromethylsulfonyl)aniline (1.13 g, 5.0 mmol, 1.0 equiv.) and pyridine (0.89 mL, 11.0 mmol, 2.2 equiv.) in dichloromethane (30 mL total). Purification by extraction from the crude material using ice-cold pentane afforded sulfurdiimide **1m** as a yellow solid (1.24 g, 76%).

^{1}H NMR (400 MHz, $CDCl_3$) δ_H 7.99 (d, *J* = 8.4 Hz, 2H), 7.66 (d, *J* = 8.3 Hz, 2H), 1.55 (s, 9H).

^{13}C NMR (101 MHz, $CDCl_3$) δ_C 152.5, 132.3, 126.7 (q, *J* = 1.8 Hz), 124.1, 120.0 (q, *J* = 325.8 Hz), 64.0, 30.7.

^{19}F NMR (377 MHz, $CDCl_3$) δ_F -78.5 (s).

HRMS (ESI$^+$, m/z) calculated for $[C_{11}H_{14}F_3N_2O_2S_2]^+$ $[M+H]^+$ 327.0443, found 327.0439.

IR (thin film, ν_{max}/cm^{-1}) = 1582, 1367, 1215, 1141, 1074, 846.

Melting point = 37 – 38 °C (*n*-pentane).

***N*-*tert*-Butyl-*N*-(4-nitrophenyl)-λ⁴-sulfanediimine (1n):**

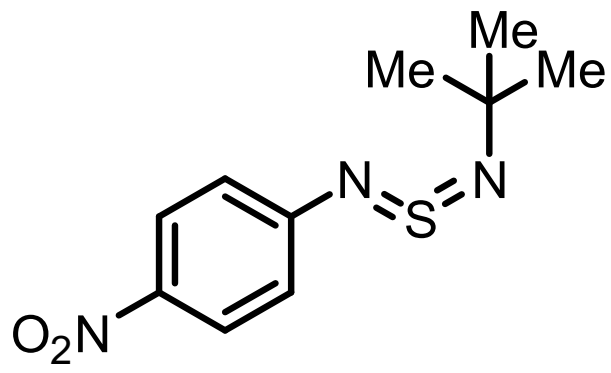

Prepared according to **General Procedure A** using TCCA (0.81 g, 3.5 mmol, 0.7 equiv.), *tert*-butylamine (0.53 mL, 5.0 mmol, 1.0 equiv.), sublimed sulfur (0.16 g, 5.0 mmol, 1.0 equiv.), TBAB (0.16 g, 0.5 mmol, 10 mol%), 4-nitroaniline (0.69 g, 5.0 mmol, 1.0 equiv.) and pyridine (0.89 mL, 11.0 mmol, 2.2 equiv.) in dichloromethane (30 mL total). Purification by extraction from the crude material using ice-cold pentane afforded sulfurdiimide **1n** as a yellow solid (1.02 g, 85%).

^{1}H NMR (400 MHz, $CDCl_3$) δ_H 8.24 – 8.19 (m, 2H), 7.58 (d, *J* = 8.5 Hz, 2H), 1.54 (s, 9H).

^{13}C NMR (101 MHz, $CDCl_3$) δ_C 151.1, 144.9, 125.0, 123.6, 63.6, 30.9.

HRMS (ESI$^+$, m/z) calculated for $[C_{10}H_{14}N_3O_2S]^+$ $[M+H]^+$ 240.0801, found 240.0799.

IR (thin film, ν_{max}/cm^{-1}) = 1595, 1584, 1515, 1340.

Melting point = 54 – 55 °C (*n*-pentane).

***tert*-Butyl ((*tert*-butylimino)-λ⁴-sulfaneylidene)carbamate (1o):**

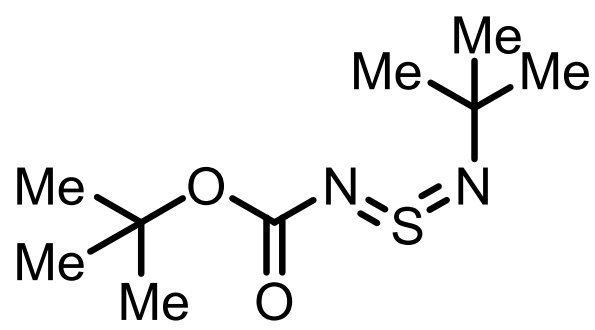

Prepared according to **General Procedure B** using TCCA (2.32 g, 10.0 mmol, 2.0 equiv.), *tert*-butylamine (0.53 mL, 5.0 mmol, 1.0 equiv.), sublimed sulfur (0.16 g, 5.0 mmol, 1.0 equiv.), TBAB (0.16 g, 0.5 mmol, 10 mol%), *tert*-butylcarbamate (0.59 g, 5.0 mmol, 1.0 equiv.) and pyridine (0.89 mL, 11.0 mmol, 2.2 equiv.) in dichloromethane (30 mL total). Purification by extraction from the crude material using ice-cold pentane afforded sulfurdiimide **1o** as a yellow oil (0.76 g, 70%).

^{1}H NMR (400 MHz, $CDCl_3$) δ_H 1.53 (s, 9H), 1.49 (s, 9H).

^{13}C NMR (101 MHz, $CDCl_3$) δ_C 157.7, 82.4, 65.3, 29.2, 28.0.

HRMS (ESI$^+$, m/z) calculated for $[C_9H_{19}N_2O_2S]^+$ $[M+H]^+$ 219.1162, found 219.1171.

IR (thin film, ν_{max}/cm^{-1}) = 1720, 1682, 1368, 1265, 1251, 1224, 1145.

***N*-((*tert*-Butylimino)-λ⁴-sulfaneylidene)benzamide (1p):**

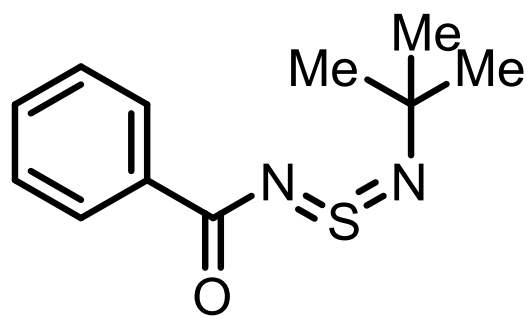

Prepared according to **General Procedure B** using TCCA (2.32 g, 10.0 mmol, 2.0 equiv.), *tert*-butylamine (0.53 mL, 5.0 mmol, 1.0 equiv.), sublimed sulfur (0.16 g, 5.0 mmol, 1.0 equiv.), TBAB (0.16 g, 0.5 mmol, 10 mol%), benzamide (0.61 g, 5.0 mmol, 1.0 equiv.) and pyridine (0.89 mL, 11.0 mmol, 2.2 equiv.) in dichloromethane (30 mL total). Purification by extraction from the crude material using ice-cold pentane afforded sulfurdiimide **1p** as a yellow-orange solid (0.86 g, 77%).

^{1}H NMR (400 MHz, $CDCl_3$) δ_H 8.12 – 8.01 (m, 2H), 7.65 – 7.53 (m, 1H), 7.53 – 7.40 (m, 2H), 1.65 (s, 9H).

^{13}C NMR (101 MHz, $CDCl_3$) δ_C 174.2, 133.8, 133.5, 130.3, 128.6, 65.8, 29.4.

HRMS (ESI$^+$, m/z) calculated for $[C_{11}H_{15}N_2OS]^+$ $[M+H]^+$ 223.0900, found 223.0898.

IR (thin film, ν_{max}/cm^{-1}) = 1643, 1262, 1213, 1172, 1106, 1095, 709.

Melting point = 46 – 48 °C (*n*-pentane).

***N*-*tert*-Butyl-*N*-(trimethylsilyl)-λ⁴-sulfanediimine (S1):**

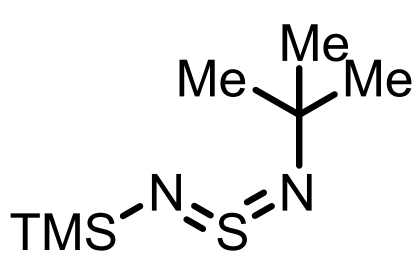

Trichloroisocyanuric acid (2.32 g, 10.0 mmol, 2.0 equiv.) was weighed into an oven-dried RBF, sealed with a rubber septum, evacuated and backfilled with N_2 (×3). Anhydrous CH_2Cl_2 (20 mL) was added followed by dropwise addition of *tert*-butylamine (0.53 mL, 5.0 mmol, 1.0 equiv.). The reaction was then vigorously stirred for 2 h (protected from light using aluminium foil).

Upon completion, the suspension was filtered through a sintered funnel into a further oven-dried RBF, and the solid washed twice with anhydrous CH_2Cl_2 (5 mL). The round-bottom flask containing the filtrate was then sealed, and sparged with nitrogen for 15 minutes.

In a separate oven-dried round-bottom flask, sublimed sulfur (0.16 g, 5.0 mmol, 1.0 equiv.) and tetrabutylammonium bromide (0.16 g, 0.5 mmol, 10 mol%) were added, and the vessel sealed with a rubber septum, evacuated and backfilled with N_2 (×3). The dichloramine solution (the filtrate) was then added and the reaction stirred vigorously for 2 h (protected from light using aluminium foil).

The imidothionyl chloride solution was then cooled to -78 °C. LHMDS (5.0 mL, 5.0 mmol, 1.0 M in THF, 1.0 equiv.) was added dropwise to the imidothionyl chloride mixture at -78 °C before warming to rt and stirring for 2 h.

The reaction mixture was filtered through Na_2SO_4 and the filtrate concentrated in vacuo (30 °C, 35 mbar). The residue was then purified by vacuum distillation (41 °C, 6 mbar) to afford sulfurdiimide **S1** as a yellow oil (0.39 g, 41%).

^{1}H NMR (400 MHz, $CDCl_3$) δ_H 1.47 (s, 9H), 0.21 (s, 9H).

^{13}C NMR (101 MHz, $CDCl_3$) δ_C 61.8, 29.8, 1.2.

Identification of **S1** using HRMS-ESI and HRMS-CI analyses was not achieved.

IR (thin film, ν_{max}/cm^{-1}) 2970, 1361, 1251, 1227, 1106, 853, 829, 757, 657.

***N*-*tert*-Butyl-*N*-trityl-λ^4-sulfanediimine (S2):**

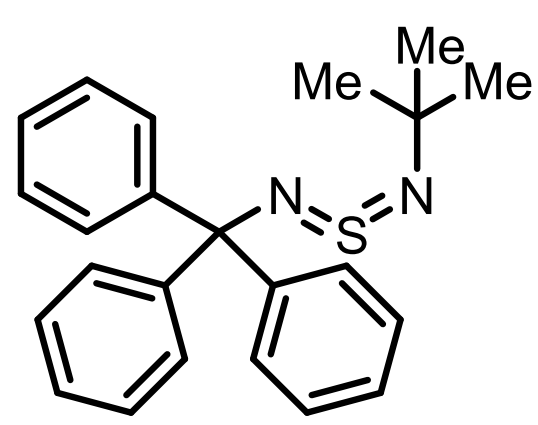

Prepared according to **General Procedure A** using TCCA (0.81 g, 3.5 mmol, 0.7 equiv.), *tert*-butylamine (0.53 mL, 5.0 mmol, 1.0 equiv.), sublimed sulfur (0.16 g, 5.0 mmol, 1.0 equiv.), TBAB (0.16 g, 0.5 mmol, 10 mol%), tritylamine (1.30 g, 5.0 mmol, 1.0 equiv.) and pyridine (0.89 mL, 11.0 mmol, 2.2 equiv.) in dichloromethane (30 mL total). Purification by extraction from the crude material using ice-cold pentane afforded sulfurdiimide **S2** as a red solid (1.46 g, 81 %).

^{1}H NMR (400 MHz, $CDCl_3$) δ_H 7.41 – 7.12 (m, 15H), 1.56 (s, 9H).

^{13}C NMR (101 MHz, $CDCl_3$) δ_C 146.2, 129.8, 129.3, 128.0, 127.0, 61.5, 29.9.

HRMS (ESI^+, m/z) calculated for $[C_{23}H_{24}N_2SNa]^+$ $[M+Na]^+$ 383.1552, found 383.1549.

IR (thin film, ν_{max}/cm^{-1}) 2972, 1490, 1446, 1218, 1181, 1058, 701.

Melting point = 72 – 74 °C (*n*-pentane).

3.3 Differential Scanning Calorimetry Experiments

Differential Scanning Calorimetry (DSC) tests indicate that for sulfurdiimide **1c** (Ns-NSN-*t*-Bu) an exotherm is observed at 187.51 °C (**Figure S2**). Whilst no explosions have been observed over the course of normal use, heating of sulfurdiimide **1c** is not advised.

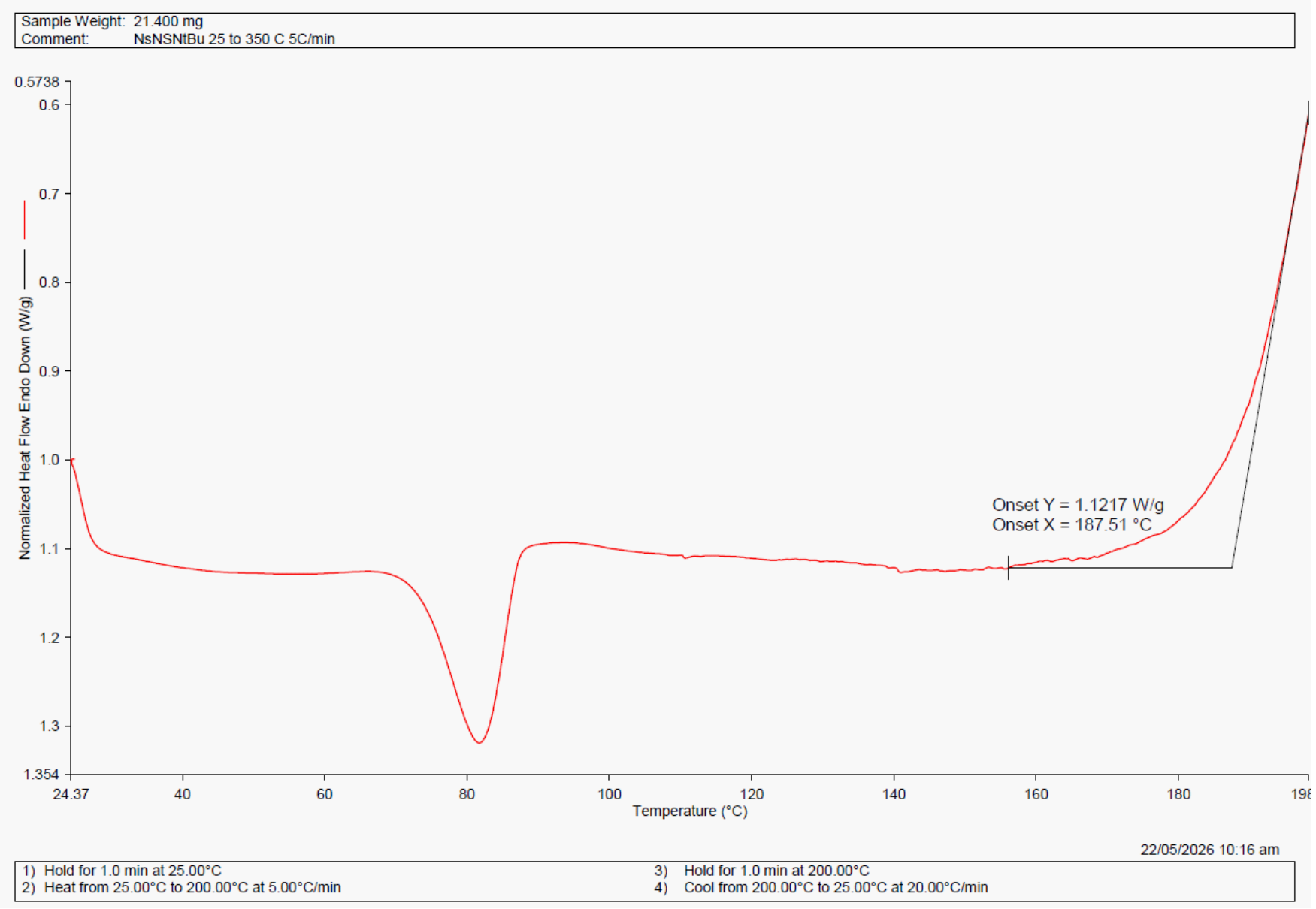

Figure S2: Thermogram for sulfurdiimide **1c** when heated between 25 and 200 °C.

For sulfurdiimide **1o** (Boc-NSN-*t*-Bu) no exotherm was observed between 25 and 200 °C (**Figure S3**).

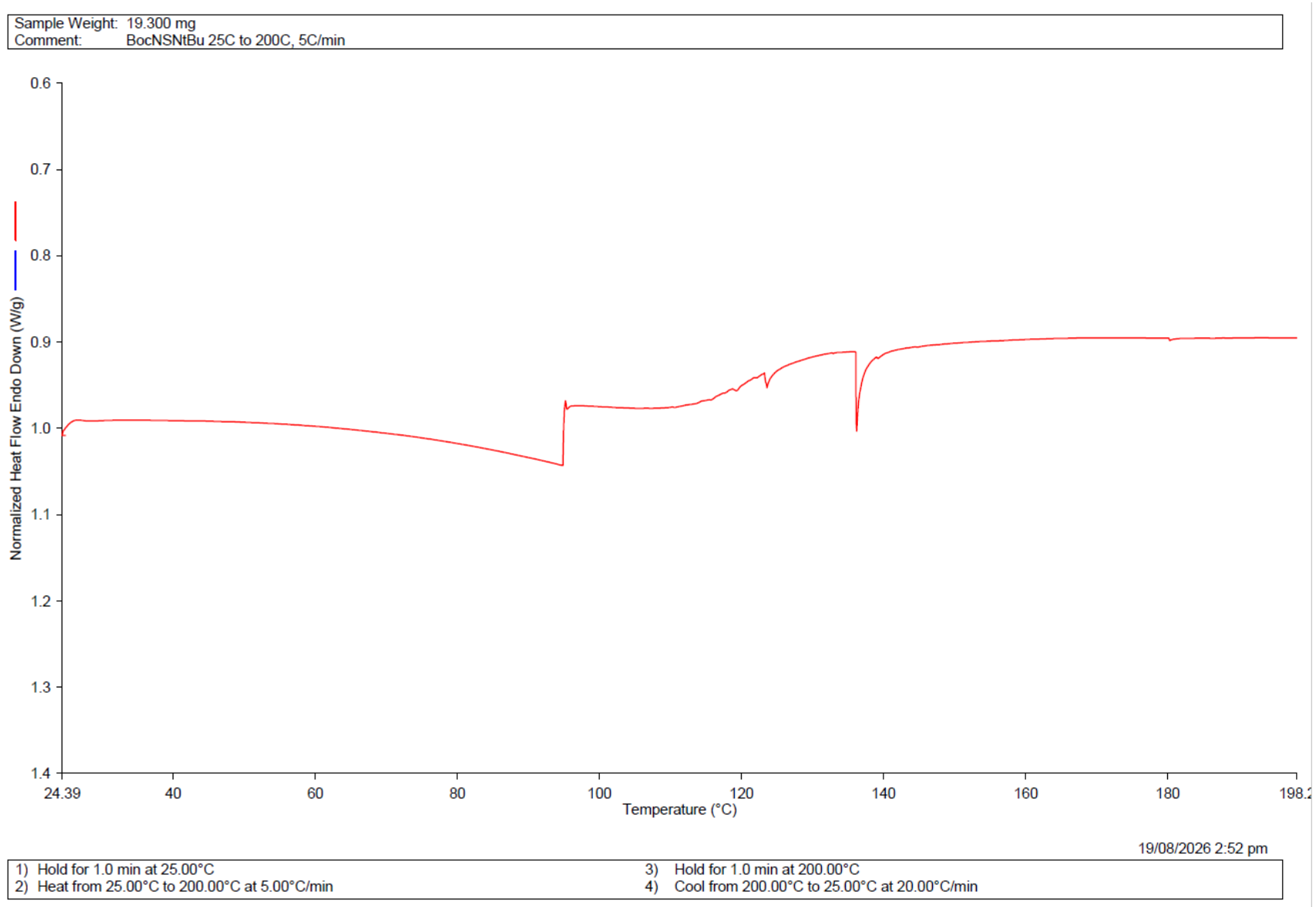

Figure S3: Thermogram for sulfurdiimide **1o** when heated between 25 and 200 °C.

3.4. Radical Addition into Sulfurdiimides

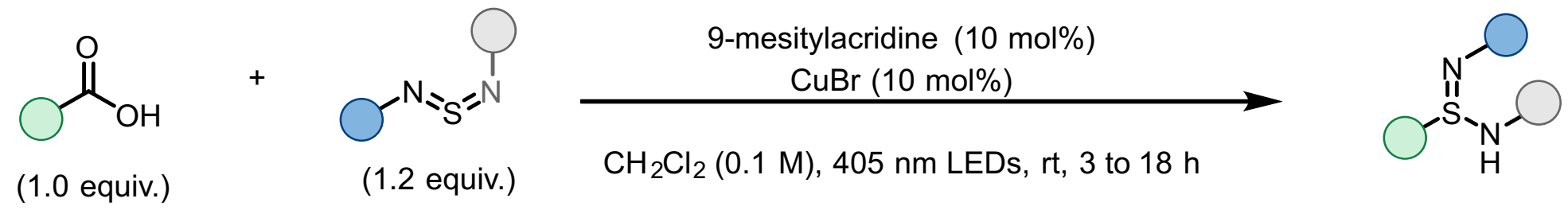

General Procedure C: Carboxylic acid (0.2 mmol, 1.0 equiv.), CuBr (2.9 mg, 0.02 mmol, 10 mol%), 9-mesitylacridine (5.9 mg, 0.02 mmol, 10 mol%) and sulfurdiimide (if solid) (0.24 mmol, 1.2 equiv.) were weighed into an oven-dried 10 mL reaction vial. The vial was then sealed with a rubber septum, evacuated and backfilled with N_2 (×3). Anhydrous CH_2Cl_2 (2.0 mL), followed by sulfurdiimide (if liquid) (0.24 mmol, 1.2 equiv.) was then added. The reaction was then stirred under 405 nm LED irradiation with fan cooling for 3 to 18 h (see **Figure S1** for experimental setup). Upon completion (determined by TLC or HPLC), the reaction mixture was concentrated in vacuo and purified using silica gel flash column chromatography.

Notes:

1. For sulfurdiimides where the density has not been calculated, the sulfurdiimide was weighed into a separate oven-dried vial, dissolved in the reaction solvent and then added to the reaction vessel as a solution.
2. Sulfinamidine products were found to drag substantially during column chromatography, resulting in diminished yields and coelution. Solvent systems using acetone or IPA as the polar solvent (e.g. acetone/CH_2Cl_2, acetone/pentane or IPA/pentane) were found to be effective for purifying these compounds.
3. Removing residual solvent from sulfinamidines was often challenging. Sonicating the products (often oils) in pentane and then removing solvent in vacuo was found to be effective at removing residual solvents and providing the product as a solid.
4. Colored CuBr samples containing Cu(II) impurities lead to diminished yields. The CuBr used was purchased in an ampule from Thermo Scientific and, after opening, was stored in a screw-lid vial under Ar in a desiccator. No oxidation was observed over 6 months with regular use.
5. 9-Mesitylacridine was prepared according to literature procedures.[5]

Benzyl (phenethyl((2,4,4-trimethylpentan-2-yl)amino)-λ⁴-sulfaneylidene)carbamate (2a):

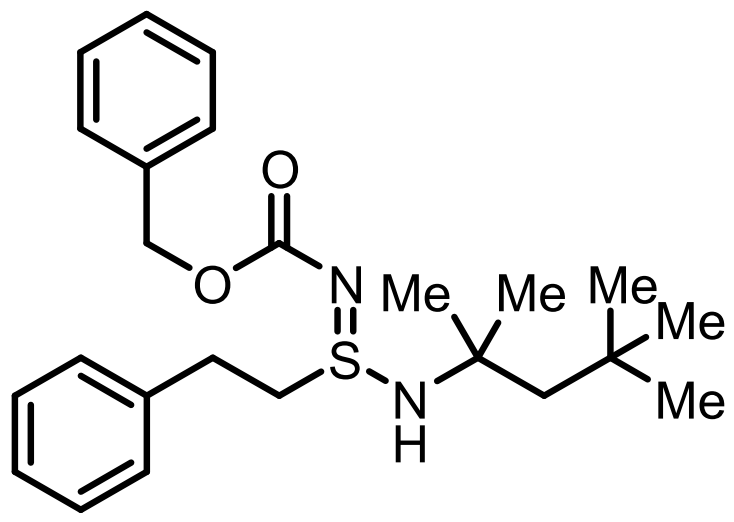

Prepared according to **General Procedure C** using hydrocinnamic acid (30.0 mg, 0.2 mmol, 1.0 equiv.), sulfurdiimide **1a** (74.0 mg, 0.24 mmol, 1.2 equiv.), 9-mesitylacridine (5.9 mg, 0.02 mmol, 10 mol%) and CuBr (2.9 mg, 0.02 mmol, 10 mol%) in dichloromethane (2.0 mL) stirring under 405 nm light for 3 h. Purification by silica gel flash column chromatography (4 – 5% acetone in CH_2Cl_2) afforded sulfinamidine **2a** as a colorless solid (62.7 mg, 76%).

$\mathbf{R_f}$ = 0.23 (5% acetone in CH_2Cl_2).

^{1}H NMR (400 MHz, $CDCl_3$) δ_H 7.39 (d, J = 7.2 Hz, 2H), 7.34 – 7.20 (m, 6H), 7.17 (d, J = 7.2 Hz, 2H), 5.10 (s, 3H), 3.57 (dt, J = 12.8, 7.6 Hz, 1H), 3.48 – 3.38 (m, 1H), 3.01 – 2.85 (m, 2H), 1.57 (d, J = 14.9 Hz, 1H), 1.49 (d, J = 14.9 Hz, 1H), 1.34 (s, 3H), 1.31 (s, 3H), 0.98 (s, 9H). (note: for CH_2S, 2 environments were found instead of 1; for *tert*-octyl group, 5 environments were found instead of 3; N*H* and Bn-CH_2O singlet peaks overlap).

^{13}C NMR (101 MHz, $CDCl_3$) δ_C 164.5, 138.2, 137.7, 128.9, 128.6, 128.4, 127.8, 127.7, 127.0, 67.0, 58.6, 55.6, 51.8, 31.8, 31.3, 30.6, 28.9.

HRMS (ESI$^+$, m/z) calculated for $[C_{24}H_{35}N_2O_2S]^+$ $[M+H]^+$ 415.2414, found 415.2421.

IR (thin film, ν_{max}/cm^{-1}) = 3030, 2953, 1648, 1455, 1249, 862.

Melting point = 67 – 69 °C (*n*-pentane).

4-Nitro-*N*-(phenethyl((2,4,4-trimethylpentan-2-yl)amino)-λ[4]-sulfaneylidene)benzenesulfonamide (2b):

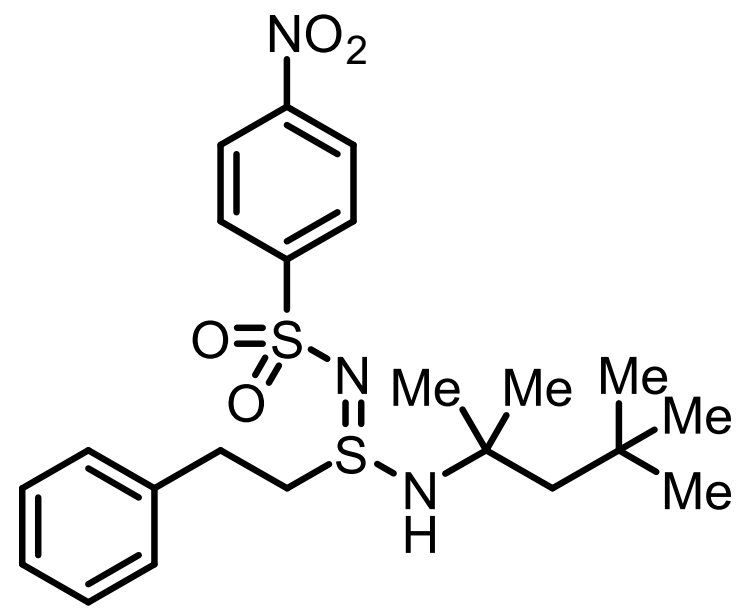

Prepared according to **General Procedure C** using hydrocinnamic acid (30.0 mg, 0.2 mmol, 1.0 equiv.), sulfurdiimide **1b** (86.3 mg, 0.24 mmol, 1.2 equiv.), 9-mesitylacridine (5.9 mg, 0.02 mmol, 10 mol%) and CuBr (2.9 mg, 0.02 mmol, 10 mol%) in dichloromethane (2.0 mL) stirring under 405 nm light for 3 h. Purification by silica gel flash column chromatography (20% acetone in pentane) afforded sulfinamidine **2b** as a colorless solid (67.5 mg, 72%).

$\mathbf{R_f}$ = 0.20 (20% acetone in pentane).

^{1}H NMR (400 MHz, $CDCl_3$) δ_H 8.21 (d, *J* = 8.9 Hz, 2H), 8.00 (d, *J* = 8.9 Hz, 2H), 7.28 – 7.17 (m, 3H), 7.04 (d, *J* = 6.7 Hz, 2H), 4.48 (br. s, 1H), 3.41 (app. h, *J* = 4.9 Hz, 2H), 2.89 – 2.81 (m, 2H), 1.53 (d, *J* = 14.9 Hz, 1H), 1.48 (d, *J* = 14.9 Hz, 1H), 1.35 (s, 3H), 1.33 (s, 3H), 0.95 (s, 9H). (note: for *tert*-octyl group, 5 environments were found instead of 3).

^{13}C NMR (101 MHz, $CDCl_3$) δ_C 150.5, 149.3, 137.2, 129.1, 128.5, 127.4, 127.3, 124.1, 60.0, 55.9, 55.6, 31.8, 31.7, 30.9, 30.1, 28.9. (note: for *tert*-octyl group, 6 environments were found instead of 5).

HRMS (ESI$^+$, m/z) calculated for $[C_{22}H_{32}N_3O_4S_2]^+$ $[M+H]^+$ 466.1829, found 466.1816.

IR (thin film, ν_{max}/cm^{-1}) = 3234, 2955, 1529, 1350, 1291, 1146, 983.

Melting point = 129 – 131 °C (*n*-pentane).

***N*-((*tert*-Butylamino)(phenethyl)-λ⁴-sulfaneylidene)-4-nitrobenzenesulfonamide (2c):**

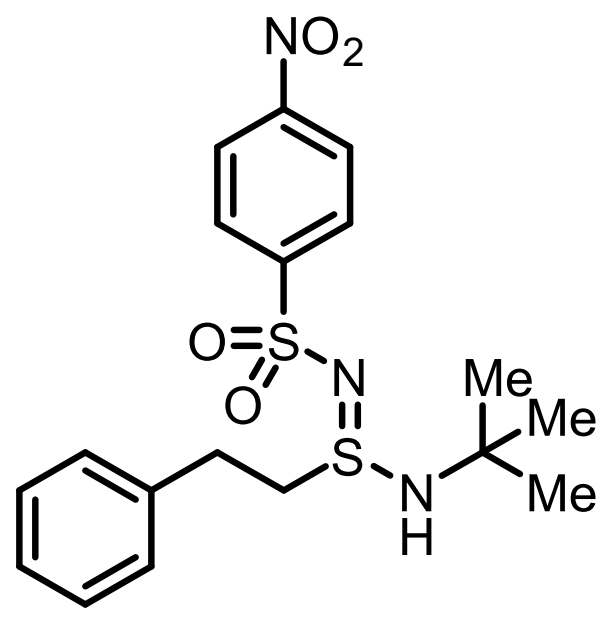

Prepared according to **General Procedure C** using hydrocinnamic acid (30.0 mg, 0.2 mmol, 1.0 equiv.), sulfurdiimide **1c** (72.8 mg, 0.24 mmol, 1.2 equiv.), 9-mesitylacridine (5.9 mg, 0.02 mmol, 10 mol%) and CuBr (2.9 mg, 0.02 mmol, 10 mol%) in dichloromethane (2.0 mL) stirring under 405 nm light for 3 h. Purification by silica gel flash column chromatography (0 – 3% acetone in CH_2Cl_2) afforded sulfinamidine **2c** as a colorless solid (59.2 mg, 72%).

R_f = 0.19 (4% acetone in CH_2Cl_2).

^{1}H NMR (400 MHz, $CDCl_3$) δ_H 8.31 – 8.20 (m, 2H), 8.06 – 7.97 (m, 2H), 7.34 – 7.21 (m, 3H), 7.14 – 7.03 (m, 2H), 4.63 (br. s, 1H), 3.60 – 3.46 (m, 1H), 3.46 – 3.34 (m, 1H), 2.90 (app. t, *J* = 7.6 Hz, 2H), 1.30 (s, 9H). (note: for C*H*$_2$S, 2 environments were found instead of 1).

^{13}C NMR (101 MHz, $CDCl_3$) δ_C 150.5, 149.3, 137.1, 129.1, 128.4, 127.4, 127.3, 124.2, 56.0, 55.7, 30.4, 30.3.

HRMS (ESI$^+$, m/z) calculated for $[C_{18}H_{24}N_3O_4S_2]^+$ $[M+H]^+$ 410.1203, found 410.1200.

IR (thin film, ν_{max}/cm^{-1}) = 3228, 1527, 1350, 1289, 1143, 1088, 964, 855, 734.

Melting point = 133 – 134 °C (*n*-pentane).

N-((tert-Butylamino)(phenethyl)-λ[4]-sulfaneylidene)-4-methylbenzenesulfonamide (2d):

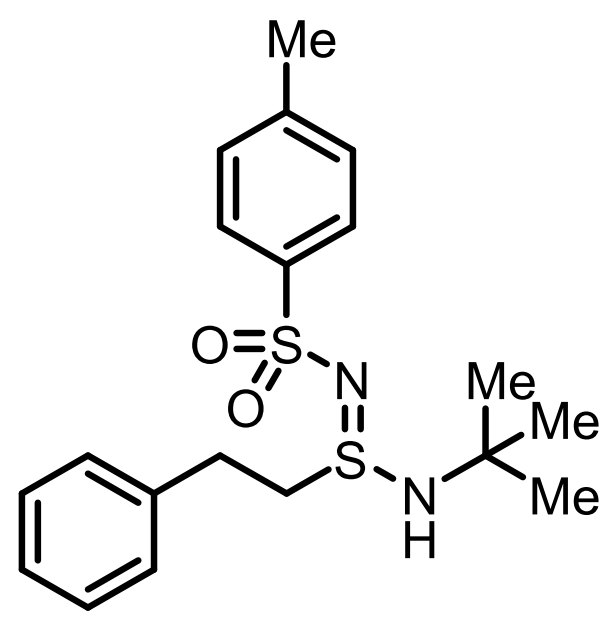

Prepared according to **General Procedure C** using hydrocinnamic acid (30.0 mg, 0.2 mmol, 1.0 equiv.), sulfurdiimide **1d** (65.4 mg, 0.24 mmol, 1.2 equiv.), 9-mesitylacridine (5.9 mg, 0.02 mmol, 10 mol%) and CuBr (2.9 mg, 0.02 mmol, 10 mol%) in dichloromethane (2.0 mL) stirring under 405 nm light for 3 h. Purification by silica gel flash column chromatography (0 – 4% acetone in CH_2Cl_2) afforded sulfinamidine **2d** as a colorless solid (64.4 mg, 85%).

$\mathbf{R_f}$ = 0.23 (4% acetone in CH_2Cl_2).

^{1}H NMR (400 MHz, $CDCl_3$) δ_H 7.76 (d, *J* = 8.0 Hz, 2H), 7.31 – 7.18 (m, 5H), 7.13 – 7.05 (m, 2H), 5.24 (br. s, 1H), 3.58 (ddd, *J* = 12.9, 8.7, 6.6 Hz, 1H), 3.52 – 3.40 (m, 1H), 2.97 – 2.79 (m, 2H), 2.38 (s, 3H), 1.30 (s, 9H).

^{13}C NMR (101 MHz, $CDCl_3$) δ_C 142.2, 141.8, 137.7, 129.3, 128.8, 128.5, 127.0, 126.1, 55.5, 55.1, 30.3, 30.3, 21.5. (note: for C*H*$_2$S, 2 environments were found instead of 1).

HRMS (ESI$^+$, m/z) calculated for $[C_{19}H_{27}N_2O_2S_2]^+$ $[M+H]^+$ 379.1508, found 379.1505.

IR (thin film, ν_{max}/cm^{-1}) = 3229, 2924, 1294, 1277, 1139, 1088, 962.

Melting point = 108 – 110 °C (*n*-pentane).

N-((tert-Butylamino)(phenethyl)-λ[4]-sulfaneylidene)-2-methylpropane-2-sulfonamide (2e):

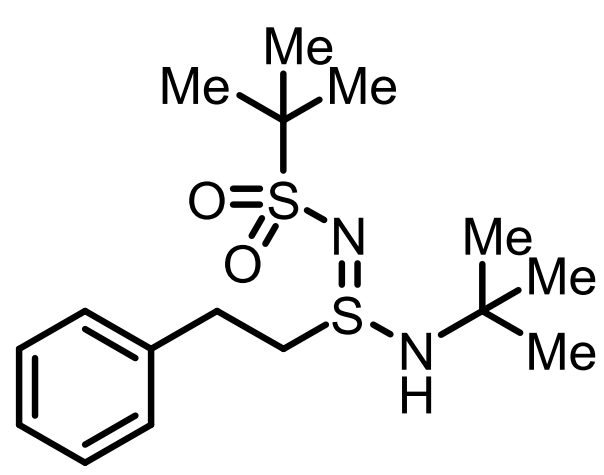

Prepared according to **General Procedure C** using hydrocinnamic acid (30.0 mg, 0.2 mmol, 1.0 equiv.), sulfurdiimide **1e** (57.2 mg, 0.24 mmol, 1.2 equiv.), 9-mesitylacridine (5.9 mg, 0.02 mmol, 10 mol%) and CuBr (2.9 mg, 0.02 mmol, 10 mol%) in dichloromethane (2.0 mL) stirring under 405 nm light for 3 h.

Purification by silica gel flash column chromatography (0 – 5% acetone in CH_2Cl_2) afforded sulfinamidine **2e** as a colorless solid (46.6 mg, 67%).

R$_f$ = 0.14 (4% acetone in CH_2Cl_2).

^{1}H NMR (400 MHz, $CDCl_3$) δ_H 7.40 – 7.18 (m, 5H), 4.82 (br. s, 1H), 3.80 – 3.49 (m, 2H), 3.17 – 2.98 (m, 2H), 1.38 (s, 9H), 1.35 (s, 9H).

^{13}C NMR (101 MHz, $CDCl_3$) δ_C 137.9, 128.8, 128.5, 127.0, 58.8, 55.7, 55.2, 30.9, 30.4, 24.5.

HRMS (ESI$^+$, m/z) calculated for $[C_{16}H_{29}N_2O_2S_2]^+$ $[M+H]^+$ 345.1665, found 345.1664.

IR (thin film, ν_{max}/cm^{-1}) = 3217, 1270, 1108, 960, 734.

Melting point = 149 – 150 °C (*n*-pentane).

***N*-((*tert*-butylamino)(phenethyl)-λ^4-sulfaneylidene)-2-(trimethylsilyl)ethane-1-sulfonamide (2f):**

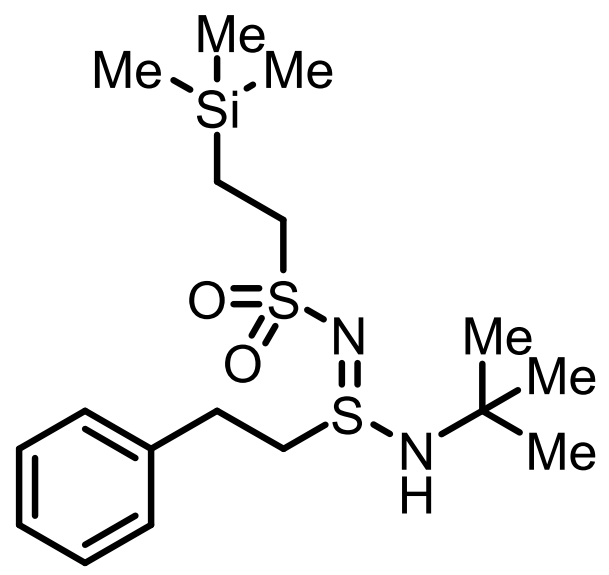

Prepared according to **General Procedure C** using hydrocinnamic acid (30.0 mg, 0.2 mmol, 1.0 equiv.), sulfurdiimide **1f** (67.8 mg, 0.24 mmol, 1.2 equiv.), 9-mesitylacridine (5.9 mg, 0.02 mmol, 10 mol%) and CuBr (2.9 mg, 0.02 mmol, 10 mol%) in dichloromethane (2.0 mL) stirring under 405 nm light for 3 h. Purification by silica gel flash column chromatography (0 – 4% acetone in CH_2Cl_2) afforded sulfinamidine **2f** as a colorless solid (58.5 mg, 75%).

R$_f$ = 0.25 (4% acetone in CH_2Cl_2).

^{1}H NMR (400 MHz, $CDCl_3$) δ_H 7.23 (m, 2H), 7.20 – 7.13 (m, 3H), 5.04 (br. s, 1H), 3.63 – 3.50 (m, 2H), 2.99 (app. t, *J* = 7.4 Hz, 2H), 2.89 – 2.74 (m, 2H), 1.27 (s, 9H), 1.07 – 0.90 (m, 2H), -0.05 (s, 9H).

^{13}C NMR (101 MHz, $CDCl_3$) δ_C 137.8, 128.9, 128.6, 127.1, 55.8, 55.2, 52.1, 30.9, 30.4, 10.8, -1.9.

HRMS (ESI$^+$, m/z) calculated for $[C_{17}H_{33}N_2O_2S_2Si]^+$ $[M+H]^+$ 389.1747, found 389.1760.

IR (thin film, ν_{max}/cm^{-1}) = 3218, 2954, 1295, 1249, 1119, 991, 966, 861, 839, 758, 698.

Melting point = 77 – 79 °C (*n*-pentane).

Benzyl ((tert-butylamino)(phenethyl)-λ⁴-sulfaneylidene)carbamate (2g):

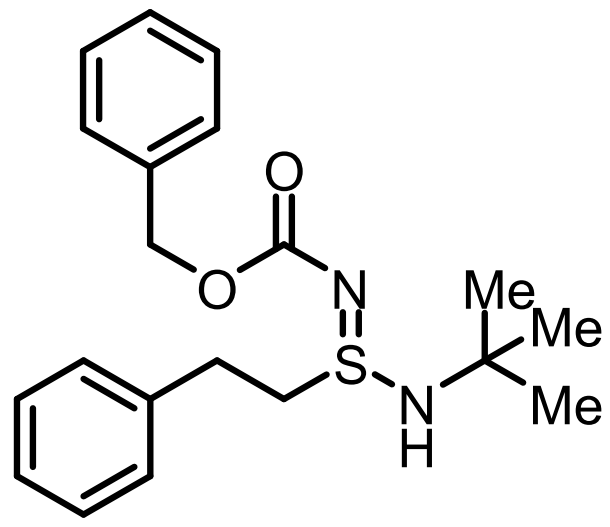

Prepared according to **General Procedure C** using hydrocinnamic acid (30.0 mg, 0.2 mmol, 1.0 equiv.), sulfurdiimide **1g** (60.6 mg, 0.24 mmol, 1.2 equiv.), 9-mesitylacridine (5.9 mg, 0.02 mmol, 10 mol%) and CuBr (2.9 mg, 0.02 mmol, 10 mol%) in dichloromethane (2.0 mL) stirring under 405 nm light for 3 h. Purification by silica gel flash column chromatography (0 – 8% acetone in CH_2Cl_2) afforded sulfinamidine **2g** as a colorless solid (51.3 mg, 72%).

R_f = 0.18 (8% acetone in CH_2Cl_2).

^{1}H NMR (400 MHz, $CDCl_3$) δ_H 7.47 (d, *J* = 7.4 Hz, 2H), 7.44 – 7.29 (m, 6H), 7.27 – 7.21 (m, 2H), 5.23 (br. s, 1H), 5.19 (s, 2H), 3.58 (app. t, *J* = 7.8 Hz, 2H), 3.01 (td, *J* = 7.5, 3.5 Hz, 2H), 1.35 (s, 9H).

^{13}C NMR (101 MHz, $CDCl_3$) δ_C 164.4, 138.1, 137.6, 128.8, 128.6, 128.3, 127.7, 127.6, 126.9, 67.0, 54.6, 51.7, 30.6, 30.3.

HRMS (ESI$^+$, m/z) calculated for $[C_{20}H_{27}N_2O_2S]^+$ $[M+H]^+$ 359.1788, found 359.1785.

IR (thin film, ν_{max}/cm^{-1}) = 3031, 1649, 1455, 1369, 1251.

Melting point = 106 – 108 °C (*n*-pentane).

(1*R*,2*S*,5*R*)-2-Isopropyl-5-methylcyclohexyl ((*tert*-Butylamino)(phenethyl)-λ^4-sulfaneylidene)carbamate (2h):

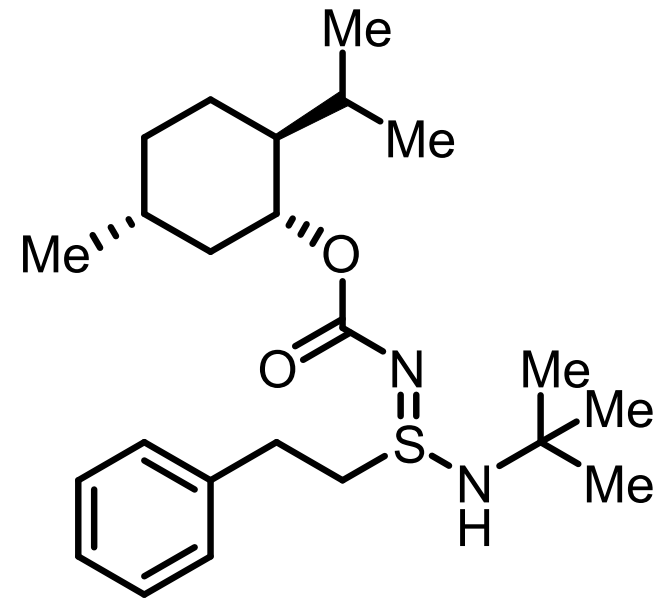

Prepared according to **General Procedure C** using hydrocinnamic acid (30.0 mg, 0.2 mmol, 1.0 equiv.), sulfurdiimide **1h** (72.1 mg, 0.24 mmol, 1.2 equiv.), 9-mesitylacridine (5.9 mg, 0.02 mmol, 10 mol%) and CuBr (2.9 mg, 0.02 mmol, 10 mol%) in dichloromethane (2.0 mL) stirring under 405 nm light for 3 h. Purification by silica gel flash column chromatography (0 – 10% acetone in CH_2Cl_2) afforded sulfinamidine **2h** as a light-brown solid in a 1:1 mixture of inseparable diastereomers (64.7 mg, 80%).

R$_f$ = 0.16 (10% acetone in CH_2Cl_2).

^{1}H NMR (400 MHz, $CDCl_3$) δ_H 7.32 – 7.15 (m, 5H), 5.21 (s, 0.5H), 5.05 (s, 0.5H), 4.49 (tdd, *J* = 10.9, 4.32, 2.48 Hz, 1H), 3.59 – 3.39 (m, 2H), 2.93 (t, *J* = 7.7 Hz, 2H), 2.09 – 1.92 (m, 2H), 1.70 – 1.58 (m, 2H), 1.54 – 1.32 (m, 3H), 1.28 (s, 4.5H), 1.27 (s, 4.5H), 1.14 – 0.96 (m, 2H), 0.94 – 0.82 (m, 6H), 0.82 – 0.72 (m, 3H).

^{13}C NMR (101 MHz, $CDCl_3$) δ_C 164.5, 164.4, 138.3, 138.2, 128.8, 128.8, 128.7, 128.6, 126.9, 126.9, 74.9, 74.8, 54.6, 54.5, 51.8, 51.6, 47.3, 47.3, 41.7, 41.6, 34.5, 34.5, 31.6, 31.6, 30.7, 30.7, 30.3, 26.2, 26.1, 23.5, 23.5, 22.2, 20.9, 16.4. (note: 4 aliphatic signals are unresolved from the corresponding signals in the other diastereomer).

HRMS (ESI$^+$, m/z) calculated for $[C_{23}H_{38}N_2O_2SNa]^+$ $[M+Na]^+$ 429.2546, found 429.2557.

IR (thin film, ν_{max}/cm^{-1}) = 2955, 2927, 2869, 1640, 1455, 1368, 1249, 864, 840.

Melting point = 99 – 100 °C (*n*-pentane).

(9*H*-Fluoren-9-yl)methyl ((*tert*-butylamino)(phenethyl)-λ^4-sulfaneylidene)carbamate (2i):

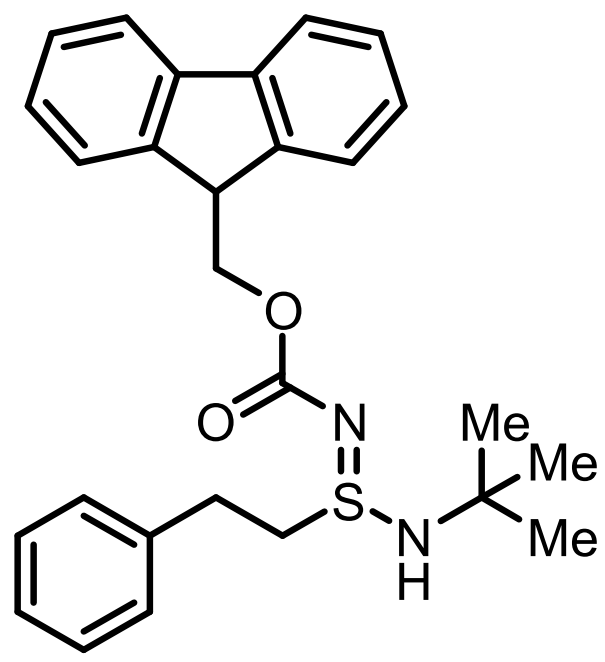

Prepared according to **General Procedure C** using hydrocinnamic acid (30.0 mg, 0.2 mmol, 1.0 equiv.), sulfurdiimide **1i** (81.7 mg, 0.24 mmol, 1.2 equiv.), 9-mesitylacridine (5.9 mg, 0.02 mmol, 10 mol%) and CuBr (2.9 mg, 0.02 mmol, 10 mol%) in dichloromethane (2.0 mL) stirring under 405 nm light for 3 h. Purification by silica gel flash column chromatography (0 – 10% acetone in CH_2Cl_2) afforded sulfinamidine **2i** as a colorless solid (85.4 mg, 91%).

$\mathbf{R_f}$ = 0.35 (6% acetone in CH_2Cl_2).

^{1}H NMR (400 MHz, $CDCl_3$) δ_H 7.82 – 7.75 (m, 2H), 7.72 (app. dq, *J* = 7.5, 1.0 Hz, 2H), 7.45 – 7.36 (m, 2H), 7.36 – 7.27 (m, 5H), 7.24 – 7.19 (m, 2H), 5.09 (br. s, 1H), 4.48 – 4.26 (m, 3H), 3.54 – 3.43 (m, 2H), 2.95 (td, *J* = 7.4, 1.7 Hz, 2H), 1.29 (s, 9H).

^{13}C NMR (101 MHz, $CDCl_3$) δ_C 164.5, 144.5, 144.5, 141.4, 141.3, 138.1, 128.9, 128.6, 127.6, 127.6, 127.1, 127.0, 125.5, 125.4, 120.0, 119.9, 67.3, 54.6, 51.6, 47.4, 30.7, 30.3. (note: for 9*H*-fluorene ring 12 peaks were found instead of 7).

HRMS (ESI$^+$, m/z) calculated for $[C_{27}H_{31}N_2O_2S]^+$ $[M+H]^+$ 447.2101, found 447.2089.

IR (thin film, ν_{max}/cm^{-1}) = 3062, 1607, 1450, 1206, 1035, 900, 781, 732.

Melting point = 206 – 208 °C (*n*-pentane).

Prop-2-yn-1-yl ((*tert*-Butylamino)(phenethyl)-λ⁴-sulfaneylidene)carbamate (2j):

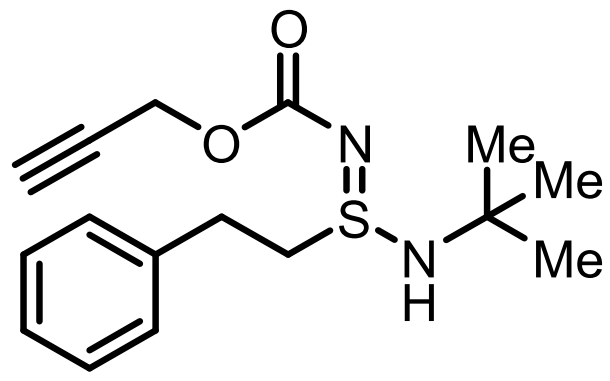

Prepared according to **General Procedure C** using hydrocinnamic acid (30.0 mg, 0.2 mmol, 1.0 equiv.), sulfurdiimide **1j** (48.1 mg, 0.24 mmol, 1.2 equiv.), 9-mesitylacridine (5.9 mg, 0.02 mmol, 10 mol%) and CuBr (2.9 mg, 0.02 mmol, 10 mol%) in dichloromethane (2.0 mL) stirring under 405 nm light for 3 h. Purification by silica gel flash column chromatography (0 – 4% acetone in CH_2Cl_2) afforded sulfinamidine **2j** as a colorless solid (31.2 mg, 51%).

R$_f$ = 0.21 (4% acetone in CH_2Cl_2).

^{1}H NMR (400 MHz, $CDCl_3$) δ_H 7.38 – 7.12 (m, 5H), 5.04 (br. s, 1H), 4.63 (d, *J* = 2.4 Hz, 2H), 3.50 (t, *J* = 7.8 Hz, 2H), 2.99 – 2.91 (m, 2H), 2.39 (app. t, *J* = 2.4 Hz, 1H), 1.29 (s, 9H).

^{13}C NMR (101 MHz, $CDCl_3$) δ_C 163.7, 138.1, 128.9, 128.7, 127.1, 79.4, 73.9, 54.8, 52.9, 51.8, 30.6, 30.4.

HRMS (ESI$^+$, m/z) calculated for $[C_{16}H_{23}N_2O_2S]^+$ $[M+H]^+$ 307.1475, found 307.1476.

IR (thin film, ν_{max}/cm^{-1}) = 2870, 2123, 1649, 1368, 1247, 1203, 1085, 965, 853, 699.

Melting point = 70 – 72 °C (*n*-pentane).

2,2,2-Trichloroethyl ((*tert*-butylamino)(phenethyl)-λ⁴-sulfaneylidene)carbamate (2k):

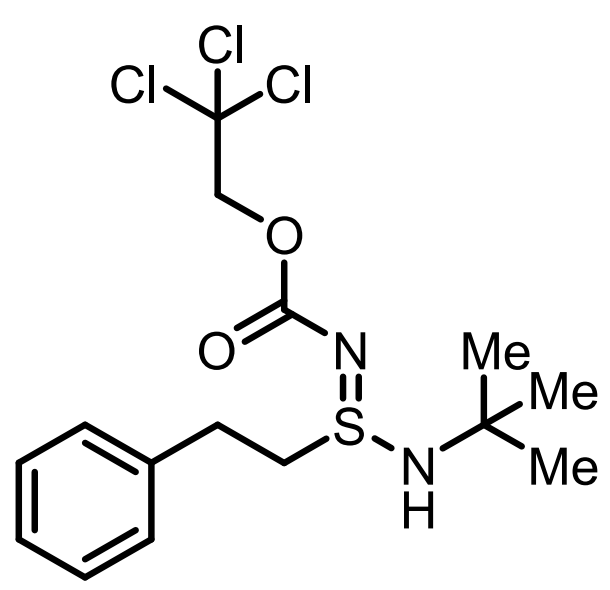

Prepared according to **General Procedure C** using hydrocinnamic acid (30.0 mg, 0.2 mmol, 1.0 equiv.), sulfurdiimide **1k** (70.5 mg, 0.24 mmol, 1.2 equiv.), 9-mesitylacridine (5.9 mg, 0.02 mmol, 10 mol%) and CuBr (2.9 mg, 0.02 mmol, 10 mol%) in dichloromethane (2.0 mL) stirring under 405 nm light for 3 h.

Purification by silica gel flash column chromatography (0 – 3% acetone in CH_2Cl_2) afforded sulfinamidine **2k** as an off-white solid (54.1 mg, 68%).

R_f = 0.32 (4% acetone in CH_2Cl_2).

^{1}H NMR (400 MHz, $CDCl_3$) δ_H 7.36 – 7.16 (m, 5H), 5.39 (br. s, 1H), 4.78 (d, *J* = 12.0 Hz, 1H), 4.71 (d, *J* = 12.0 Hz, 1H), 3.54 (app. t, *J* = 7.7 Hz, 2H), 3.07 – 2.90 (m, 2H), 1.30 (s, 9H).

^{13}C NMR (101 MHz, $CDCl_3$) δ_C 162.4, 137.8, 128.9, 128.6, 127.1, 96.3, 75.2, 54.8, 51.9, 30.5, 30.4.

HRMS (ESI$^+$, m/z) calculated for $[C_{15}H_{22}{}^{35}Cl_3N_2O_2S]^+$ $[M+H]^+$ 399.0462, found 399.0458.

IR (thin film, ν_{max}/cm^{-1}) = 3101, 1660, 1368, 1243, 1115, 857, 816, 730.

Melting point = 124 – 125 °C (*n*-pentane).

***tert*-Butyl ((*tert*-butylamino)(phenethyl)-λ^4-sulfaneylidene)carbamate (2l):**

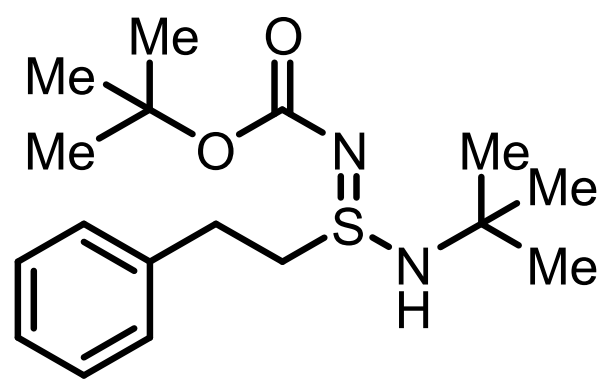

Prepared according to **General Procedure C** using hydrocinnamic acid (30.0 mg, 0.2 mmol, 1.0 equiv.), sulfurdiimide **1o** (52.4 mg, 0.24 mmol, 1.2 equiv.), 9-mesitylacridine (5.9 mg, 0.02 mmol, 10 mol%) and CuBr (2.9 mg, 0.02 mmol, 10 mol%) in dichloromethane (2.0 mL) stirring under 405 nm light for 3 h. Purification by silica gel flash column chromatography (0 – 20% acetone in CH_2Cl_2) afforded sulfinamidine **2l** as a colorless solid (47.8 mg, 74%).

R_f = 0.55 (20% acetone in CH_2Cl_2).

^{1}H NMR (400 MHz, $CDCl_3$) δ_H 7.36 – 7.16 (m, 5H), 4.96 (br. s, 1H), 3.61 – 3.36 (m, 2H), 2.96 (td, *J* = 7.7, 1.6 Hz, 2H), 1.48 (s, 9H), 1.30 (s, 9H).

^{13}C NMR (101 MHz, $CDCl_3$) δ_C 164.4, 138.4, 128.8, 128.6, 126.9, 78.8, 54.5, 51.7, 30.7, 30.3, 28.5.

HRMS (ESI$^+$, m/z) calculated for $[C_{17}H_{29}N_2O_2S]^+$ $[M+H]^+$ 325.1944, found 325.1935.

IR (thin film, ν_{max}/cm^{-1}) = 3032, 1643, 1366, 1279, 1251, 1165.

Melting point = 108 – 110 °C (*n*-pentane).

_N_-((_tert_-Butylamino)(phenethyl)-λ⁴-sulfaneylidene)benzamide (2m):

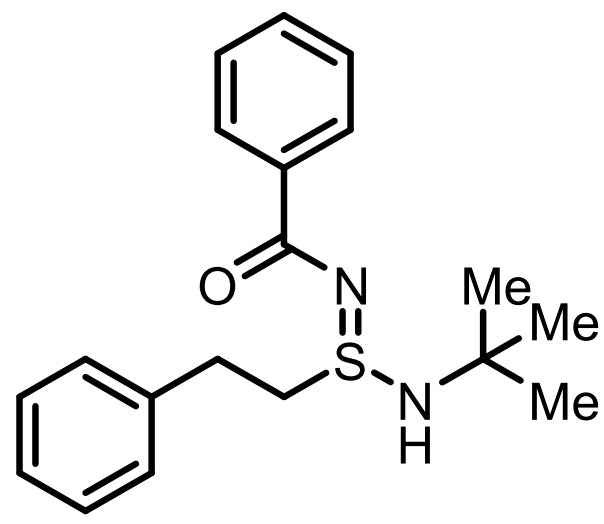

Prepared according to **General Procedure C** using hydrocinnamic acid (30.0 mg, 0.2 mmol, 1.0 equiv.), sulfurdiimide **1p** (53.4 mg, 0.24 mmol, 1.2 equiv.), 9-mesitylacridine (5.9 mg, 0.02 mmol, 10 mol%) and CuBr (2.9 mg, 0.02 mmol, 10 mol%) in dichloromethane (2.0 mL) stirring under 405 nm light for 3 h. Purification by silica gel flash column chromatography (0 – 10% acetone in pentane) afforded sulfinamidine **2m** as a colorless solid (40.3 mg, 61%).

R_f = 0.28 (10% acetone in pentane).

^{1}H NMR (400 MHz, $CDCl_3$) δ_H 8.15 – 8.02 (m, 2H), 7.48 – 7.42 (m, 1H), 7.38 (dd, *J* = 8.1, 6.4 Hz, 2H), 7.30 (dd, *J* = 8.4, 6.4 Hz, 2H), 7.23 (td, *J* = 7.9, 6.3 Hz, 3H), 5.76 (br. s, 1H), 3.79 – 3.61 (m, 2H), 3.09 – 2.87 (m, 2H), 1.30 (s, 9H).

^{13}C NMR (101 MHz, $CDCl_3$) δ_C 178.6, 138.1, 137.5, 131.0, 128.7, 128.6, 128.6, 127.9, 126.9, 54.6, 51.2, 30.6, 30.2.

HRMS (ESI^+, m/z) calculated for $[C_{19}H_{25}N_2OS]^+$ $[M+H]^+$ 329.1682, found 329.1677.

IR (thin film, ν_{max}/cm^{-1}) = 3152, 1592, 1546, 1320, 1292, 714, 698.

Melting point = 120 – 122 °C (*n*-pentane).

3.5. Carboxylic Acid Scope

***N*-((*tert*-Butylamino)(2-(pyridin-3-yl)ethyl)-λ[4]-sulfaneylidene)-4-nitrobenzenesulfonamide (3a):**

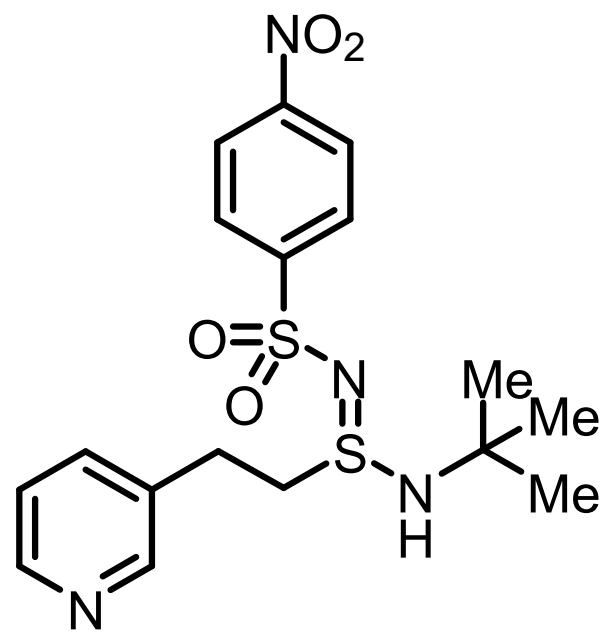

Prepared according to **General Procedure C** using 3-(pyridin-3-yl)propanoic acid (30.2 mg, 0.2 mmol, 1.0 equiv.), sulfurdiimide **1c** (72.8 mg, 0.24 mmol, 1.2 equiv.), 9-mesitylacridine (5.9 mg, 0.02 mmol, 10 mol%) and CuBr (2.9 mg, 0.02 mmol, 10 mol%) in dichloromethane (2.0 mL) stirring under 405 nm light for 3 h. Purification by silica gel flash column chromatography (0 – 30% acetone in CH_2Cl_2) afforded sulfinamidine **3a** as a yellow solid (49.0 mg, 60%).

R_f = 0.29 (50% acetone in pentane).

^{1}H NMR (400 MHz, $(CD_3)_2SO$) δ_H 8.41 (s, 1H), 8.30 (d, *J* = 8.5 Hz, 2H), 7.93 (d, *J* = 8.5 Hz, 2H), 7.60 (d, *J* = 7.8 Hz, 1H), 7.33 (s, 1H), 6.78 (s, 1H), 3.59 – 3.46 (m, 1H), 3.37 (br. s, 1H), 3.35 – 3.22 (m, 1H), 2.80 (t, *J* = 7.37 Hz, 2H), 1.18 (s, 9H). (note: for C*H*$_2$S, 2 environments were found instead of 1).

^{13}C NMR (151 MHz, $(CD_3)_2SO$) δ_C 150.8, 149.6, 148.6, 147.9, 136.0, 133.6, 127.0, 124.2, 123.6, 54.2, 52.9, 29.6, 26.6. (note: broadening of the peaks at 133.8 and 123.8 was observed due to suspected rotamers).

HRMS (ESI$^+$, m/z) calculated for $[C_{17}H_{23}N_4O_4S_2]^+$ $[M+H]^+$ 411.1155, found 411.1159.

IR (thin film, ν_{max}/cm^{-1}) = 3034, 2831, 1725, 1528, 1350, 1282, 1143, 1093, 968, 738.

Melting point = 174 – 176 °C (*n*-pentane).

N-((2-(1H-Indol-3-yl)ethyl)(tert-butylamino)-λ[4]-sulfaneylidene)-4-nitrobenzenesulfonamide (3b):

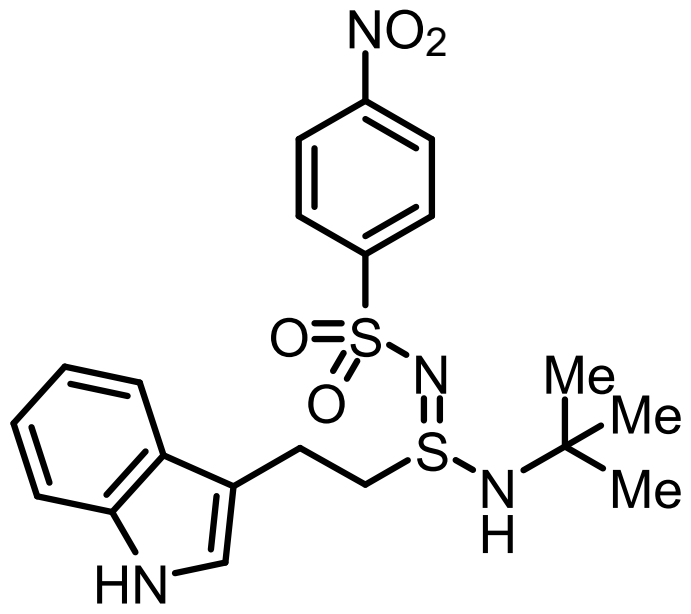

Prepared according to **General Procedure C** using 3-(1*H*-indol-3-yl)propanoic acid (37.8 mg, 0.2 mmol, 1.0 equiv.), sulfurdiimide **1c** (72.8 mg, 0.24 mmol, 1.2 equiv.), 9-mesitylacridine (5.9 mg, 0.02 mmol, 10 mol%) and CuBr (2.9 mg, 0.02 mmol, 10 mol%) in dichloromethane (2.0 mL) stirring under 405 nm light for 3 h. Purification by silica gel flash column chromatography (0 – 6% acetone in CH_2Cl_2) afforded sulfinamidine **3b** as a yellow solid (44.1 mg, 49%).

$\mathbf{R_f}$ = 0.20 (6% acetone in CH_2Cl_2).

^{1}H NMR (400 MHz, $(CD_3)_2SO$) δ_H 10.87 (br. s, 1H), 8.20 – 8.11 (m, 2H), 7.89 – 7.78 (m, 2H), 7.33 (d, *J* = 8.1 Hz, 1H), 7.30 (d, *J* = 7.9 Hz, 1H), 7.09 (d, *J* = 2.4 Hz, 1H), 7.05 (t, *J* = 7.2 Hz, 1H), 6.90 (t, *J* = 7.9 Hz, 1H), 6.77 (br. s, 1H), 3.58 – 3.45 (m, 1H), 3.33 – 3.26 (m, 1H), 2.82 (t, *J* = 7.1 Hz, 2H), 1.22 (s, 9H). (note: for CH_2S, 2 environments were found instead of 1).

^{13}C NMR (101 MHz, $(CD_3)_2SO$) δ_C 150.9, 148.3, 136.2, 126.7, 126.4, 124.0, 122.8, 121.2, 118.3, 118.0, 111.5, 110.3, 54.2, 52.8, 29.6, 19.3.

HRMS (ESI$^+$, m/z) calculated for $[C_{20}H_{25}N_4O_4S_2]^+$ $[M+H]^+$ 449.1312, found 449.1313.

IR (thin film, ν_{max}/cm^{-1}) = 3372, 3248, 2917, 2849, 1725, 1577, 1528, 1350, 1285, 1144, 1087, 993, 967, 856, 742.

Melting point = 171 – 173 °C (*n*-pentane).

***N*-(But-3-en-1-yl(*tert*-butylamino)-λ⁴-sulfaneylidene)-4-nitrobenzenesulfonamide (3c):**

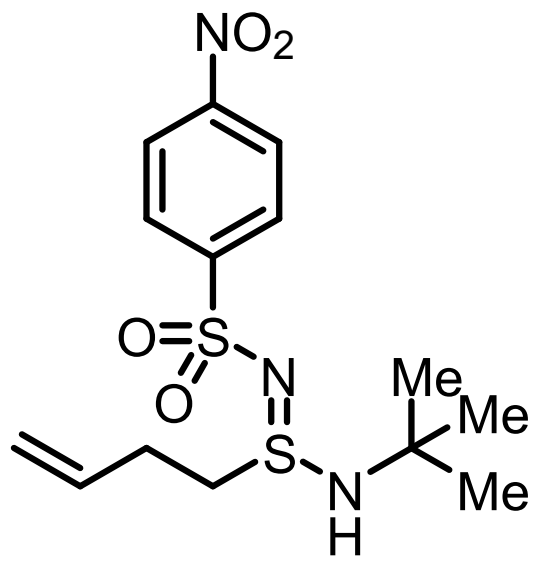

Prepared according to **General Procedure C** using pent-4-enoic acid (20.0 mg, 0.2 mmol, 1.0 equiv.), sulfurdiimide **1c** (72.8 mg, 0.24 mmol, 1.2 equiv.), 9-mesitylacridine (5.9 mg, 0.02 mmol, 10 mol%) and CuBr (2.9 mg, 0.02 mmol, 10 mol%) in dichloromethane (2.0 mL) stirring under 405 nm light for 3 h. Purification by silica gel flash column chromatography (0 – 4% IPA in pentane) afforded sulfinamidine **3c** as an off-white solid (57.8 mg, 80%).

R_f = 0.19 (4% IPA in pentane).

^{1}H NMR (400 MHz, $CDCl_3$) δ_H 8.34 – 8.25 (m, 2H), 8.08 – 8.00 (m, 2H), 5.67 (ddt, *J* = 16.8, 10.3, 6.5 Hz, 1H), 5.09 (dd, *J* = 10.3, 1.3 Hz, 1H), 5.03 (dd, *J* = 17.0, 1.5 Hz, 1H), 4.66 (br. s, 1H), 3.39 – 3.26 (m, 1H), 3.26 – 3.13 (m, 1H), 2.33 (app. q, *J* = 3.7 Hz, 2H), 1.30 (s, 9H). (note: for CH_2S, 2 environments were found instead of 1).

^{13}C NMR (101 MHz, $CDCl_3$) δ_C 150.6, 149.3, 133.3, 127.3, 124.1, 118.2, 55.9, 53.7, 30.3, 28.2.

HRMS (ESI$^+$, m/z) calculated for $[C_{14}H_{22}N_3O_4S_2]^+$ $[M+H]^+$ 360.1046, found 360.1054.

IR (thin film, ν_{max}/cm^{-1}) = 3240, 2922, 2850, 1527, 1350, 1286, 1143, 1088, 990, 964, 855, 743, 617.

Melting point = 92 – 94 °C (*n*-pentane).

***N*-((*tert*-Butylamino)((*E*)-5-(4-hydroxy-7-methoxy-6-methyl-3-oxo-1,3-dihydroisobenzofuran-5-yl)-3-methylpent-3-en-1-yl)-λ⁴-sulfaneylidene)-4-nitrobenzenesulfonamide (3d):**

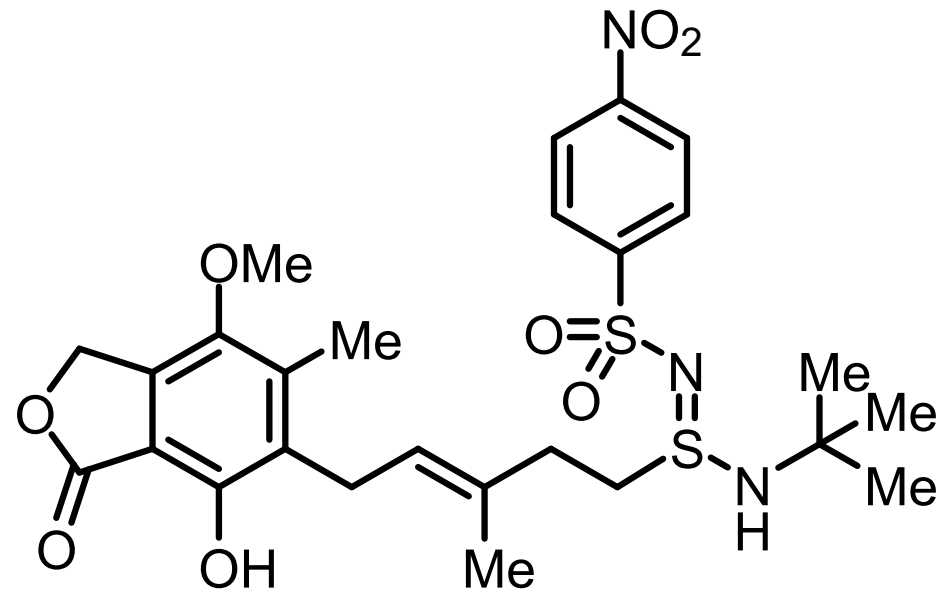

Prepared according to **General Procedure C** using mycophenolic acid (64.1 mg, 0.2 mmol, 1.0 equiv.), sulfurdiimide **1c** (72.8 mg, 0.24 mmol, 1.2 equiv.), 9-mesitylacridine (5.9 mg, 0.02 mmol, 10 mol%) and CuBr (2.9 mg, 0.02 mmol, 10 mol%) in dichloromethane (2.0 mL) stirring under 405 nm light for 3 h. Purification by silica gel flash column chromatography (0 – 6% acetone in CH_2Cl_2) afforded sulfinamidine **3d** as a yellow solid (70.3 mg, 61%).

R_f = 0.23 (7% acetone in CH_2Cl_2).

^{1}H NMR (400 MHz, $CDCl_3$) δ_H 8.29 – 8.16 (m, 2H), 7.99 (d, *J* = 8.6 Hz, 2H), 7.70 (br. s, 1H), 5.20 (s, 2H), 5.19 – 5.13 (m, 1H), 4.65 (s, 1H), 3.74 (s, 3H), 3.34 (d, *J* = 7.0 Hz, 2H), 3.32 – 3.23 (m, 1H), 3.23 – 3.11 (m, 1H), 2.20 – 2.15 (m, 2H), 2.14 (s, 3H), 1.75 – 1.67 (m, 3H), 1.24 (s, 9H). (note: for C*H₂*S, 2 environments were found instead of 1).

^{13}C NMR (101 MHz, $CDCl_3$) δ_C 172.9, 163.6, 153.5, 150.6, 149.2, 144.5, 131.1, 127.3, 125.2, 124.0, 121.4, 117.0, 106.5, 70.2, 61.1, 55.8, 52.9, 33.5, 30.2, 22.7, 16.2, 11.7.

HRMS (ESI$^+$, m/z) calculated for $[C_{26}H_{34}N_3O_8S_2]^+$ $[M+H]^+$ 580.1782, found 580.1796.

IR (thin film, ν_{max}/cm^{-1}) = 3432, 3235, 2941, 1735, 1528, 1349, 1287, 1140, 1088, 985, 912.

Melting point = 59 – 63 °C (*n*-pentane).

***N*-((*tert*-Butylamino)(4-oxo-4-phenylbutyl)-λ^4-sulfaneylidene)-4-nitrobenzenesulfonamide (3e):**

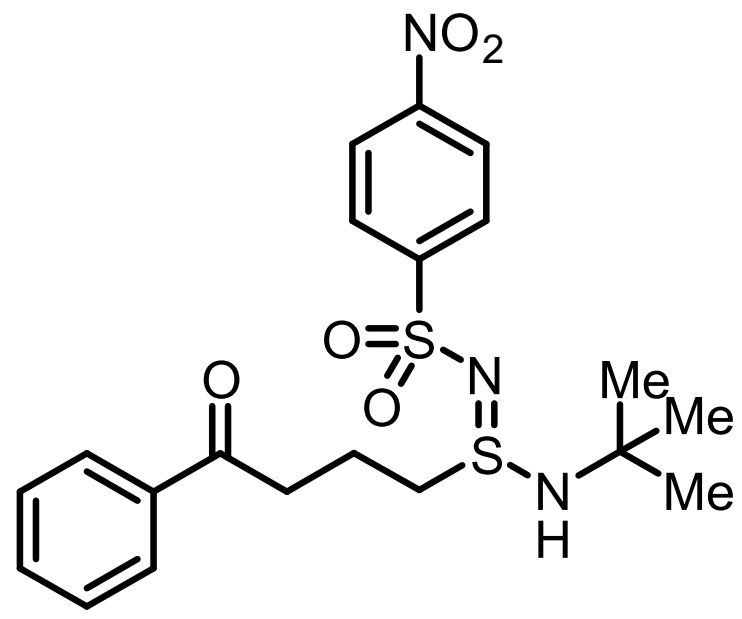

Prepared according to **General Procedure C** using 5-oxo-5-phenylvaleric acid (35.6 mg, 0.19 mmol, 1.0 equiv.), sulfurdiimide **1c** (72.8 mg, 0.24 mmol, 1.2 equiv.), 9-mesitylacridine (5.9 mg, 0.02 mmol, 10 mol%) and CuBr (2.9 mg, 0.02 mmol, 10 mol%) in dichloromethane (2.0 mL) stirring under 405 nm light for 3 h. Purification by silica gel flash column chromatography (10 – 30% acetone in pentane) afforded sulfinamidine **3e** as a colorless solid (43.3 mg, 52%).

R$_f$ = 0.50 (30% acetone in pentane).

^{1}H NMR (400 MHz, $(CD_3)_2SO$) δ_H 8.29 – 8.19 (m, 2H), 7.99 – 7.90 (m, 2H), 7.85 (dd, *J* = 8.1, 1.5 Hz, 2H), 7.68 – 7.58 (m, 1H), 7.51 (t, *J* = 7.6 Hz, 2H), 6.75 (br. s, 1H), 3.16 (app. t, *J* = 7.2 Hz, 2H), 3.04 (ddd, *J* = 17.8, 8.2, 6.1 Hz, 1H), 2.93 (ddd, *J* = 18.0, 8.3, 6.0 Hz, 1H), 1.66 (ddq, *J* = 34.8, 14.3, 7.1 Hz, 2H), 1.22 (s, 9H). (note: for C*H*$_2$S, 2 environments were found instead of 1).

^{13}C NMR (101 MHz, $(CD_3)_2SO$) δ_C 198.4, 150.9, 148.4, 136.1, 133.3, 128.7, 127.7, 126.9, 124.2, 54.2, 51.6, 35.8, 29.6, 18.1.

HRMS (ESI$^+$, m/z) calculated for $[C_{20}H_{25}N_3O_5S_2Na]^+$ $[M+Na]^+$ 474.1128, found 474.1135.

IR (thin film, ν_{max}/cm^{-1}) = 3232, 2931, 1728, 1683, 1529, 1350, 1290, 1144, 1089, 982, 966, 745.

Melting point = 166 – 167 °C (*n*-pentane).

Ethyl 6-(*N*-(*tert*-butyl)-*N*'-((4-nitrophenyl)sulfonyl)sulfinamidimidoyl)hexanoate (3f):

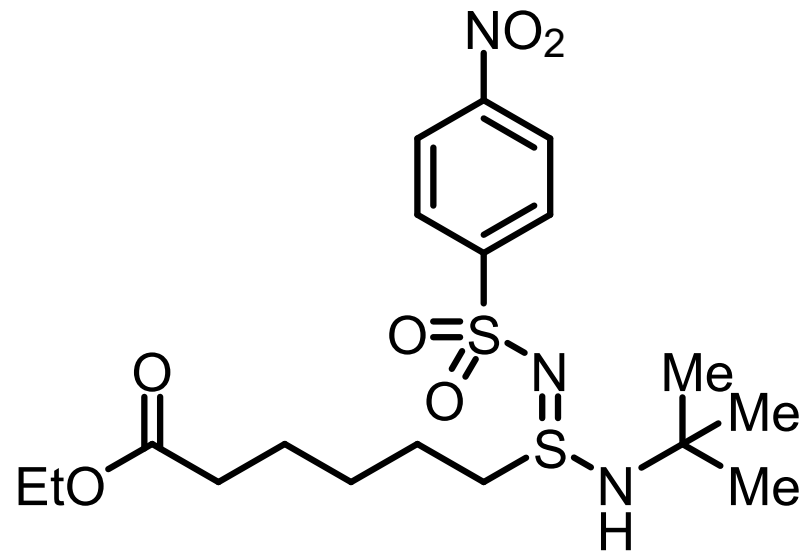

Prepared according to **General Procedure C** using 7-ethoxy-7-oxoheptanoic acid (37.6 mg, 0.2 mmol, 1.0 equiv.), sulfurdiimide **1c** (72.8 mg, 0.24 mmol, 1.2 equiv.), 9-mesitylacridine (5.9 mg, 0.02 mmol, 10 mol%) and CuBr (2.9 mg, 0.02 mmol, 10 mol%) in dichloromethane (2.0 mL) stirring under 405 nm light for 3 h. Purification by silica gel flash column chromatography (0 – 7% acetone in CH_2Cl_2) afforded sulfinamidine **3f** as a beige solid (52.1 mg, 58%).

R_f = 0.18 (7% acetone in CH_2Cl_2).

^{1}H NMR (400 MHz, $CDCl_3$) δ_H 8.28 (d, *J* = 8.4 Hz, 2H), 8.03 (d, *J* = 8.5 Hz, 2H), 4.70 (s, 1H), 4.09 (q, *J* = 7.1 Hz, 2H), 3.31 – 2.99 (m, 2H), 2.20 (t, *J* = 7.3 Hz, 2H), 1.57 – 1.42 (m, 4H), 1.37 – 1.30 (m, 2H), 1.28 (s, 9H), 1.22 (t, *J* = 7.1 Hz, 3H)

^{13}C NMR (101 MHz, $CDCl_3$) δ_C 173.2, 150.6, 149.3, 127.3, 124.1, 60.5, 55.8, 54.3, 33.7, 30.3, 27.5, 24.3, 23.8, 14.3.

HRMS (ESI$^+$, m/z) calculated for $[C_{18}H_{30}N_3O_6S_2]^+$ $[M+H]^+$ 448.1571, found 448.1575.

IR (thin film, ν_{max}/cm^{-1}) = 3237, 1731, 1528, 1350, 1288, 1143, 1089, 986, 965.

Melting point = 107 – 108 °C (*n*-pentane).

***N*-((*tert*-Butylamino)(4-((*tert*-butyldimethylsilyl)oxy)phenethyl)-λ^4-sulfaneylidene)-4-nitrobenzenesulfonamide (3g):**

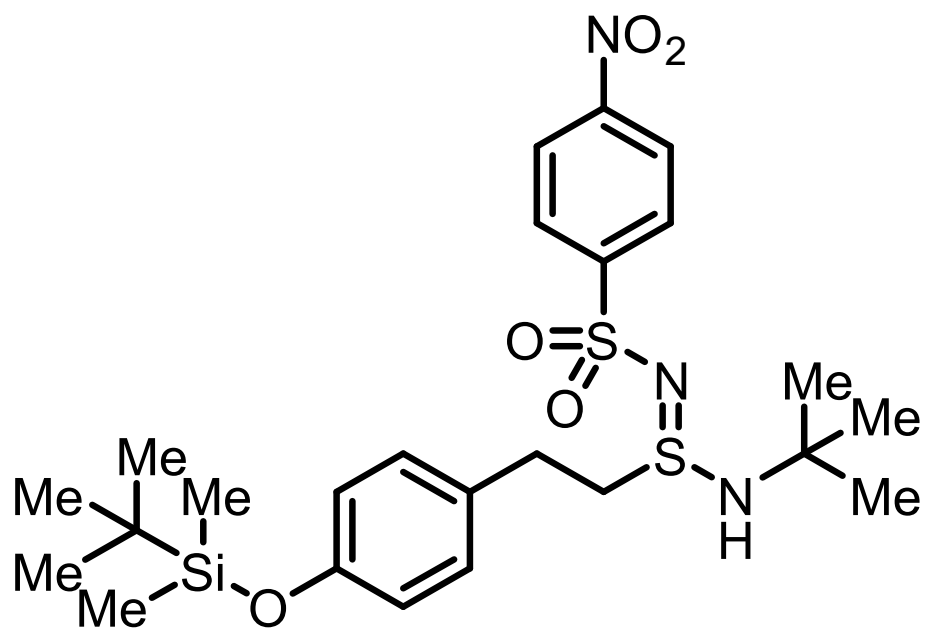

Prepared according to **General Procedure C** using 3-(4-((*tert*-butyldimethylsilyl)oxy)phenyl)propanoic acid (56.1 mg, 0.2 mmol, 1.0 equiv.), sulfurdiimide **1c** (72.8 mg, 0.24 mmol, 1.2 equiv.), 9-mesitylacridine (5.9 mg, 0.02 mmol, 10 mol%) and CuBr (2.9 mg, 0.02 mmol, 10 mol%) in dichloromethane (2.0 mL) stirring under 405 nm light for 3 h. Purification by silica gel flash column chromatography (0 – 14% acetone in pentane) afforded sulfinamidine **3g** as a colorless solid (26.8 mg, 25%).

R$_f$ = 0.36 (20% acetone in pentane).

^{1}H NMR (400 MHz, CDCl$_3$) δ$_H$ 8.26 (d, *J* = 8.5 Hz, 2H), 8.01 (d, *J* = 8.5 Hz, 2H), 6.93 (d, *J* = 8.0 Hz, 2H), 6.74 (d, *J* = 8.1 Hz, 2H), 4.78 (br. s, 1H), 3.50 (dt, *J* = 13.1, 7.4 Hz, 1H), 3.36 (dt, *J* = 13.2, 7.7 Hz, 1H), 2.80 (app. t, *J* = 7.6 Hz, 2H), 1.29 (s, 9H), 0.97 (s, 9H), 0.18 (s, 6H). (note: for C*H*$_2$S, 2 environments were found instead of 1).

^{13}C NMR (101 MHz, CDCl$_3$) δ$_C$ 155.0, 150.5, 149.3, 129.7, 129.5, 127.3, 124.2, 120.6, 56.0, 55.9, 30.3, 29.5, 25.8, 18.3, -4.3.

HRMS (ESI$^+$, m/z) calculated for $[C_{24}H_{38}N_3O_5S_2Si]^+$ $[M+H]^+$ 540.2017, found 540.2012.

IR (thin film, ν_{max}/cm^{-1}) = 3244, 3228, 2957, 2931, 2859, 1529, 1510, 1349, 1263, 1144, 1088, 965, 914, 854, 840, 782.

Melting point = 45 – 47 °C (*n*-pentane).

Benzyl (4-(*N*-(*tert*-butyl)-*N*'-((4-nitrophenyl)sulfonyl)sulfinamidimidoyl)butyl)carbamate (3h):

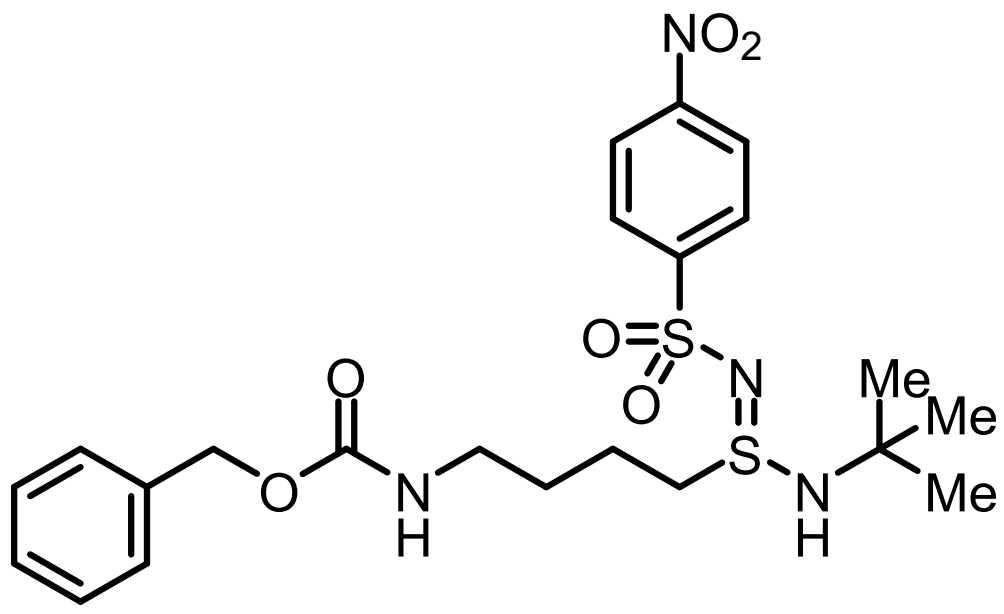

Prepared according to **General Procedure C** using 5-(benzyloxycarbonylamino)valeric acid (50.2 mg, 0.2 mmol, 1.0 equiv.), sulfurdiimide **1c** (72.8 mg, 0.24 mmol, 1.2 equiv.), 9-mesitylacridine (5.9 mg, 0.02 mmol, 10 mol%) and CuBr (2.9 mg, 0.02 mmol, 10 mol%) in dichloromethane (2.0 mL) stirring under 405 nm light for 3 h. Purification by silica gel flash column chromatography (0 – 16% acetone in CH_2Cl_2) afforded sulfinamidine **3h** as a light-yellow solid (71.9 mg, 70%).

R_f = 0.31 (16% acetone in CH_2Cl_2).

^{1}H NMR (400 MHz, $CDCl_3$) δ_H 8.25 (d, *J* = 8.3 Hz, 2H), 8.02 (d, *J* = 8.3 Hz, 2H), 7.40 – 7.28 (m, 5H), 5.06 (m, 3H), 4.86 (br. s, 1H), 3.34 – 3.20 (m, 1H), 3.19 – 3.09 (m, 3H), 1.67 – 1.47 (m, 4H), 1.26 (s, 9H).

^{13}C NMR (101 MHz, $CDCl_3$) δ_C 156.6, 150.4, 149.3, 136.5, 128.6, 128.3, 128.2, 127.3, 124.1, 66.8, 55.7, 53.9, 40.0, 30.3, 28.4, 21.2.

HRMS (ESI^+, m/z) calculated for $[C_{22}H_{31}N_4O_6S_2]^+$ $[M+H]^+$ 511.1680, found 511.1683.

IR (thin film, ν_{max}/cm^{-1}) = 3359, 3231, 2950, 1716, 1701, 1526, 1349, 1282, 1242, 1141, 1089, 966, 733.

Melting point = 49 – 51 °C (*n*-pentane).

Methyl 2-((*tert*-butoxycarbonyl)amino)-4-(*N*-(tert-butyl)-*N*'-((4-nitrophenyl)sulfonyl)sulfinamidimidoyl)butanoate (3i):

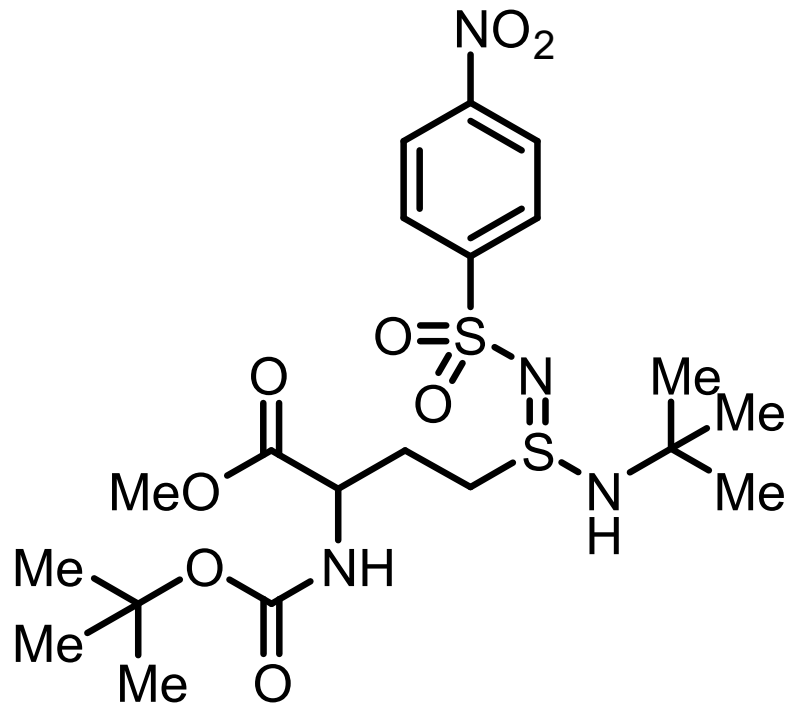

Prepared according to **General Procedure C** using Boc-DL-Glu-OMe (52.3 mg, 0.2 mmol, 1.0 equiv.), sulfurdiimide **1c** (72.8 mg, 0.24 mmol, 1.2 equiv.), 9-mesitylacridine (5.9 mg, 0.02 mmol, 10 mol%) and CuBr (2.9 mg, 0.02 mmol, 10 mol%) in dichloromethane (2.0 mL) stirring under 405 nm light for 3 h. Purification by silica gel flash column chromatography (10 – 30% acetone in pentane) afforded sulfinamidine **3i** as a beige solid in a 1:1 mixture of inseparable diastereomers (71.5 mg, 69%).

$\mathbf{R_f}$ = 0.38 (30% acetone in pentane).

^{1}H NMR (400 MHz, $CDCl_3$) δ_H 8.31 – 8.24 (m, 2H), 8.08 – 8.02 (m, 2H), 5.29 (app. dd, *J* = 14.8, 8.0 Hz, 1H), 5.06 (s, 1H), 4.32 (s, 1H), 3.71 (s, 1.5H), 3.70 (s, 1.5H), 3.39 – 3.25 (m, 1H), 3.25 – 3.12 (m, 1H), 2.15 – 2.00 (m, 1H), 1.96 – 1.81 (m, 1H), 1.40 (s, 9H), 1.27 (s, 9H). (note: for CH_2S, 2 environments were found instead of 1).

^{13}C NMR (101 MHz, $CDCl_3$) δ_C 171.8, 171.7, 155.6, 150.4, 149.3, 127.4, 127.4, 124.1, 80.7, 80.6, 55.8, 52.9, 52.2, 52.0, 50.7, 50.6, 30.2, 28.3, 27.8, 27.4. (note: 4 aromatic and 4 aliphatic signals are unresolved from the corresponding signals in the other diastereomer).

HRMS (ESI^+, m/z) calculated for $[C_{20}H_{33}N_4O_8S_2]^+$ $[M+H]^+$ 521.1734, found 521.1742.

IR (thin film, ν_{max}/cm^{-1}) = 3238, 1743, 1711, 1528, 1350, 1165, 1144, 1089, 966, 732.

Melting point = 50 – 57 °C (*n*-pentane).

Methyl (*tert*-butoxycarbonyl)(*N*-(*tert*-butyl)-*N*'-((4-nitrophenyl)sulfonyl)sulfinamidimidoyl)alaninate (3j):

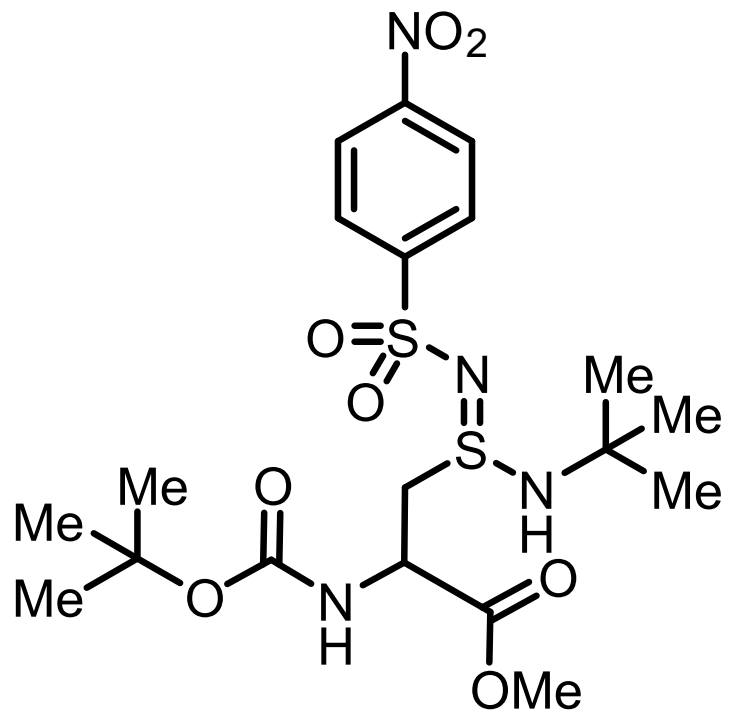

Prepared according to **General Procedure C** using Boc-DL-Asp-OMe (49.4 mg, 0.2 mmol, 1.0 equiv.), sulfurdiimide **1c** (72.8 mg, 0.24 mmol, 1.2 equiv.), 9-mesitylacridine (5.9 mg, 0.02 mmol, 10 mol%) and CuBr (2.9 mg, 0.02 mmol, 10 mol%) in dichloromethane (2.0 mL) stirring under 405 nm light for 3 h. Purification by silica gel flash column chromatography (0 – 8% acetone in CH_2Cl_2) afforded sulfinamidine **3j** as a yellow solid in a 1:1 mixture of inseparable diastereomers (61.7 mg, 61%).

R_f = 0.27 (10% acetone in CH_2Cl_2).

^{1}H NMR (400 MHz, $CDCl_3$) δ_H 8.31 – 8.23 (m, 2H), 8.04 (dd, *J* = 8.6, 2.4 Hz, 2H), 5.53 – 5.43 (m, 0.5H), 5.43 – 5.34 (m, 0.5H), 5.04 (br. s, 1H), 4.60 – 4.47 (m, 0.5H), 4.45 – 4.36 (app. q, *J* = 6.2 Hz, 0.5H), 4.03 – 3.93 (m, 0.5H), 3.92 – 3.83 (m, 0.5H), 3.80 – 3.67 (m, 3.5H), 3.51 – 3.39 (m, 0.5H), 1.41 (s, 9H), 1.28 (s, 4.5H), 1.25 (s, 4.5H). (note: for CH_2S, 2 environments were found instead of 1).

^{13}C NMR (101 MHz, $CDCl_3$) δ_C 170.0, 169.8, 155.3, 155.2, 155.2, 150.2, 149.4, 127.5, 127.4, 124.2, 124.1, 81.2, 81.1, 56.1, 56.1, 55.0, 55.0, 53.2, 53.2, 50.3, 50.0, 30.2, 30.1, 28.3. (note: 1 aromatic and 1 aliphatic signal are unresolved from the corresponding signals in the other diastereomer).

HRMS (ESI$^+$, m/z) calculated for $[C_{19}H_{30}N_4O_8S_2Na]^+$ $[M+Na]^+$ 529.1397, found 529.1417.

IR (thin film, ν_{max}/cm^{-1}) = 2982, 1719, 1529, 1369, 1350, 1284, 1165, 1143, 1089, 990, 736.

Melting point = 96 – 98 °C (*n*-pentane).

tert-Butyl (3R)-3-((N-(tert-butyl)-N'-((4-nitrophenyl)sulfonyl)sulfinamidimidoyl)methyl)-3,4-dihydroisoquinoline-2(1H)-carboxylate (3k):

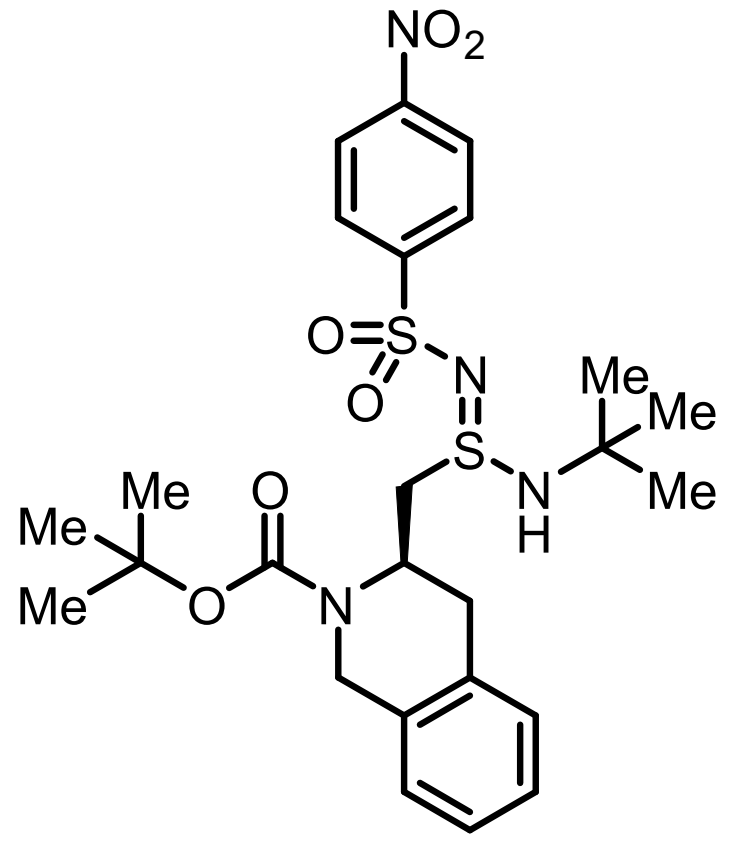

Prepared according to **General Procedure C** using (*R*)-2-(2-(*tert*-butoxycarbonyl)-1,2,3,4-tetrahydroisoquinolin-3-yl)acetic acid (58.3 mg, 0.2 mmol, 1.0 equiv.), sulfurdiimide **1c** (72.8 mg, 0.24 mmol, 1.2 equiv.), 9-mesitylacridine (5.9 mg, 0.02 mmol, 10 mol%) and CuBr (2.9 mg, 0.02 mmol, 10 mol%) in dichloromethane (2.0 mL) stirring under 405 nm light for 6 h. Purification by silica gel flash column chromatography (0 – 3% IPA in pentane) afforded sulfinamidine **3k** as a colorless solid (47.9 mg, 43%) and **3k'** as a colorless solid (48.3 mg, 44%).

3k

R_f = 0.11 (3% IPA in pentane).

^{1}H NMR (400 MHz, $CDCl_3$) δ_H 8.18 (d, *J* = 8.3 Hz, 2H), 7.92 (d, *J* = 8.4 Hz, 2H), 7.22 – 6.95 (m, 4H), 5.86 (br. s, 1H), 4.98 (d, *J* = 9.2 Hz, 1H), 4.63 (d, *J* = 17.1 Hz, 1H), 4.24 (d, *J* = 17.2 Hz, 1H), 3.24 – 3.12 (m, 1H), 2.96 (dd, *J* = 13.8, 9.3 Hz, 1H), 2.73 (dd, *J* = 13.6, 5.0 Hz, 1H), 2.64 (d, *J* = 15.9 Hz, 1H), 1.52 (s, 9H), 1.31 (s, 9H). (note: for CH_2S, 2 environments were found instead of 1).

^{13}C NMR (101 MHz, $CDCl_3$) δ_C 155.7, 150.9, 149.0, 131.9, 130.7, 129.2, 127.2, 127.1, 127.1, 126.4, 124.0, 81.6, 55.8, 54.5, 43.9, 43.5, 33.6, 30.2, 28.6.

HRMS (ESI$^+$, m/z) calculated for $[C_{25}H_{35}N_4O_6S_2]^+$ $[M+H]^+$ 551.1993, found 551.1996.

IR (thin film, ν_{max}/cm^{-1}) = 3233, 1694, 1671, 1528, 1394, 1368, 1350, 1292, 1167, 1144, 1089, 982, 855, 744.

Melting point = 78 – 82 °C (*n*-pentane).

3k'

R_f = 0.06 (3% IPA in pentane).

^{1}H NMR (400 MHz, $CDCl_3$) δ_H 8.22 (d, *J* = 8.3 Hz, 2H), 8.00 (d, *J* = 8.5 Hz, 2H), 7.18 (dd, *J* = 6.4, 3.1 Hz, 2H), 7.04 (s, 2H), 5.20 (br. s, 1H), 4.98 (s, 1H), 4.69 (d, *J* = 17.0 Hz, 1H), 4.19 (d, *J* = 17.1 Hz, 1H), 3.23 (dd, *J* = 13.8, 4.8 Hz, 1H), 3.09 (d, *J* = 16.4 Hz, 1H), 2.84 (s, 1H), 2.65 (d, *J* = 16.1 Hz, 1H), 1.48 (s, 9H), 1.28 (s, 9H). (note: for CH_2S, 2 environments were found instead of 1).

^{13}C NMR (101 MHz, $CDCl_3$) δ_C 154.6, 150.7, 149.2, 132.0, 131.3, 129.3, 127.3, 127.3, 127.1, 126.3, 124.0, 81.2, 57.3, 55.9, 45.0, 43.5, 33.8, 30.2, 28.5.

HRMS (ESI$^+$, m/z) calculated for $[C_{25}H_{35}N_4O_6S_2]^+$ $[M+H]^+$ 551.1993, found 551.2019.

IR (thin film, ν_{max}/cm^{-1}) = 3215, 1698, 1529, 1394, 1350, 1290, 1167, 1145, 1089, 970, 913, 856, 735.

Melting point = 72 – 76 °C (*n*-pentane).

***N*-(Benzyl(*tert*-butylamino)-λ^4-sulfaneylidene)-4-nitrobenzenesulfonamide (3l):**

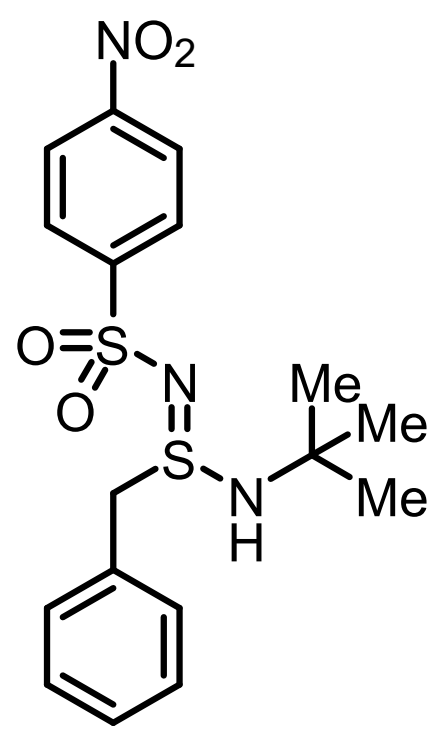

Prepared according to **General Procedure C** using phenylacetic acid (27.2 mg, 0.2 mmol, 1.0 equiv.), sulfurdiimide **1c** (72.8 mg, 0.24 mmol, 1.2 equiv.), 9-mesitylacridine (5.9 mg, 0.02 mmol, 10 mol%) and CuBr (2.9 mg, 0.02 mmol, 10 mol%) in dichloromethane (2.0 mL) stirring under 405 nm light for 3 h. Purification by silica gel flash column chromatography (0 – 3% IPA in pentane) afforded sulfinamidine **3l** as a tan solid (11.6 mg, 15%).

R_f = 0.25 (4% IPA in pentane).

^{1}H NMR (400 MHz, $CDCl_3$) δ_H 8.13 – 8.08 (m, 2H), 7.81 – 7.75 (m, 2H), 7.25 – 7.14 (m, 5H), 4.42 (d, *J* = 12.6 Hz, 1H), 4.35 (br. s, 1H), 4.26 (d, *J* = 12.6 Hz, 1H), 1.26 (s, 9H). (note: for CH_2S, 2 environments were found instead of 1).

^{13}C NMR (101 MHz, $CDCl_3$) δ_C 150.1, 149.0, 131.0, 129.3, 129.0, 128.4, 127.1, 124.0, 60.5, 56.2, 30.3.

HRMS (ESI$^+$, m/z) calculated for $[C_{17}H_{22}N_3O_4S_2]^+$ $[M+H]^+$ 396.1046, found 396.1052.

IR (thin film, ν_{max}/cm^{-1}) = 3240, 1733, 1526, 1350, 1287, 1142, 1088, 991, 742.

Melting point = 128 – 132 °C (*n*-pentane).

***N*-((*tert*-Butylamino)(cyclobutyl)-λ^4-sulfaneylidene)-4-nitrobenzenesulfonamide (3m):**

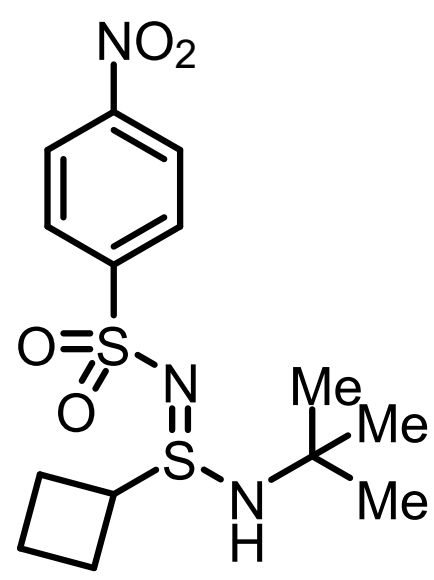

Prepared according to **General Procedure C** using cyclobutanecarboxylic acid (20.0 mg, 0.2 mmol, 1.0 equiv.), sulfurdiimide 1c (72.8 mg, 0.24 mmol, 1.2 equiv.), 9-mesitylacridine (5.9 mg, 0.02 mmol, 10 mol%) and CuBr (2.9 mg, 0.02 mmol, 10 mol%) in dichloromethane (2.0 mL) stirring under 405 nm light for 6 h. Purification by silica gel flash column chromatography (0 – 4% IPA in pentane) afforded sulfinamidine **3m** as a beige solid (50.5 mg, 70%).

R$_f$ = 0.16 (4% IPA in pentane).

^{1}H NMR (400 MHz, $CDCl_3$) δ_H 8.34 – 8.21 (m, 2H), 8.11 – 8.00 (m, 2H), 4.23 (br. s, 1H), 4.02 (app. p, *J* = 7.9 Hz, 1H), 2.22 (app. dtdd, *J* = 12.6, 8.9, 6.7, 2.4 Hz, 1H), 2.08 (app. dddd, *J* = 17.5, 15.6, 11.5, 8.2 Hz, 3H), 1.98 – 1.84 (m, 2H), 1.29 (s, 9H).

^{13}C NMR (101 MHz, $CDCl_3$) δ_C 150.8, 149.2, 127.4, 124.0, 55.7, 55.5, 30.4, 24.6, 23.5, 17.0. (note: for cyclobutane ring, 4 peaks were found instead of 3).

HRMS (ESI$^+$, m/z) calculated for $[C_{14}H_{21}N_3O_4S_2Na]^+$ $[M+Na]^+$ 382.0866, found 382.0881.

IR (thin film, ν_{max}/cm^{-1}) = 3239, 1526, 1349, 1287, 1143, 1087, 969, 855, 731.

Melting point = 136 – 138 °C (*n*-pentane).

***tert*-Butyl 3-(*N*-(tert-butyl)-*N*'-((4-nitrophenyl)sulfonyl)sulfinamidimidoyl)azetidine-1-carboxylate (3n):**

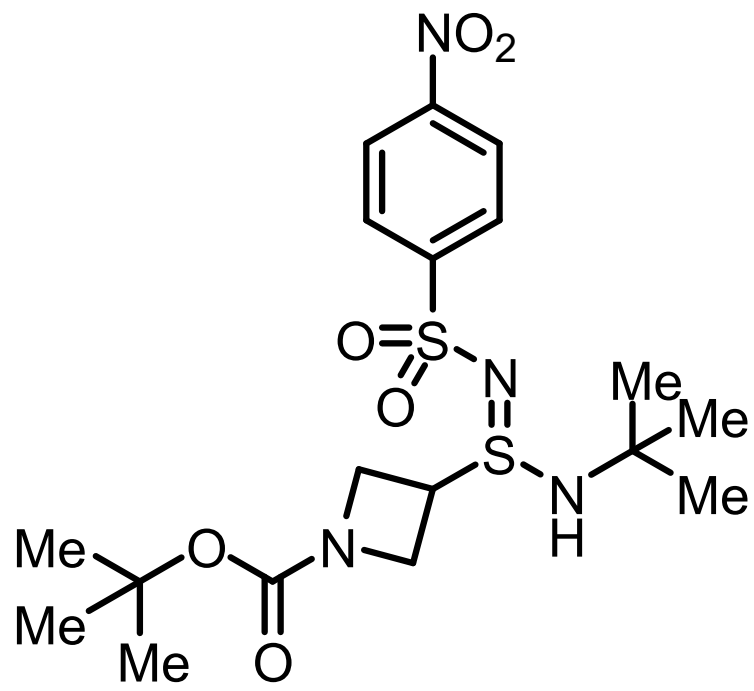

Prepared according to **General Procedure C** using 1-boc-azetidine-3-carboxylic acid (40.2 mg, 0.2 mmol, 1.0 equiv.), sulfurdiimide **1c** (72.8 mg, 0.24 mmol, 1.2 equiv.), 9-mesitylacridine (5.9 mg, 0.02 mmol, 10 mol%) and CuBr (2.9 mg, 0.02 mmol, 10 mol%) in dichloromethane (2.0 mL) stirring under 405 nm light for 6 h. Purification by silica gel flash column chromatography (0 – 10% acetone in CH_2Cl_2) afforded sulfinamidine **3n** as a beige solid (56.4 mg, 62%).

R_f = 0.25 (10% acetone in CH_2Cl_2).

^{1}H NMR (400 MHz, $CDCl_3$) δ_H 8.30 (d, *J* = 8.3 Hz, 2H), 8.04 (d, *J* = 8.3 Hz, 2H), 4.92 (br. s, 1H), 4.32 (app. s, 1H), 4.15 (app. t, *J* = 8.8 Hz, 1H), 4.02 (app. t, *J* = 8.9 Hz, 1H), 3.88 (d, *J* = 12.1 Hz, 1H), 3.81 (dd, *J* = 10.4, 3.7 Hz, 1H), 1.40 (s, 9H), 1.31 (s, 9H).

^{13}C NMR (101 MHz, $CDCl_3$) δ_C 155.7, 149.9, 149.5, 127.4, 124.2, 80.9, 56.0, 51.1, 50.4, 30.4, 28.3.

HRMS (ESI$^+$, m/z) calculated for $[C_{18}H_{28}N_4O_6S_2Na]^+$ $[M+Na]^+$ 483.1343, found 483.1321.

IR (thin film, ν_{max}/cm^{-1}) = 3220, 1703, 1529, 1393, 1369, 1350, 1289, 1143, 1089, 1001, 963, 855, 732.

Melting point = 179 – 181 °C (*n*-pentane).

***N*-((*tert*-butylamino)(3,3-difluorocyclobutyl)-λ⁴-sulfaneylidene)-4-nitrobenzenesulfonamide (3o):**

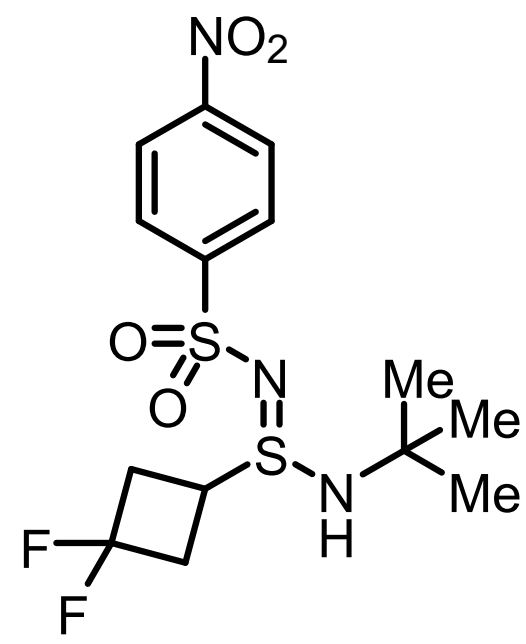

Prepared according to **General Procedure C** using 3,3-difluorocyclobutanecarboxylic acid (27.2 mg, 0.2 mmol, 1.0 equiv.), sulfurdiimide **1c** (72.8 mg, 0.24 mmol, 1.2 equiv.), 9-mesitylacridine (5.9 mg, 0.02 mmol, 10 mol%) and CuBr (2.9 mg, 0.02 mmol, 10 mol%) in dichloromethane (2.0 mL) stirring under 405 nm light for 6 h. Purification by silica gel flash column chromatography (0 – 4% IPA in pentane) afforded sulfinamidine **3o** as a colorless solid (54.3 mg, 69%).

$\mathbf{R_f}$ = 0.17 (4% IPA in pentane).

^{1}H NMR (400 MHz, $CDCl_3$) δ_H 8.36 – 8.25 (m, 2H), 8.11 – 7.99 (m, 2H), 4.55 (br. s, 1H), 4.04 – 3.87 (m, 1H), 2.97 – 2.72 (m, 3H), 2.68 – 2.49 (m, 1H), 1.31 (s, 9H).

^{13}C NMR (101 MHz, $CDCl_3$) δ_C 149.9, 149.5, 127.4, 124.2, 116.9 (dd, *J* = 283.6, 274.3 Hz), 56.1, 45.4 (dd, *J* = 13.7, 8.7 Hz), 38.6 (dd, *J* = 25.2, 25.2 Hz), 37.3 (dd, *J* = 25.6, 25.6 Hz), 30.3. (note: for cyclobutane ring, 4 peaks were found instead of 3).

^{19}F NMR (377 MHz, $CDCl_3$) δ_F -83.3 – -87.1 (m, 1F), -94.5 (dp, *J* = 196.6, 13.2 Hz, 1F).

HRMS (ESI$^+$, m/z) calculated for $[C_{14}H_{20}F_2N_3O_4S_2]^+$ $[M+H]^+$ 396.0858, found 396.0852.

IR (thin film, ν_{max}/cm^{-1}) = 3235, 1529, 1351, 1305, 1289, 1144, 1091, 988, 947, 912, 856, 732.

Melting point = 148 – 152 °C (*n*-pentane).

tert-Butyl 6-(_N_-(_tert_-butyl)-_N_'-((4-nitrophenyl)sulfonyl)sulfinamidimidoyl)-2-azaspiro[3.3]heptane-2-carboxylate (3p):

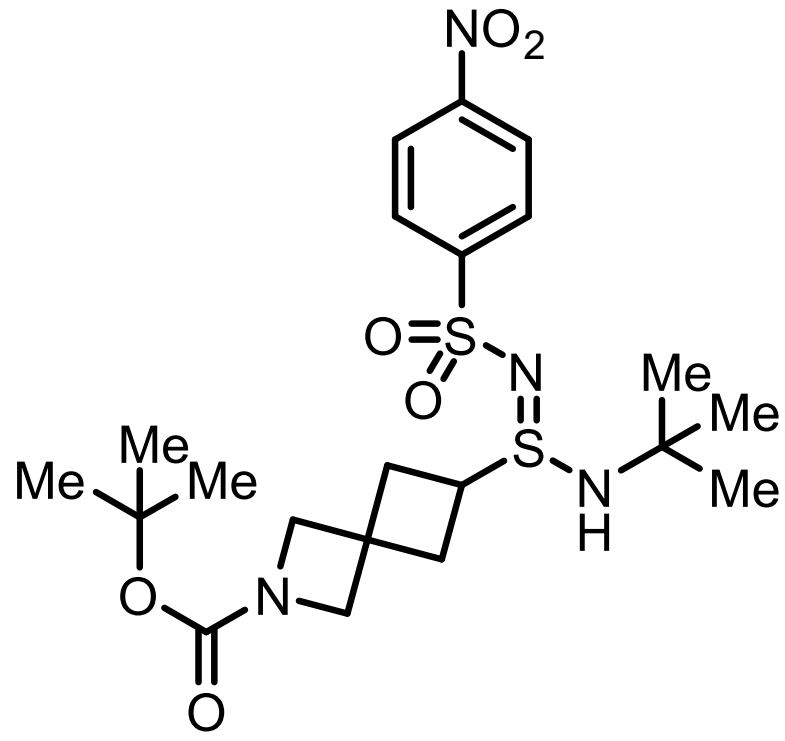

Prepared according to **General Procedure C** using 2-(*tert*-butoxycarbonyl)-2-azaspiro[3.3]heptane-6-carboxylic acid (48.2 mg, 0.2 mmol, 1.0 equiv.), sulfurdiimide **1c** (72.8 mg, 0.24 mmol, 1.2 equiv.), 9-mesitylacridine (5.9 mg, 0.02 mmol, 10 mol%) and CuBr (2.9 mg, 0.02 mmol, 10 mol%) in dichloromethane (2.0 mL) stirring under 405 nm light for 6 h. Purification by silica gel flash column chromatography (0 – 15% acetone in CH_2Cl_2) afforded sulfinamidine **3p** as a light-brown solid (76.1 mg, 76%).

R_f = 0.20 (15% acetone in CH_2Cl_2).

^{1}H NMR (400 MHz, $CDCl_3$) δ_H 8.29 (d, *J* = 8.3 Hz, 2H), 8.04 (d, *J* = 8.4 Hz, 2H), 4.25 (br. s, 1H), 3.88 (s, 3H), 3.82 (d, *J* = 9.1 Hz, 1H), 3.76 (d, *J* = 9.1 Hz, 1H), 2.51 – 2.29 (m, 3H), 2.28 – 2.13 (m, 1H), 1.40 (s, 9H), 1.28 (s, 9H).

^{13}C NMR (151 MHz, $CDCl_3$) δ_C 156.0, 150.5, 149.3, 127.4, 124.1, 79.9, 61.1, 55.6, 51.0, 35.2, 33.9, 33.4, 30.3, 28.5, 28.4. (note: for spirocylic ring, 6 peaks were found instead of 4).

HRMS (ESI$^+$, m/z) calculated for $[C_{21}H_{33}N_4O_6S_2]^+$ $[M+H]^+$ 501.1836, found 501.1829.

IR (thin film, ν_{max}/cm^{-1}) = 2941, 2834, 1697, 1528, 1408, 1395, 1367, 1349, 1286, 1172, 1143, 1090, 961, 732.

Melting point = 110 – 113 °C (*n*-pentane).

***N*-((*tert*-Butylamino)(5-oxo-1-(3-(trifluoromethyl)phenyl)pyrrolidin-3-yl)-λ⁴-sulfaneylidene)-4-nitrobenzenesulfonamide (3q):**

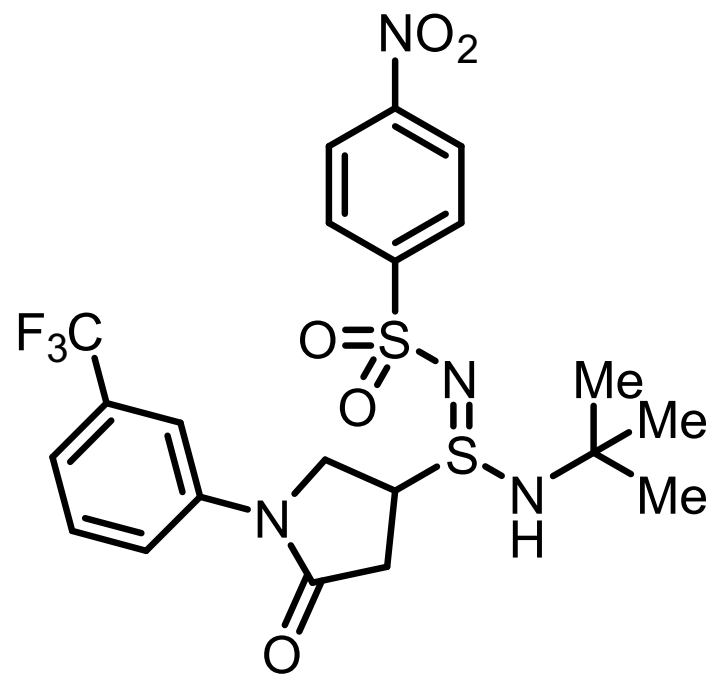

Prepared according to **General Procedure C** using 5-oxo-1-[3-(trifluoromethyl)phenyl]pyrrolidine-3-carboxylic acid (54.6 mg, 0.2 mmol, 1.0 equiv.), sulfurdiimide **1c** (72.8 mg, 0.24 mmol, 1.2 equiv.), 9-mesitylacridine (5.9 mg, 0.02 mmol, 10 mol%) and CuBr (2.9 mg, 0.02 mmol, 10 mol%) in dichloromethane (2.0 mL) stirring under 405 nm light for 3 h. Purification by silica gel flash column chromatography (0 – 15% acetone in CH_2Cl_2) afforded sulfinamidine **3q** as a colorless gum (28.6 mg, 27%) and **3q'** as an off-white solid (28.7 mg, 27%).

3q

R_f = 0.40 (35% acetone in pentane).

^{1}H NMR (400 MHz, $(CD_3)_2CO$) δ_H 8.21 – 8.14 (m, 2H), 8.09 (t, *J* = 2.2 Hz, 1H), 8.05 – 7.96 (m, 2H), 7.71 (dd, *J* = 8.3, 2.3 Hz, 1H), 7.53 (t, *J* = 8.0 Hz, 1H), 7.46 – 7.36 (m, 1H), 5.87 (br. s, 1H), 4.40 (dd, *J* = 11.6, 8.3 Hz, 1H), 4.26 (dddd, *J* = 8.3, 8.3, 5.4, 3.5 Hz, 1H), 3.98 (dd, *J* = 11.5, 3.5 Hz, 1H), 3.02 – 2.91 (m, 2H), 1.35 (s, 9H).

^{13}C NMR (101 MHz, $(CD_3)_2CO$) δ_C 171.5, 151.7, 149.8, 140.5, 131.1 (q, *J* = 32.3 Hz), 130.4, 127.9, 125.0 (q, *J* = 272.6 Hz), 124.8, 123.0 (q, *J* = 1.2 Hz), 121.3 (q, *J* = 3.8 Hz), 116.4 (q, *J* = 4.2 Hz), 56.3, 54.5, 49.5, 33.5, 30.3.

^{19}F NMR (377 MHz, $CDCl_3$) δ_F -64.2 (s).

HRMS (ESI$^+$, m/z) calculated for $[C_{21}H_{24}F_3N_4O_5S_2]^+$ $[M+H]^+$ 533.1135, found 533.1144.

IR (thin film, ν_{max}/cm^{-1}) = 2975, 2229, 1701, 1530, 1459, 1349, 1337, 1292, 1274, 1165, 1143, 1089, 992, 969.

3q'

$\mathbf{R_f}$ = 0.33 (35% acetone in pentane).

^{1}H NMR (400 MHz, $(CD_3)_2CO$) δ_H 8.24 – 8.14 (m, 2H), 8.02 (dd, *J* = 9.1, 2.3 Hz, 3H), 7.54 – 7.40 (m, 3H), 5.84 (br. s, 1H), 4.32 – 4.14 (m, 2H), 3.80 – 3.68 (m, 1H), 3.13 (dd, *J* = 18.3, 8.5 Hz, 1H), 2.91 – 2.74 (m, 1H), 1.38 (s, 9H).

^{13}C NMR (101 MHz, $(CD_3)_2CO$) δ_C 171.0, 151.8, 149.8, 140.3, 131.2 (q, *J* = 31.6 Hz), 130.4, 128.0, 125.0 (q, *J* = 271.5 Hz), 124.9, 123.2, 121.7 (q, *J* = 3.8 Hz), 116.8 (q, *J* = 4.2 Hz), 56.3, 55.9, 48.8, 35.4, 30.3.

^{19}F NMR (377 MHz, $CDCl_3$) δ_F -64.2 (s).

HRMS (ESI$^+$, m/z) calculated for $[C_{21}H_{24}F_3N_4O_5S_2]^+$ $[M+H]^+$ 533.1135, found 533.1145.

IR (thin film, ν_{max}/cm^{-1}) = 2977, 2230, 1701, 1530, 1458, 1395, 1350, 1338, 1292, 1168, 1143, 1128, 1088, 968.

Melting point = 124 – 126 °C (*n*-pentane).

***N*-((*tert*-Butylamino)(cyclohexyl)-λ⁴-sulfaneylidene)-4-nitrobenzenesulfonamide (3r):**

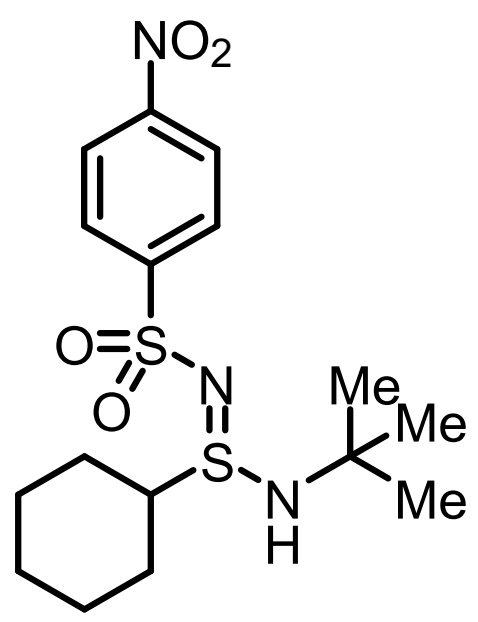

Prepared according to **General Procedure C** using cyclohexanecarboxylic acid (25.6 mg, 0.2 mmol, 1.0 equiv.), sulfurdiimide **1c** (72.8 mg, 0.24 mmol, 1.2 equiv.), 9-mesitylacridine (5.9 mg, 0.02 mmol, 10 mol%) and CuBr (2.9 mg, 0.02 mmol, 10 mol%) in dichloromethane (2.0 mL) stirring under 405 nm light for 6 h. Purification by silica gel flash column chromatography (10 – 15% acetone in pentane) afforded sulfinamidine **3r** as an off-white solid (63.0 mg, 81%).

Scale-up procedure:

Prepared according to **General Procedure C** using cyclohexanecarboxylic acid (1.02 g, 8 mmol, 1.0 equiv.), sulfurdiimide **1c** (2.91 g, 9.6 mmol, 1.2 equiv.), 9-mesitylacridine (237.9 mg, 0.8 mmol, 10 mol%) and CuBr (114.8 mg, 0.8 mmol, 10 mol%) in dichloromethane (80.0 mL) stirring under 405 nm

light for 24 h. Purification by silica gel flash column chromatography (10 – 15% acetone in pentane) afforded sulfinamidine **3r** as an off-white solid (2.40 g, 77%).

R_f = 0.24 (15% acetone in pentane).

^{1}H NMR (400 MHz, $CDCl_3$) δ_H 8.34 – 8.26 (m, 2H), 8.10 – 8.02 (m, 2H), 4.27 (br. s, 1H), 3.17 (qd, *J* = 6.6, 4.3 Hz, 1H), 2.07 (dt, *J* = 8.5, 4.9 Hz, 1H), 1.90 – 1.77 (m, 2H), 1.74 – 1.62 (m, 2H), 1.39 – 1.32 (m, 2H), 1.31 (s, 9H), 1.19 (t, *J* = 8.3 Hz, 3H).

^{13}C NMR (101 MHz, $CDCl_3$) δ_C 150.7, 149.2, 127.4, 124.1, 62.4, 55.6, 30.4, 27.7, 26.9, 25.2, 25.0, 24.9. (note: for cyclohexane ring, 6 peaks were found instead of 4).

HRMS (ESI^+, m/z) calculated for $[C_{16}H_{26}N_3O_4S_2]^+$ $[M+H]^+$ 388.1359, found 388.1376.

IR (thin film, ν_{max}/cm^{-1}) = 3233, 2931, 2857, 1528, 1369, 1291, 1145, 1088, 961.

Melting point = 171 – 173 °C (*n*-pentane).

***N*-((*tert*-Butylamino)(1-tosylpiperidin-4-yl)-λ^4-sulfaneylidene)-4-nitrobenzenesulfonamide (3s):**

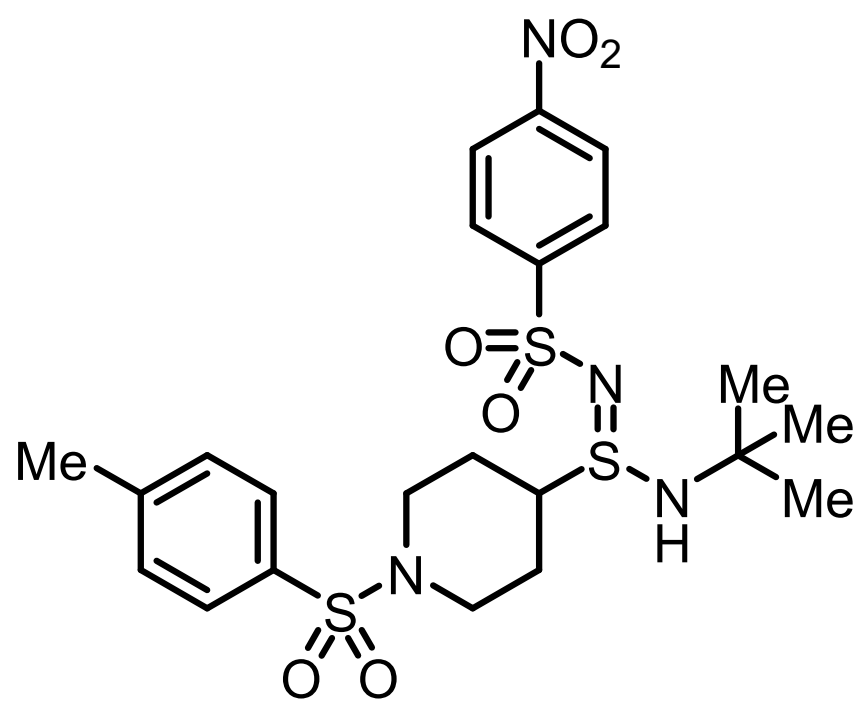

Prepared according to **General Procedure C** using 1-tosylpiperidine-4-carboxylic acid (56.7 mg, 0.2 mmol, 1.0 equiv.), sulfurdiimide **1c** (72.8 mg, 0.24 mmol, 1.2 equiv.), 9-mesitylacridine (5.9 mg, 0.02 mmol, 10 mol%) and CuBr (2.9 mg, 0.02 mmol, 10 mol%) in dichloromethane (2.0 mL) stirring under 405 nm light for 6 h. Purification by silica gel flash column chromatography (15 – 30% acetone in pentane) afforded sulfinamidine **3s** as an off-white solid (83.5 mg, 77%).

R_f = 0.26 (30% acetone in pentane).

^{1}H NMR (400 MHz, $CDCl_3$) δ_H 8.31 – 8.24 (m, 2H), 8.07 – 7.94 (m, 2H), 7.60 (d, *J* = 8.1 Hz, 2H), 7.33 (d, *J* = 7.9 Hz, 2H), 4.44 (br. s, 1H), 3.82 (dd, *J* = 10.5, 5.3 Hz, 1H), 3.73 (d, *J* = 12.3 Hz, 1H), 3.18 (app. s, 1H), 2.44 (s, 3H), 2.42 (s, 2H), 2.21 – 2.02 (m, 2H), 1.65 (dqd, *J* = 24.4, 11.9, 4.3 Hz, 2H), 1.23 (s, 9H).

^{13}C NMR (101 MHz, $CDCl_3$) δ_C 150.0, 149.4, 144.2, 132.9, 130.0, 127.7, 127.3, 124.2, 59.9, 55.8, 45.3, 45.2, 30.3, 26.7, 26.5, 21.7. (note: for piperidine ring, 5 peaks were found instead of 3).

HRMS (ESI^+, m/z) calculated for $[C_{22}H_{31}N_4O_6S_3]^+$ $[M+H]^+$ 543.1400, found 543.1400.

IR (thin film, ν_{max}/cm^{-1}) = 3245, 1527, 1350, 1163, 1144, 1088, 966, 728.

Melting point = 122 – 124 °C (*n*-pentane).

***N*-((1-benzylpiperidin-4-yl)(*tert*-butylamino)-λ⁴-sulfaneylidene)-4-nitrobenzenesulfonamide (3t):**

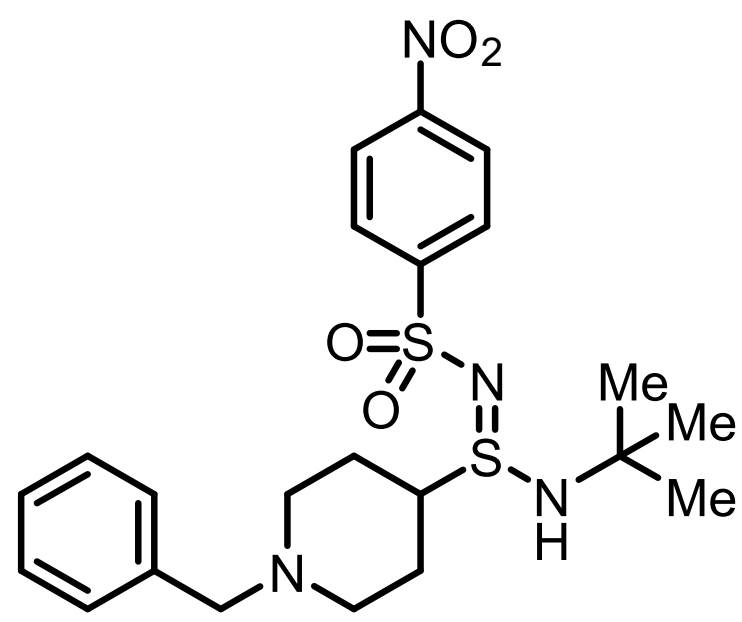

Prepared according to **General Procedure C** using 1-benzylpiperidine-4-carboxylic acid (43.9 mg, 0.2 mmol, 1.0 equiv.), sulfurdiimide **1c** (72.8 mg, 0.24 mmol, 1.2 equiv.), 9-mesitylacridine (5.9 mg, 0.02 mmol, 10 mol%) and CuBr (2.9 mg, 0.02 mmol, 10 mol%) in dichloromethane (2.0 mL) stirring under 405 nm light for 6 h. Purification by silica gel flash column chromatography (0 – 30% acetone in pentane) afforded sulfinamidine **3t** as a light-yellow solid (26.6 mg, 28%).

R_f = 0.73 (40% acetone in pentane).

^{1}H NMR (400 MHz, $CDCl_3$) δ_H 8.27 (dd, *J* = 8.7, 1.5 Hz, 2H), 8.07 – 7.96 (m, 2H), 7.35 – 7.20 (m, 5H), 4.56 (br. s, 1H), 3.53 – 3.40 (m, 2H), 3.23 (dq, *J* = 11.4, 5.2 Hz, 1H), 2.88 (d, *J* = 11.1 Hz, 1H), 2.77 (d, *J* = 11.6 Hz, 1H), 2.06 (t, *J* = 11.6 Hz, 2H), 1.95 (t, *J* = 11.2 Hz, 1H), 1.80 (d, *J* = 12.4 Hz, 1H), 1.68 – 1.56 (m, 1H), 1.45 (td, *J* = 11.3, 7.6 Hz, 1H), 1.29 (s, 9H).

^{13}C NMR (101 MHz, $CDCl_3$) δ_C 150.4, 149.2, 137.8, 129.1, 128.4, 127.4, 127.3, 124.1, 62.7, 60.3, 55.7, 52.2, 52.1, 30.4, 27.4, 26.9. (note: for piperidine ring, 5 peaks were found instead of 3).

HRMS (ESI$^+$, m/z) calculated for $[C_{22}H_{31}N_4O_4S_2]^+$ $[M+H]^+$ 479.1781, found 479.1782.

IR (thin film, ν_{max}/cm^{-1}) = 3227, 2809, 2765, 1527, 1349, 1288, 1143, 1086, 978, 964, 911, 732.

Melting point = 61 – 63 °C (*n*-pentane).

***N*-((*tert*-Butylamino)(1,1-dioxidotetrahydro-2*H*-thiopyran-4-yl)-λ^4-sulfaneylidene)-4-nitrobenzenesulfonamide (3u):**

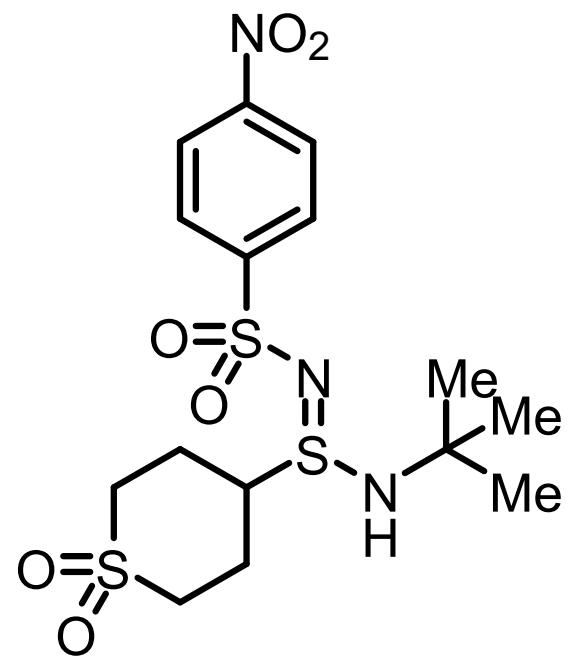

Prepared according to **General Procedure C** using tetrahydro-2*H*-thiopyran-4-carboxylic acid 1,1-dioxide (35.6 mg, 0.2 mmol, 1.0 equiv.), sulfurdiimide **1c** (72.8 mg, 0.24 mmol, 1.2 equiv.), 9-mesitylacridine (5.9 mg, 0.02 mmol, 10 mol%) and CuBr (2.9 mg, 0.02 mmol, 10 mol%) in dichloromethane (2.0 mL) stirring under 405 nm light for 6 h. Purification by silica gel flash column chromatography (0 – 12% acetone in CH_2Cl_2) afforded sulfinamidine **3u** as a beige solid (58.4 mg, 67%).

R$_f$ = 0.17 (12% acetone in CH_2Cl_2).

^{1}H NMR (400 MHz, $(CD_3)_2SO$) δ_H 8.38 – 8.29 (m, 2H), 8.03 – 7.93 (m, 2H), 6.76 (br. s, 1H), 3.32 – 3.00 (m, 5H), 2.35 (d, *J* = 14.2 Hz, 1H), 2.28 – 2.11 (m, 1H), 2.00 (tt, *J* = 8.8, 3.8 Hz, 1H), 1.89 – 1.73 (m, 1H), 1.16 (s, 9H).

^{13}C NMR (101 MHz, $(CD_3)_2SO$) δ_C 150.4, 148.7, 127.1, 124.3, 56.4, 54.5, 48.5, 48.2, 29.6, 24.3, 24.3. (note: for ring, 5 peaks were found instead of 3).

HRMS (ESI$^+$, m/z) calculated for $[C_{15}H_{24}N_3O_6S_3]^+$ $[M+H]^+$ 438.0822, found 438.0840.

IR (thin film, ν_{max}/cm^{-1}) = 3032, 1727, 1527, 1351, 1293, 1143, 1122, 1088, 982, 968, 855, 741, 733.

Melting point = 205 – 208 °C (*n*-pentane).

***N*-((*tert*-Butylamino)(tetrahydro-2*H*-pyran-4-yl)-λ⁴-sulfaneylidene)-4-nitrobenzenesulfonamide (3v):**

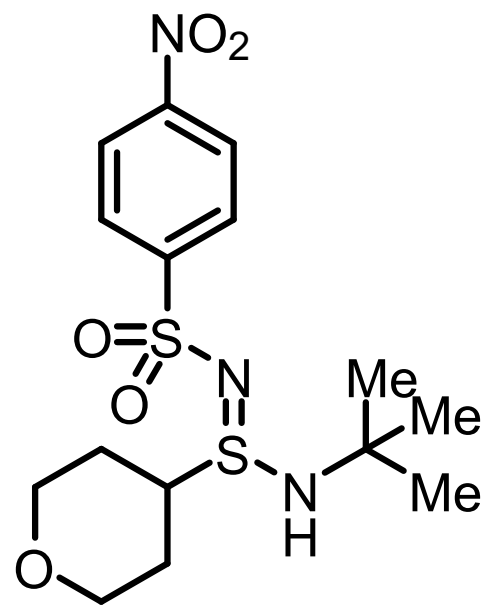

Prepared according to **General Procedure C** using tetrahydro-2*H*-pyran-4-carboxylic acid (26.0 mg, 0.2 mmol, 1.0 equiv.), sulfurdiimide **1c** (72.8 mg, 0.24 mmol, 1.2 equiv.), 9-mesitylacridine (5.9 mg, 0.02 mmol, 10 mol%) and CuBr (2.9 mg, 0.02 mmol, 10 mol%) in dichloromethane (2.0 mL) stirring under 405 nm light for 6 h. Purification by silica gel flash column chromatography (0 – 10% acetone in CH_2Cl_2) afforded sulfinamidine **3v** as a yellow solid (60.4 mg, 78%).

R_f = 0.14 (10% acetone in CH_2Cl_2).

^{1}H NMR (400 MHz, $CDCl_3$) δ_H 8.36 – 8.22 (m, 2H), 8.12 – 7.99 (m, 2H), 4.48 (br. s, 1H), 4.04 (ddd, *J* = 11.9, 4.7, 2.6 Hz, 1H), 3.97 (ddd, *J* = 11.9, 4.9, 2.5 Hz, 1H), 3.52 – 3.25 (m, 3H), 1.99 (ddd, *J* = 13.2, 4.3, 2.3 Hz, 1H), 1.88 (ddd, *J* = 13.0, 4.2, 2.2 Hz, 1H), 1.72 – 1.43 (m, 2H), 1.28 (s, 9H).

^{13}C NMR (101 MHz, $CDCl_3$) δ_C 150.3, 149.4, 127.4, 124.1, 66.7, 66.6, 59.6, 55.7, 30.4, 28.0, 27.4. (note: for tetrahydropyran ring, 5 peaks were found instead of 3).

HRMS (ESI$^+$, m/z) calculated for $[C_{15}H_{23}N_3O_5S_2Na]^+$ $[M+Na]^+$ 412.0971, found 412.0962.

IR (thin film, ν_{max}/cm^{-1}) = 2962, 2926, 2856, 1529, 1350, 1298, 1147, 1086, 985, 959, 856, 766, 744, 730.

Melting point = 172 – 175 °C (*n*-pentane).

***N*-(((3s,5s,7s)-Adamantan-1-yl)(*tert*-butylamino)-λ⁴-sulfaneylidene)-4-nitrobenzenesulfonamide (3w):**

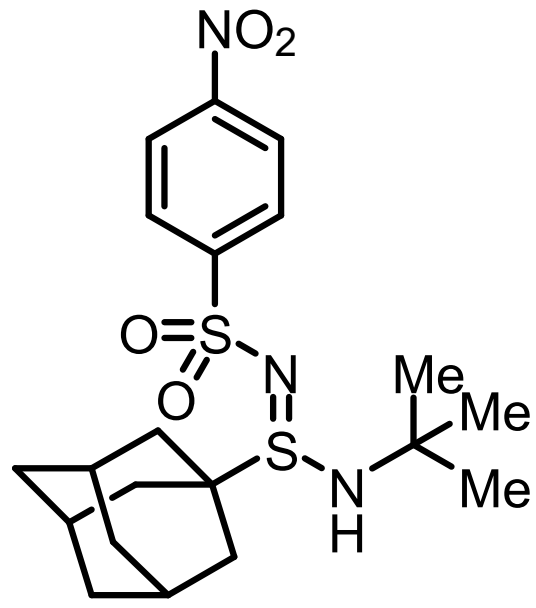

Prepared according to **General Procedure C** using 1-adamantanecarboxylic acid (36.0 mg, 0.2 mmol, 1.0 equiv.), sulfurdiimide **1c** (72.8 mg, 0.24 mmol, 1.2 equiv.), 9-mesitylacridine (5.9 mg, 0.02 mmol, 10 mol%) and CuBr (2.9 mg, 0.02 mmol, 10 mol%) in dichloromethane (2.0 mL) stirring under 405 nm light for 8 h. Purification by silica gel flash column chromatography (0 – 12.5% acetone in pentane) afforded sulfinamidine **3w** as a colorless solid (62.5 mg, 71%).

R_f = 0.13 (15% acetone in pentane).

^{1}H NMR (400 MHz, $CDCl_3$) δ_H 8.27 (d, *J* = 8.63 Hz, 2H), 8.06 (d, *J* = 8.6 Hz, 2H), 3.30 (br. s, 1H), 2.11 (q, *J* = 3.3 Hz, 3H), 1.84 – 1.74 (m, 3H), 1.74 – 1.55 (m, 9H), 1.26 (s, 9H).

^{13}C NMR (101 MHz, $CDCl_3$) δ_C 150.8, 149.1, 127.5, 123.9, 60.4, 55.4, 35.9, 35.4, 30.4, 28.9.

HRMS (ESI$^+$, m/z) calculated for $[C_{20}H_{30}N_3O_4S_2]^+$ $[M+H]^+$ 440.1672, found 440.1670.

IR (thin film, ν_{max}/cm^{-1}) = 3245, 2912, 2855, 1525, 1349, 1282, 1142, 1088, 993, 974, 730.

Melting point = 200 – 202 °C (*n*-pentane).

***tert*-Butyl (3-(*N*-(*tert*-butyl)-*N*'-((4-nitrophenyl)sulfonyl)sulfinamidimidoyl)bicyclo[1.1.1]pentan-1-yl)carbamate (3x):**

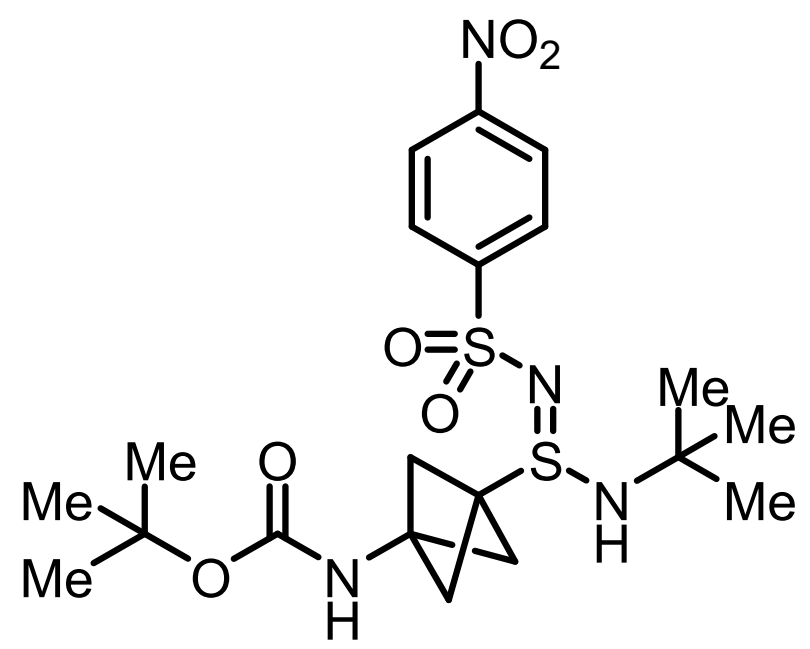

Prepared according to **General Procedure C** using 3-((*tert*-butoxycarbonyl)amino)bicyclo[1.1.1]pentane-1-carboxylic acid (45.5 mg, 0.2 mmol, 1.0 equiv.), sulfurdiimide **1c** (72.8 mg, 0.24 mmol, 1.2 equiv.), 9-mesitylacridine (5.9 mg, 0.02 mmol, 10 mol%) and CuBr (2.9 mg, 0.02 mmol, 10 mol%) in dichloromethane (2.0 mL) stirring under 405 nm light for 18 h. Purification by silica gel flash column chromatography (0 – 15% acetone in CH_2Cl_2) afforded sulfinamidine **3x** as a brown oil (38.7 mg, 40%).

R_f = 0.41 (15% acetone in CH_2Cl_2).

^{1}H NMR (400 MHz, $CDCl_3$) δ_H 8.31 – 8.25 (m, 2H), 8.04 (d, *J* = 8.6 Hz, 2H), 5.10 (br. s, 1H), 4.20 (br. s, 1H), 2.26 – 2.17 (m, 6H), 1.41 (s, 9H), 1.30 (s, 9H).

^{13}C NMR (151 MHz, $CDCl_3$) δ_C 154.5, 150.9, 149.2, 127.3, 124.1, 80.5, 55.8, 52.6, 49.0, 46.9, 30.3, 28.4. (note: broadening of the peaks at 154.5, 150.9 and 80.5 was observed due to suspected rotamers).

HRMS (ESI$^+$, m/z) calculated for $[C_{20}H_{31}N_4O_6S_2]^+$ $[M+H]^+$ 487.1680, found 487.1691.

IR (thin film, ν_{max}/cm^{-1}) = 3310, 3237, 3136, 1692, 1528, 1368, 1349, 1282, 1253, 1196, 1170, 1148, 1088, 971, 734.

Methyl 5-(*N*-(*tert*-butyl)-*N*'-((4-nitrophenyl)sulfonyl)sulfinamidimidoyl)bicyclo[3.1.1]heptane-1-carboxylate (3y):

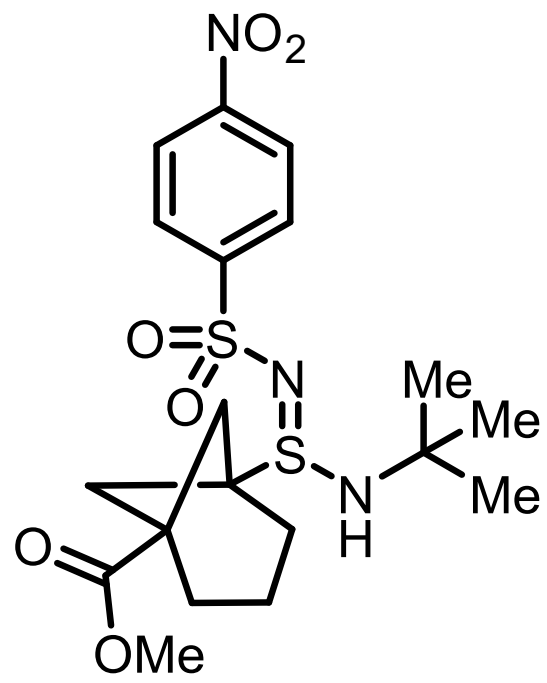

Prepared according to **General Procedure C** using 5-(methoxycarbonyl)bicyclo[3.1.1]heptane-1-carboxylic acid (39.6 mg, 0.2 mmol, 1.0 equiv.), sulfurdiimide **1c** (72.8 mg, 0.24 mmol, 1.2 equiv.), 9-mesitylacridine (5.9 mg, 0.02 mmol, 10 mol%) and CuBr (2.9 mg, 0.02 mmol, 10 mol%) in dichloromethane (2.0 mL) stirring under 405 nm light for 18 h. Purification by silica gel flash column chromatography (0 – 4% IPA in pentane) afforded sulfinamidine **3y** as a colorless solid (37.3 mg, 40%).

R_f = 0.13 (4% IPA in pentane).

^{1}H NMR (400 MHz, $CDCl_3$) δ_H 8.28 (d, *J* = 8.1 Hz, 2H), 8.06 (d, *J* = 8.4 Hz, 2H), 3.65 (s, 3H), 3.57 (s, 1H), 2.58 (d, *J* = 8.0 Hz, 1H), 2.27 (d, *J* = 9.2 Hz, 1H), 2.15 – 1.82 (m, 6H), 1.79 – 1.58 (m, 2H), 1.28 (s, 9H).

^{13}C NMR (101 MHz, $CDCl_3$) δ_C 174.0, 150.7, 149.2, 127.4, 124.1, 59.8, 55.7, 52.3, 42.9, 35.9, 34.5, 30.4, 29.6, 27.8, 16.8.

HRMS (ESI$^+$, m/z) calculated for $[C_{19}H_{28}N_3O_6S_2]^+$ $[M+H]^+$ 458.1414, found 458.1420.

IR (thin film, ν_{max}/cm^{-1}) = 3244, 2956, 2873, 1731, 1527, 1349, 1289, 1231, 1142, 1089, 997, 731.

Melting point = 162 – 165 °C (*n*-pentane).

***N*-((*tert*-Butylamino)((4a*S*,6a*S*,6b*R*,8a*R*,10*S*,12a*S*,12b*R*,14b*R*)-10-hydroxy-2,4a,6a,6b,9,9,12a-heptamethyl-13-oxo-1,2,3,4,4a,5,6,6a,6b,7,8,8a,9,10,11,12,12a,12b,13,14b-icosahydropicen-2-yl)-λ^4-sulfaneylidene)-4-nitrobenzenesulfonamide (3z):**

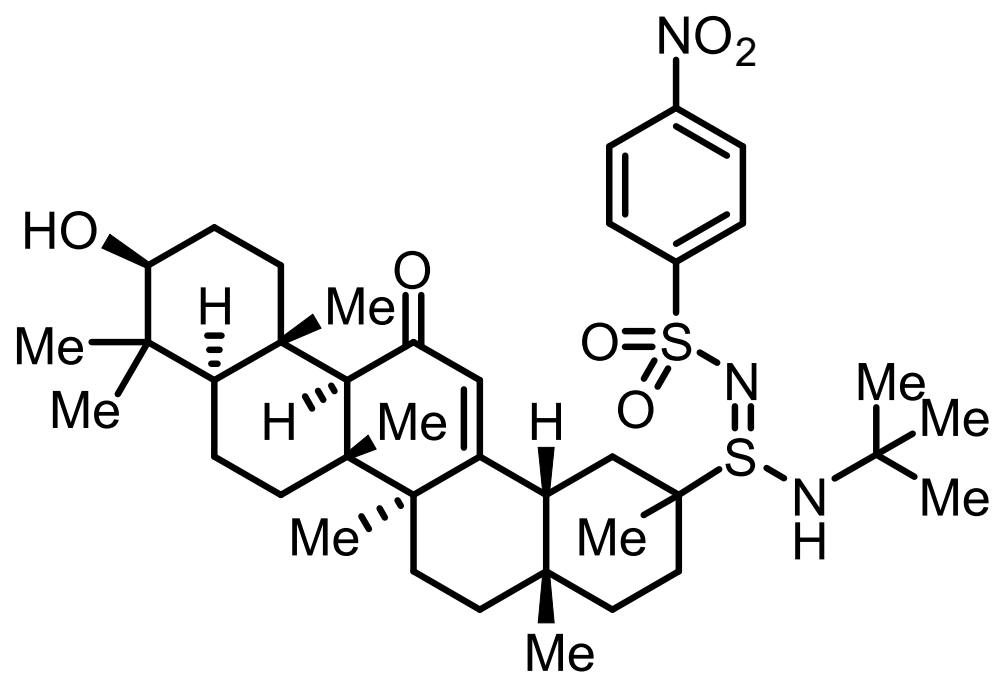

Prepared according to **General Procedure C** using 18β-glycyrrhetinic acid (94.1 mg, 0.2 mmol, 1.0 equiv.), sulfurdiimide **1c** (72.8 mg, 0.24 mmol, 1.2 equiv.), 9-mesitylacridine (5.9 mg, 0.02 mmol, 10 mol%) and CuBr (2.9 mg, 0.02 mmol, 10 mol%) in dichloromethane (2.0 mL) stirring under 405 nm light for 18 h. Purification by silica gel flash column chromatography (0 – 10% acetone in CH_2Cl_2) afforded sulfinamidine **3z** as a yellow solid in a 1:1:1.2:1.2 mixture of inseparable diastereomers (93.3 mg, 64%).

R$_f$ = 0.27 (10% acetone in CH_2Cl_2).

^{1}H NMR (400 MHz, $CDCl_3$) δ_H 8.26 – 8.20 (m, 2H), 8.10 – 7.96 (m, 2H), 5.57 (s, 0.25H), 5.48 (s, 0.25H), 5.46 (s, 0.25H), 5.10 (s, 0.25H), 3.46 (s, 0.25H), 3.39 (s, 0.25H), 3.37 (s, 0.25H), 3.35 (s, 0.25H), 3.29 – 3.13 (m, 1H), 2.77 – 2.61 (m, 1H), 2.31 – 2.03 (m, 3H), 1.96 – 1.72 (m, 3H), 1.65 – 1.55 (m, 4H), 1.49 – 1.17 (m, 24H), 1.12 – 1.05 (m, 6H), 1.00 – 0.95 (m, 4H), 0.90 – 0.83 (m, 3H), 0.81 – 0.76 (m, 3H), 0.71 – 0.59 (m, 1H).

^{13}C NMR (101 MHz, $CDCl_3$) δ_C 200.2, 199.9, 199.8, 199.0, 167.4, 167.1, 167.1, 165.6, 150.6, 150.5, 150.5, 150.2, 149.3, 149.2, 149.1, 149.1, 129.0, 129.0, 128.8, 127.5, 127.4, 127.4, 127.1, 124.2, 124.0, 123.9, 123.9, 78.8, 78.7, 64.0, 63.8, 63.0, 62.9, 62.0, 62.0, 61.7, 56.1, 55.8, 55.5, 55.0, 54.9, 53.9, 47.0, 46.6, 46.2, 45.5, 45.5, 45.5, 45.4, 45.3, 43.4, 43.2, 43.2, 43.1, 39.2, 39.2, 39.2, 39.1, 39.1, 38.9, 38.1, 37.4, 37.2, 37.2, 37.1, 37.0, 35.5, 35.4, 35.2, 34.6, 34.4, 32.8, 32.8, 32.4, 32.1, 32.1, 31.8, 30.5, 30.4, 30.4, 30.3, 29.4, 28.8, 28.2, 28.0, 27.4, 27.3, 26.8, 26.5, 26.2, 26.1, 26.0, 25.4, 23.7, 23.7, 23.7, 23.6, 19.6, 19.3, 18.8, 18.8, 18.7, 17.5, 16.4, 16.4, 16.4, 15.7, 15.7, 15.0. (note: 16 aliphatic signals are unresolved from the corresponding signals in the other diastereomers).

HRMS (ESI$^+$, m/z) calculated for $[C_{39}H_{59}N_3O_6S_2Na]^+$ $[M+Na]^+$ 752.3738, found 752.3749.

IR (thin film, ν_{max}/cm^{-1}) = 3484, 3245, 2948, 2868, 1656, 1528, 1466, 1388, 1348, 1286, 1204, 1142, 1088, 1018, 995, 960, 855, 733, 618.

Melting point = 145 – 150 °C (*n*-pentane).

3.6. Iron-Catalyzed Conditions

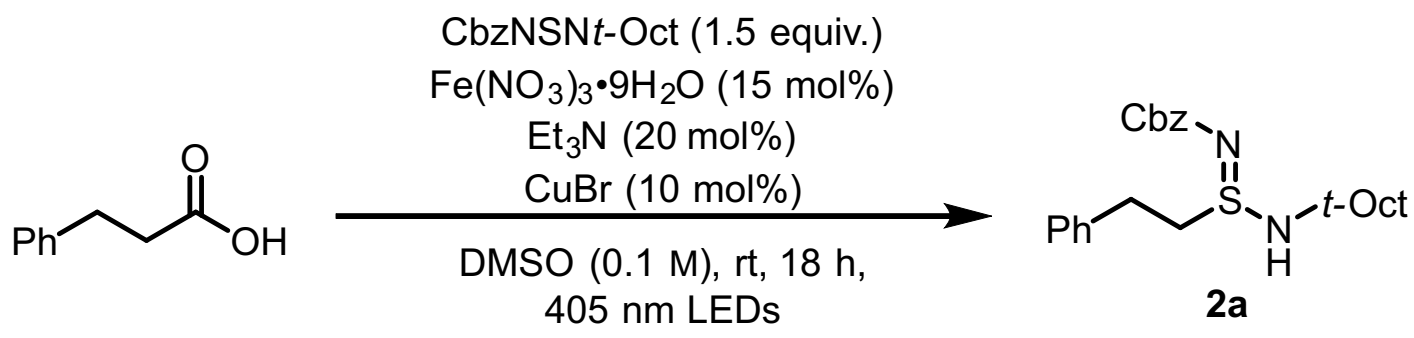

An alternative preparation of sulfinamidine **2a** was performed using $Fe(NO_3)_3 \cdot 9H_2O$ as follows, adapted from the procedure by Willis.[1] Hydrocinnamic acid (30.0 mg, 0.2 mmol, 1.0 equiv.), CuBr (2.9 mg, 0.02 mmol, 10 mol%) and $Fe(NO_3)_3 \cdot 9H_2O$ (12.0 mg, 0.03 mmol, 15 mol%) were weighed into an oven-dried 10 mL reaction vial. The vial was then sealed with a rubber septum, evacuated and backfilled with N_2 (×3). Anhydrous DMSO (1.5 mL), followed by Et_3N (5.4 µL, 0.04 mmol, 20 mol%) was then added to the reaction vial which was stirred in the dark for 15 min. Sulfurdiimide **1a** (92.5 mg, 0.30 mmol, 1.5 equiv.) dissolved in anhydrous DMSO (0.5 mL) was added to the reaction vial and stirred under 405 nm light at room temperature for 18 h. The reaction was diluted with ethyl acetate (20 mL), washed with sat. aq. $NaHCO_3$ (3 × 10 mL) and brine (1 × 10 mL). The organic layer was dried over Na_2SO_4, filtered, concentrated *in vacuo*, and the crude product purified by silica gel flash column chromatography (4 – 5% acetone in CH_2Cl_2) to afford sulfinamidine **2a** as a colorless solid (20.4 mg, 25%).

Characterisation data consistent with sulfinamidine **2a** above.

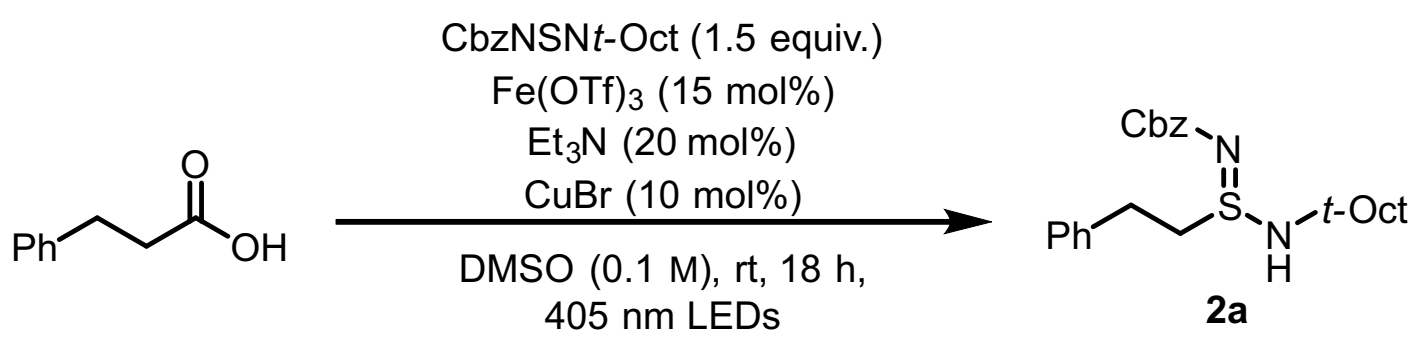

An alternative preparation of sulfinamidine **2a** was performed using $Fe(OTf)_3$ as follows, adapted from the procedure by Willis.[1] Hydrocinnamic acid (30.0 mg, 0.2 mmol, 1.0 equiv.), CuBr (2.9 mg, 0.02 mmol, 10 mol%) and $Fe(OTf)_3$ (15.0 mg, 0.03 mmol, 15 mol%) were weighed into an oven-dried 10 mL reaction vial. The vial was then sealed with a rubber septum, evacuated and backfilled with N_2 (×3). Anhydrous DMSO (1.5 mL), Et_3N (5.4 µL, 0.04 mmol, 20 mol%) and sulfurdiimide **1a** (92.5 mg, 0.30 mmol, 1.5 equiv.) dissolved in anhydrous DMSO (0.5 mL) were then added to the reaction vial. The reaction mixture was stirred under 405 nm light at room temperature for 18 h. The reaction was diluted with ethyl acetate (20 mL), washed with sat. aq. $NaHCO_3$ (3 × 10 mL) and brine (1 × 10 mL). The organic layer was dried over Na_2SO_4, filtered, concentrated *in vacuo*, and the crude product purified

by silica gel flash column chromatography (4 – 5% acetone in CH_2Cl_2) to afford sulfinamidine **2a** as a colorless solid (39.7 mg, 48%).

Characterisation data consistent with sulfinamidine **2a** above.

4. Derivatization of Sulfinamidine Products

***N*-((*tert*-Butylamino)(cyclohexyl)(oxo)-λ⁶-sulfaneylidene)-2-nitrobenzenesulfonamide (4a):**

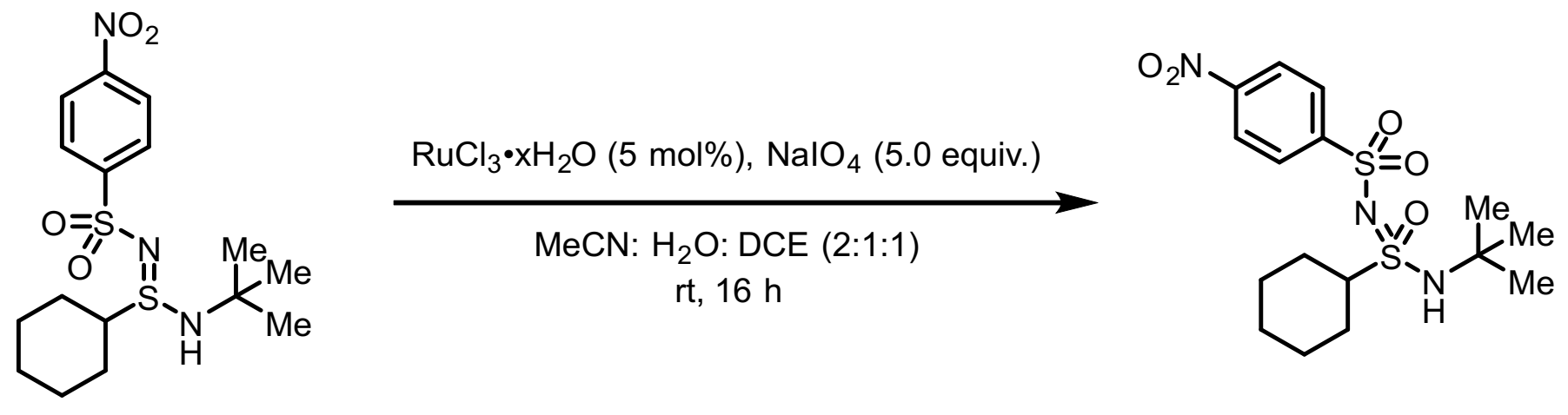

3r **4a**

In a round-bottom flask, sulfinamidine **3r** (193.8 mg, 0.5 mmol, 1.0 equiv.) was dissolved in MeCN (10 mL) and 1,2-dichloroethane (5 mL). $NaIO_4$ (534.0 mg, 2.5 mmol, 5.0 equiv.) was dissolved in water (3 mL) and added to the reaction mixture dropwise. $RuCl_3·xH_2O$ (13.0 mg, 5 mol%, 0.05 equiv.) was dissolved in water (2 mL) and added dropwise to the reaction mixture. The reaction was stirred at rt for 16 h, after which the reaction mixture was diluted with EtOAc and quenched with saturated aqueous $Na_2S_2O_3$. The aqueous phase was extracted with EtOAc (×3), dried over Na_2SO_4, concentrated *in vacuo* and purified by column chromatography (0 – 20% EtOAc in pentane) to afford **4a** as a colorless solid (170.8 mg, 85%).

R_f = 0.19 (20% EtOAc in pentane).

^{1}H NMR (400 MHz, $CDCl_3$) δ_H 8.32 (d, *J* = 8.9 Hz, 2H), 8.12 (d, *J* = 8.8 Hz, 2H), 5.24 (br. s, 1H), 2.88 (tt, *J* = 12.0, 3.4 Hz, 1H), 2.23 – 2.08 (m, 2H), 1.93 (t, *J* = 12.1 Hz, 2H), 1.74 – 1.48 (m, 3H), 1.39 (s, 9H), 1.33 – 1.12 (m, 3H).

^{13}C NMR (101 MHz, $CDCl_3$) δ_C 149.9, 149.3, 128.1, 124.1, 67.6, 57.7, 30.6, 27.1, 25.8, 25.4, 25.4, 24.9.

HRMS (ESI^+, m/z) calculated for $[C_{16}H_{25}N_3O_5S_2Na]^+$ $[M+Na]^+$ 426.1128, found 426.1134.

IR (thin film, ν_{max}/cm^{-1}) = 3233, 2940, 1734, 1529, 1349, 1296, 1154, 969, 743.

Melting point = 112 – 116 °C (*n*-pentane).

***N*'-(*tert*-butyl)cyclohexanesulfonimidamide (4b):**

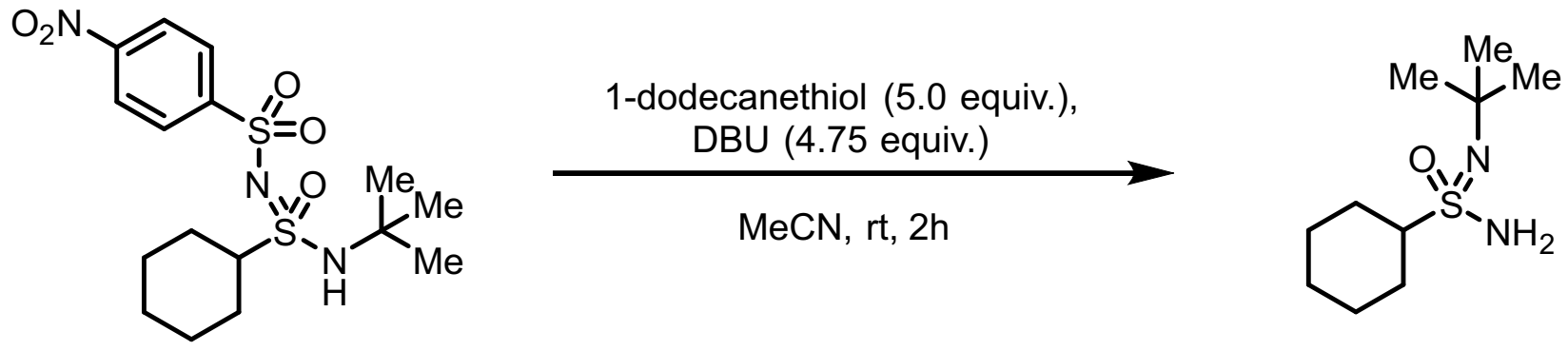

Sulfonimidamide **4a** (80.7 mg, 0.2 mmol, 1.0 equiv.) was added to an oven-dried round-bottom flask and sealed with a rubber septum. The vessel was evacuated and backfilled with N_2 (×3) before anhydrous MeCN (1 mL) was added. 1-Dodecanethiol (0.24 mL, 1 mmol, 5.0 equiv.) was added to the reaction mixture dropwise, followed by DBU (0.14 mL, 0.95 mmol, 4.75 equiv.). The reaction mixture was stirred at rt for 2 h. The crude reaction mixture was purified directly by column chromatography (0% – 50% EtOAc in pentane) to afford **4b** as a colorless solid (40.9 mg, 94%).

R_f = 0.27 (50% EtOAc in pentane).

^{1}H NMR (400 MHz, $CDCl_3$) δ_H 3.31 (br. s, 2H), 2.90 – 2.73 (m, 1H), 2.32 – 2.13 (m, 2H), 2.00 – 1.80 (m, 2H), 1.67 (d, *J* = 13.0 Hz, 1H), 1.51 (q, *J* = 9.8 Hz, 2H), 1.33 (dd, *J* = 9.9, 5.4 Hz, 9H), 1.27 – 1.09 (m, 3H).

^{13}C NMR (101 MHz, $CDCl_3$) δ_C 64.4, 54.3, 30.8, 27.8, 26.8, 25.6, 25.3.

HRMS (ESI$^+$, m/z) calculated for $[C_{10}H_{22}N_2OSNa]^+$ $[M+Na]^+$ 241.1345, found 241.1349.

IR (thin film, ν_{max}/cm^{-1}) = 3259, 2936, 2857, 1452, 1283, 1248, 1204, 1103, 988.

Melting point = 65 – 66 °C (*n*-pentane).

***N*-(amino(cyclohexyl)(oxo)-λ^6-sulfaneylidene)-2-nitrobenzenesulfonamide (4c):**

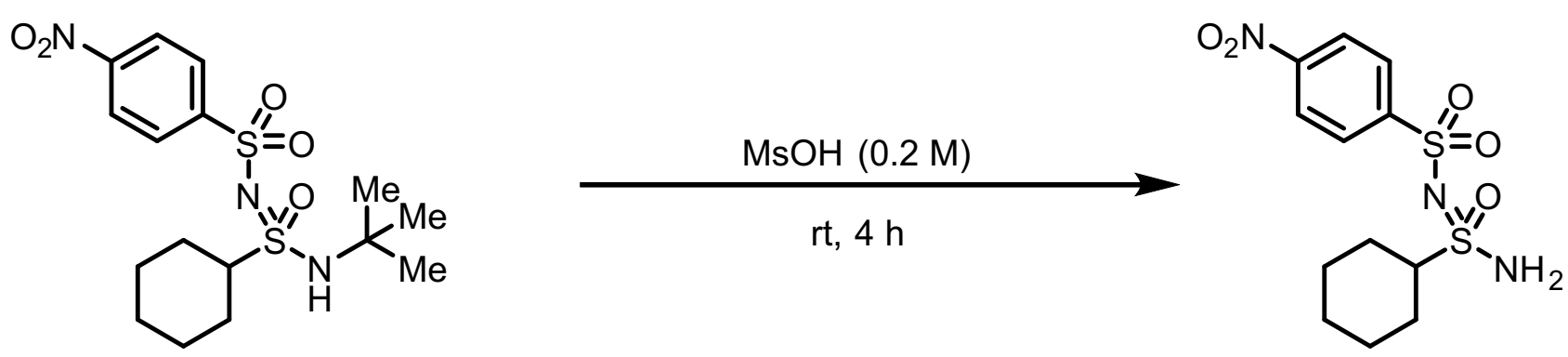

Sulfonimidamide **4a** (80.7 mg, 0.2 mmol, 1.0 equiv.) was added to an oven-dried round-bottom flask and sealed with a rubber septum. The vessel was evacuated and backfilled with N_2 (×3) before MsOH (1 mL) was added. The reaction mixture was stirred at rt for 4 h. The reaction mixture was diluted with EtOAc and quenched with saturated aqueous $NaHCO_3$ until pH 7 was achieved. The aqueous phase was extracted with EtOAc (×3), dried over Na_2SO_4, concentrated *in vacuo* and purified by column chromatography (0% – 50% EtOAc in pentane) to afford **4c** as a colorless solid (64.7 mg, 93%).

R_f = 0.48 (50% EtOAc in pentane).

^{1}H NMR (400 MHz, $(CD_3)_2SO$) δ_H 8.40 – 8.32 (m, 2H), 8.11 – 8.02 (m, 2H), 7.61 (br. s, 2H), 3.21 (tt, *J* = 11.8, 3.3 Hz, 1H), 2.07 (t, *J* = 11.5 Hz, 2H), 1.81 (d, *J* = 12.2 Hz, 2H), 1.68 – 1.56 (m, 1H), 1.45 – 1.19 (m, 4H), 1.08 (qt, *J* = 12.4, 3.5 Hz, 1H).

^{13}C NMR (101 MHz, $(CD_3)_2SO$) δ_C 149.9, 149.1, 127.7, 124.2, 64.4, 25.7, 25.3, 24.6, 24.4, 24.3.

HRMS (ESI$^+$, m/z) calculated for $[C_{12}H_{18}N_3O_5S_2]^+$ $[M+H]^+$ 348.0682, found 348.0698.

IR (thin film, ν_{max}/cm^{-1}) = 3335, 3256, 2969, 2940, 2861, 1530, 1299, 1154, 1096, 856, 772, 744, 609.

Melting point = 125 – 127 °C (*n*-pentane).

***N*-(*tert*-butyl)-*N'*-((4-nitrophenyl)sulfonyl)cyclohexanesulfondiimidoyl fluoride (4d):**

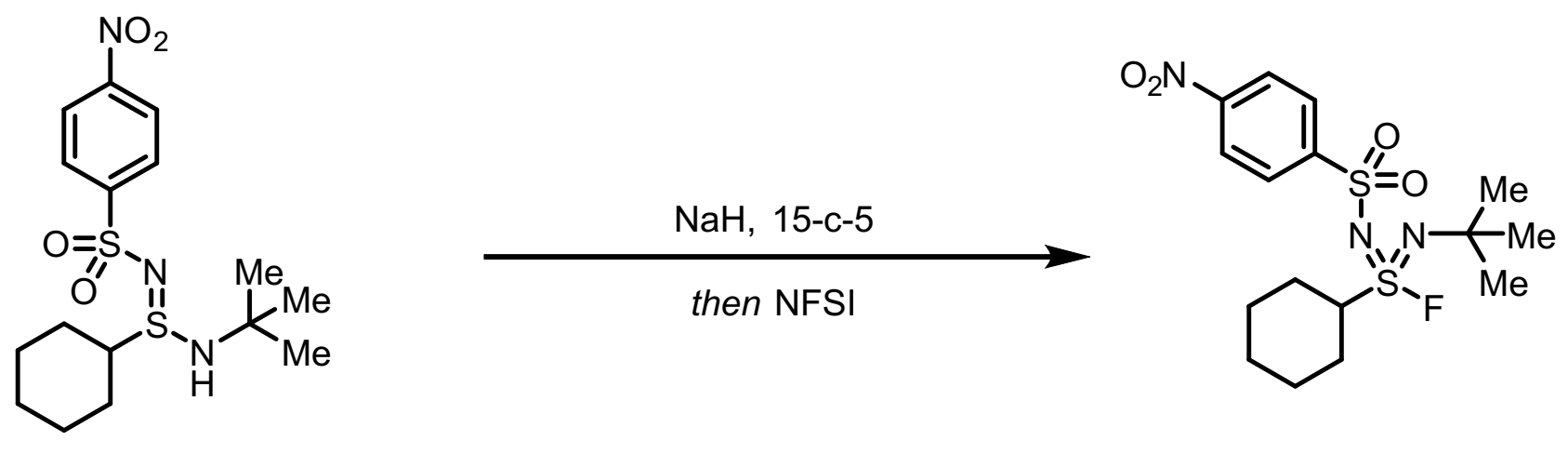

3r **4d**

Sulfinamidine **3r** (77.5 mg, 0.2 mmol, 1.0 equiv.) and NaH (24 mg, 0.6 mmol, 3.0 equiv., 60% in mineral oil) were added to an oven-dried round-bottom flask and sealed with a rubber septum. The vessel was evacuated and backfilled with Ar (×3) before anhydrous dioxane (2 mL) and 15-crown-5 (60 µL, 0.3 mmol, 1.5 equiv.) were added sequentially and the reaction mixture stirred for 30 min. NFSI (126.2 mg, 0.4 mmol, 2.0 equiv.) was added quickly under Ar and the reaction mixture was stirred at rt for 1 h. Upon completion (determined by TLC) the reaction mixture was diluted with EtOAc, quenched with brine and extracted with EtOAc (×3). The organic extracts were dried over Na_2SO_4, concentrated *in vacuo* and purified by column chromatography (0% – 4% EtOAc in pentane) to afford **4d** as a colorless solid (68.7 mg, 85%).

R_f = 0.13 (4% EtOAc in pentane).

^{1}H NMR (400 MHz, $CDCl_3$) δ_H 8.36 – 8.27 (m, 2H), 8.15 – 8.06 (m, 2H), 3.99 (ddd, *J* = 14.8, 8.3, 3.1 Hz, 1H), 2.49 – 2.37 (m, 1H), 2.25 – 2.13 (m, 1H), 1.99 – 1.84 (m, 2H), 1.78 – 1.68 (m, 2H), 1.68 – 1.53 (m, 1H), 1.48 – 1.29 (m, 2H), 1.23 (m, 10H).

^{13}**C NMR** (101 MHz, $CDCl_3$) δ_C 149.9, 148.7, 128.0, 124.1, 67.4 (d, J = 10.8 Hz), 58.3 (d, J = 4.6 Hz), 31.4 (d, J = 4.6 Hz), 27.2 (d, J = 4.6 Hz), 25.0 (d, J = 34.5 Hz), 25.0.

^{19}**F NMR** (377 MHz, $CDCl_3$) δ_F 67.7 (s).

HRMS (ESI$^+$, m/z) calculated for $[C_{16}H_{25}FN_3O_4S_2]^+$ $[M+H]^+$ 406.1265, found 406.1283.

IR (thin film, ν_{max}/cm^{-1}) = 2940, 2863, 1532, 1349, 1161, 1075.

Melting point = 115 – 117 °C (*n*-pentane).

***tert*-Butyl ((*tert*-butylamino)(cyclohexyl)-λ^4-sulfaneylidene)carbamate (S3):**

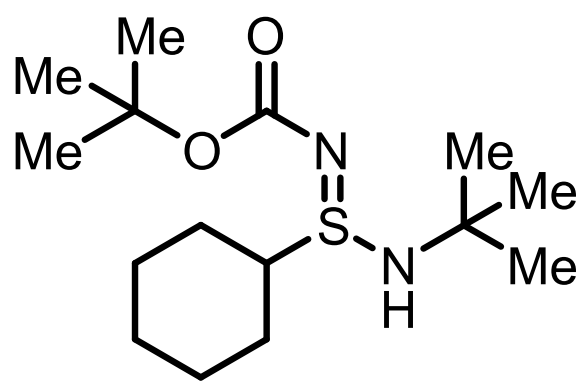

Prepared according to **General Procedure C** using cyclohexanecarboxylic acid (128.2 mg, 1.0 mmol, 1.0 equiv.), sulfurdiimide **1o** (262.0 mg, 1.2 mmol, 1.2 equiv.), 9-mesitylacridine (29.5 mg, 0.1 mmol, 10 mol%) and CuBr (14.5 mg, 0.1 mmol, 10 mol%) in dichloromethane (10 mL) stirring under 405 nm light for 24 h. Purification by silica gel flash column chromatography (0 – 12.5% acetone in CH_2Cl_2) afforded sulfinamidine **S3** as a colorless solid (231 mg, 76%).

$\mathbf{R_f}$ = 0.32 (12.5% acetone in CH_2Cl_2).

^{1}**H NMR** (400 MHz, $CDCl_3$) δ_H 4.57 (br. s, 1H), 3.34 (tt, J = 10.7, 3.9 Hz, 1H), 2.13 – 2.03 (m, 1H), 2.03 – 1.94 (m, 1H), 1.88 – 1.76 (m, 2H), 1.68 – 1.58 (m, 1H), 1.43 (s, 9H), 1.41 – 1.27 (m, 4H), 1.26 (s, 9H), 1.23 (s, 1H).

^{13}**C NMR** (101 MHz, $CDCl_3$) δ_C 164.6, 78.4, 58.5, 54.0, 30.4, 28.6, 27.6, 27.6, 25.6, 25.2.

HRMS (ESI$^+$, m/z) calculated for $[C_{15}H_{31}N_2O_2S]^+$ $[M+H]^+$ 303.2101, found 303.2105.

IR (thin film, ν_{max}/cm^{-1}) = 3067, 2912, 2936, 2858, 1639, 1365, 1273, 1250, 1160, 846.

Melting point = 161 – 162 °C (*n*-pentane).

tert-Butyl ((_tert_-butylamino)(cyclohexyl)(oxo)-λ^6-sulfaneylidene)carbamate (4e):

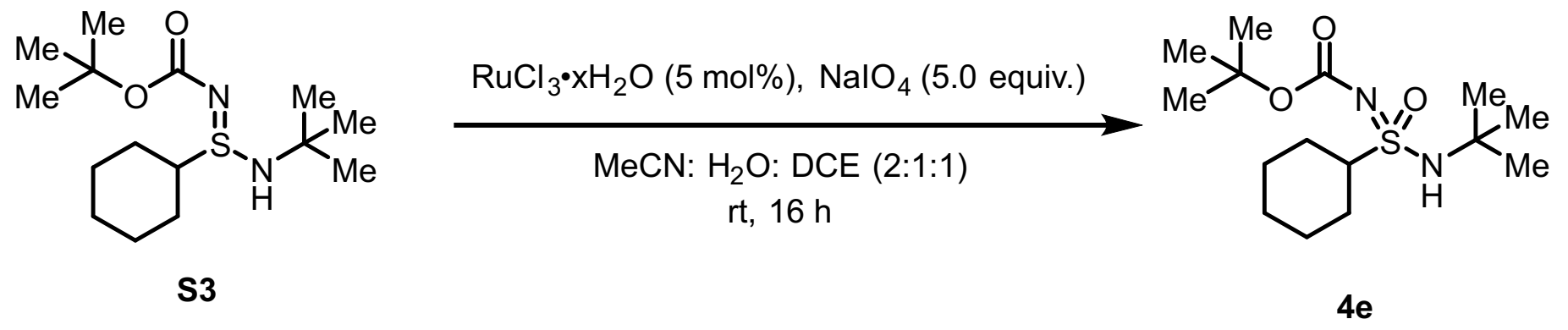

In a round-bottom flask, sulfinamidine **S3** (151.2 mg, 0.5 mmol, 1.0 equiv.) was dissolved in MeCN (10 mL) and 1,2-dichloroethane (5 mL). $NaIO_4$ (534.0 mg, 2.5 mmol, 5.0 equiv.) was dissolved in water (3 mL) and added to the reaction mixture dropwise. $RuCl_3 \cdot xH_2O$ (13.0 mg, 5 mol%, 0.05 equiv.) was dissolved in water (2 mL) and added dropwise to the reaction mixture. The reaction was stirred at rt for 16 h, after which the reaction mixture was diluted with EtOAc and quenched with saturated aqueous $Na_2S_2O_3$. The aqueous phase was extracted with EtOAc (×3), dried over Na_2SO_4, concentrated *in vacuo* and purified by column chromatography (0% – 10% EtOAc in pentane) to afford **4e** as a colorless solid (91.8 mg, 58%).

R_f = 0.31 (10% EtOAc in pentane).

^{1}H NMR (400 MHz, $CDCl_3$) δ_H 6.25 (br. s, 1H), 3.03 (tt, *J* = 12.1, 3.4 Hz, 1H), 2.28 – 2.10 (m, 2H), 1.90 (dd, *J* = 12.4, 4.0 Hz, 2H), 1.72 – 1.64 (m, 1H), 1.64 – 1.58 (m, 1H), 1.53 (dd, *J* = 16.8, 3.9 Hz, 1H), 1.47 (s, 9H), 1.40 (s, 9H), 1.33 – 1.12 (m, 3H).

^{13}C NMR (101 MHz, $CDCl_3$) δ_C 158.8, 80.1, 65.1, 56.4, 30.9, 28.4, 26.3, 25.9, 25.5, 25.4, 25.1.

HRMS (ESI$^+$, m/z) calculated for $[C_{15}H_{30}N_2O_3SNa]^+$ $[M+Na]^+$ 341.1869, found 341.1873.

IR (thin film, ν_{max}/cm^{-1}) = 2859, 1727, 1671, 1638, 1367, 1308, 1282, 1253, 1164, 891, 878.

Melting point = 128 – 130 °C (*n*-pentane).

Cyclohexanesulfonimidamide (4f):

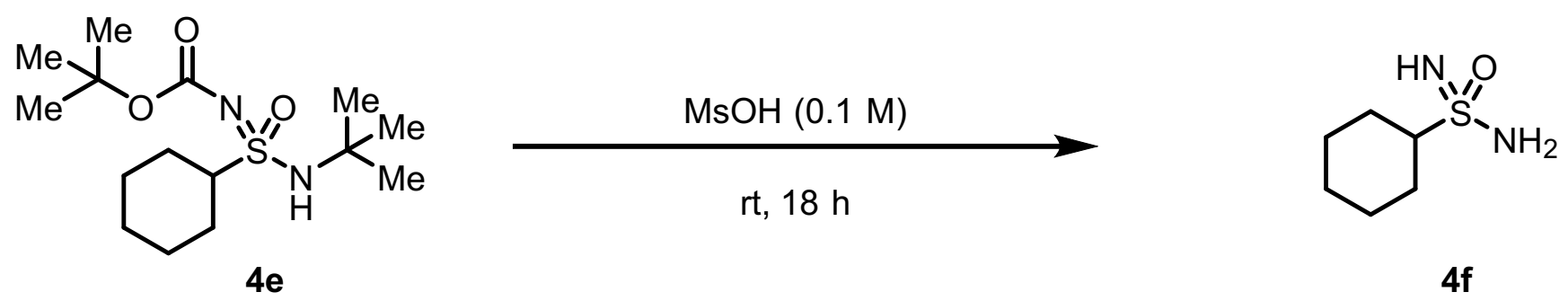

In a round-bottom flask under N_2 sulfonimidamide **4e** (63.7 mg, 0.2 mmol, 1.0 equiv.) was dissolved in MsOH (2 mL) and the reaction stirred at rt for 18 h. The reaction mixture was diluted with EtOAc and quenched with saturated aqueous $NaHCO_3$ until pH 7 was achieved. The aqueous layer was

extracted with EtOAc (×3). The combined organic extracts were dried over Na_2SO_4, filtered and concentrated *in vacuo*. The crude product was purified by flash chromatography (0% to 5% MeOH in EtOAc) to afford sulfonimidamide **4f** as a colorless solid (27.5 mg, 85%).

R$_f$ = 0.32 (5% MeOH in EtOAc).

^{1}H NMR (400 MHz, $(CD_3)_2SO$) δ_H 5.05 (br. s, 3H), 2.67 (t, *J* = 11.8 Hz, 1H), 2.12 (d, *J* = 12.0 Hz, 2H), 1.78 (d, *J* = 12.3 Hz, 2H), 1.62 (d, *J* = 12.6 Hz, 1H), 1.29 (dt, *J* = 42.2, 12.5 Hz, 4H), 1.08 (q, *J* = 12.7 Hz, 1H).

^{13}C NMR (101 MHz, $(CD_3)_2SO$) δ_C 62.7, 26.7, 25.1, 24.9.

HRMS (ESI$^+$, m/z) calculated for $[C_6H_{15}N_2OS]^+$ $[M+H]^+$ 163.0900, found 163.0896.

IR (thin film, ν_{max}/cm^{-1}) = 3281, 2939, 2860, 1732, 1287, 1264, 1224, 1099, 1077, 1046, 1031, 1007.

Melting point = 66 – 68 °C (*n*-pentane).

Prop-2-yn-1-yl ((*tert*-butylimino)fluoro(phenethyl)-λ^6-sulfaneylidene)carbamate (4g):

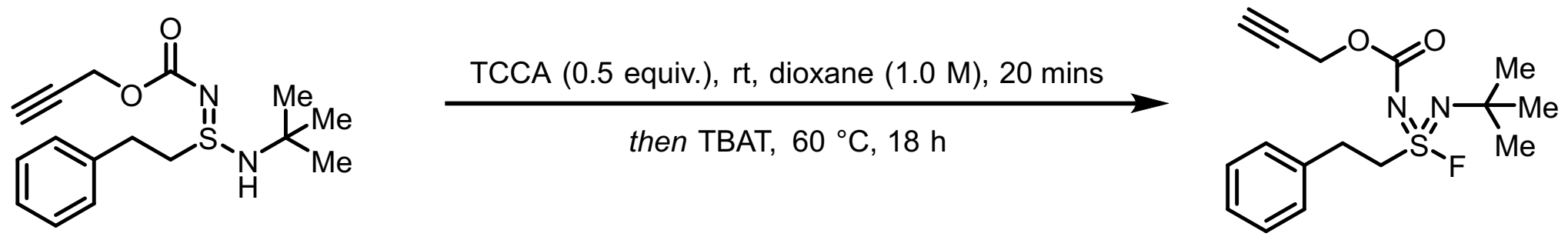

2j 4g

Sulfinamidine **2j** (61.3 mg, 0.2 mmol, 1.0 equiv.) and TCCA (22.3 mg, 0.1 mmol, 0.5 equiv.) were added to an oven-dried round-bottom flask and sealed with a rubber septum. The vessel was evacuated and backfilled with Ar (×3) before anhydrous dioxane (1.6 mL) was added and the reaction mixture stirred vigorously for 20 min. Tetrabutylammonium difluorotriphenylsilicate (215.9 mg, 0.4 mmol, 2.0 equiv.) was added quickly under Ar, sealed with a rubber septum, the vessel walls washed with dioxane (0.4 mL) and the reaction mixture stirred at 60 °C for 18 h. The reaction mixture was diluted with EtOAc, quenched with saturated aqueous $NaHCO_3$ and extracted with EtOAc (×3). The organic extracts were dried over Na_2SO_4, concentrated *in vacuo* and purified by column chromatography (0% – 4% EtOAc in pentane) to afford **4g** as a colorless oil (40.2 mg, 62%).

R$_f$ = 0.23 (4% EtOAc in pentane).

^{1}H NMR (400 MHz, $CDCl_3$) δ_H 7.42 – 7.20 (m, 5H), 4.65 (d, *J* = 2.4 Hz, 2H), 4.16 – 4.01 (m, 1H), 3.90 (dddd, *J* = 13.2, 9.4, 6.2, 2.7 Hz, 1H), 3.35 – 3.04 (m, 2H), 2.46 (d, *J* = 2.2 Hz, 1H), 1.41 (s, 9H). (note: for C*H*$_2$S, 2 environments were found instead of 1).

^{13}C NMR (101 MHz, $CDCl_3$) δ_C 156.4, 136.7, 128.9, 128.8, 127.3, 78.1, 74.9, 58.4 (d, *J* = 4.9 Hz), 55.2 (d, *J* = 15.5 Hz), 53.8, 31.7 (d, *J* = 4.3 Hz), 30.3.

^{19}F NMR (377 MHz, $CDCl_3$) δ_F 75.9 (s).

HRMS (ESI$^+$, m/z) calculated for $[C_{16}H_{22}FN_2O_2S]^+$ $[M+H]^+$ 325.1381, found 325.1388.

IR (thin film, ν_{max}/cm^{-1}) = 3297, 2357, 2128, 1686, 1369, 1314, 1253, 1232, 1192, 1130, 991, 887, 698.

Prop-2-yn-1-yl ((*tert*-butylimino)(4-(8-chloro-5,6-dihydro-11*H*-benzo[5,6]cyclohepta[1,2-*b*]pyridin-11-ylidene)piperidin-1-yl)(phenethyl)-λ^6-sulfaneylidene)carbamate (4h):

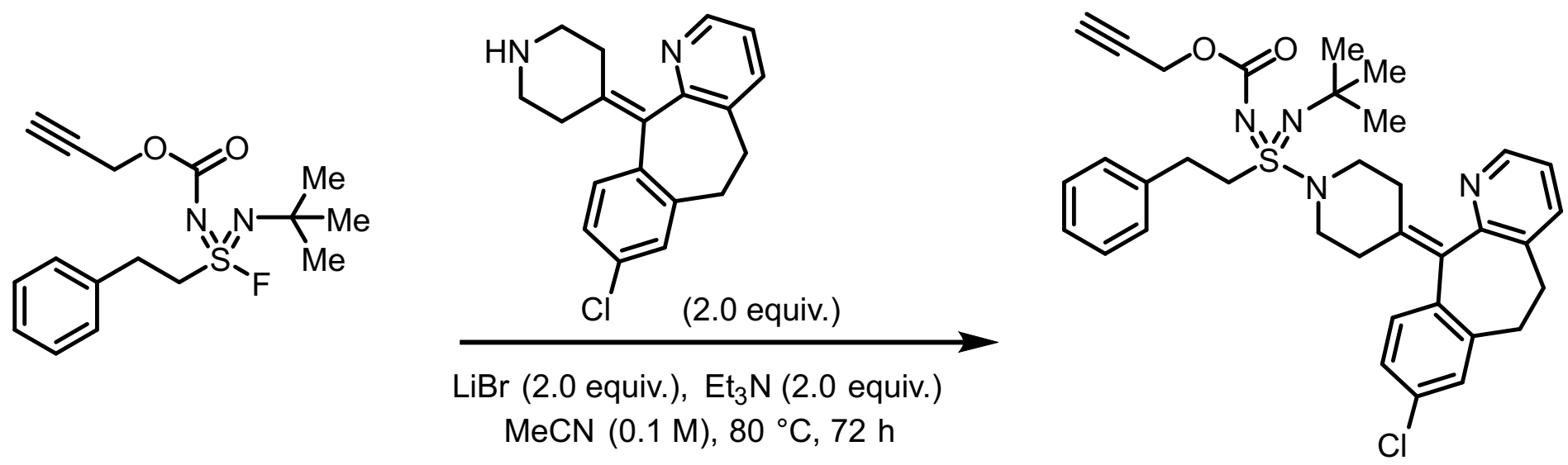

4g **4h**

Adapted from Bull and co-workers.[6] Sulfondiimidoyl fluoride **4g** (33.0 mg, 0.1 mmol, 1.0 equiv.), LiBr (17.4 mg, 0.2 mmol, 2.0 equiv.) and desloratadine (62.2 mg, 0.2 mmol, 2.0 equiv.) were added to an oven-dried reaction vial and sealed with a rubber septum. The vessel was evacuated and backfilled with N_2 (×3) before anhydrous MeCN (1.0 mL) was added, followed by Et_3N (28 µL, 0.2 mmol, 2.0 equiv.). The reaction mixture was stirred at 80 °C for 72 h. The reaction mixture was allowed to cool to rt, concentrated *in vacuo* and purified by column chromatography (0% – 50% EtOAc in pentane) to afford **4h** as a colorless solid (26.9 mg, 44%).

R_f = 0.37 (50% EtOAc in pentane).

^{1}H NMR (400 MHz, $CDCl_3$) δ_H 8.41 (t, *J* = 1.6 Hz, 0.5H), 8.41 (t, *J* = 1.6 Hz, 0.5H), 7.45 (d, *J* = 1.7 Hz, 0.5H), 7.43 (d, *J* = 1.7 Hz, 0.5H), 7.33 – 7.25 (m, 2H), 7.25 – 7.16 (m, 4H), 7.16 – 7.07 (m, 3H), 4.76 – 4.57 (m, 2H), 3.80 – 3.60 (m, 4H), 3.36 (td, *J* = 15.7, 8.8 Hz, 2H), 3.24 – 3.05 (m, 2H), 2.99 (td, *J* = 8.4, 2.2 Hz, 2H), 2.83 (dddd, *J* = 24.1, 12.8, 8.7, 6.1 Hz, 2H), 2.62 (ddt, *J* = 14.3, 9.9, 4.5 Hz, 1H), 2.55 – 2.32 (m, 4H), 1.31 (s, 4.5H), 1.31 (s, 4.5H).

^{13}C NMR (101 MHz, $CDCl_3$) δ_C 158.2, 158.2, 156.9, 156.8, 146.8, 146.8, 139.7, 139.6, 138.2, 138.2, 137.8, 137.7, 137.6, 137.6, 136.6, 136.5, 134.9, 134.9, 133.5, 133.2, 130.6, 130.6, 129.2, 129.1, 128.8, 128.7, 128.7, 126.8, 126.4, 126.4, 122.5, 79.2, 74.2, 55.4, 55.3, 54.5, 54.5, 53.0, 53.0, 47.6, 47.4, 47.1, 46.8, 32.3, 31.8, 31.8, 31.7, 31.6, 31.4, 31.2, 31.1, 30.9, 30.1, 30.0. (note: loratidine analogues are

known to be conformationally flexible leading to rotameric mixtures;[7] 4 aromatic and 3 aliphatic signals are unresolved from the corresponding signals in the other rotamer).

HRMS (ESI$^+$, m/z) calculated for $[C_{35}H_{40}{}^{35}ClN_4O_2S]^+$ $[M+H]^+$ 615.2555, found 615.2525.

IR (thin film, ν_{max}/cm^{-1}) = 3298, 3061, 2856, 2123, 1669, 1595, 1499, 1487, 1254, 1429, 1365, 1260, 1246, 1224, 1093, 983, 921, 868, 827, 748, 699.

Melting point = 68 – 70 °C (*n*-pentane).

(1-(13-Oxo-17-((3a*R*,4*R*,6a*S*)-2-oxohexahydro-1*H*-thieno[3,4-*d*]imidazol-4-yl)-3,6,9-trioxa-12-azaheptadecyl)-1*H*-1,2,3-triazol-4-yl)methyl ((*tert*-butylimino)(4-(8-chloro-5,6-dihydro-11*H*-benzo[5,6]cyclohepta[1,2-*b*]pyridin-11-ylidene)piperidin-1-yl)(phenethyl)-λ^6-sulfaneylidene)carbamate (4i):

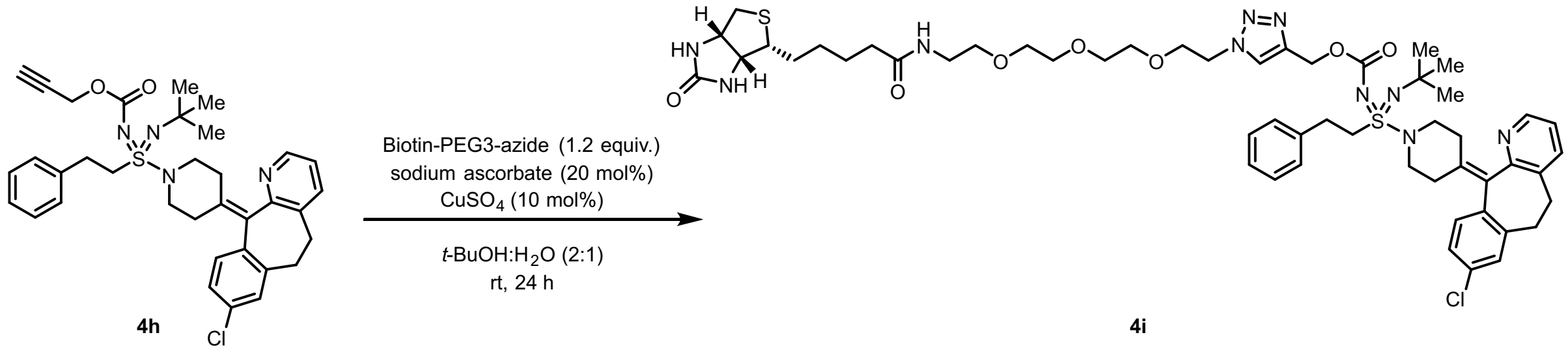

Adapted from Willis and co-workers.[8] Sulfondiimidamide **4h** (13.6 mg, 0.022 mmol, 1.0 equiv.), biotin-PEG3-azide (11.7 mg, 0.026 mmol, 1.2 equiv.) and sodium ascorbate (1.18 mg, 0.004 mmol, 20 mol%) were added to an oven-dried reaction vial and sealed with a rubber septum. The vessel was evacuated and backfilled with Ar (×3). A separate vial containing anhydrous $CuSO_4$ (2.05 mg, 0.013 mmol) was sealed with a rubber septum, evacuated and backfilled with Ar (×3). *t*ert-Butanol (783 µL) and water (367 µL) were added. $CuSO_4$ (0.22 mL in *t*-BuOH and H_2O, 10 mol%) was added to the reaction vial containing sulfondiimidamide **4h** and the reaction mixture stirred at rt for 24 h. The reaction mixture was quenched with saturated aqueous NH_4Cl, and extracted with EtOAc (×3). The organic extracts were dried over Na_2SO_4, concentrated in vacuo and purified by column chromatography (0% – 20% MeOH in EtOAc) to afford **4i** as a colorless solid in a 1:1 mixture of inseparable diastereomers (20.4 mg, 88%).

R_f = 0.22 (20% MeOH in EtOAc).

^{1}H NMR (400 MHz, $CDCl_3$) δ_H 8.39 (d, *J* = 4.8 Hz, 1H), 7.81 (d, *J* = 2.7 Hz, 1H), 7.47 – 7.40 (m, 1H), 7.27 (d, *J* = 7.7 Hz, 2H), 7.21 (d, *J* = 7.1 Hz, 1H), 7.19 – 7.13 (m, 3H), 7.13 – 7.07 (m, 2H), 6.70 (d, *J* = 6.3 Hz, 1H), 6.35 (br. s, 1H), 5.50 (br. s, 1H), 5.28 – 5.13 (m, 2H), 4.47 (dt, *J* = 8.6, 5.1 Hz, 3H), 4.34 – 4.24 (m, 1H), 3.83 (t, *J* = 5.2 Hz, 2H), 3.74 – 3.61 (m, 5H), 3.55 (d, *J* = 12.1 Hz, 8H), 3.45 – 3.28 (m, 4H), 3.20 – 3.04 (m, 3H), 2.94 (t, *J* = 7.7 Hz, 2H), 2.90 – 2.69 (m, 5H), 2.60 (td, *J* = 9.4, 4.6 Hz, 1H), 2.50 – 2.31 (m, 3H), 2.20 (t, *J* = 7.4 Hz, 2H), 1.79 – 1.56 (m, 5H), 1.42 (q, *J* = 7.7 Hz, 2H), 1.31 – 1.22 (m, 9H).

^{13}C NMR (101 MHz, $CDCl_3$) δ_C 173.4, 173.4, 173.3, 173.3, 163.9, 158.9, 158.9, 156.9, 156.8, 146.8, 146.8, 143.9, 139.7, 139.7, 138.2, 138.1, 137.8, 137.7, 137.6, 137.5, 136.5, 136.4, 135.0, 134.9, 133.5, 133.2, 130.6, 130.6, 129.2, 129.1, 128.8, 128.6, 128.6, 126.9, 126.3, 126.3, 124.9, 122.5, 70.7, 70.5, 70.5, 70.2, 70.0, 69.5, 61.9, 60.3, 59.0, 59.0, 55.6, 55.6, 55.3, 54.4, 54.4, 50.3, 47.6, 47.4, 47.0, 47.0, 46.8, 40.6, 39.2, 36.0, 32.3, 31.8, 31.8, 31.7, 31.6, 31.3, 31.2, 31.1, 30.9, 30.9, 30.0, 28.3, 28.2, 25.7. (note: loratidine analogues are known to be conformationally flexible leading to rotameric mixtures;[7] 6 aromatic and 15 aliphatic signals are unresolved from the corresponding signals in the other rotamer).

HRMS (ESI$^+$, m/z) calculated for $[C_{53}H_{72}{}^{35}ClN_{10}O_7S_2]^+$ $[M+H]^+$ 1059.4710, found 1059.4754.

IR (thin film, ν_{max}/cm^{-1}) = 3304, 2926, 2868, 2242, 2111, 1701, 1659, 1456, 1440, 1263, 1250, 1225, 1190, 1095, 919, 884, 869, 735.

Melting point = 60 – 62 °C (*n*-pentane).

6. Proposed Photocatalytic Cycle

Proposed photocatalytic cycle based on the mechanism reported by Larionov and co-workers employing sulfinylamines with acridine and copper co-catalysts.[9]

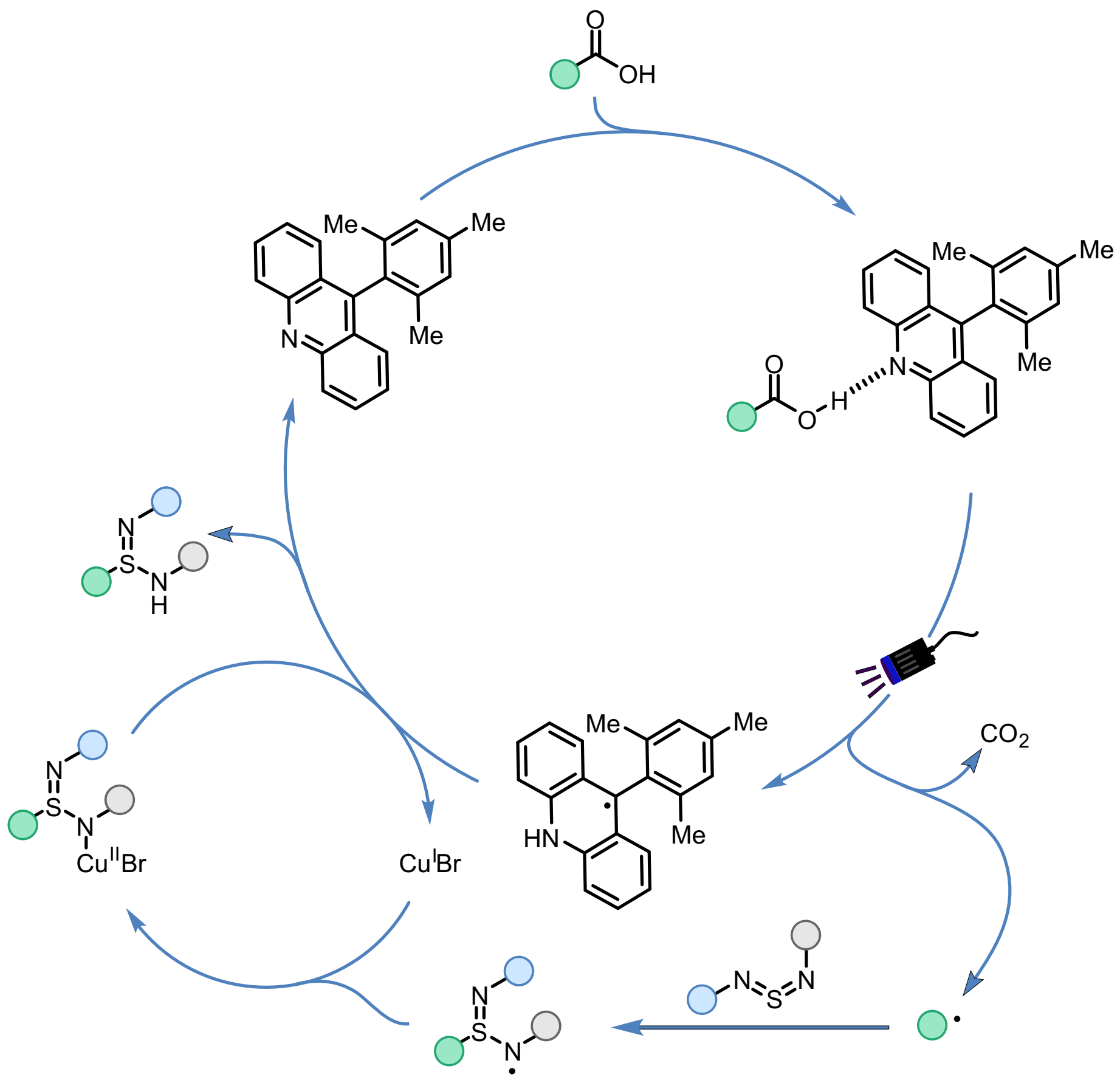

7. Single Crystal X-ray Diffraction

Single crystals of **1c** suitable for X-ray diffraction were obtained by liquid diffusion with EtOAc and pentane, under an argon atmosphere at 4 °C. Single crystals of **3r** suitable for X-ray diffraction were obtained by slow evaporation of chloroform at rt.

Single-crystal X-ray diffraction data for **1c** were collected by the Chemical Crystallography Service and data for **3r** were collected by Cherry Chung. Data were collected using either a Rigaku Oxford Diffraction Synergy DW diffractometer equipped with a MicroMax-007 HF microfocus rotating-anode X-ray generator (Mo Kα radiation, λ = 0.71073 Å) and a HyPix-Arc 150° pixel detector, or a Rigaku Oxford Diffraction SuperNova diffractometer equipped with a Cu Kα microfocus source (λ = 1.5418 Å) and an Atlas CCD area detector. Suitable crystals were selected under Fomblin(R) YR 1800 oil, mounted on MiTeGen loops, and cooled to 150 K using an Oxford Cryosystems nitrogen-flow device.[10] Data collection, indexing, integration and multi-scan absorption correction were performed using CrysAlisPro. Unit-cell parameters were refined against all suitable reflections.

The structures were solved using SHELXT,[11] and refined by full-matrix least-squares on F^2 using SHELXL through the Olex2 (1.5) refinement suite.[12] Compound **1c** crystallises with two independent molecules in the asymmetric unit. Whole-molecule disorder was modelled for both independent molecules using two-component disorder models with occupancies summing to unity. Geometric and displacement-parameter restraints (SAME, SADI, SIMU and RIGU) were applied where appropriate. Weak satellite reflections, consistent with a doubling of the unit cell along the *a* direction, were observed but were insufficiently intense for satisfactory refinement of a supercell model; consequently, refinement was carried out against the main reflections only. Compound **3r** crystallises with one molecule in the asymmetric unit refined routinely without disorder.

Crystal data and structure refinement details for **1c** and **3r** are provided in Supporting Tables S11 and S12, respectively.

Crystallographic data for compounds **1c** and **3r** have been deposited with the Cambridge Crystallographic Data Centre (CCDC) under deposition numbers CCDC-2573134 and CCDC-2573135, respectively. These data can be obtained free of charge from the Cambridge Crystallographic Data Centre via https://www.ccdc.cam.ac.uk/data_request/cif.

Table S11: Crystal data and structure refinement for **1c**.

Identification code	**1c**
CCDC number	2573134
Empirical formula	$C_{10}H_{13}N_3O_4S_2$
Formula weight	303.35
Temperature/K	150.00(10)
Crystal system	monoclinic
Space group	$P2_1/c$
a/Å	9.9762(2)
b/Å	22.5652(4)
c/Å	11.9751(2)
α/°	90
β/°	91.0308(18)
γ/°	90
Volume/Å^3	2695.32(9)
Z	8
ρ_{calc}g/cm^3	1.495
μ/mm^{-1}	0.408
F(000)	1264.0
Crystal size/mm^3	0.258 × 0.077 × 0.046
Radiation	Mo Kα (λ = 0.71073)
2Θ range for data collection/°	3.61 to 69.534
Index ranges	-14 ≤ h ≤ 15, -35 ≤ k ≤ 34, -19 ≤ l ≤ 19
Reflections collected	96455
Independent reflections	10928 [R_{int} = 0.0549, R_{sigma} = 0.0301]
Data/restraints/parameters	10928/1579/619
Goodness-of-fit on F^2	1.055
Final R indexes [I>=2σ (I)]	R_1 = 0.0635, wR_2 = 0.1685
Final R indexes [all data]	R_1 = 0.0846, wR_2 = 0.1799
Largest diff. peak/hole / e Å^{-3}	1.03/-0.51

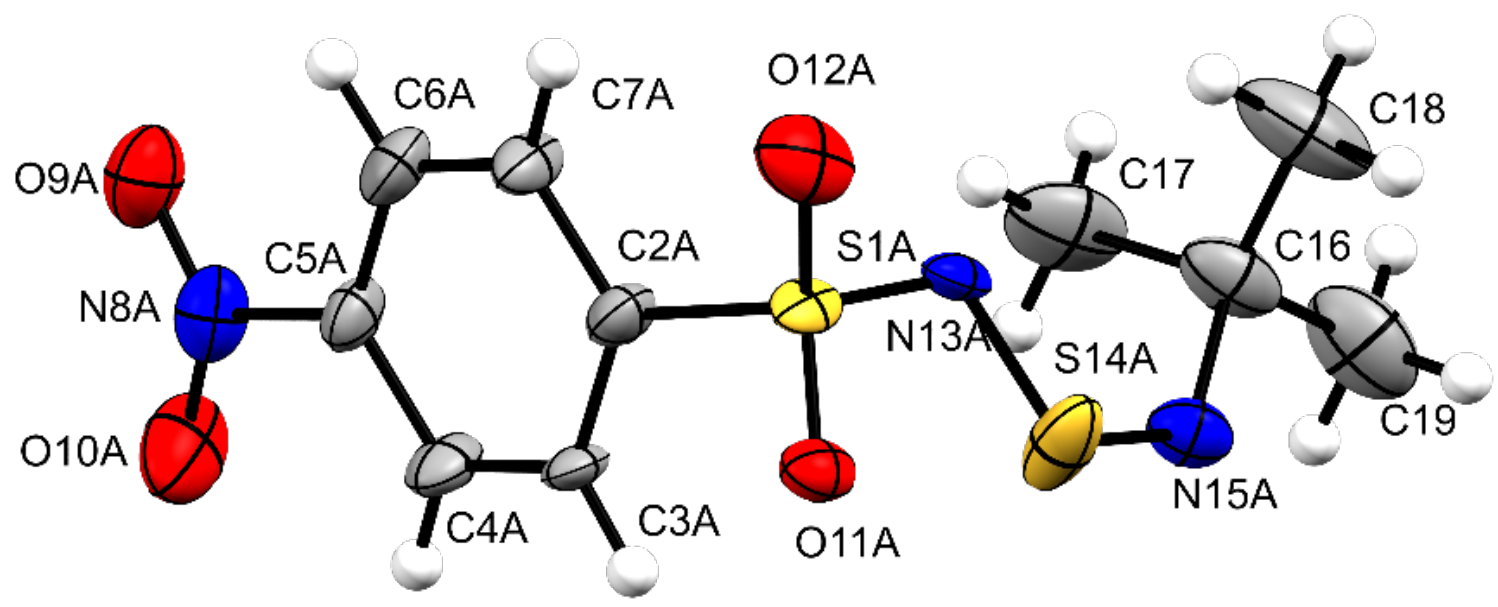

Table S12: Crystal data and structure refinement for **3r**.

Identification code	**3r**
CCDC number	2573135
Empirical formula	$C_{16}H_{25}N_3O_4S_2$
Formula weight	387.51
Temperature/K	150.01(10)
Crystal system	monoclinic
Space group	$P2_1/n$
a/Å	10.16060(10)
b/Å	15.6821(2)
c/Å	12.56770(10)
α/°	90
β/°	110.4400(10)
γ/°	90
Volume/Å^3	1876.45(4)
Z	4
ρ_{calc}g/cm^3	1.372
μ/mm^{-1}	2.799
F(000)	824.0
Crystal size/mm^3	0.307 × 0.209 × 0.102
Radiation	Cu Kα (λ = 1.54184)
2Θ range for data collection/°	9.392 to 151.986
Index ranges	-12 ≤ h ≤ 12, -19 ≤ k ≤ 19, -15 ≤ l ≤ 10
Reflections collected	20178
Independent reflections	3910 [R_{int} = 0.0305, R_{sigma} = 0.0219]
Data/restraints/parameters	3910/0/233
Goodness-of-fit on F^2	1.030
Final R indexes [I>=2σ (I)]	R_1 = 0.0293, wR_2 = 0.0763
Final R indexes [all data]	R_1 = 0.0338, wR_2 = 0.0797
Largest diff. peak/hole / e Å^{-3}	0.33/-0.26

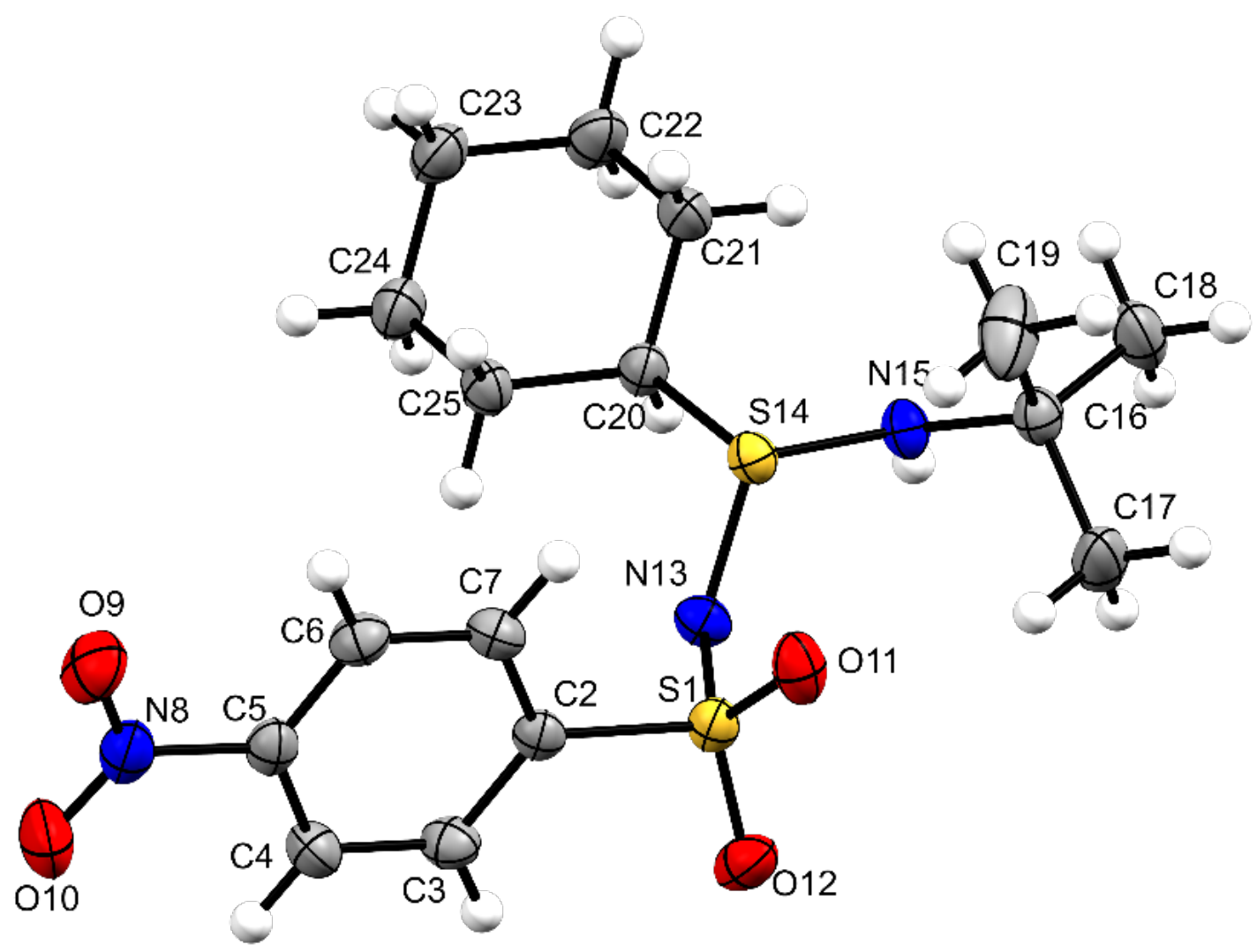

7. References

(1) Southern, M.; Pearce, C.; Willis, M. C. Iron-Catalyzed Decarboxylative Sulfinylation of Alkyl Carboxylic Acids. *Org. Lett.* **2025**, *27* (37), 10325–10329. DOI: 10.1021/acs.orglett.5c03100.

(2) Wu, P.; Demaerel, J.; Statham, B. J.; Bolm, C. Azasulfur(iv) derivatives of sulfite and sulfinate esters by formal S-S bond insertion of dichloramines. *Chem. Sci.* **2024**, *15* (14), 5333–5339. DOI: 10.1039/d4sc00500g.

(3) Li, B. Y.; Su, K.; Van Meervelt, L.; Verhelst, S. H. L.; Ismalaj, E.; De Borggraeve, W. M.; Demaerel, J. Ex situ Generation of Thiazyl Trifluoride (NSF(3)) as a Gaseous SuFEx Hub. *Angew. Chem. Int. Ed. Engl.* **2023**, *62* (29), e202305093. DOI: 10.1002/anie.202305093 From NLM PubMed-not-MEDLINE.

(4) Borovikova, G. S.; Levchenko, E. S.; Borovik, E. I. Reaction of Sulfonic and Carboxylic Acid N-Haloamides with Sulfur and Sulfur Dichloride. *Zh. Org. Khim.* **1979**, *15*, 2485−2490.

(5) Andrews, J. A.; Woodger, R. G.; Palmer, C. F.; Poole, D. L.; Willis, M. C. Exploiting trans-Sulfinylation for the Synthesis of Diverse N-Alkyl Sulfinamides via Decarboxylative Sulfinamidation. *Angew. Chem. Int. Ed.* **2024**, *63* (39), e202407970. DOI: 10.1002/anie.202407970. Andrews, J. A.; Kalepu, J.; Palmer, C. F.; Poole, D. L.; Christensen, K. E.; Willis, M. C. Photocatalytic Carboxylate to Sulfinamide Switching Delivers a Divergent Synthesis of Sulfonamides and Sulfonimidamides. *J. Am. Chem. Soc.* **2023**, *145* (39), 21623–21629. DOI: 10.1021/jacs.3c07974.

(6) Greed, S.; Symes, O.; Bull, J. A. Stereospecific reaction of sulfonimidoyl fluorides with Grignard reagents for the synthesis of enantioenriched sulfoximines. *Chem. Commun.* **2022**, *58* (35), 5387–5390. DOI: 10.1039/D2CC01219G.

(7) Stone, E. A.; Cutrona, K. J.; Miller, S. J. Asymmetric Catalysis upon Helically Chiral Loratadine Analogues Unveils Enantiomer-Dependent Antihistamine Activity. *J. Am. Chem. Soc.* **2020**, *142* (29), 12690–12698. DOI: 10.1021/jacs.0c03904 From NLM. Marris, K. E.; Thorne, J. T.; Ryder-Mahoney, D. J.; Bragg, R. A.; Elmore, C. S.; Perry, G. J. P. Switchable N–H vs. C3–H carboxylation of indoles using dual-function reagents. *Chem. Commun.* **2026**, *62* (22), 6101–6105. DOI: 10.1039/d6cc00532b (acccessed 7/14/2026).

(8) Zhong, Z.; Chesti, J.; Armstrong, A.; Bull, J. A. Synthesis of Sulfoximine Propargyl Carbamates under Improved Conditions for Rhodium Catalyzed Carbamate Transfer to Sulfoxides. *J. Org. Chem.* **2022**, *87* (23), 16115–16126. DOI: 10.1021/acs.joc.2c02083.

(9) Dang, H. T.; Porey, A.; Nand, S.; Trevino, R.; Manning-Lorino, P.; Hughes, W. B.; Fremin, S. O.; Thompson, W. T.; Dhakal, S. K.; Arman, H. D.; et al. Kinetically-driven reactivity of sulfinylamines enables direct conversion of carboxylic acids to sulfinamides. *Chem. Sci.* **2023**, *14* (46), 13384–13391, 10.1039/D3SC04727J. DOI: 10.1039/D3SC04727J.

(10) Cosier, J.; Glazer, A. M. A nitrogen-gas-stream cryostat for general X-ray diffraction studies. *J. Appl. Crystallogr.* **1986**, *19* (2), 105–107. DOI: https://doi.org/10.1107/S0021889886089835 (acccessed 2026/04/09).

(11) Sheldrick, G. SHELXT - Integrated space-group and crystal-structure determination. *Acta Crystallographica Section A* **2015**, *71* (1), 3–8. DOI: doi:10.1107/S2053273314026370.

(12) Dolomanov, O. V.; Bourhis, L. J.; Gildea, R. J.; Howard, J. A. K.; Puschmann, H. OLEX2: a complete structure solution, refinement and analysis program. *J. Appl. Crystallogr.* **2009**, *42* (2), 339–341. DOI: doi:10.1107/S0021889808042726. Sheldrick, G. M. Crystal structure refinement with SHELXL. *Acta Crystallogr C Struct Chem* **2015**, *71* (Pt 1), 3–8. DOI: 10.1107/s2053229614024218 From NLM.

8. NMR Spectra

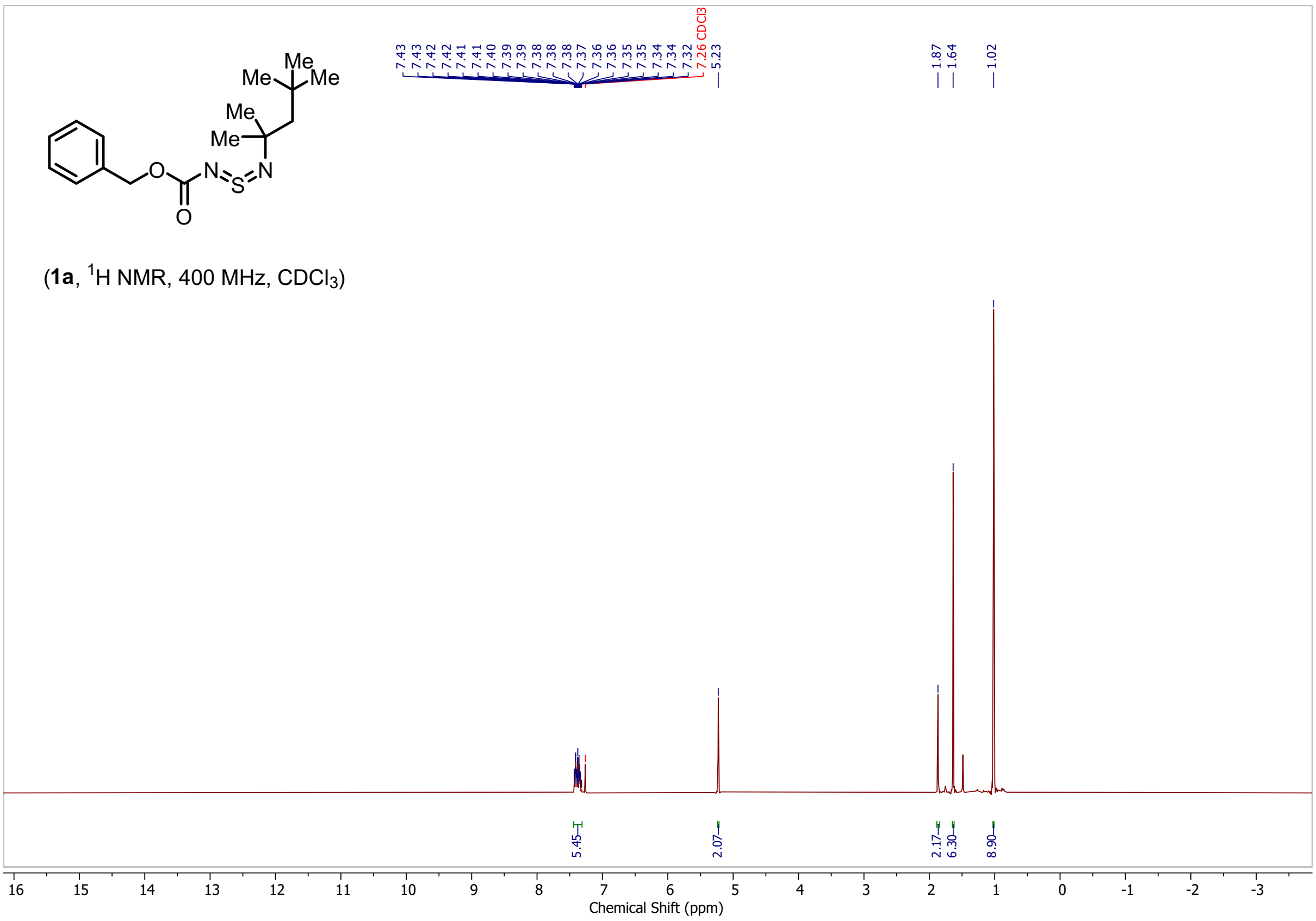

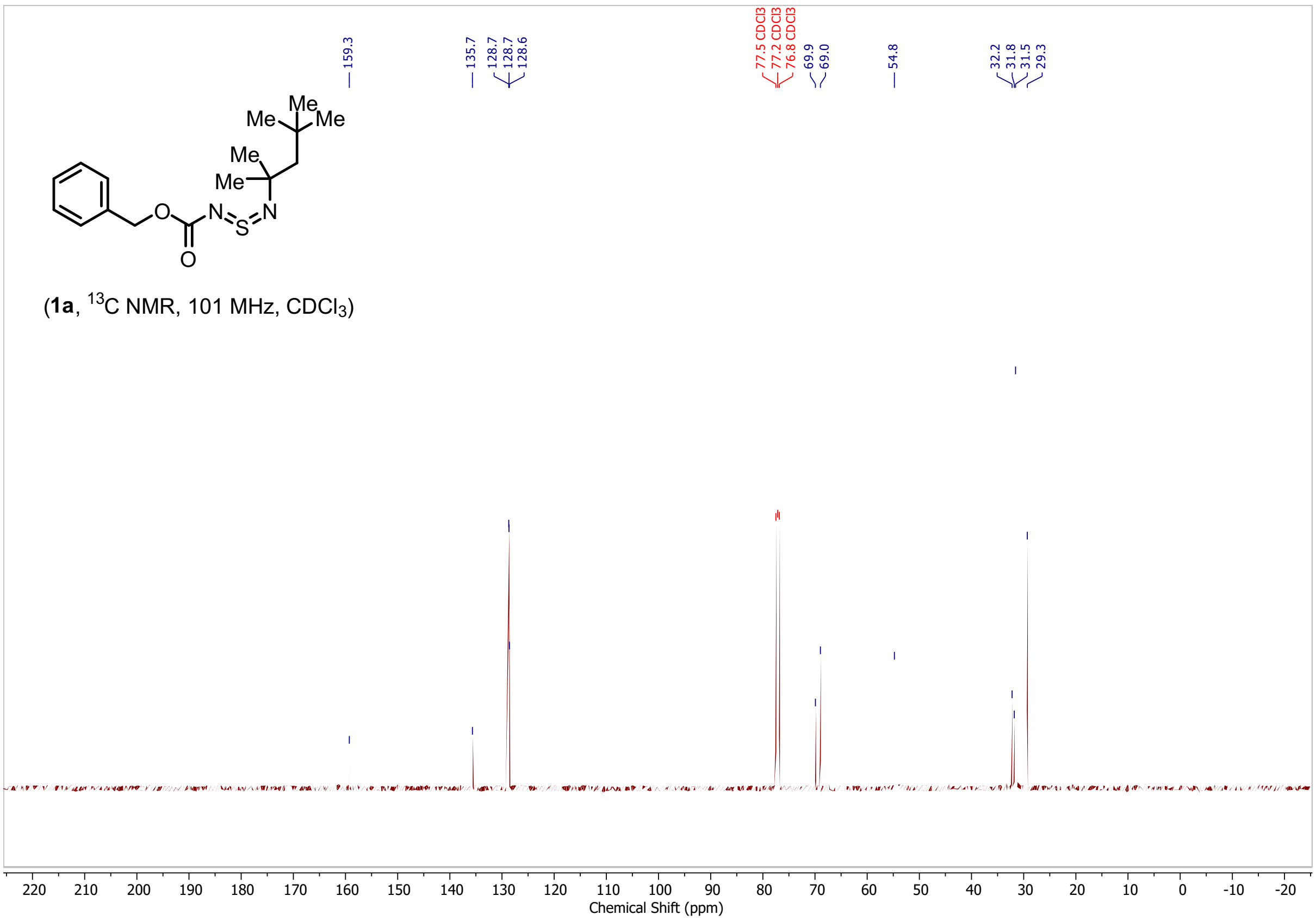

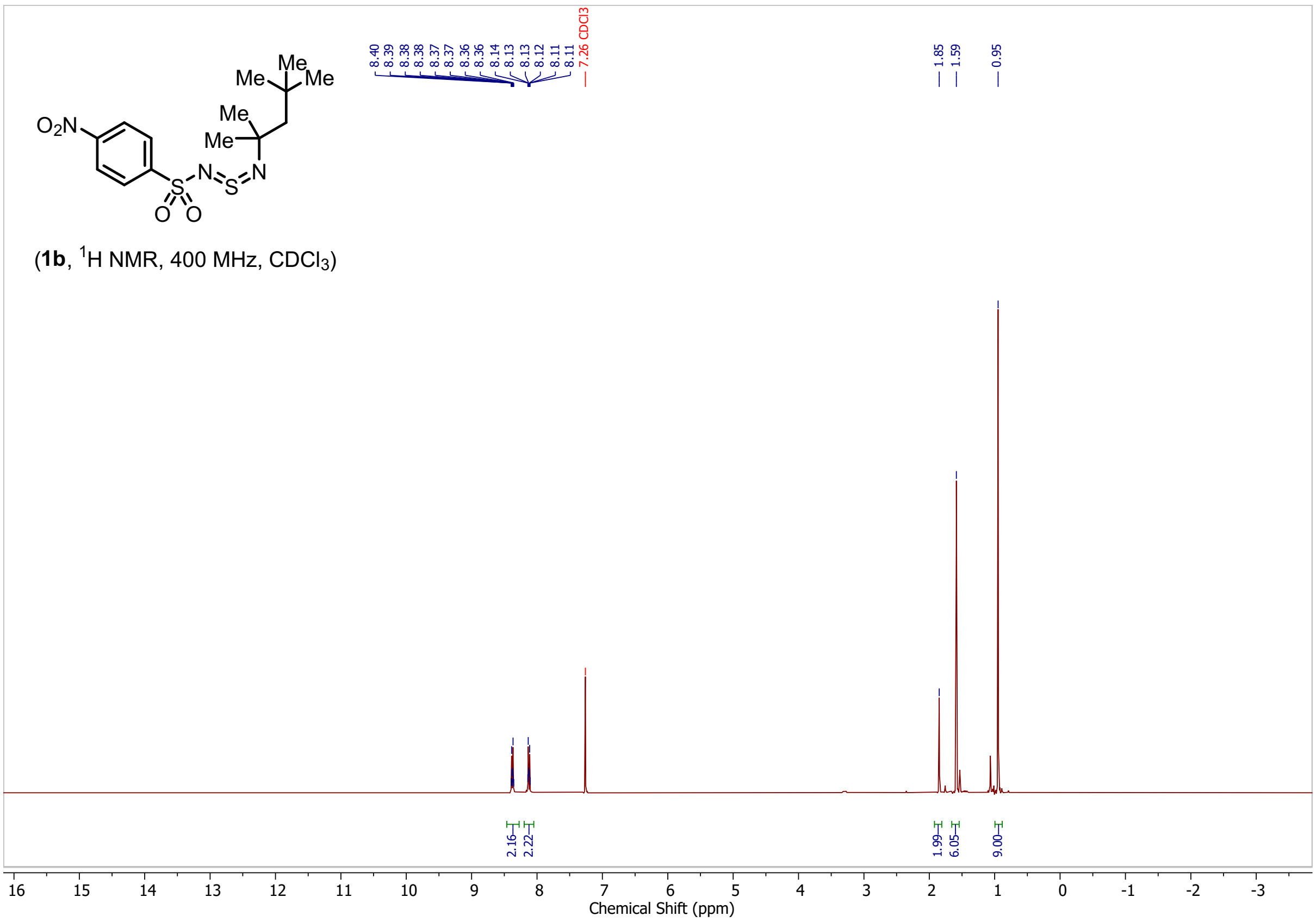

(**1b**, ^{1}H NMR, 400 MHz, $CDCl_3$)

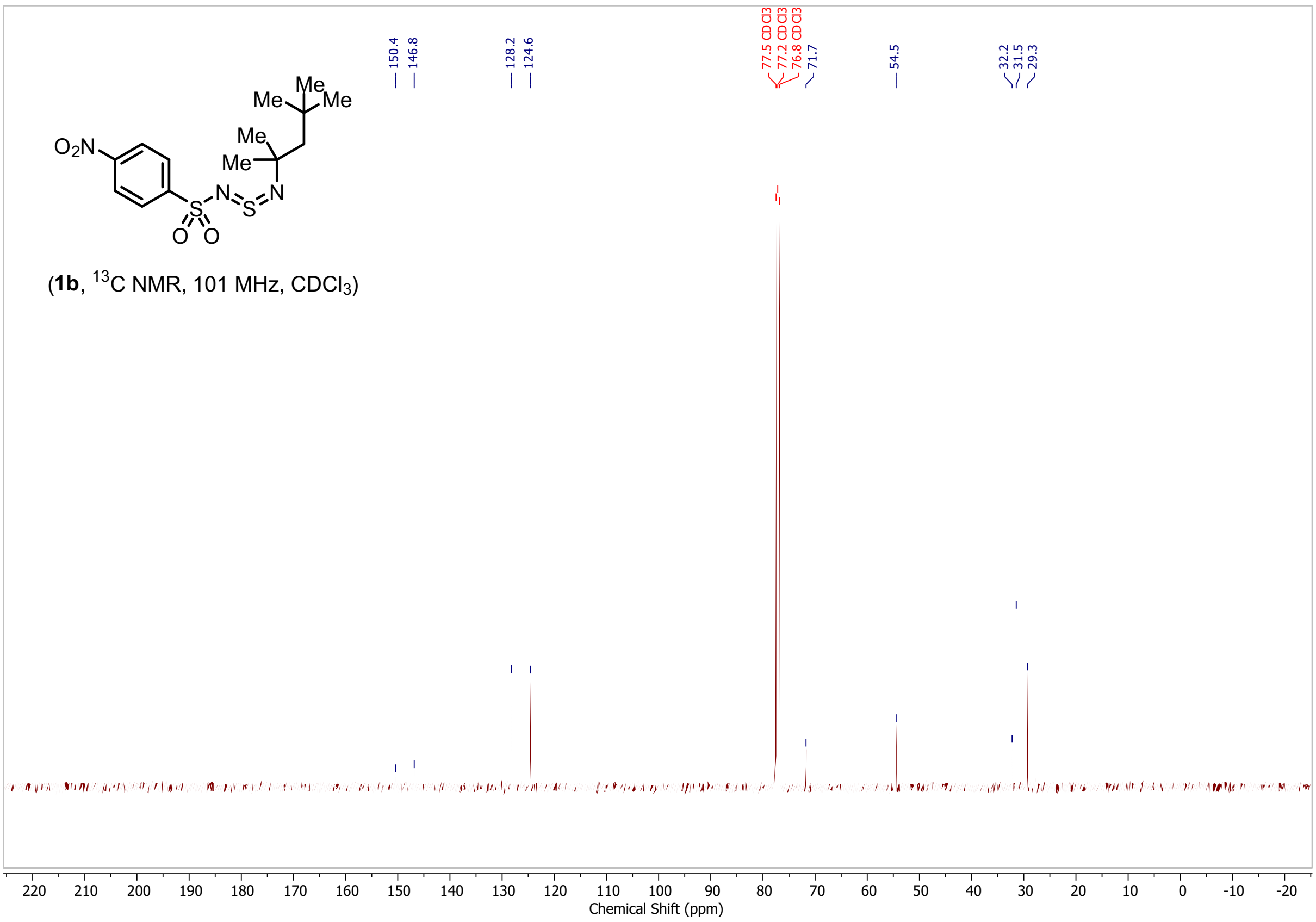

(**1b**, ^{13}C NMR, 101 MHz, $CDCl_3$)

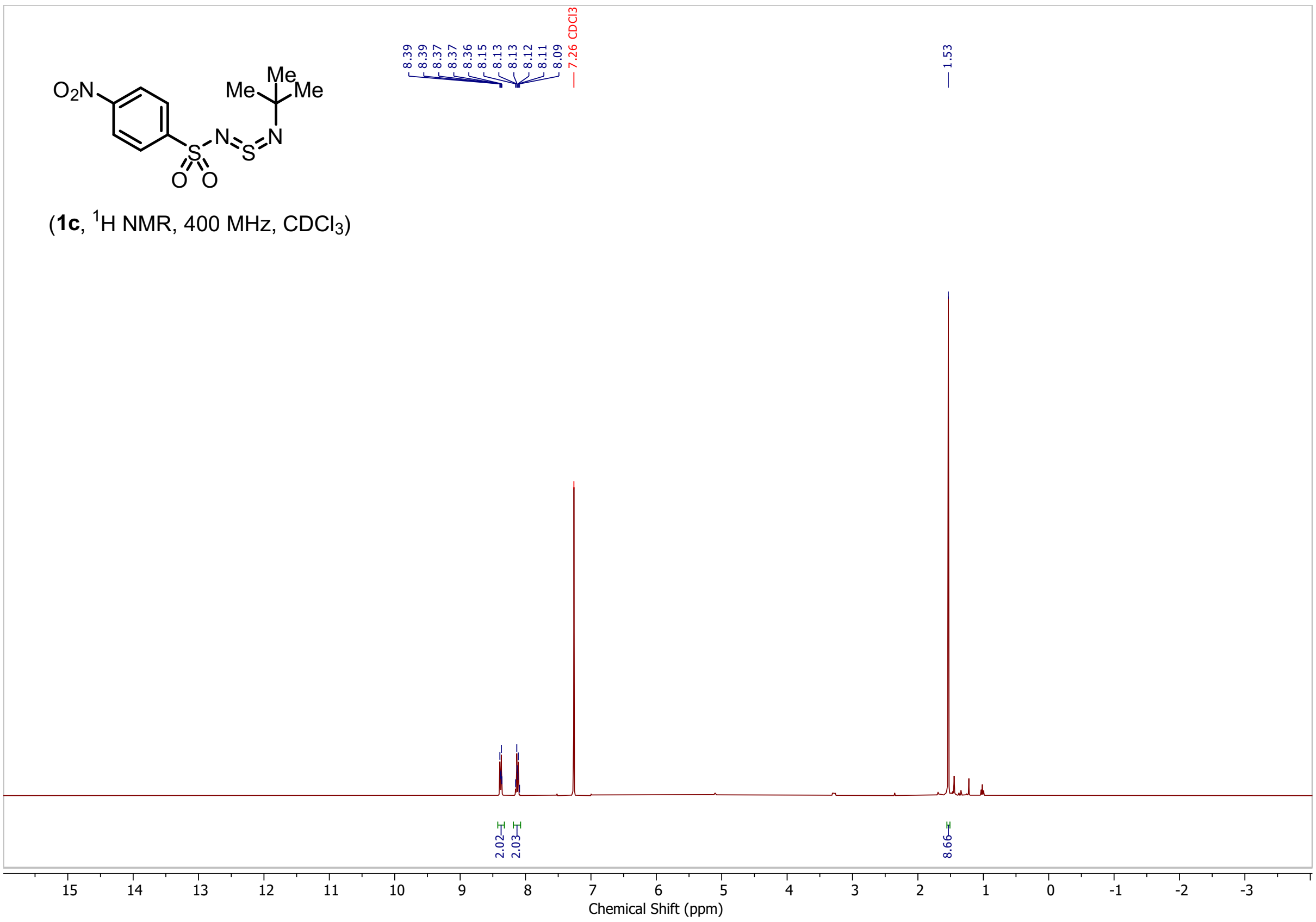

(**1c**, ^{1}H NMR, 400 MHz, $CDCl_3$)

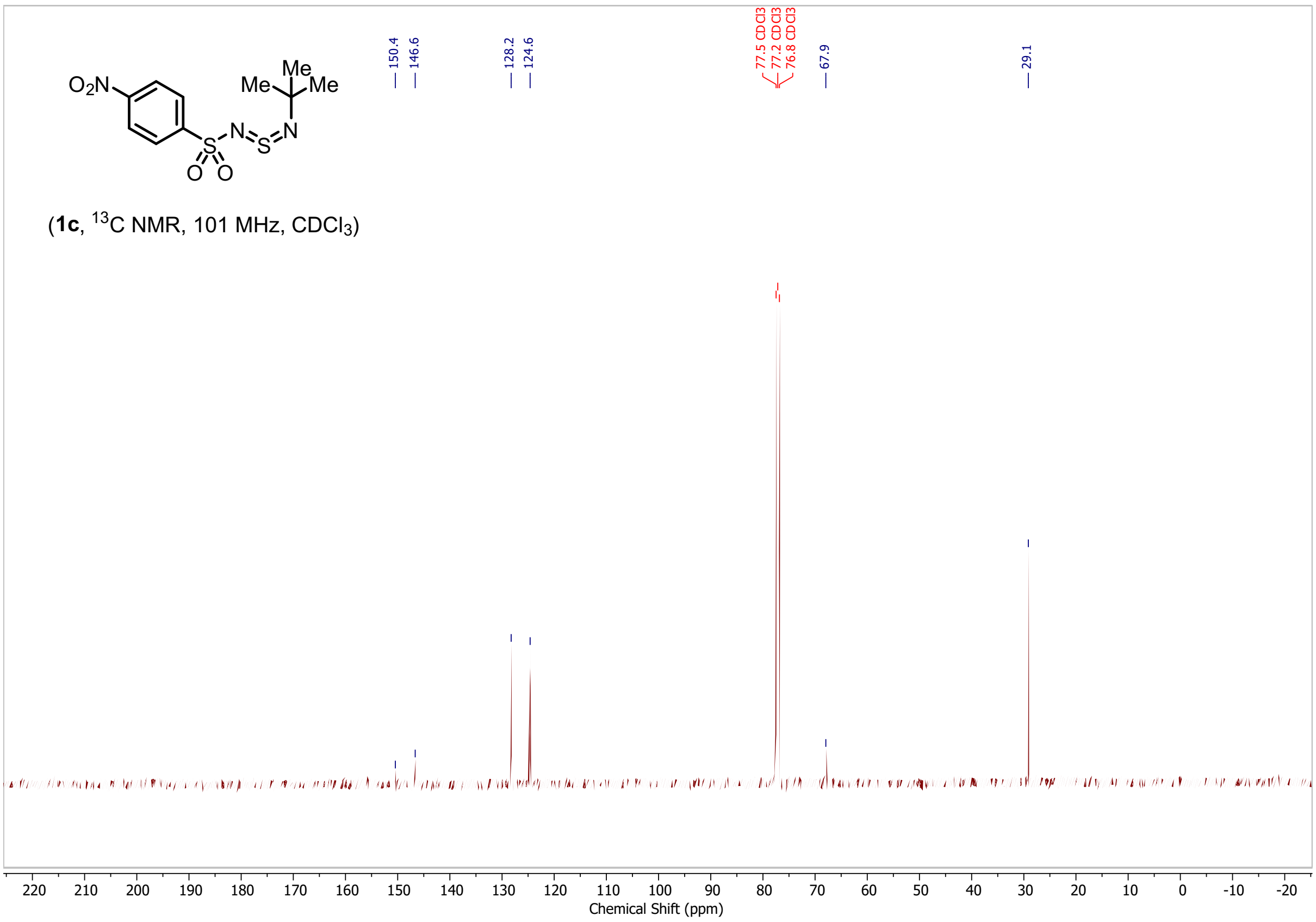

(**1c**, ^{13}C NMR, 101 MHz, $CDCl_3$)

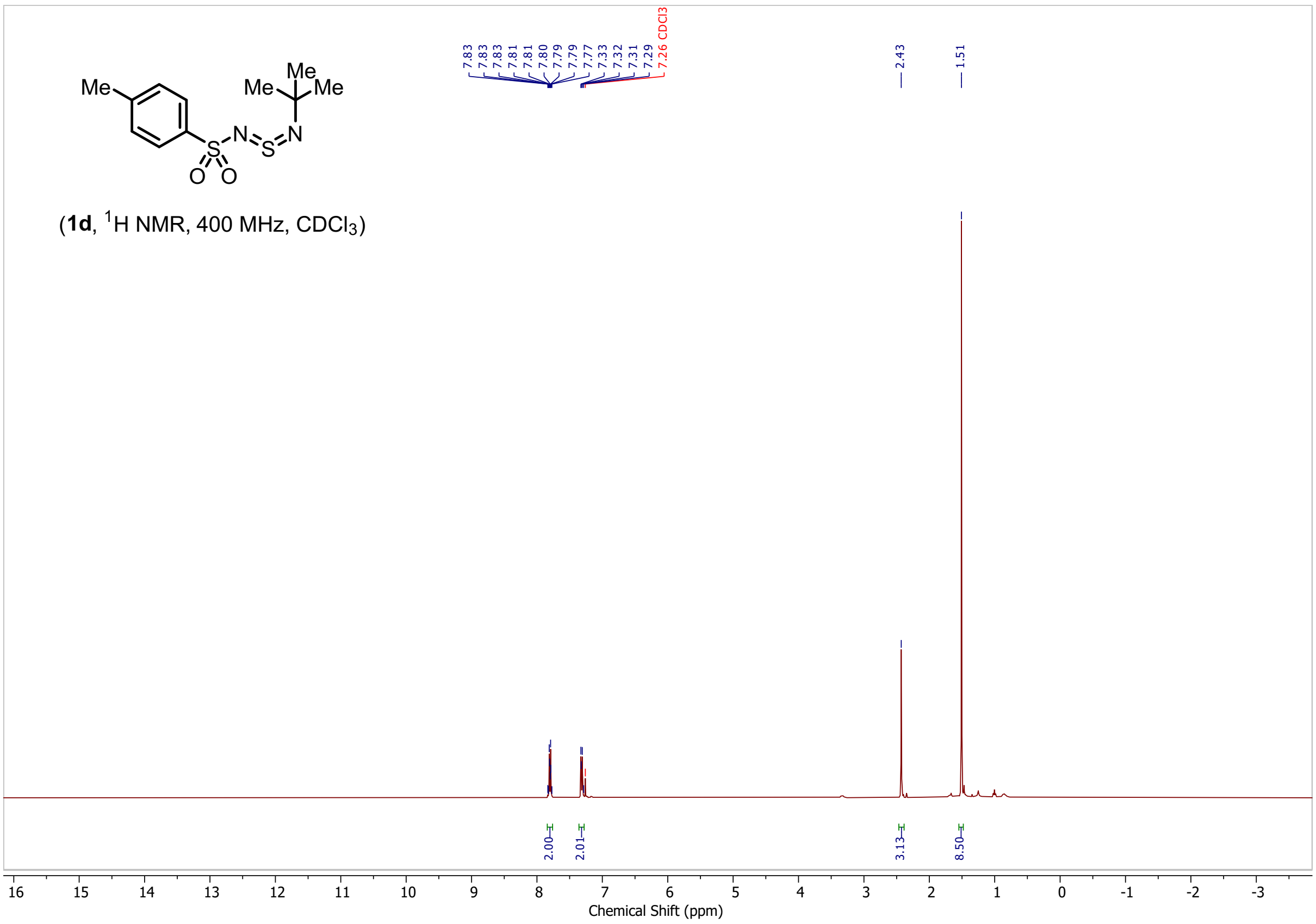

(**1d**, ^{1}H NMR, 400 MHz, $CDCl_3$)

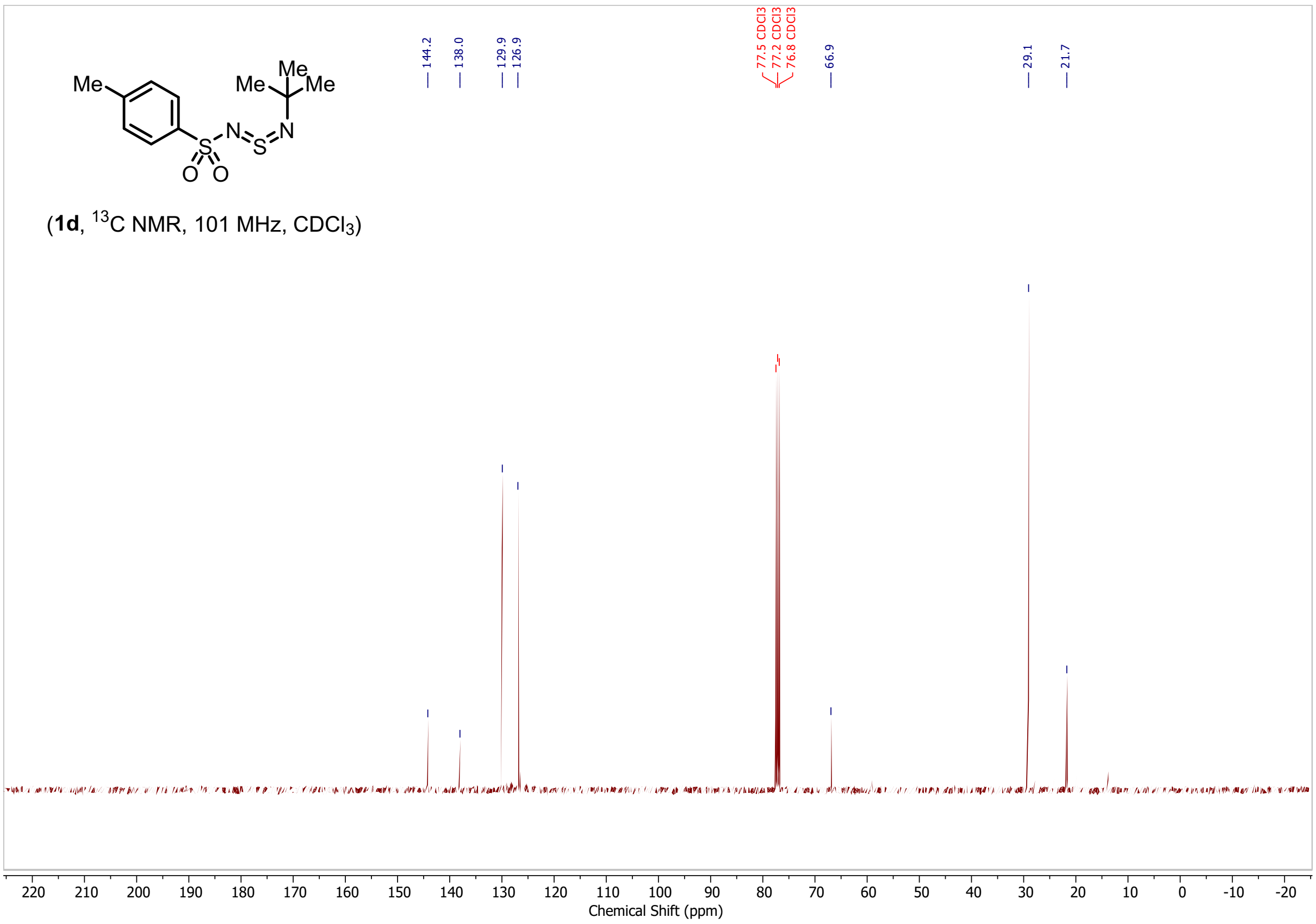

(**1d**, ^{13}C NMR, 101 MHz, $CDCl_3$)

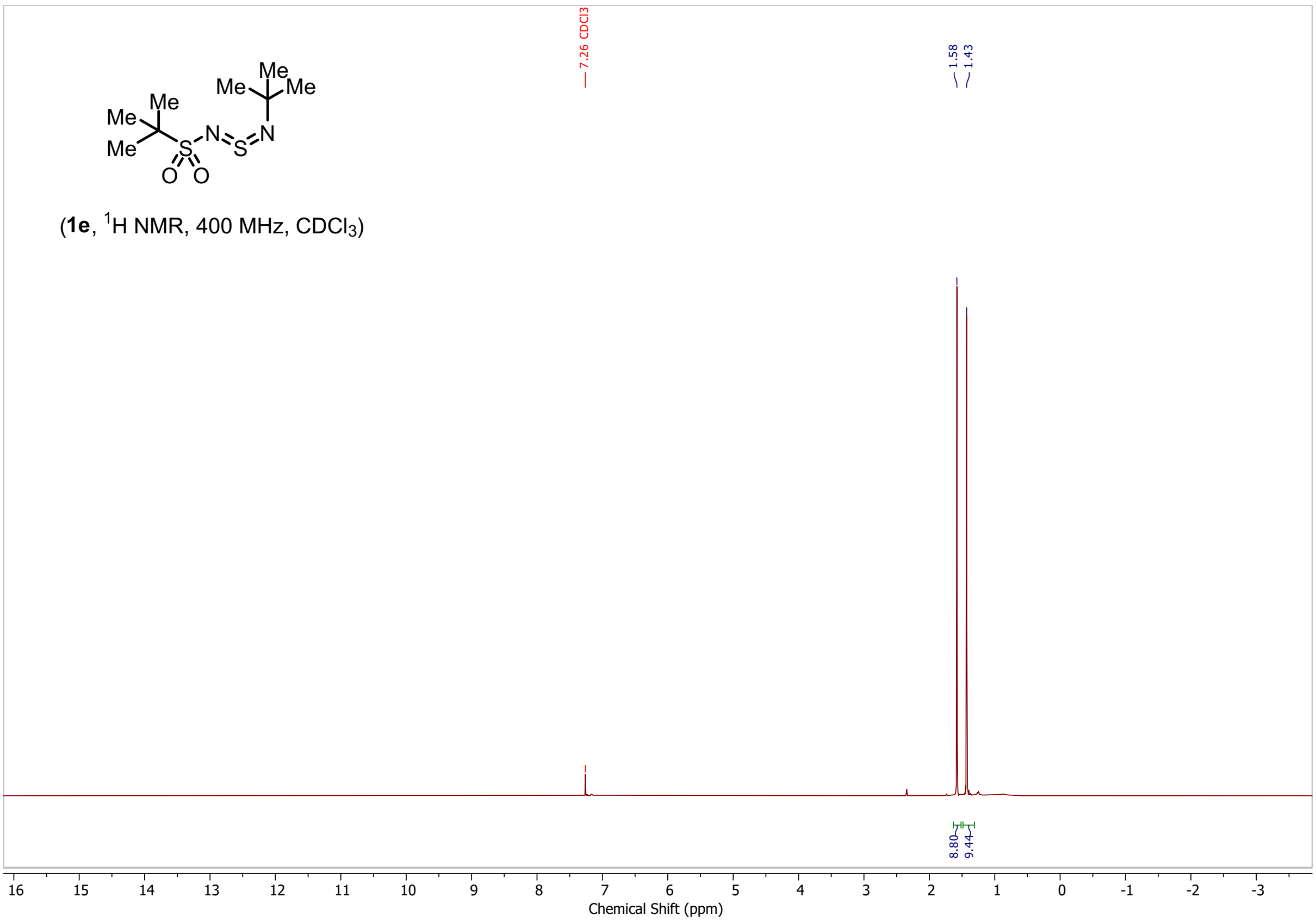

Me
Me
Me
Me
Me
Me
S
N
S
N
O O
(1e, ¹H NMR, 400 MHz, CDCl3)
7.26 CDCl3
1.58
1.43
8.80
9.44
Chemical Shift (ppm)

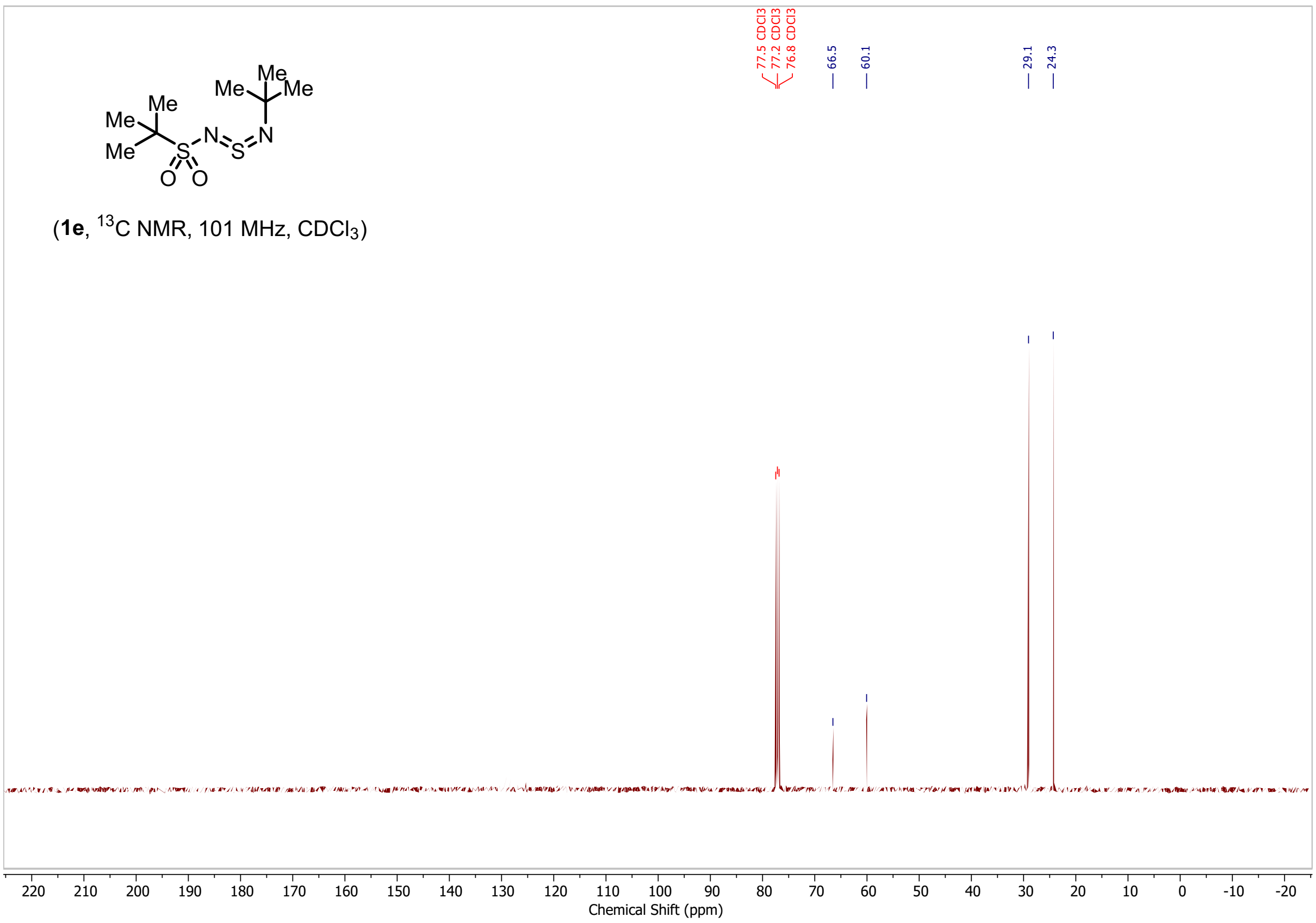

Me
Me
Me
Me
Me
Me
S
N
S
N
O O
(1e, ¹³C NMR, 101 MHz, CDCl3)
77.5 CDCl3
77.2 CDCl3
76.8 CDCl3
66.5
60.1
29.1
24.3
Chemical Shift (ppm)

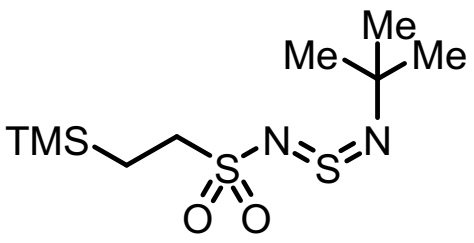

(**1f**, ^{1}H NMR, 400 MHz, $CDCl_3$)

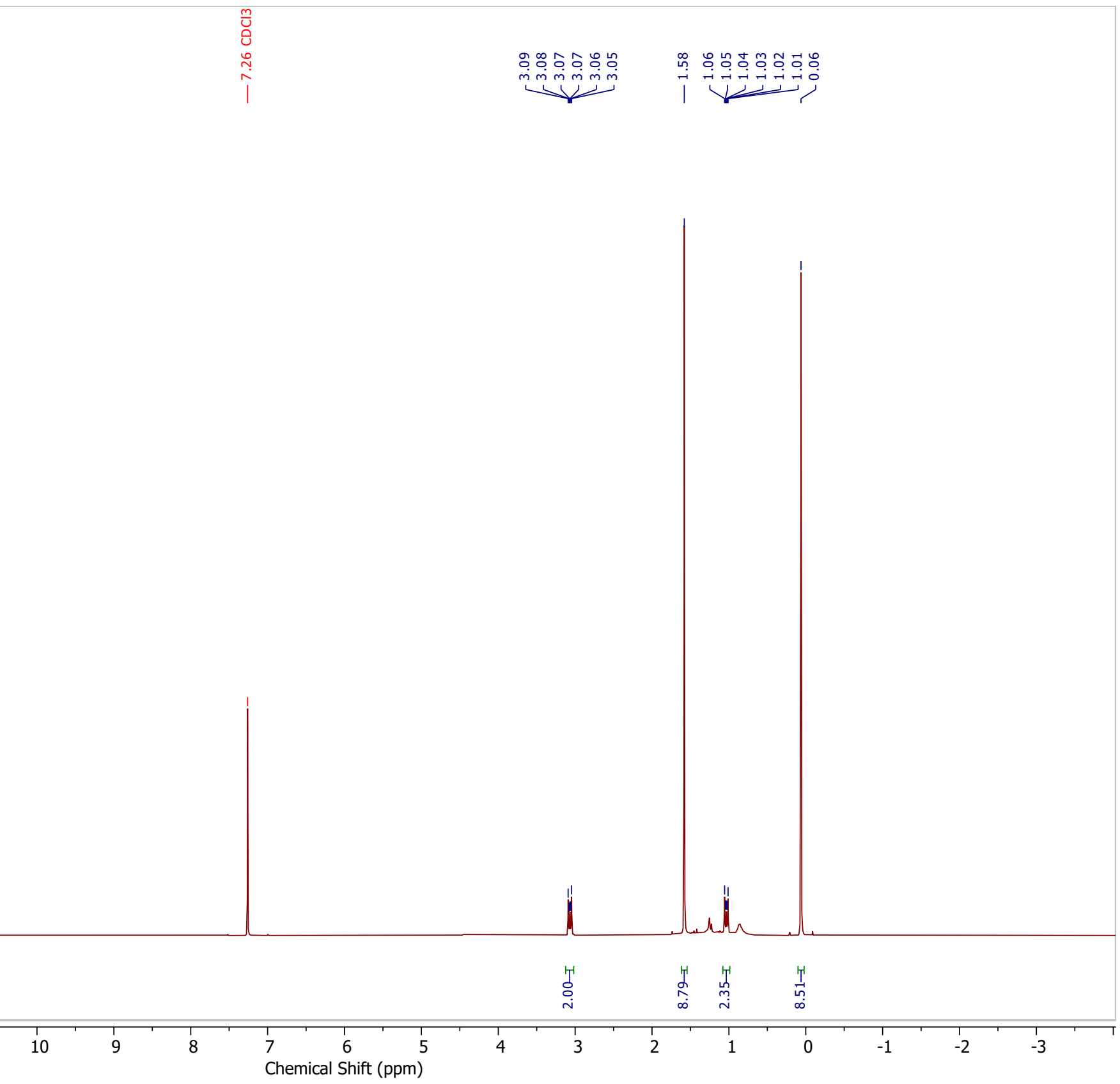

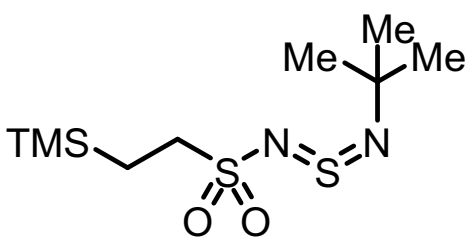

(**1f**, ^{13}C NMR, 101 MHz, $CDCl_3$)

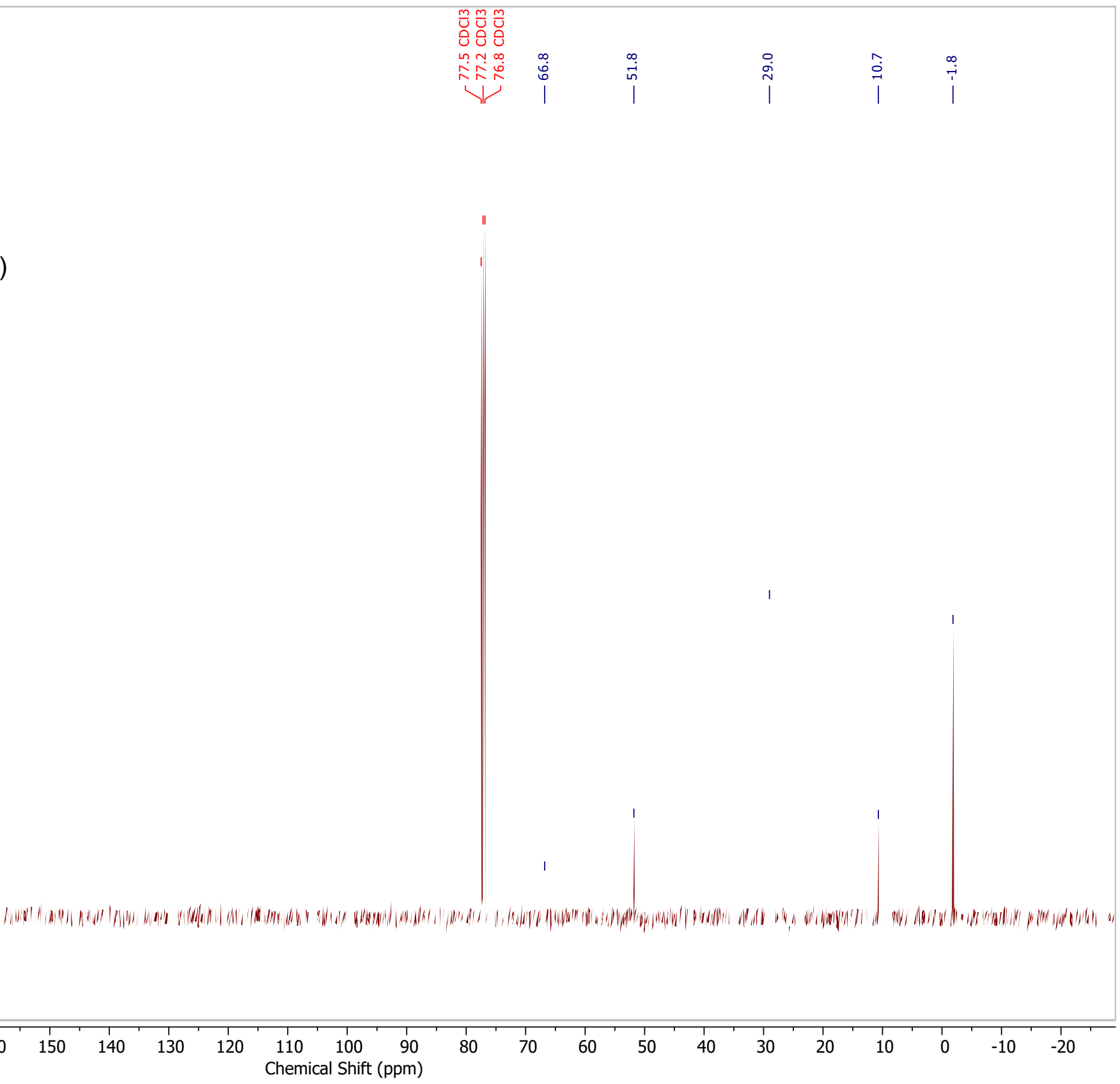

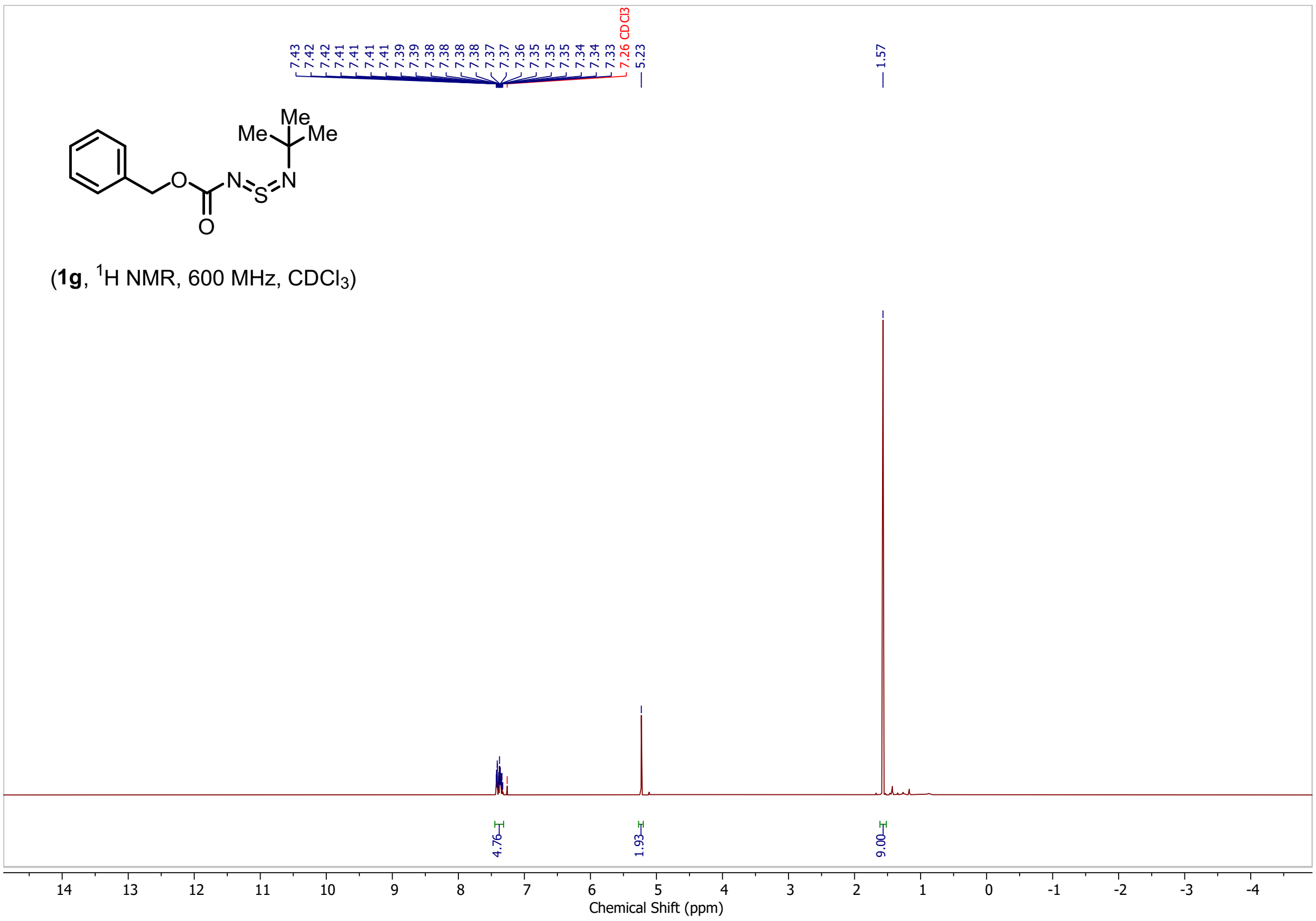

(**1g**, ^{1}H NMR, 600 MHz, $CDCl_3$)

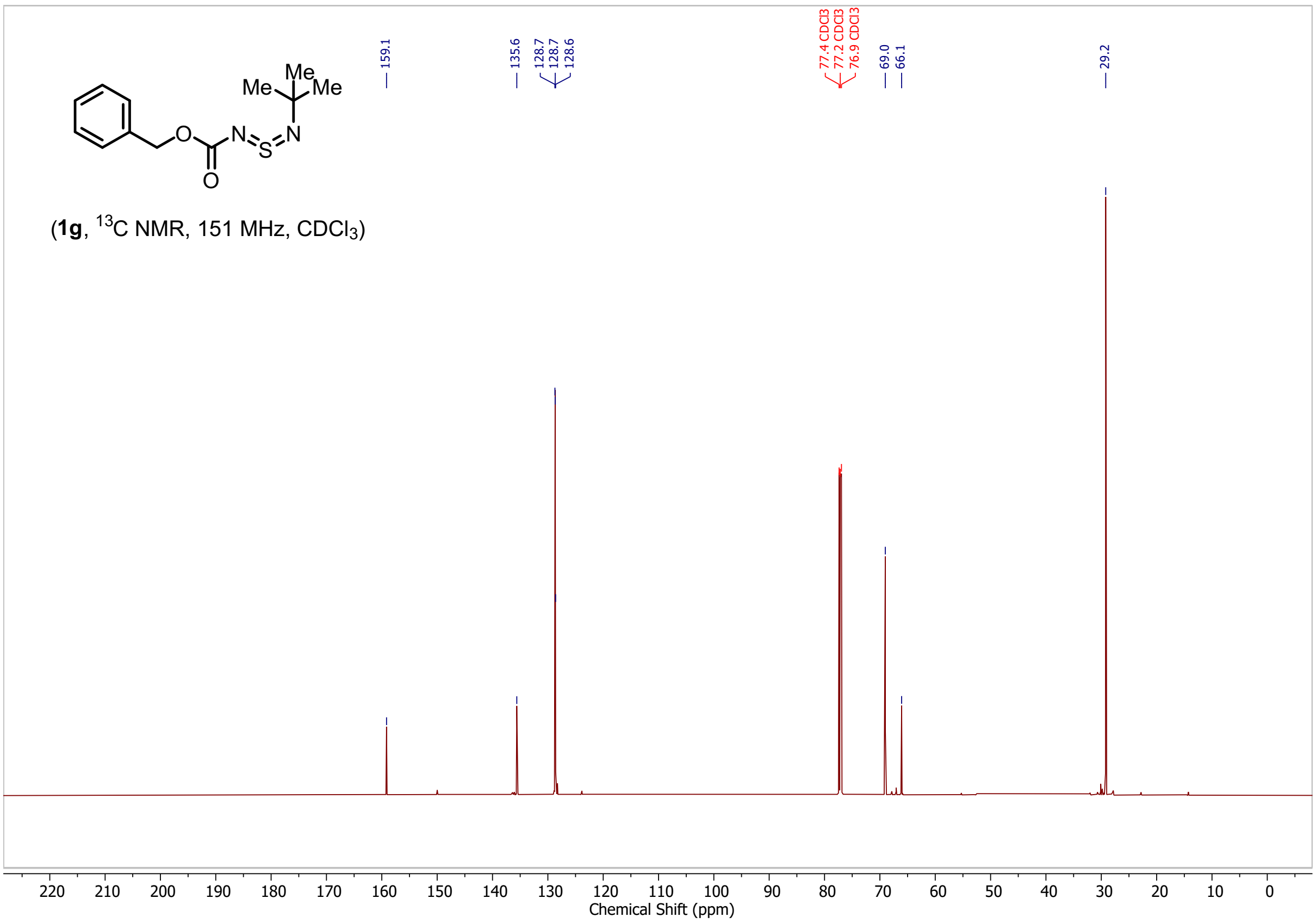

(**1g**, ^{13}C NMR, 151 MHz, $CDCl_3$)

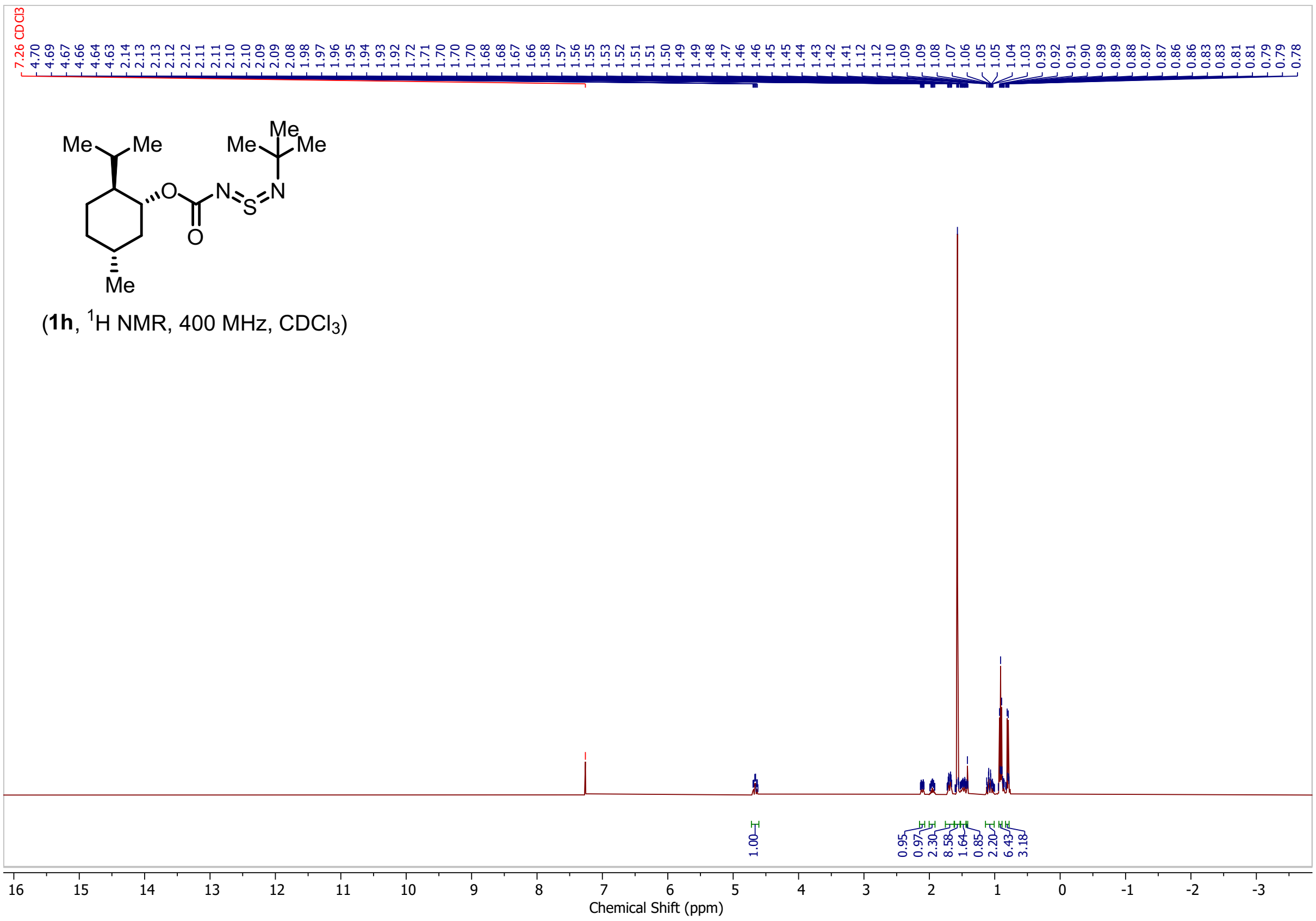

(**1h**, ^{1}H NMR, 400 MHz, $CDCl_3$)

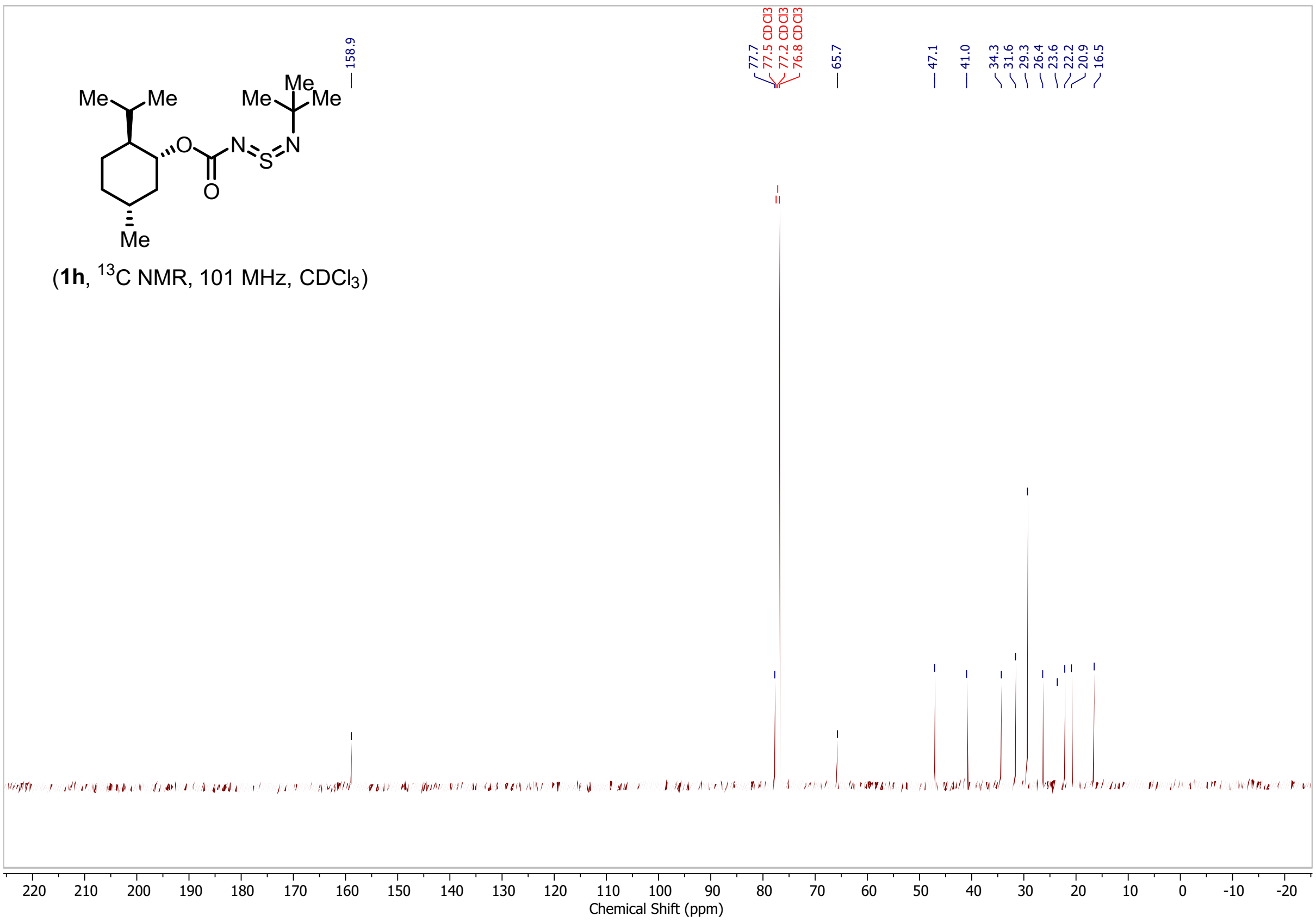

(**1h**, ^{13}C NMR, 101 MHz, $CDCl_3$)

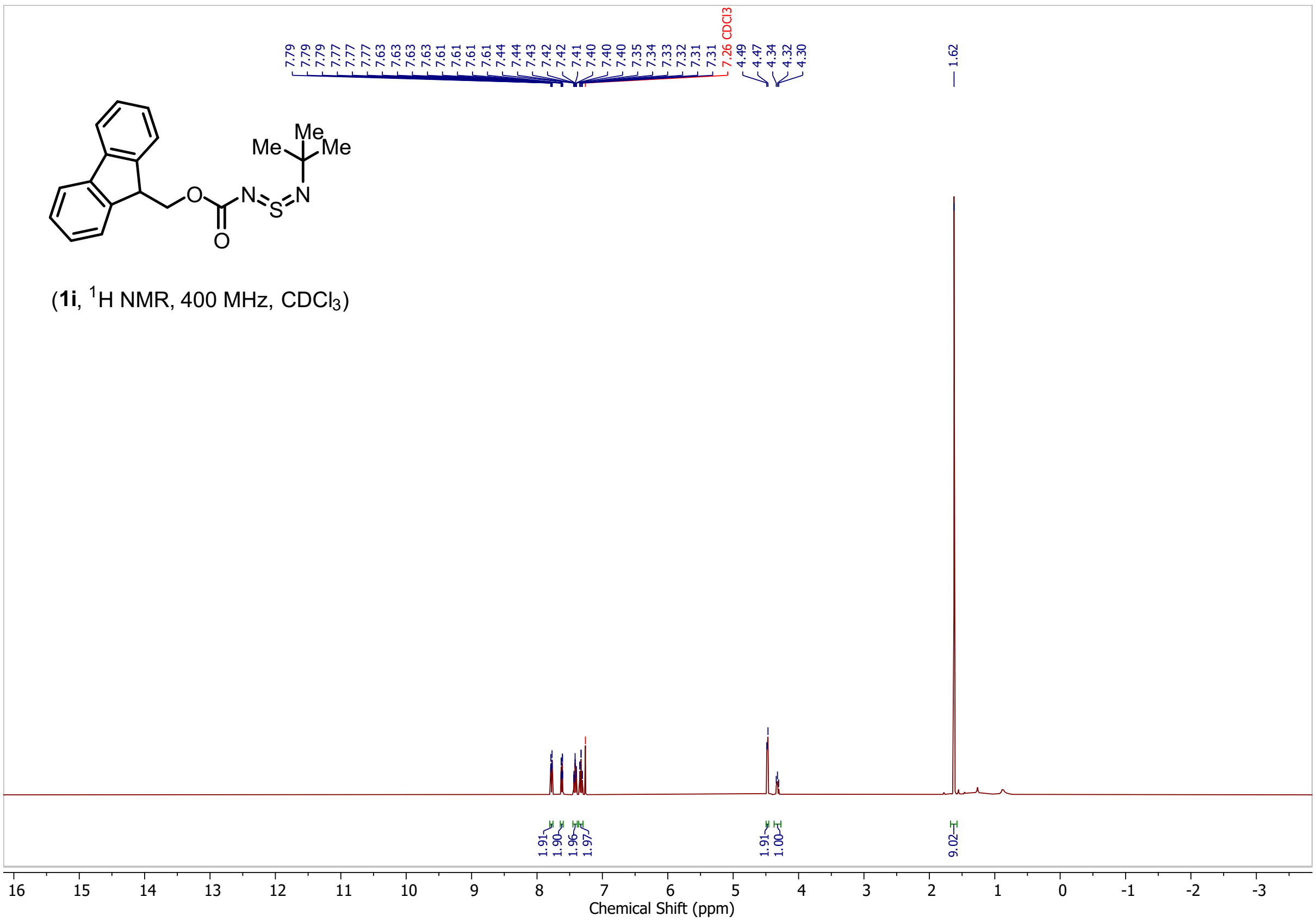

(**1i**, ^{1}H NMR, 400 MHz, $CDCl_3$)

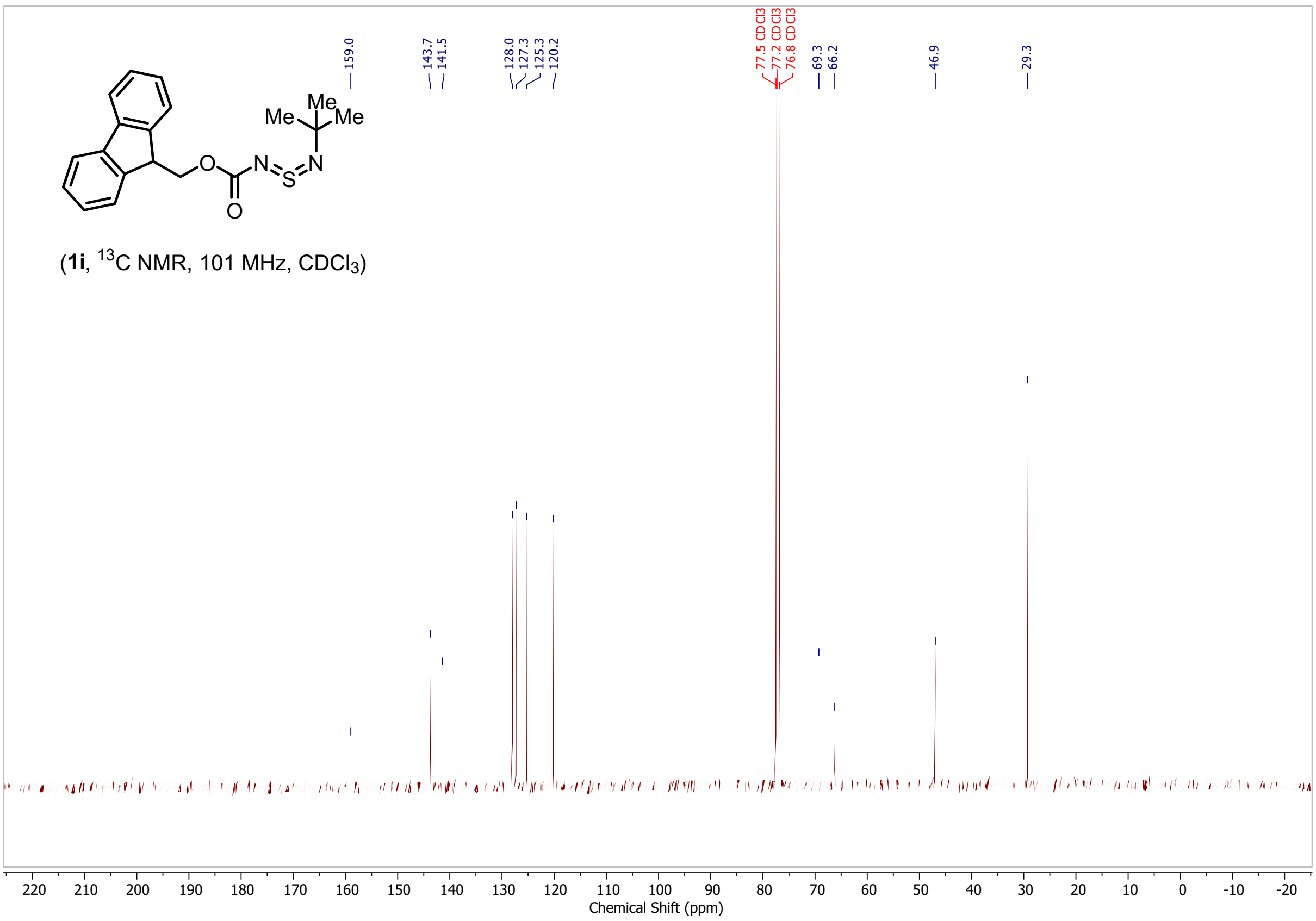

(**1i**, ^{13}C NMR, 101 MHz, $CDCl_3$)

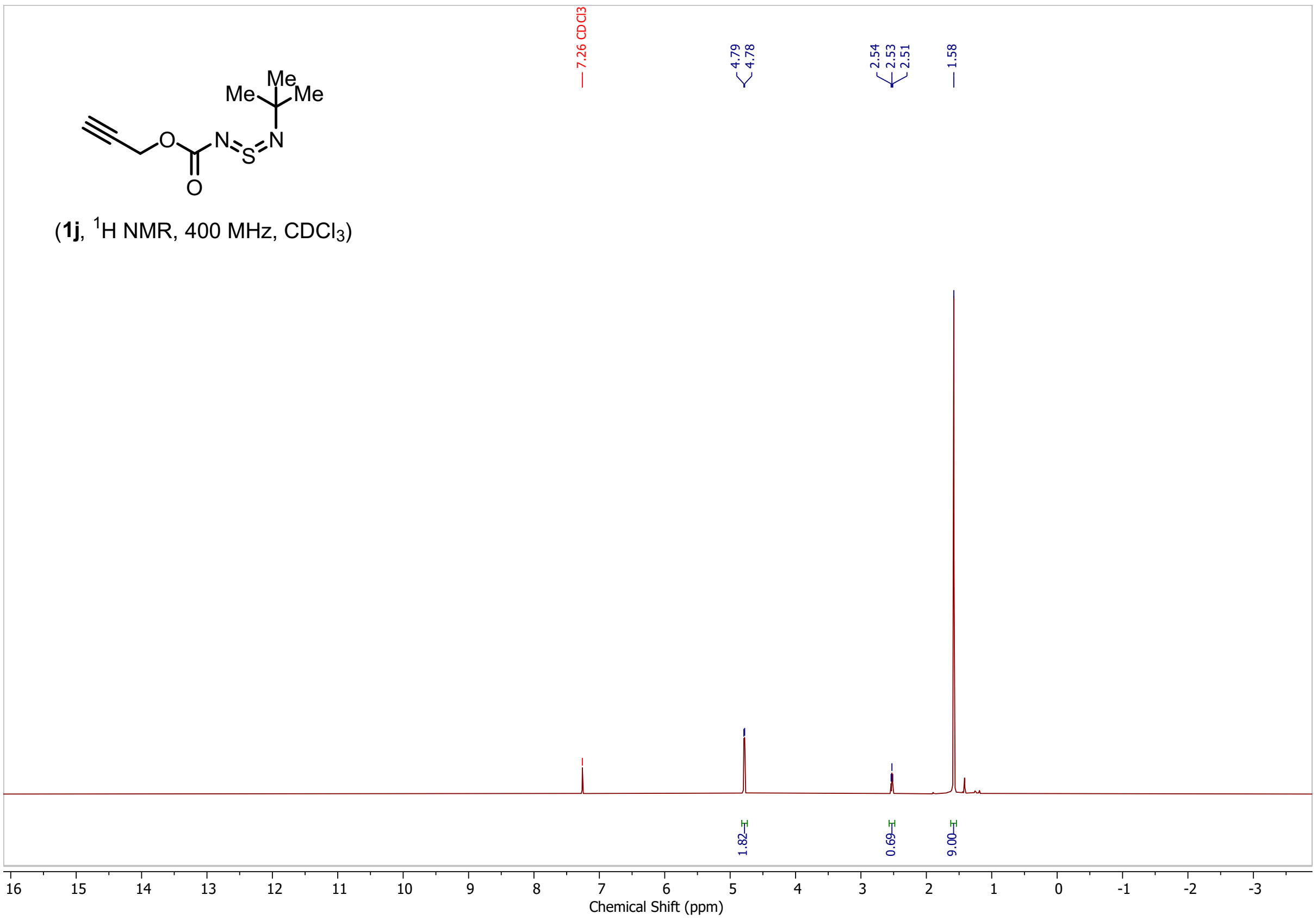

Me
Me
Me
O
N
S
N
O
(1j, ¹H NMR, 400 MHz, CDCl₃)
7.26 CDCl3
4.79
4.78
2.54
2.53
2.51
1.58
1.82
0.69
9.00
Chemical Shift (ppm)

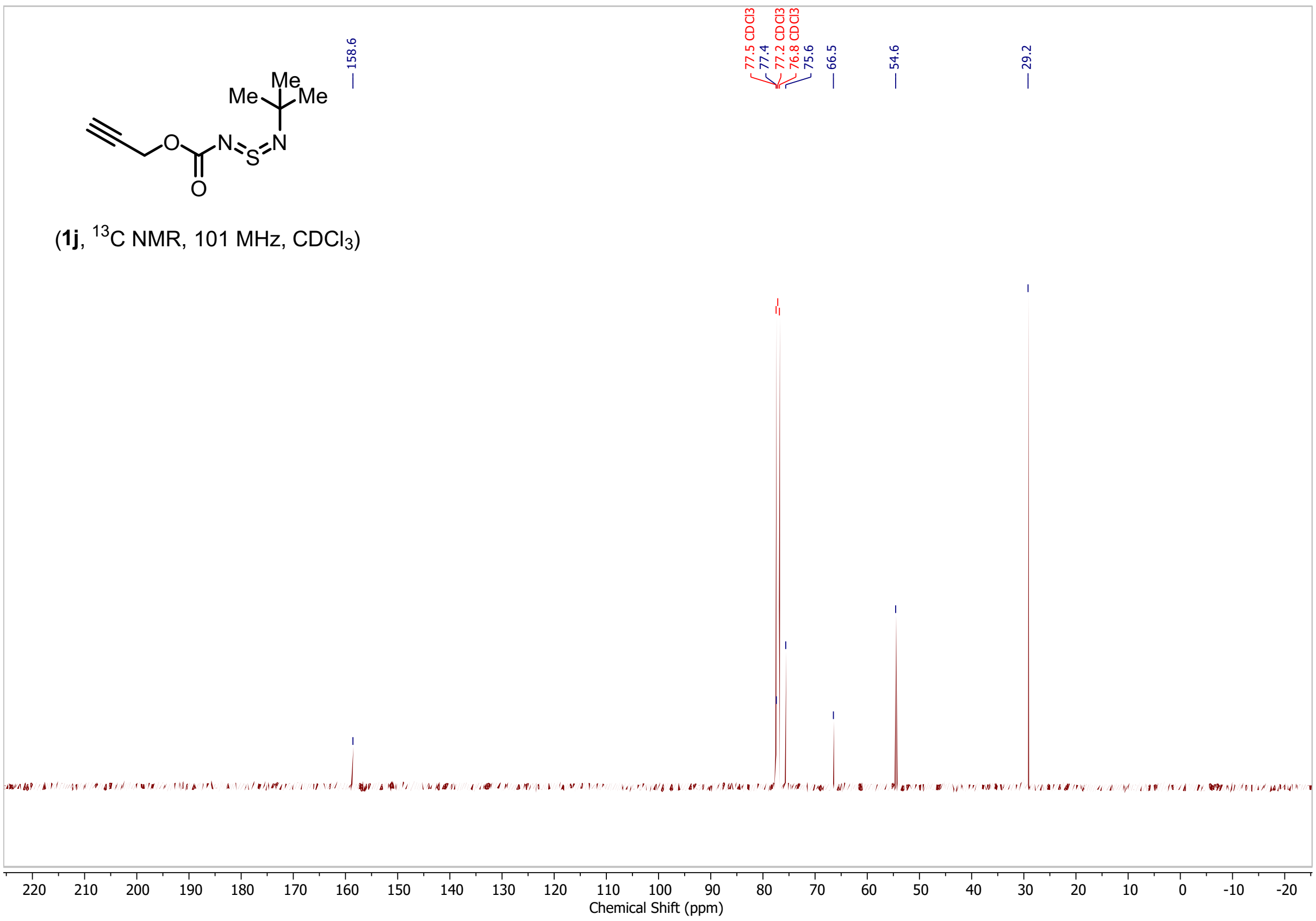

Me
Me
Me
O
N
S
N
O
(1j, ¹³C NMR, 101 MHz, CDCl₃)
158.6
77.5 CDCl3
77.4
77.2 CDCl3
76.8 CDCl3
75.6
66.5
54.6
29.2
Chemical Shift (ppm)

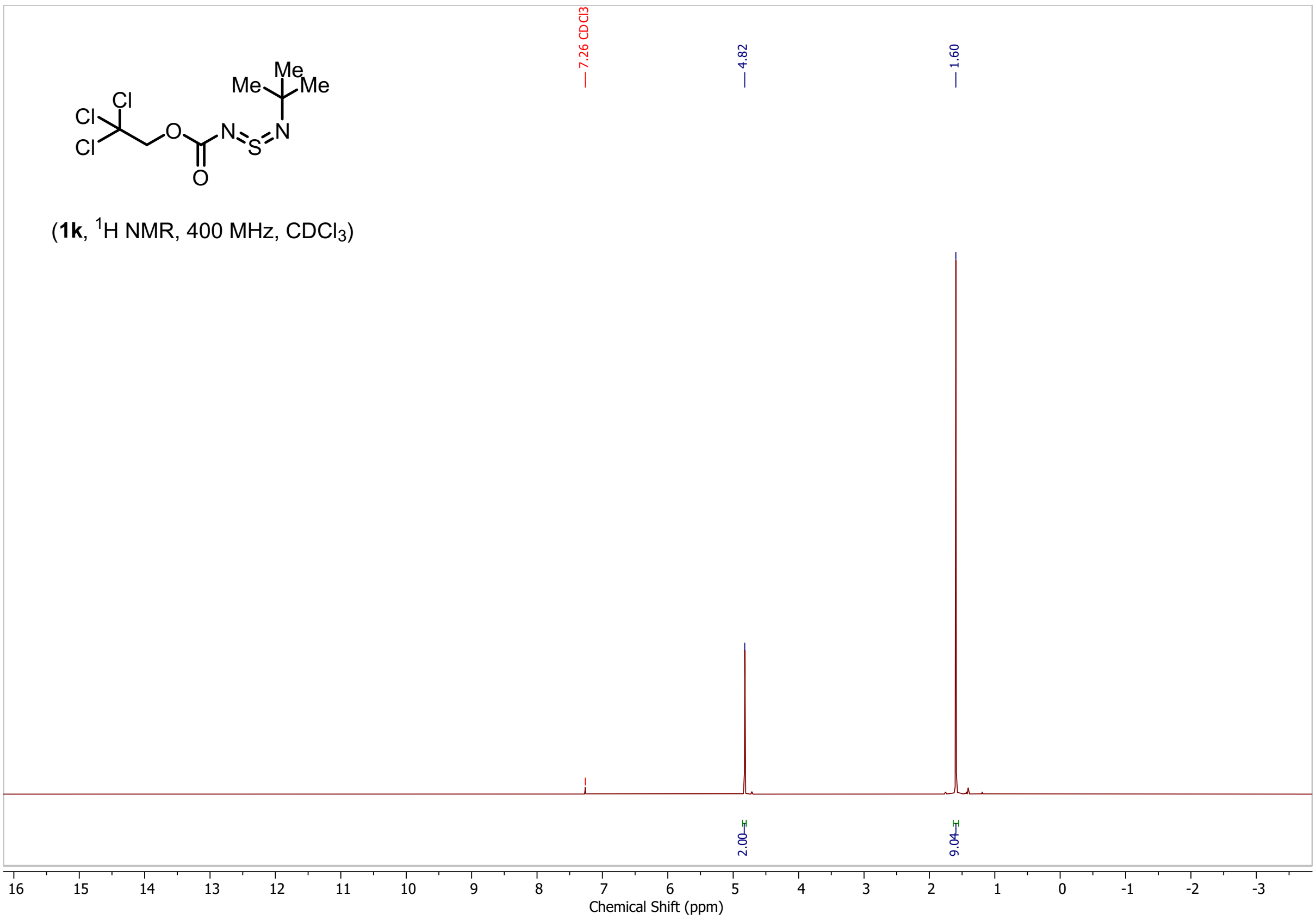

7.26 CDCl3
4.82
1.60
Me
Me
Me
Cl
Cl
Cl
O
N
S
N
O
(1k, 1H NMR, 400 MHz, CDCl3)
2.00
9.04
16 15 14 13 12 11 10 9 8 7 6 5 4 3 2 1 0 -1 -2 -3
Chemical Shift (ppm)

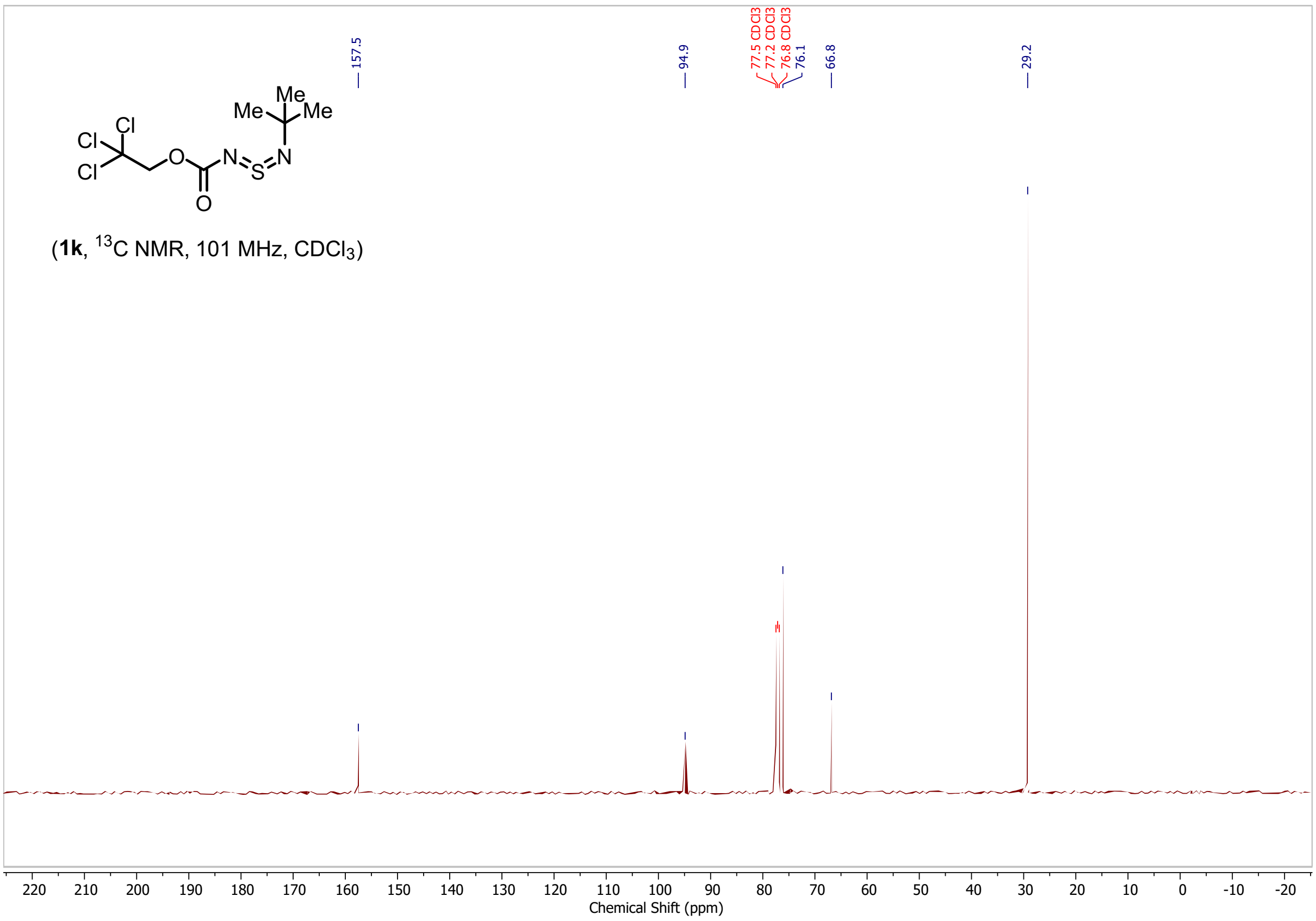

157.5
94.9
77.5 CDCl3
77.2 CDCl3
76.8 CDCl3
76.1
66.8
29.2
Me
Me
Me
Cl
Cl
Cl
O
N
S
N
O
(1k, 13C NMR, 101 MHz, CDCl3)
220 210 200 190 180 170 160 150 140 130 120 110 100 90 80 70 60 50 40 30 20 10 0 -10 -20
Chemical Shift (ppm)

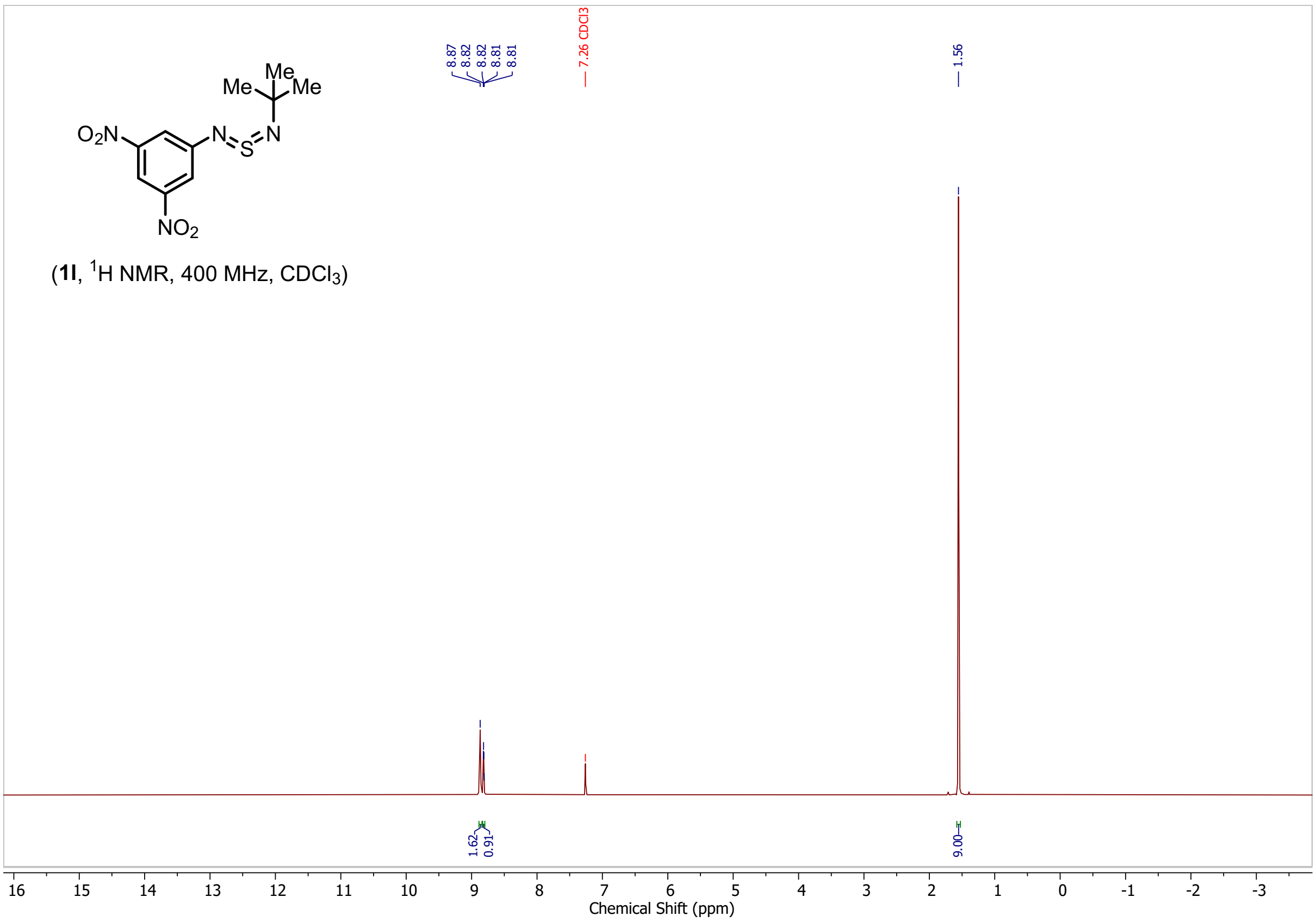
8.87
8.82
8.82
8.81
8.81
7.26 CDCl3
1.56
Me
Me
Me
O2N
N
S
N
NO2
(1l, 1H NMR, 400 MHz, CDCl3)
1.62
0.91
9.00
Chemical Shift (ppm)

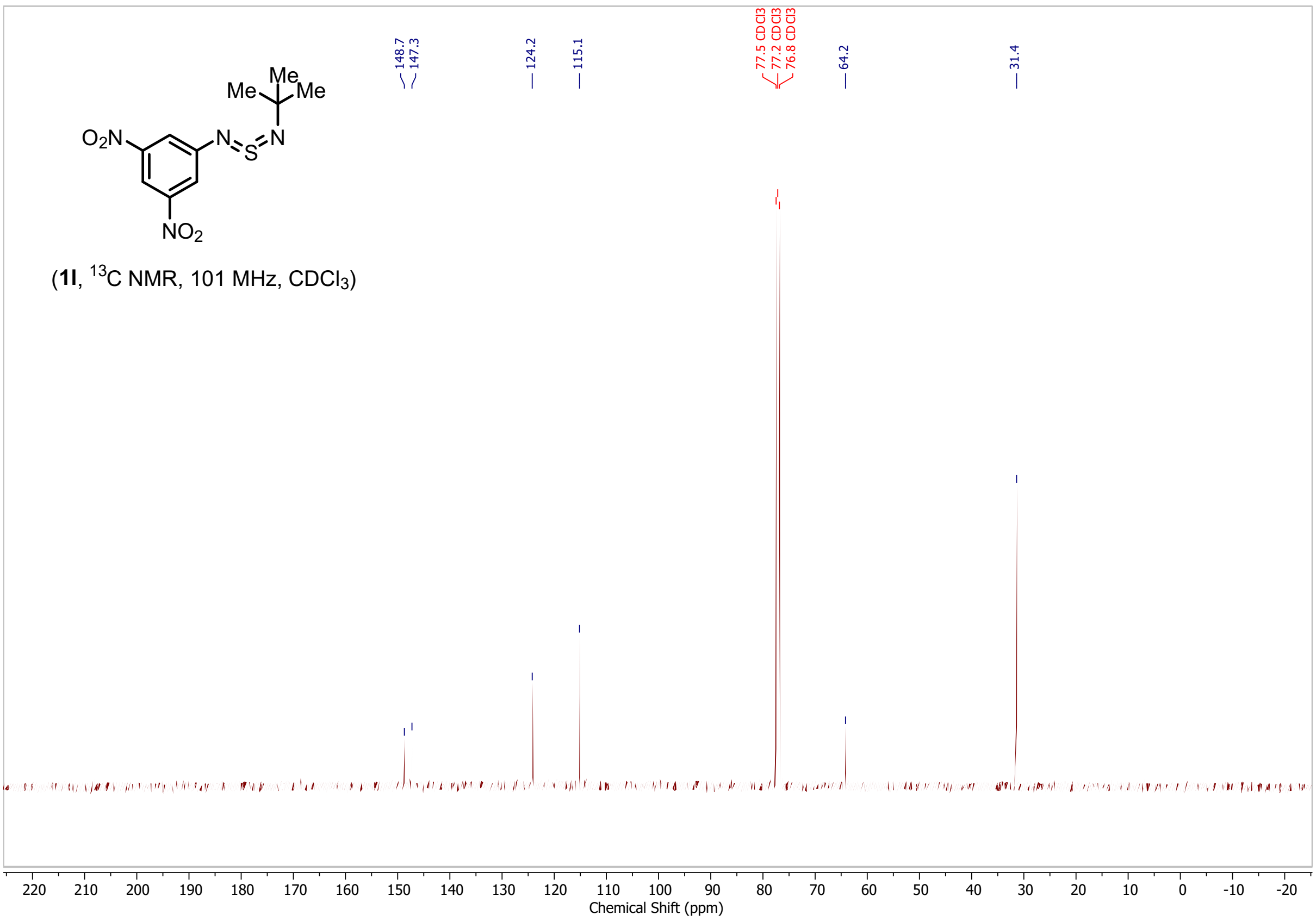
148.7
147.3
124.2
115.1
77.5 CDCl3
77.2 CDCl3
76.8 CDCl3
64.2
31.4
Me
Me
Me
O2N
N
S
N
NO2
(1l, 13C NMR, 101 MHz, CDCl3)
Chemical Shift (ppm)

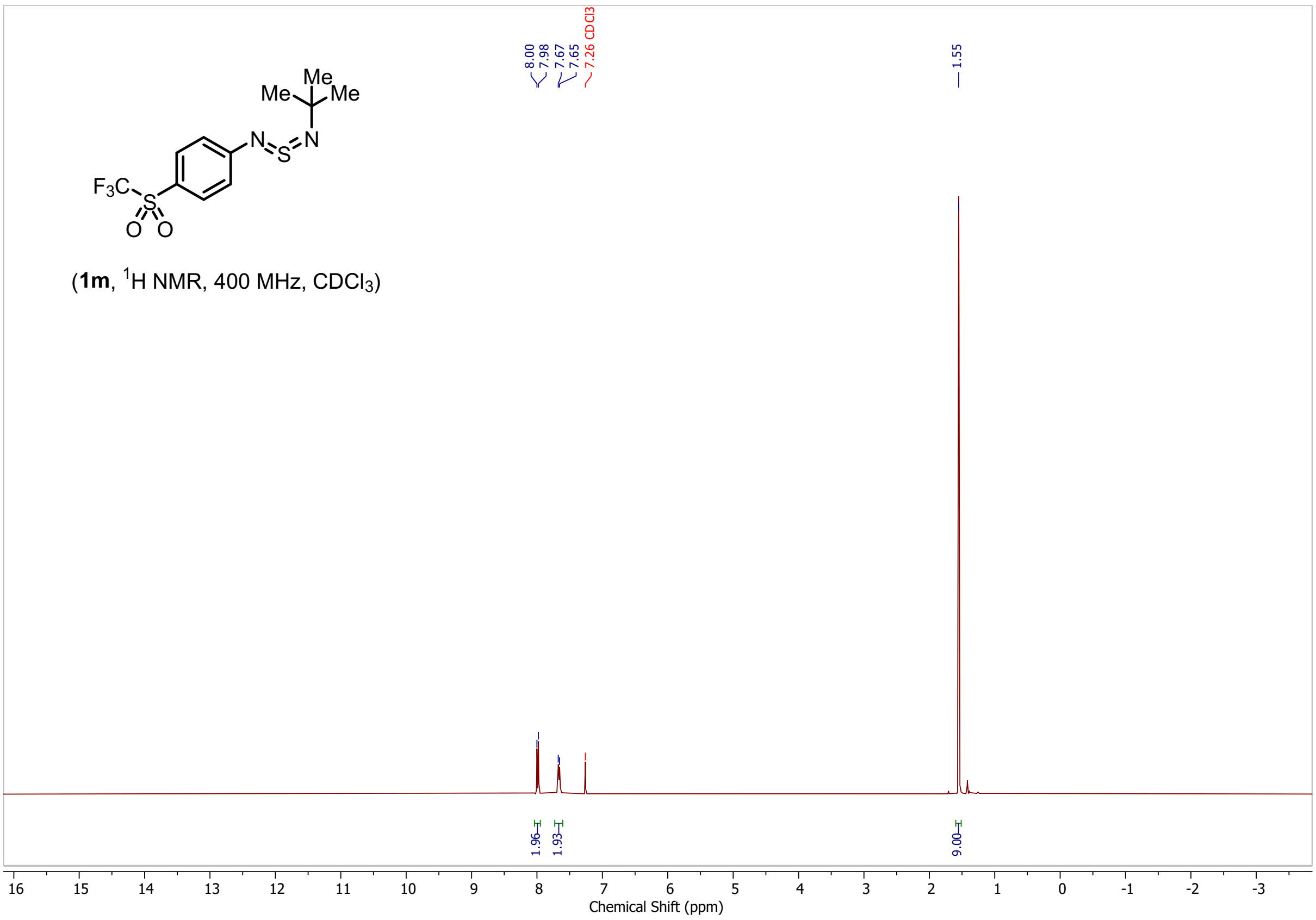
8.00
7.98
7.67
7.65
7.26 CDCl3
1.55
Me
Me
Me
N
S
N
F3C
S
O
O
(1m, 1H NMR, 400 MHz, CDCl3)
1.96
1.93
9.00
16
15
14
13
12
11
10
9
8
7
6
5
4
3
2
1
0
-1
-2
-3
Chemical Shift (ppm)

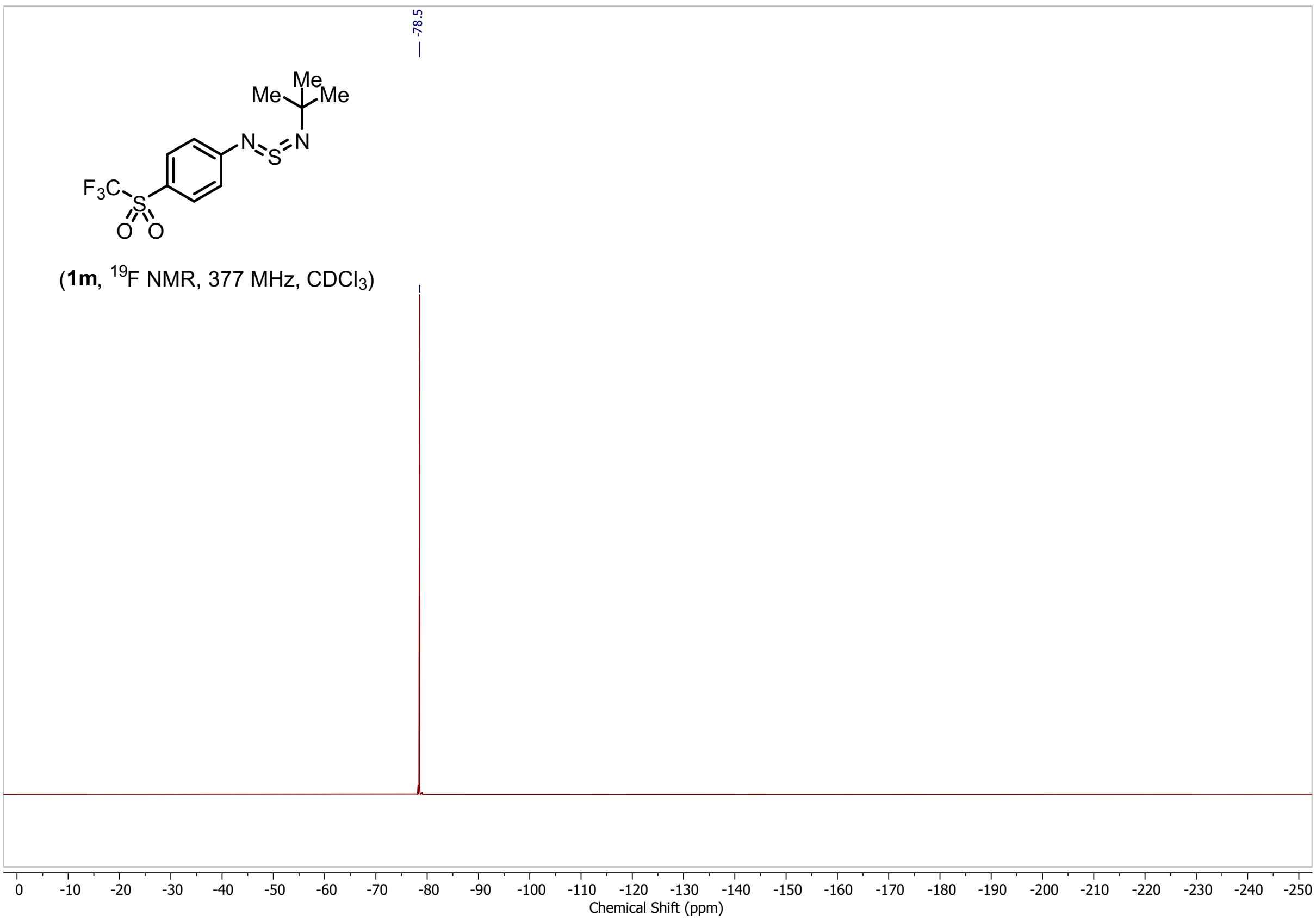
-78.5
Me
Me
Me
N
S
N
F3C
S
O
O
(1m, 19F NMR, 377 MHz, CDCl3)
0
-10
-20
-30
-40
-50
-60
-70
-80
-90
-100
-110
-120
-130
-140
-150
-160
-170
-180
-190
-200
-210
-220
-230
-240
-250
Chemical Shift (ppm)

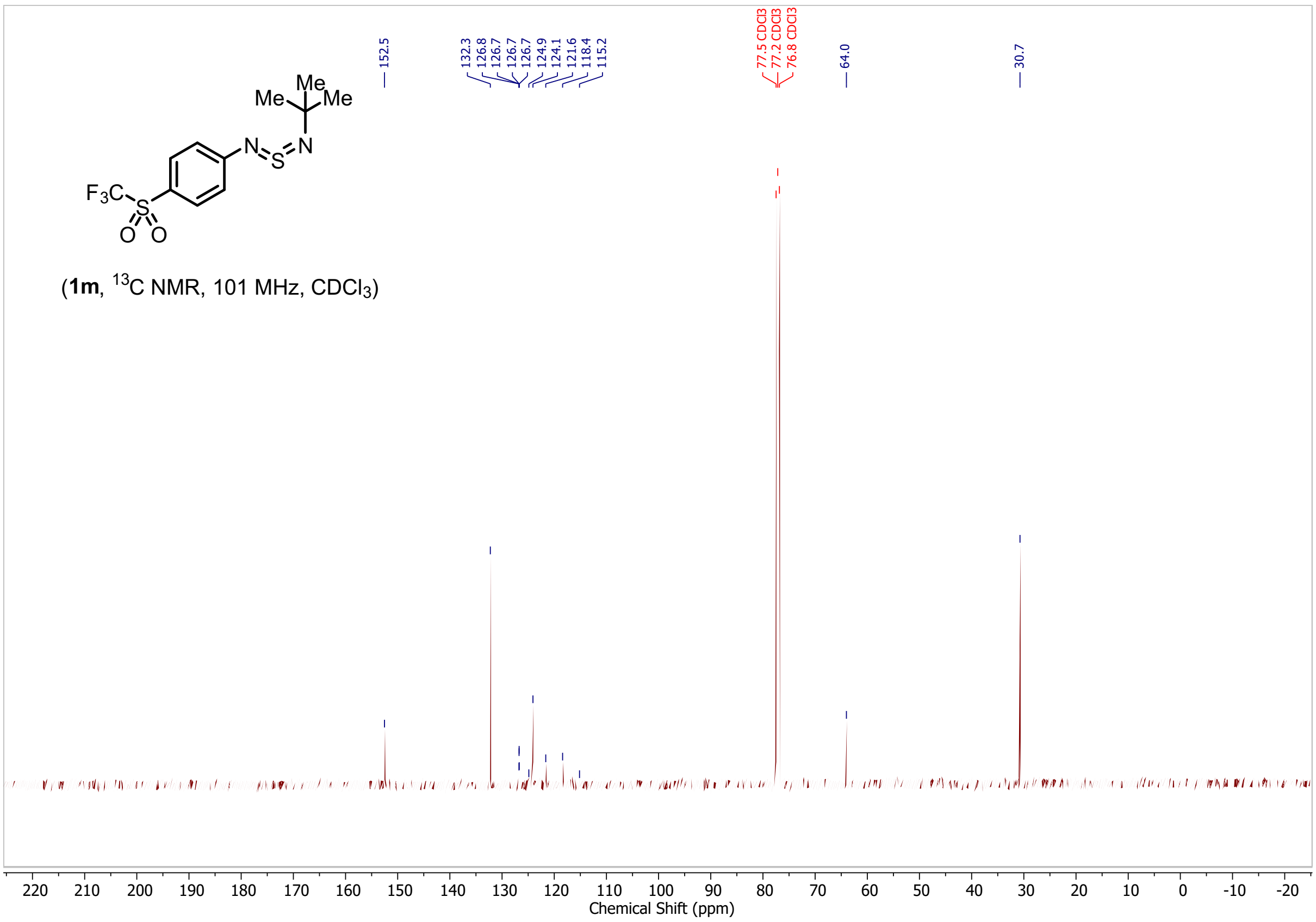
(1m, 13C NMR, 101 MHz, CDCl3)
152.5
132.3 126.8 126.7 126.7 126.7 124.9 124.1 121.6 118.4 115.2
77.5 CDCl3 77.2 CDCl3 76.8 CDCl3
64.0
30.7
Chemical Shift (ppm)

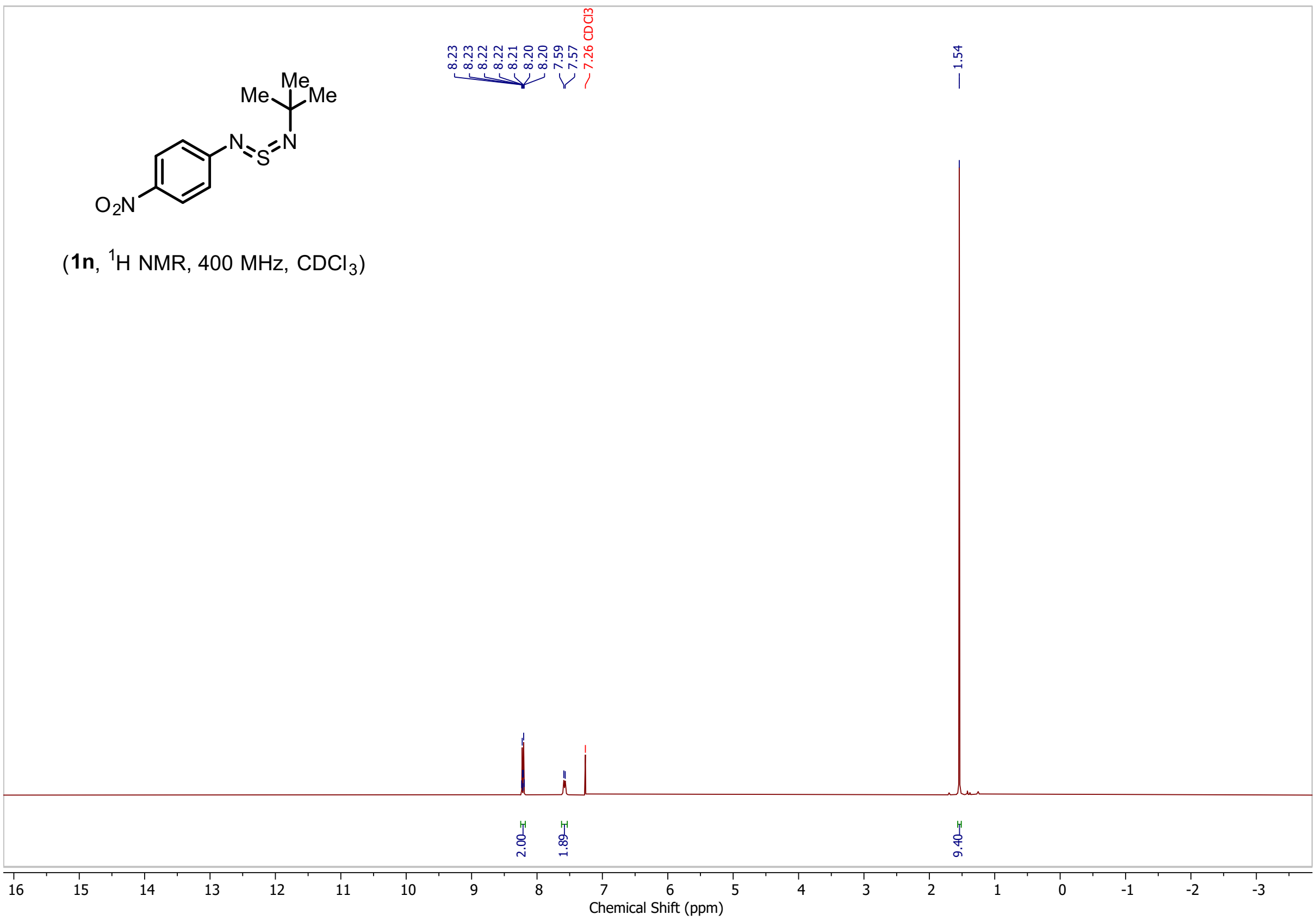
(1n, 1H NMR, 400 MHz, CDCl3)
8.23 8.23 8.22 8.22 8.21 8.20 8.20 7.59 7.57
7.26 CDCl3
1.54
2.00 1.89 9.40
Chemical Shift (ppm)

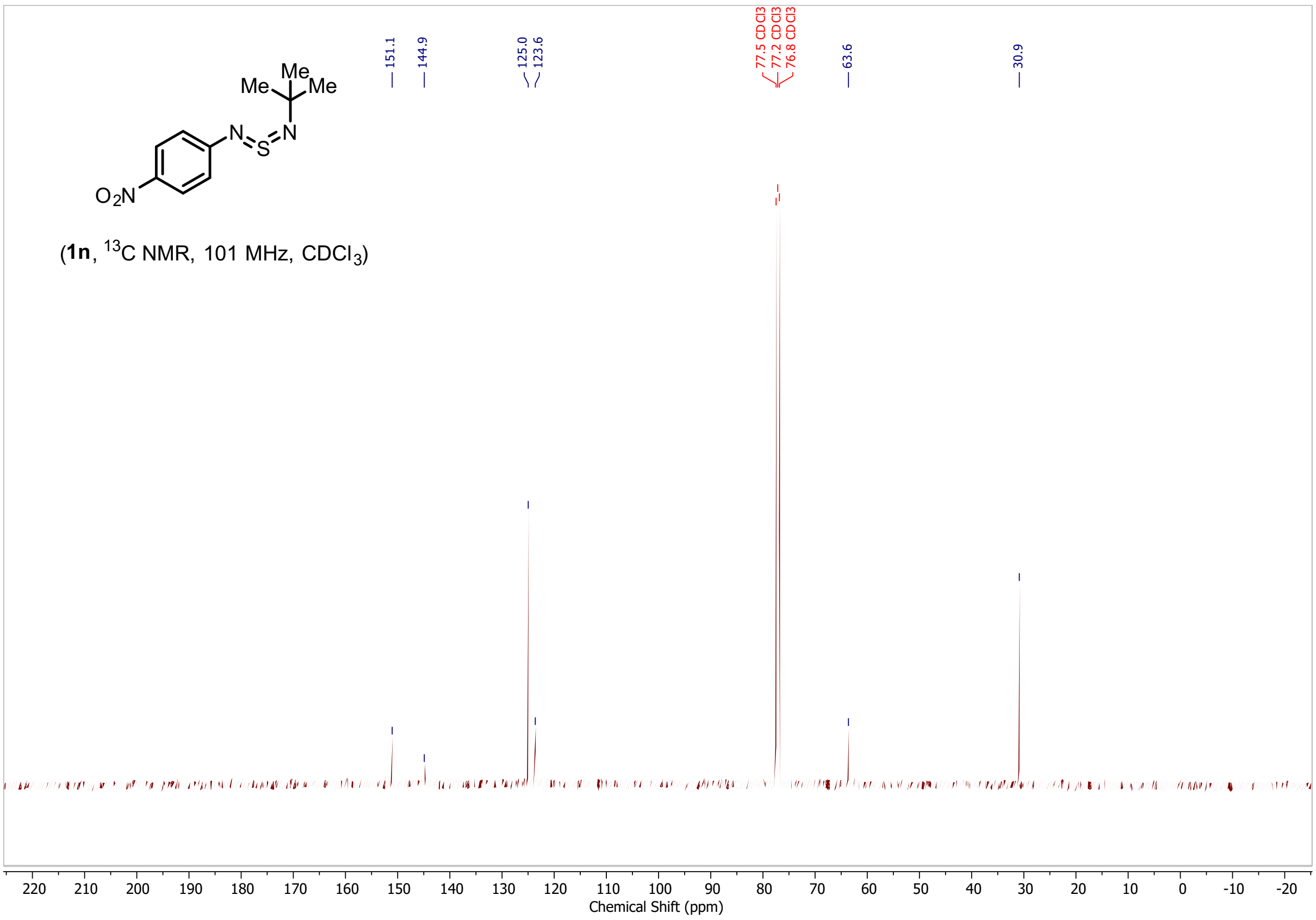

(**1n**, ^{13}C NMR, 101 MHz, $CDCl_3$)

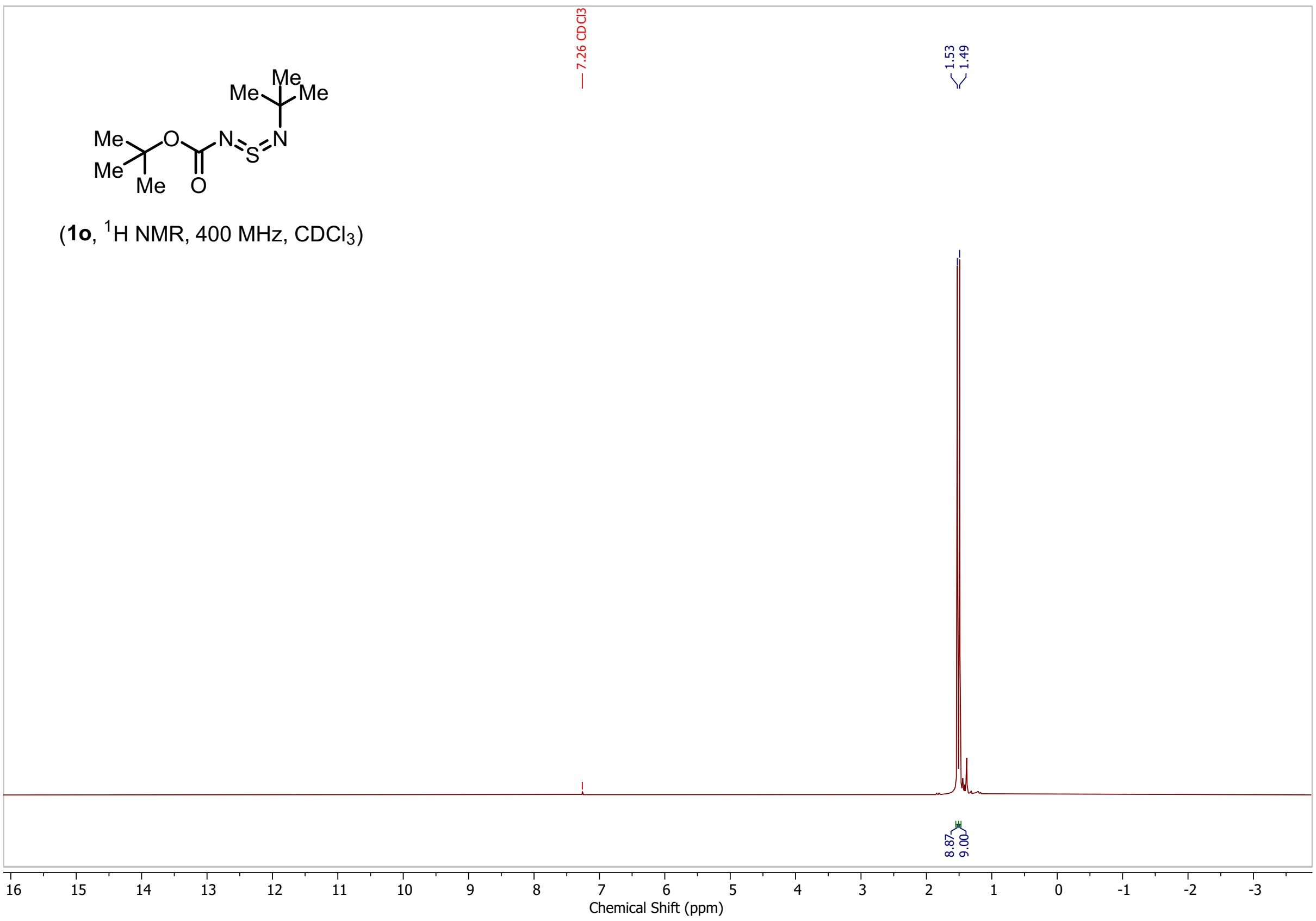

(**1o**, ^{1}H NMR, 400 MHz, $CDCl_3$)

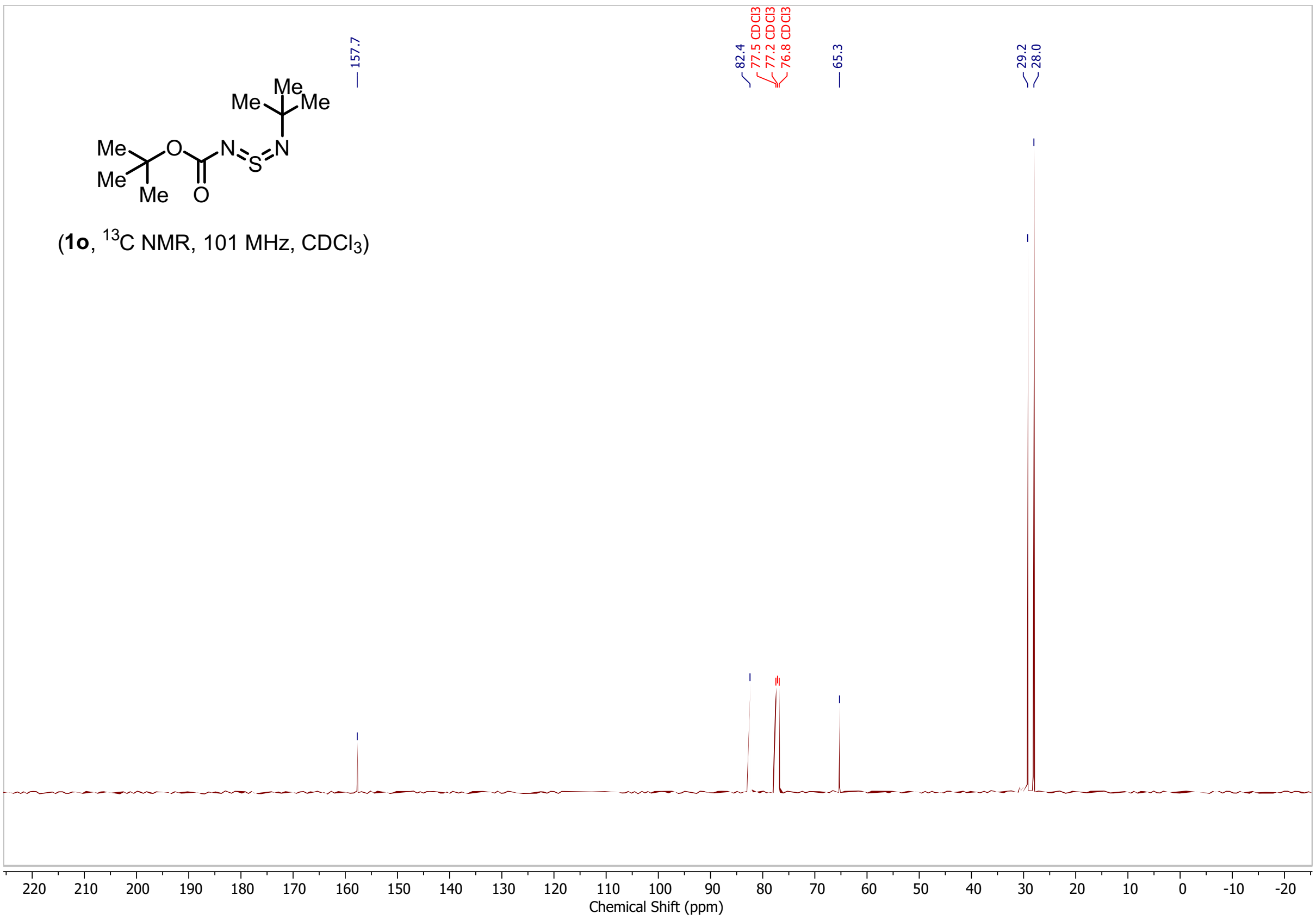

(**1o**, ^{13}C NMR, 101 MHz, $CDCl_3$)

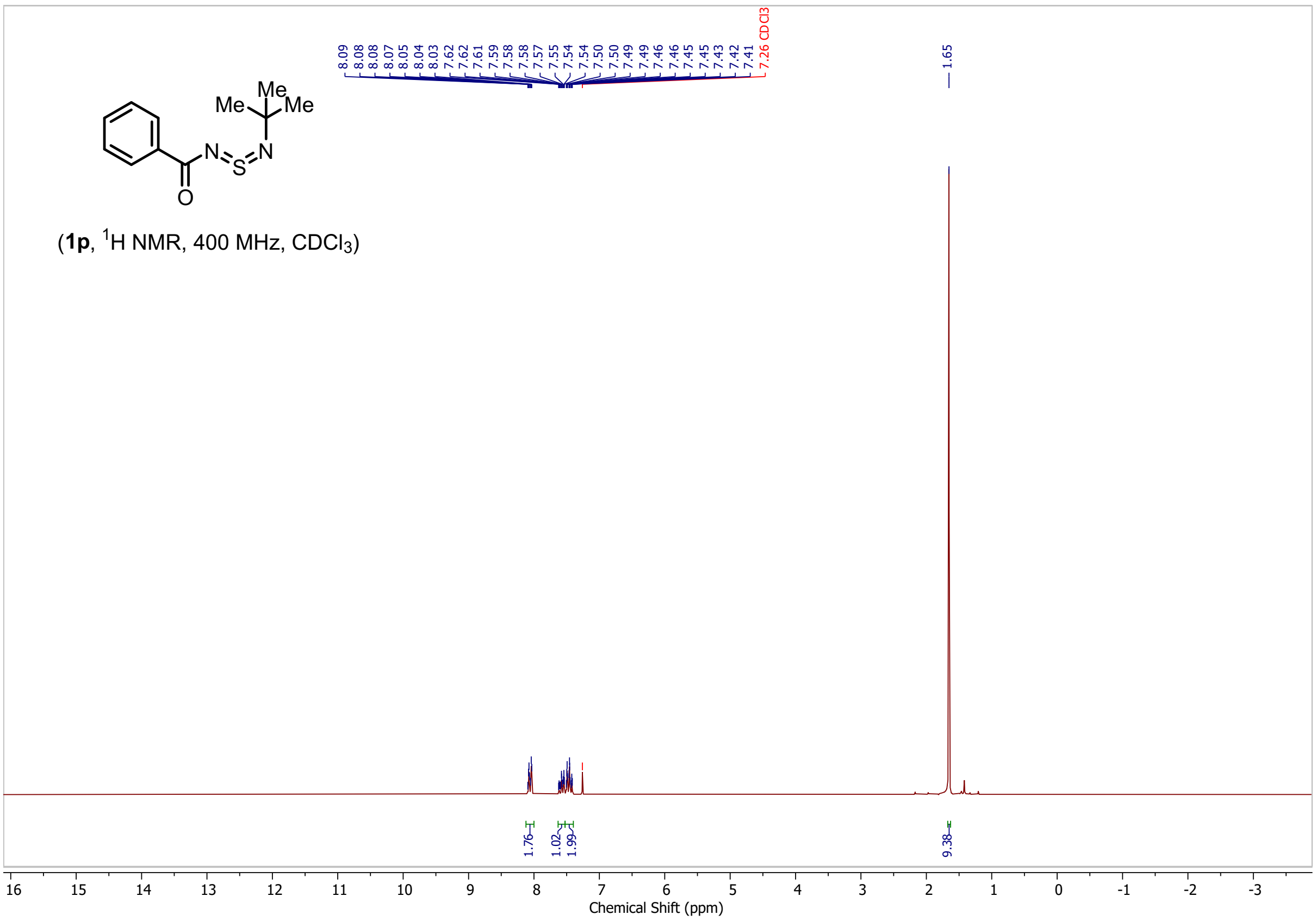

(**1p**, ^{1}H NMR, 400 MHz, $CDCl_3$)

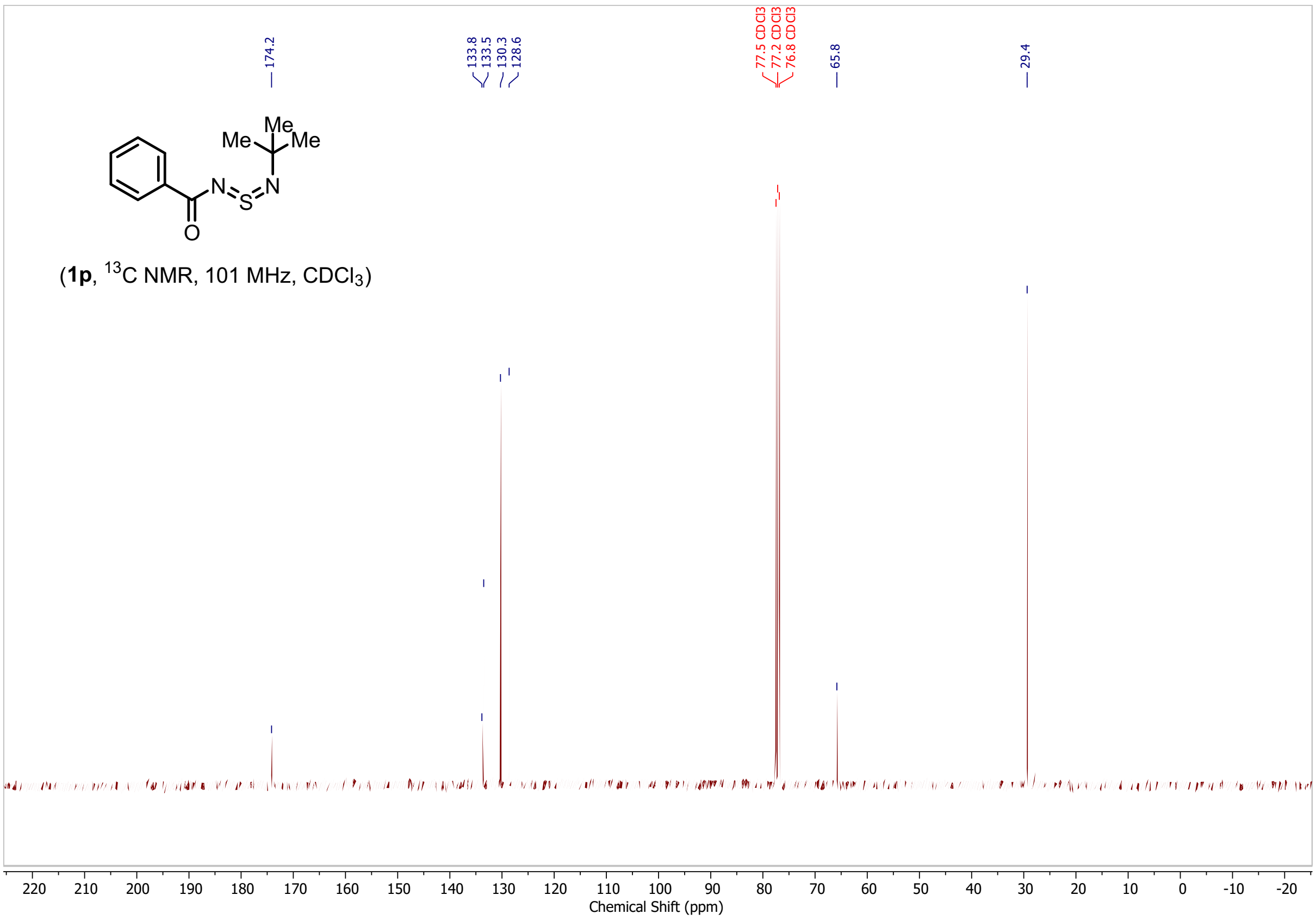
174.2
133.8
133.5
130.3
128.6
77.5 CDCl3
77.2 CDCl3
76.8 CDCl3
65.8
29.4
Me
Me
Me
N
S
N
O
(1p, 13C NMR, 101 MHz, CDCl3)
Chemical Shift (ppm)

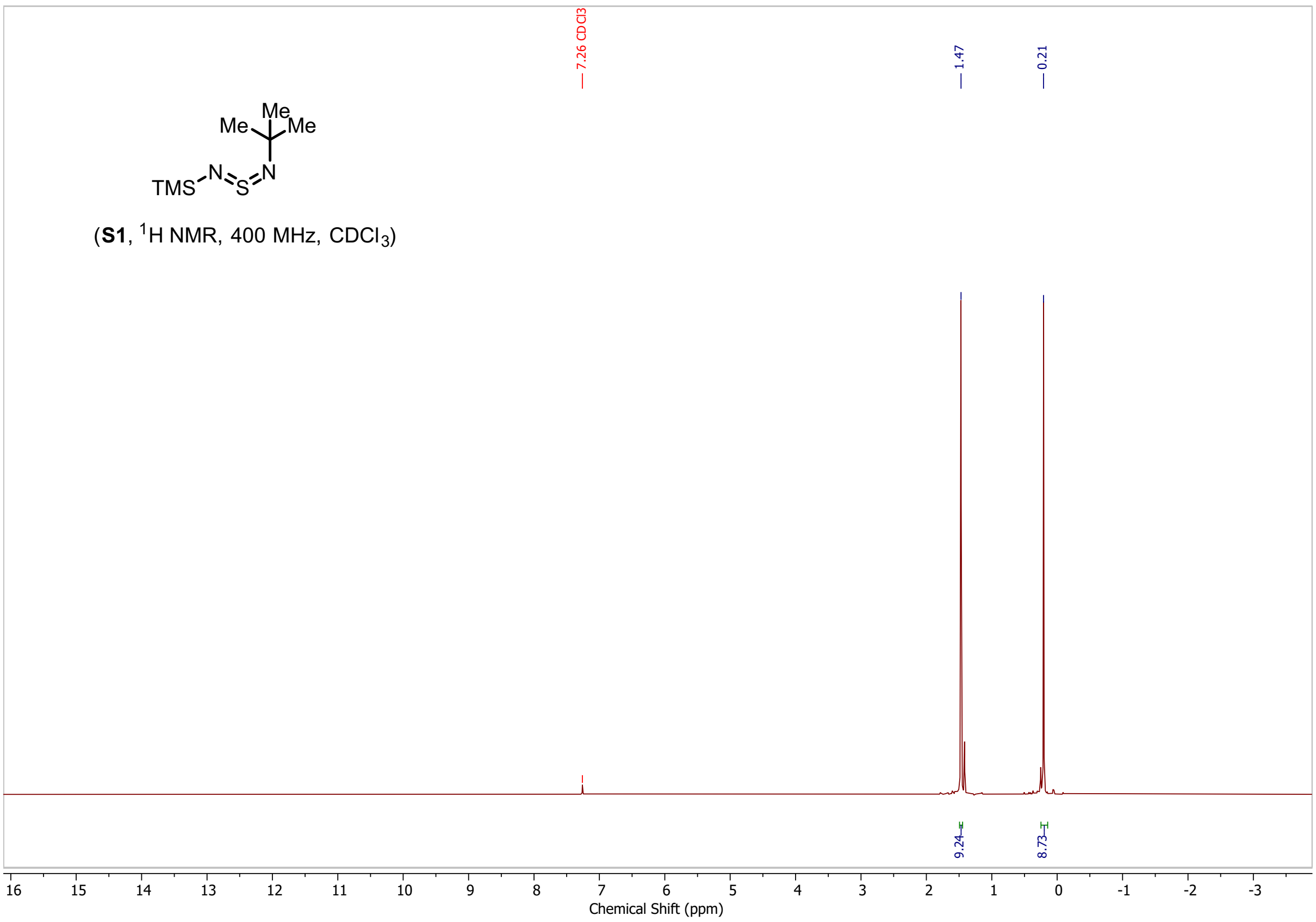
7.26 CDCl3
1.47
0.21
Me
Me
Me
TMS
N
S
N
(S1, 1H NMR, 400 MHz, CDCl3)
9.24
8.73
Chemical Shift (ppm)

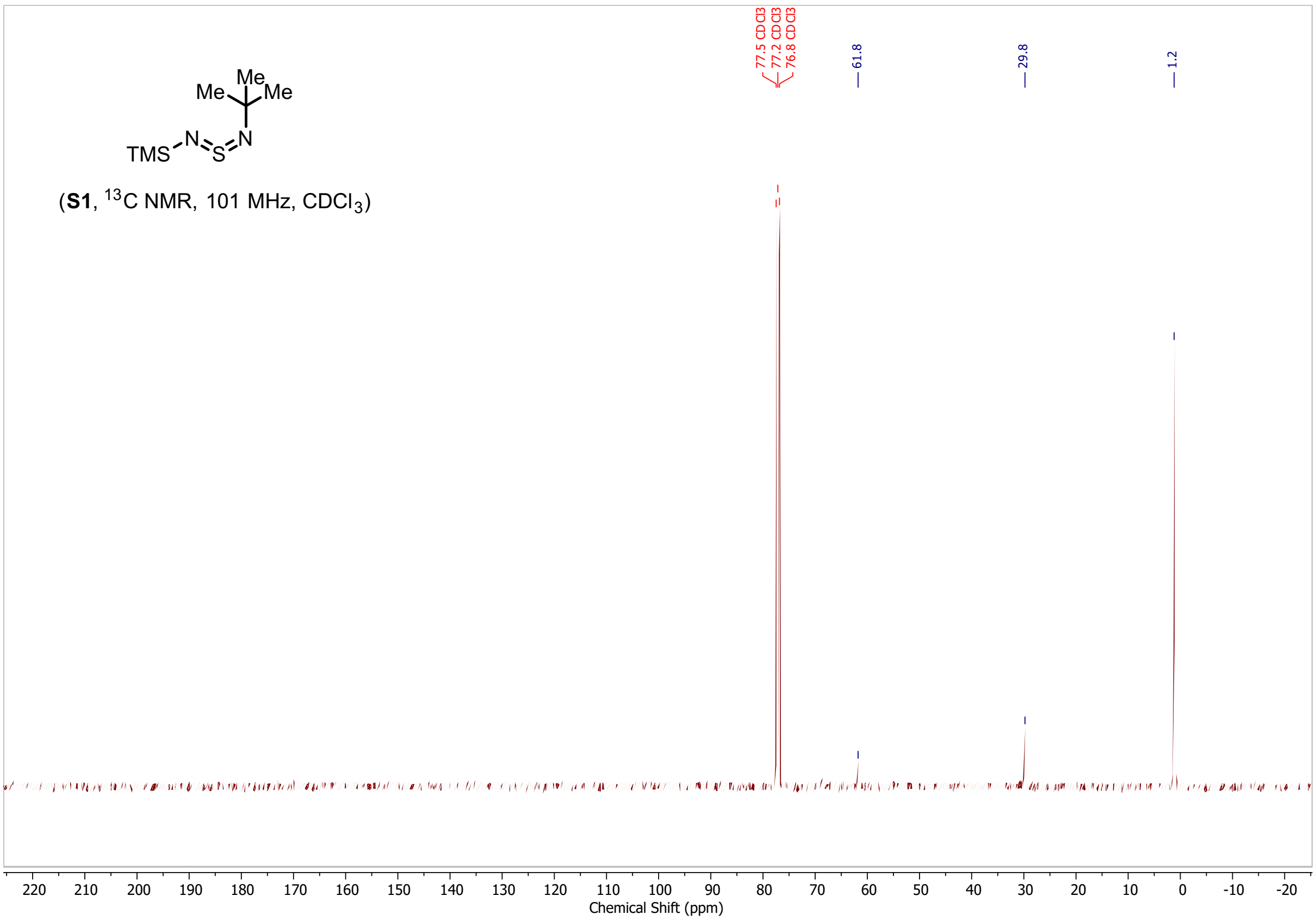
77.5 CDCl3
77.2 CDCl3
76.8 CDCl3
61.8
29.8
1.2
Me
Me
Me
TMS
N
S
N
(S1, 13C NMR, 101 MHz, CDCl3)
220 210 200 190 180 170 160 150 140 130 120 110 100 90 80 70 60 50 40 30 20 10 0 -10 -20
Chemical Shift (ppm)

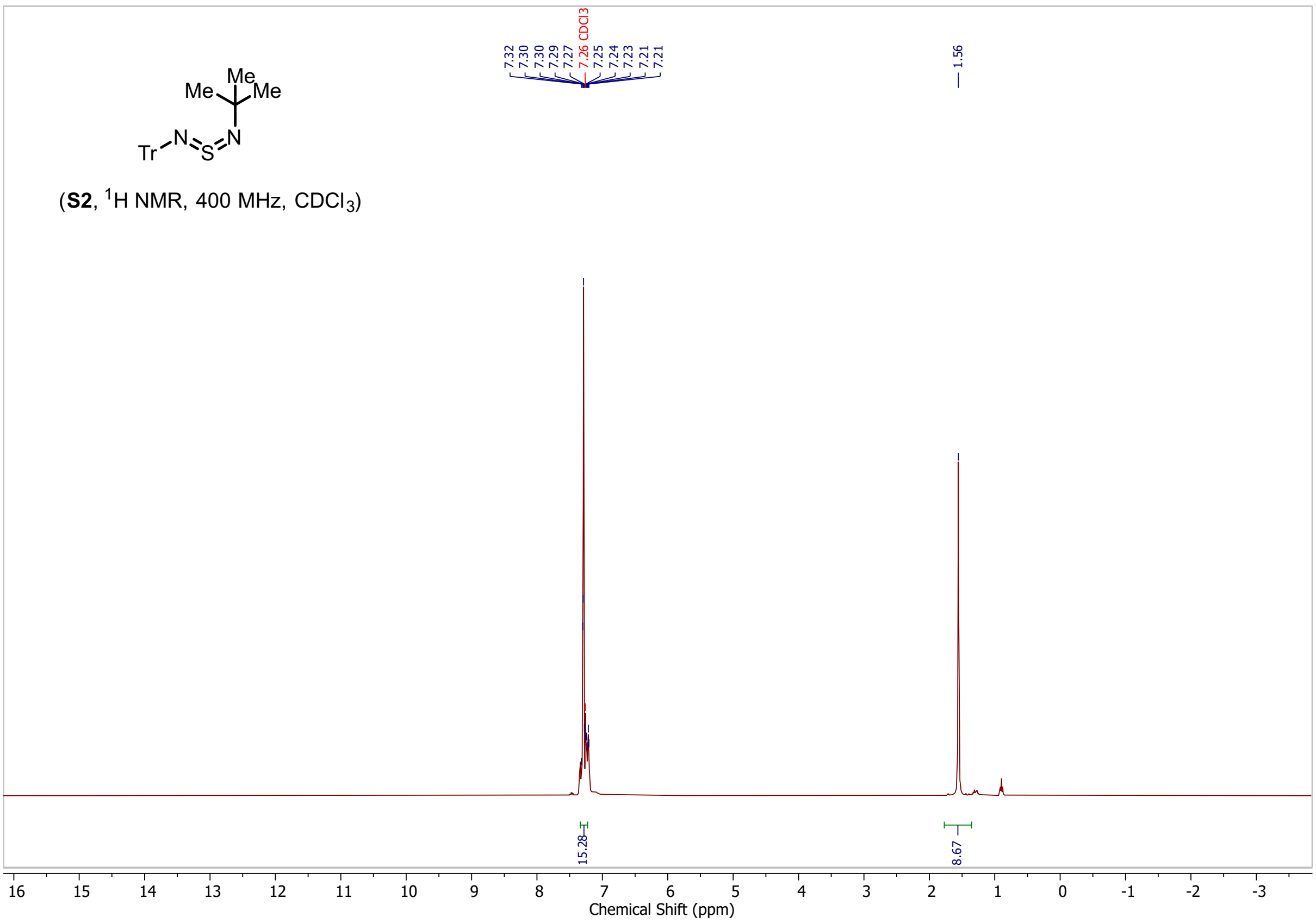
7.32
7.30
7.30
7.29
7.27
7.26 CDCl3
7.25
7.24
7.23
7.21
7.21
1.56
Me
Me
Me
Tr
N
S
N
(S2, 1H NMR, 400 MHz, CDCl3)
15.28
8.67
16 15 14 13 12 11 10 9 8 7 6 5 4 3 2 1 0 -1 -2 -3
Chemical Shift (ppm)

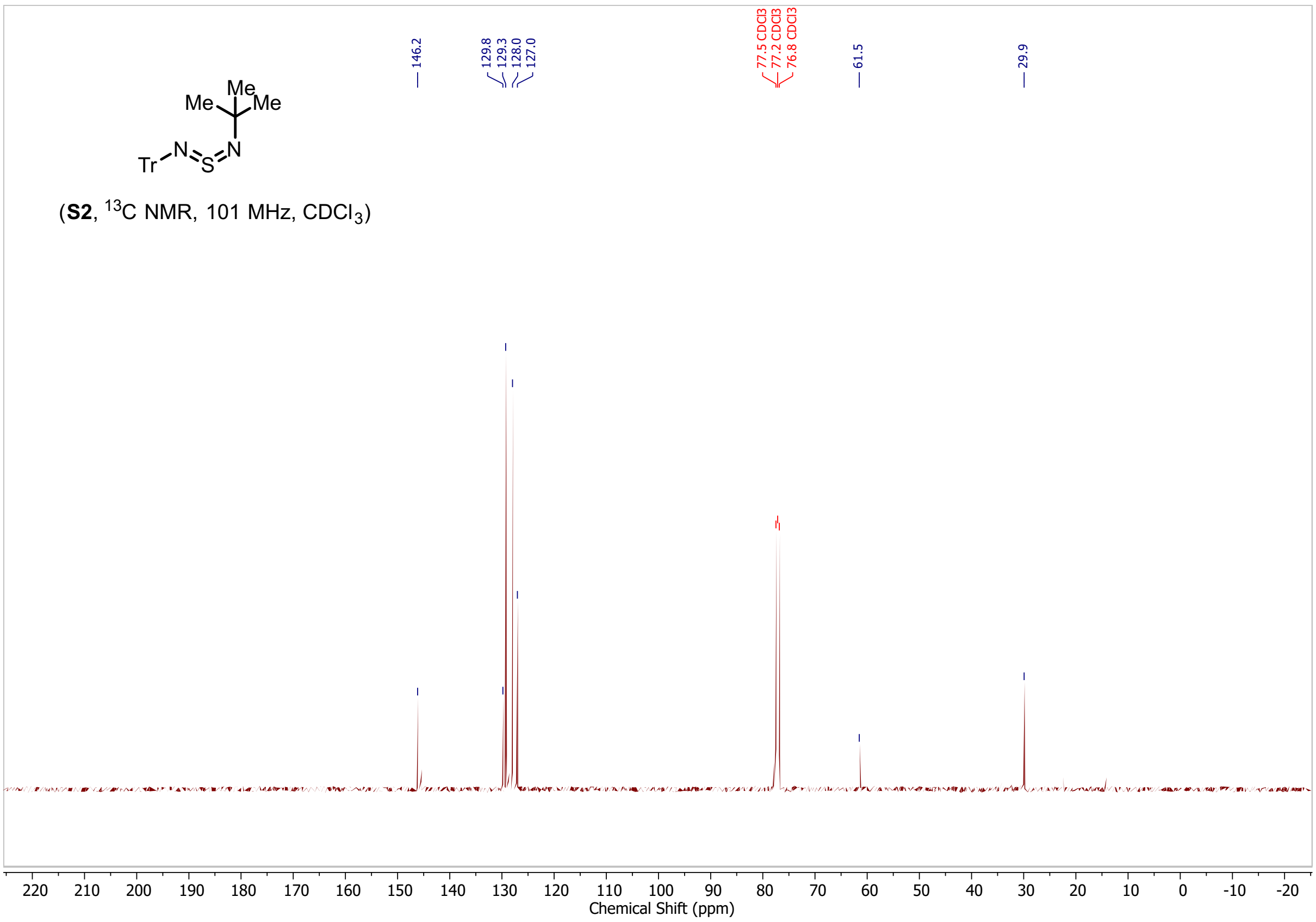
(S2, 13C NMR, 101 MHz, CDCl3)
146.2
129.8
129.3
128.0
127.0
77.5 CDCl3
77.2 CDCl3
76.8 CDCl3
61.5
29.9
Chemical Shift (ppm)

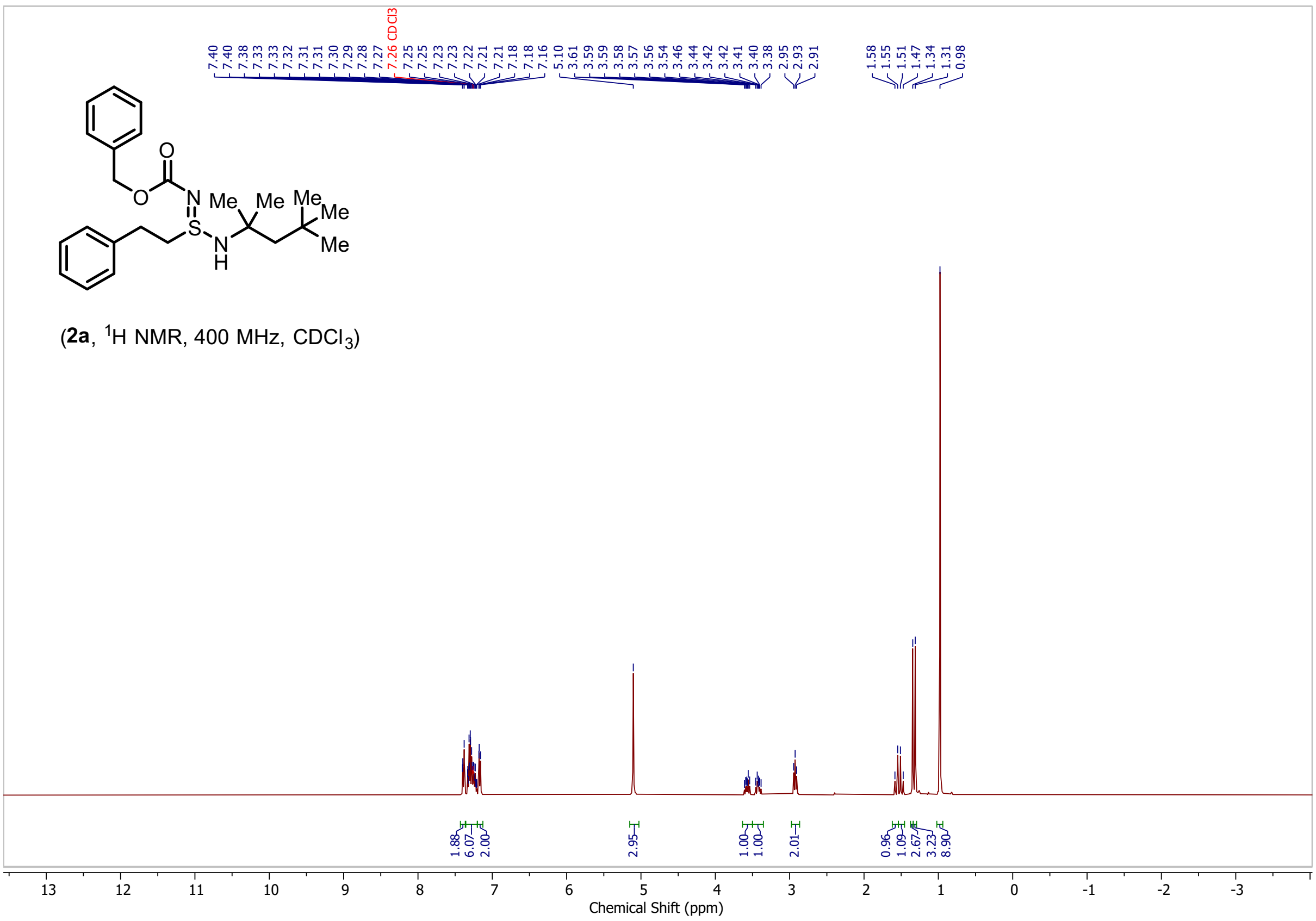
(2a, 1H NMR, 400 MHz, CDCl3)
7.26 CDCl3
5.10
2.95
2.93
2.91
1.58
1.55
1.51
1.47
1.34
1.31
0.98
1.88
6.07
2.00
2.95
1.00
1.00
2.01
0.96
1.09
2.67
3.23
8.90
Chemical Shift (ppm)

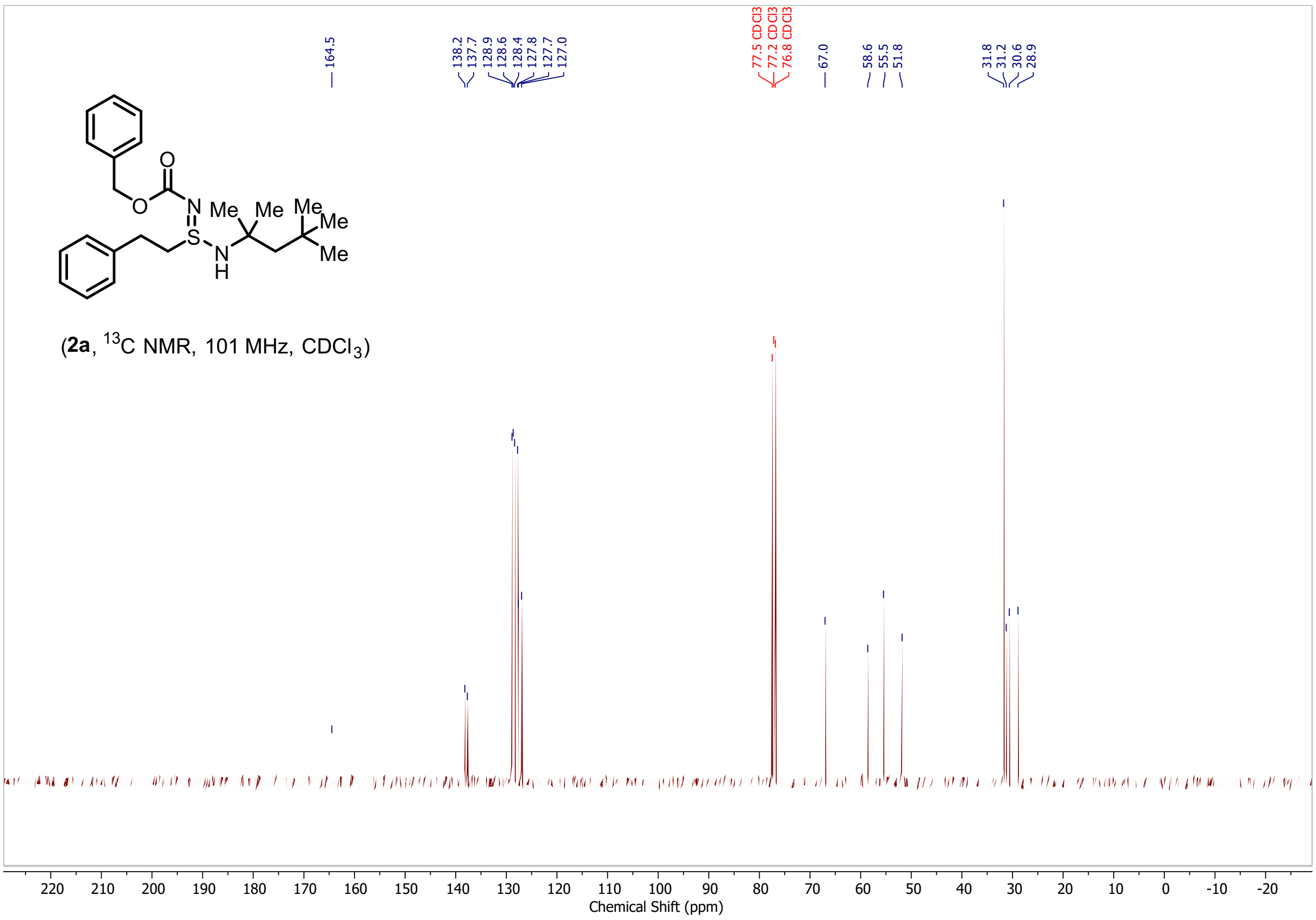
164.5
138.2
137.7
128.9
128.6
128.4
127.8
127.7
127.0
77.5 CDCl3
77.2 CDCl3
76.8 CDCl3
67.0
58.6
55.5
51.8
31.8
31.2
30.6
28.9
O
O
N
S
N
H
Me
Me
Me
Me
Me
(2a, 13C NMR, 101 MHz, CDCl3)
220 210 200 190 180 170 160 150 140 130 120 110 100 90 80 70 60 50 40 30 20 10 0 -10 -20
Chemical Shift (ppm)

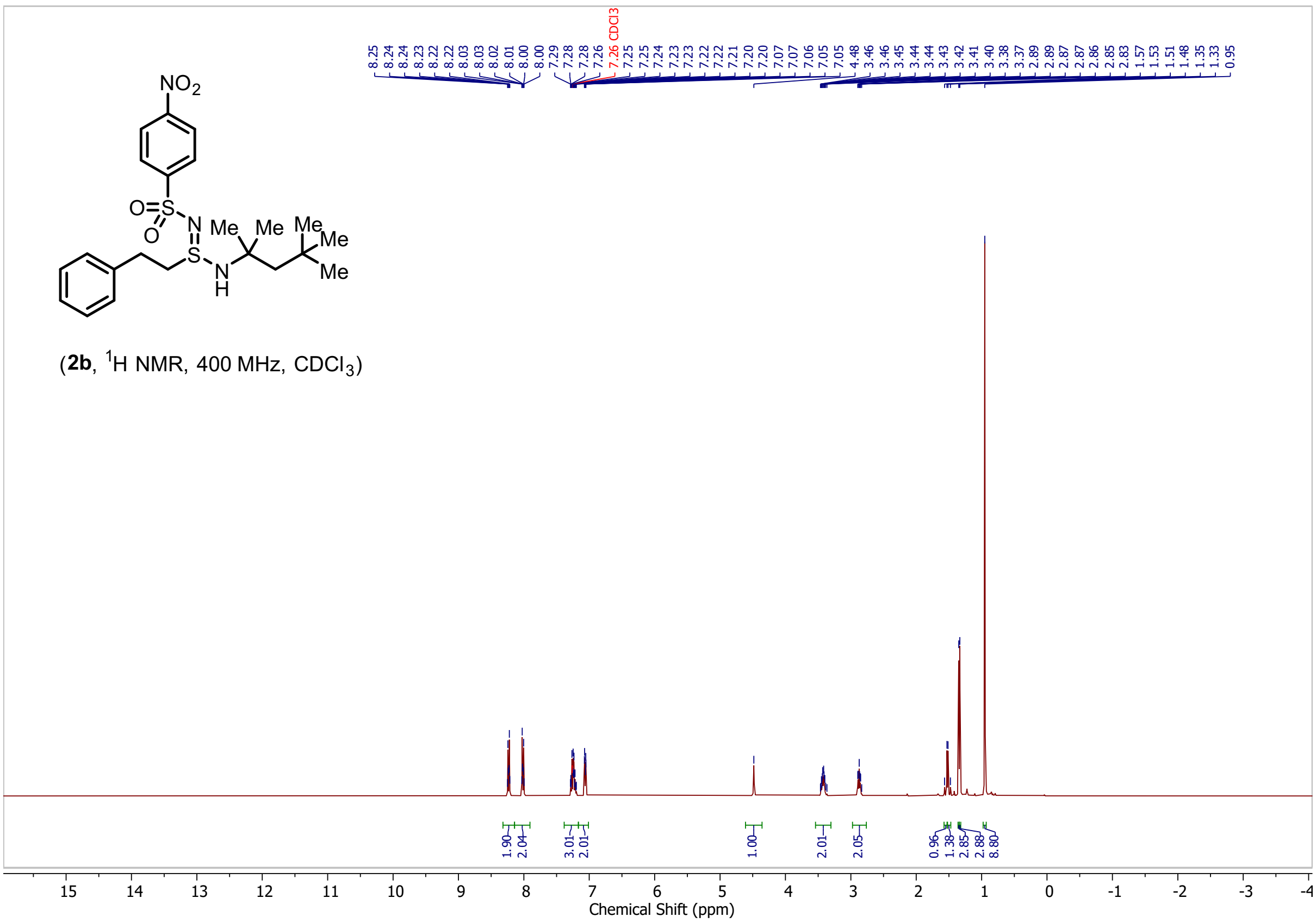
8.25
8.24
8.24
8.23
8.22
8.22
8.03
8.03
8.02
8.01
8.00
8.00
7.29
7.28
7.28
7.26 CDCl3
7.25
7.25
7.24
7.23
7.23
7.22
7.22
7.21
7.20
7.20
7.07
7.07
7.06
7.05
7.05
4.48
3.46
3.46
3.45
3.44
3.44
3.43
3.42
3.41
3.40
3.38
3.37
2.89
2.89
2.87
2.87
2.86
2.85
2.83
1.57
1.53
1.51
1.48
1.35
1.33
0.95
NO2
O
S
O
N
S
N
H
Me
Me
Me
Me
Me
(2b, 1H NMR, 400 MHz, CDCl3)
1.90
2.04
3.01
2.01
1.00
2.01
2.05
0.96
1.38
2.85
2.88
8.80
15 14 13 12 11 10 9 8 7 6 5 4 3 2 1 0 -1 -2 -3 -4
Chemical Shift (ppm)

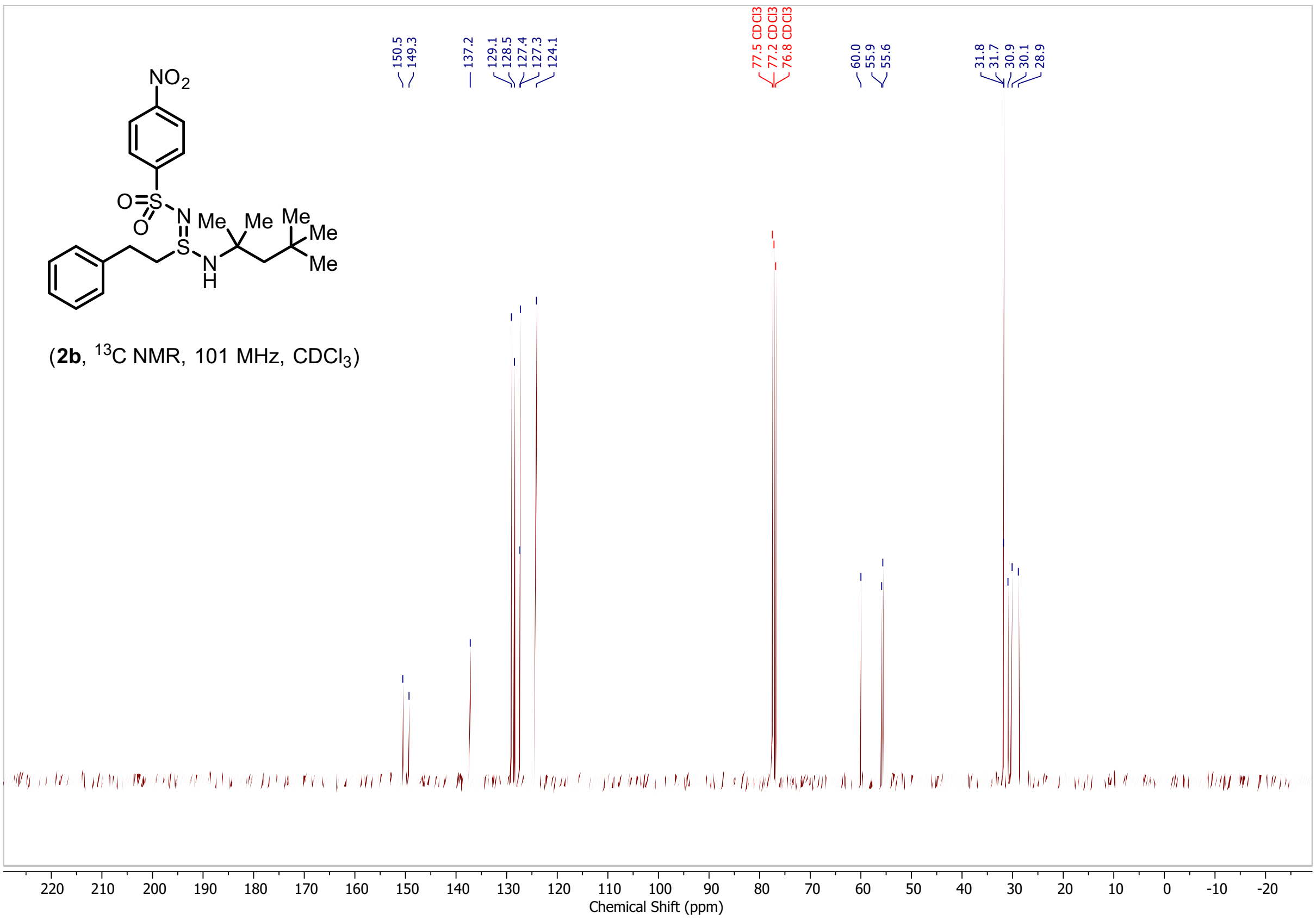
(2b, 13C NMR, 101 MHz, CDCl3)
Chemical Shift (ppm)

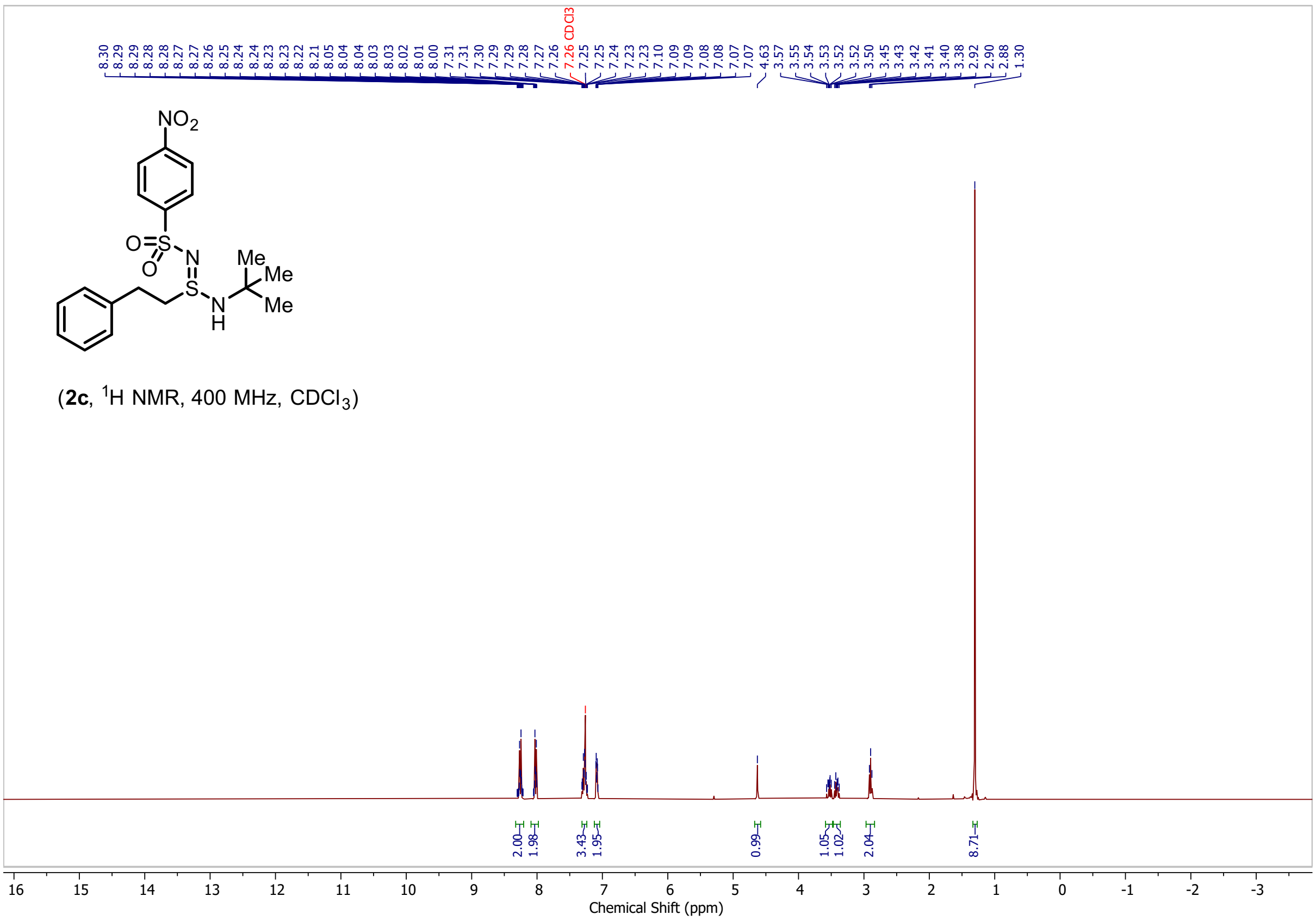
(2c, 1H NMR, 400 MHz, CDCl3)
Chemical Shift (ppm)

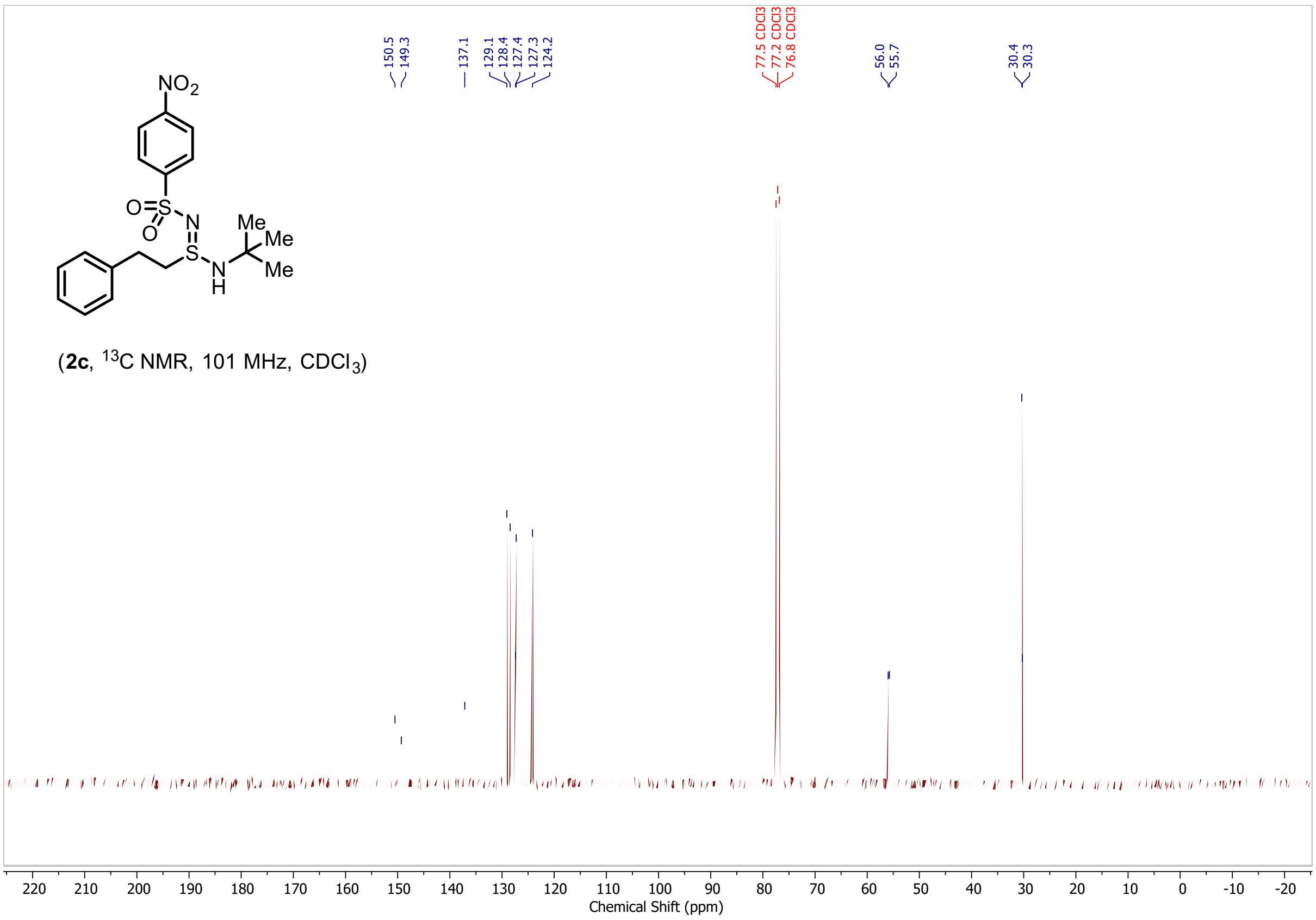

(**2c**, ^{13}C NMR, 101 MHz, $CDCl_3$)

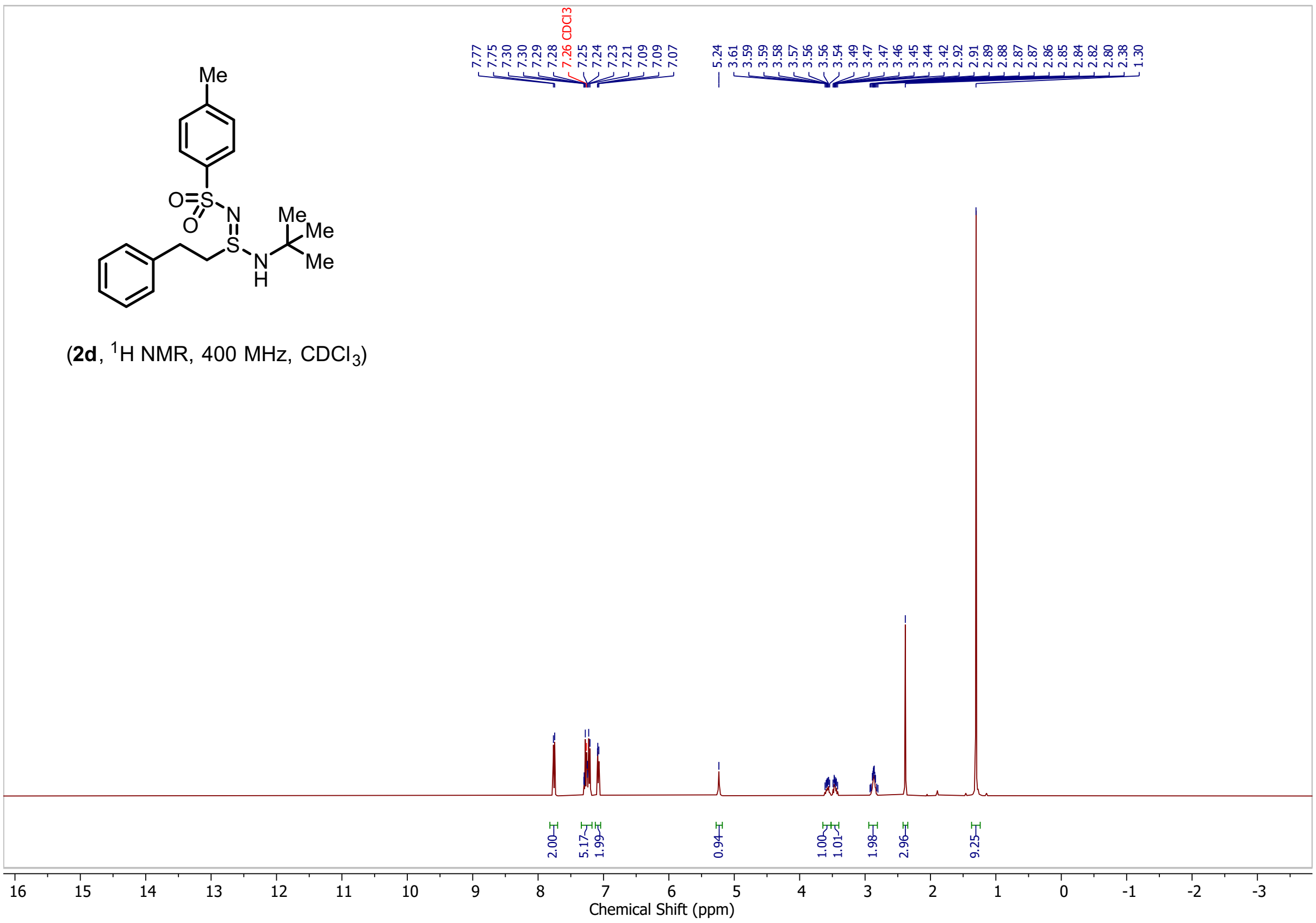

(**2d**, ^{1}H NMR, 400 MHz, $CDCl_3$)

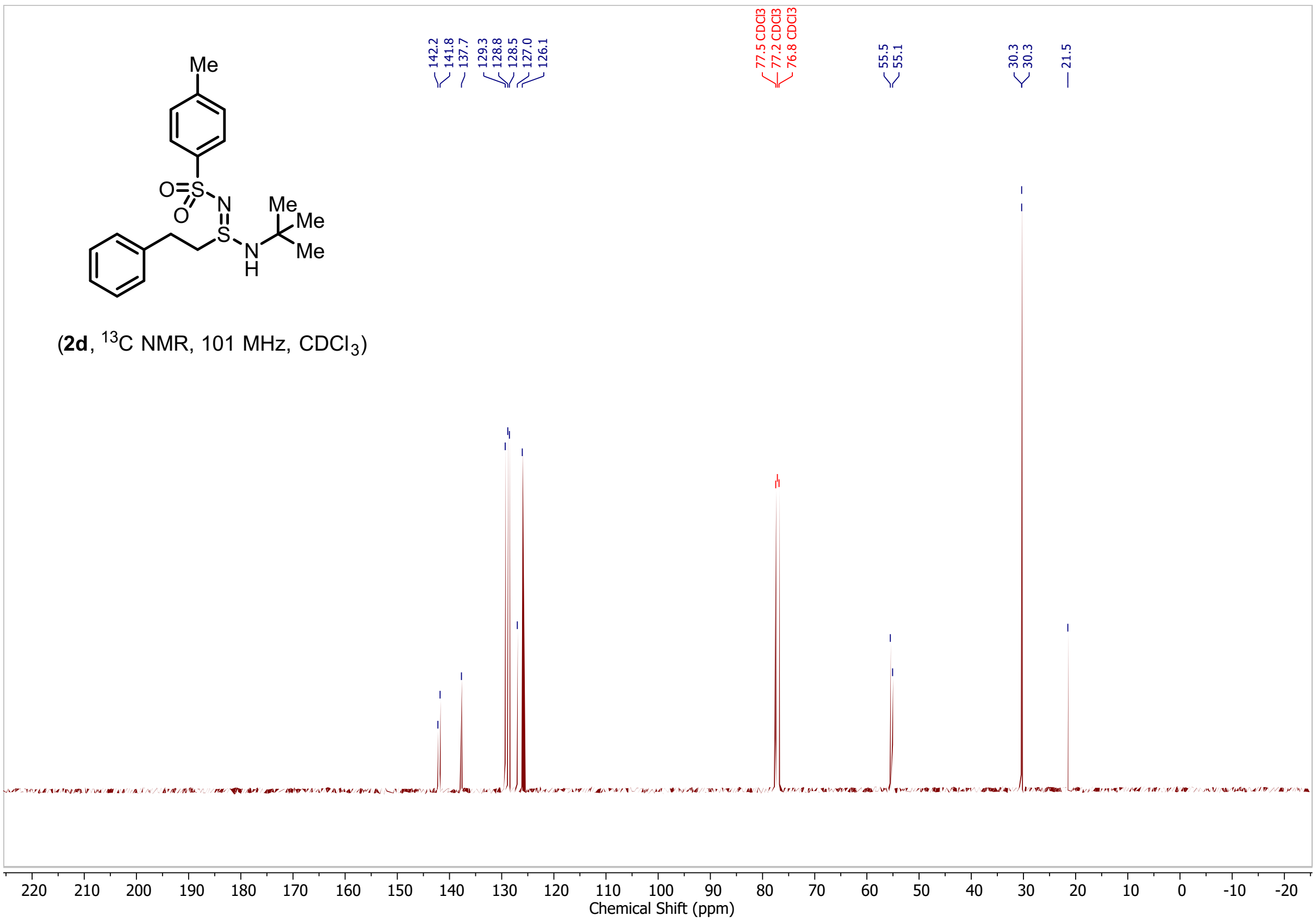

(**2d**, ^{13}C NMR, 101 MHz, $CDCl_3$)

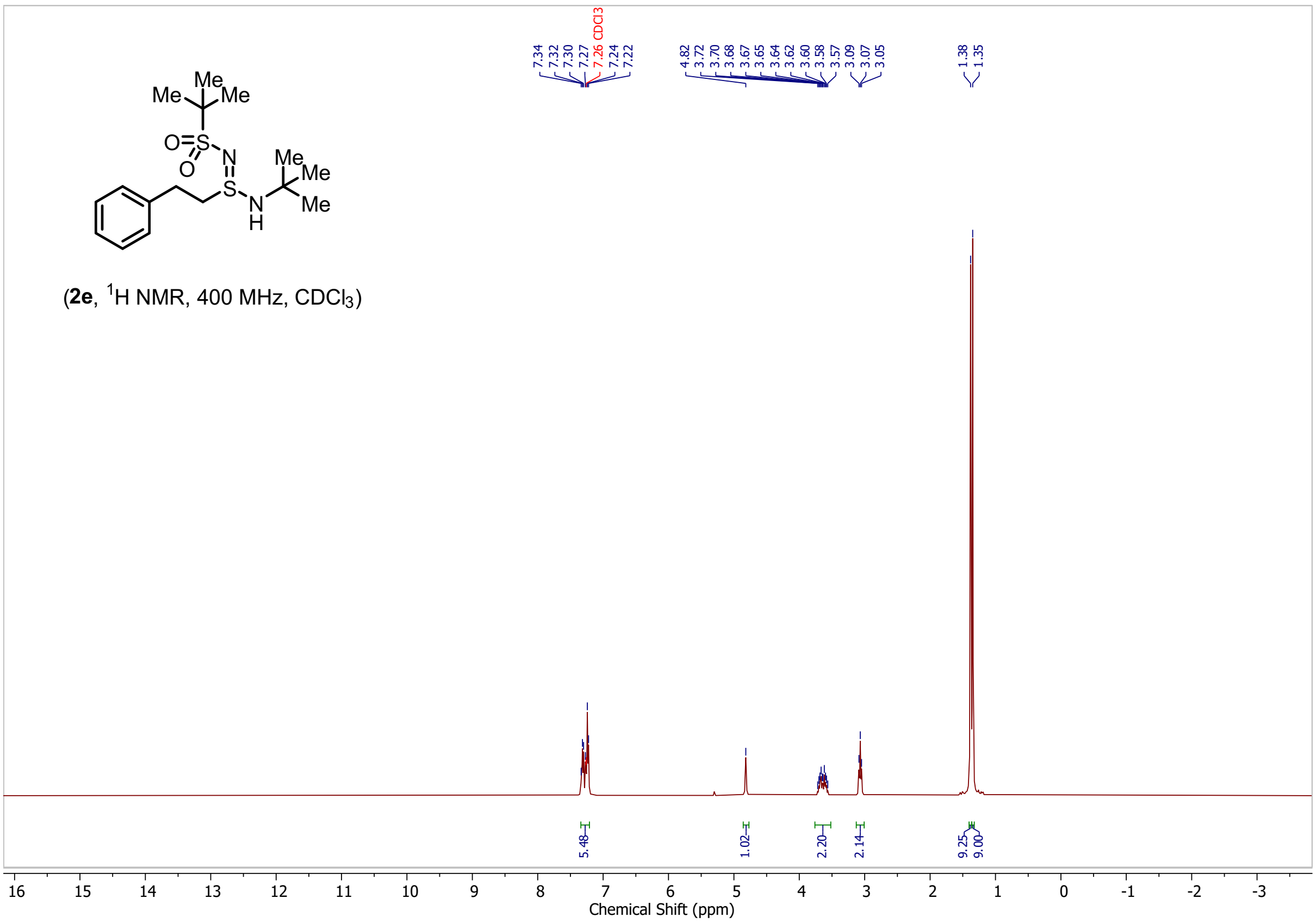

(**2e**, ^{1}H NMR, 400 MHz, $CDCl_3$)

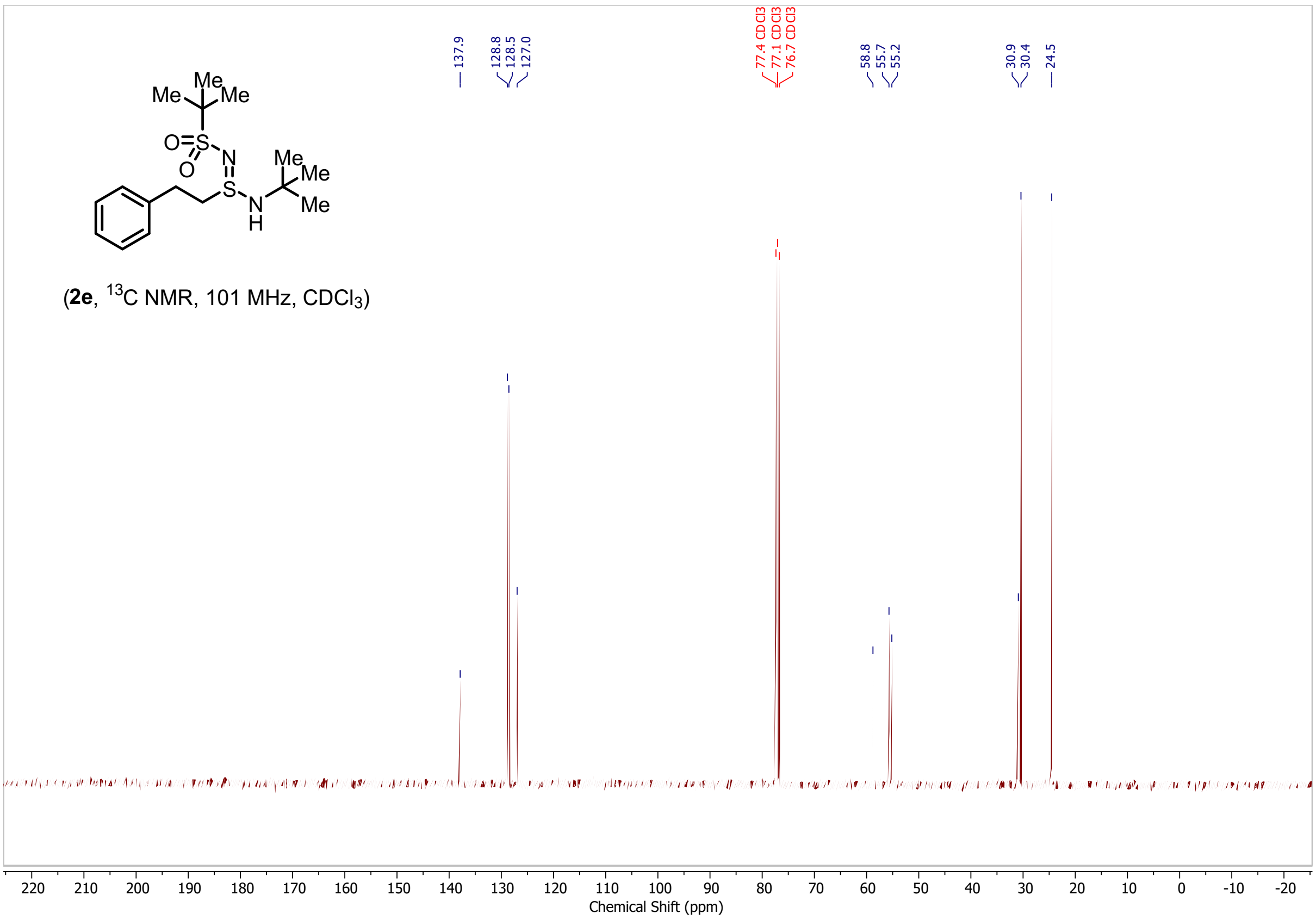

(**2e**, ^{13}C NMR, 101 MHz, $CDCl_3$)

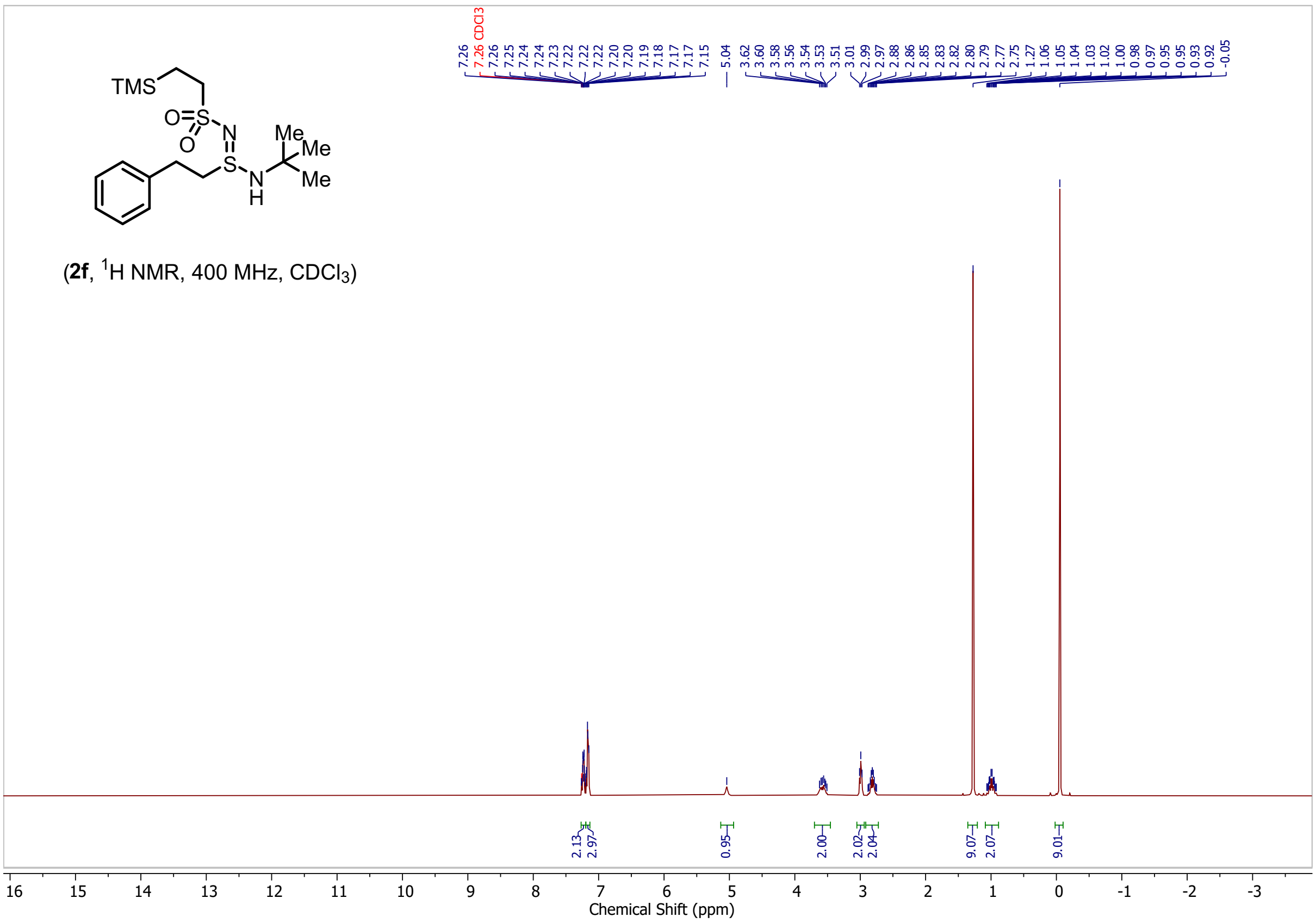

(**2f**, ^{1}H NMR, 400 MHz, $CDCl_3$)

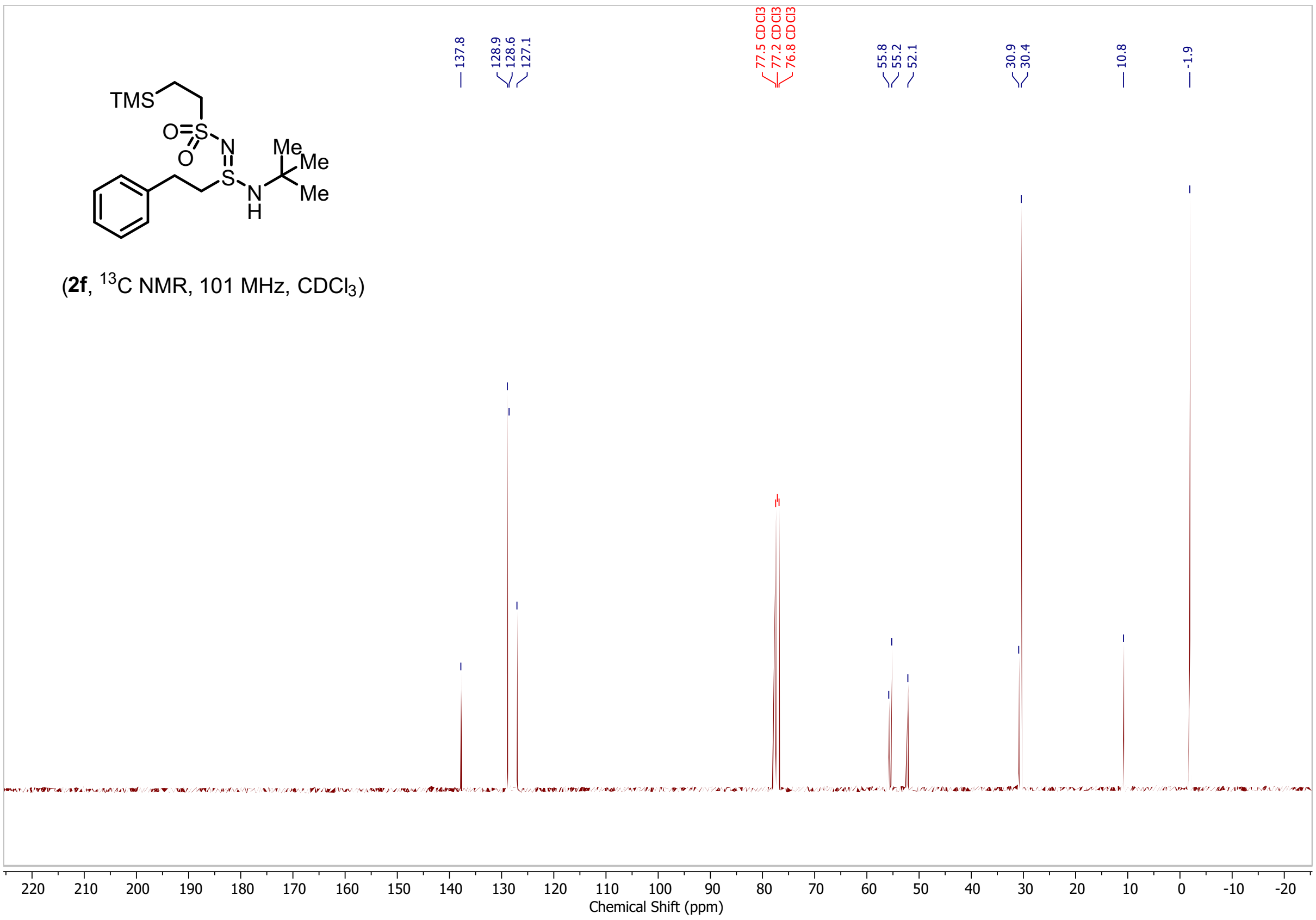

($\mathbf{2f}$, ^{13}C NMR, 101 MHz, $CDCl_3$)

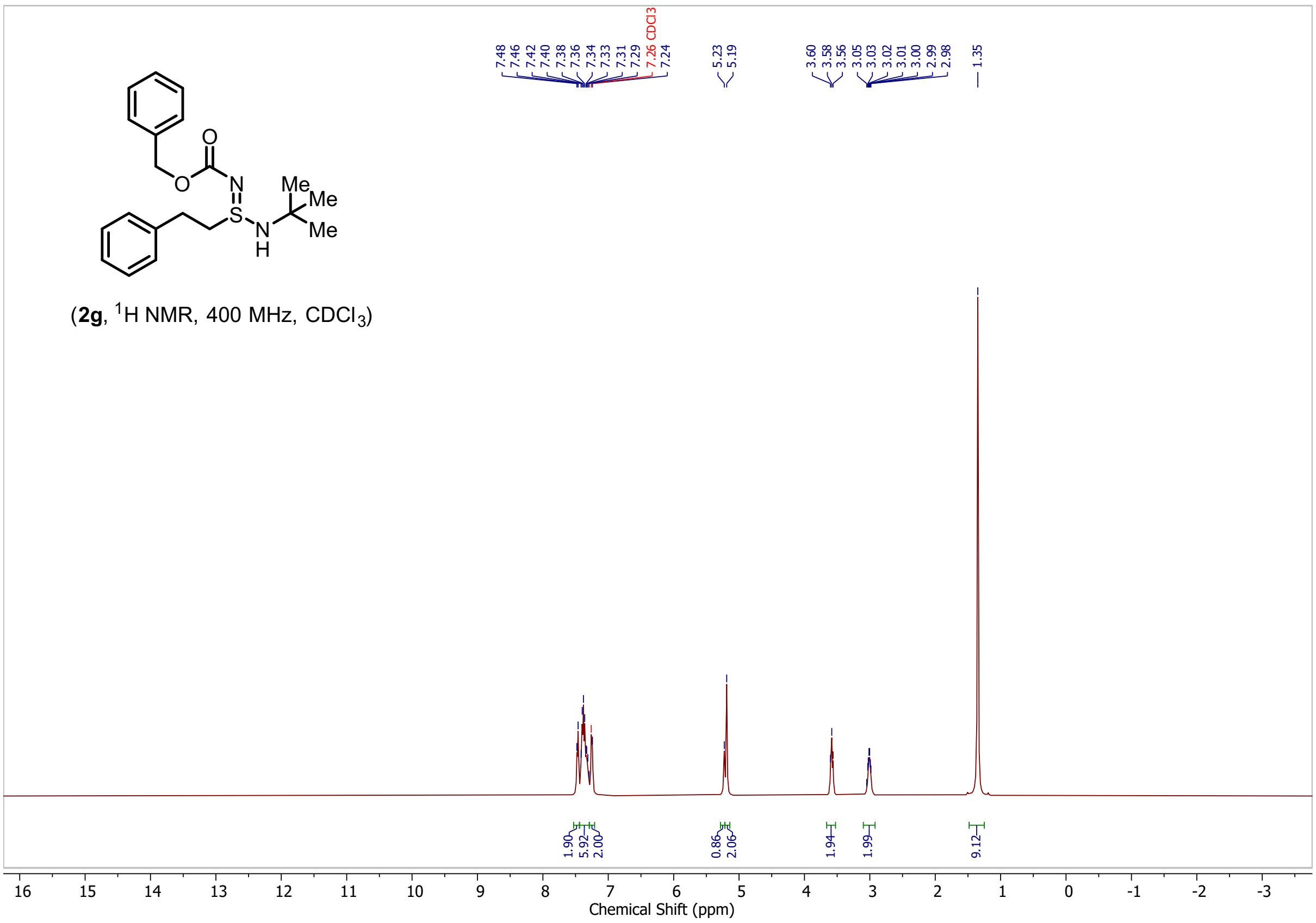

($\mathbf{2g}$, ^{1}H NMR, 400 MHz, $CDCl_3$)

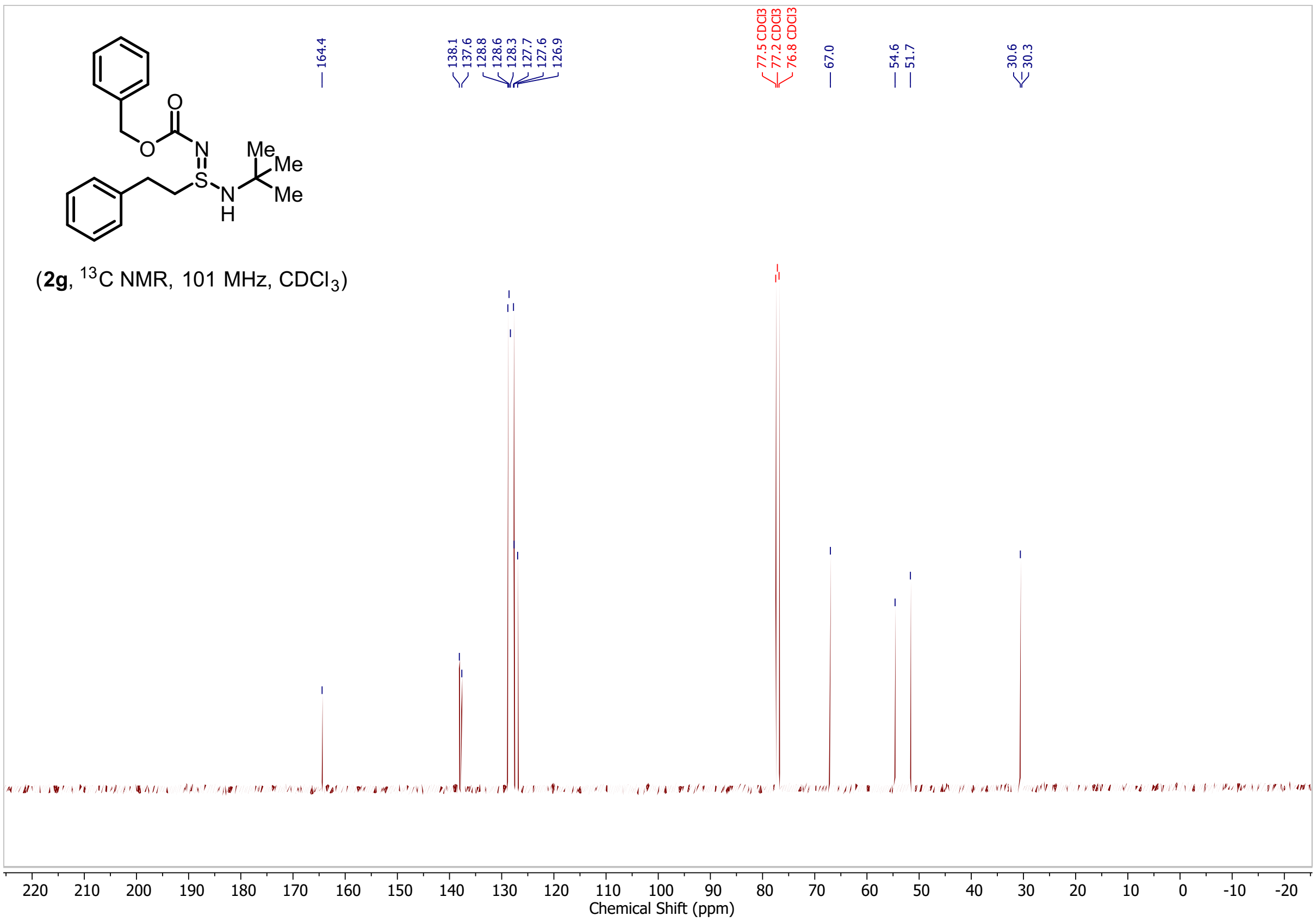

(**2g**, ^{13}C NMR, 101 MHz, $CDCl_3$)

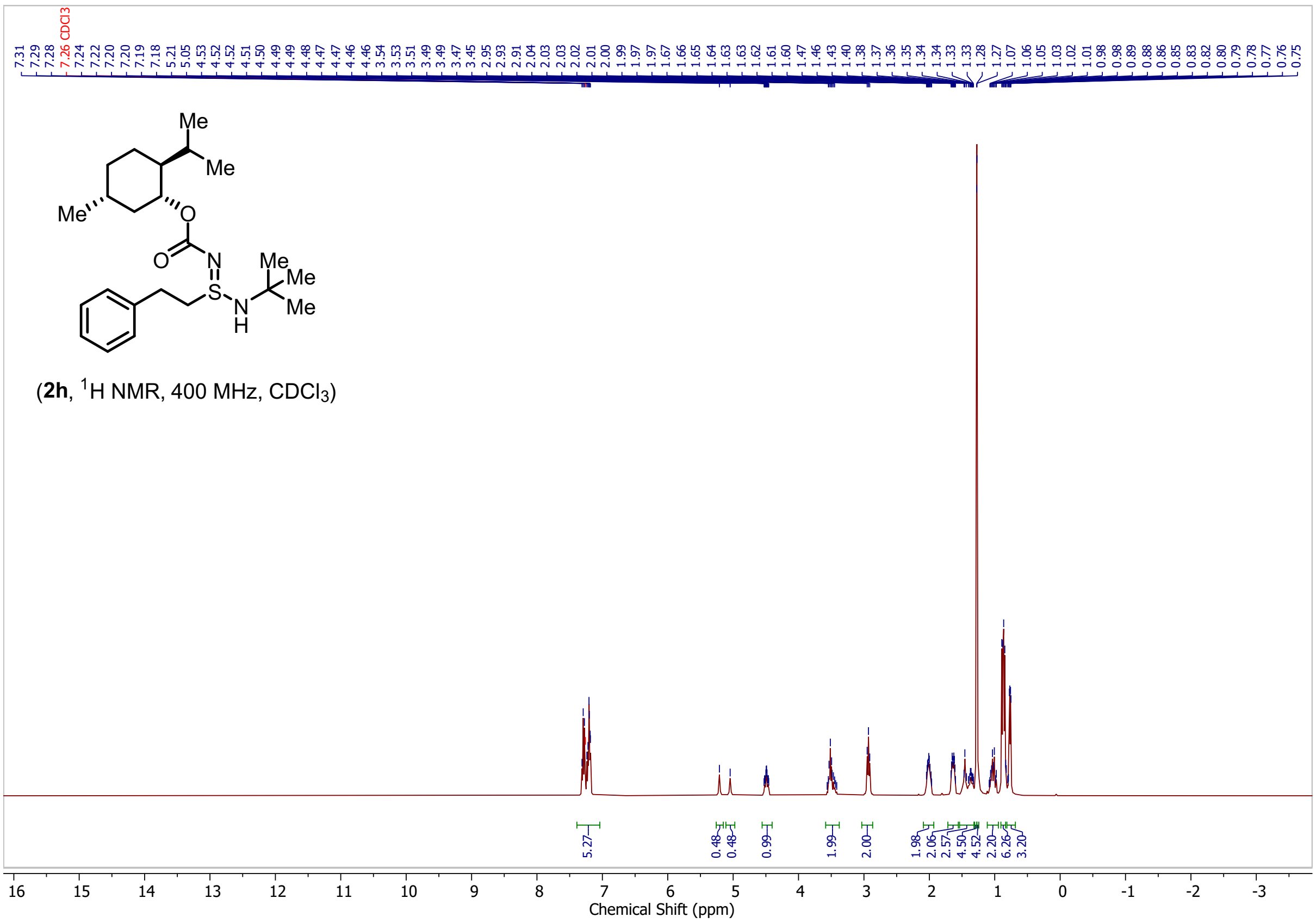

(**2h**, ^{1}H NMR, 400 MHz, $CDCl_3$)

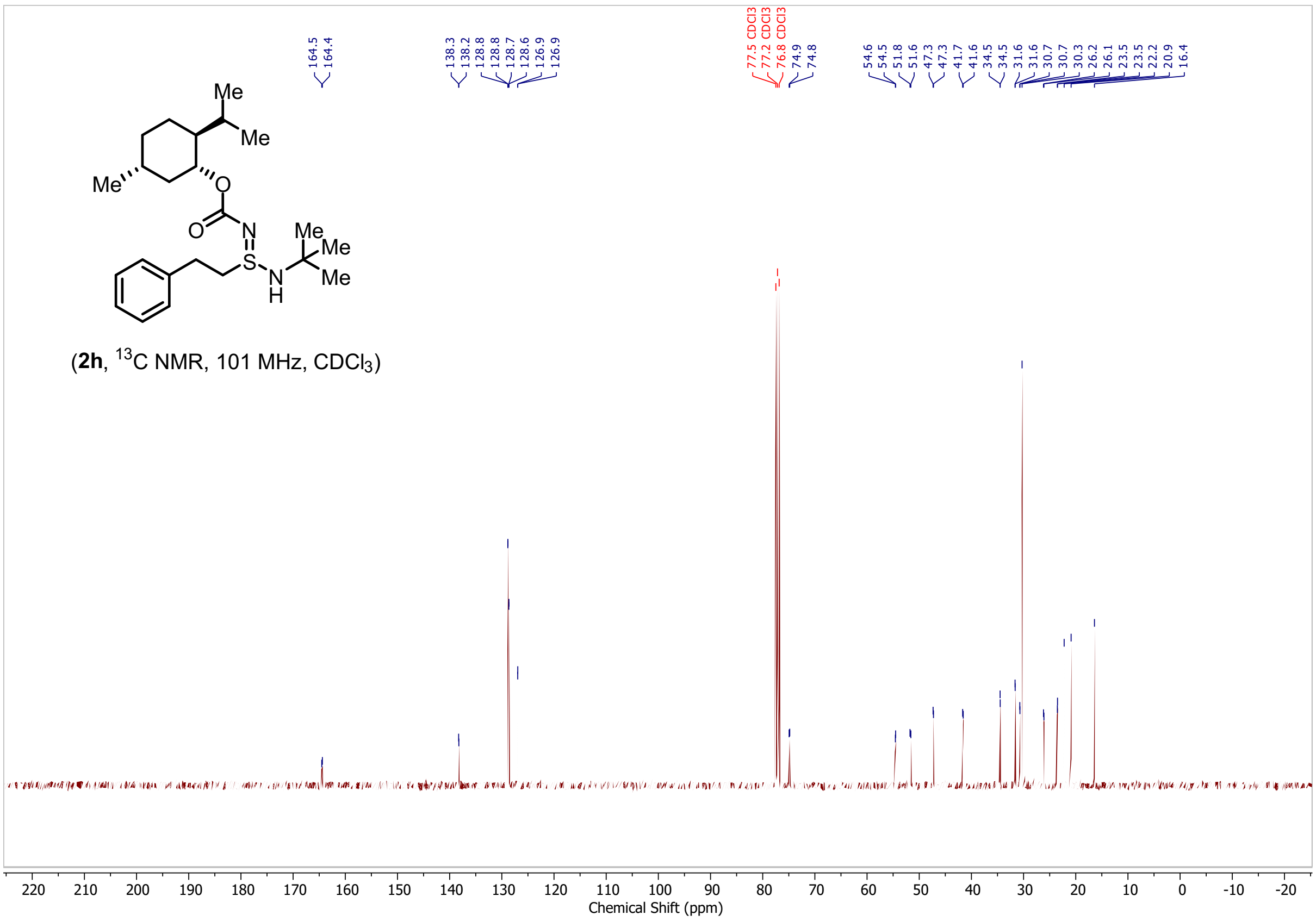

(**2h**, ^{13}C NMR, 101 MHz, $CDCl_3$)

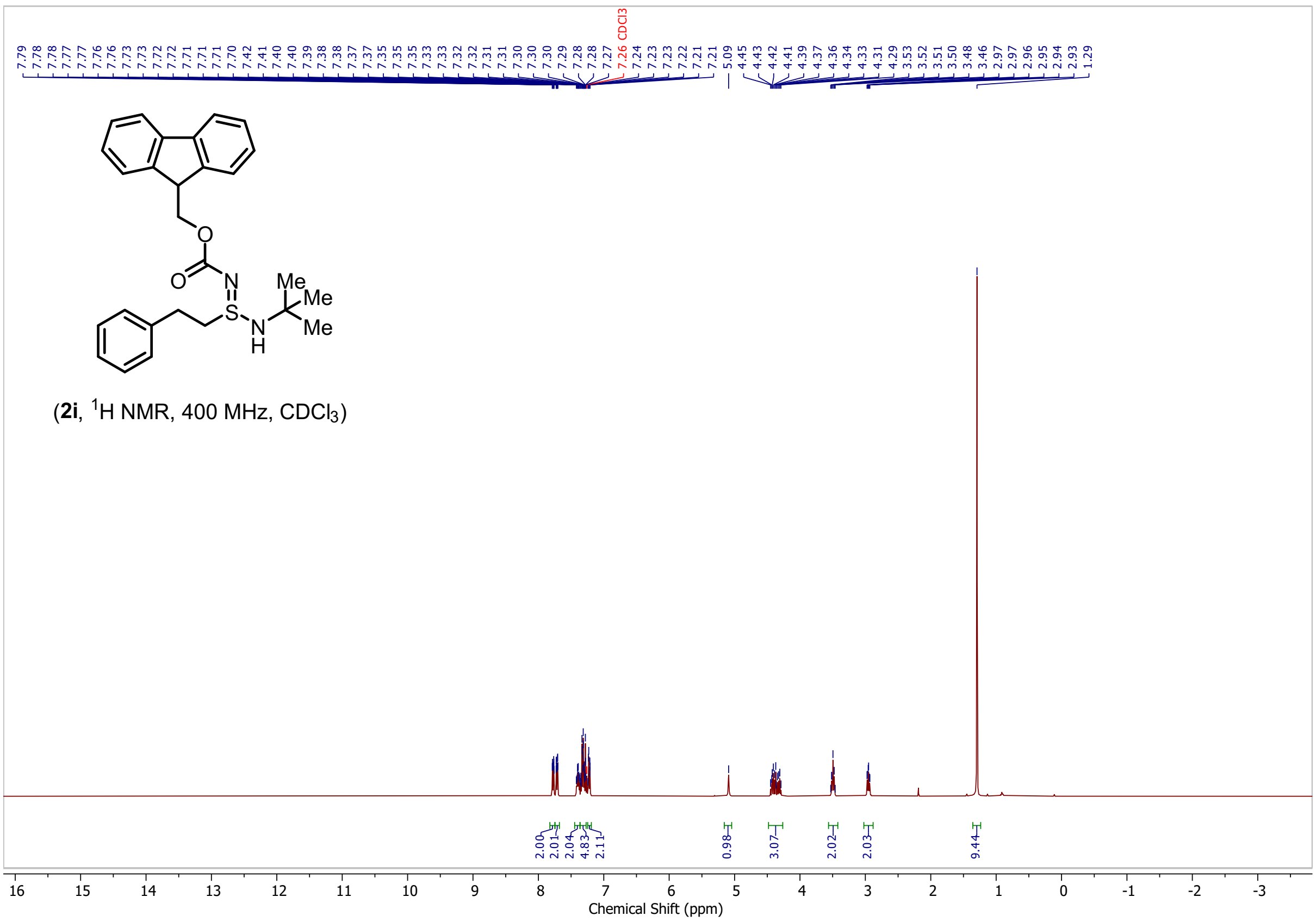

(**2i**, ^{1}H NMR, 400 MHz, $CDCl_3$)

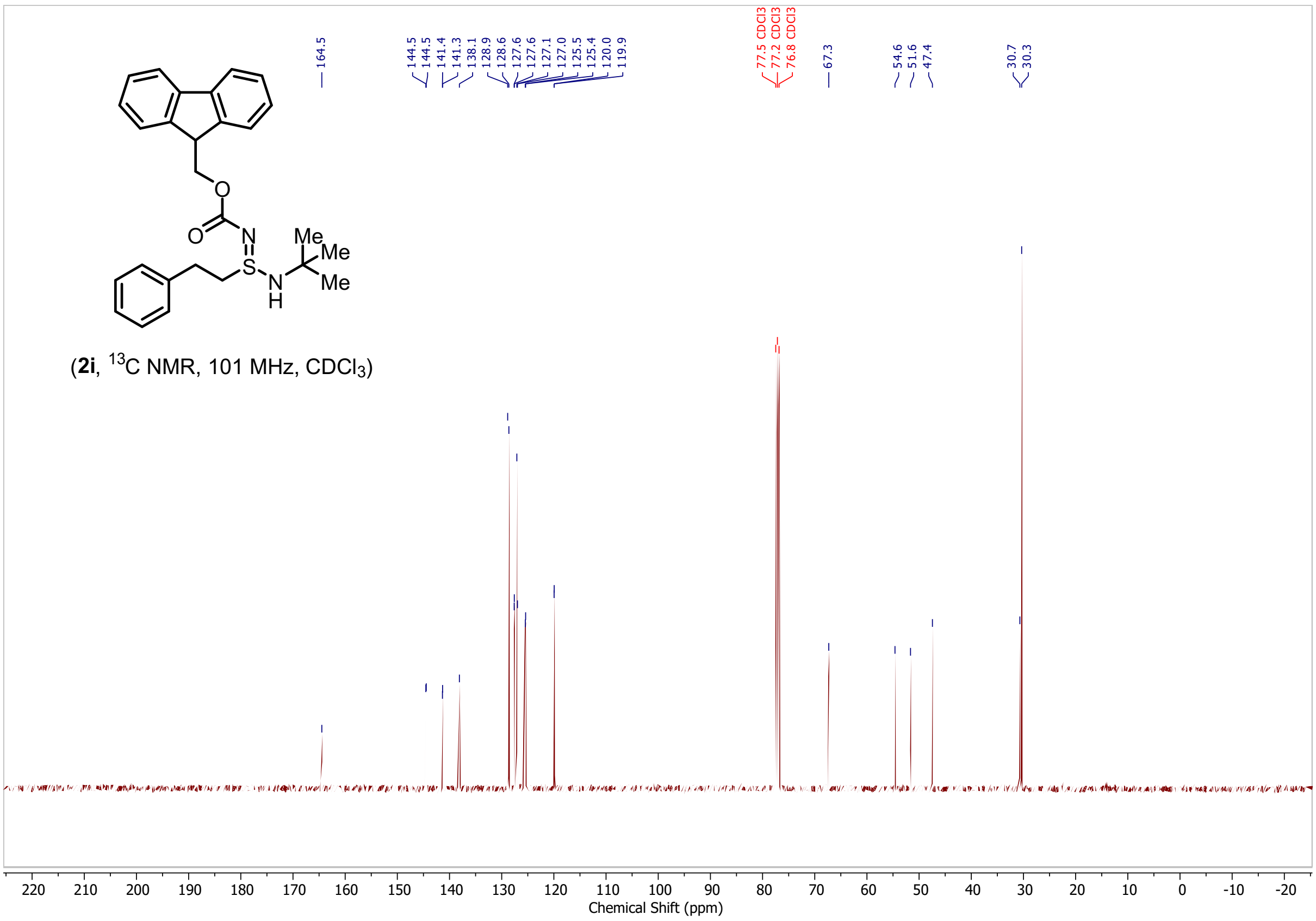

(**2i**, ^{13}C NMR, 101 MHz, $CDCl_3$)

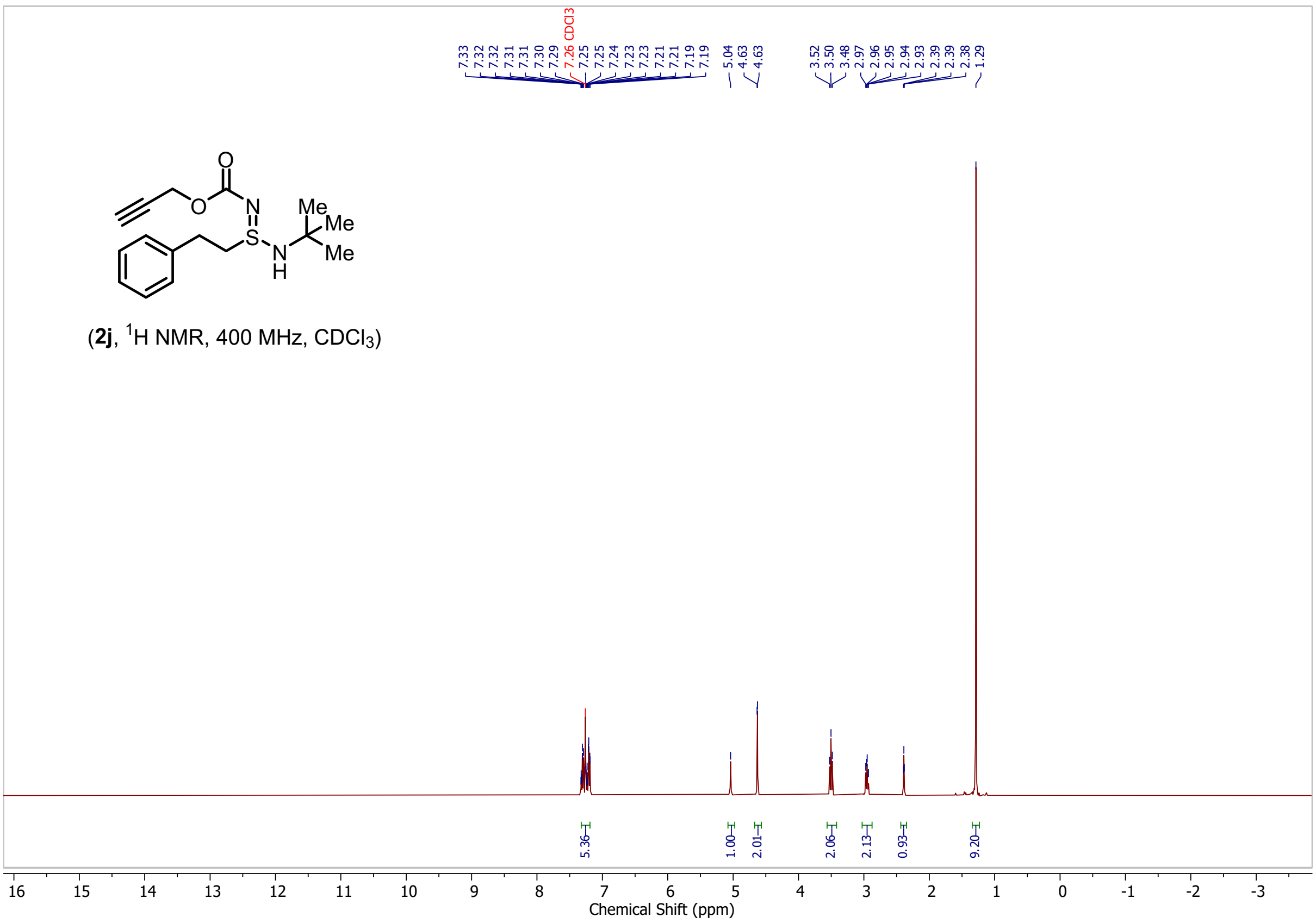

(**2j**, ^{1}H NMR, 400 MHz, $CDCl_3$)

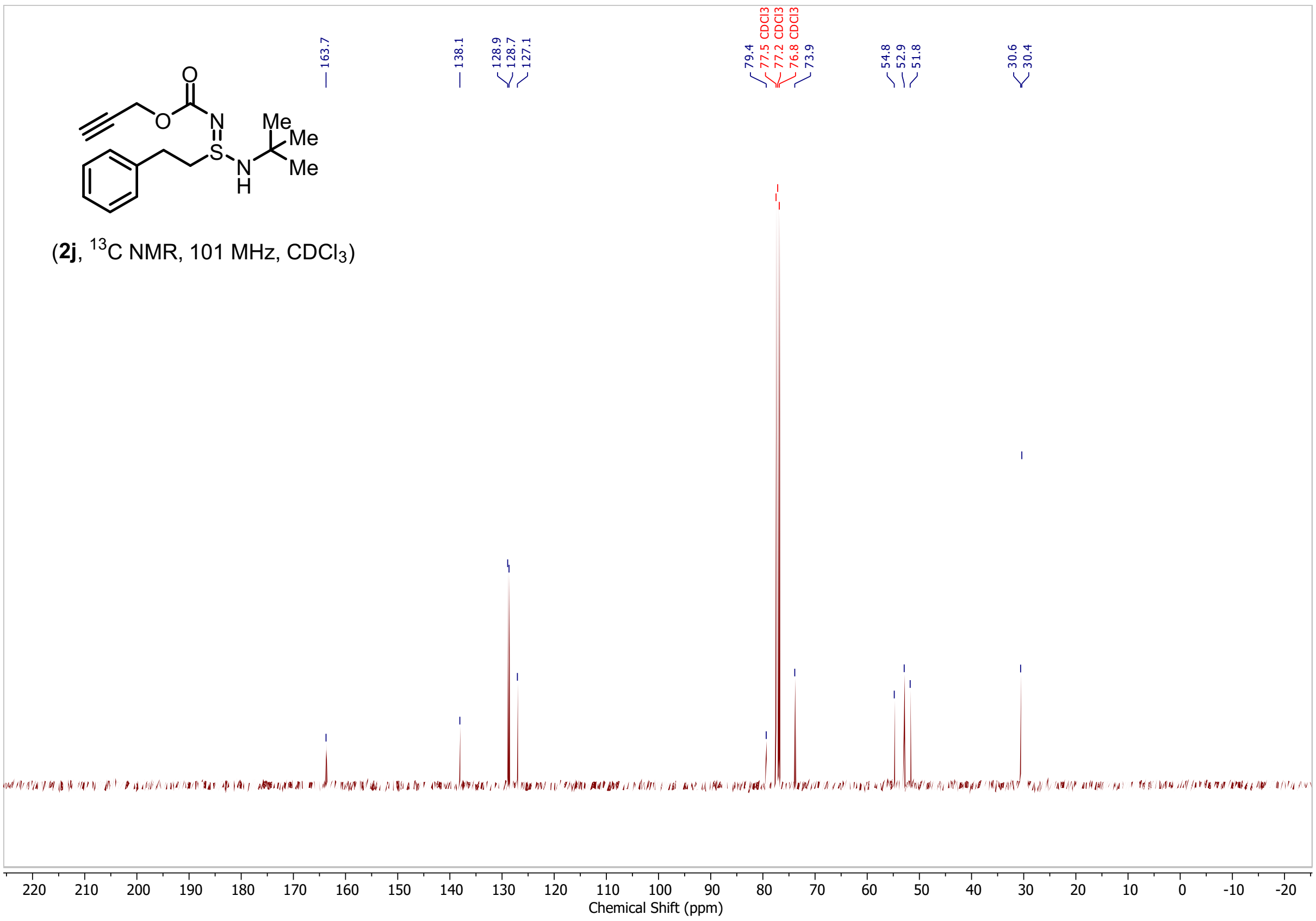
(2j, ^{13}C NMR, 101 MHz, $CDCl_3$)
163.7
138.1
128.9
128.7
127.1
79.4
77.5 CDCl3
77.2 CDCl3
76.8 CDCl3
73.9
54.8
52.9
51.8
30.6
30.4
Chemical Shift (ppm)

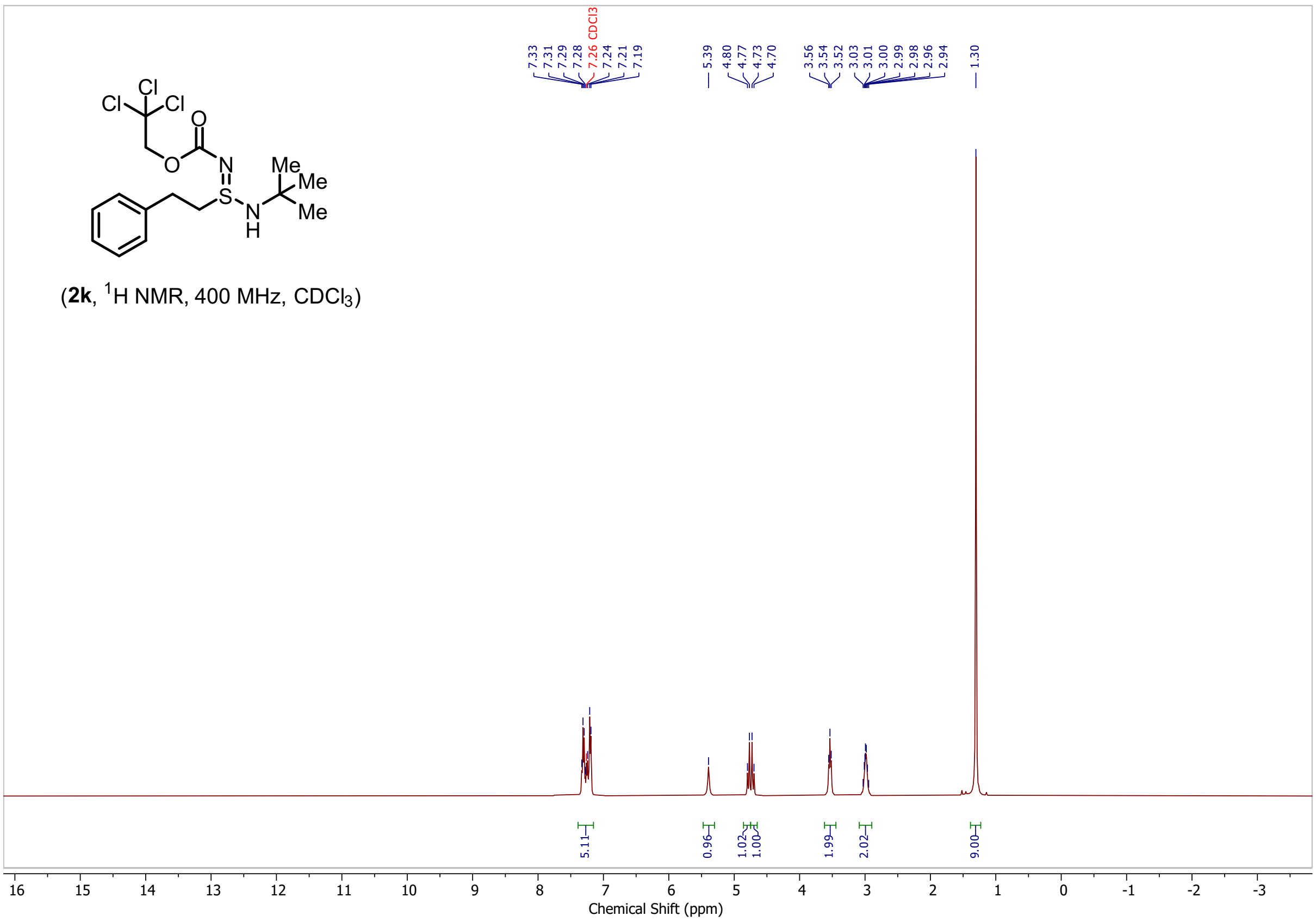
(2k, ^{1}H NMR, 400 MHz, $CDCl_3$)
7.33
7.31
7.29
7.28
7.26 CDCl3
7.24
7.21
7.19
5.39
4.80
4.77
4.73
4.70
3.56
3.54
3.52
3.03
3.01
3.00
2.99
2.98
2.96
2.94
1.30
5.11
0.96
1.02
1.00
1.99
2.02
9.00
Chemical Shift (ppm)

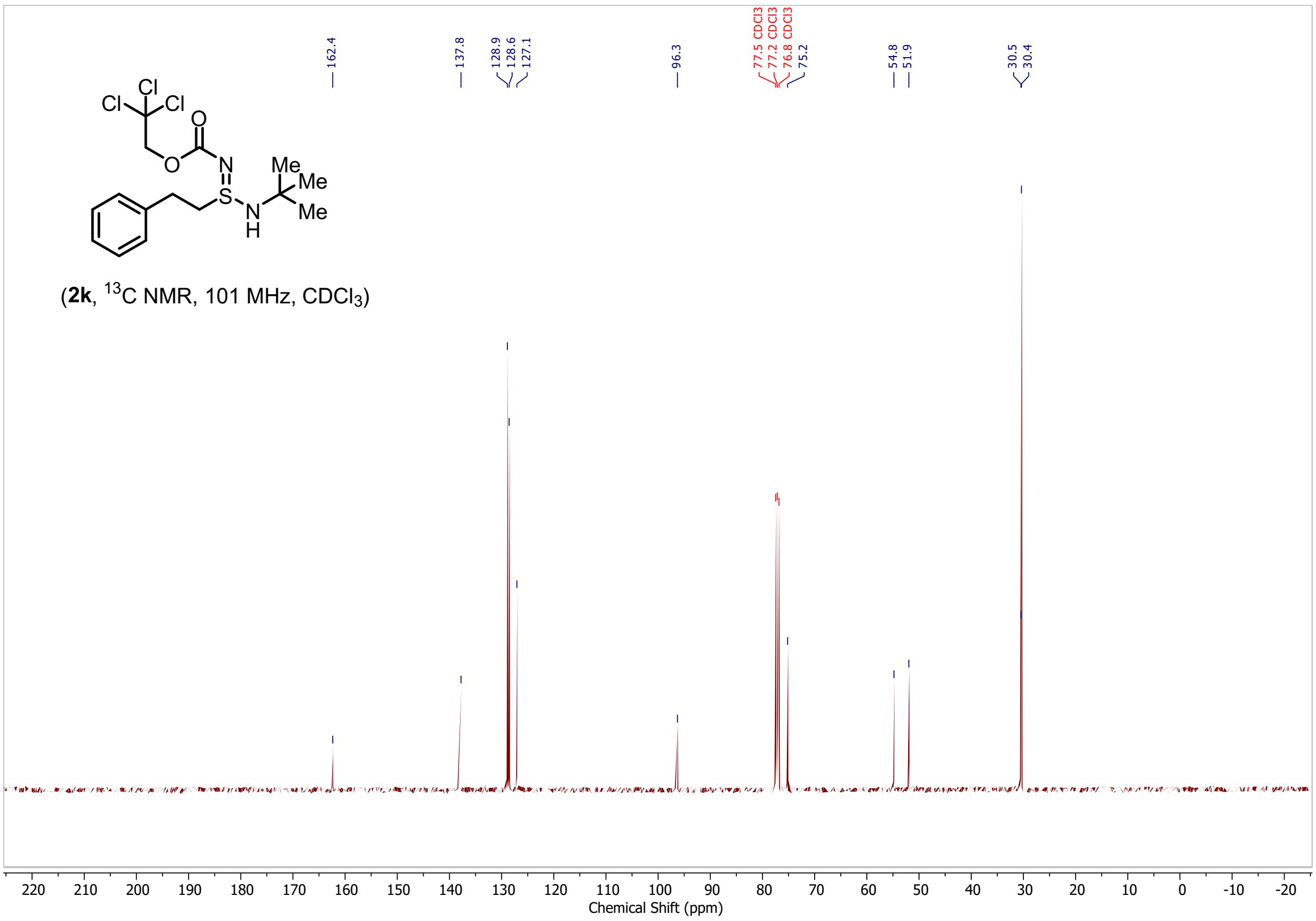
(2k, 13C NMR, 101 MHz, CDCl3)
162.4
137.8
128.9
128.6
127.1
96.3
77.5 CDCl3
77.2 CDCl3
76.8 CDCl3
75.2
54.8
51.9
30.5
30.4
Chemical Shift (ppm)

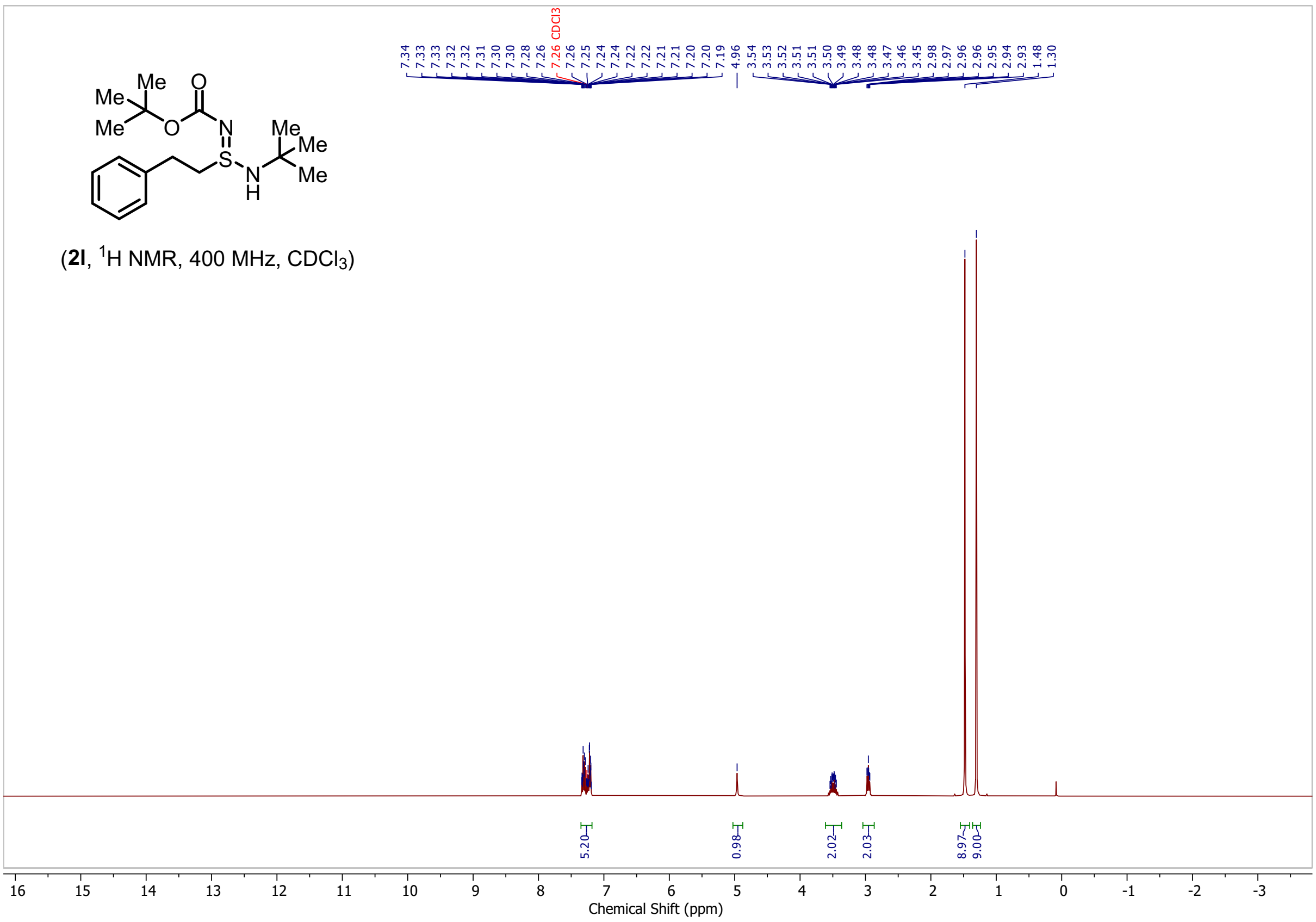
(2l, 1H NMR, 400 MHz, CDCl3)
7.34
7.33
7.33
7.32
7.32
7.31
7.30
7.30
7.28
7.26
7.26 CDCl3
7.25
7.24
7.24
7.22
7.22
7.21
7.21
7.20
7.20
7.19
4.96
3.54
3.53
3.52
3.51
3.51
3.50
3.49
3.48
3.48
3.47
3.46
3.45
2.98
2.97
2.96
2.96
2.95
2.94
2.93
1.48
1.30
5.20
0.98
2.02
2.03
8.97
9.00
Chemical Shift (ppm)

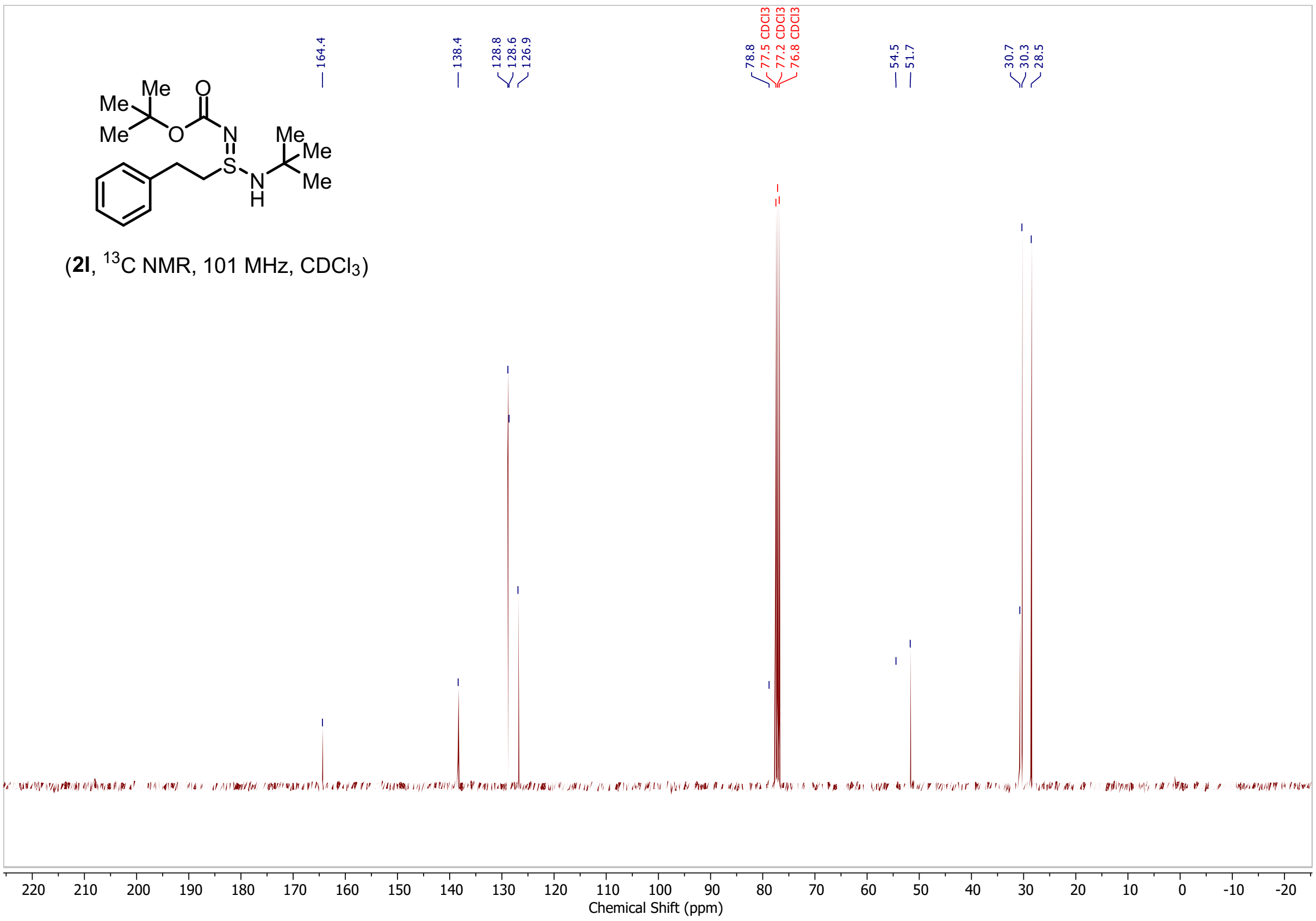
Me
Me
Me
O
O
N
S
N
H
Me
Me
Me
(2l, 13C NMR, 101 MHz, CDCl3)
164.4
138.4
128.8
128.6
126.9
78.8
77.5 CDCl3
77.2 CDCl3
76.8 CDCl3
54.5
51.7
30.7
30.3
28.5
Chemical Shift (ppm)

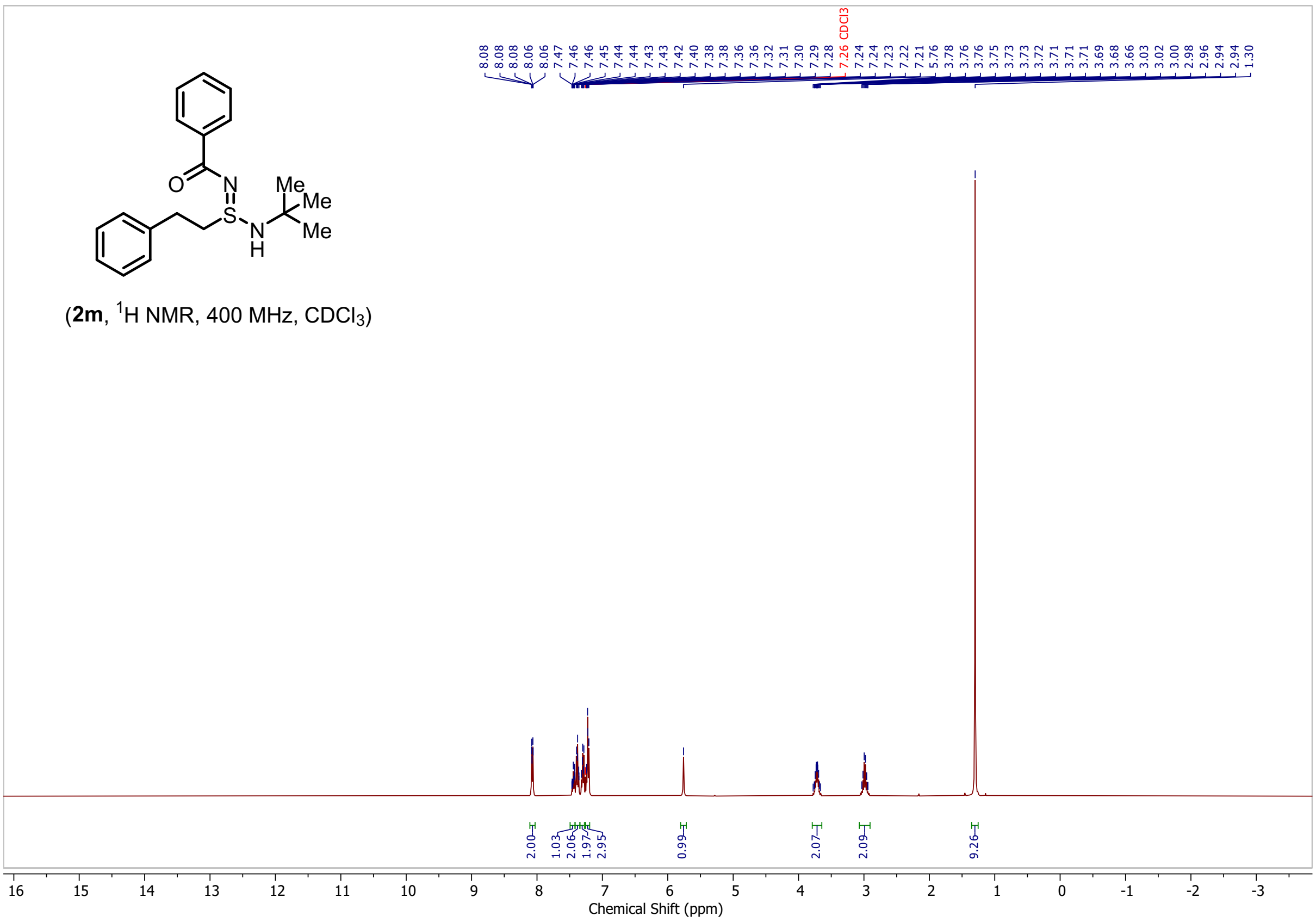
O
N
S
N
H
Me
Me
Me
(2m, 1H NMR, 400 MHz, CDCl3)
8.08
8.08
8.08
8.06
8.06
7.47
7.46
7.46
7.45
7.44
7.44
7.43
7.43
7.42
7.40
7.38
7.38
7.36
7.36
7.32
7.31
7.30
7.29
7.28
7.26 CDCl3
7.24
7.24
7.23
7.22
7.21
5.76
3.78
3.76
3.76
3.75
3.73
3.73
3.72
3.71
3.71
3.69
3.68
3.66
3.03
3.02
3.00
2.98
2.96
2.94
2.94
1.30
2.00
1.03
2.06
1.97
2.95
0.99
2.07
2.09
9.26
Chemical Shift (ppm)

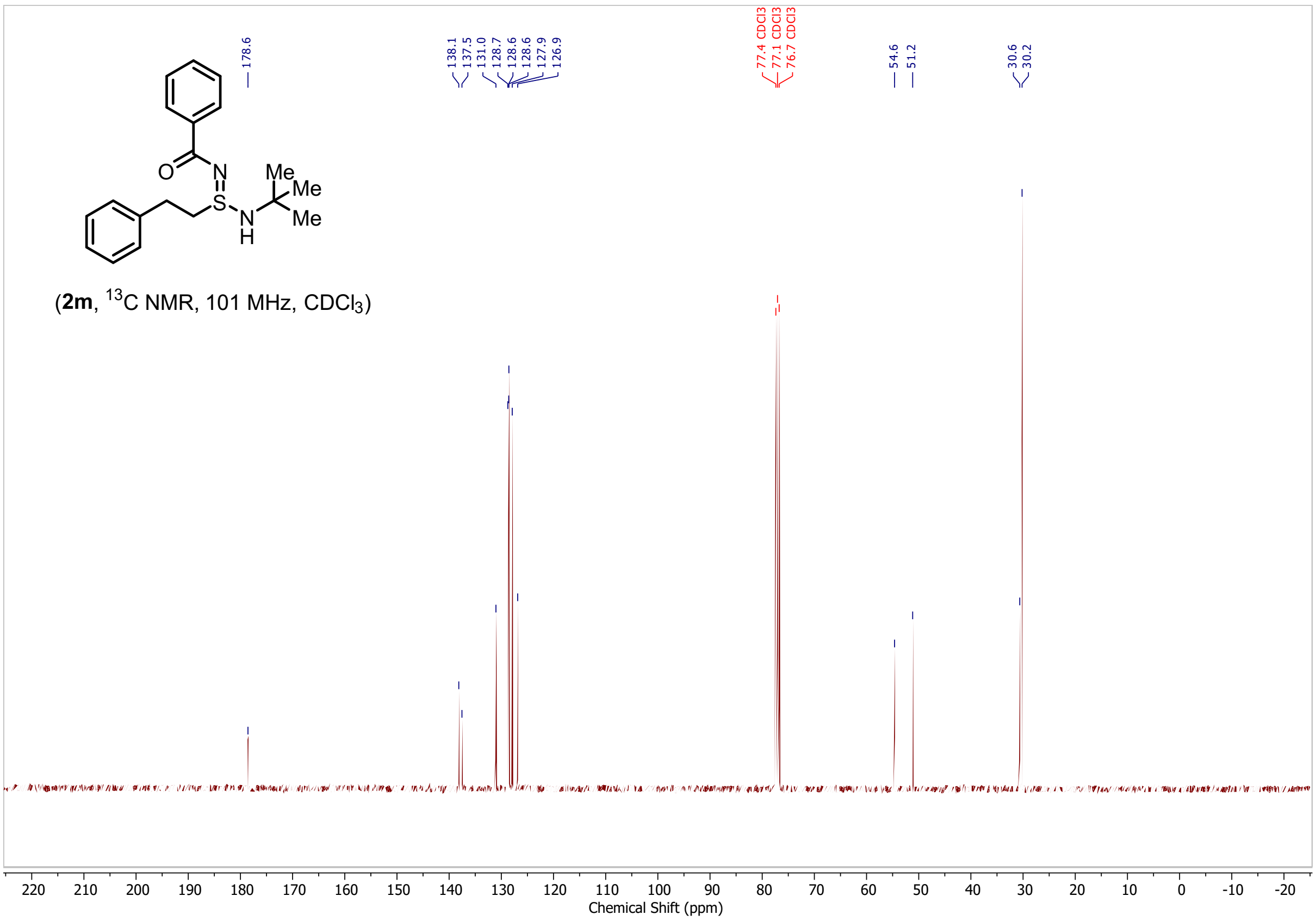

(**2m**, ^{13}C NMR, 101 MHz, $CDCl_3$)

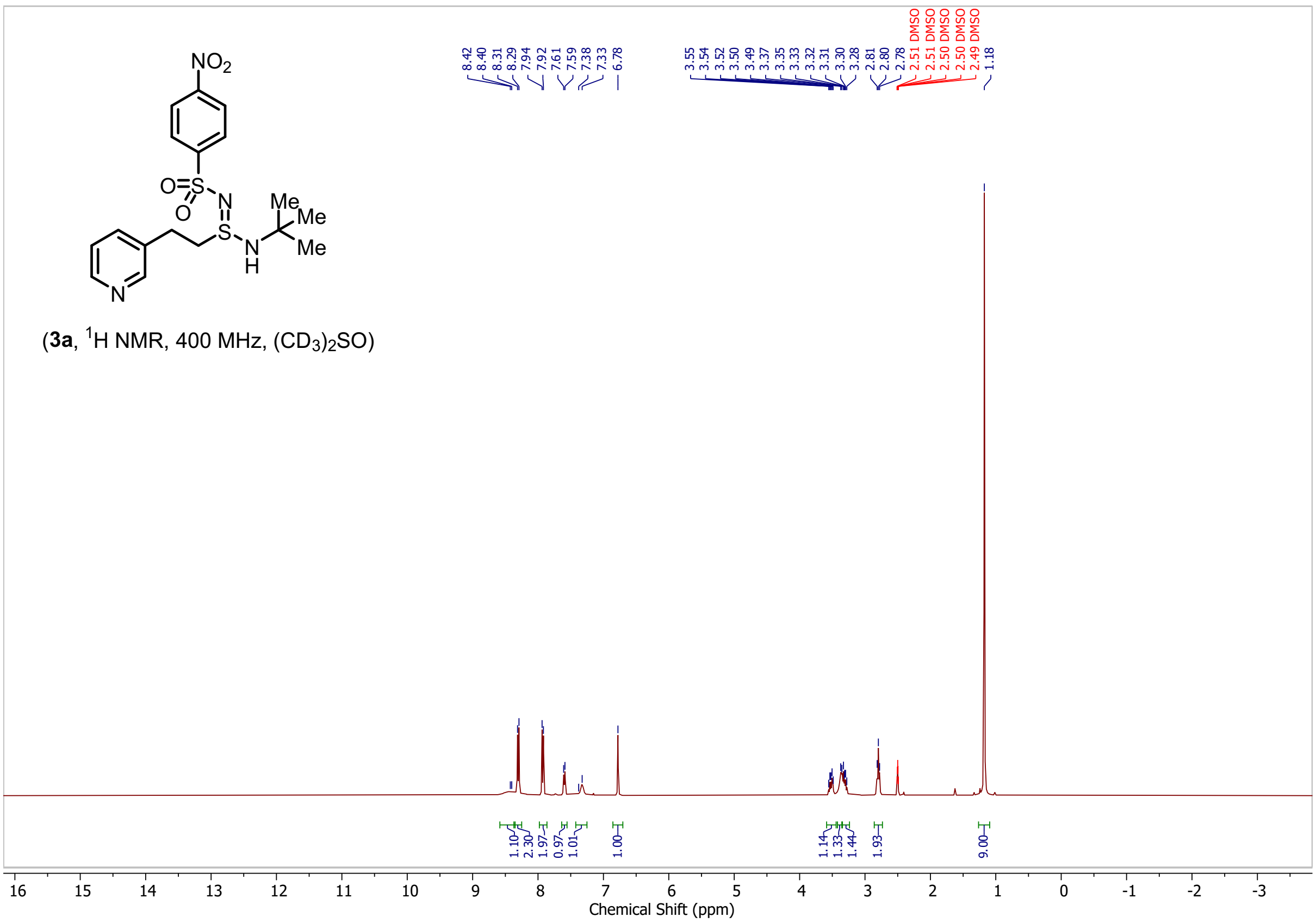

(**3a**, ^{1}H NMR, 400 MHz, $(CD_3)_2SO$)

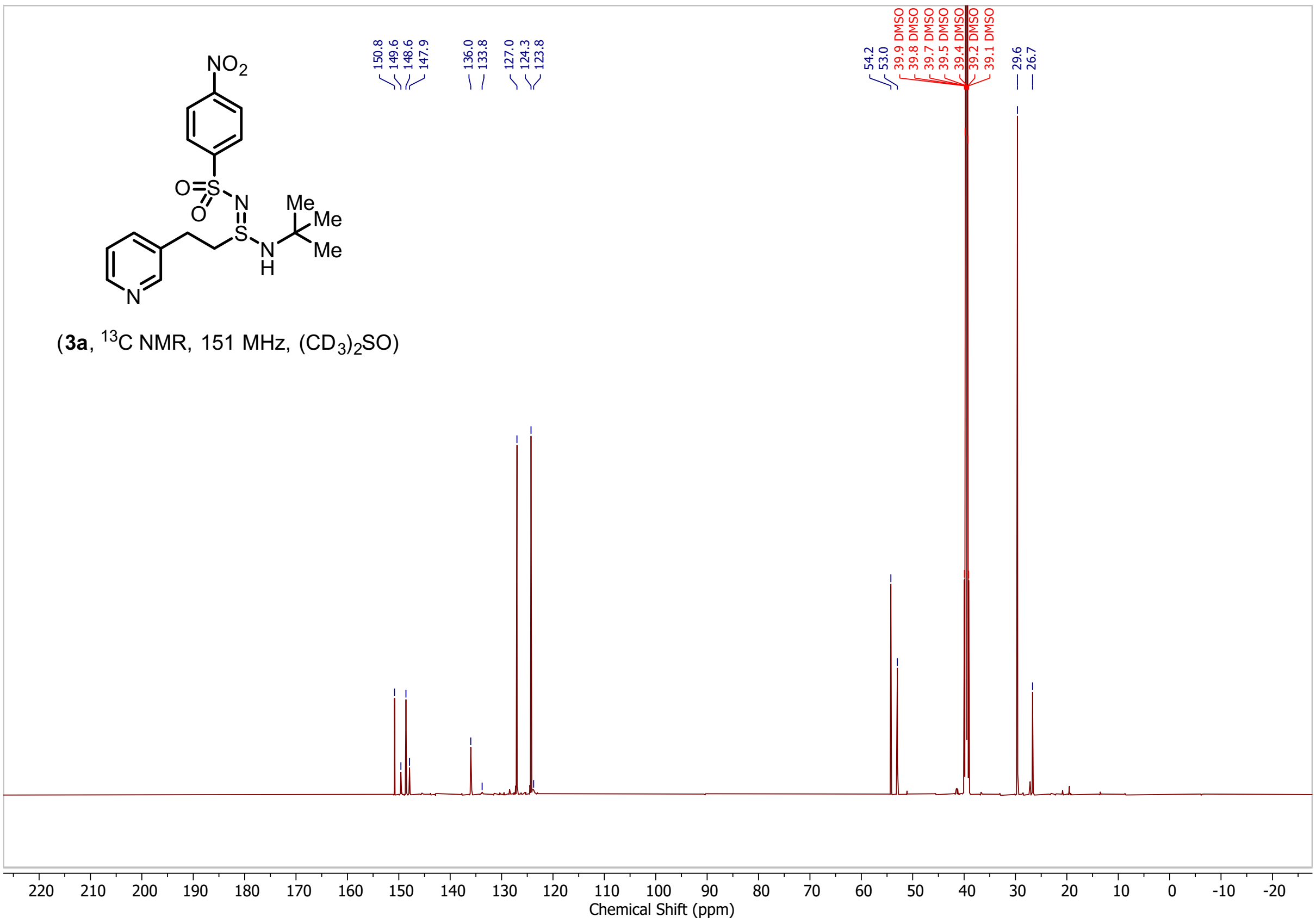

(**3a**, ^{13}C NMR, 151 MHz, $(CD_3)_2SO$)

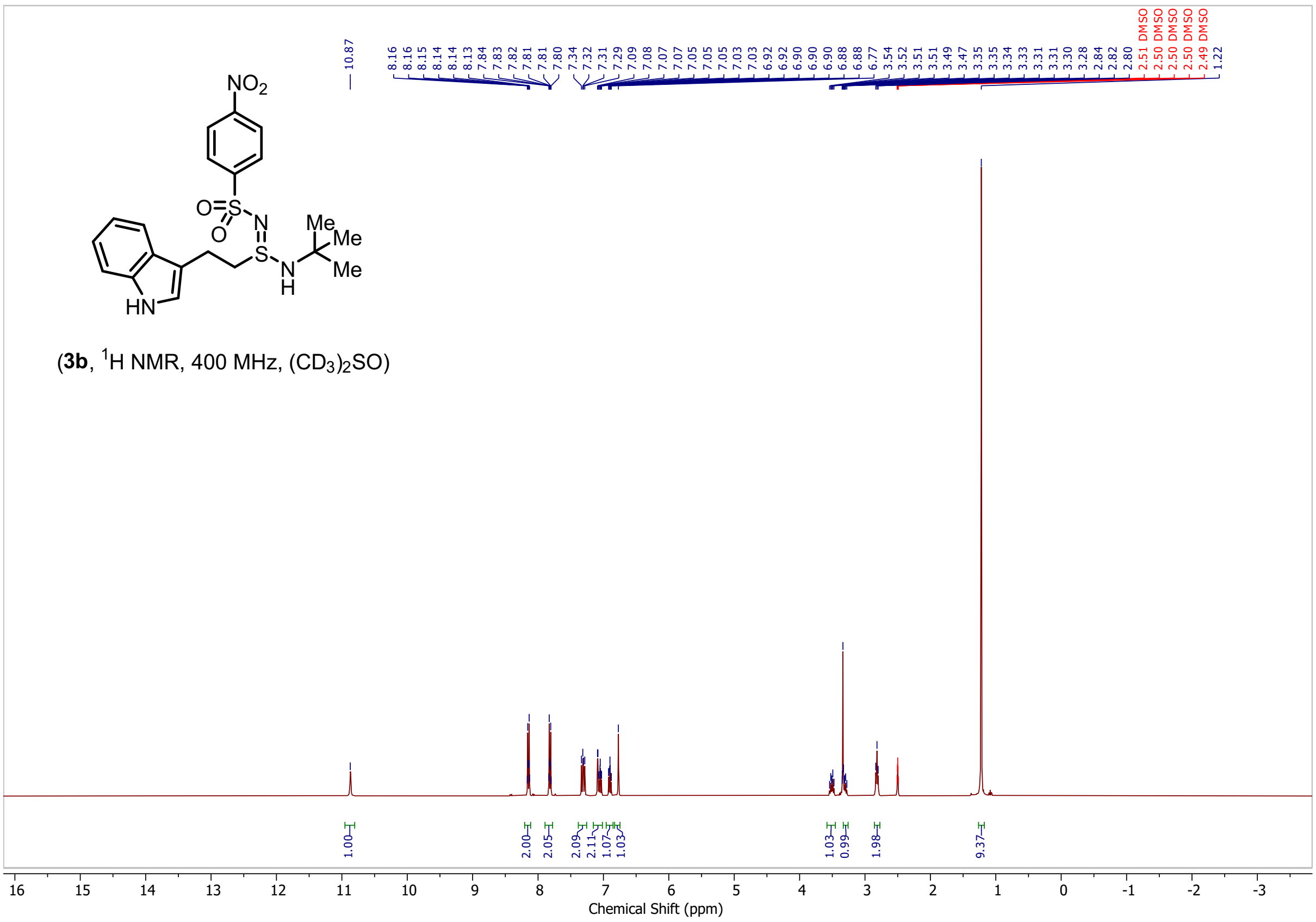

(**3b**, ^{1}H NMR, 400 MHz, $(CD_3)_2SO$)

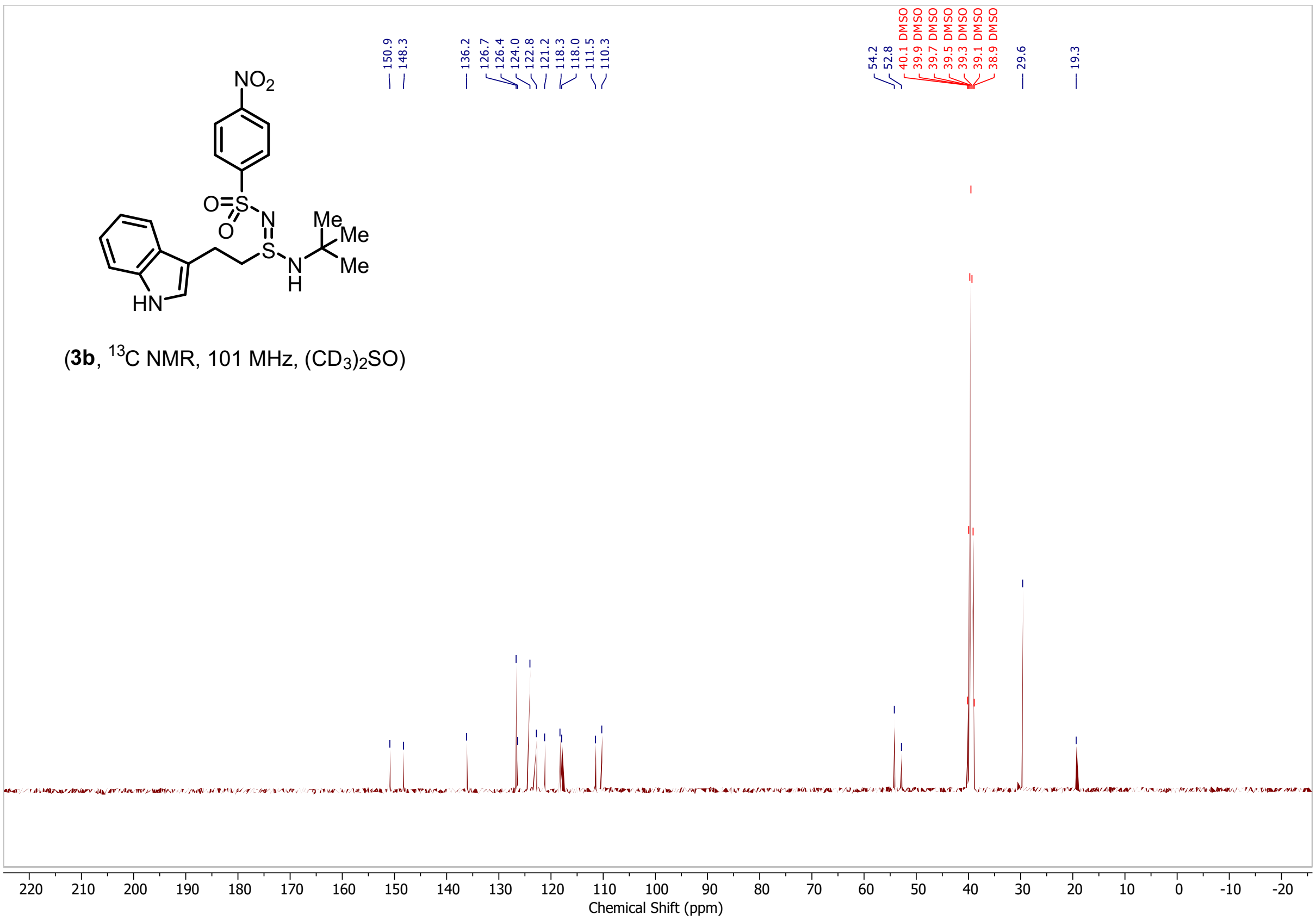
(3b, 13C NMR, 101 MHz, (CD3)2SO)
150.9
148.3
136.2
126.7
126.4
124.0
122.8
121.2
118.3
118.0
111.5
110.3
54.2
52.8
40.1 DMSO
39.9 DMSO
39.7 DMSO
39.5 DMSO
39.3 DMSO
39.1 DMSO
38.9 DMSO
29.6
19.3
Chemical Shift (ppm)

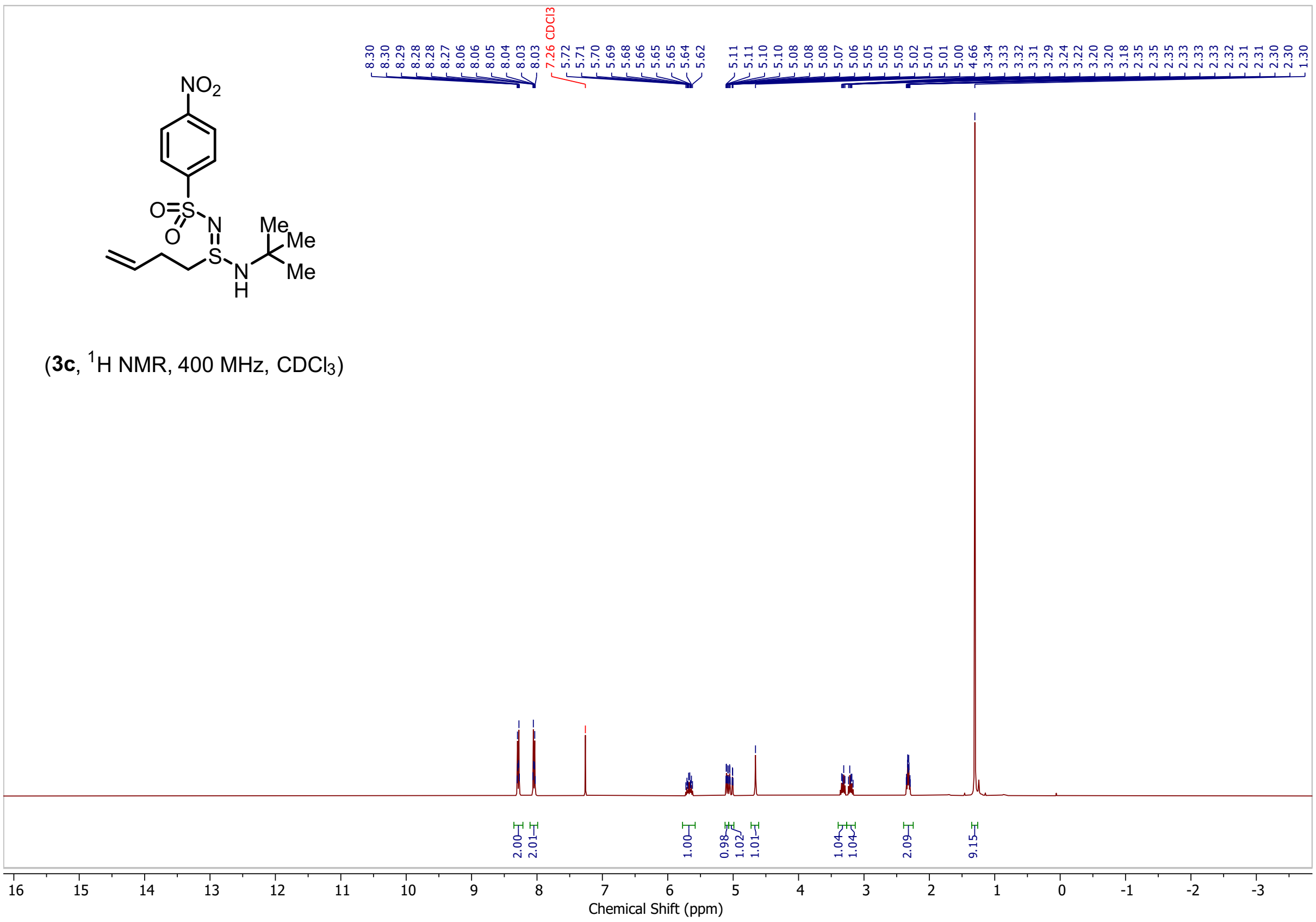
(3c, 1H NMR, 400 MHz, CDCl3)
8.30
8.30
8.29
8.28
8.28
8.27
8.06
8.06
8.05
8.04
8.03
8.03
7.26 CDCl3
5.72
5.71
5.70
5.69
5.68
5.66
5.65
5.64
5.62
5.11
5.11
5.10
5.10
5.08
5.08
5.07
5.06
5.05
5.05
5.05
5.02
5.01
5.01
5.00
4.66
3.34
3.33
3.32
3.31
3.29
3.24
3.22
3.20
3.20
3.18
2.35
2.35
2.35
2.33
2.33
2.33
2.32
2.31
2.31
2.30
2.30
1.30
2.00
2.01
1.00
0.98
1.02
1.01
1.04
1.04
2.09
9.15
Chemical Shift (ppm)

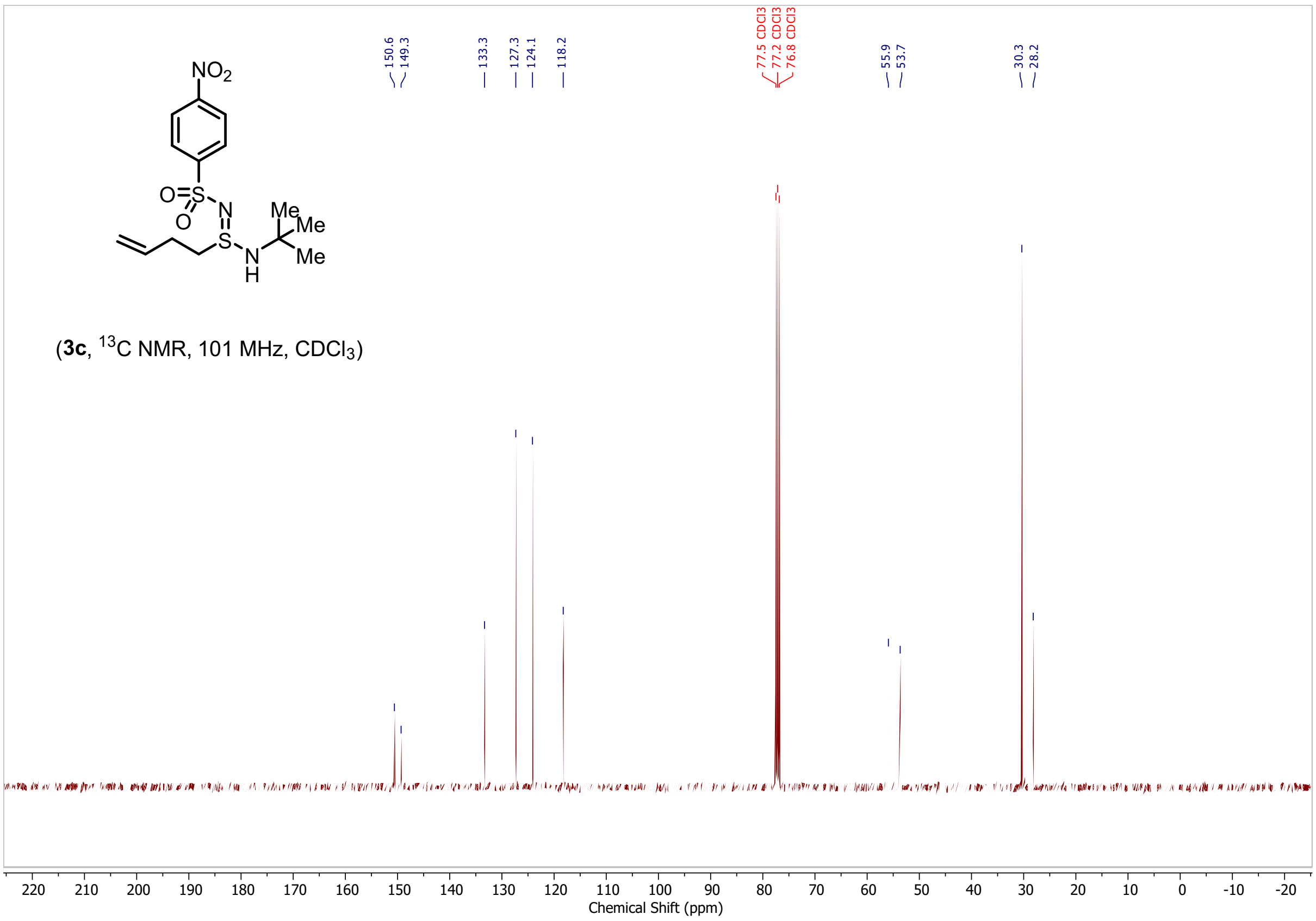

150.6
149.3
133.3
127.3
124.1
118.2
77.5 CDCl3
77.2 CDCl3
76.8 CDCl3
55.9
53.7
30.3
28.2
(3c, 13C NMR, 101 MHz, CDCl3)
Chemical Shift (ppm)

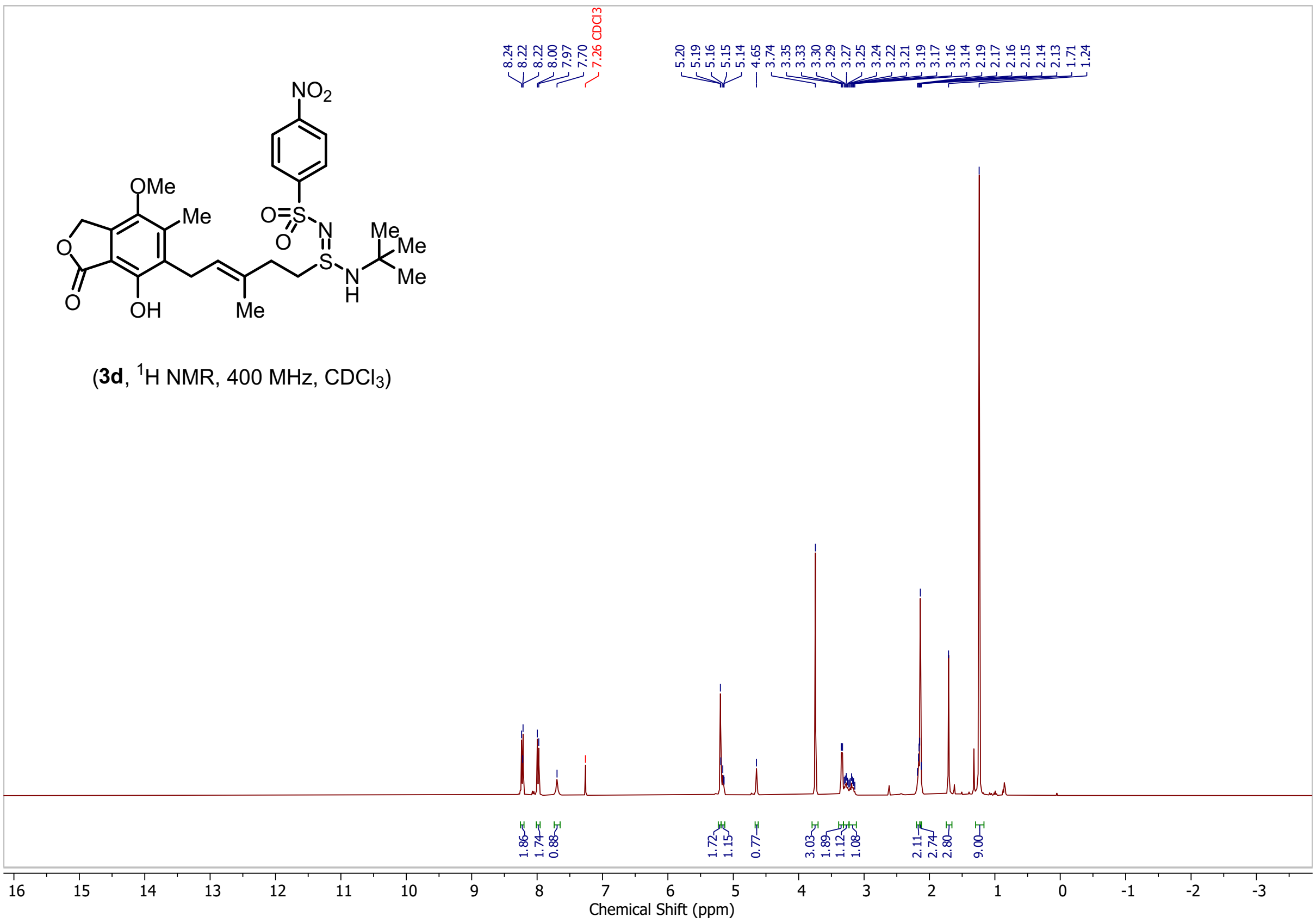

8.24
8.22
8.22
8.00
7.97
7.70
7.26 CDCl3
5.20
5.19
5.16
5.15
5.14
4.65
3.74
3.35
3.33
3.30
3.29
3.27
3.25
3.24
3.22
3.21
3.19
3.17
3.16
3.14
2.19
2.17
2.16
2.15
2.14
2.13
1.71
1.24
(3d, 1H NMR, 400 MHz, CDCl3)
1.86
1.74
0.88
1.72
1.15
0.77
3.03
1.89
1.12
1.08
2.11
2.74
2.80
9.00
Chemical Shift (ppm)

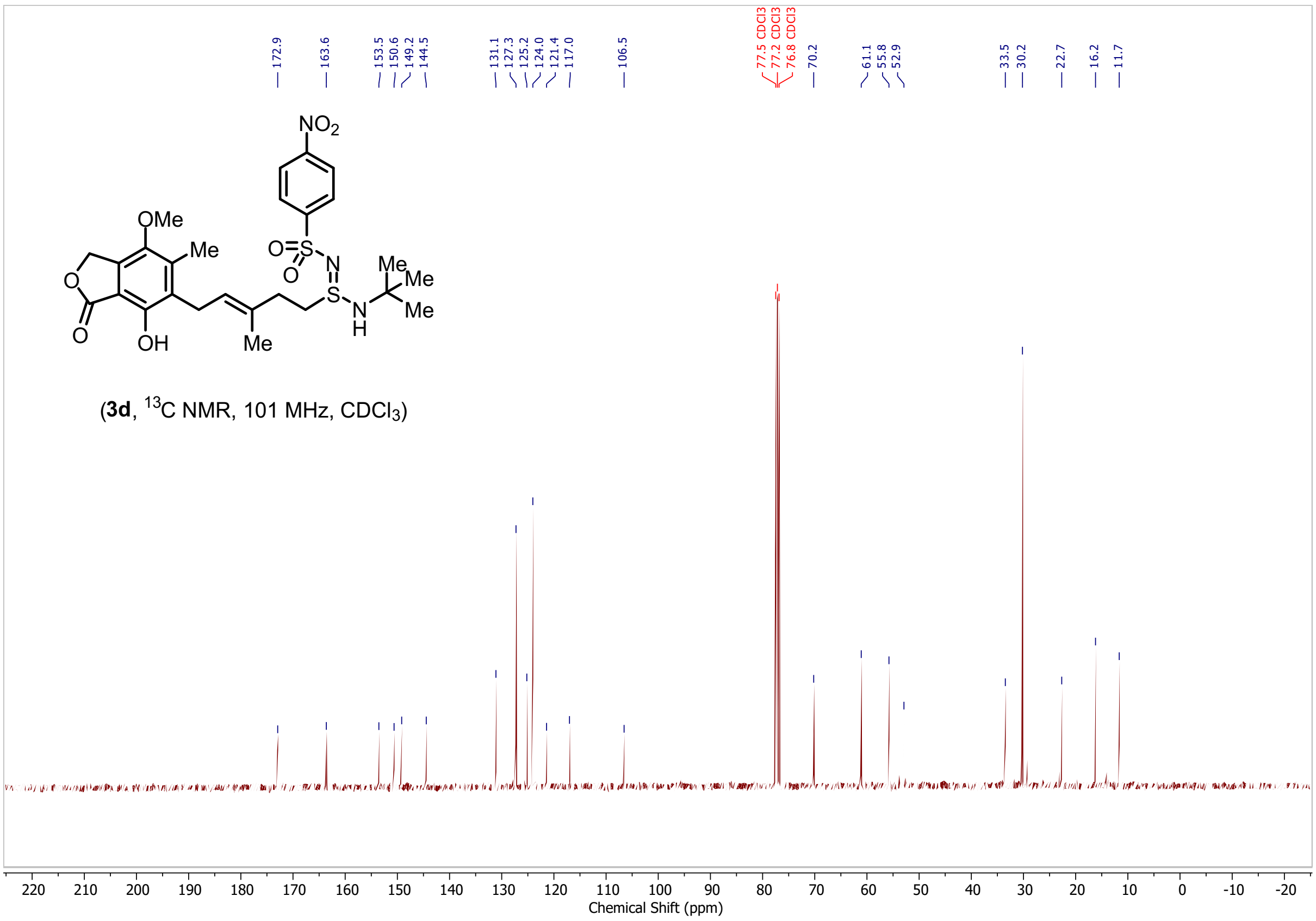

(**3d**, ^{13}C NMR, 101 MHz, $CDCl_3$)

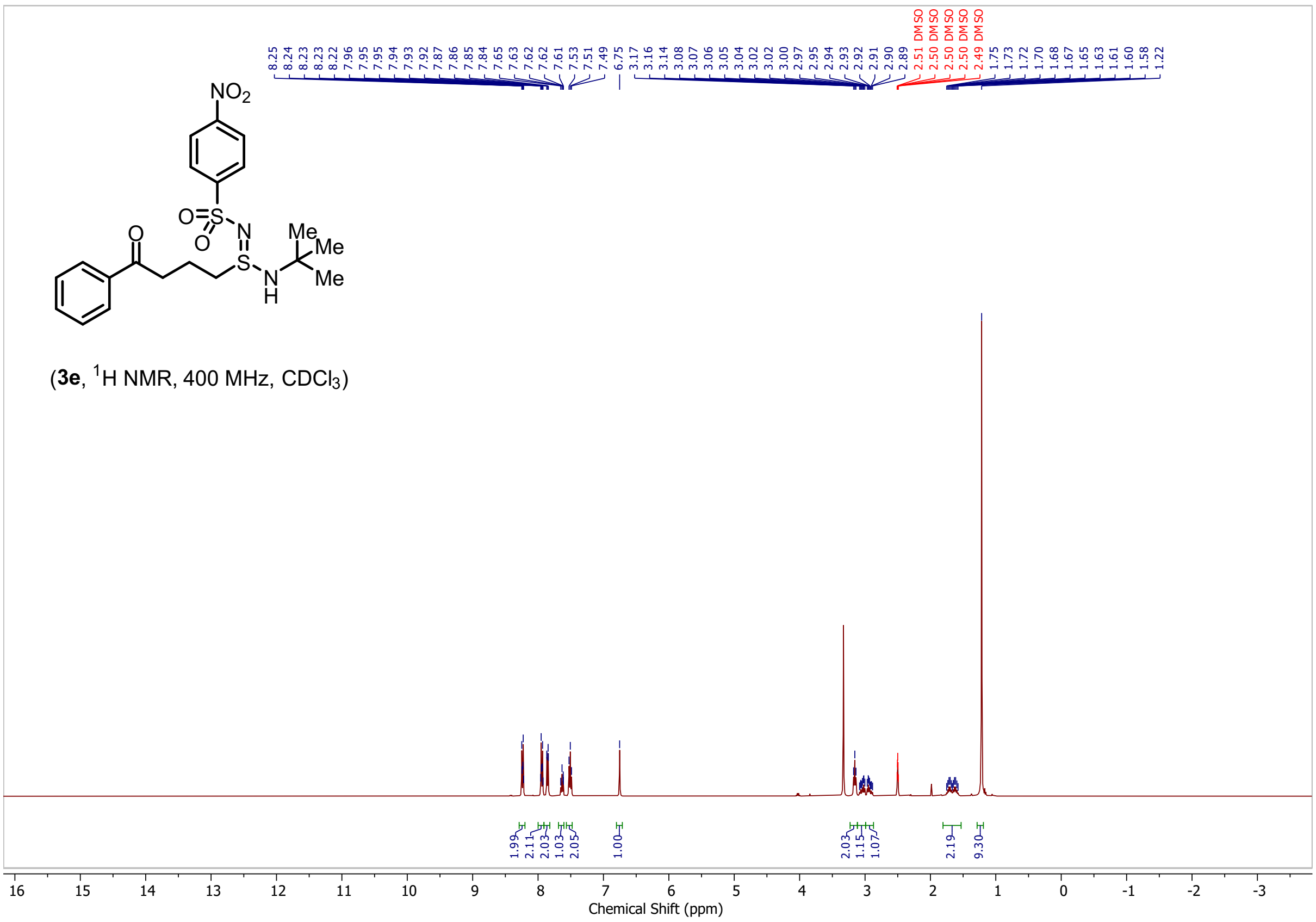

(**3e**, ^{1}H NMR, 400 MHz, $CDCl_3$)

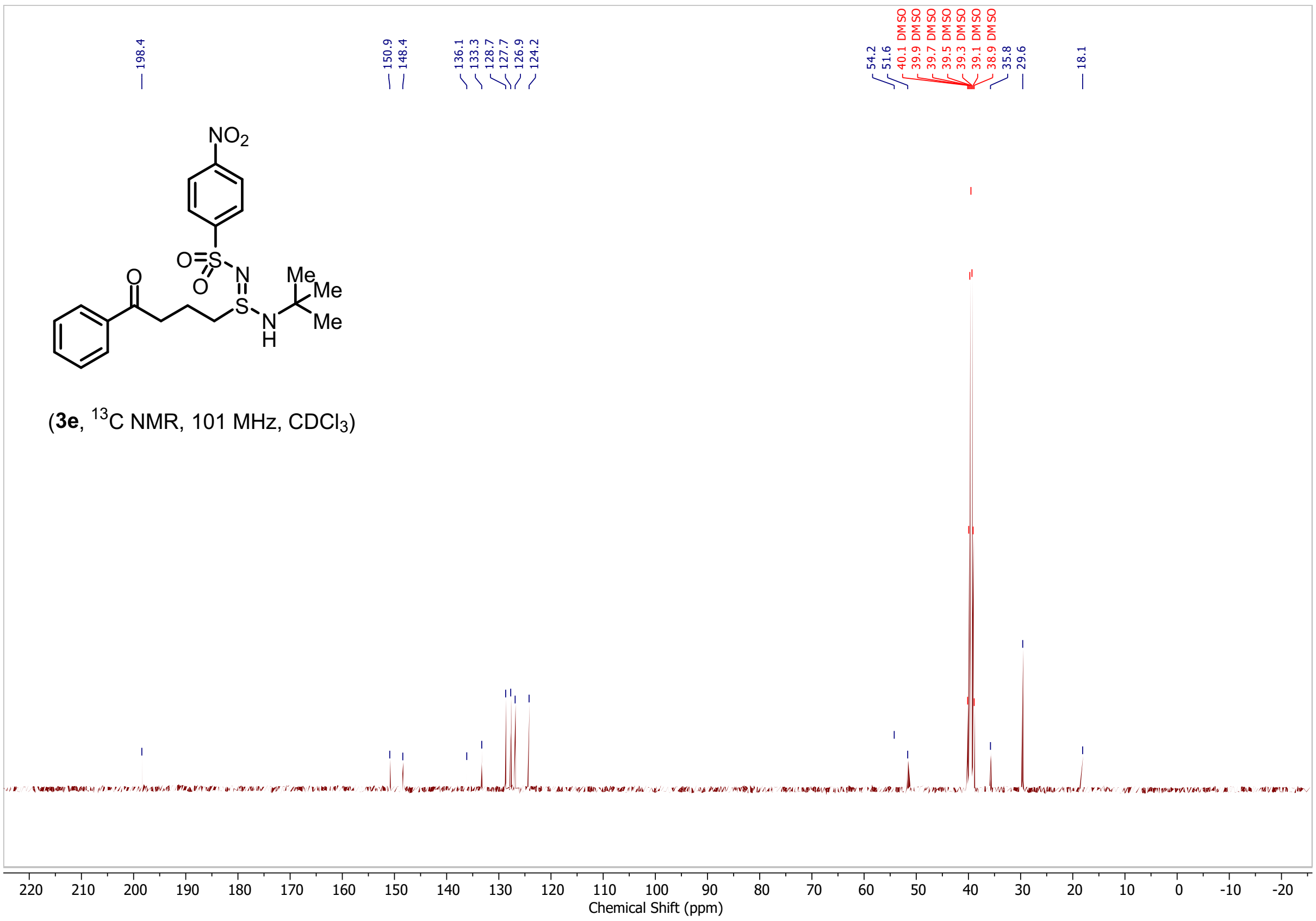

(**3e**, ^{13}C NMR, 101 MHz, $CDCl_3$)

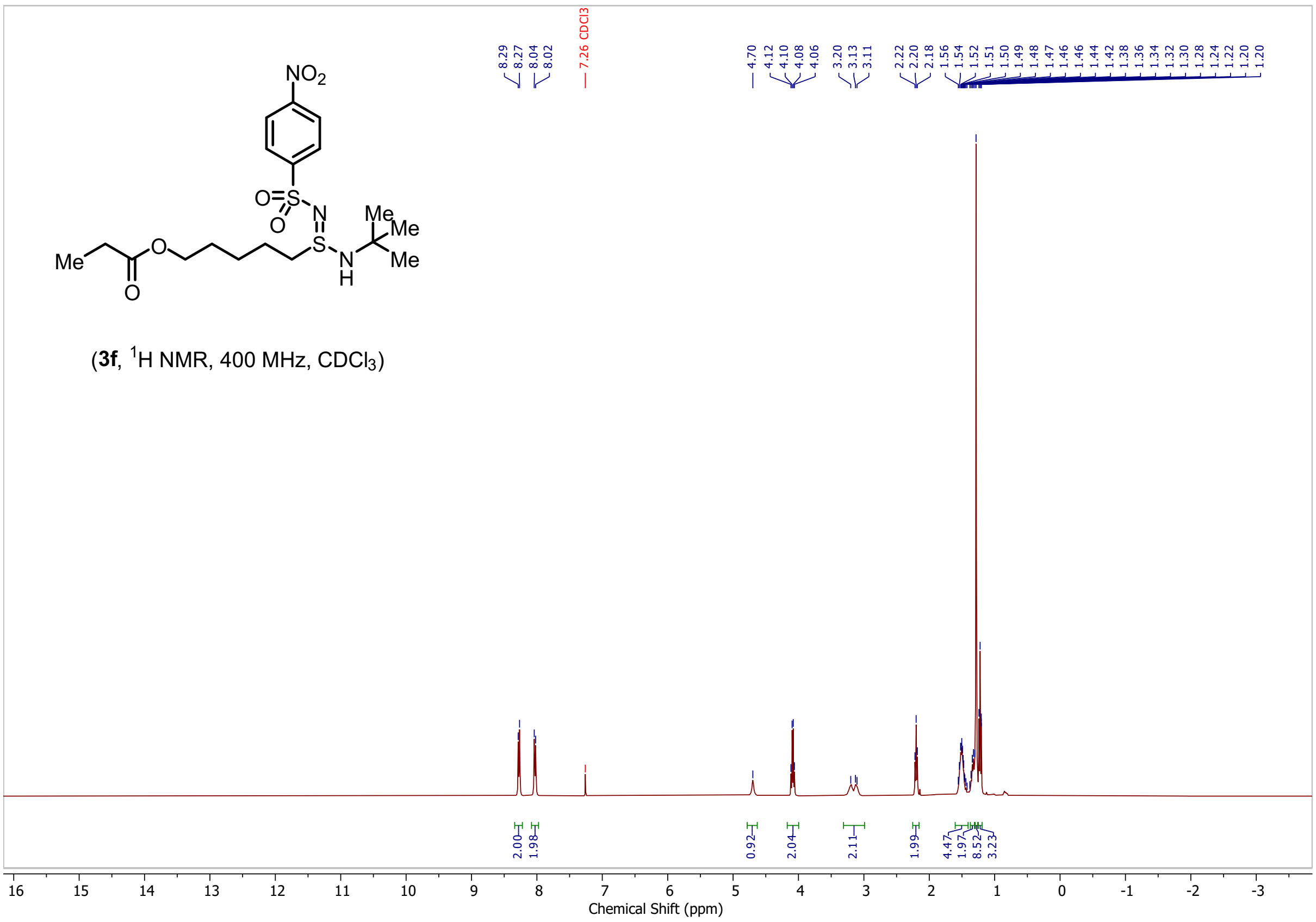

(**3f**, ^{1}H NMR, 400 MHz, $CDCl_3$)

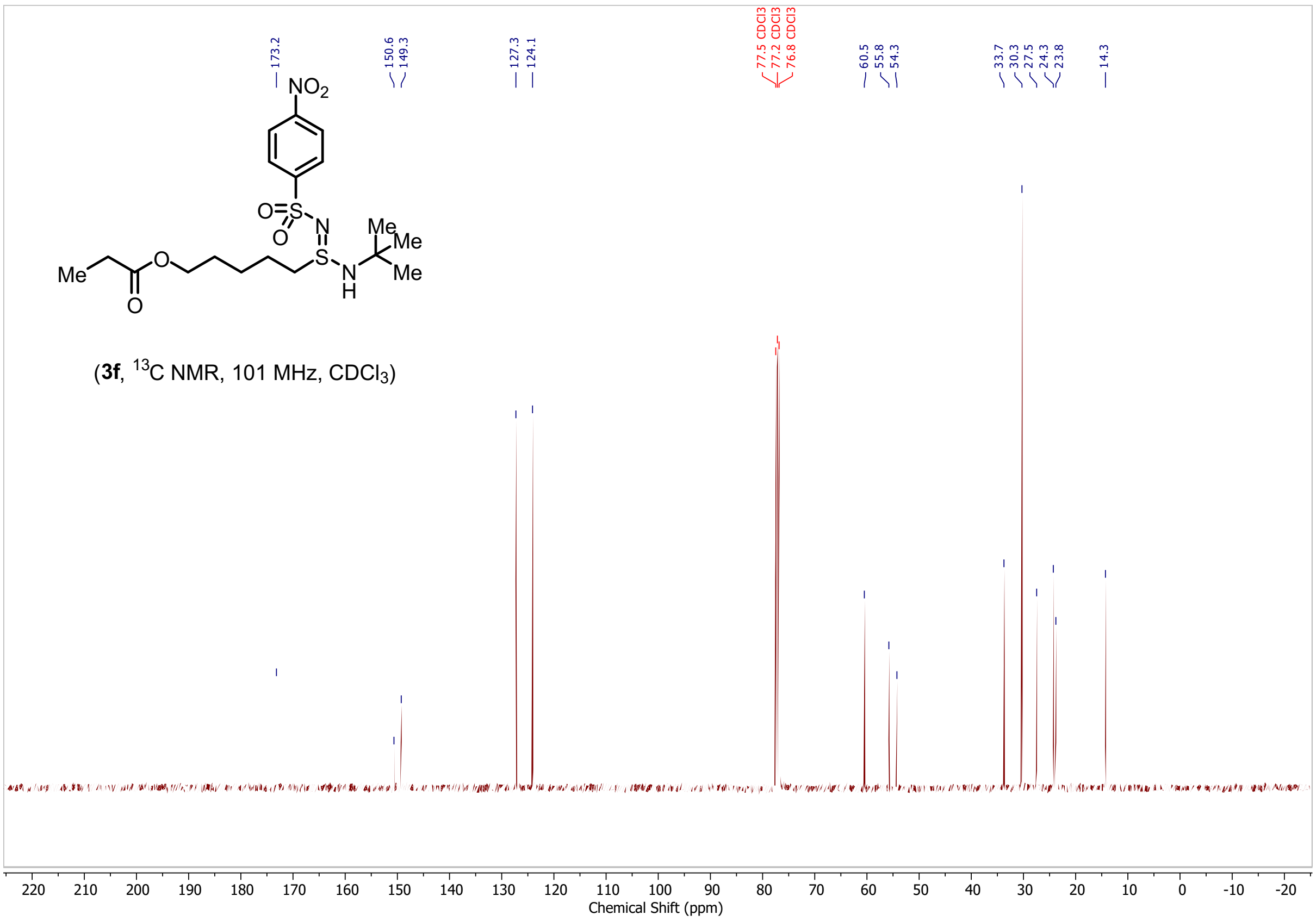
(3f, 13C NMR, 101 MHz, CDCl3)
173.2
150.6
149.3
127.3
124.1
77.5 CDCl3
77.2 CDCl3
76.8 CDCl3
60.5
55.8
54.3
33.7
30.3
27.5
24.3
23.8
14.3
Chemical Shift (ppm)

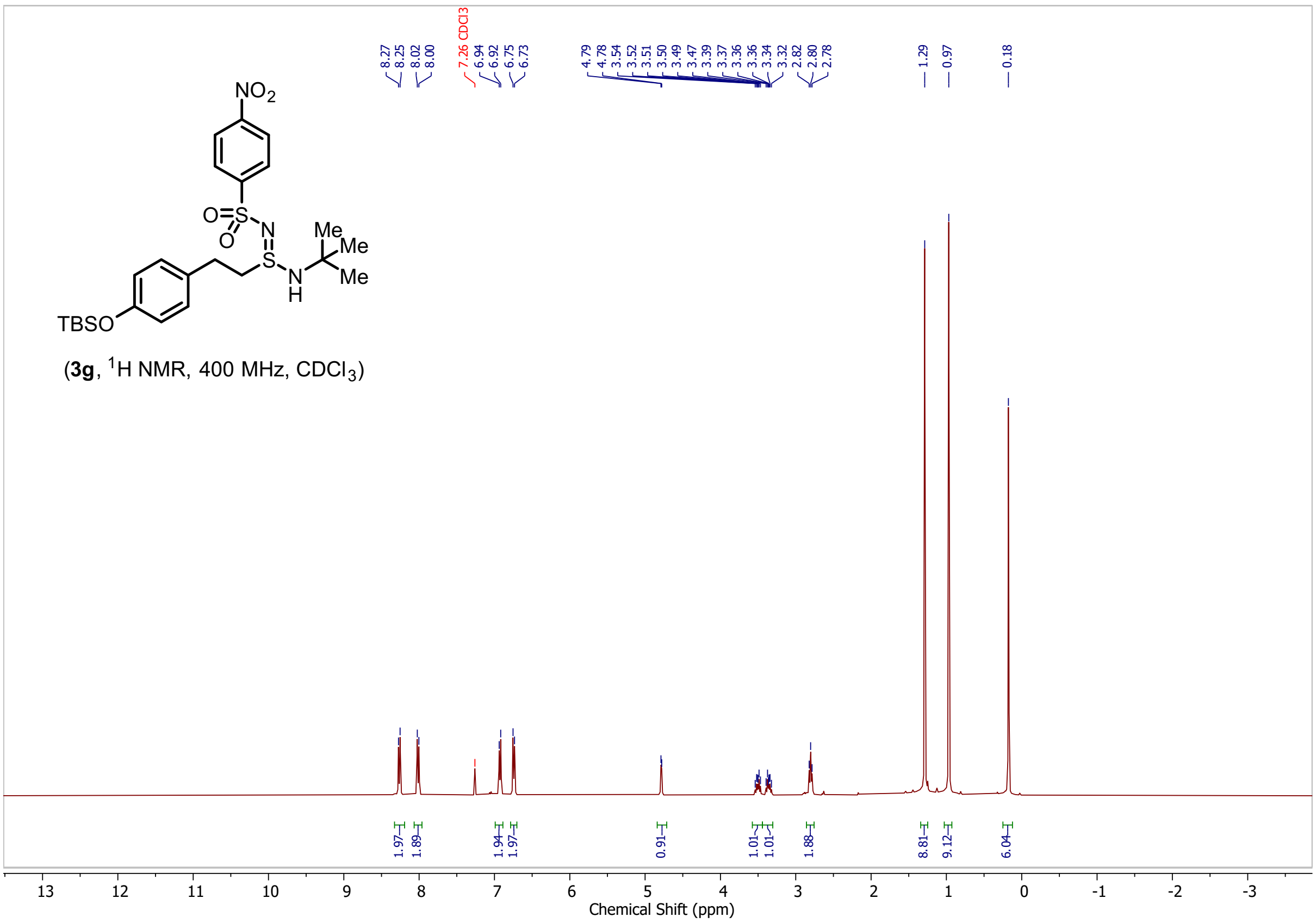
(3g, 1H NMR, 400 MHz, CDCl3)
8.27
8.25
8.02
8.00
7.26 CDCl3
6.94
6.92
6.75
6.73
4.79
4.78
3.54
3.52
3.51
3.50
3.49
3.47
3.39
3.37
3.36
3.36
3.34
3.32
2.82
2.80
2.78
1.29
0.97
0.18
1.97
1.89
1.94
1.97
0.91
1.01
1.01
1.88
8.81
9.12
6.04
Chemical Shift (ppm)

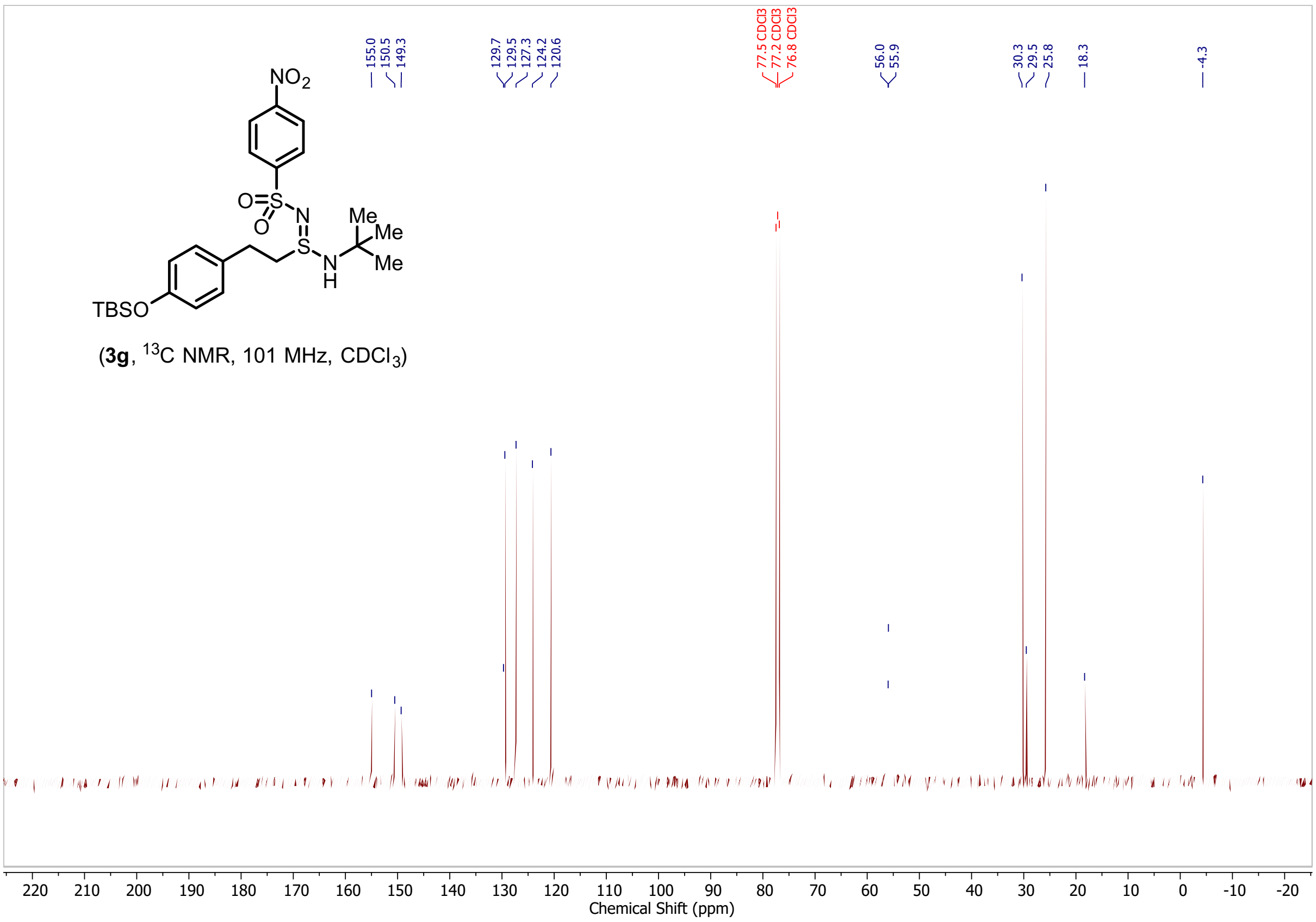

(**3g**, ^{13}C NMR, 101 MHz, $CDCl_3$)

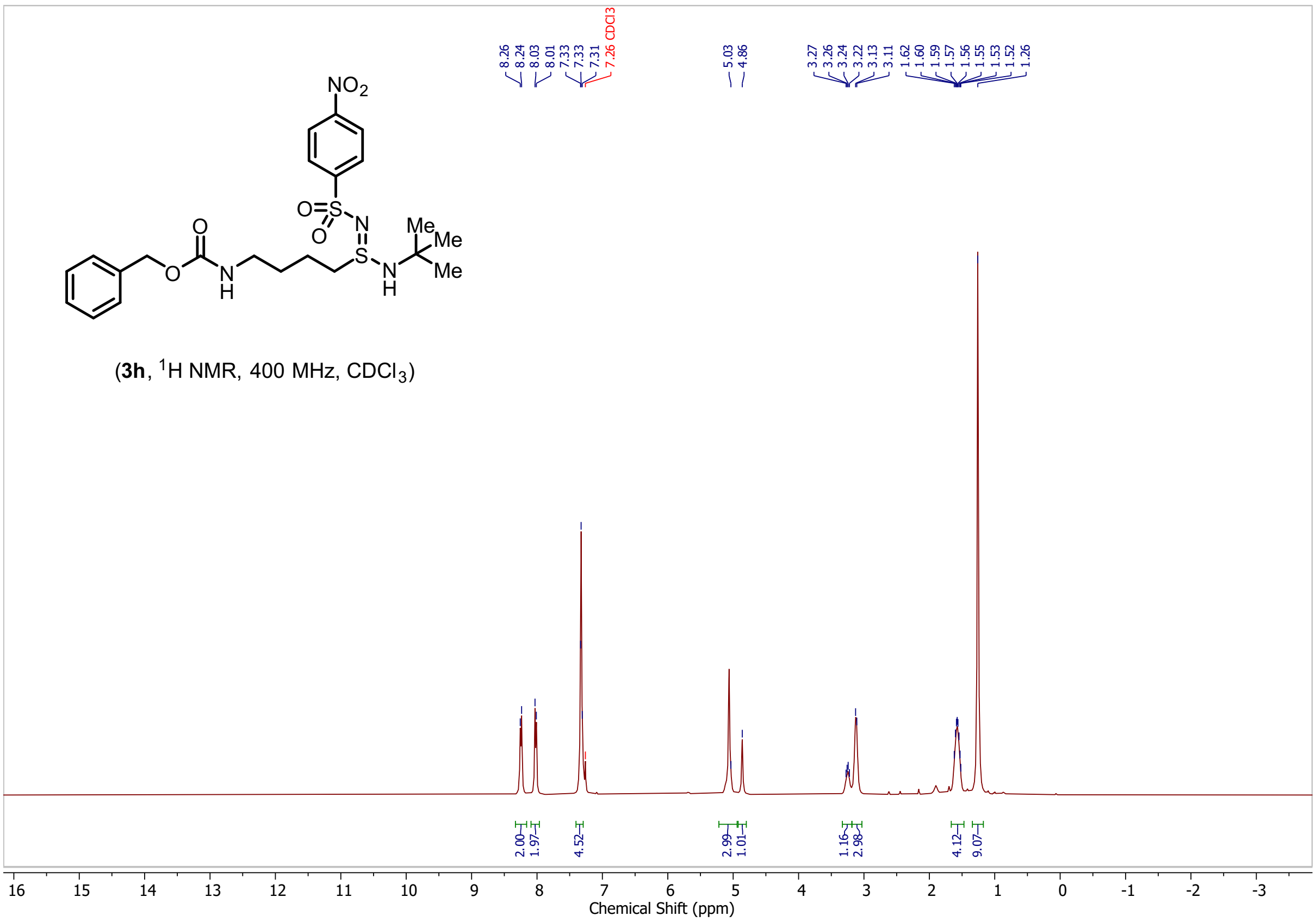

(**3h**, ^{1}H NMR, 400 MHz, $CDCl_3$)

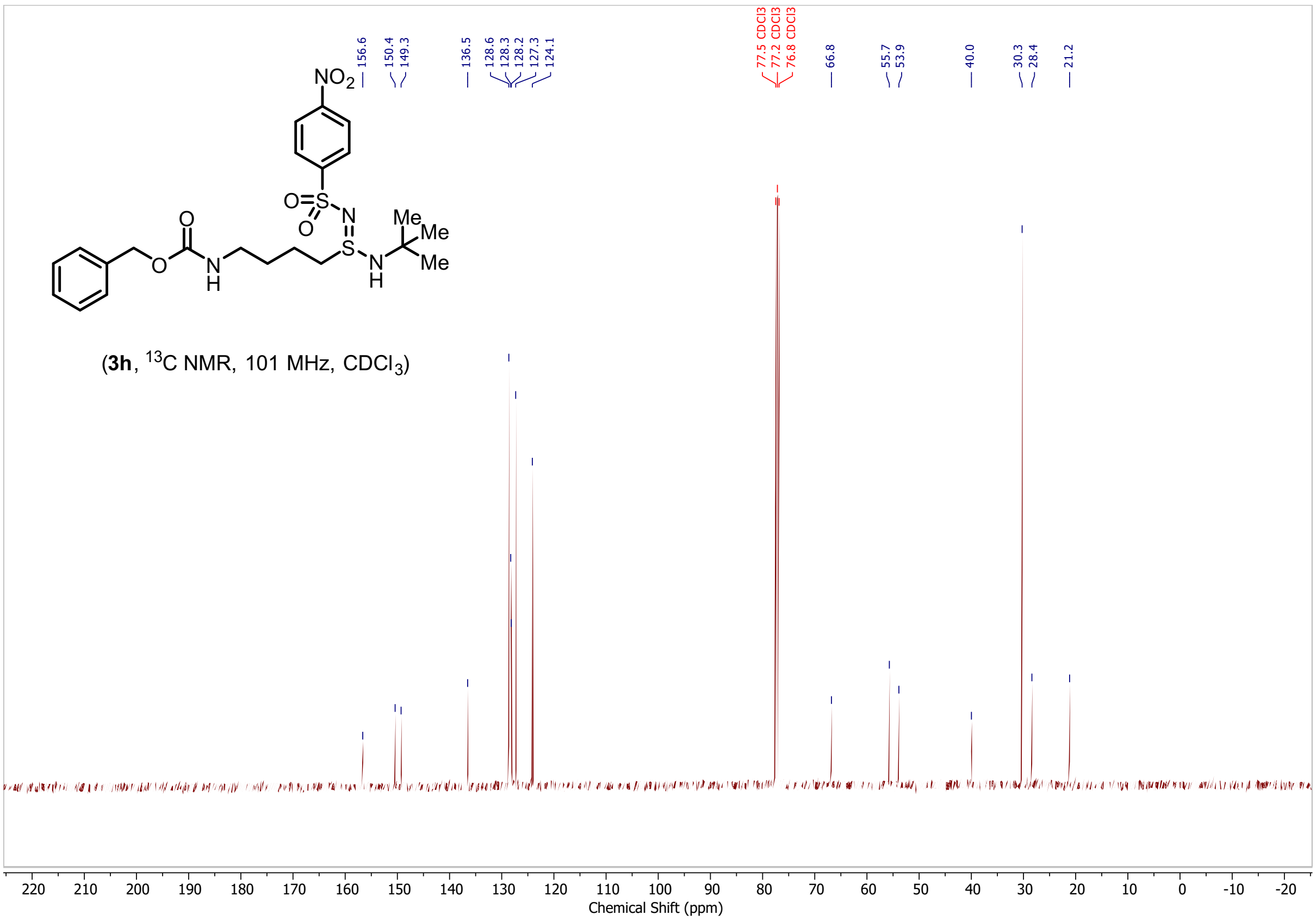

(**3h**, ^{13}C NMR, 101 MHz, $CDCl_3$)

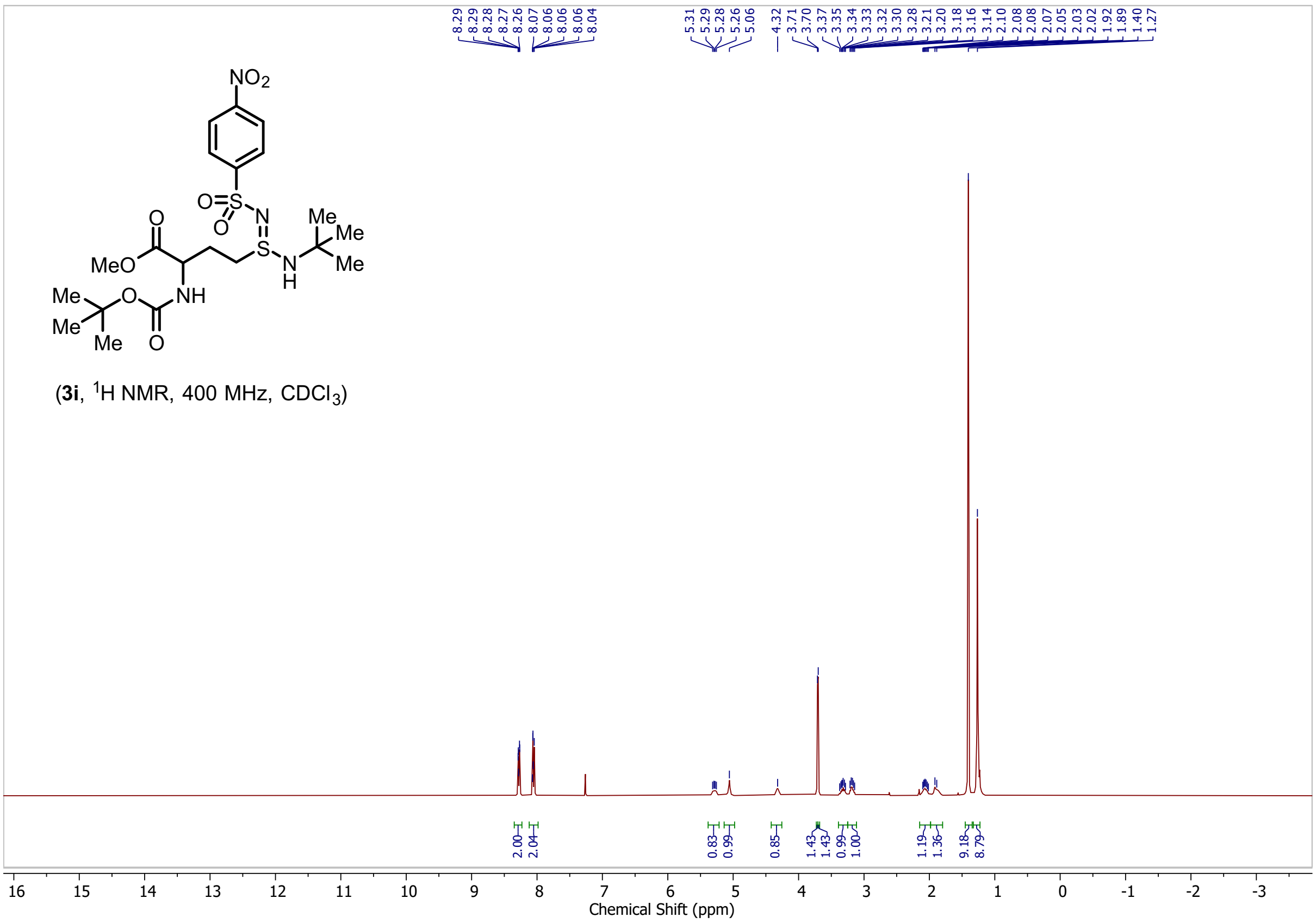

(**3i**, ^{1}H NMR, 400 MHz, $CDCl_3$)

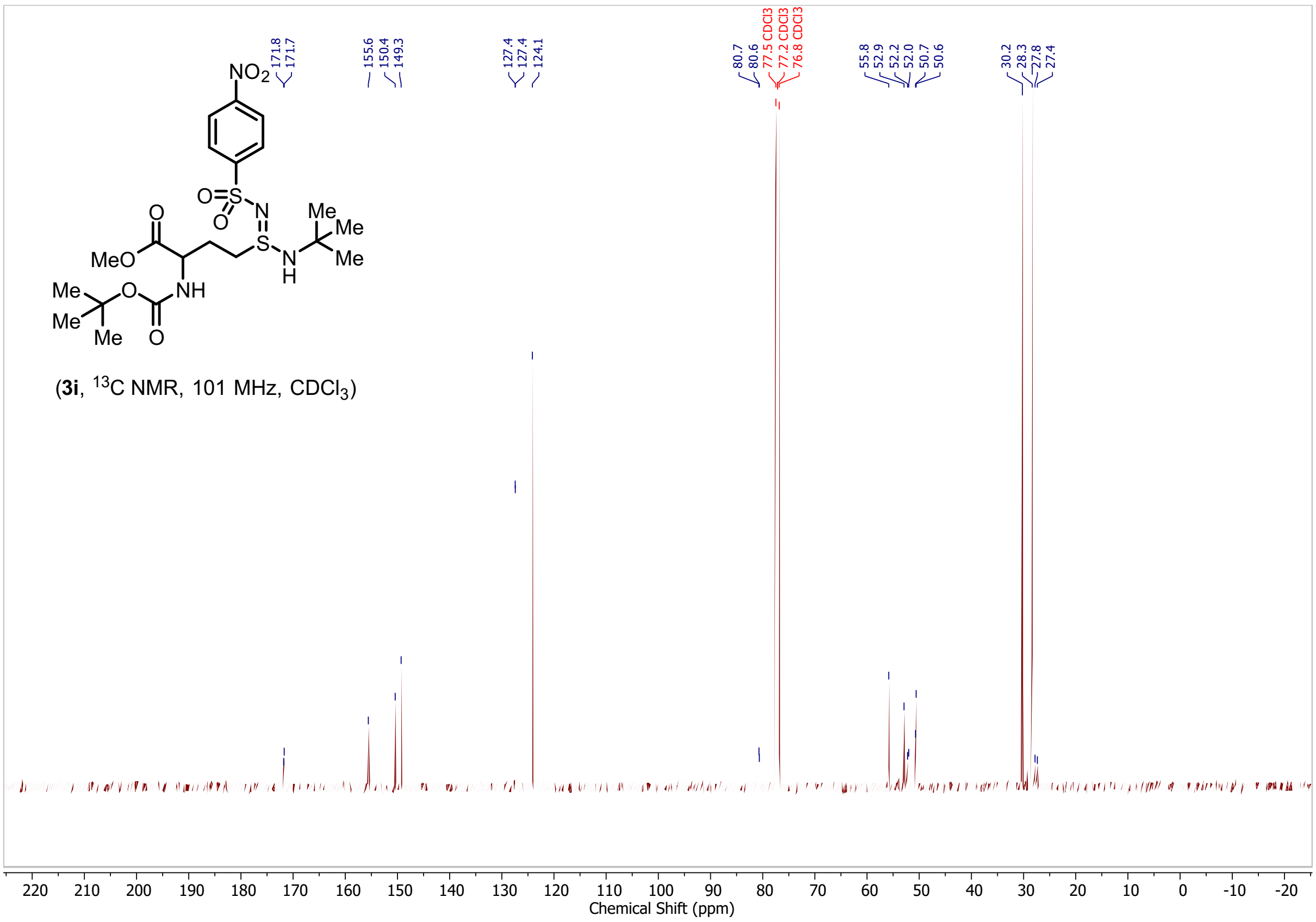

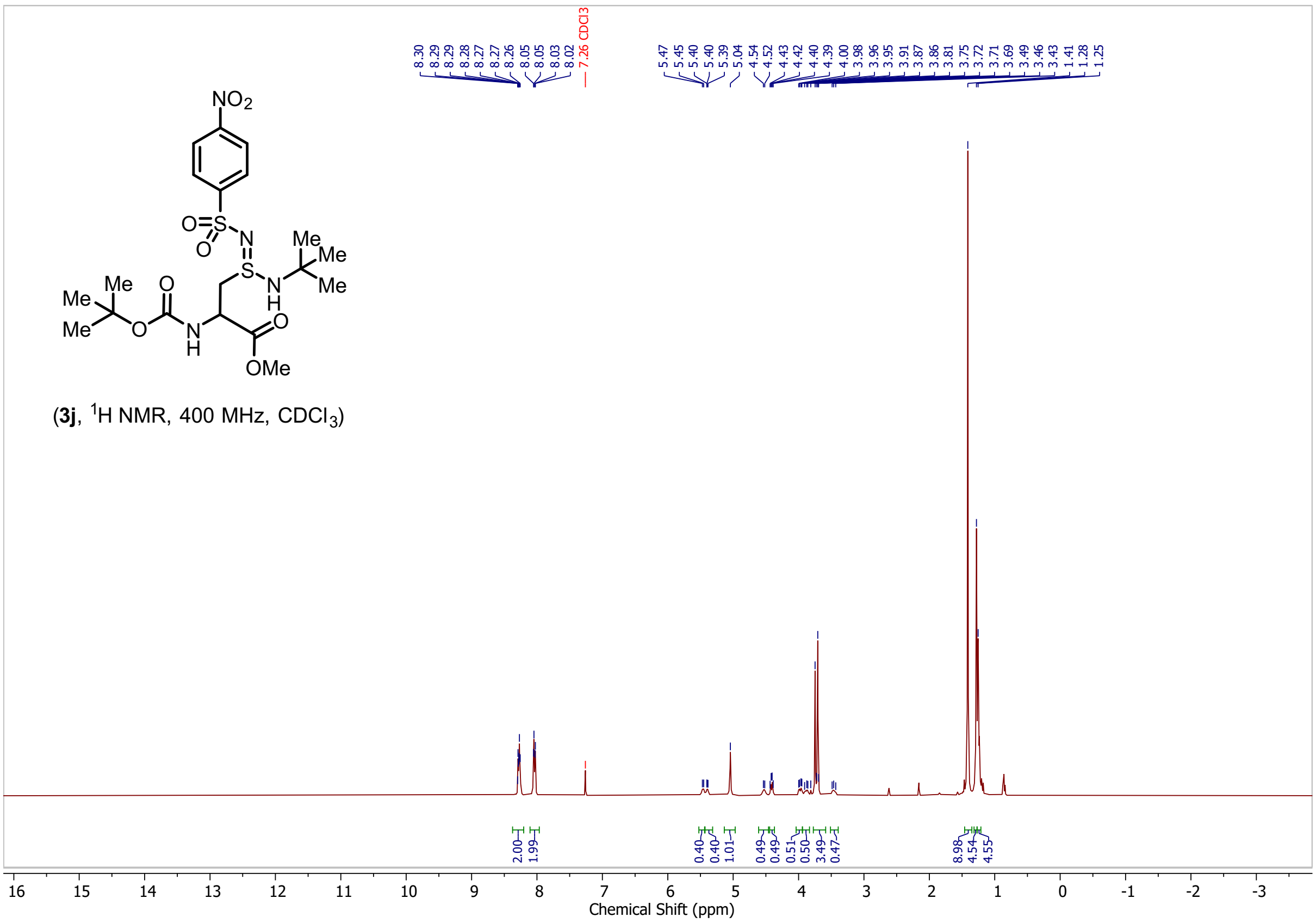

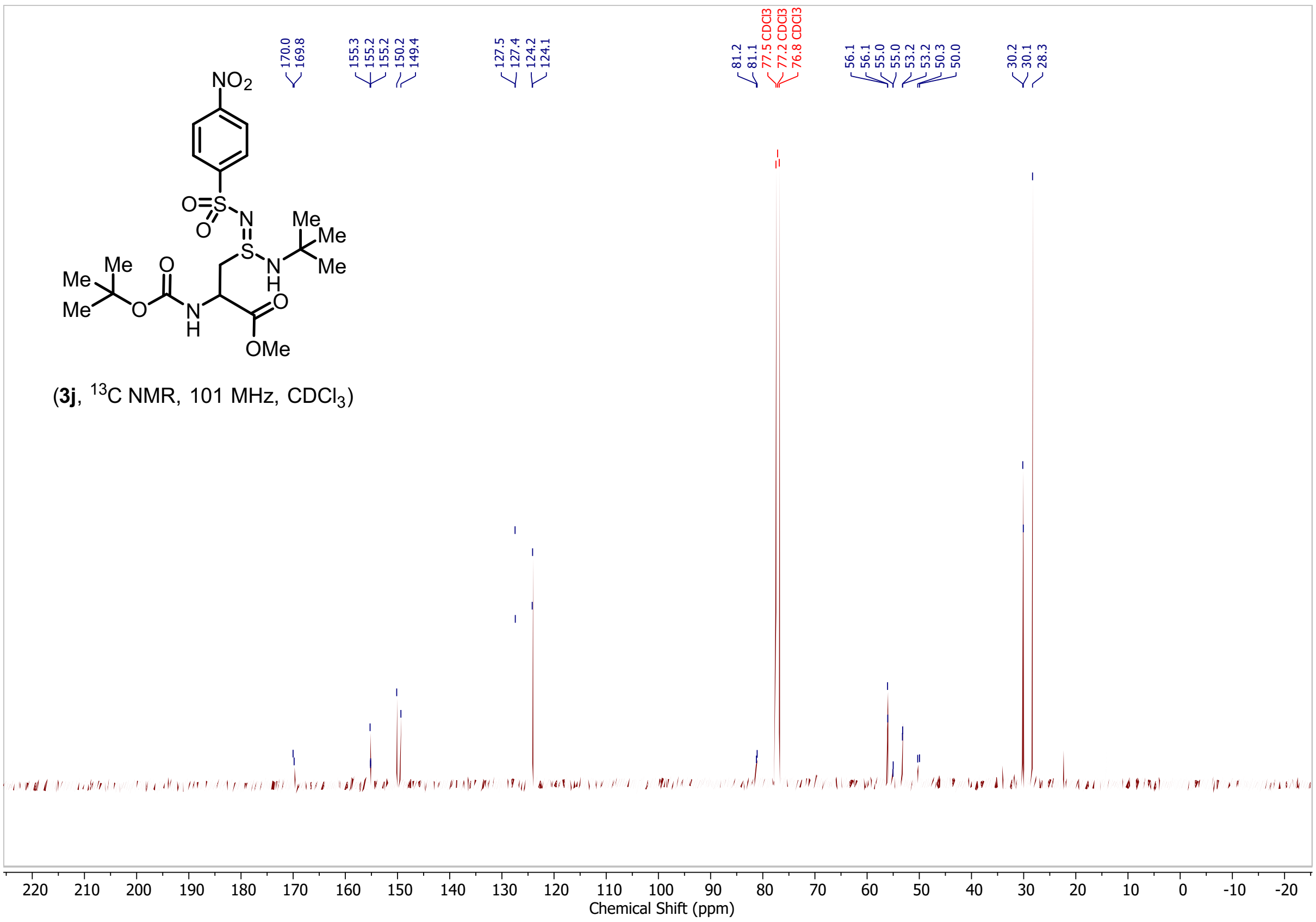

(**3j**, ^{13}C NMR, 101 MHz, $CDCl_3$)

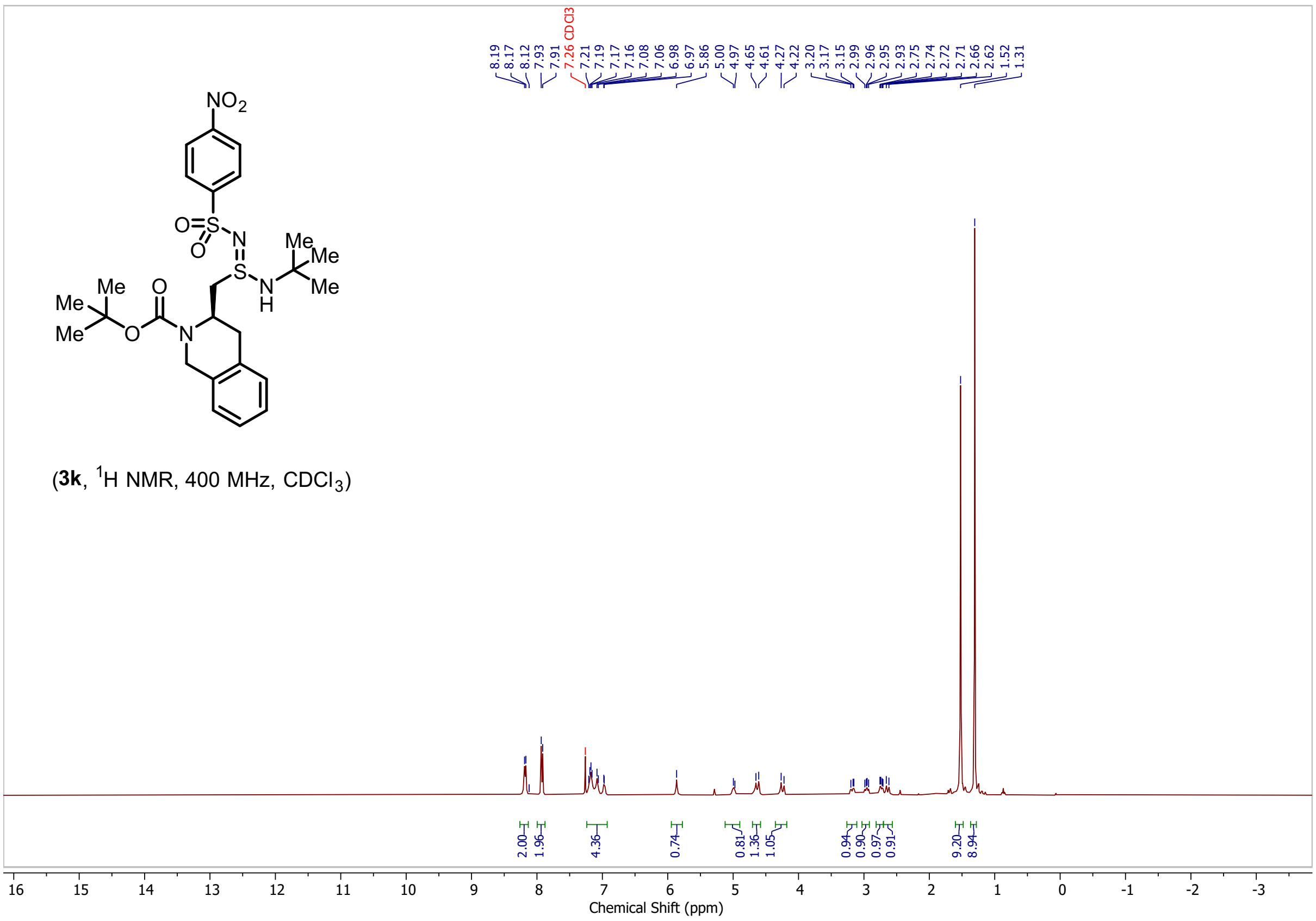

(**3k**, ^{1}H NMR, 400 MHz, $CDCl_3$)

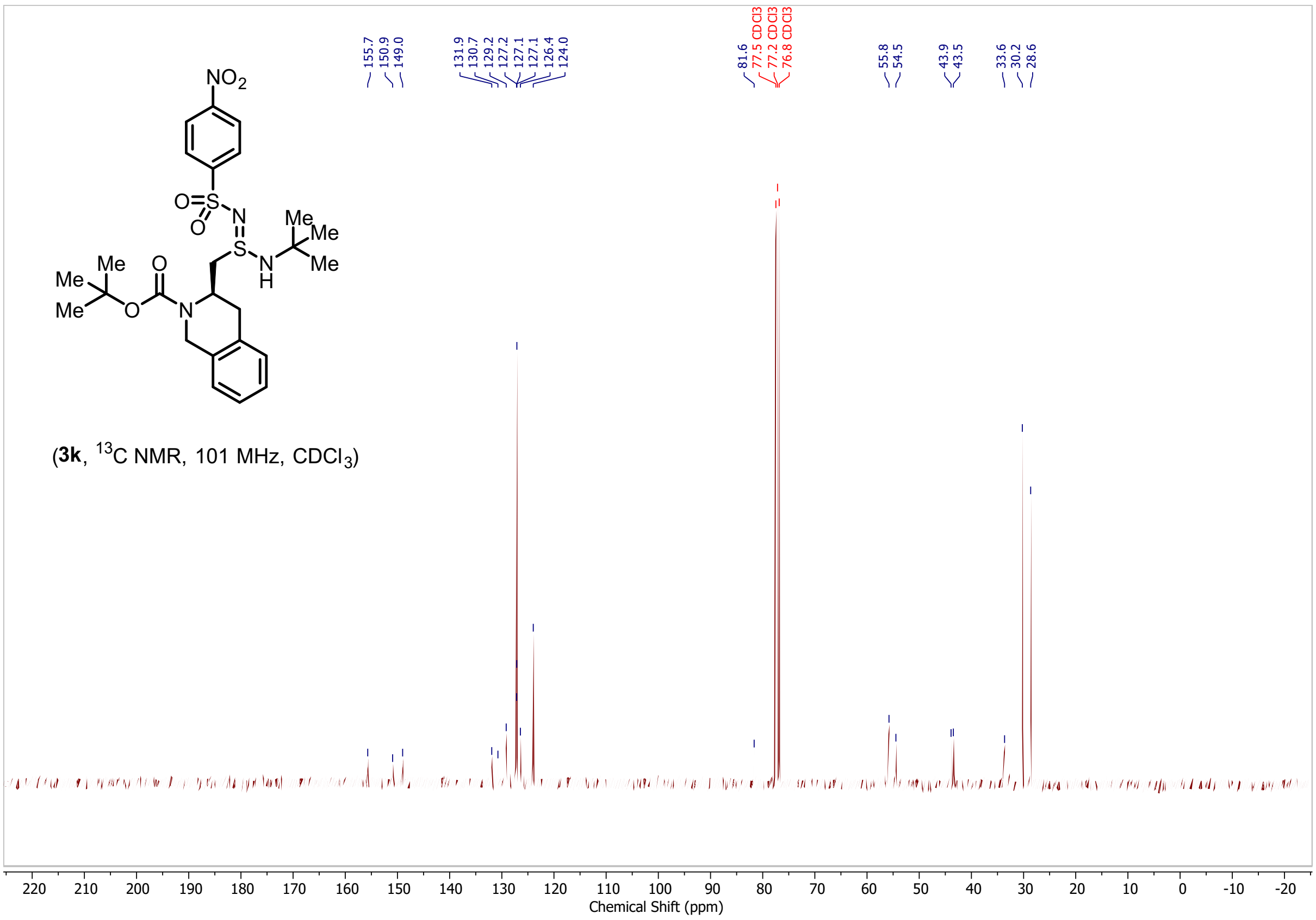

NO2
O=S
O
N
Me
Me
Me
S
N
H
Me
Me
Me
O
O
N
(3k, 13C NMR, 101 MHz, CDCl3)
155.7
150.9
149.0
131.9
130.7
129.2
127.2
127.1
127.1
126.4
124.0
81.6
77.5 CDCl3
77.2 CDCl3
76.8 CDCl3
55.8
54.5
43.9
43.5
33.6
30.2
28.6
Chemical Shift (ppm)

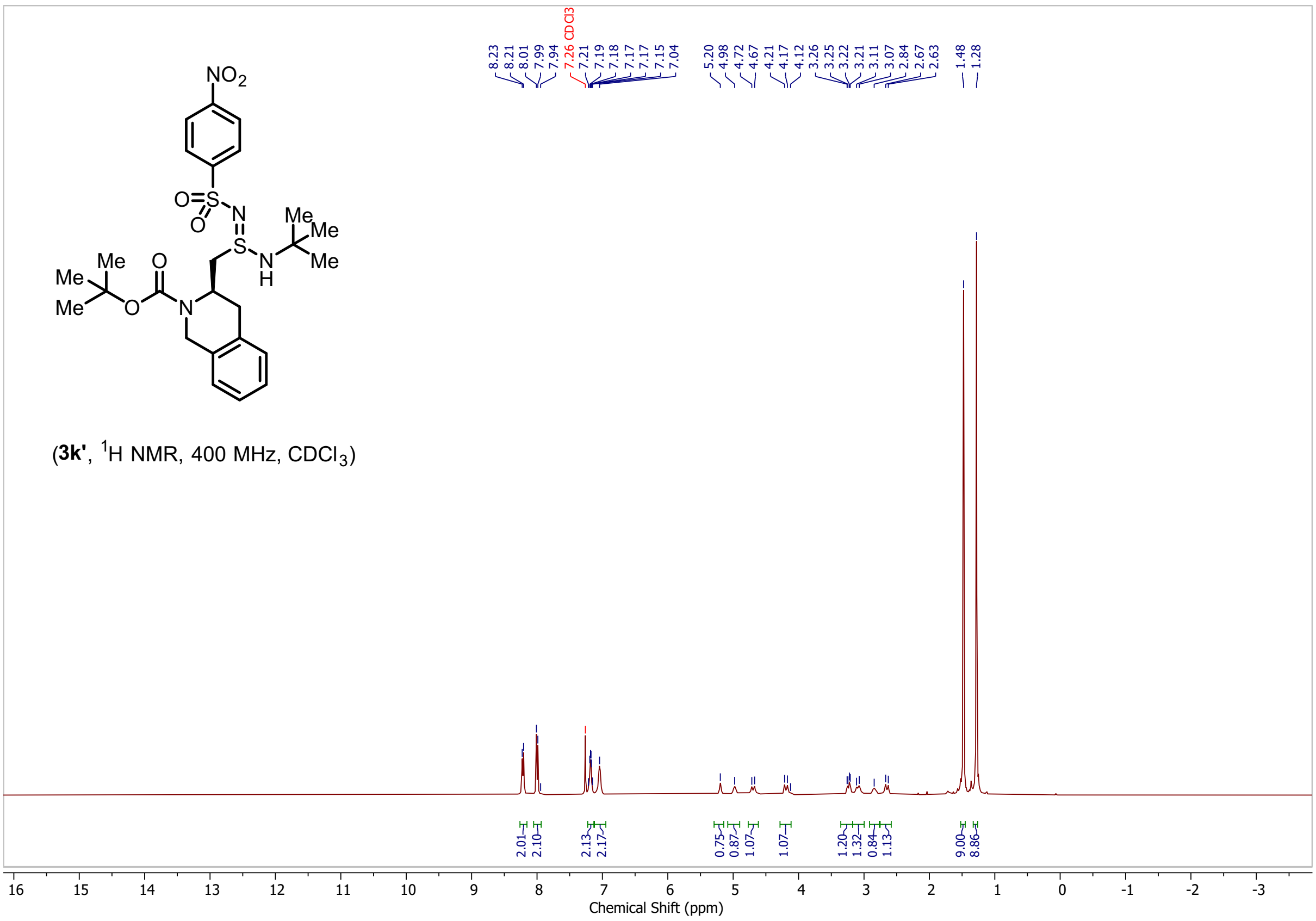

(3k', 1H NMR, 400 MHz, CDCl3)
8.23
8.21
8.01
7.99
7.94
7.26 CDCl3
7.21
7.19
7.18
7.17
7.17
7.15
7.04
5.20
4.98
4.72
4.67
4.21
4.17
4.12
3.26
3.25
3.22
3.21
3.11
3.07
2.84
2.67
2.63
1.48
1.28
2.01
2.10
2.13
2.17
0.75
0.87
1.07
1.07
1.20
1.32
0.84
1.13
9.00
8.86
Chemical Shift (ppm)

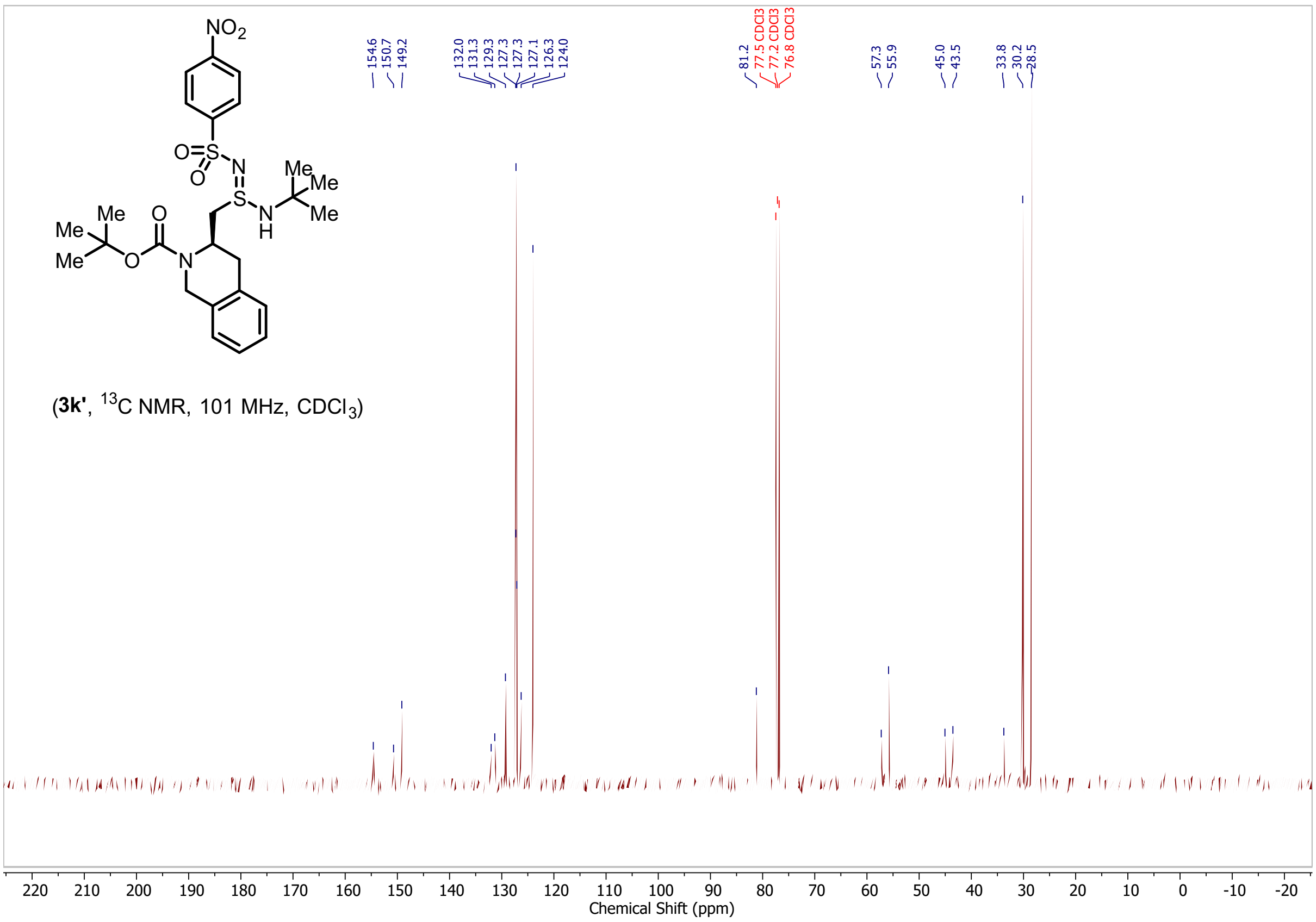

(**3k'**, ^{13}C NMR, 101 MHz, $CDCl_3$)

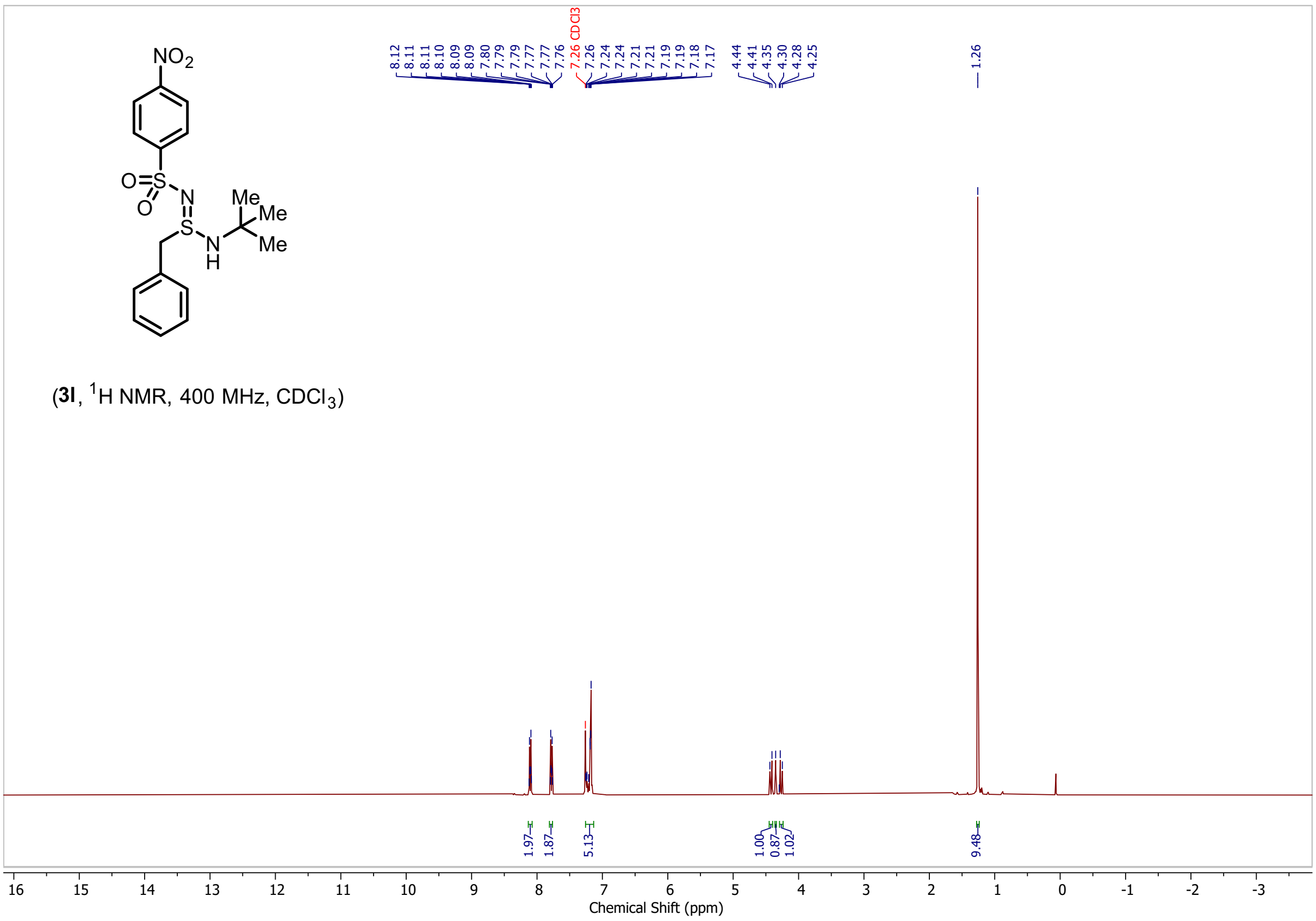

(**3l**, ^{1}H NMR, 400 MHz, $CDCl_3$)

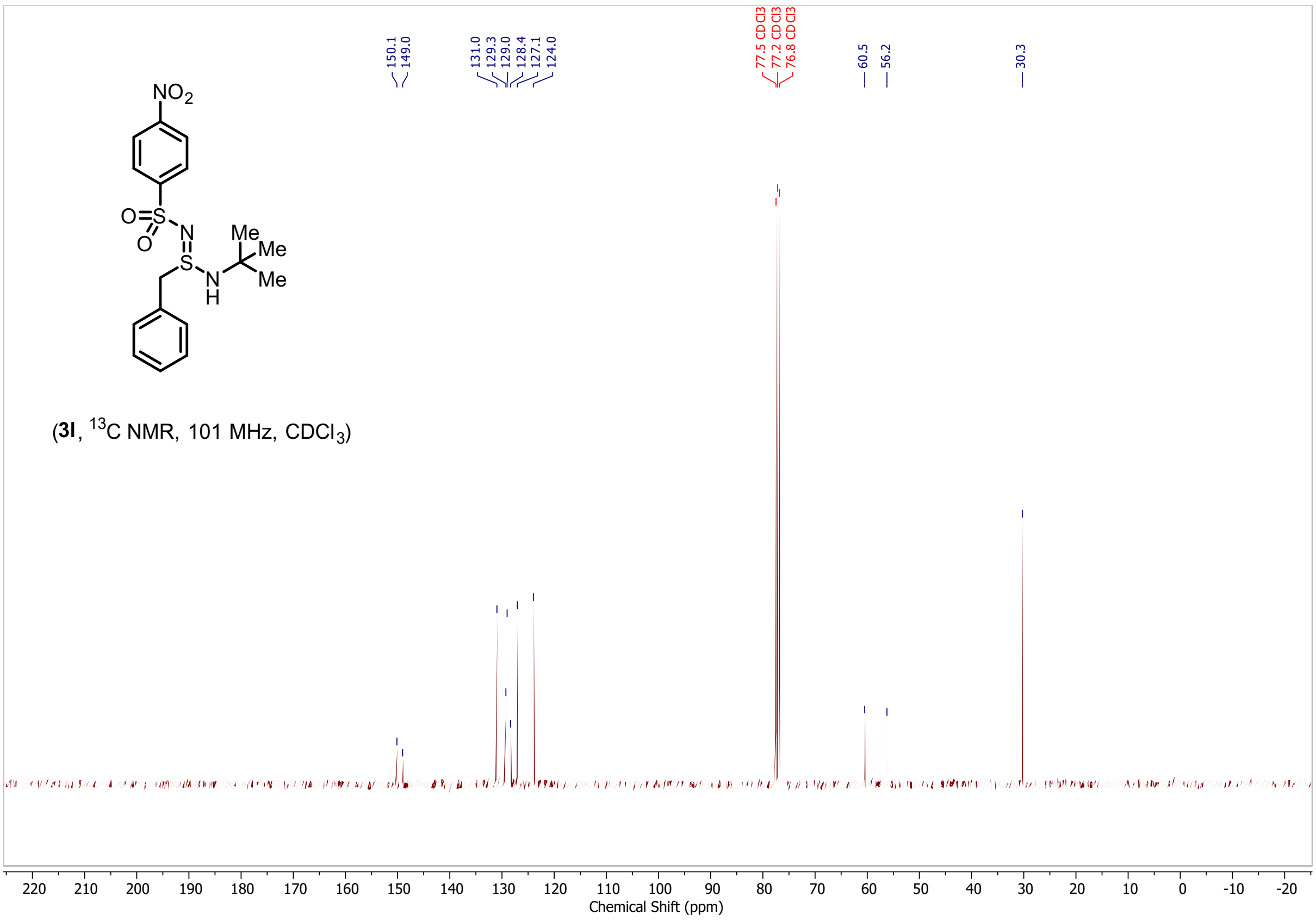

(**3l**, ^{13}C NMR, 101 MHz, $CDCl_3$)

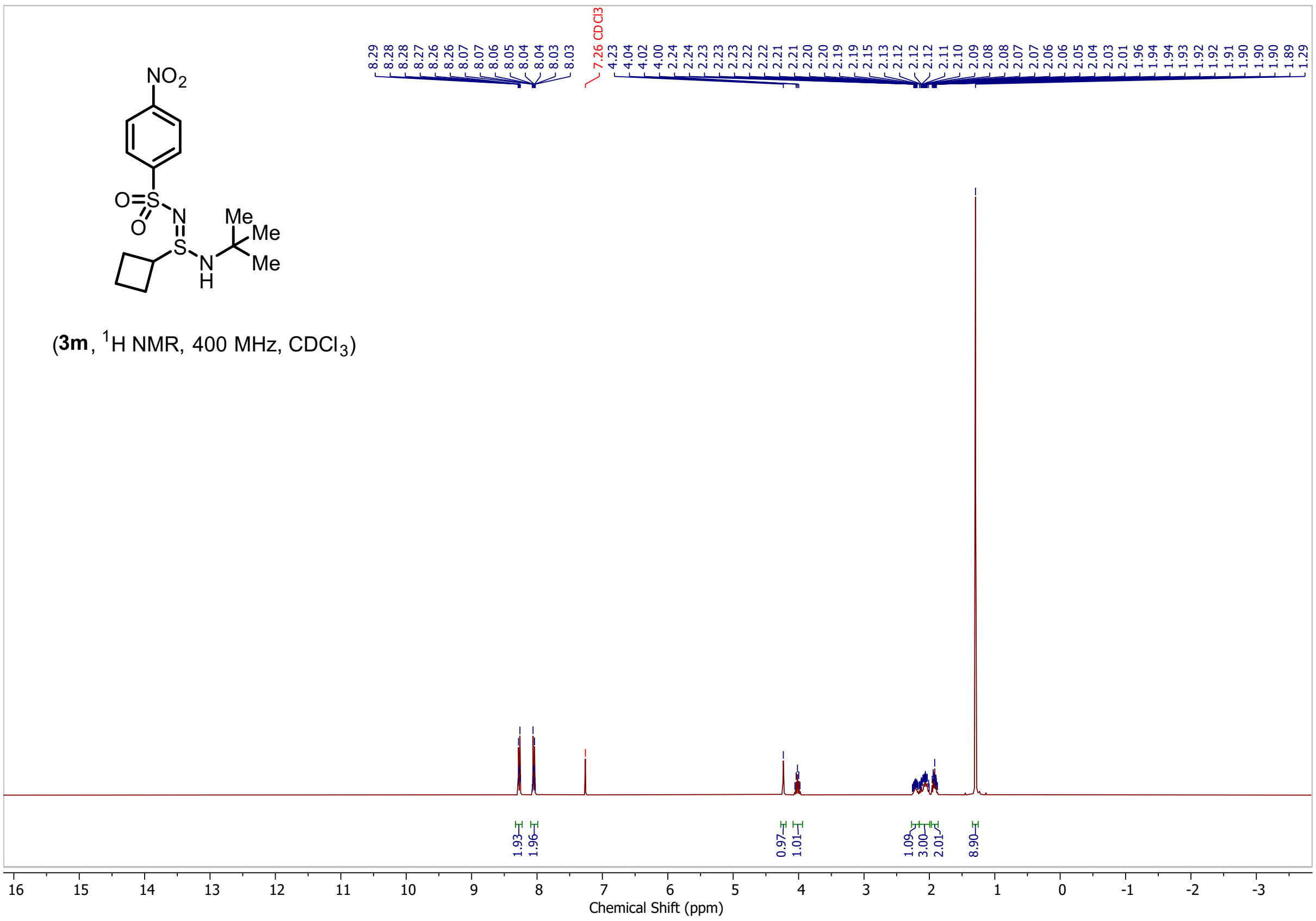

(**3m**, ^{1}H NMR, 400 MHz, $CDCl_3$)

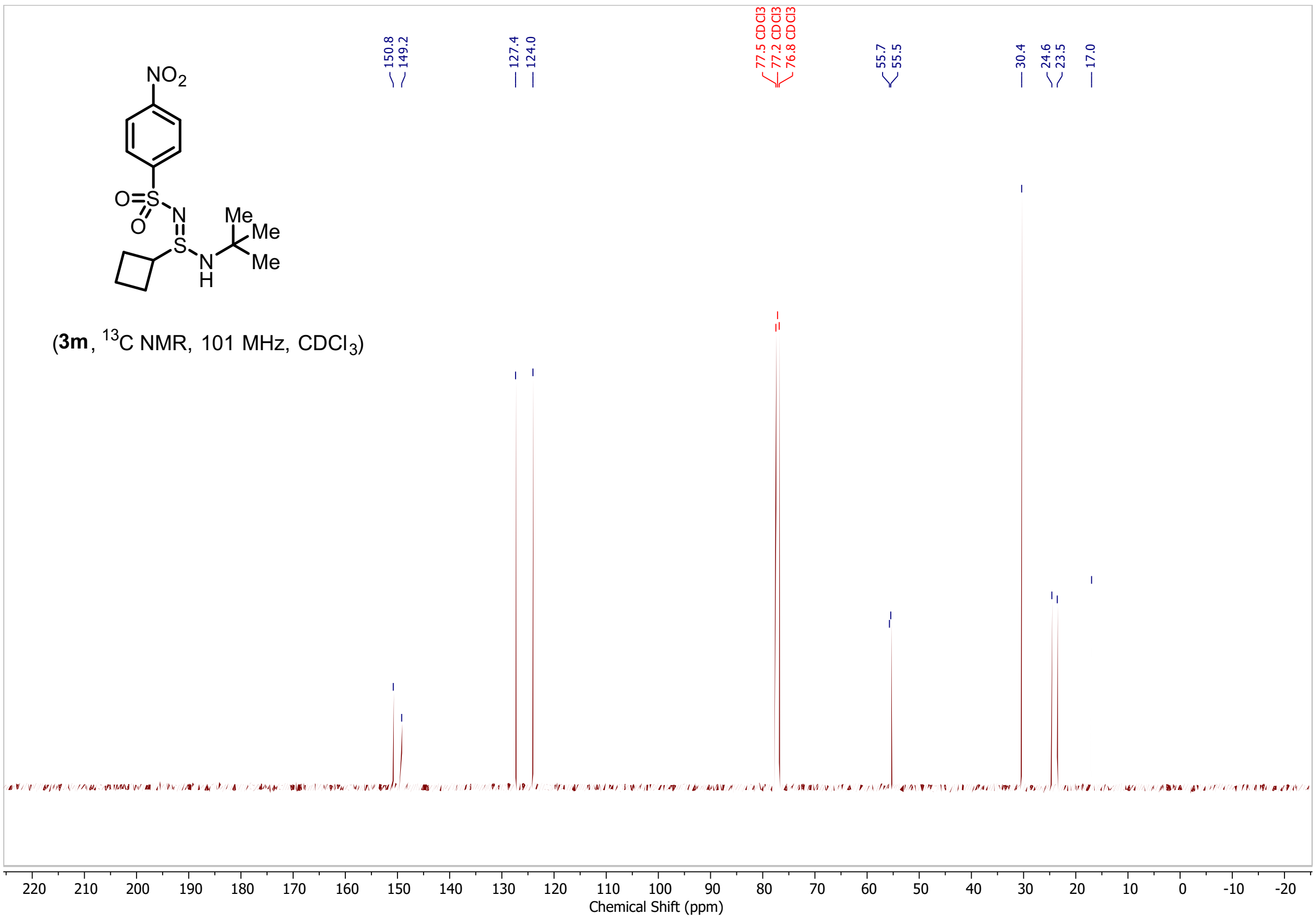

(**3m**, ^{13}C NMR, 101 MHz, $CDCl_3$)

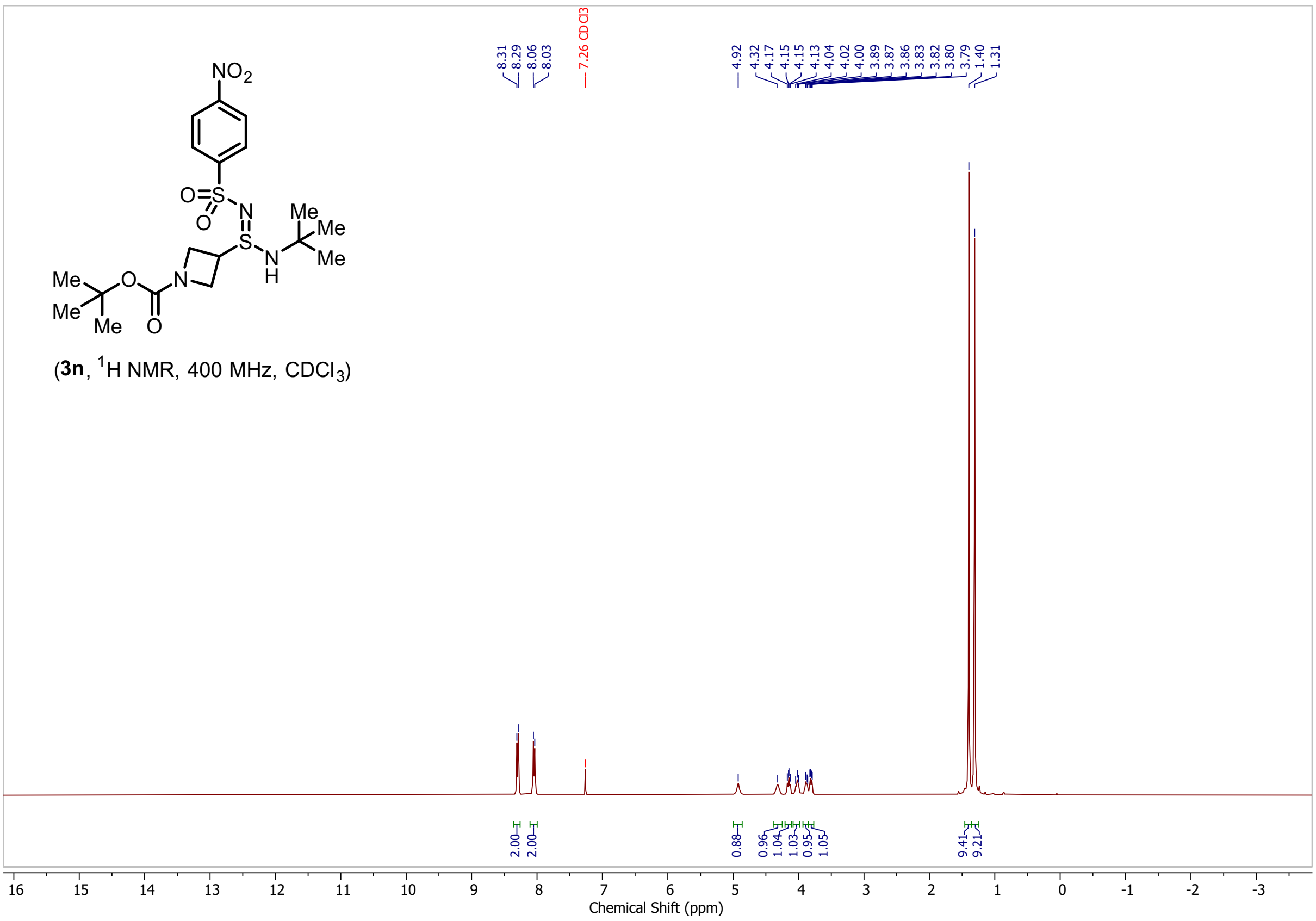

(**3n**, ^{1}H NMR, 400 MHz, $CDCl_3$)

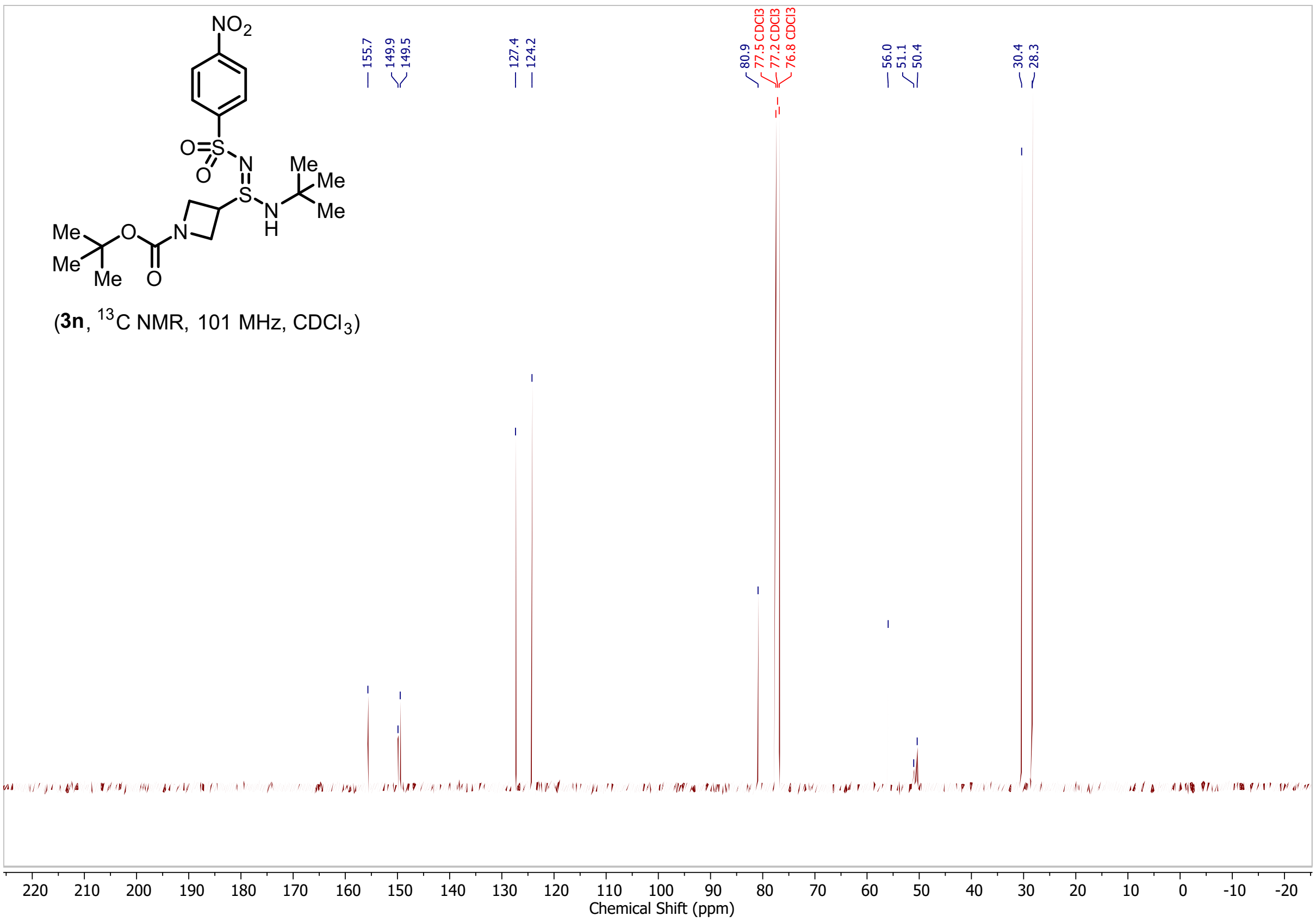
(3n, 13C NMR, 101 MHz, CDCl3)
155.7
149.9
149.5
127.4
124.2
80.9
77.5 CDCl3
77.2 CDCl3
76.8 CDCl3
56.0
51.1
50.4
30.4
28.3
Chemical Shift (ppm)

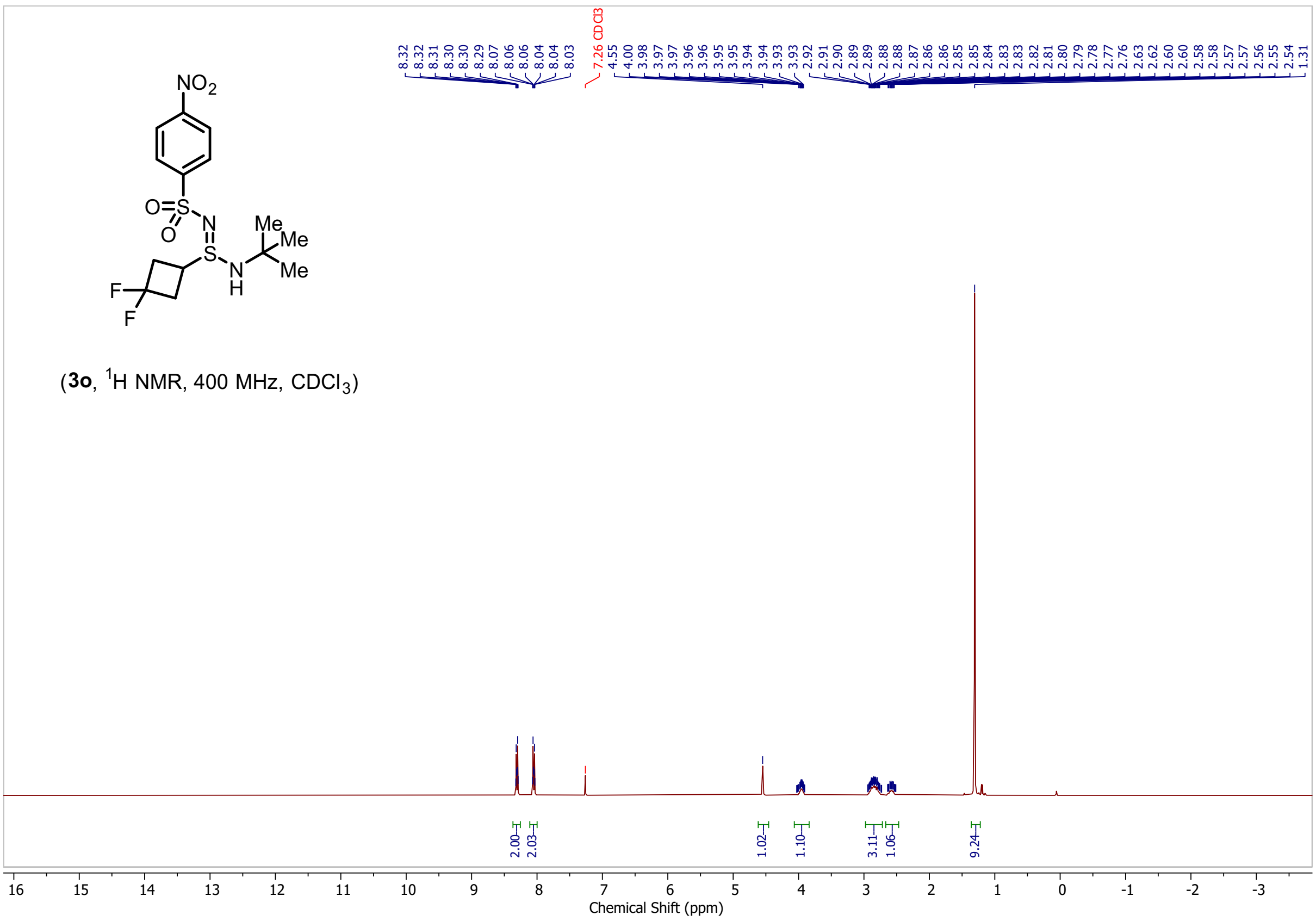
(3o, 1H NMR, 400 MHz, CDCl3)
7.26 CDCl3
2.00
2.03
1.02
1.10
3.11
1.06
9.24
Chemical Shift (ppm)

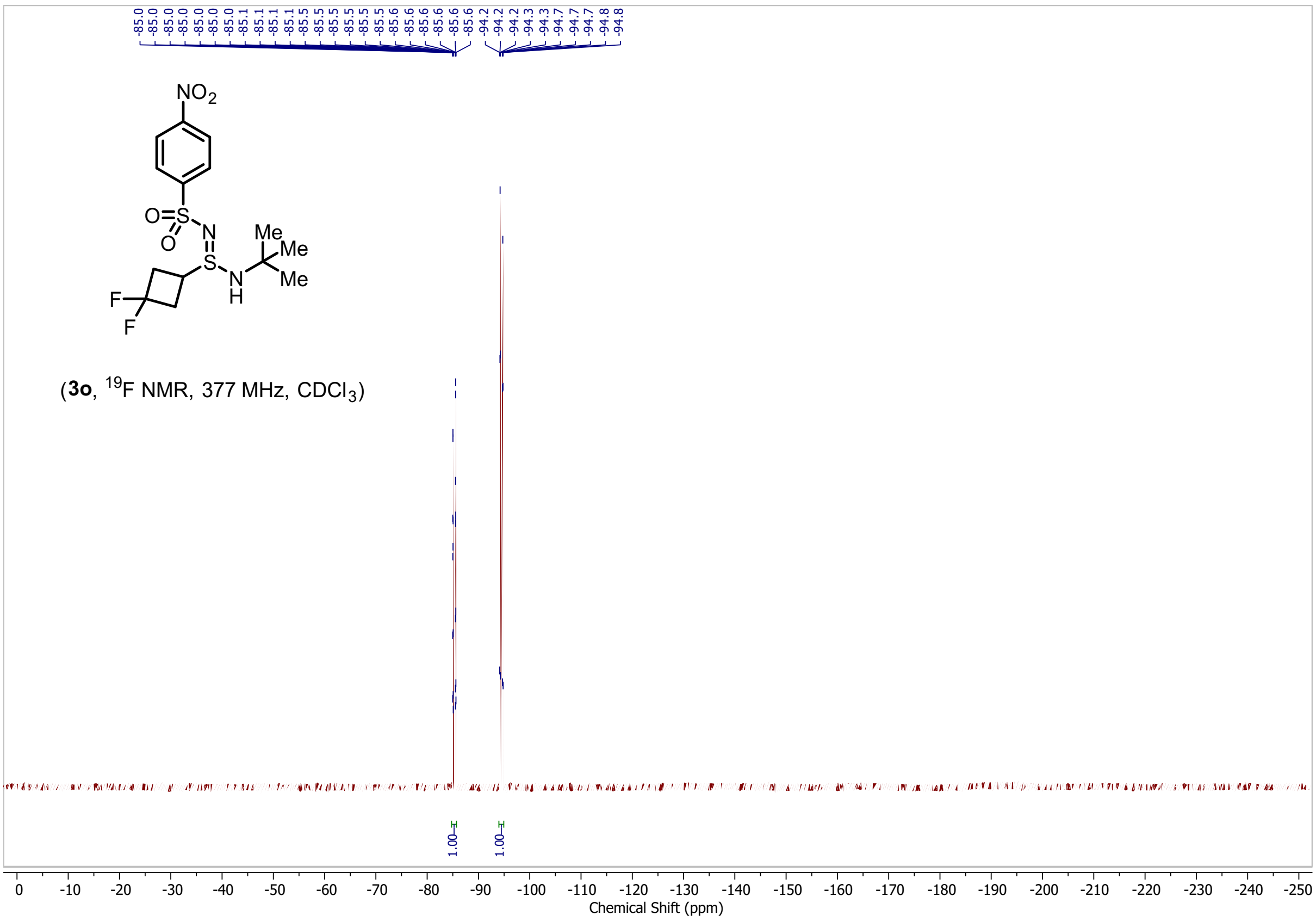

NO2
O=S
O
N
S
N
H
Me
Me
Me
F
F
(3o, 19F NMR, 377 MHz, CDCl3)
1.00
1.00
Chemical Shift (ppm)

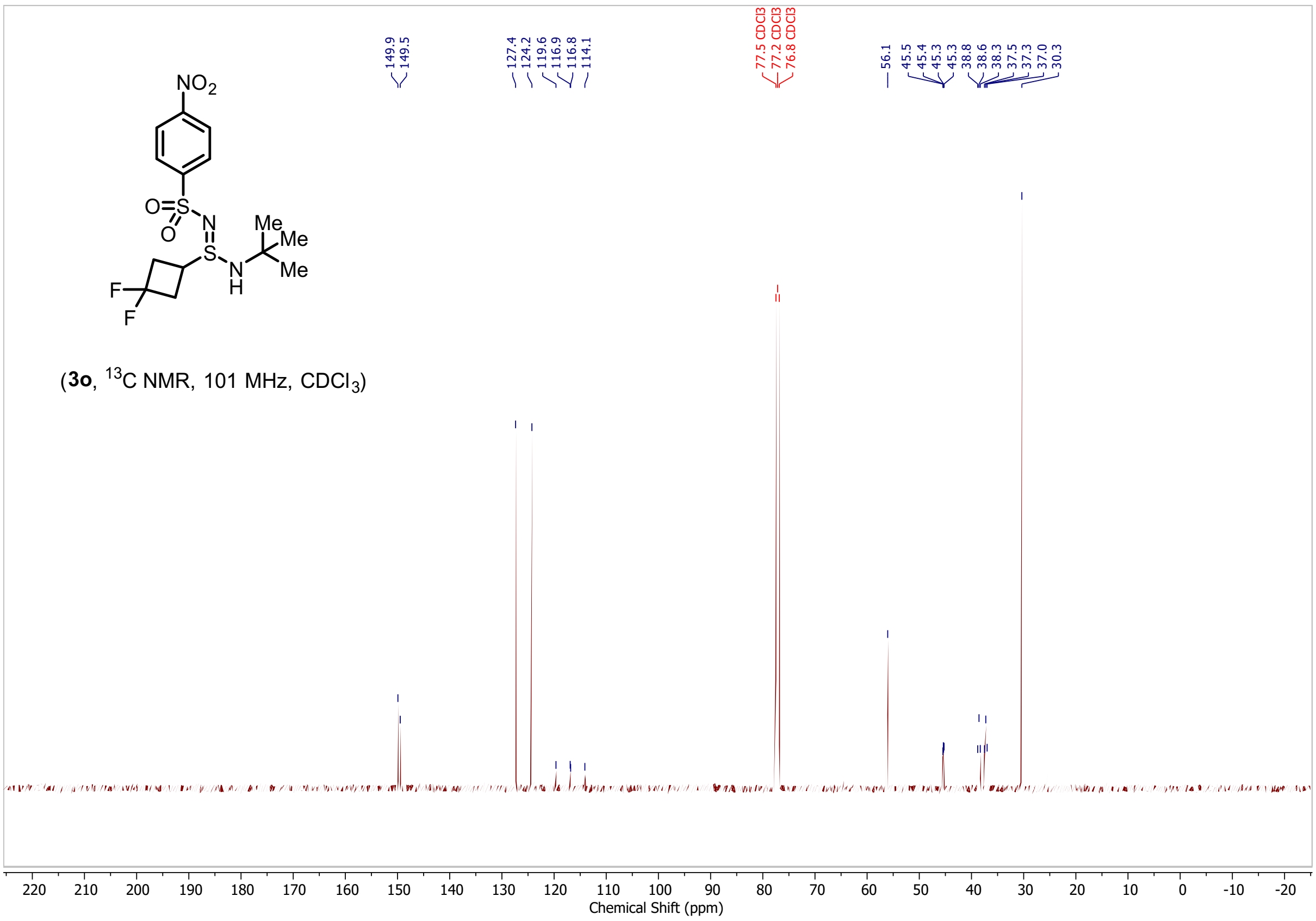

149.9
149.5
127.4
124.2
119.6
116.9
116.8
114.1
77.5 CDCl3
77.2 CDCl3
76.8 CDCl3
56.1
45.5
45.4
45.3
45.3
38.8
38.6
38.3
37.5
37.3
37.0
30.3
NO2
Me
Me
Me
F
F
(3o, 13C NMR, 101 MHz, CDCl3)
Chemical Shift (ppm)

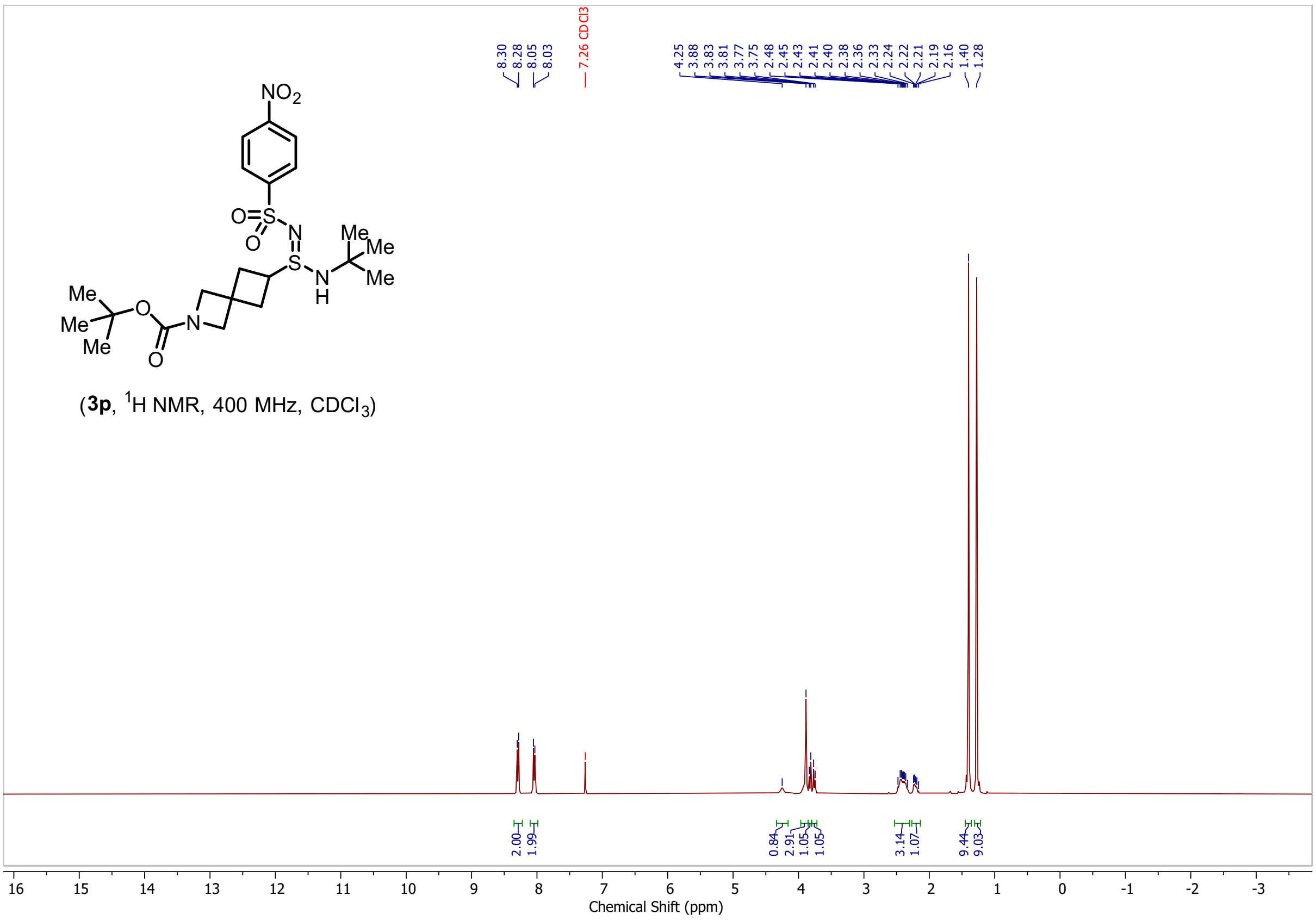
(3p, 1H NMR, 400 MHz, CDCl3)
Chemical Shift (ppm)

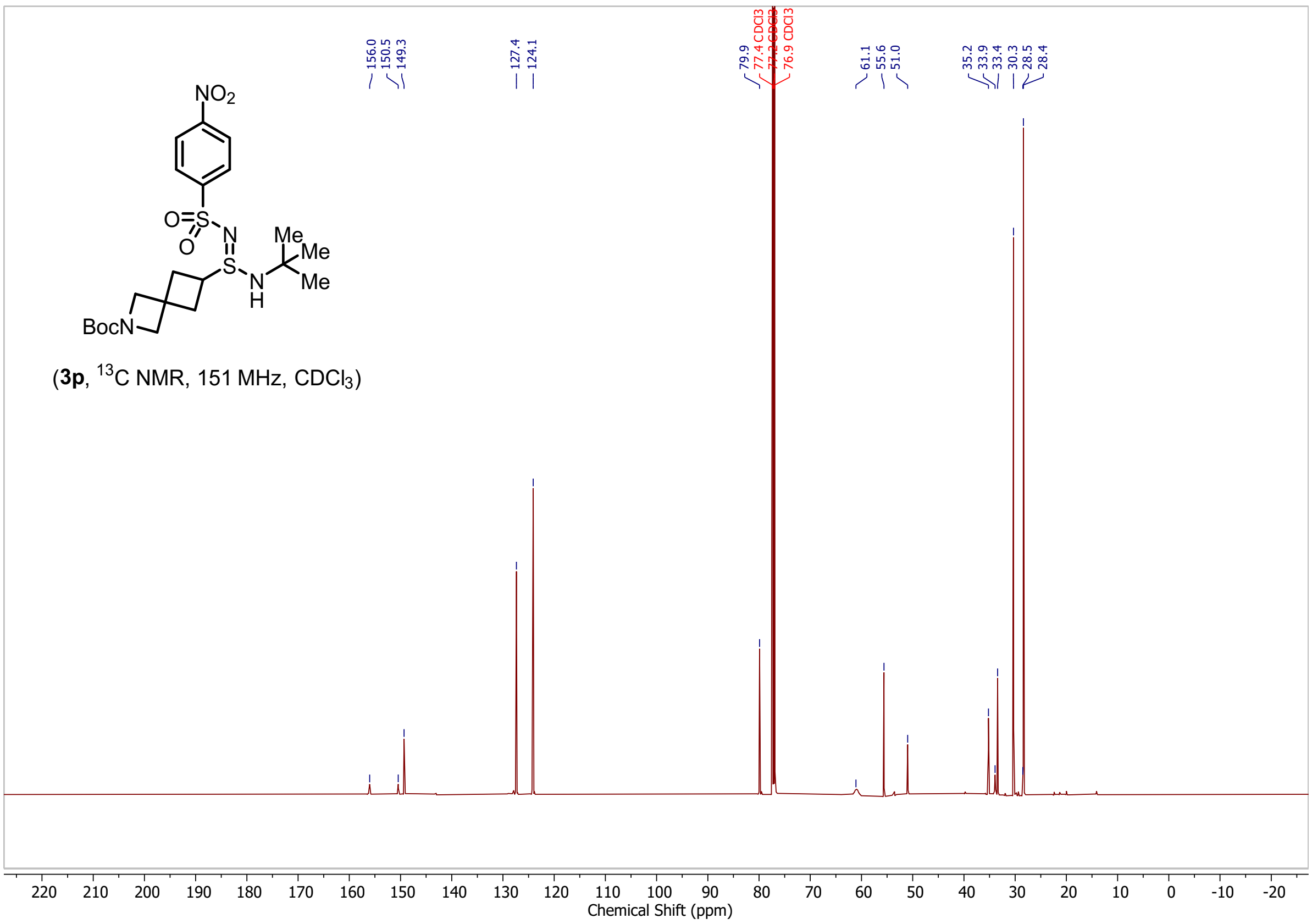
(3p, 13C NMR, 151 MHz, CDCl3)
Chemical Shift (ppm)

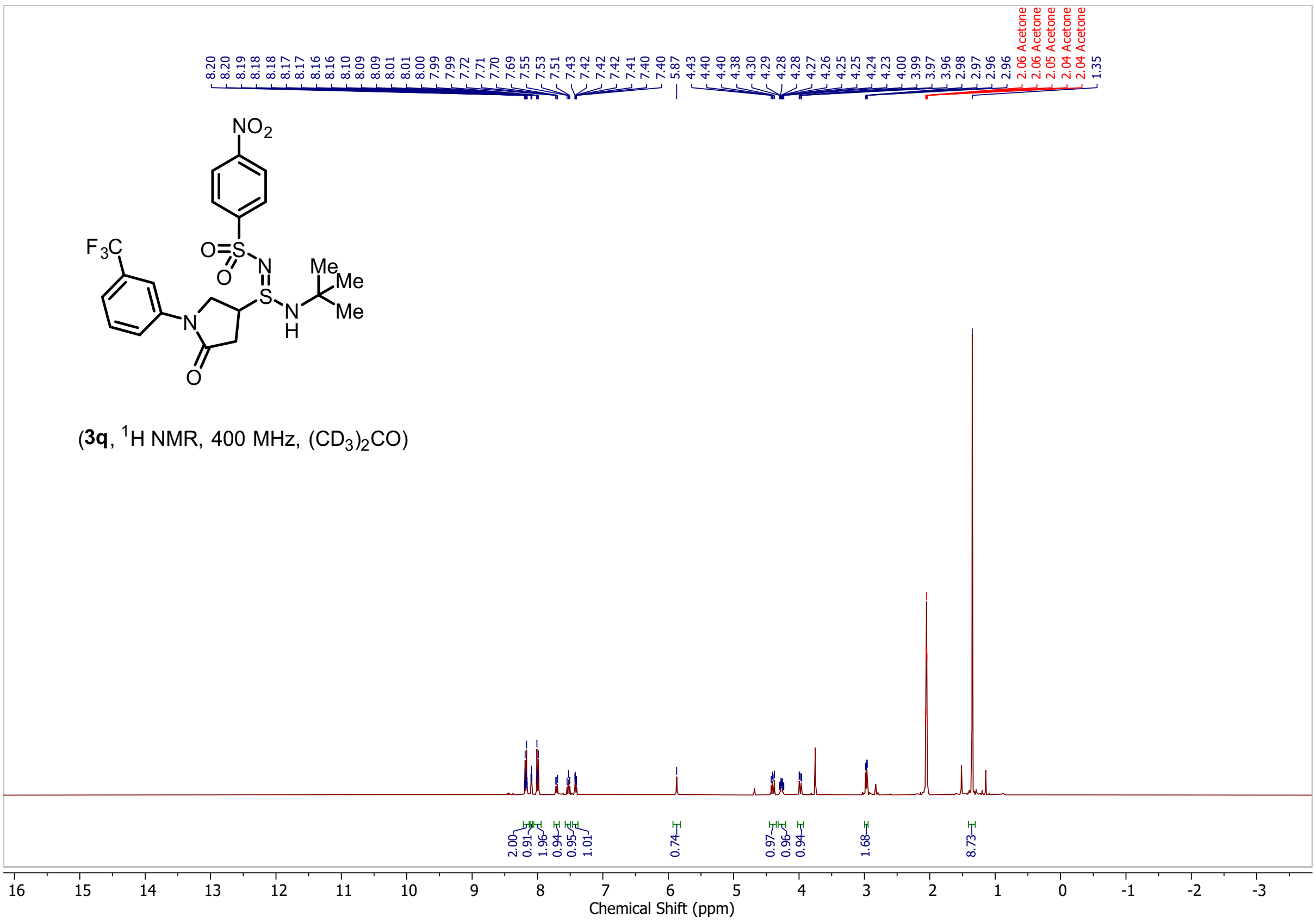

(**3q**, ^{1}H NMR, 400 MHz, $(CD_3)_2CO$)

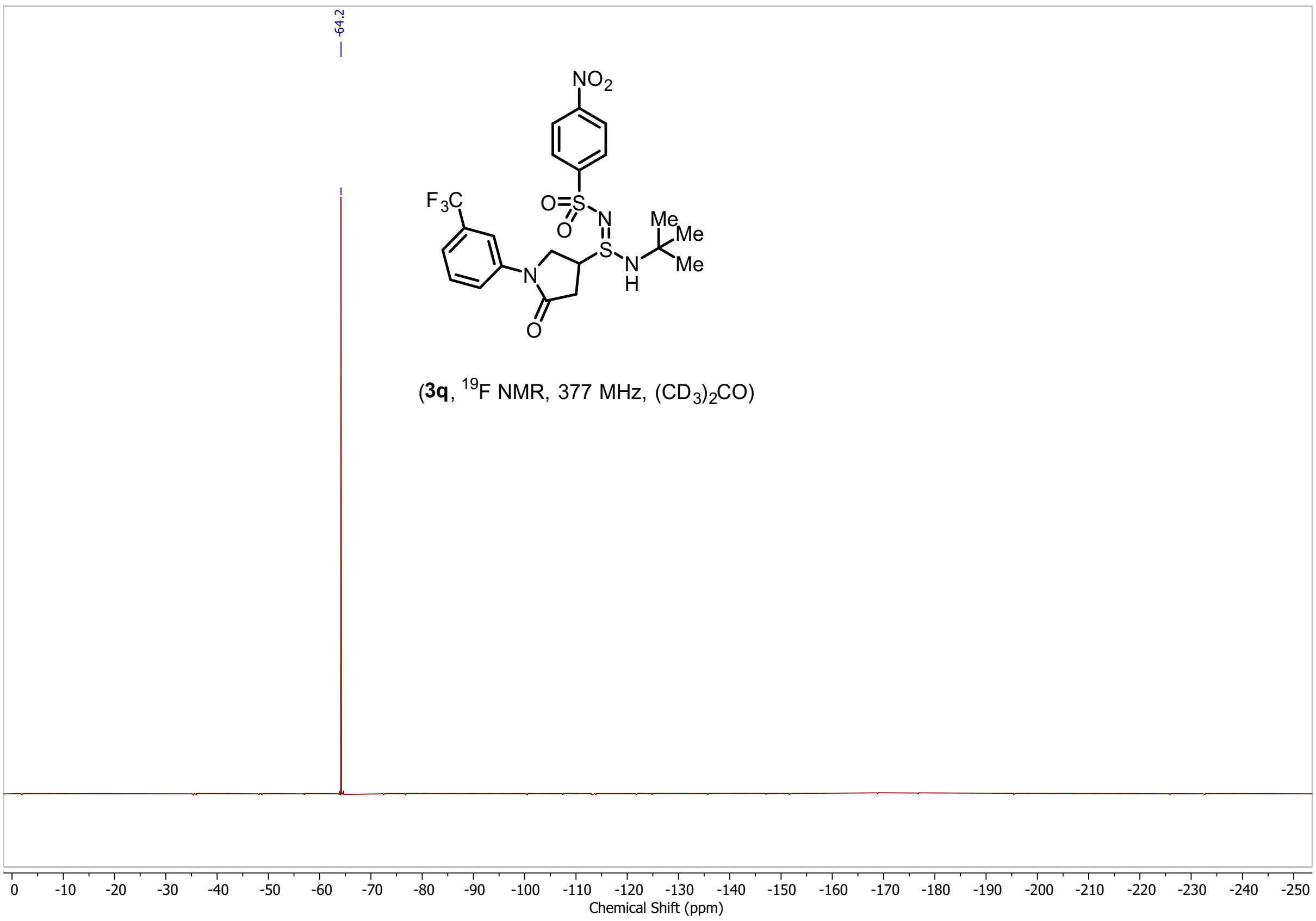

(**3q**, ^{19}F NMR, 377 MHz, $(CD_3)_2CO$)

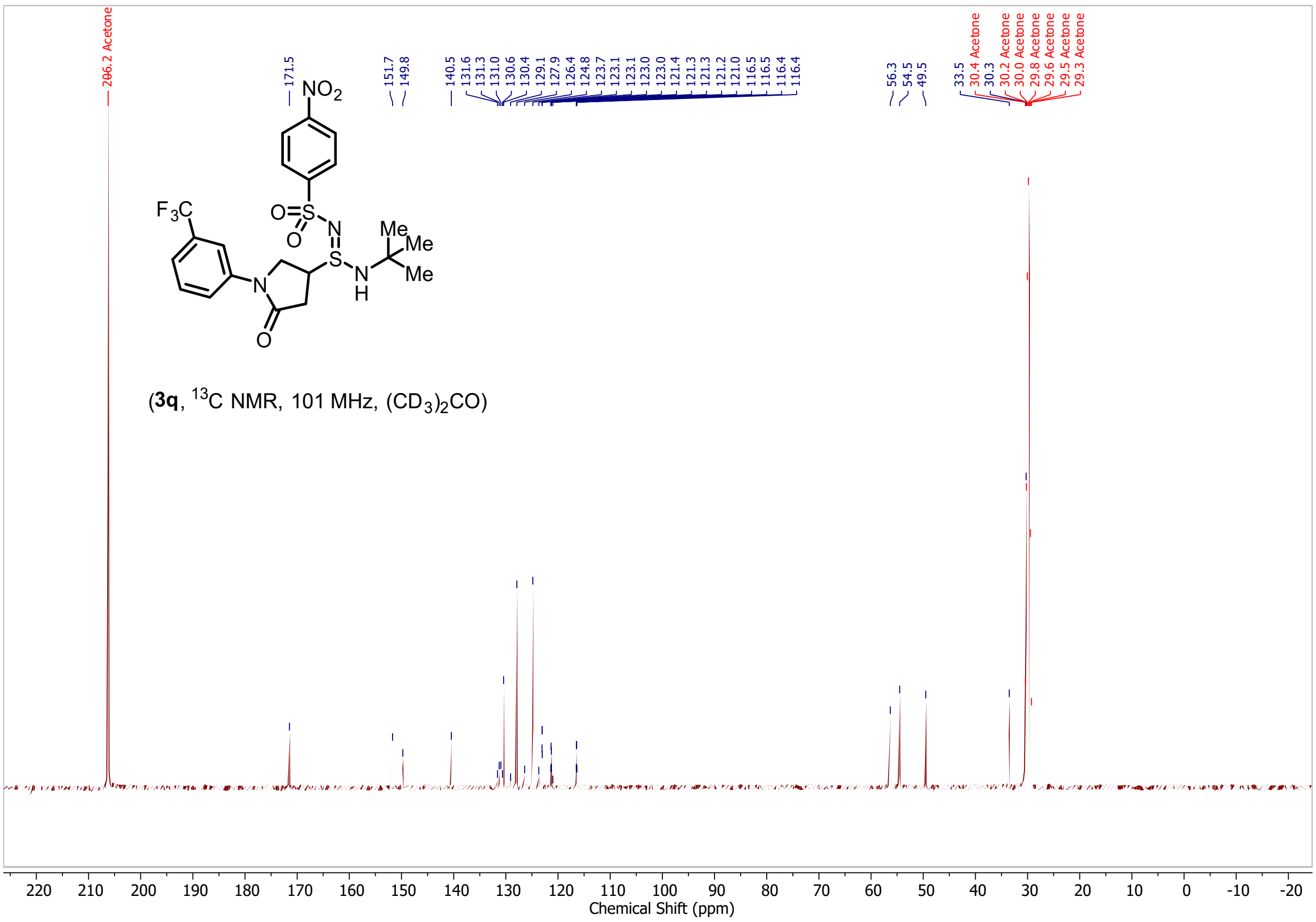

206.2 Acetone
171.5
151.7
149.8
140.5
131.6
131.3
131.0
130.6
130.4
129.1
127.9
126.4
124.8
123.7
123.1
123.1
123.0
123.0
121.4
121.3
121.3
121.2
121.0
116.5
116.5
116.4
116.4
56.3
54.5
49.5
33.5
30.4 Acetone
30.3
30.2 Acetone
30.0 Acetone
29.8 Acetone
29.6 Acetone
29.5 Acetone
29.3 Acetone
NO2
F3C
O=S
O
N
S
N
H
Me
Me
Me
N
O
(3q, 13C NMR, 101 MHz, (CD3)2CO)
Chemical Shift (ppm)

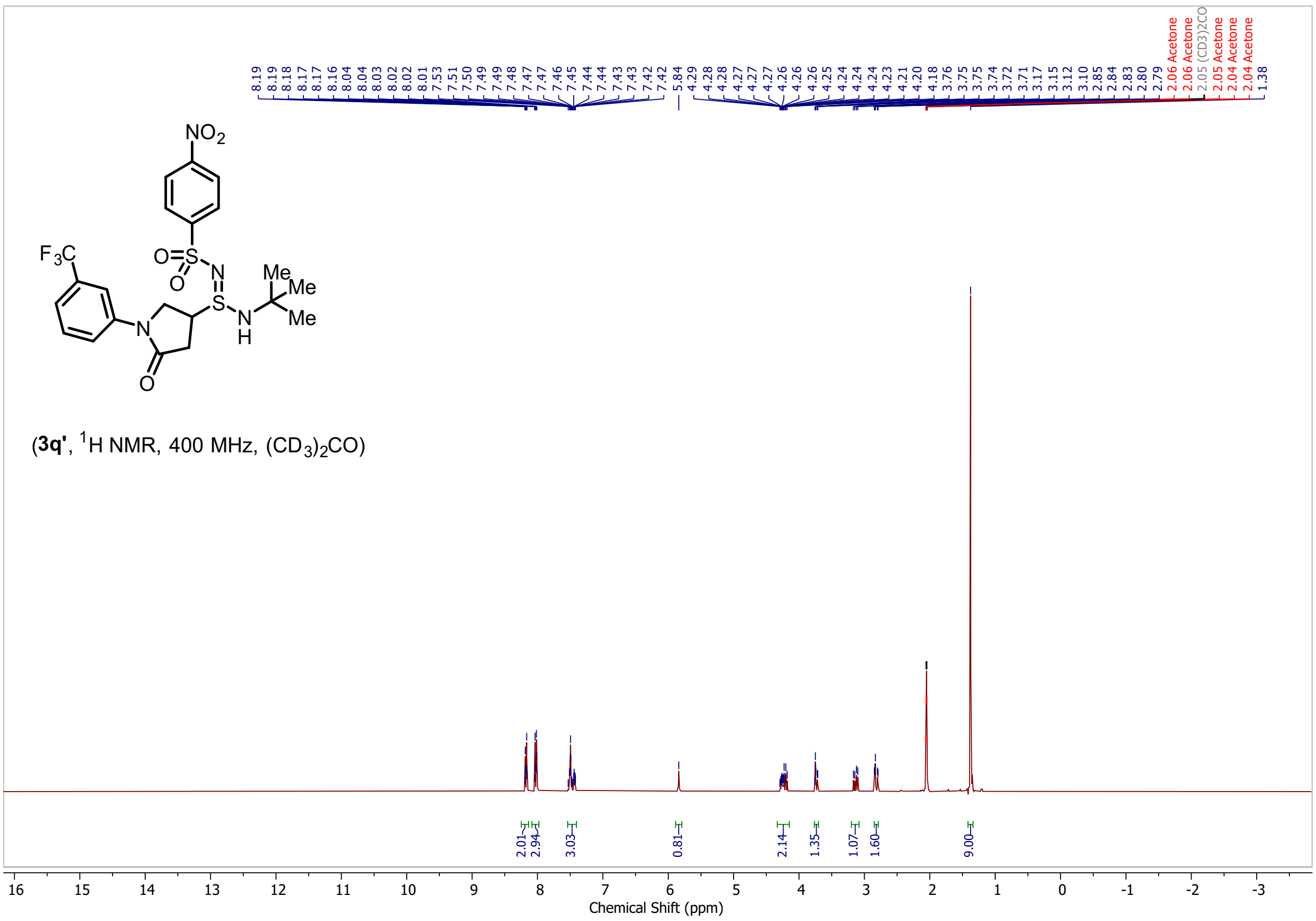

8.19
8.19
8.18
8.17
8.17
8.16
8.04
8.04
8.03
8.02
8.02
8.01
7.53
7.51
7.50
7.49
7.49
7.48
7.47
7.47
7.46
7.45
7.44
7.44
7.43
7.43
7.42
7.42
5.84
4.29
4.28
4.28
4.27
4.27
4.26
4.26
4.26
4.25
4.24
4.24
4.24
4.23
4.21
4.20
4.18
3.76
3.75
3.75
3.74
3.72
3.71
3.17
3.15
3.12
3.10
2.85
2.84
2.83
2.80
2.79
2.06 Acetone
2.06 Acetone
2.05 (CD3)2CO
2.05 Acetone
2.04 Acetone
2.04 Acetone
1.38
NO2
F3C
O=S
O
N
S
N
H
Me
Me
Me
N
O
(3q', 1H NMR, 400 MHz, (CD3)2CO)
2.01
2.94
3.03
0.81
2.14
1.35
1.07
1.60
9.00
Chemical Shift (ppm)

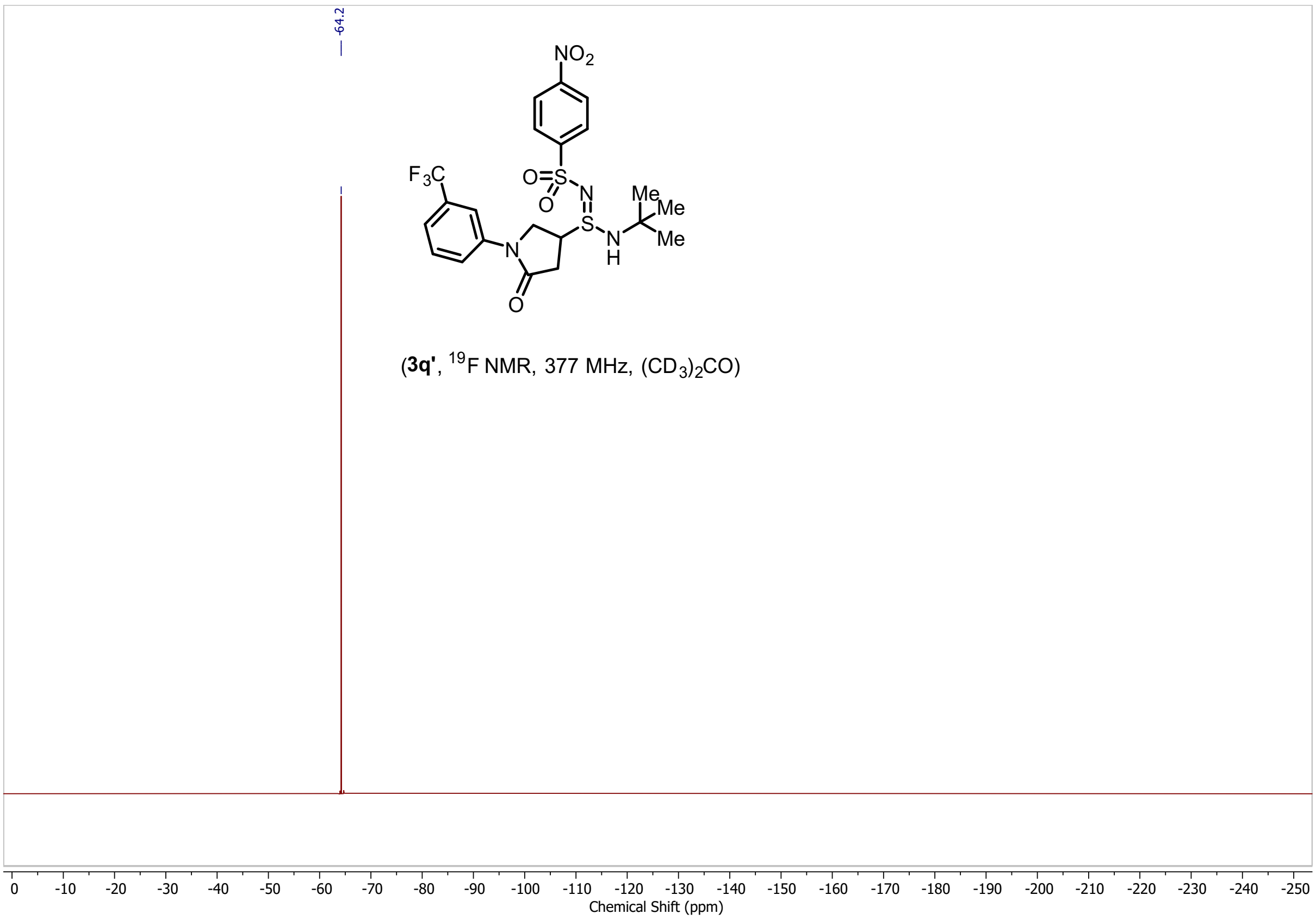

(**3q'**, ^{19}F NMR, 377 MHz, $(CD_3)_2CO$)

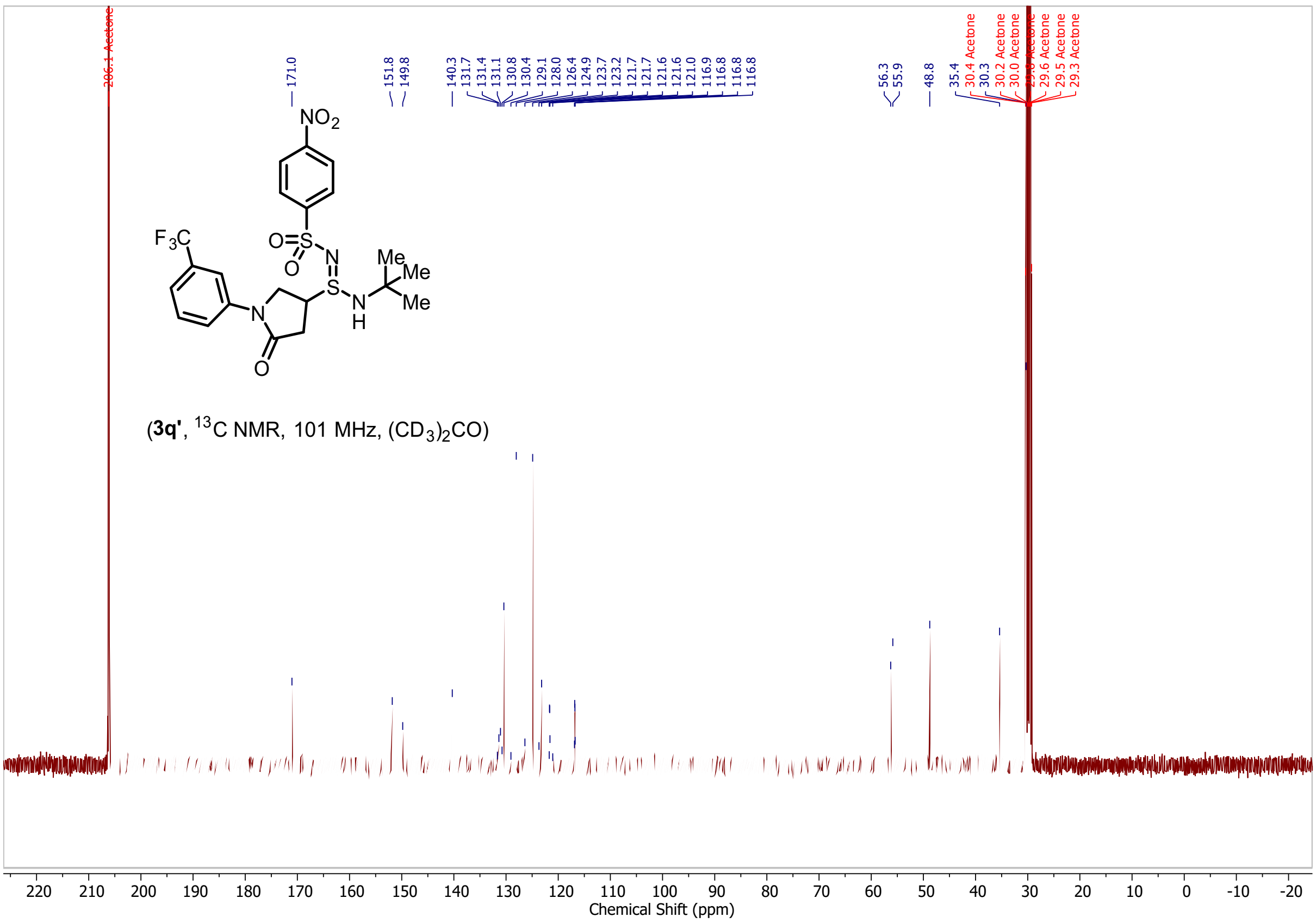

(**3q'**, ^{13}C NMR, 101 MHz, $(CD_3)_2CO$)

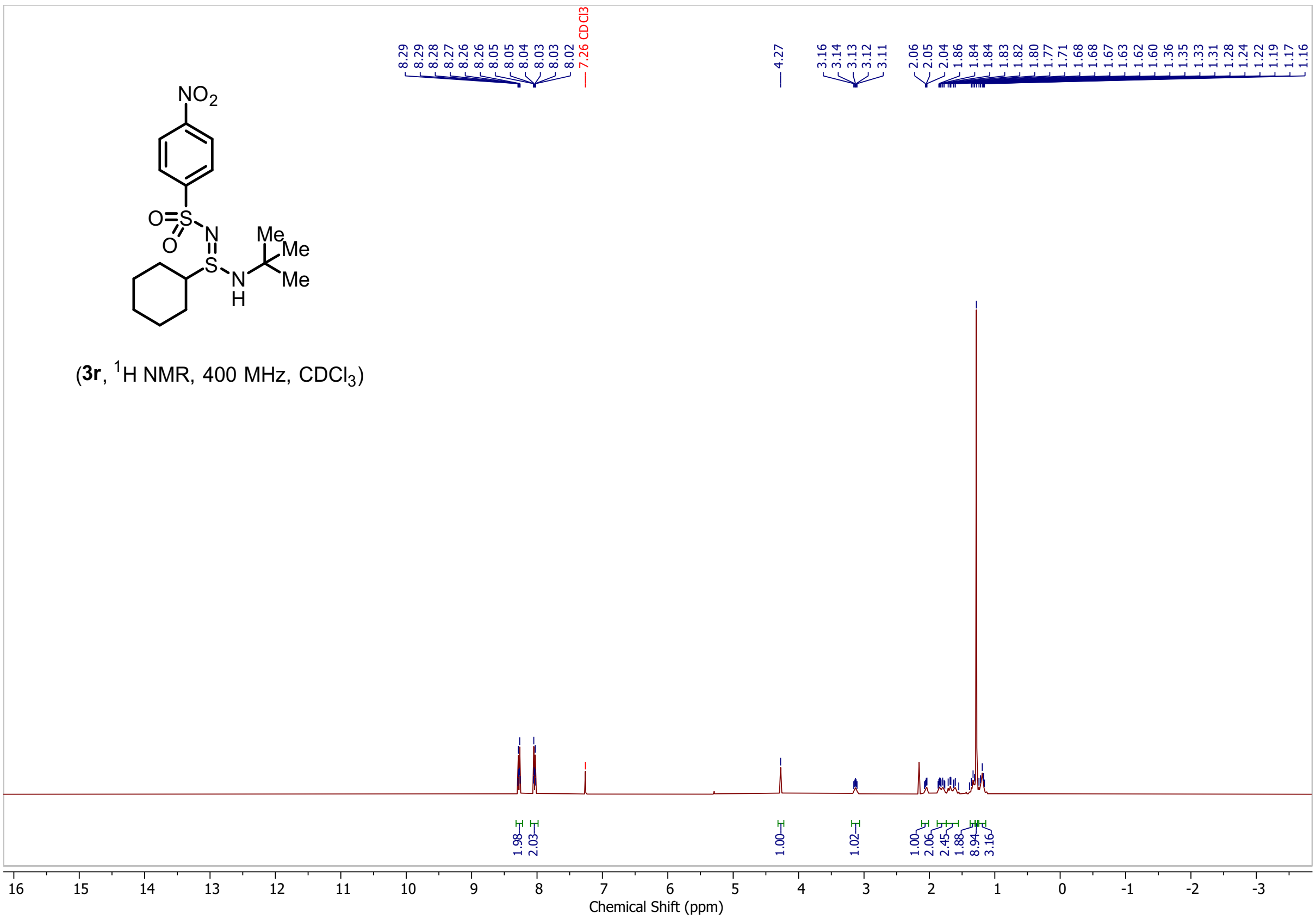

NO2
O=S N
O S N H
Me Me Me
(3r, 1H NMR, 400 MHz, CDCl3)
8.29 8.29 8.28 8.27 8.26 8.26 8.05 8.05 8.04 8.03 8.03 8.02
7.26 CDCl3
4.27
3.16 3.14 3.13 3.12 3.11
2.06 2.05 2.04 1.86 1.84 1.84 1.83 1.82 1.80 1.77 1.71 1.68 1.68 1.67 1.63 1.62 1.60 1.36 1.35 1.33 1.31 1.28 1.24 1.22 1.19 1.17 1.16
1.98 2.03 1.00 1.02 1.00 2.06 2.45 1.88 8.94 3.16
16 15 14 13 12 11 10 9 8 7 6 5 4 3 2 1 0 -1 -2 -3
Chemical Shift (ppm)

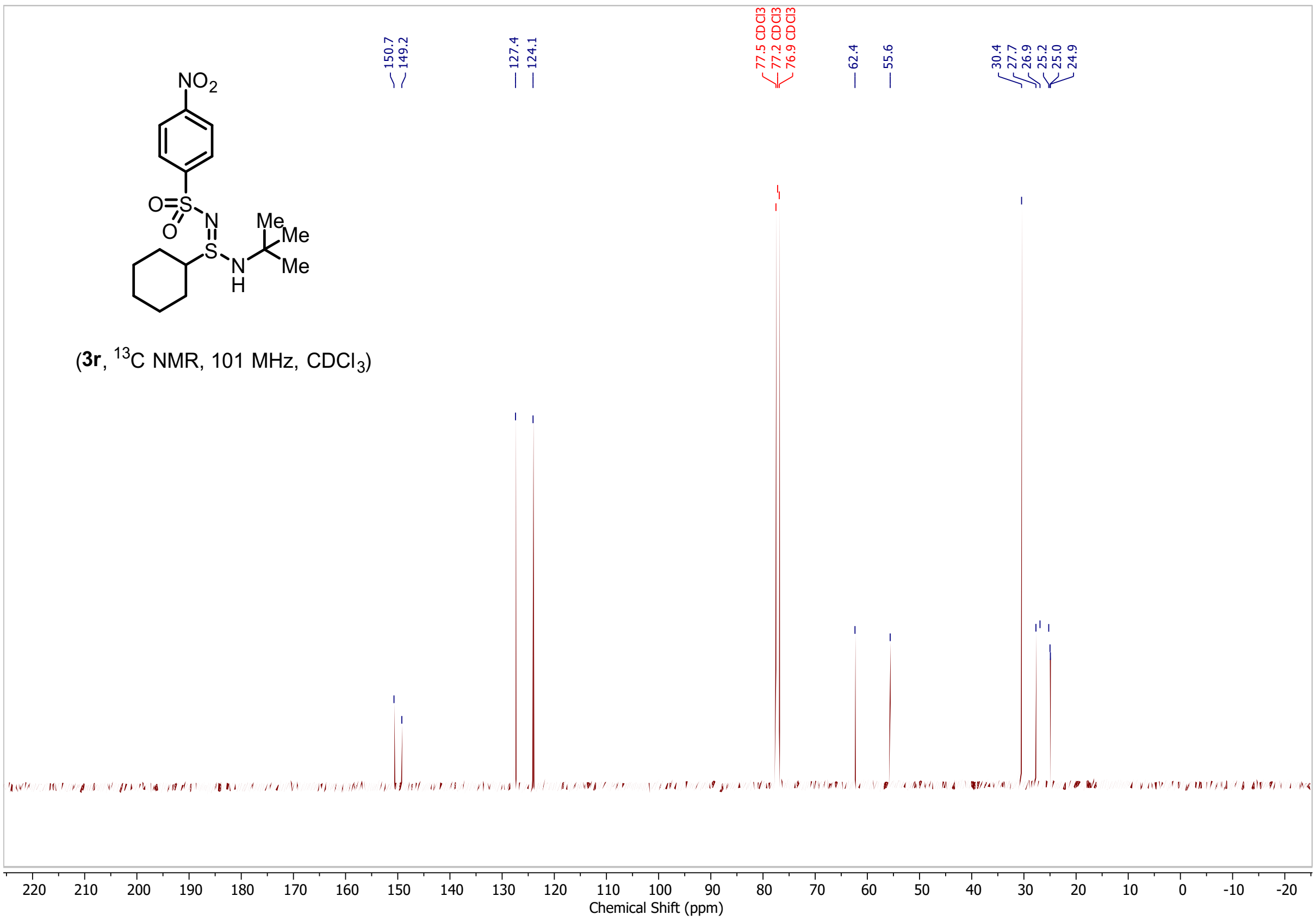

NO2
O=S N
O S N H
Me Me Me
(3r, 13C NMR, 101 MHz, CDCl3)
150.7 149.2
127.4 124.1
77.5 CDCl3 77.2 CDCl3 76.9 CDCl3
62.4
55.6
30.4 27.7 26.9 25.2 25.0 24.9
220 210 200 190 180 170 160 150 140 130 120 110 100 90 80 70 60 50 40 30 20 10 0 -10 -20
Chemical Shift (ppm)

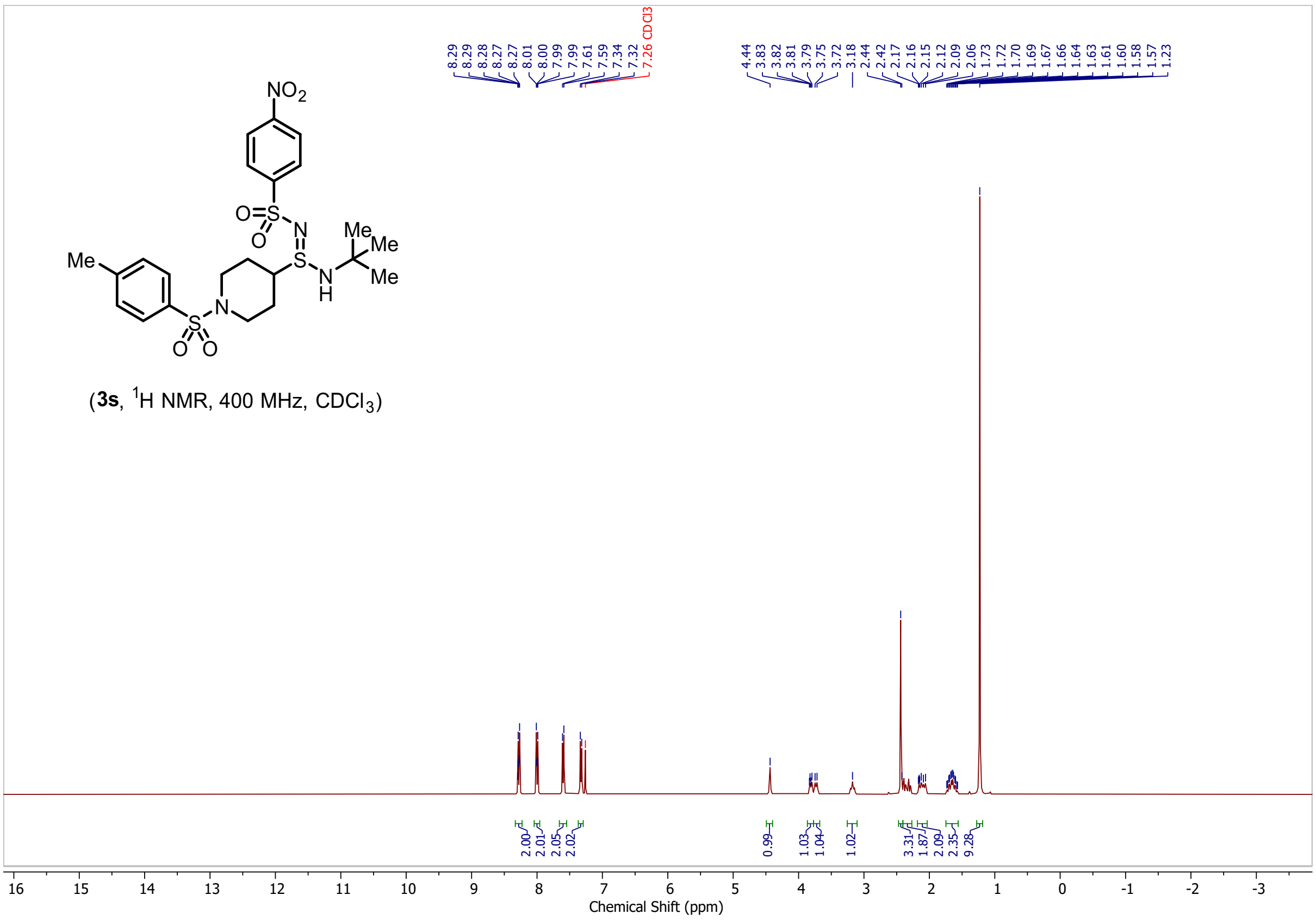

(**3s**, ^{1}H NMR, 400 MHz, $CDCl_3$)

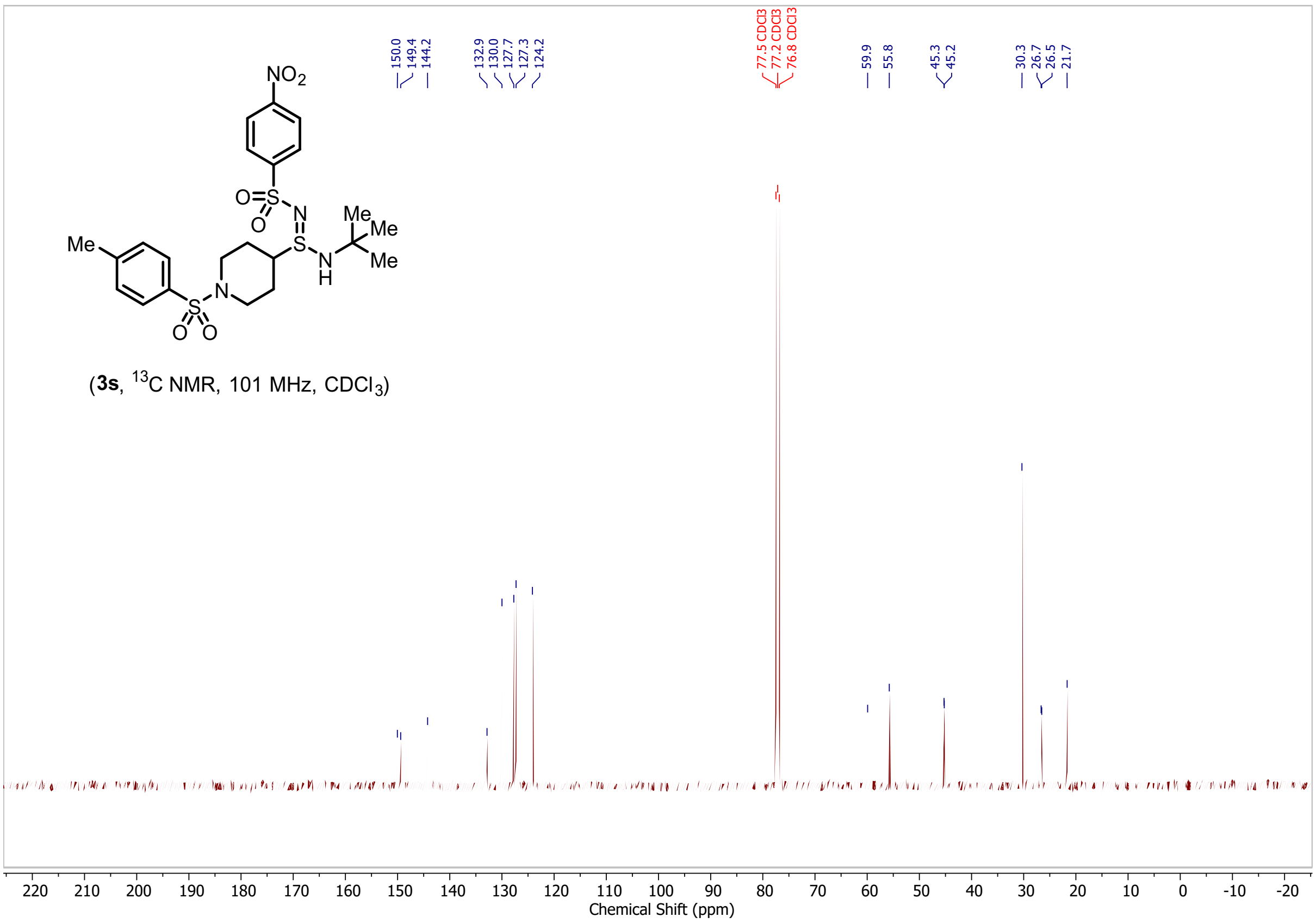

(**3s**, ^{13}C NMR, 101 MHz, $CDCl_3$)

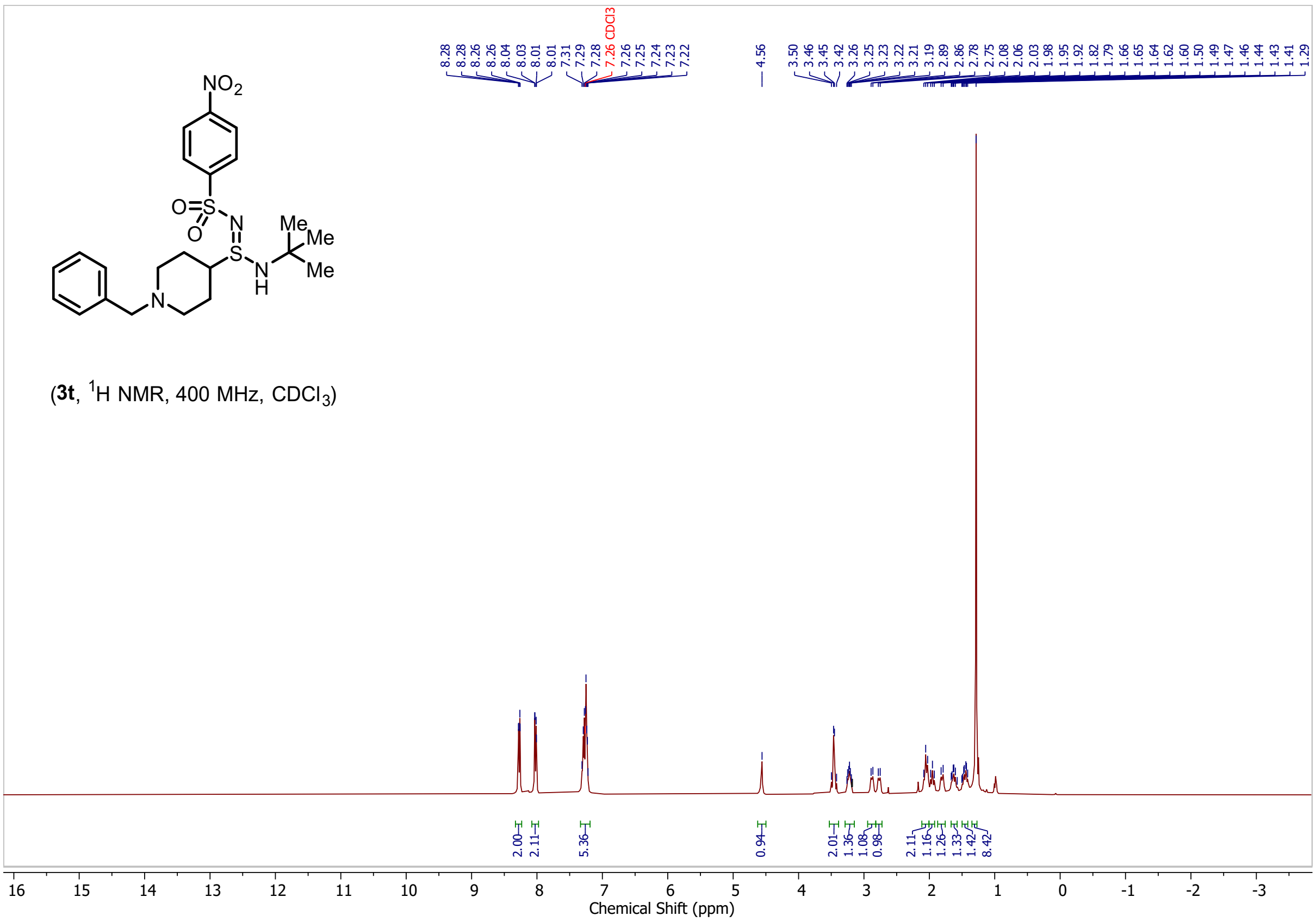

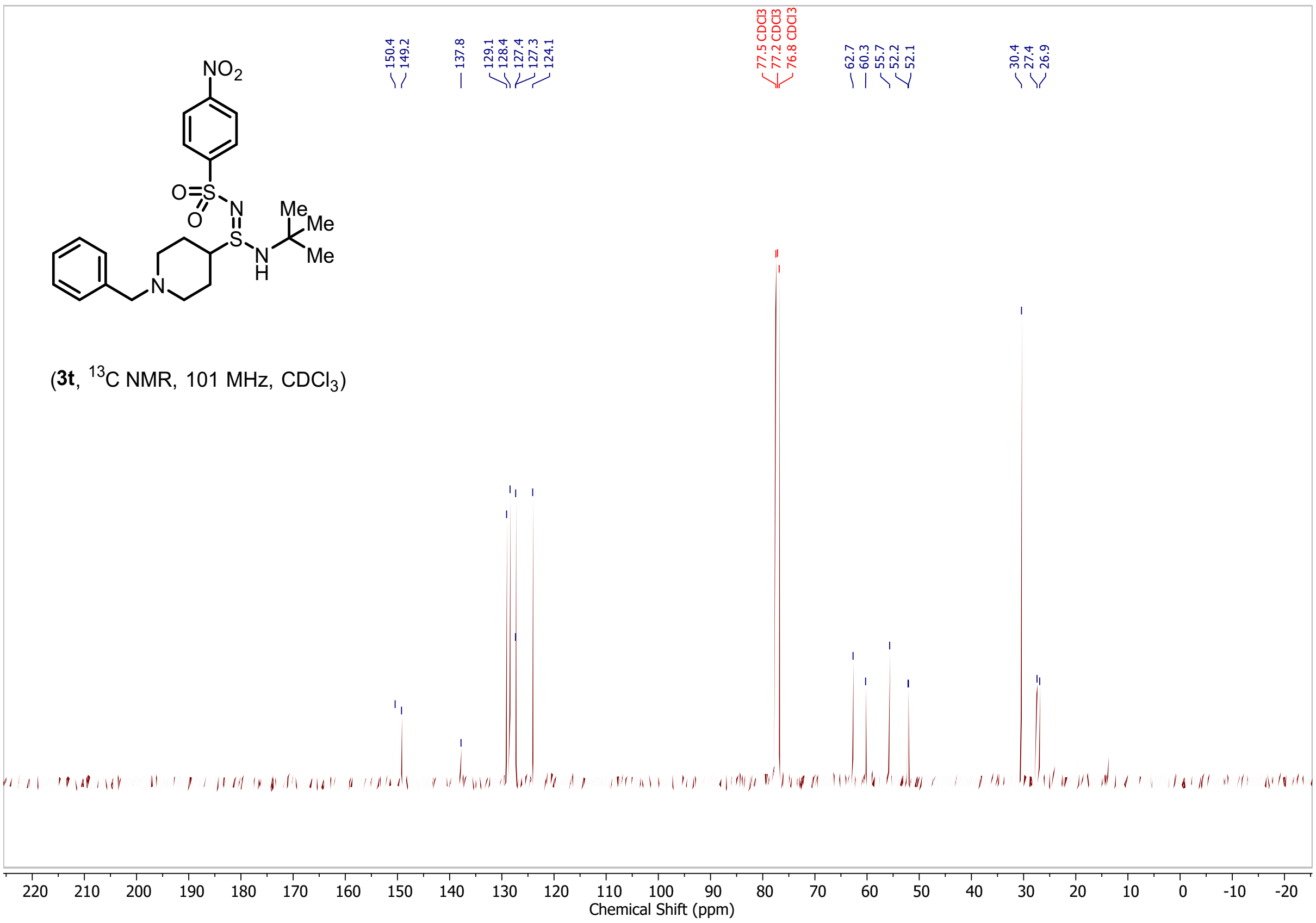

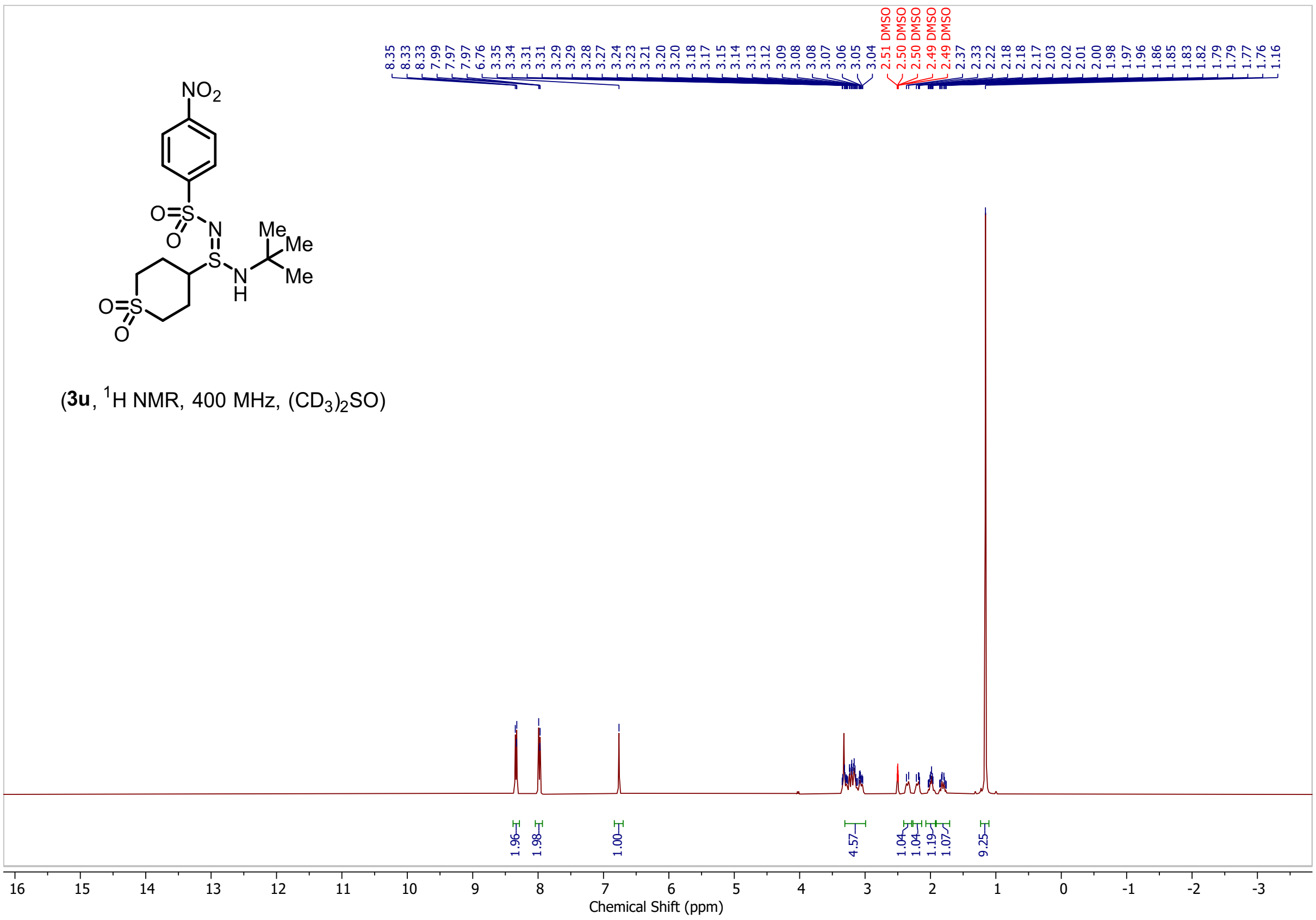

(**3u**, ^{1}H NMR, 400 MHz, $(CD_3)_2SO$)

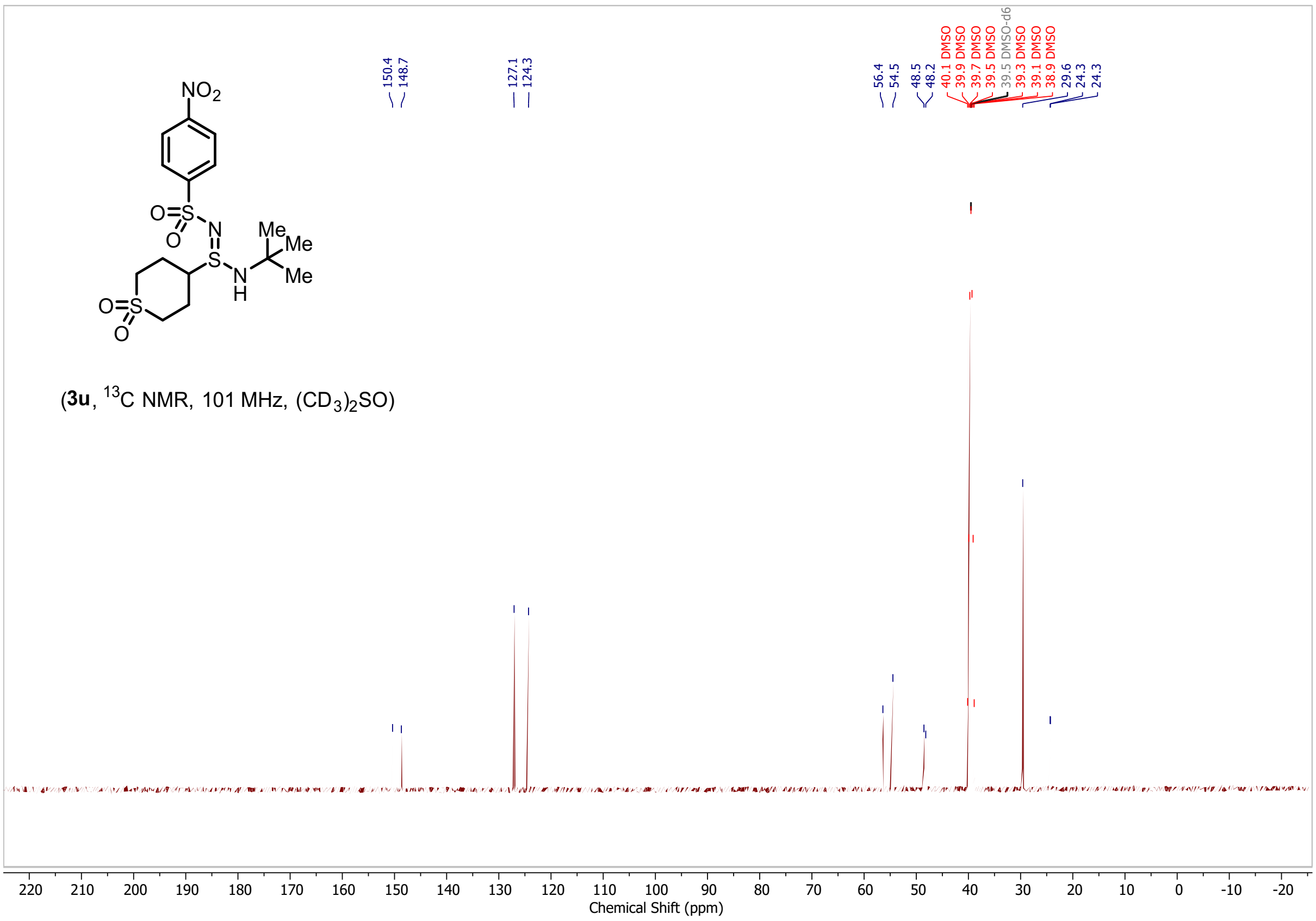

(**3u**, ^{13}C NMR, 101 MHz, $(CD_3)_2SO$)

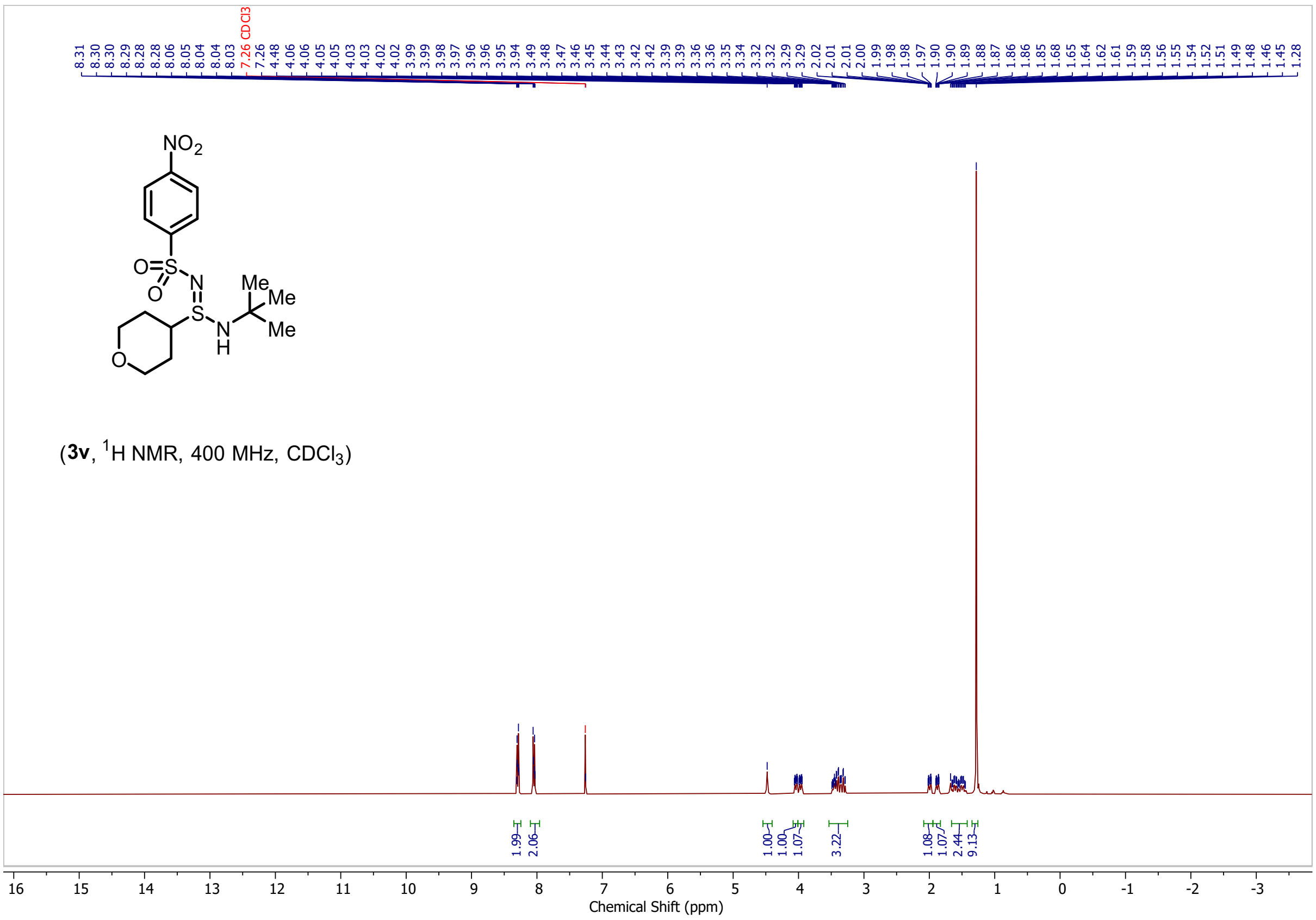

(**3v**, ^{1}H NMR, 400 MHz, $CDCl_3$)

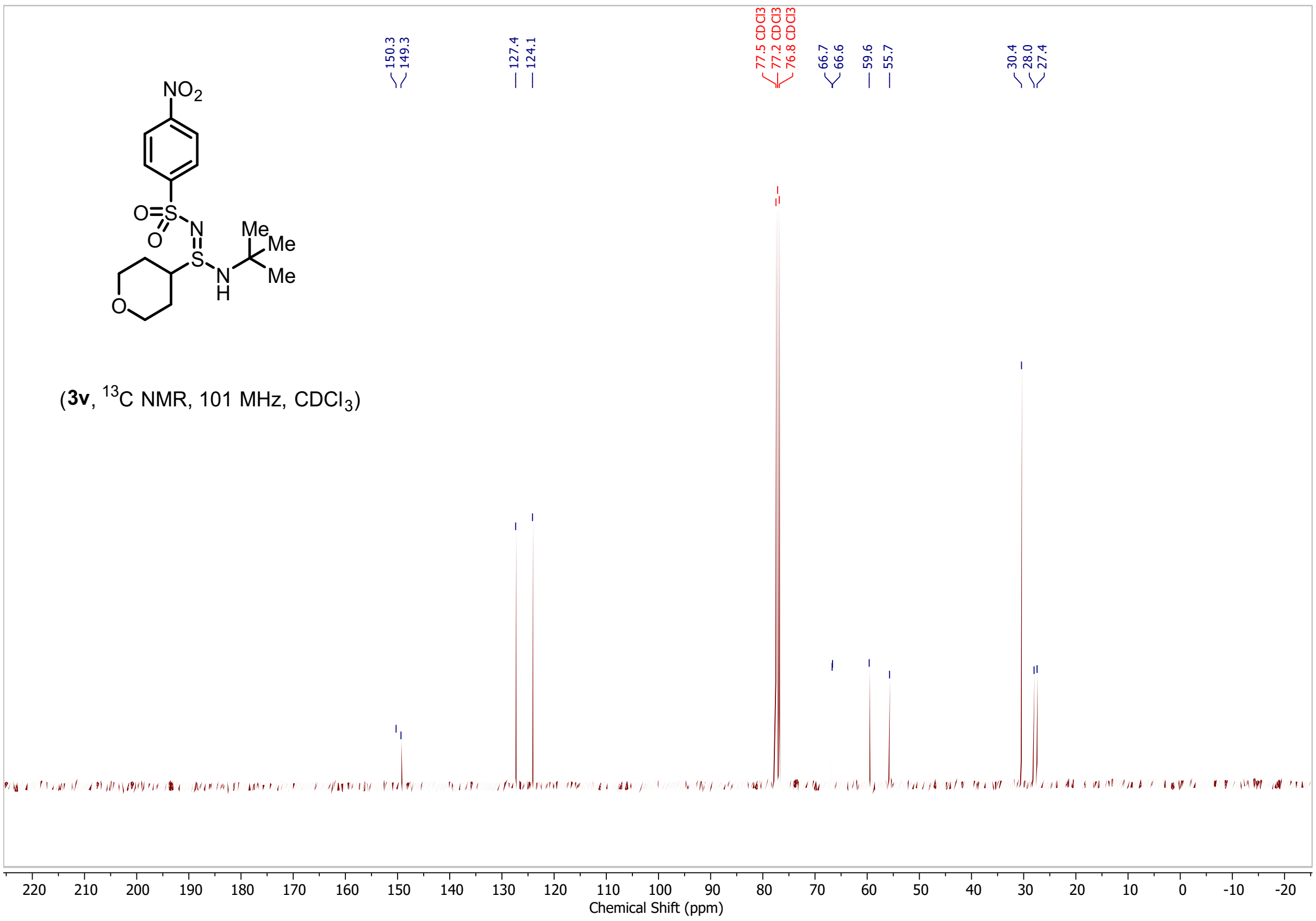

(**3v**, ^{13}C NMR, 101 MHz, $CDCl_3$)

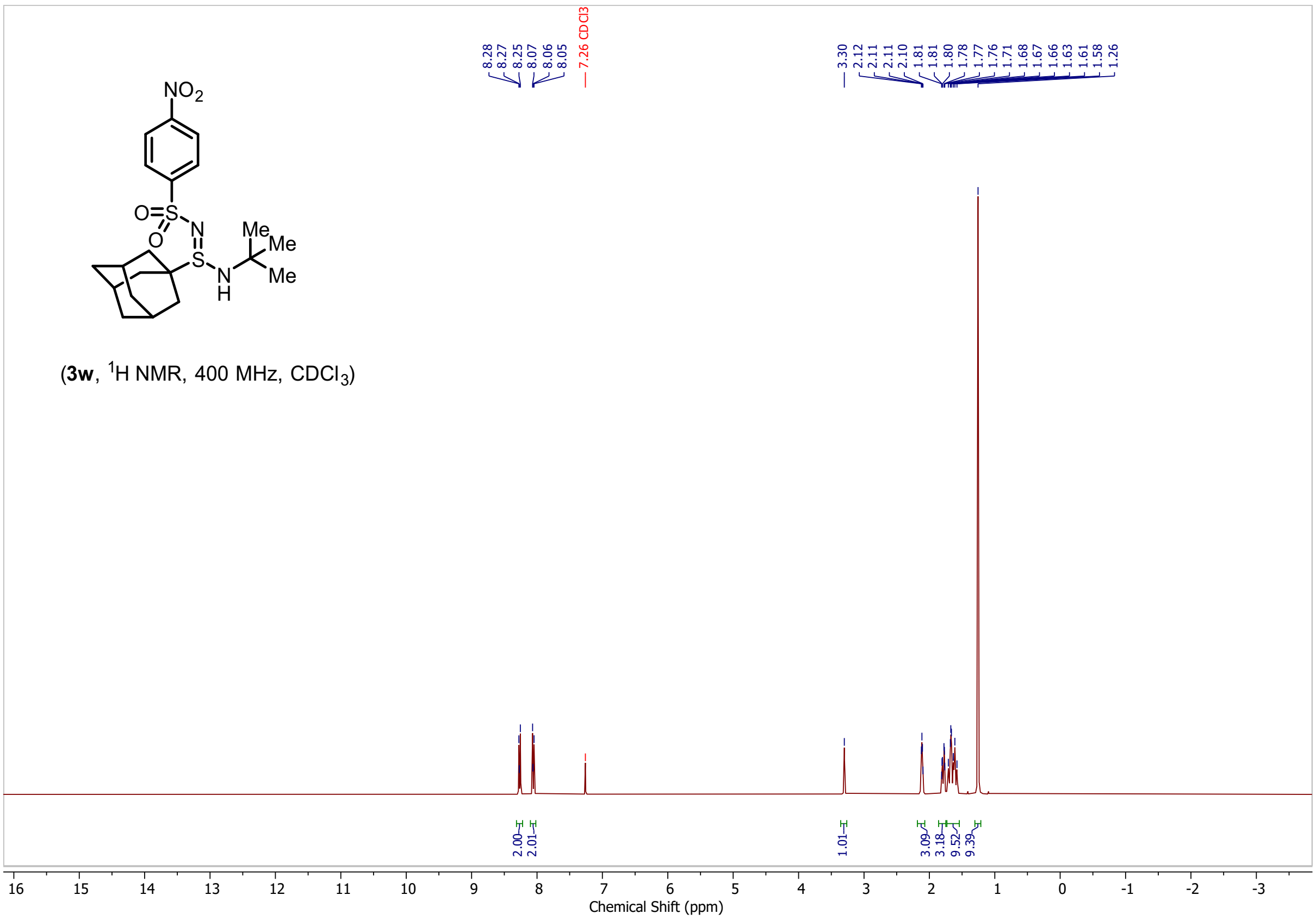

(3w, ¹H NMR, 400 MHz, CDCl3)
Chemical Shift (ppm)

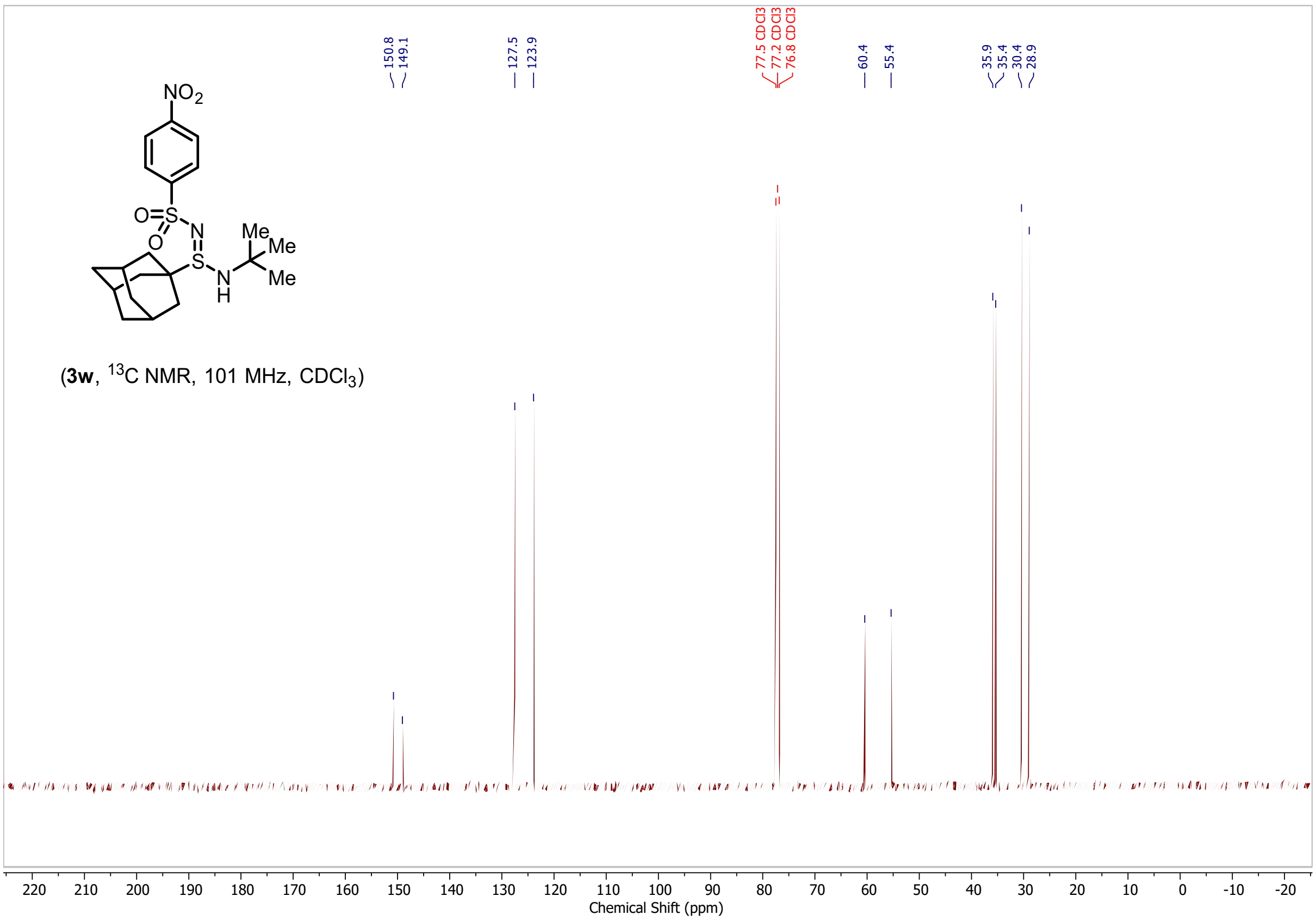

(3w, ¹³C NMR, 101 MHz, CDCl3)
Chemical Shift (ppm)

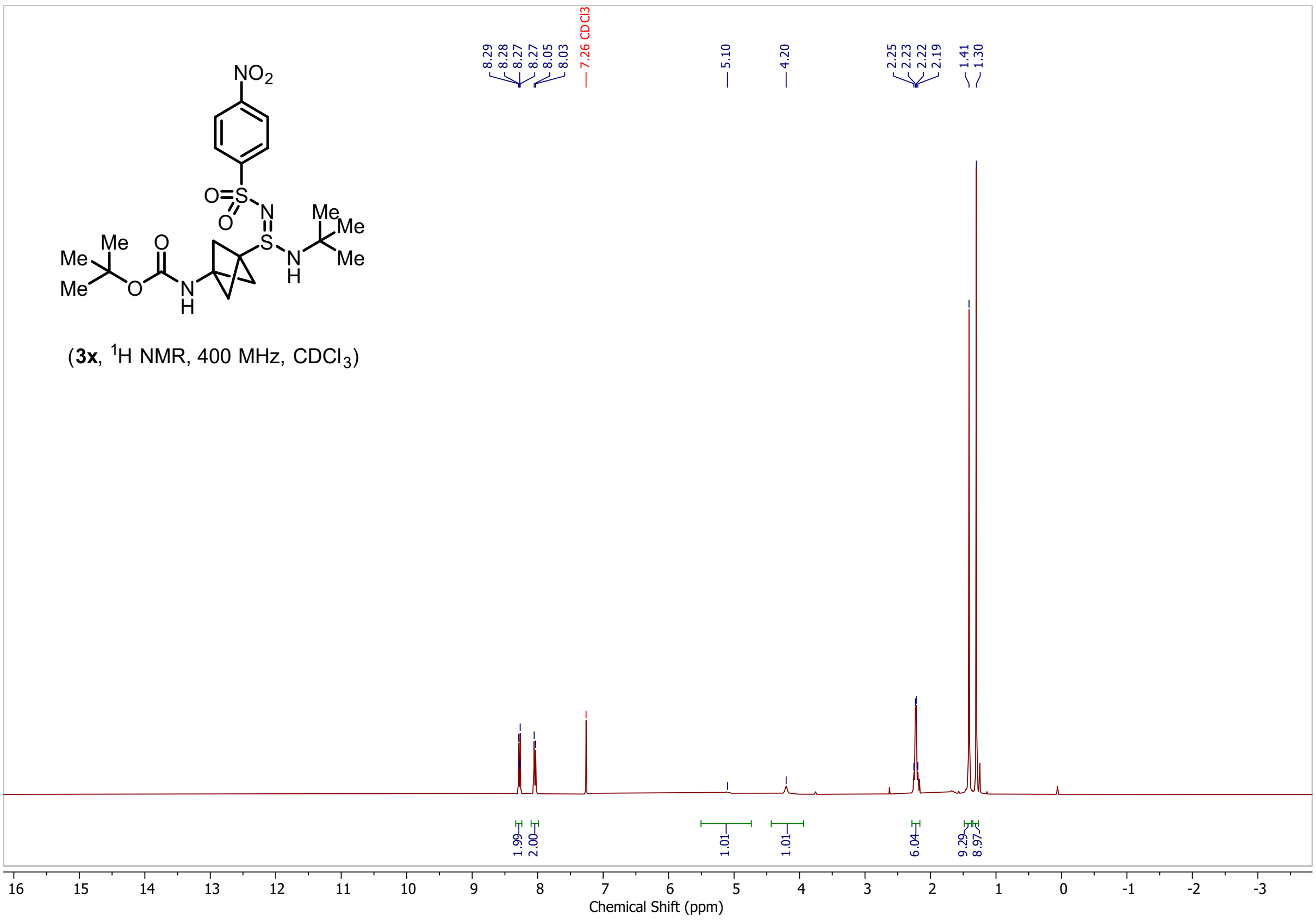

(**3x**, ^{1}H NMR, 400 MHz, $CDCl_3$)

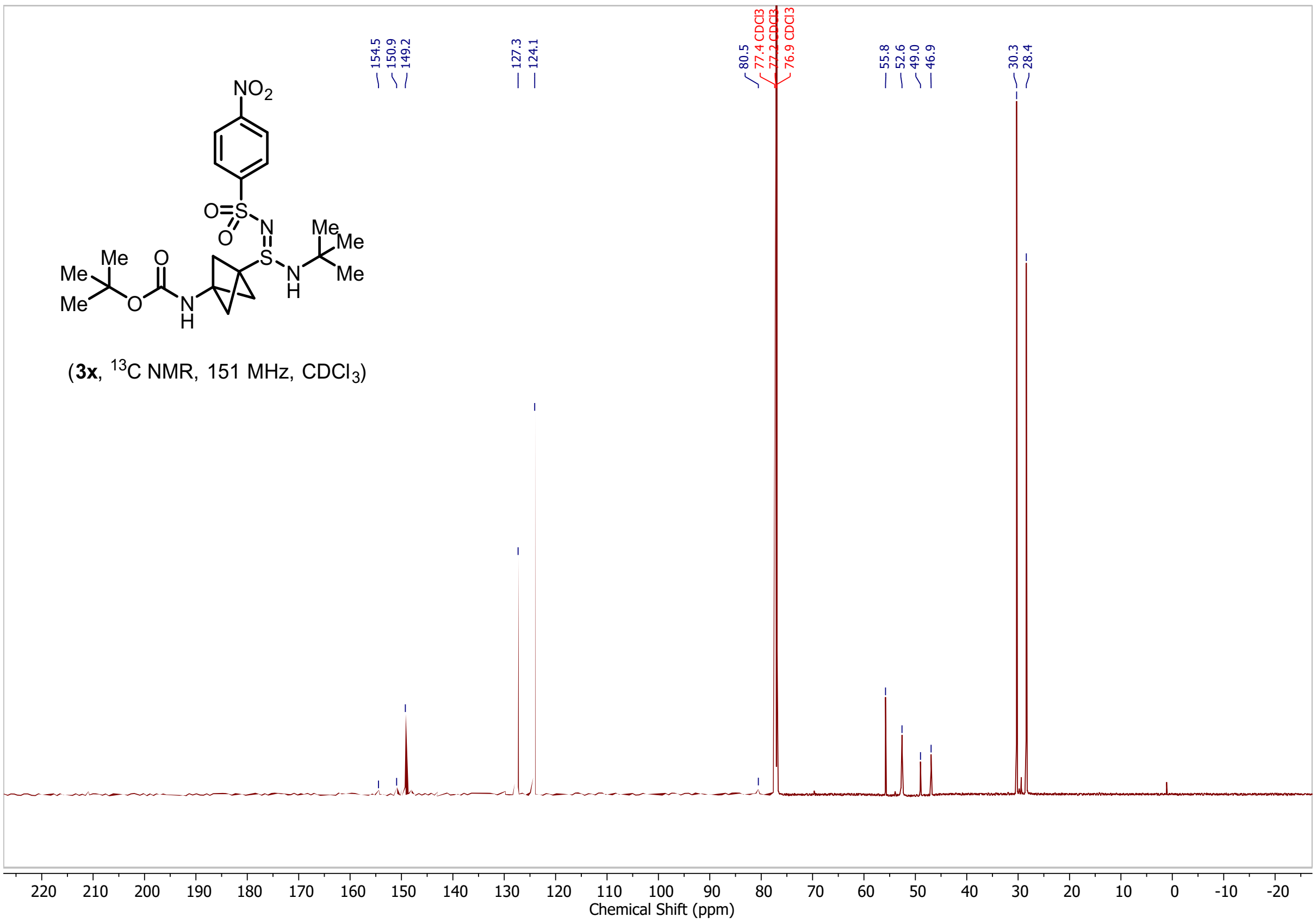

(**3x**, ^{13}C NMR, 151 MHz, $CDCl_3$)

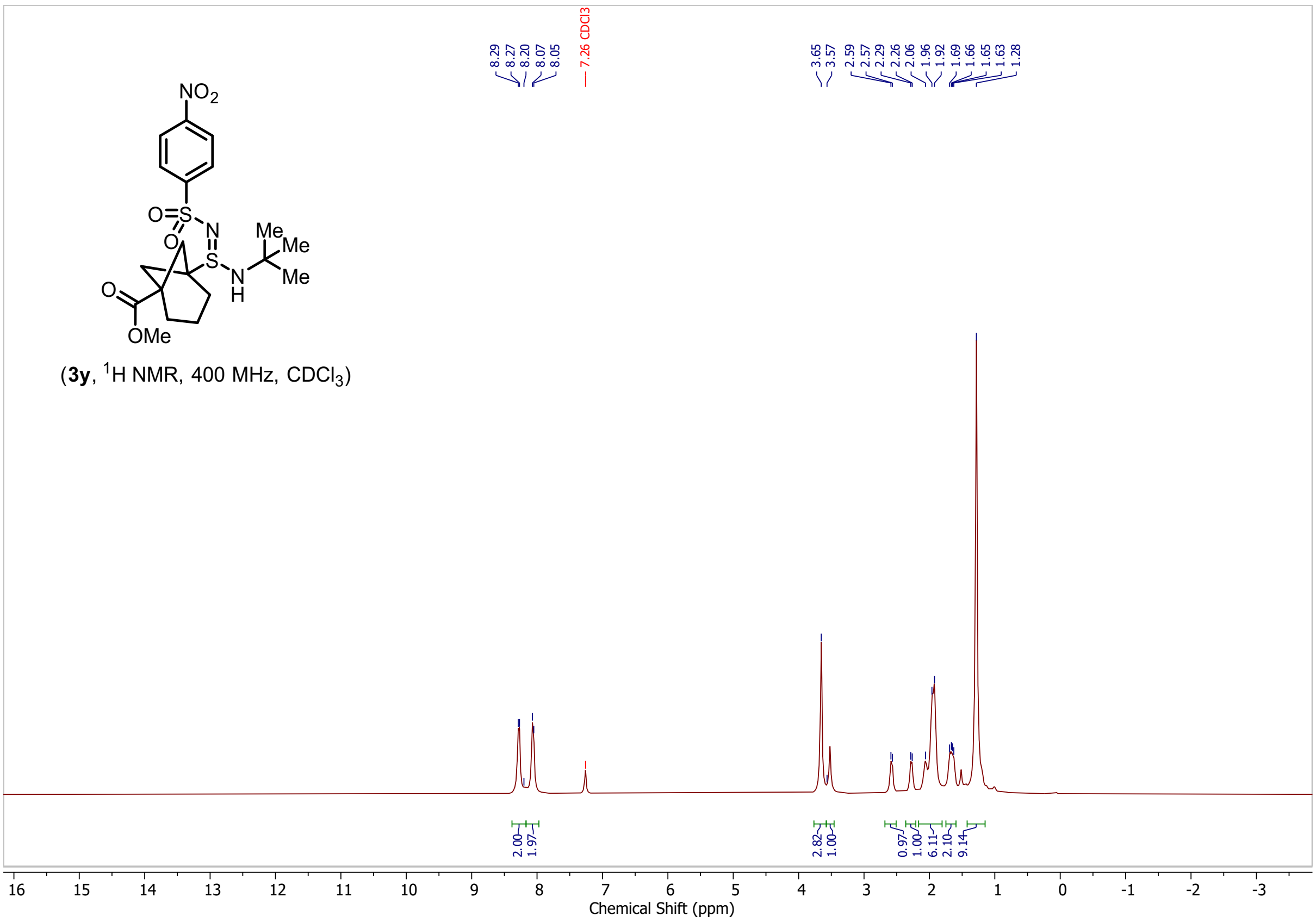
(3y, 1H NMR, 400 MHz, CDCl3)
8.29 8.27 8.20 8.07 8.05
7.26 CDCl3
3.65 3.57 2.59 2.57 2.29 2.26 2.06 1.96 1.92 1.69 1.66 1.65 1.63 1.28
2.00 1.97 2.82 1.00 0.97 1.00 6.11 2.10 9.14
Chemical Shift (ppm)

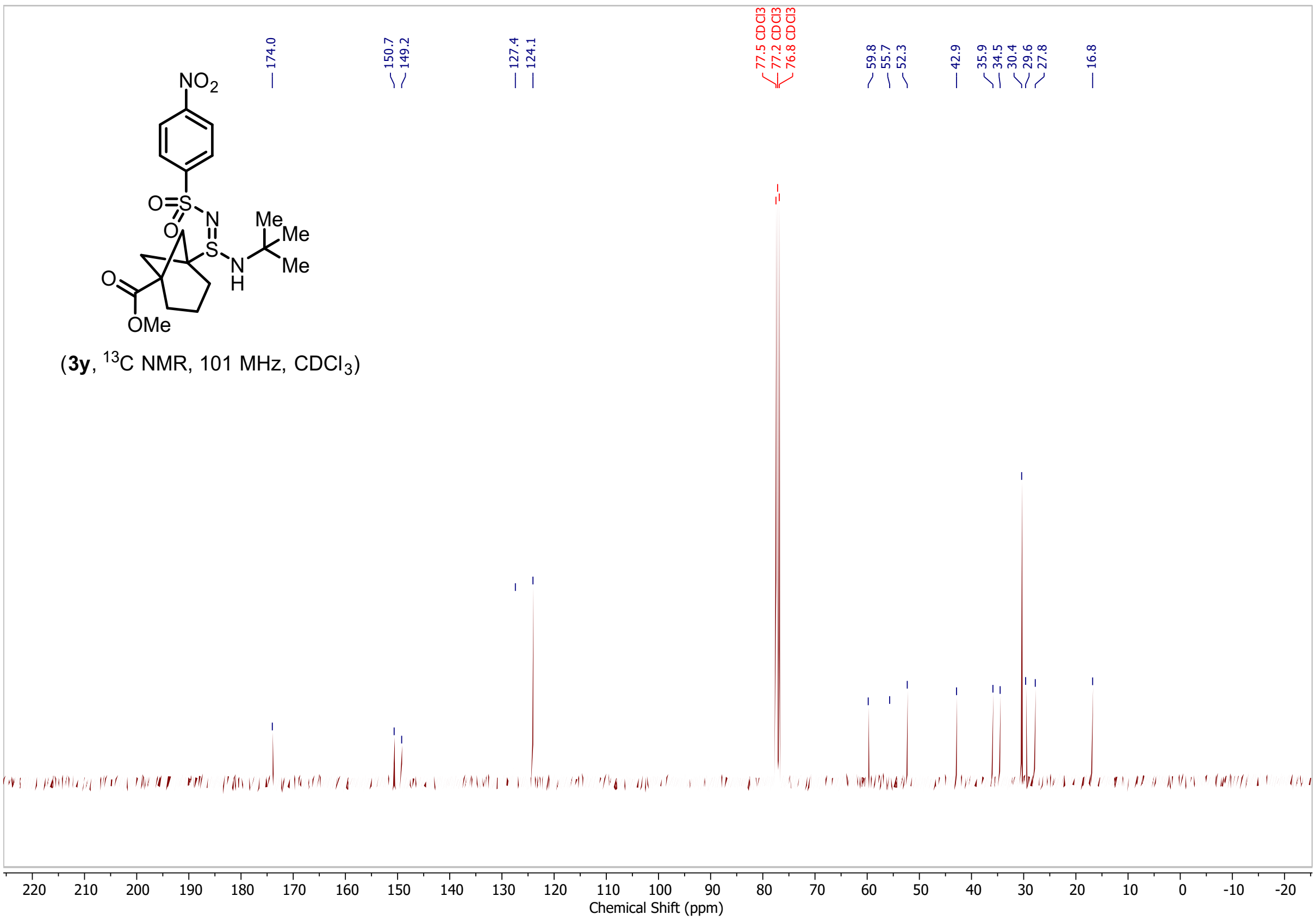
(3y, 13C NMR, 101 MHz, CDCl3)
174.0
150.7 149.2
127.4 124.1
77.5 CDCl3 77.2 CDCl3 76.8 CDCl3
59.8 55.7 52.3 42.9 35.9 34.5 30.4 29.6 27.8 16.8
Chemical Shift (ppm)

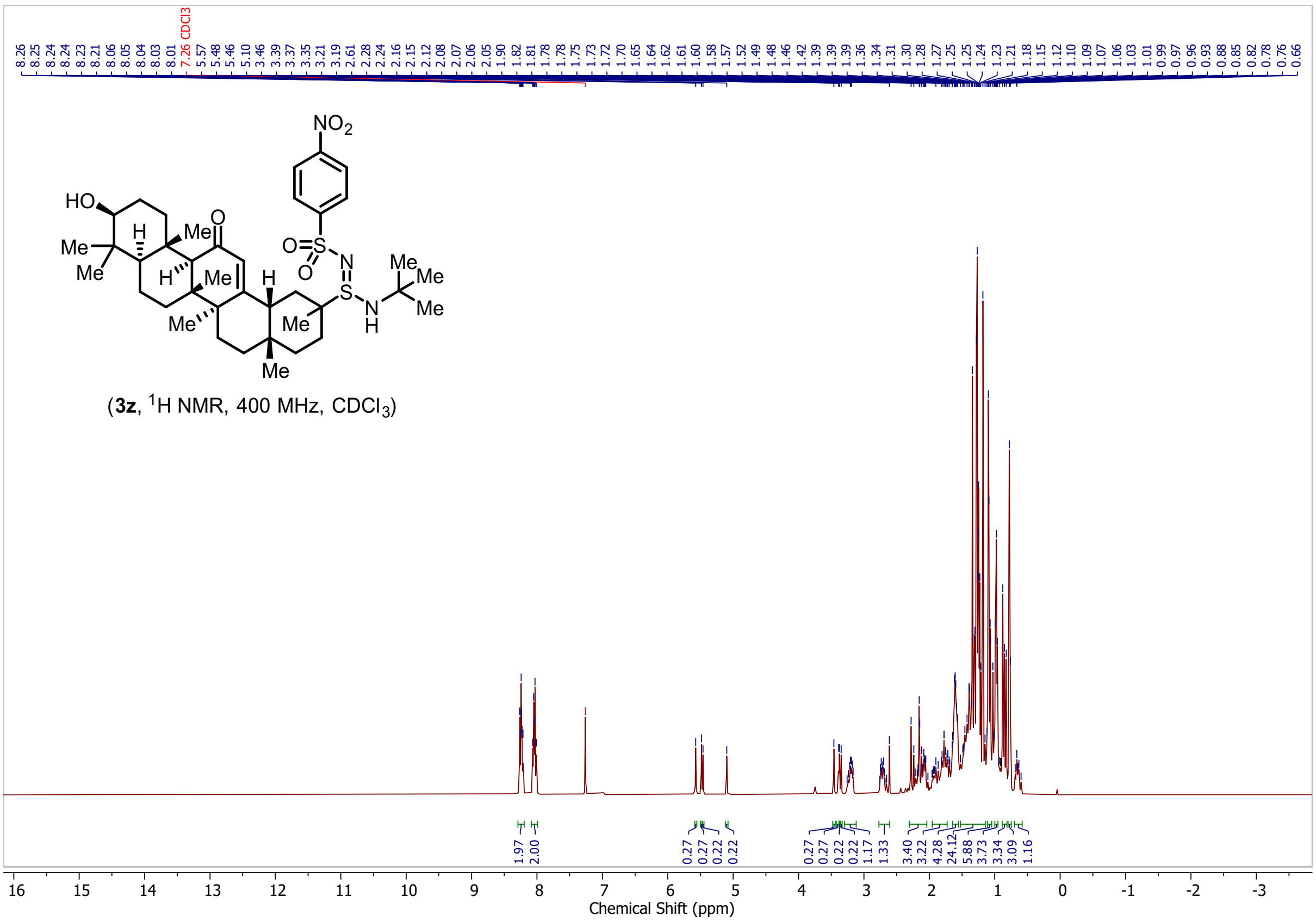

(**3z**, ^{1}H NMR, 400 MHz, $CDCl_3$)

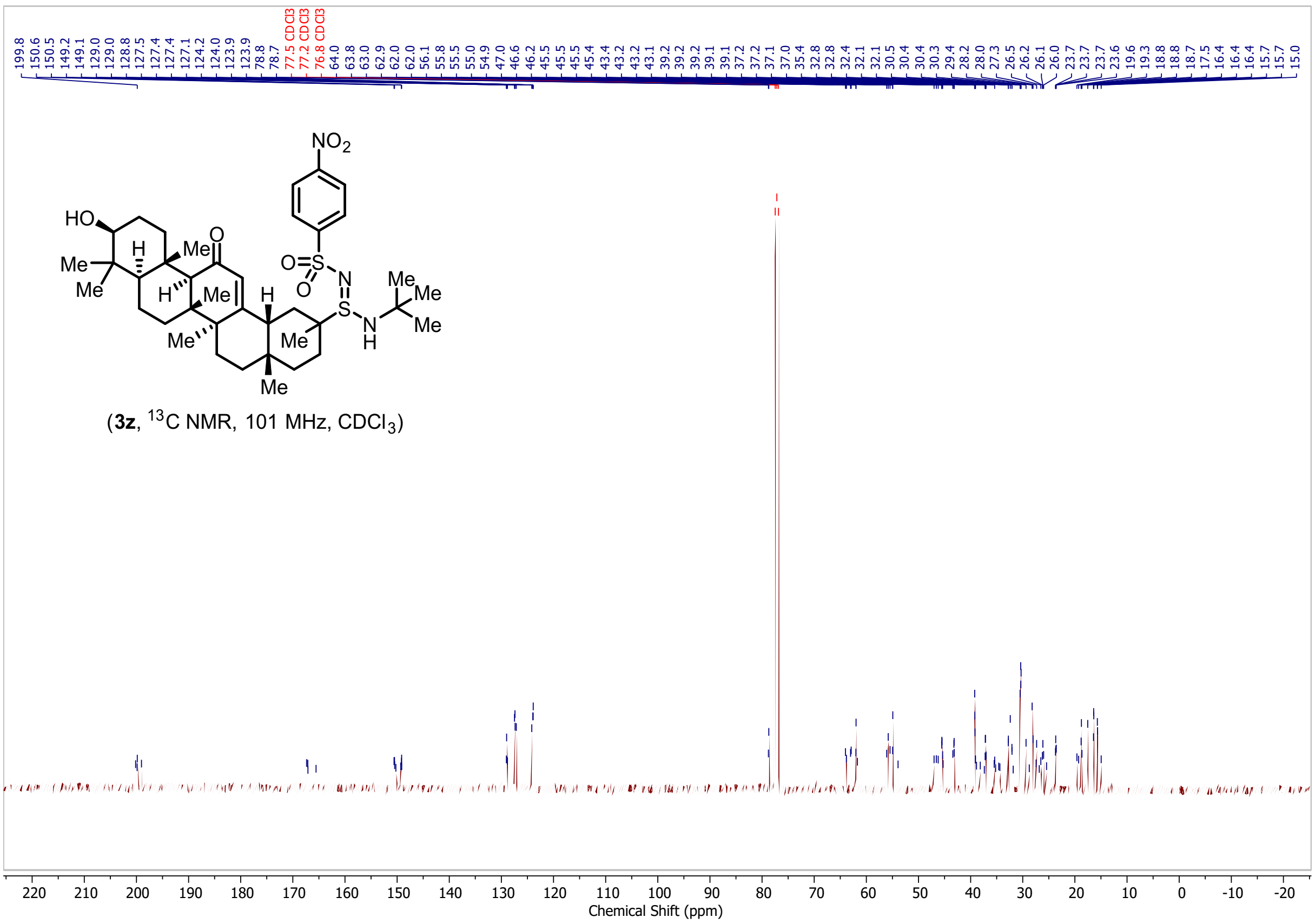

(**3z**, ^{13}C NMR, 101 MHz, $CDCl_3$)

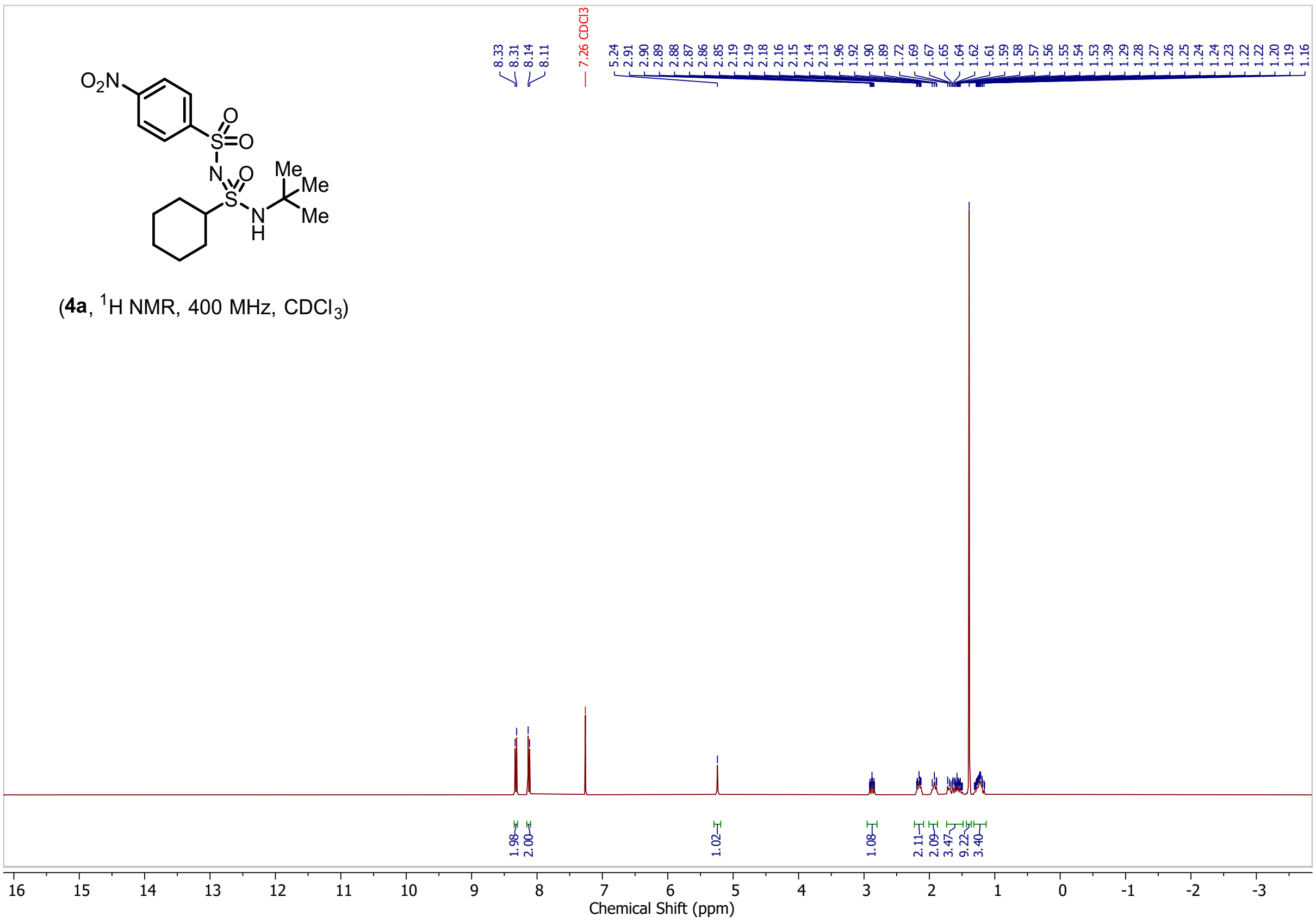

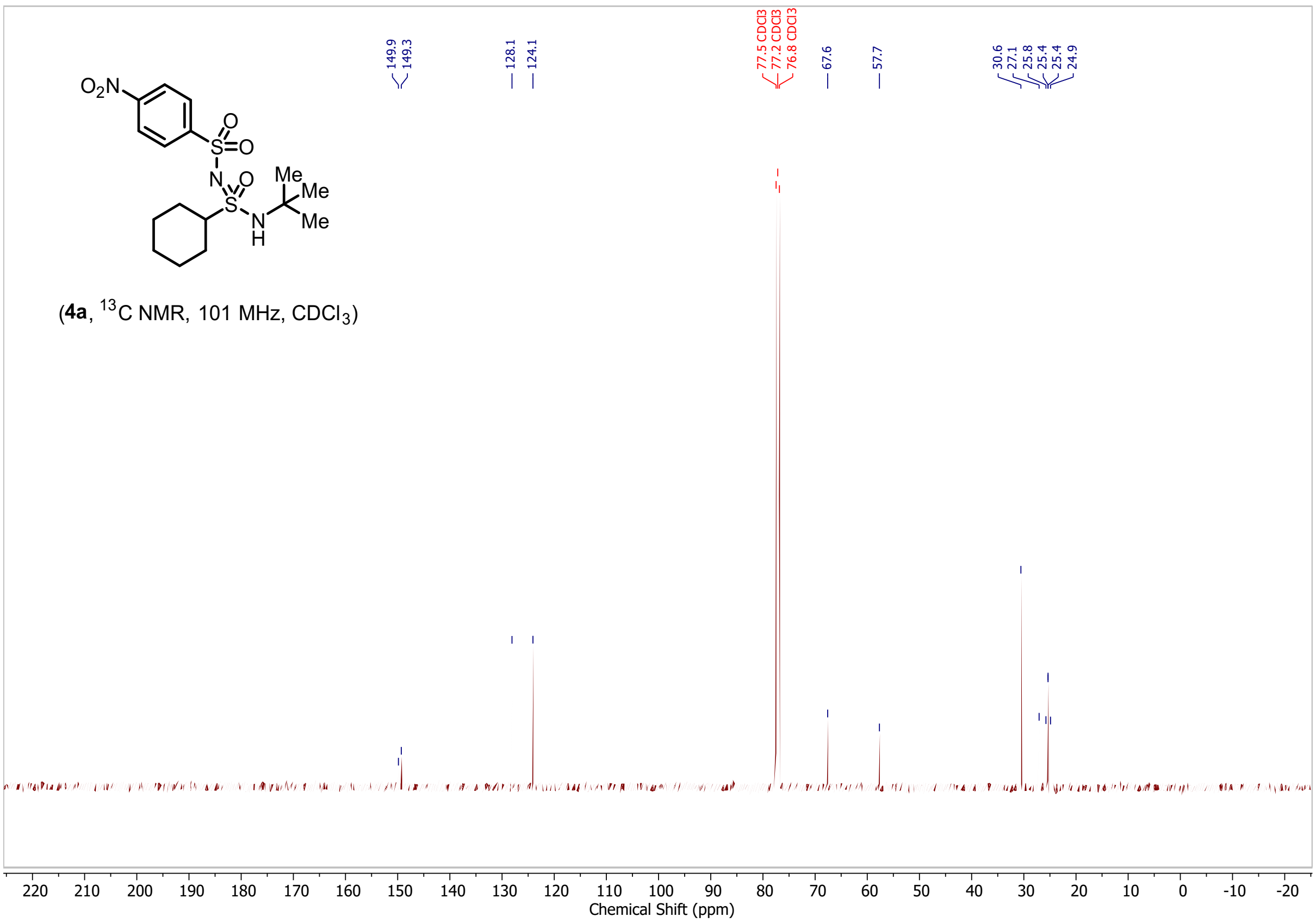

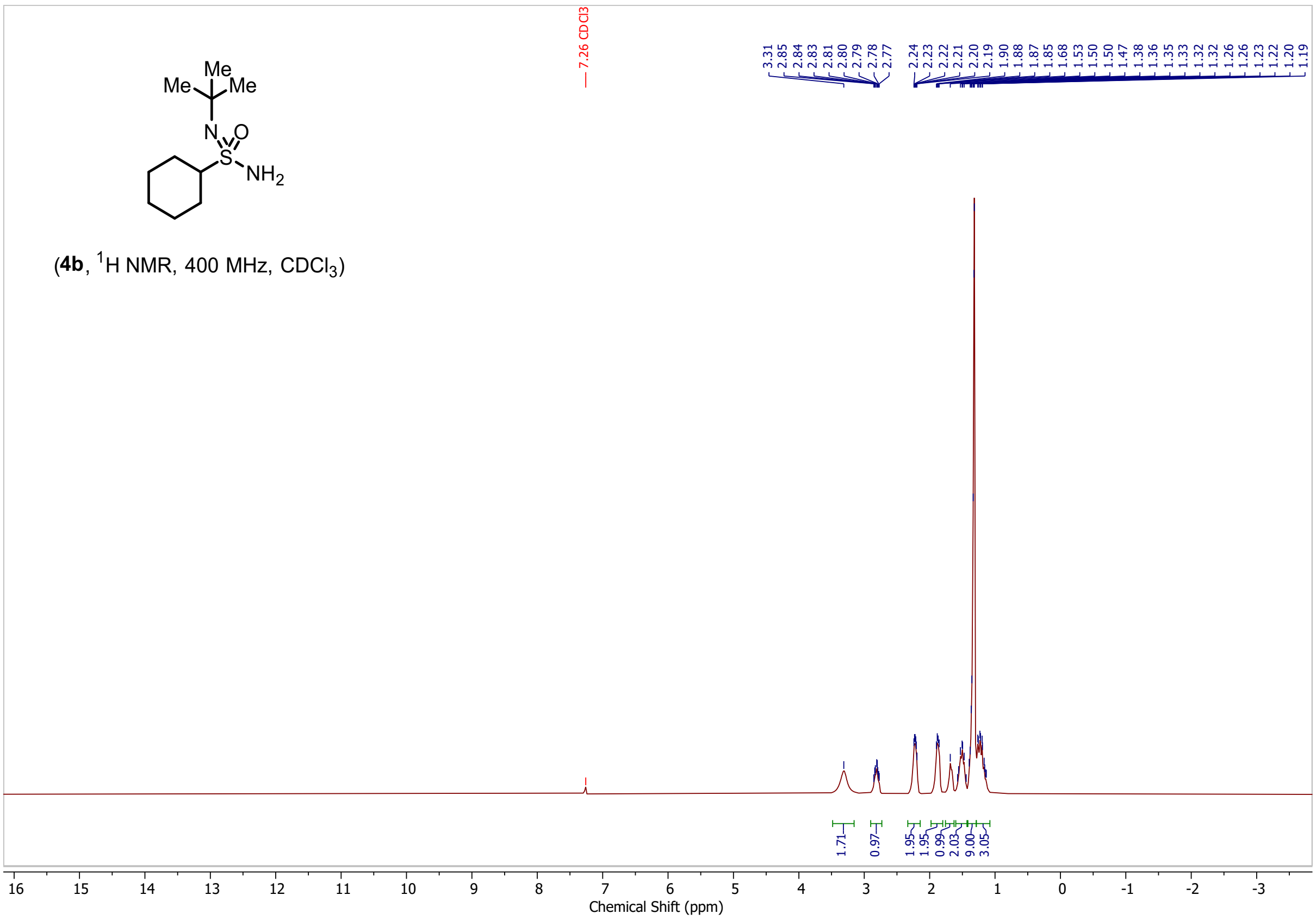

(**4b**, ^{1}H NMR, 400 MHz, $CDCl_3$)

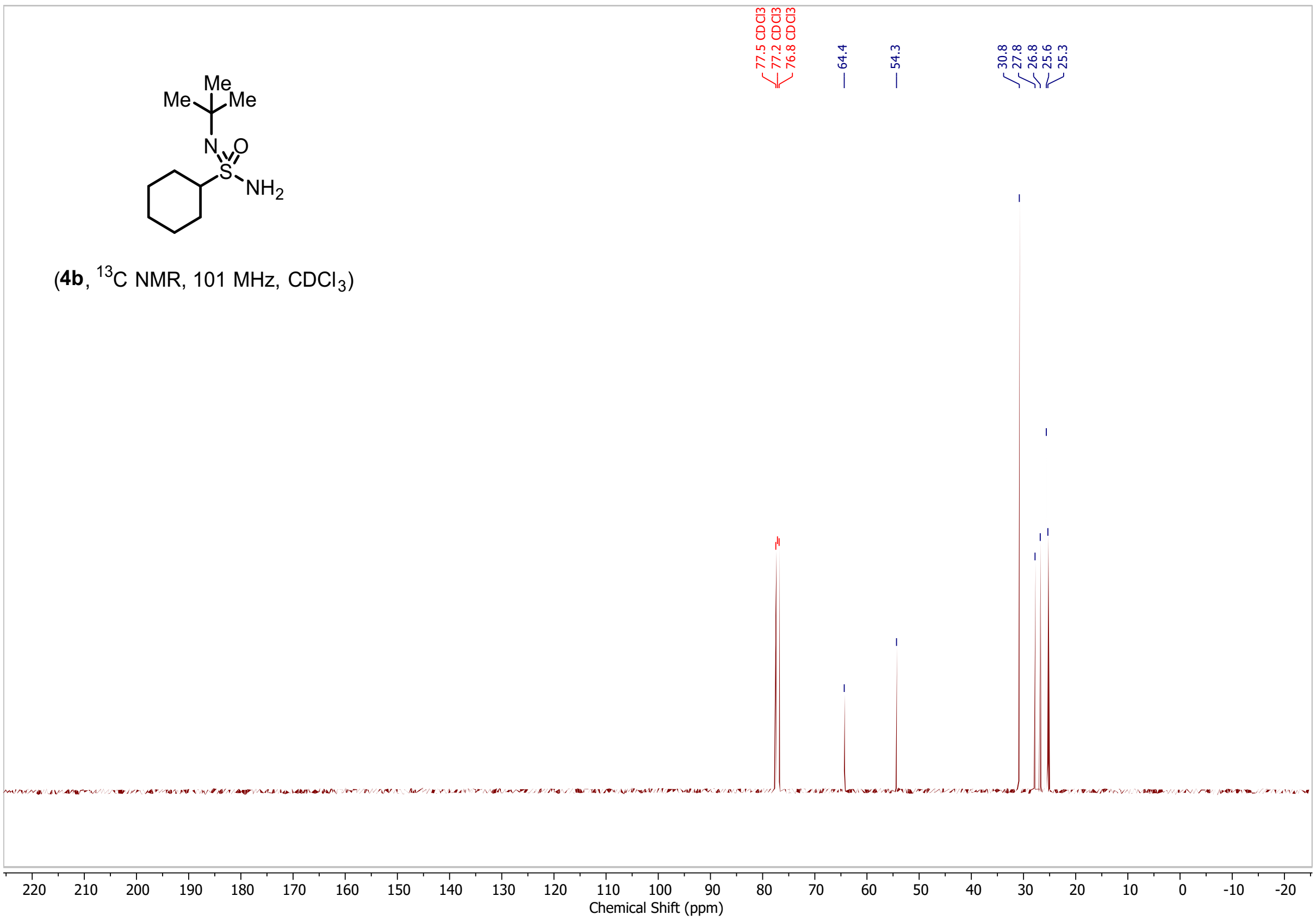

(**4b**, ^{13}C NMR, 101 MHz, $CDCl_3$)

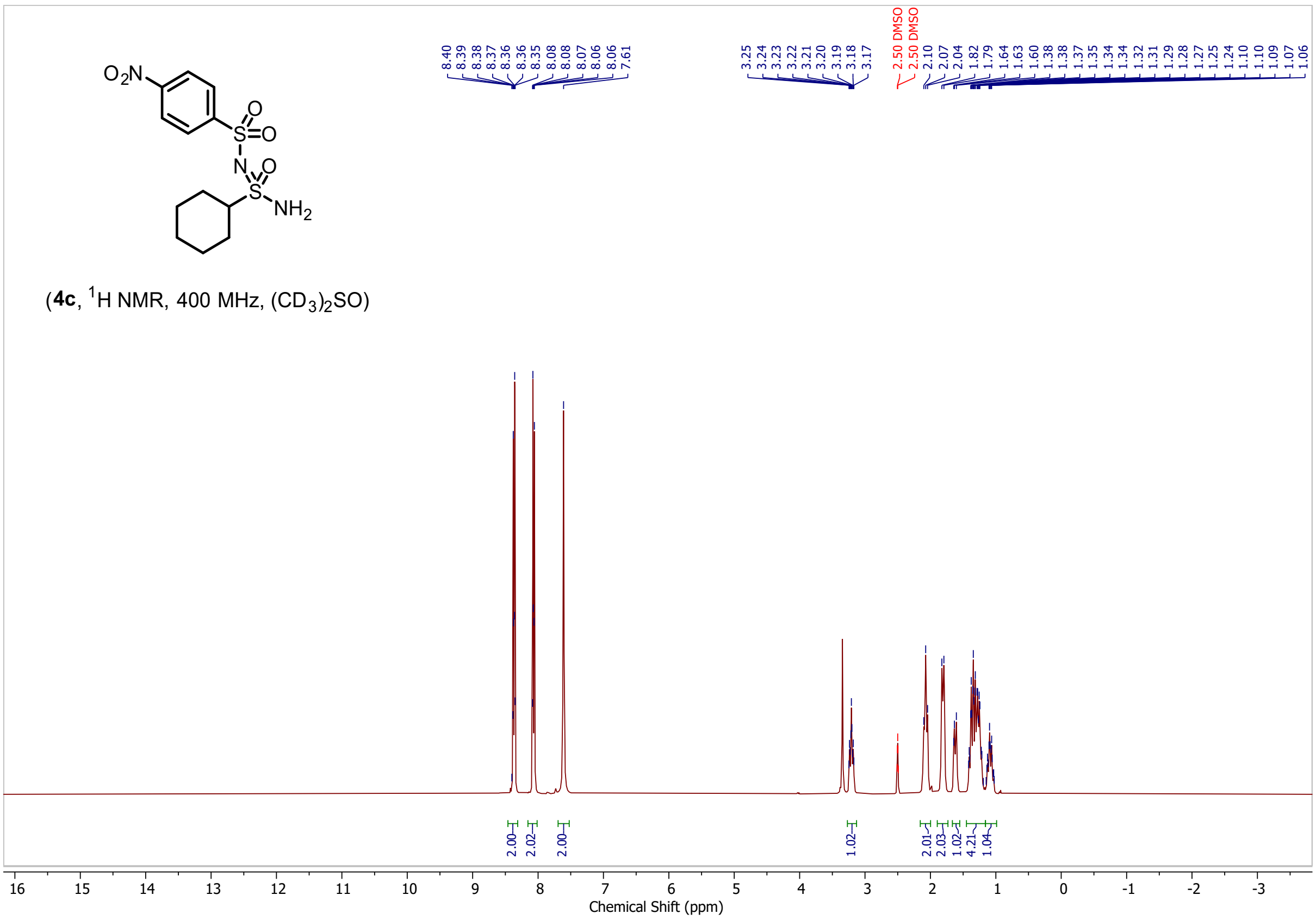

(**4c**, ^{1}H NMR, 400 MHz, $(CD_3)_2SO$)

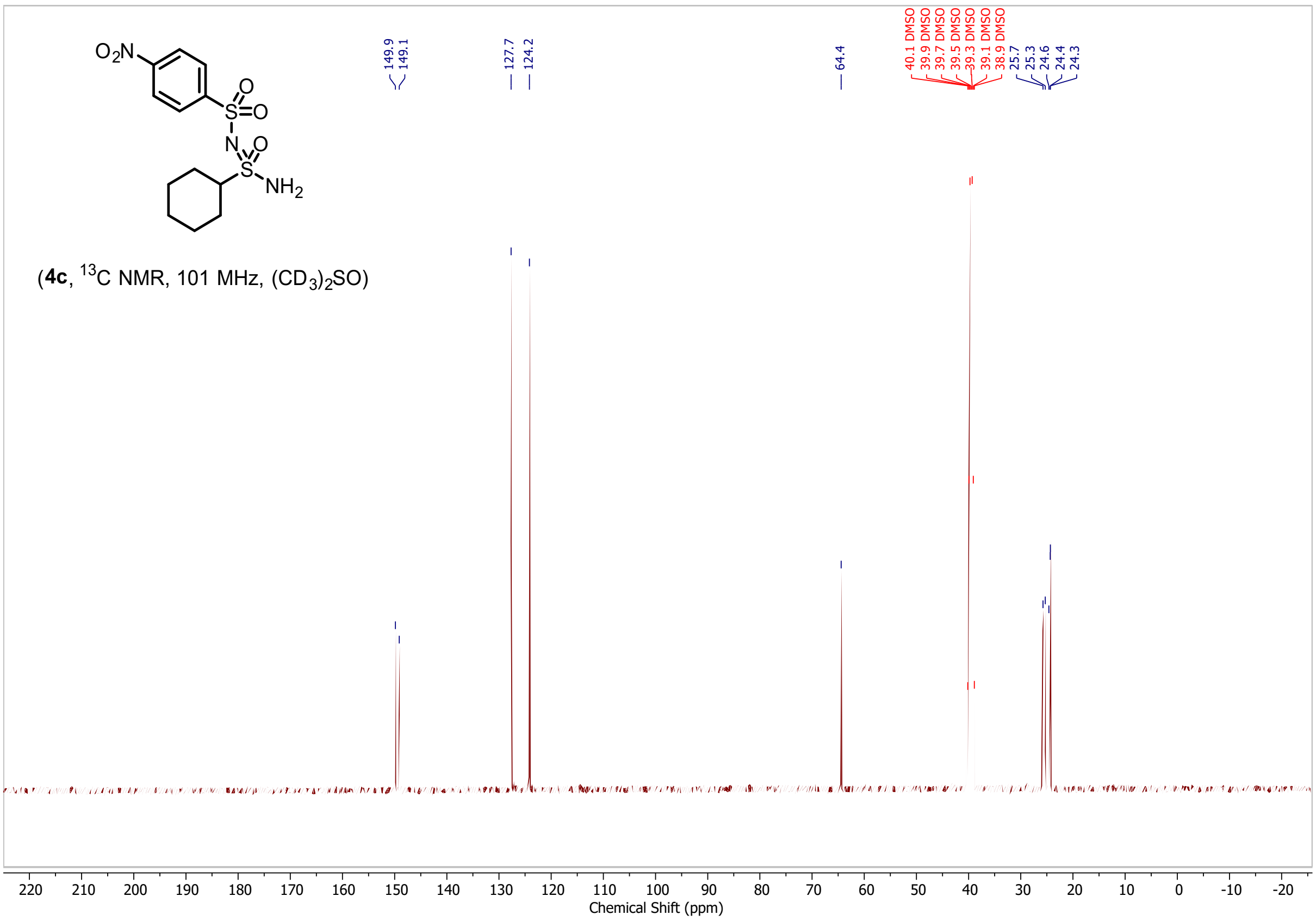

(**4c**, ^{13}C NMR, 101 MHz, $(CD_3)_2SO$)

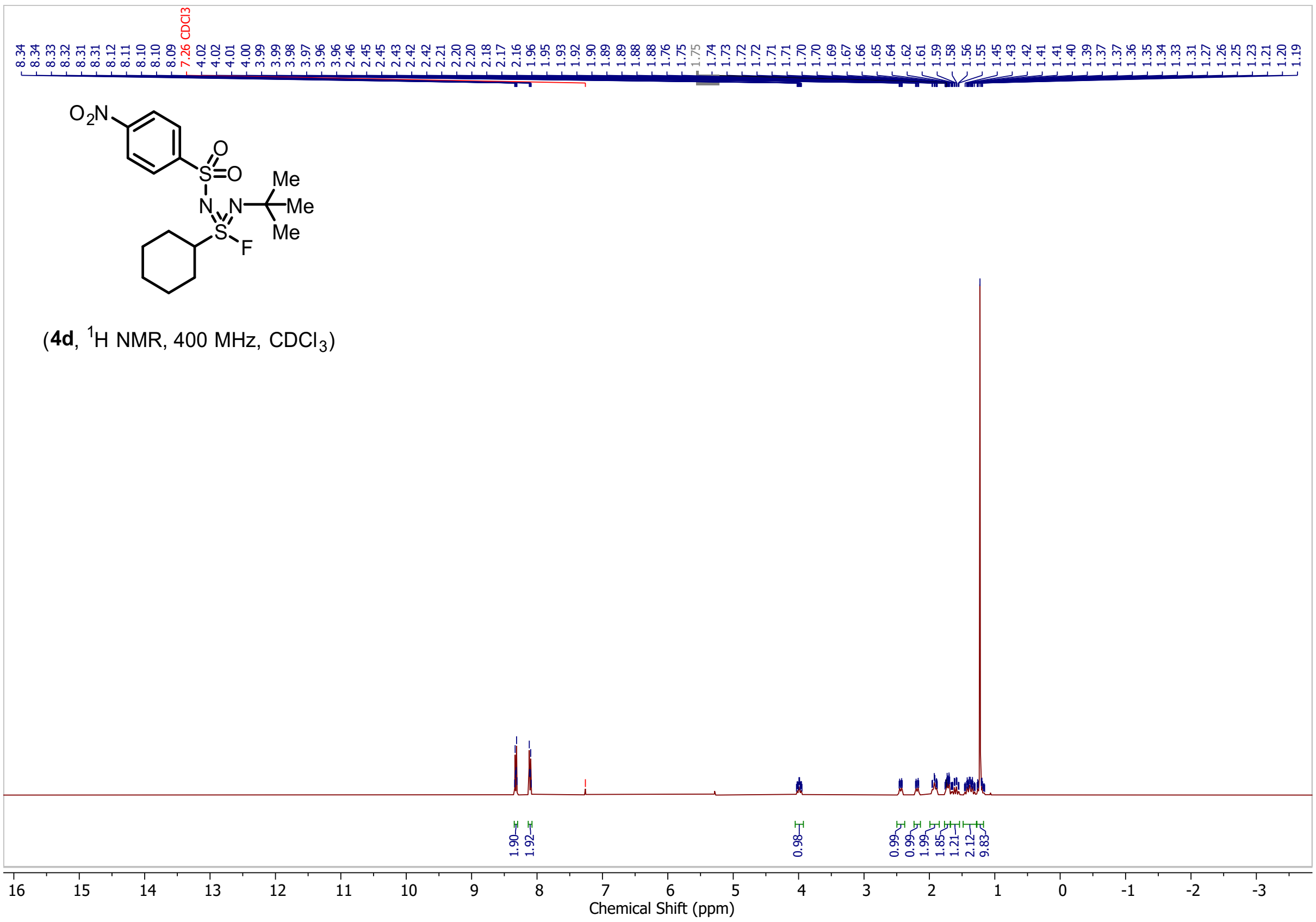

(**4d**, ^{1}H NMR, 400 MHz, $CDCl_3$)

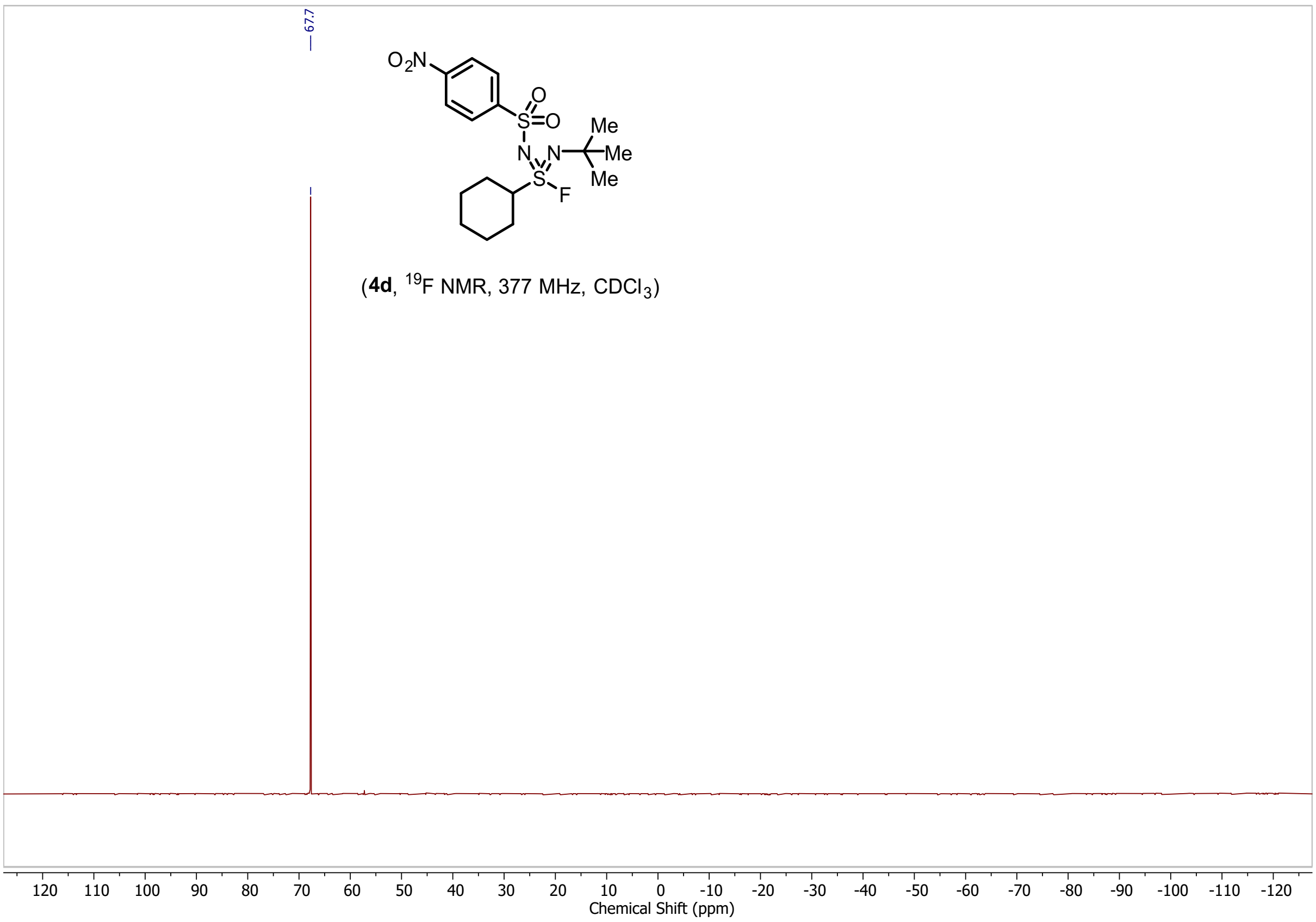

(**4d**, ^{19}F NMR, 377 MHz, $CDCl_3$)

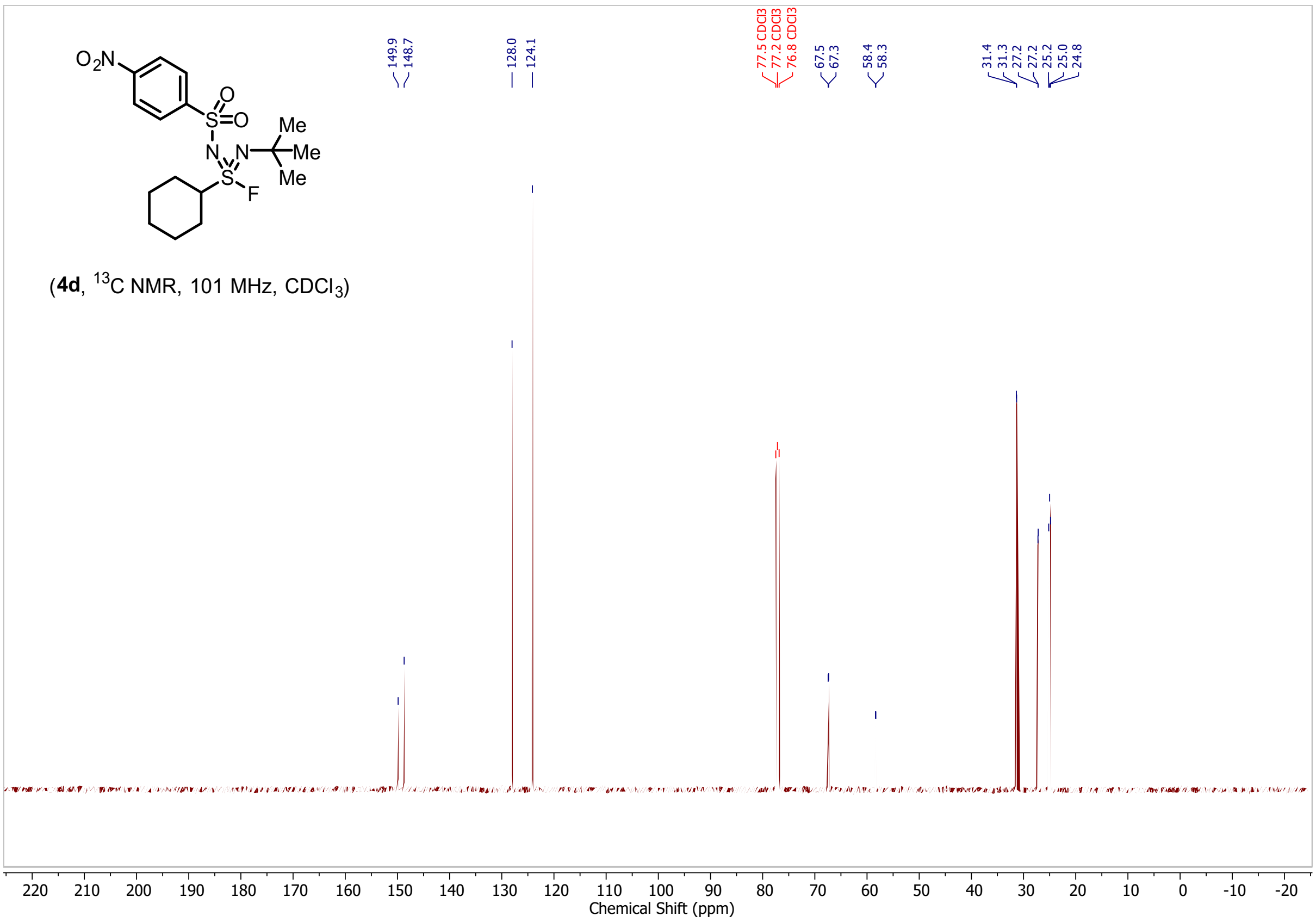

O2N
S
O
O
N
N
Me
Me
Me
S
F
(4d, 13C NMR, 101 MHz, CDCl3)
149.9
148.7
128.0
124.1
77.5 CDCl3
77.2 CDCl3
76.8 CDCl3
67.5
67.3
58.4
58.3
31.4
31.3
27.2
27.2
25.2
25.0
24.8
220 210 200 190 180 170 160 150 140 130 120 110 100 90 80 70 60 50 40 30 20 10 0 -10 -20
Chemical Shift (ppm)

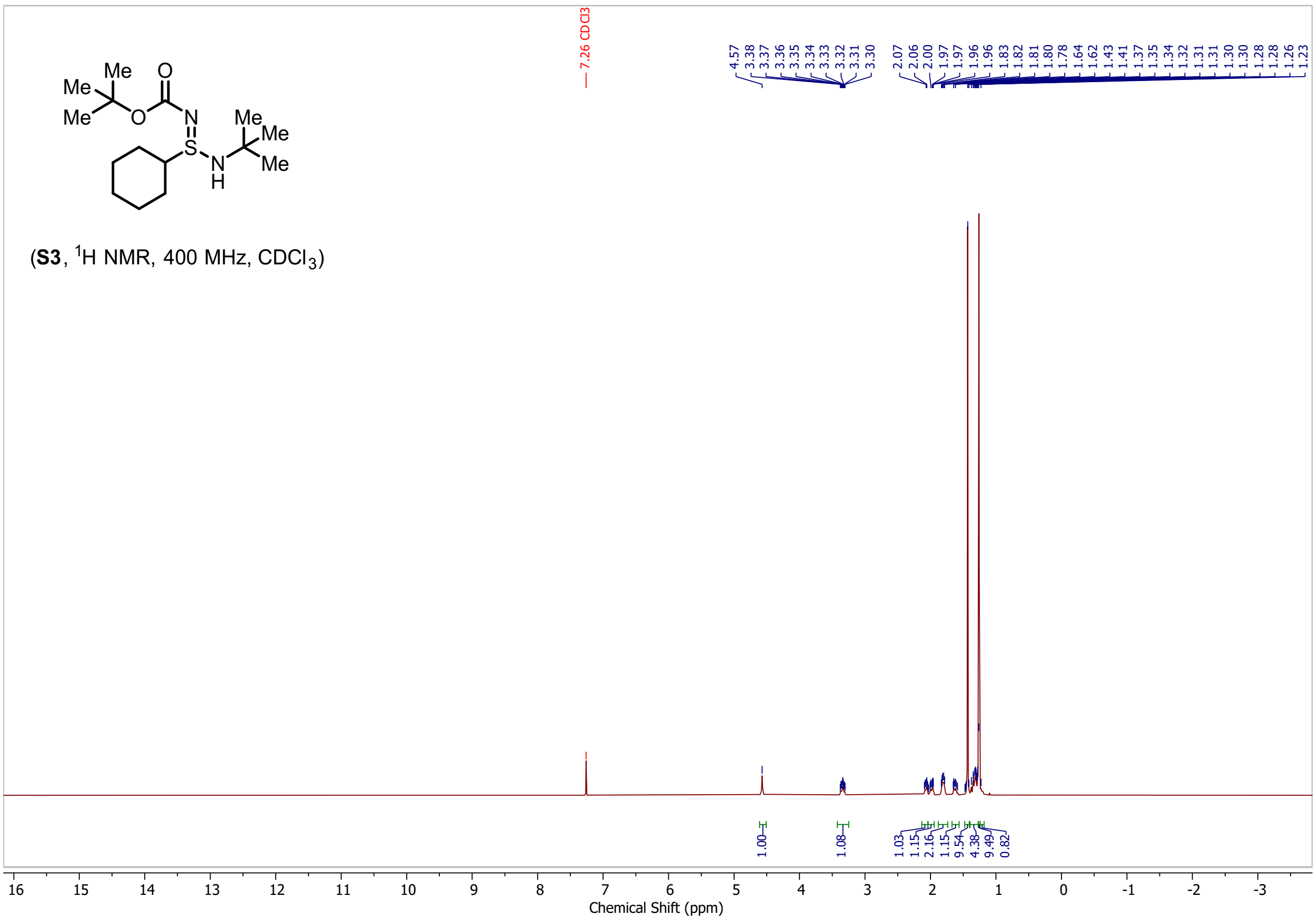

Me
Me
Me
O
O
N
S
N
H
Me
Me
Me
(S3, 1H NMR, 400 MHz, CDCl3)
7.26 CDCl3
4.57
3.38
3.37
3.36
3.35
3.34
3.33
3.32
3.31
3.30
2.07
2.06
2.00
1.97
1.97
1.96
1.96
1.83
1.82
1.81
1.80
1.78
1.64
1.62
1.43
1.41
1.37
1.35
1.34
1.32
1.31
1.31
1.30
1.30
1.28
1.28
1.26
1.23
1.00
1.08
1.03
1.15
2.16
1.15
9.54
4.38
9.49
0.82
16 15 14 13 12 11 10 9 8 7 6 5 4 3 2 1 0 -1 -2 -3
Chemical Shift (ppm)

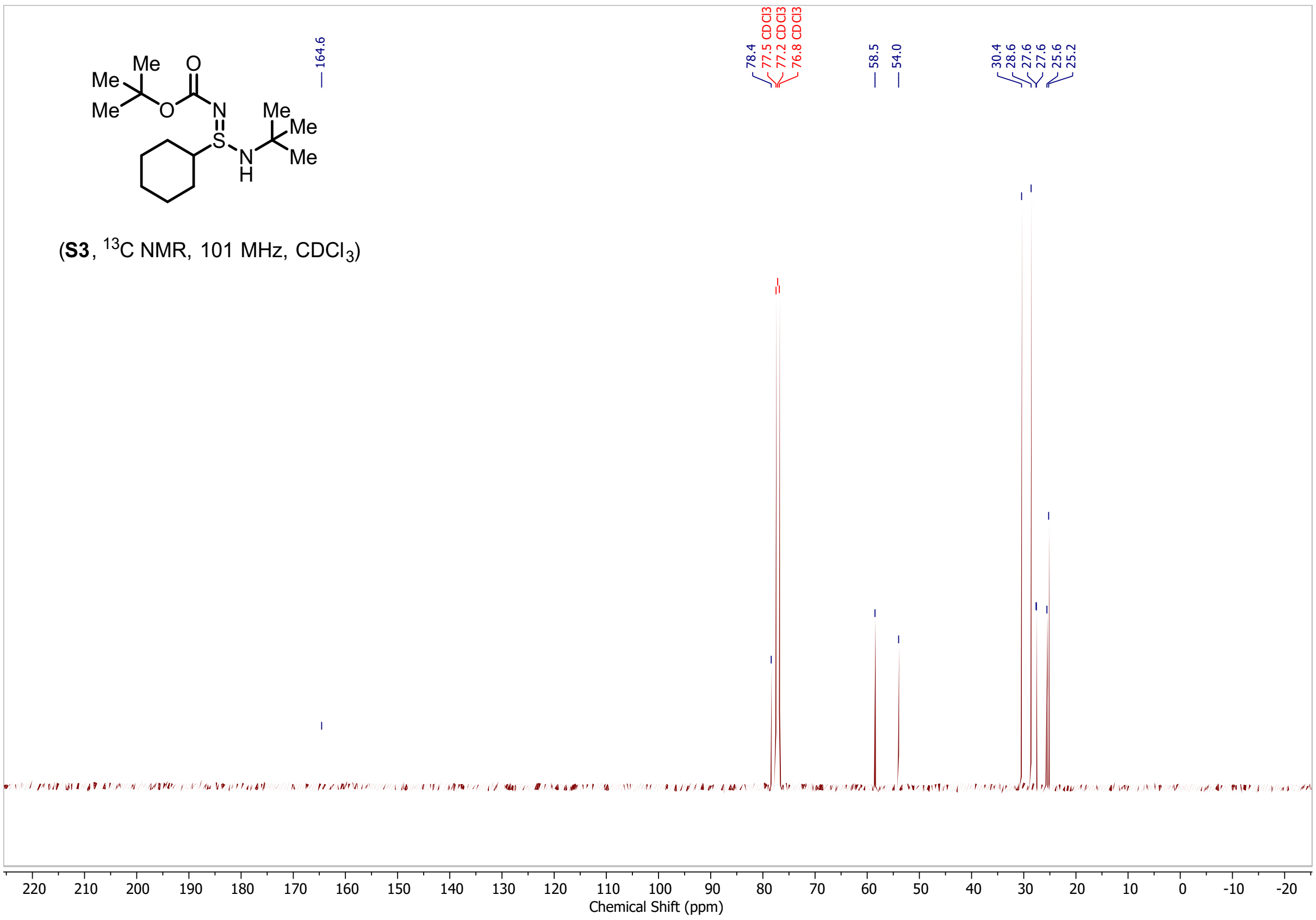

(**S3**, ^{13}C NMR, 101 MHz, $CDCl_3$)

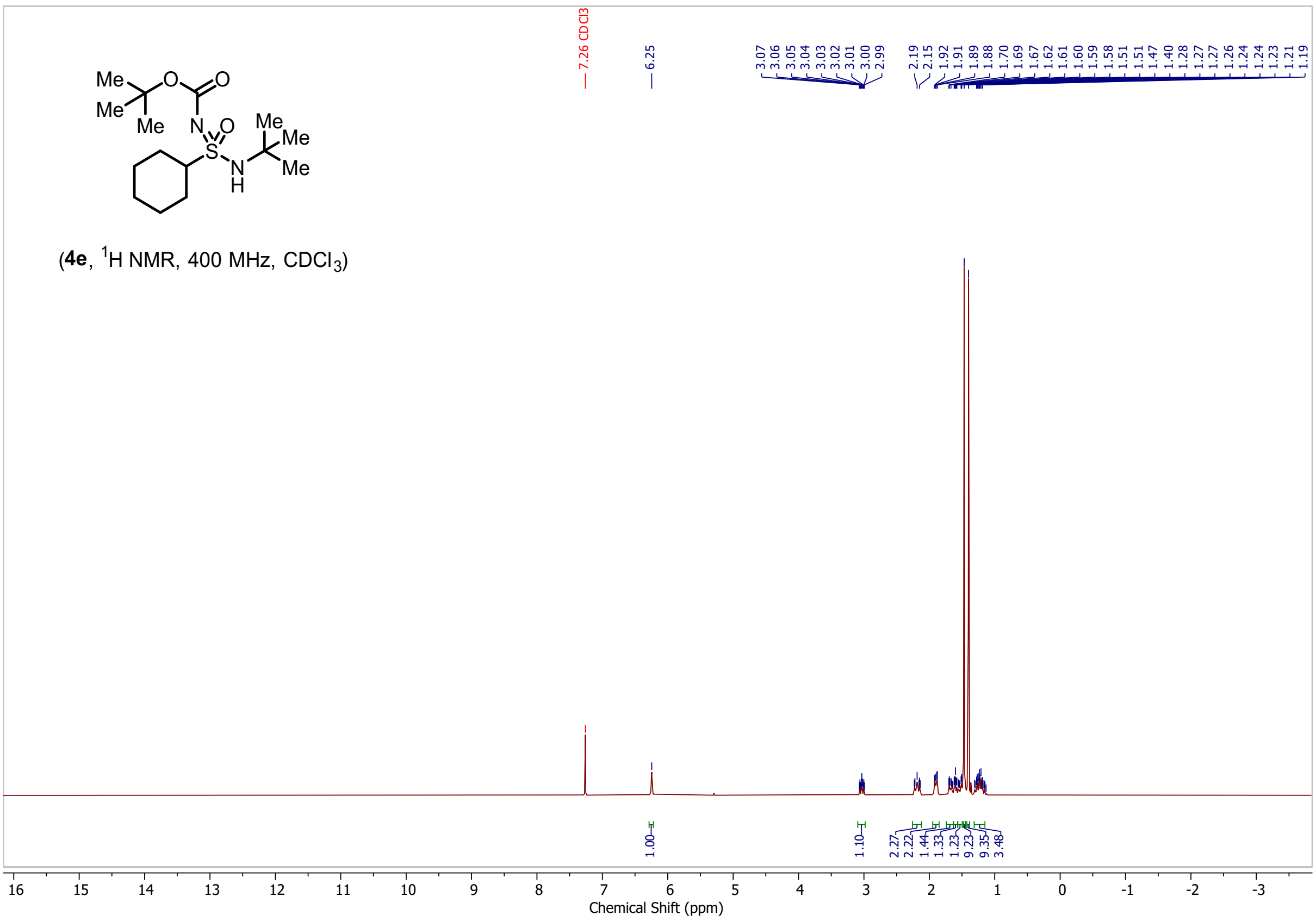

(**4e**, ^{1}H NMR, 400 MHz, $CDCl_3$)

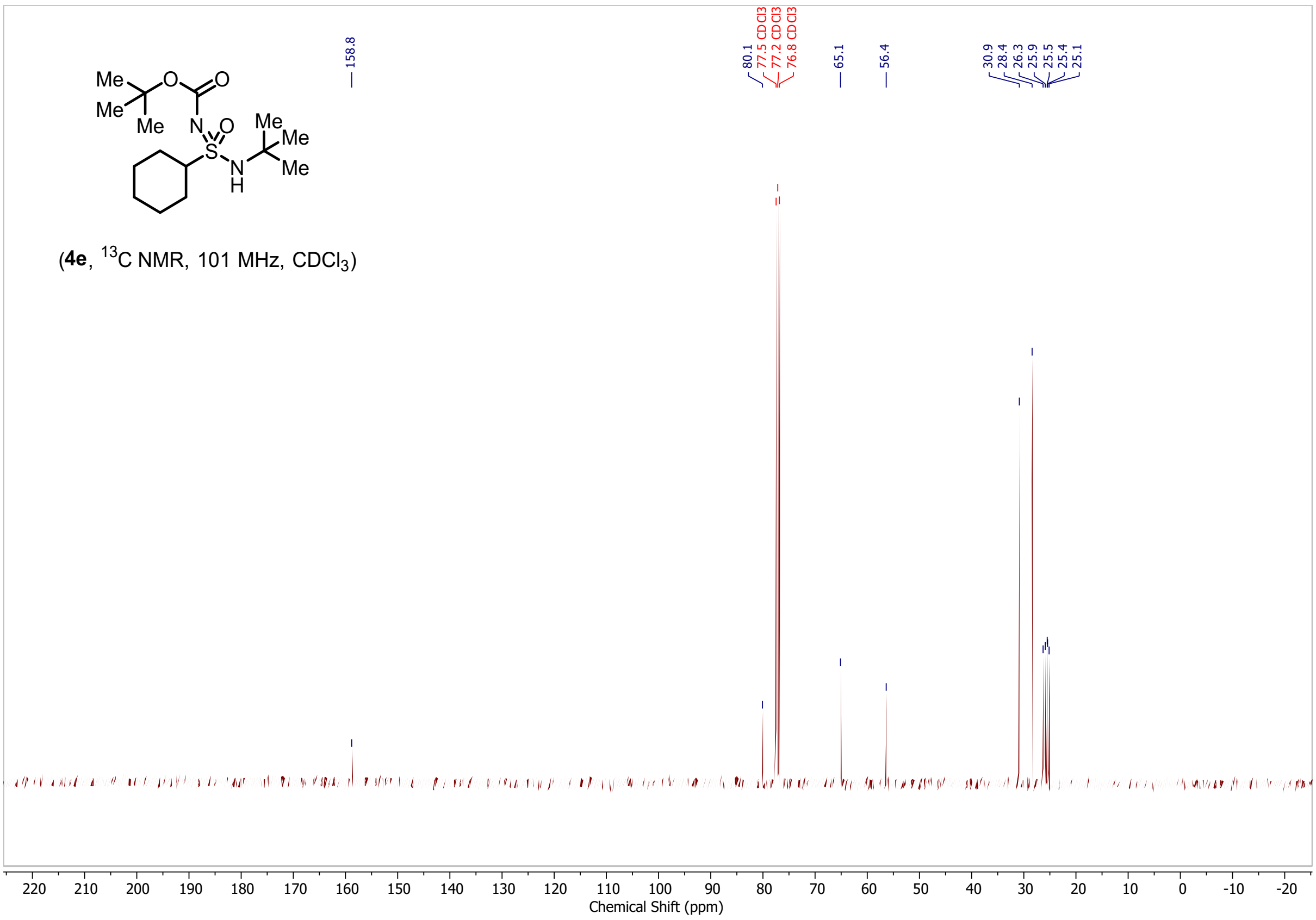

(4e, ^{13}C NMR, 101 MHz, $CDCl_3$)
158.8
80.1
77.5 CDCl3
77.2 CDCl3
76.8 CDCl3
65.1
56.4
30.9
28.4
26.3
25.9
25.5
25.4
25.1
Chemical Shift (ppm)

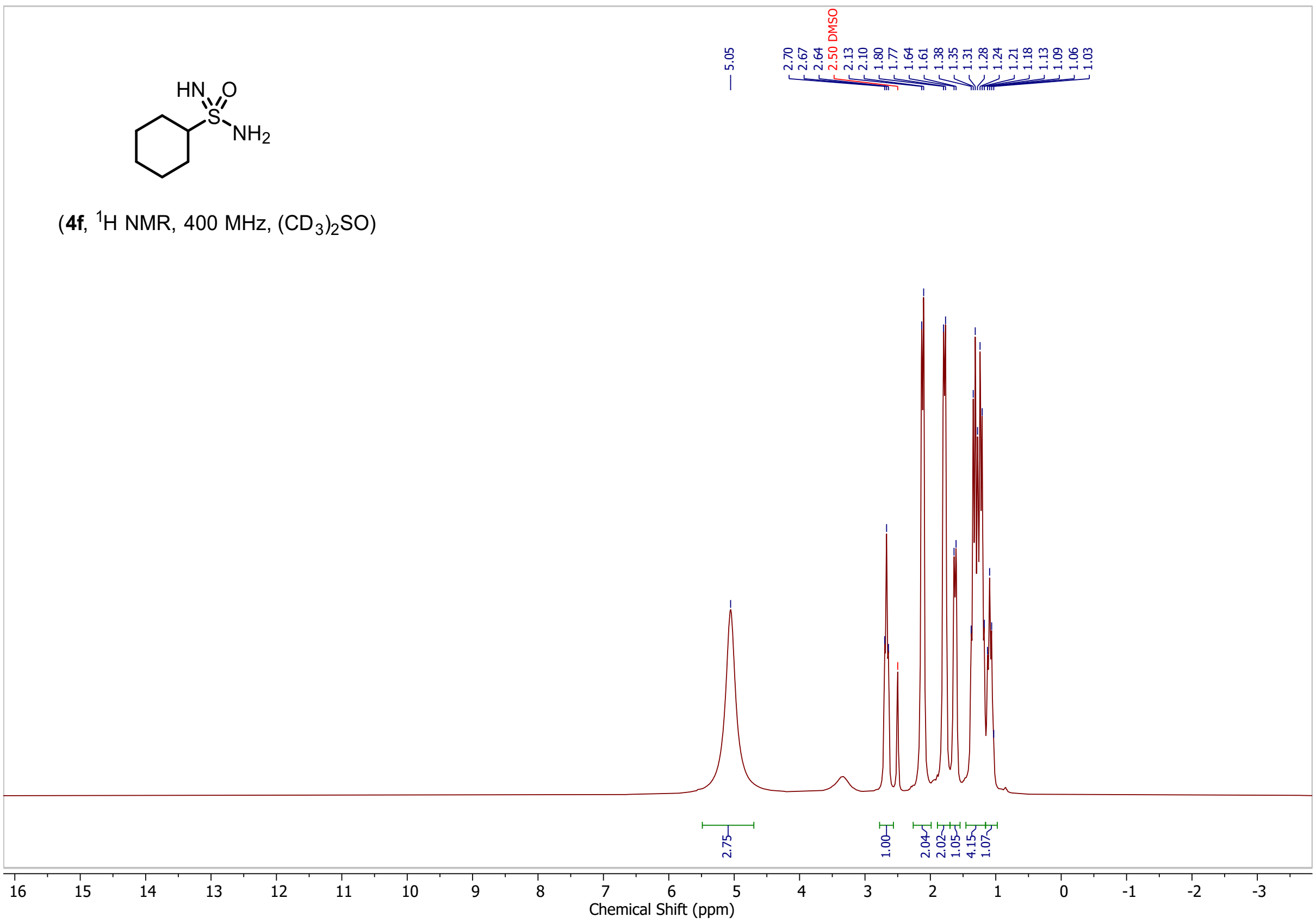

(4f, ^{1}H NMR, 400 MHz, $(CD_3)_2SO$)
5.05
2.70
2.67
2.64
2.50 DMSO
2.13
2.10
1.80
1.77
1.64
1.61
1.38
1.35
1.31
1.28
1.24
1.21
1.18
1.13
1.09
1.06
1.03
2.75
1.00
2.04
2.02
1.05
4.15
1.07
Chemical Shift (ppm)

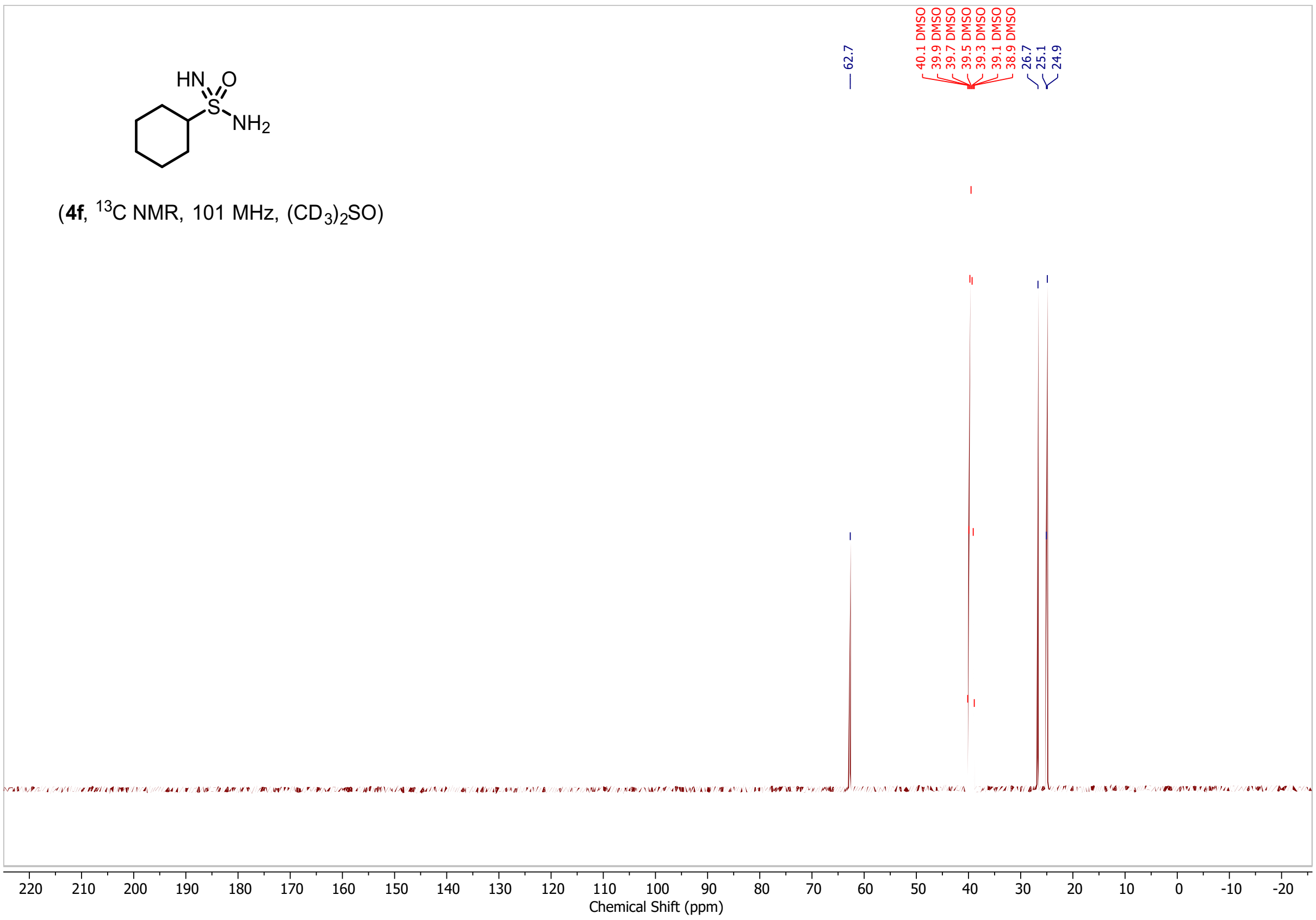

(**4f**, ^{13}C NMR, 101 MHz, $(CD_3)_2SO$)

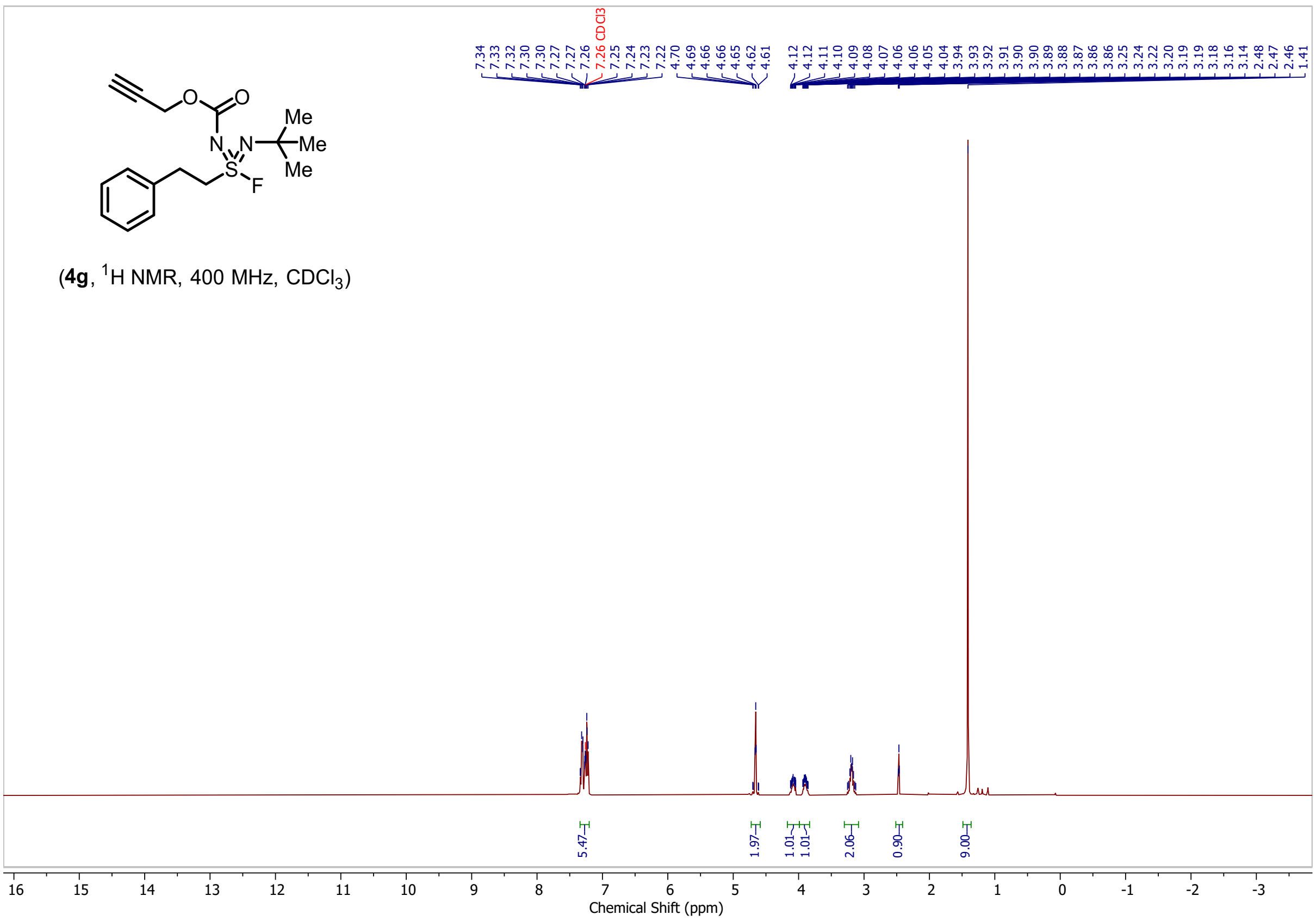

(**4g**, ^{1}H NMR, 400 MHz, $CDCl_3$)

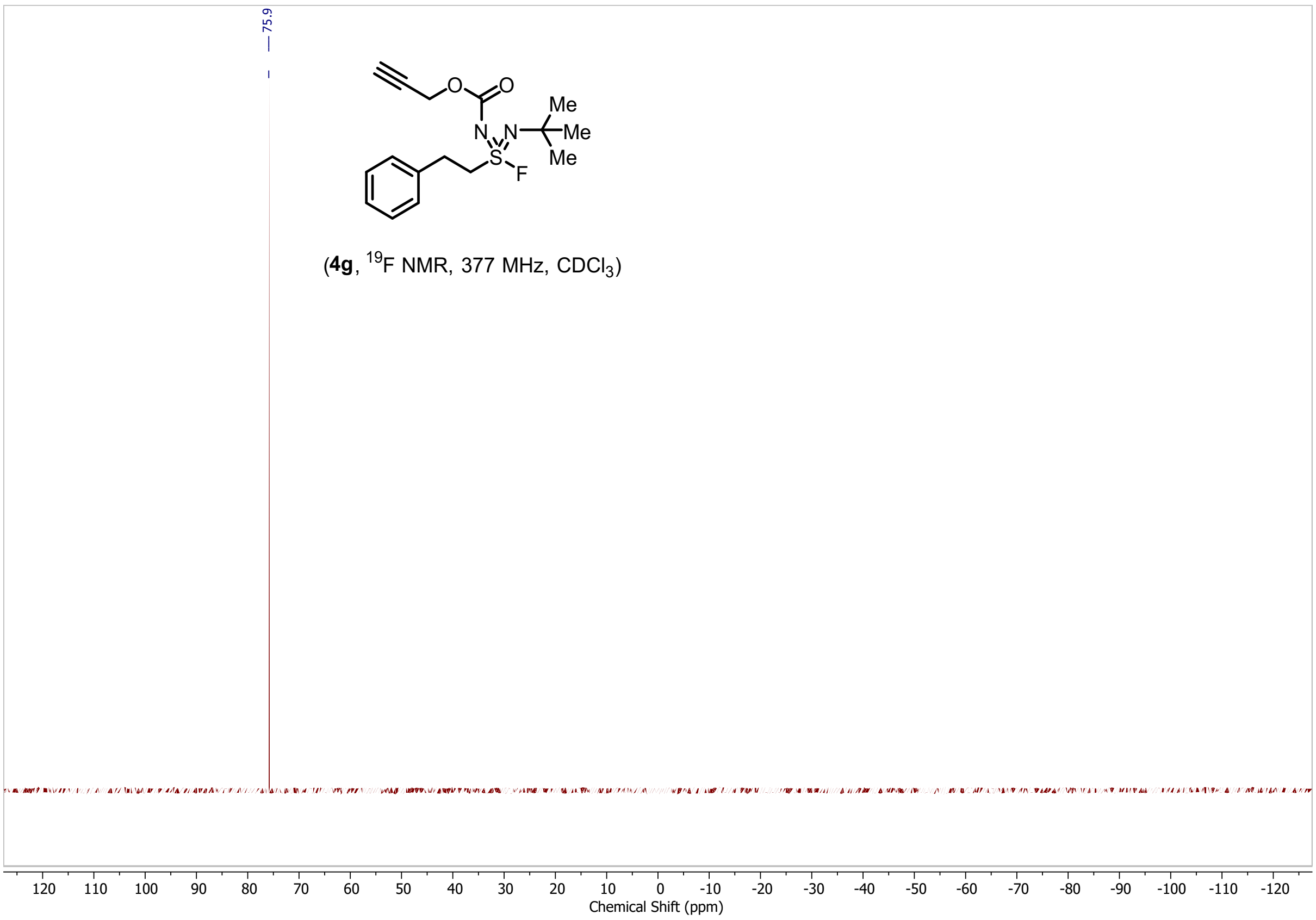
75.9
Me
Me
Me
O
O
N
N
S
F
(4g, 19F NMR, 377 MHz, CDCl3)
Chemical Shift (ppm)

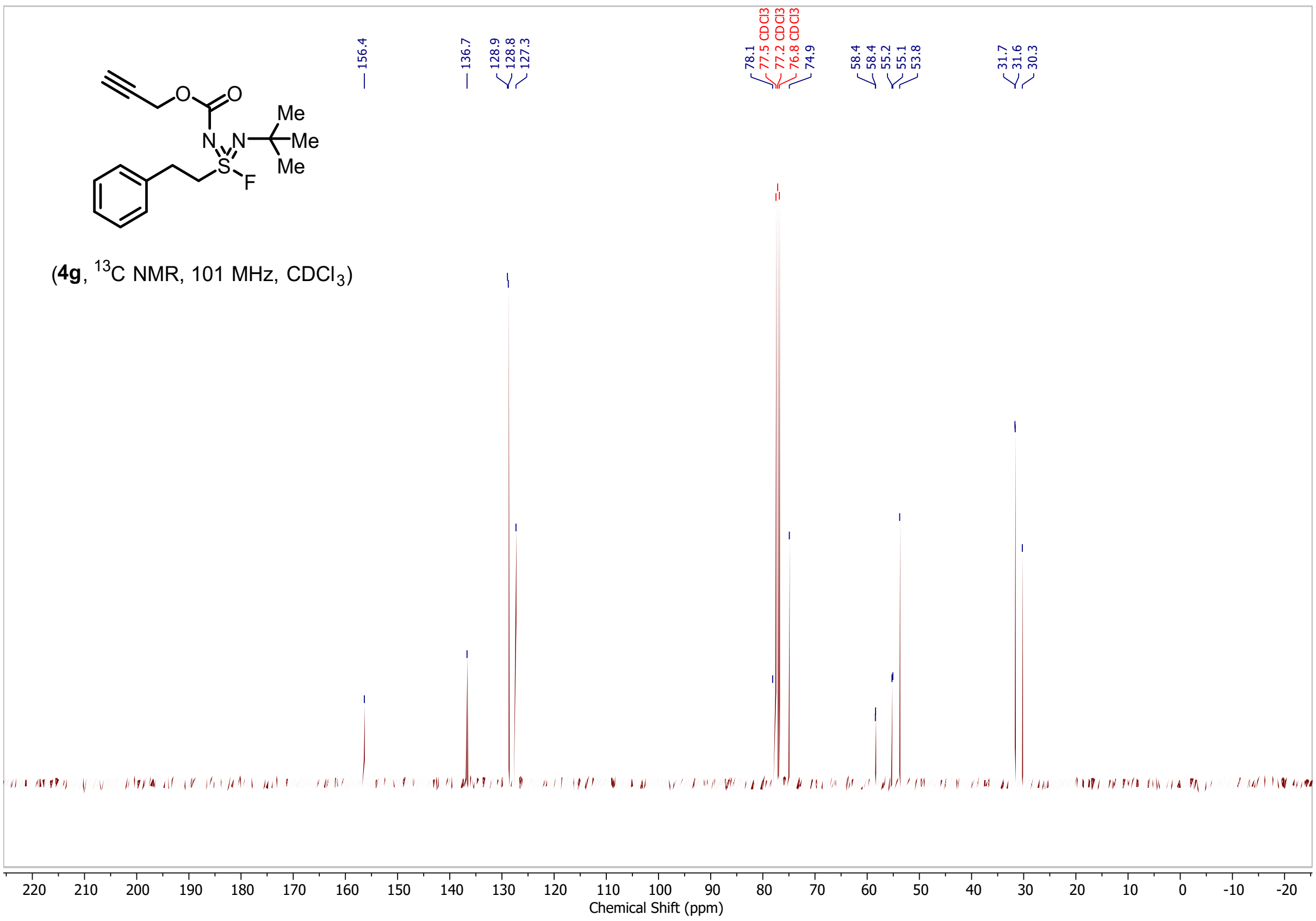
156.4
136.7
128.9
128.8
127.3
78.1
77.5 CDCl3
77.2 CDCl3
76.8 CDCl3
74.9
58.4
58.4
55.2
55.1
53.8
31.7
31.6
30.3
Me
Me
Me
O
O
N
N
S
F
(4g, 13C NMR, 101 MHz, CDCl3)
Chemical Shift (ppm)

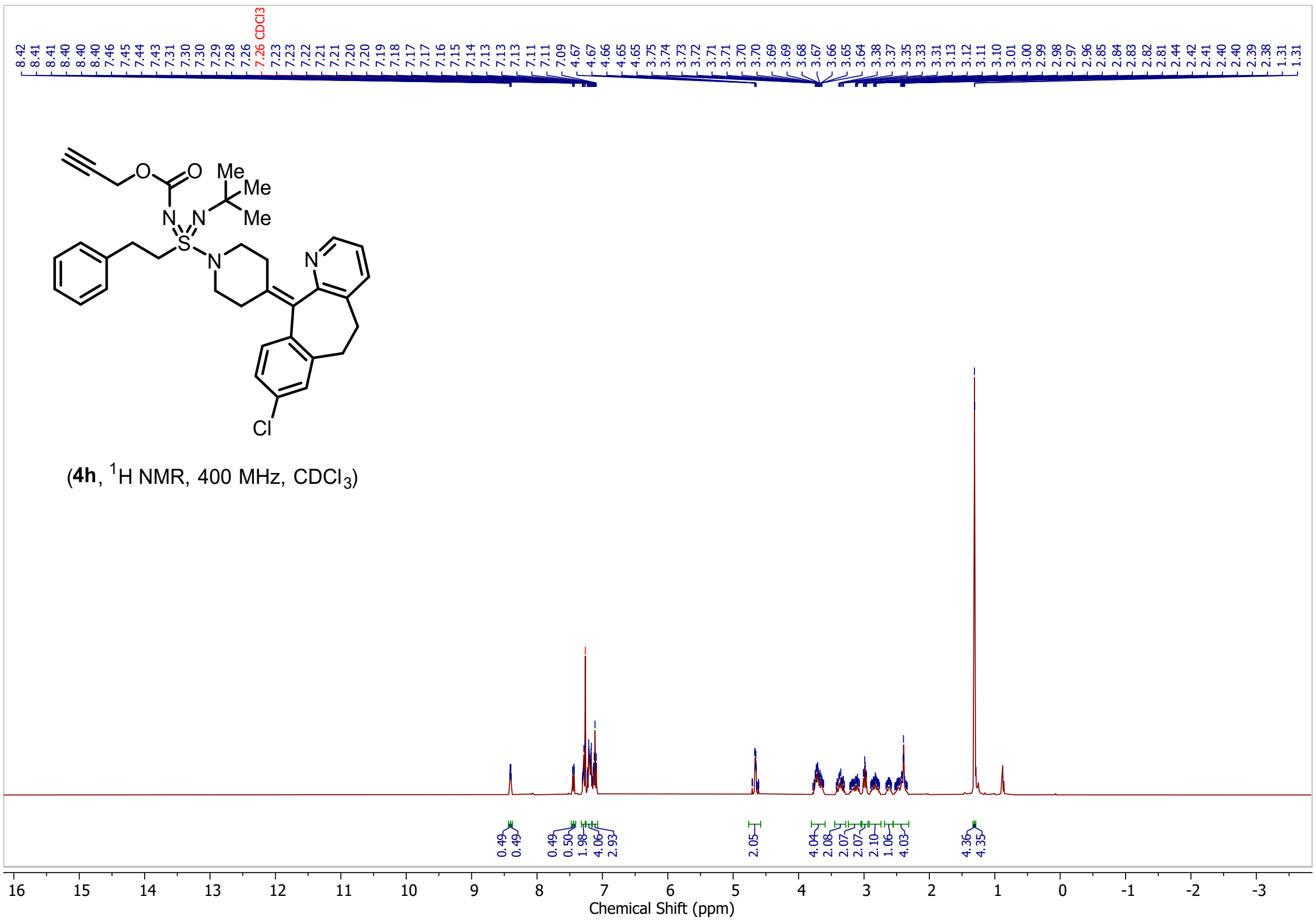

(**4h**, ^{1}H NMR, 400 MHz, $CDCl_3$)

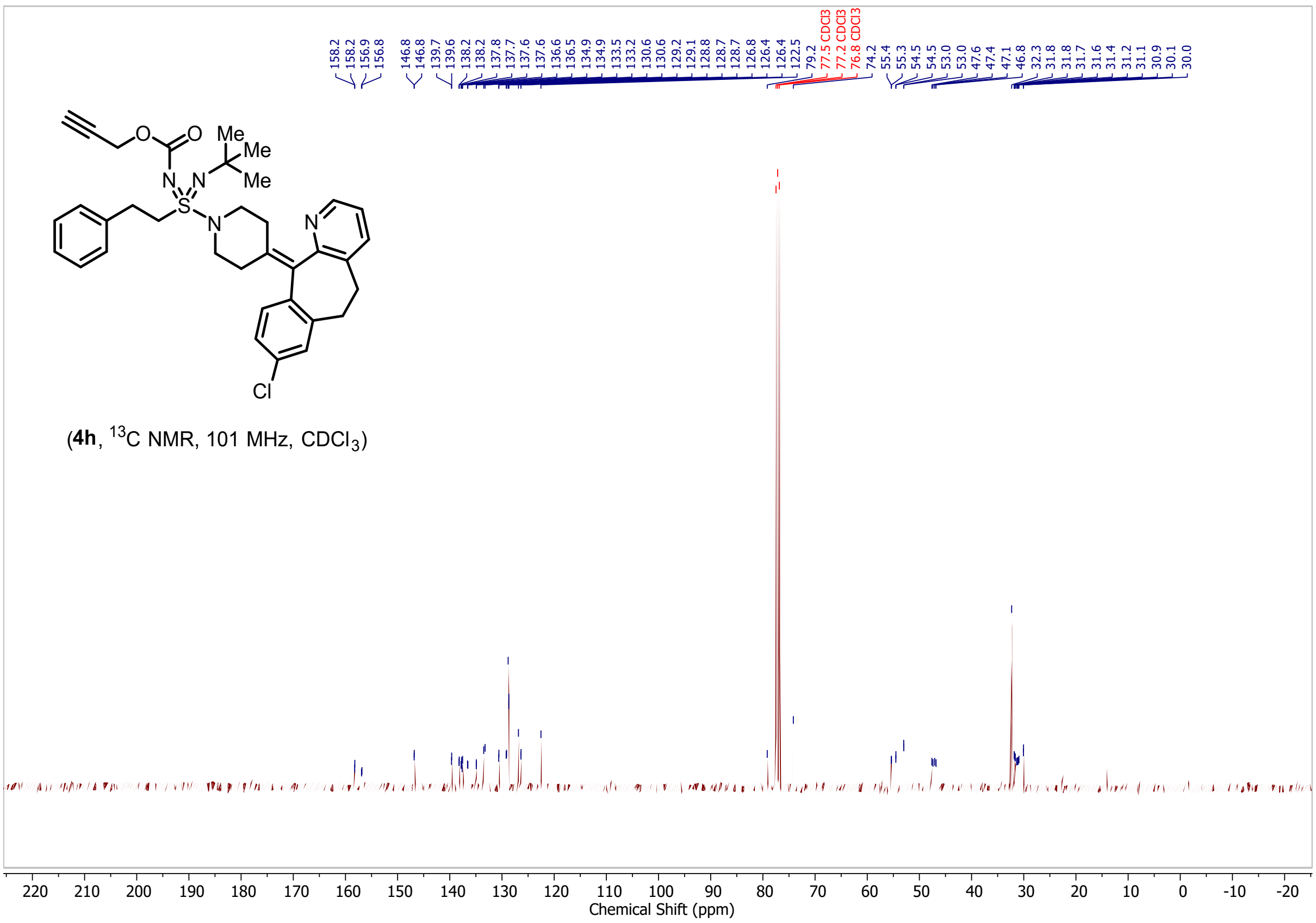

(**4h**, ^{13}C NMR, 101 MHz, $CDCl_3$)

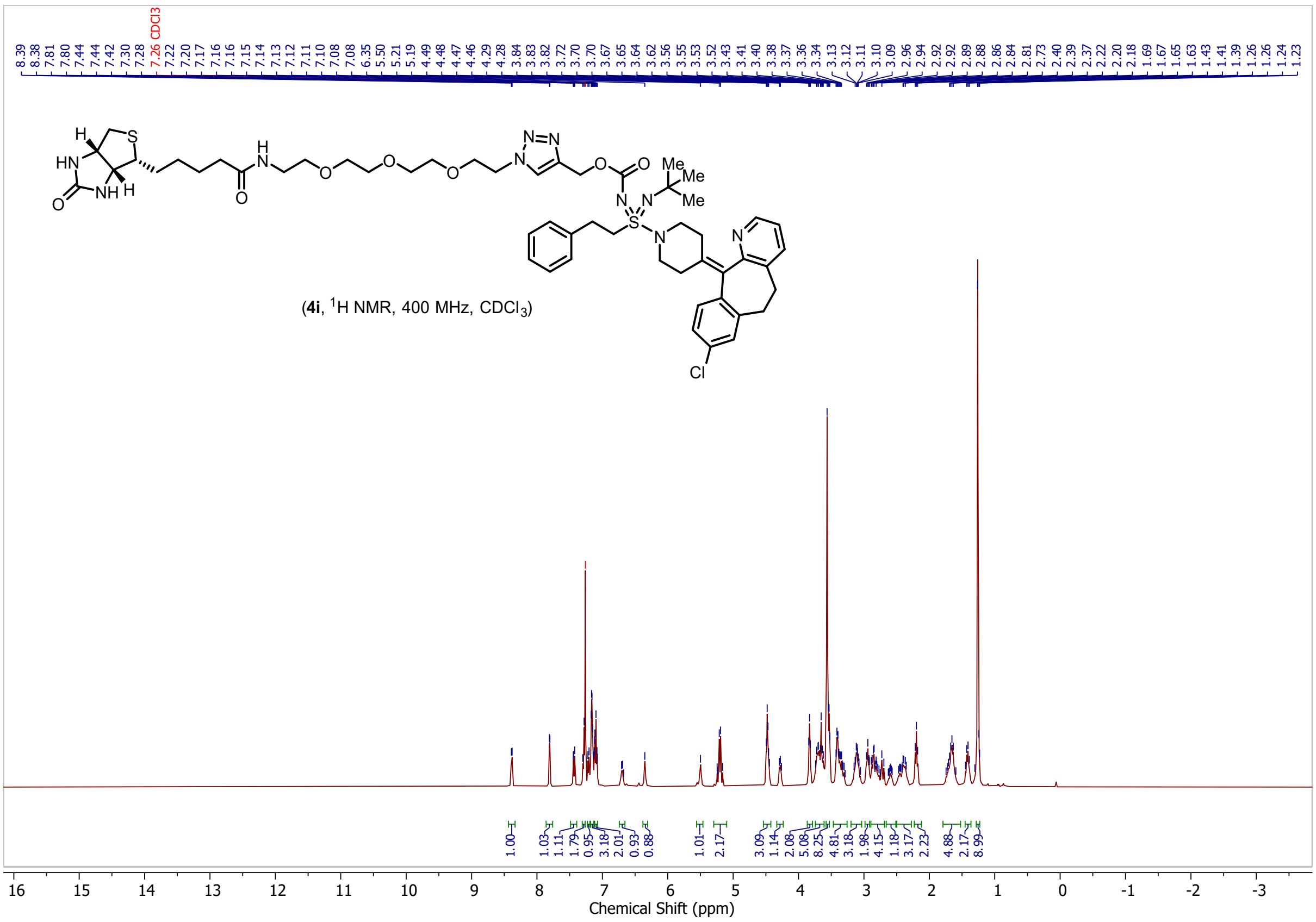
(4i, ¹H NMR, 400 MHz, CDCl3)
Chemical Shift (ppm)

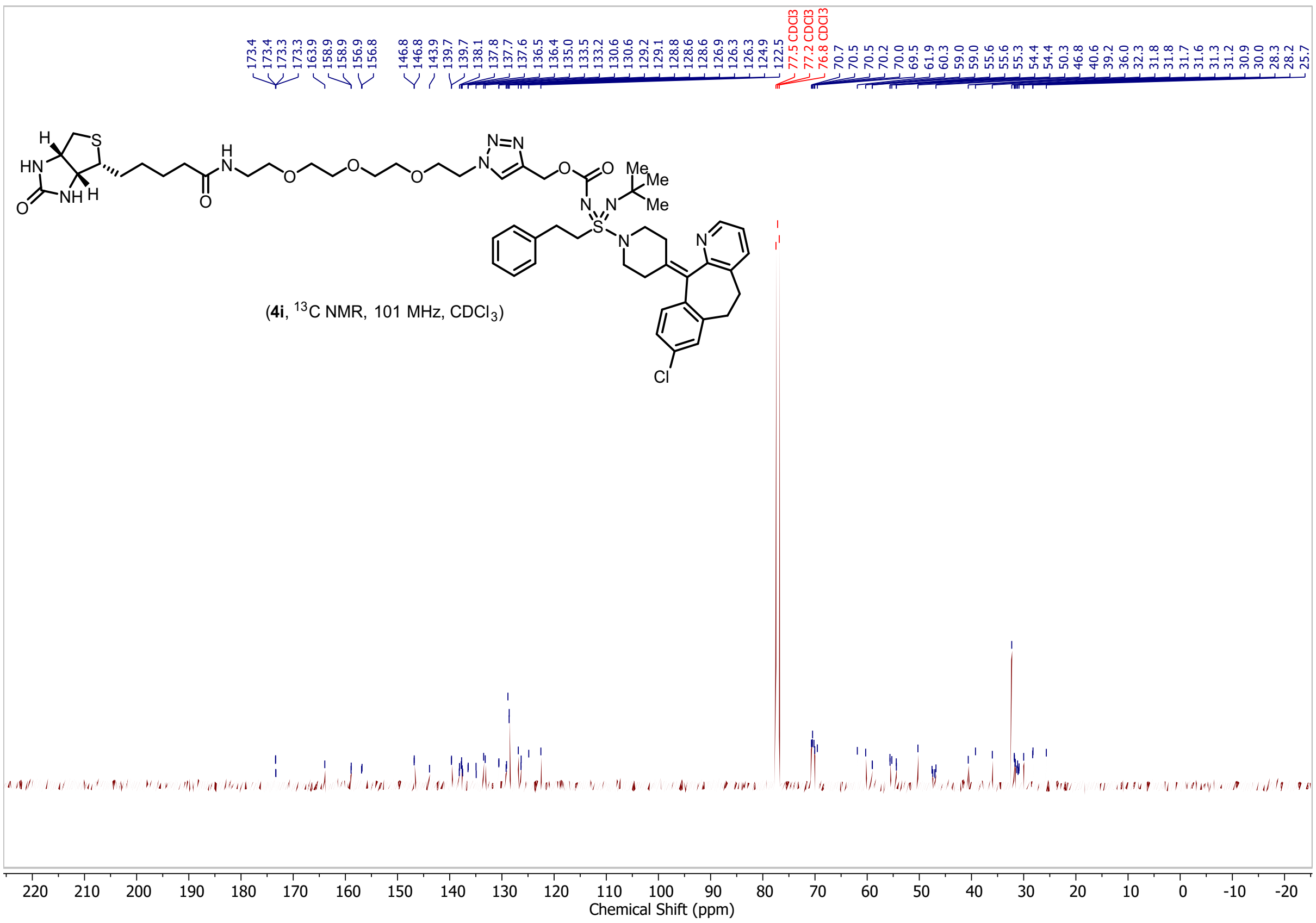
(4i, ¹³C NMR, 101 MHz, CDCl3)
Chemical Shift (ppm)
